# Supplementary material for: Solution structure and pressure response of thioredoxin-1 of Plasmodium falciparum
Source: PLoS One. 2024 Apr 18;19(4):e0301579. doi: 10.1371/journal.pone.0301579 (PMC11025842; doi:10.1371/journal.pone.0301579)
Supplement: S2 File — (PDF) [file pone.0301579.s002.pdf]

Full wwPDB NMR Structure Validation Report (i)

Jun 6, 2023 – 04:09 PM EDT

PDB ID	:	2MMO
BMRB ID	:	16147
Title	:	Solution Structure of the oxidised Thioredoxin from Plasmodium falciparum
Authors	:	Munte, C.; Kalbitzer, H.; Schirmer, R.
Deposited on	:	2014-03-16

This is a Full wwPDB NMR Structure Validation Report for a publicly released PDB entry.

We welcome your comments at *validation@mail.wwpdb.org*
A user guide is available at
https://www.wwpdb.org/validation/2017/NMRValidationReportHelp
with specific help available everywhere you see the (i) symbol.

The types of validation reports are described at
http://www.wwpdb.org/validation/2017/FAQs#types.

The following versions of software and data (see references (i)) were used in the production of this report:

MolProbity	:	4.02b-467
Percentile statistics	:	20191225.v01 (using entries in the PDB archive December 25th 2019)
wwPDB-RCI	:	v_1n_11_5_13_A (Berjanski et al., 2005)
PANAV	:	Wang et al. (2010)
wwPDB-ShiftChecker	:	v1.2
BMRB Restraints Analysis	:	v1.2
Ideal geometry (proteins)	:	Engh & Huber (2001)
Ideal geometry (DNA, RNA)	:	Parkinson et al. (1996)
Validation Pipeline (wwPDB-VP)	:	2.33

1 Overall quality at a glance (i)

The following experimental techniques were used to determine the structure:
SOLUTION NMR

The overall completeness of chemical shifts assignment is 95%.

Percentile scores (ranging between 0-100) for global validation metrics of the entry are shown in the following graphic. The table shows the number of entries on which the scores are based.

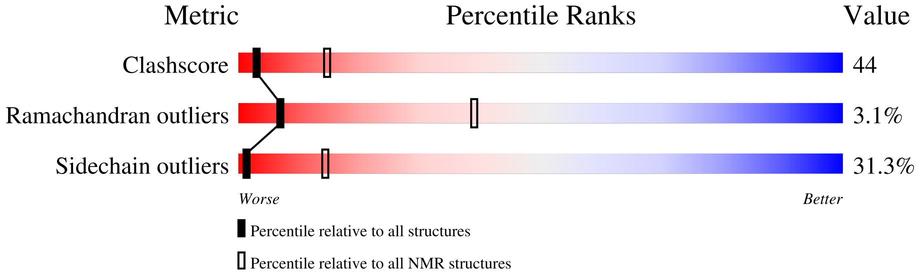

Metric	Whole archive (#Entries)	NMR archive (#Entries)
Clashscore	158937	12864
Ramachandran outliers	154571	11451
Sidechain outliers	154315	11428

The table below summarises the geometric issues observed across the polymeric chains and their fit to the experimental data. The red, orange, yellow and green segments indicate the fraction of residues that contain outliers for >=3, 2, 1 and 0 types of geometric quality criteria. A cyan segment indicates the fraction of residues that are not part of the well-defined cores, and a grey segment represents the fraction of residues that are not modelled. The numeric value for each fraction is indicated below the corresponding segment, with a dot representing fractions <=5%

Mol	Chain	Length	Quality of chain
1	A	104	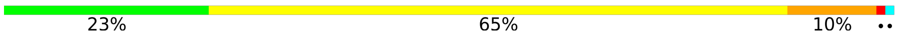

2 Ensemble composition and analysis (i)

This entry contains 10 models. Model 6 is the overall representative, medoid model (most similar to other models). The authors have identified model 1 as representative, based on the following criterion: *lowest energy*.

The following residues are included in the computation of the global validation metrics.

Well-defined (core) protein residues			
**Well-defined core**	**Residue range (total)**	**Backbone RMSD (Å)**	**Medoid model**
1	A:2-A:104 (103)	0.34	6

Ill-defined regions of proteins are excluded from the global statistics.

Ligands and non-protein polymers are included in the analysis.

The models can be grouped into 2 clusters. No single-model clusters were found.

Cluster number	Models
1	2, 4, 5, 6, 7, 8, 10
2	1, 3, 9

3 Entry composition (i)

There is only 1 type of molecule in this entry. The entry contains 1629 atoms, of which 811 are hydrogens and 0 are deuteriums.

- Molecule 1 is a protein called Thioredoxin.

Mol	Chain	Residues	Atoms						Trace
			Total	C	H	N	O	S	
1	A	104	1629	524	811	125	164	5	0

There are 2 discrepancies between the modelled and reference sequences:

Chain	Residue	Modelled	Actual	Comment	Reference
A	1	SER	-	expression tag	UNP Q7KQL8
A	9	SER	ALA	conflict	UNP Q7KQL8

4 Residue-property plots ⓘ

4.1 Average score per residue in the NMR ensemble

These plots are provided for all protein, RNA, DNA and oligosaccharide chains in the entry. The first graphic is the same as shown in the summary in section 1 of this report. The second graphic shows the sequence where residues are colour-coded according to the number of geometric quality criteria for which they contain at least one outlier: green = 0, yellow = 1, orange = 2 and red = 3 or more. Stretches of 2 or more consecutive residues without any outliers are shown as green connectors. Residues which are classified as ill-defined in the NMR ensemble, are shown in cyan with an underline colour-coded according to the previous scheme. Residues which were present in the experimental sample, but not modelled in the final structure are shown in grey.

- Molecule 1: Thioredoxin

Chain A:

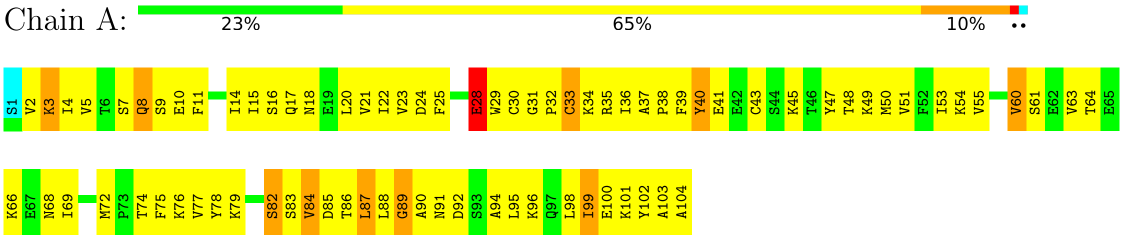

4.2 Scores per residue for each member of the ensemble

Colouring as in section 4.1 above.

4.2.1 Score per residue for model 1

- Molecule 1: Thioredoxin

Chain A:

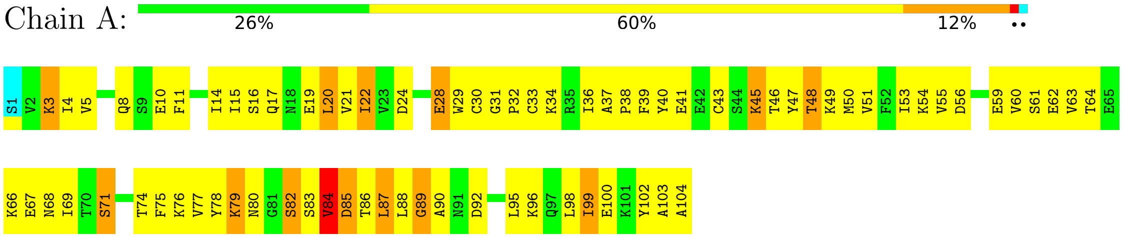

4.2.2 Score per residue for model 2

- Molecule 1: Thioredoxin

Chain A:

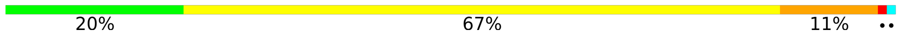

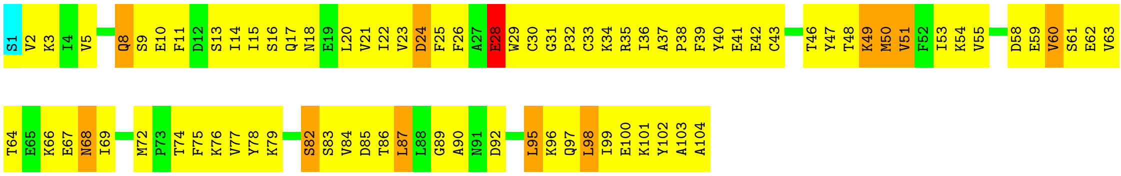

4.2.3 Score per residue for model 3

- Molecule 1: Thioredoxin

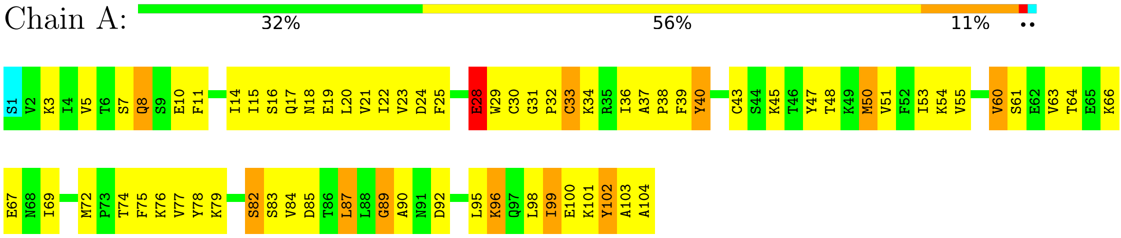

4.2.4 Score per residue for model 4

- Molecule 1: Thioredoxin

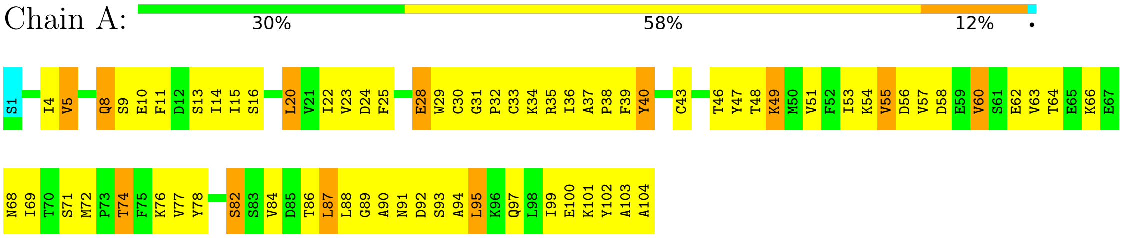

4.2.5 Score per residue for model 5

- Molecule 1: Thioredoxin

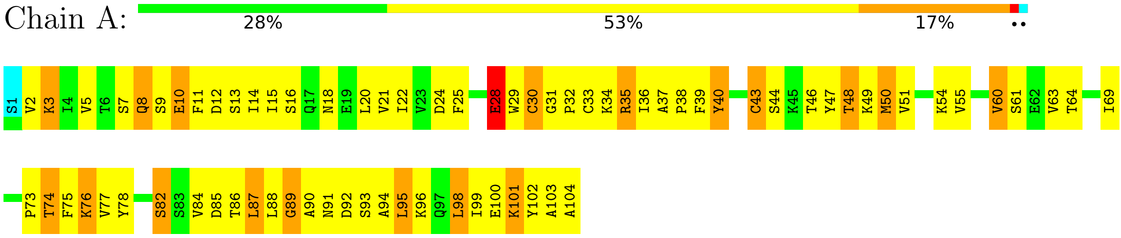

4.2.6 Score per residue for model 6 (medoid)

- Molecule 1: Thioredoxin

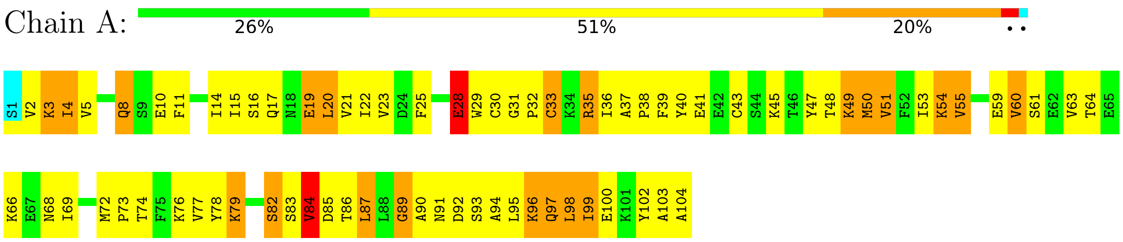

4.2.7 Score per residue for model 7

- Molecule 1: Thioredoxin

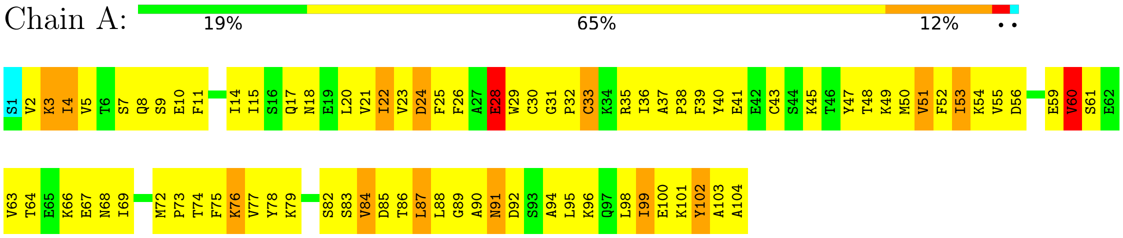

4.2.8 Score per residue for model 8

- Molecule 1: Thioredoxin

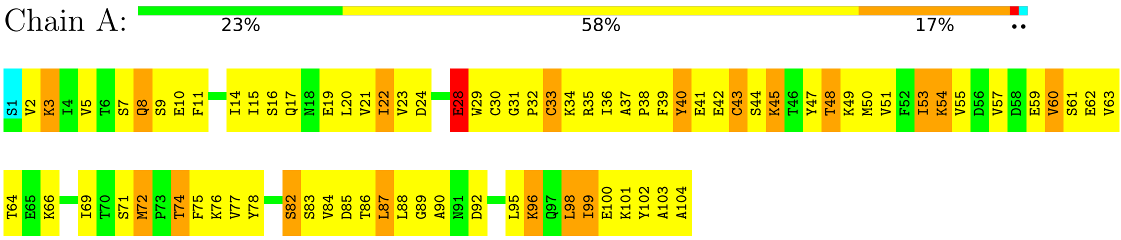

4.2.9 Score per residue for model 9

- Molecule 1: Thioredoxin

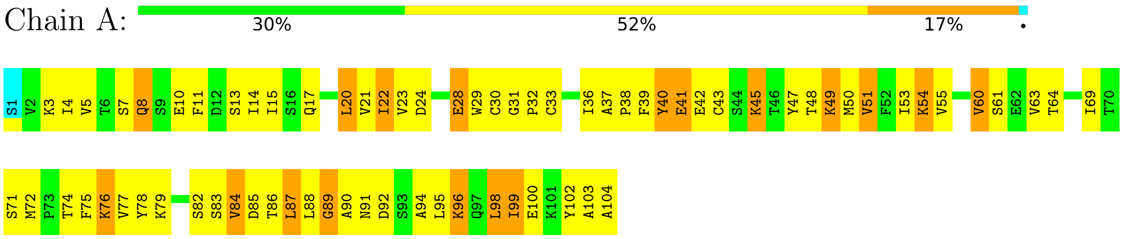

4.2.10 Score per residue for model 10

- Molecule 1: Thioredoxin

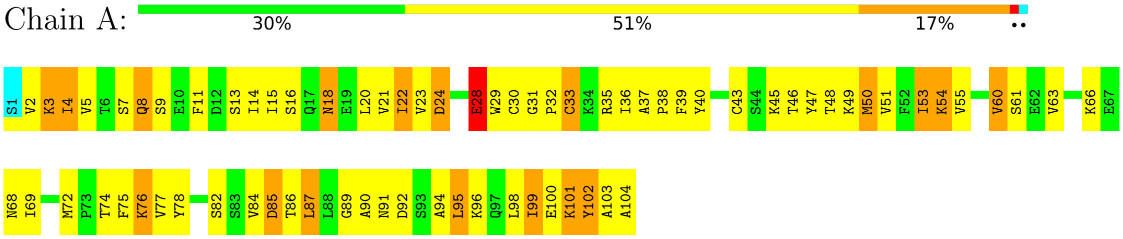

5 Refinement protocol and experimental data overview (i)

The models were refined using the following method: *simulated annealing*.

Of the 1000 calculated structures, 10 were deposited, based on the following criterion: *structures with the lowest energy*.

The following table shows the software used for structure solution, optimisation and refinement.

Software name	Classification	Version
CNS	structure solution	
CNS	refinement	

The following table shows chemical shift validation statistics as aggregates over all chemical shift files. Detailed validation can be found in section 7 of this report.

Chemical shift file(s)	working_cs.cif
Number of chemical shift lists	1
Total number of shifts	1327
Number of shifts mapped to atoms	1327
Number of unparsed shifts	0
Number of shifts with mapping errors	0
Number of shifts with mapping warnings	0
Assignment completeness (well-defined parts)	95%

6 Model quality (i)

6.1 Standard geometry (i)

The Z score for a bond length (or angle) is the number of standard deviations the observed value is removed from the expected value. A bond length (or angle) with $|Z| > 5$ is considered an outlier worth inspection. RMSZ is the (average) root-mean-square of all Z scores of the bond lengths (or angles).

Mol	Chain	Bond lengths		Bond angles	
		RMSZ	#Z>5	RMSZ	#Z>5
1	A	0.69±0.00	4±0/826 (0.5± 0.0%)	0.67±0.01	1±0/1117 (0.1± 0.0%)
All	All	0.69	40/8260 (0.5%)	0.67	13/11170 (0.1%)

All unique bond outliers are listed below. They are sorted according to the Z-score of the worst occurrence in the ensemble.

Mol	Chain	Res	Type	Atoms	Z	Observed(Å)	Ideal(Å)	Models	
								Worst	Total
1	A	29	TRP	CG-CD1	9.70	1.50	1.36	1	10
1	A	29	TRP	CB-CG	6.33	1.61	1.50	1	10
1	A	28	GLU	CA-C	5.73	1.67	1.52	2	10
1	A	29	TRP	CA-CB	5.73	1.66	1.53	3	10

All unique angle outliers are listed below. They are sorted according to the Z-score of the worst occurrence in the ensemble.

Mol	Chain	Res	Type	Atoms	Z	Observed(°)	Ideal(°)	Models	
								Worst	Total
1	A	29	TRP	CB-CG-CD1	5.69	134.40	127.00	4	10
1	A	28	GLU	CA-C-N	5.18	128.60	117.20	4	3

There are no chirality outliers.

There are no planarity outliers.

6.2 Too-close contacts (i)

In the following table, the Non-H and H(model) columns list the number of non-hydrogen atoms and hydrogen atoms in each chain respectively. The H(added) column lists the number of hydrogen atoms added and optimized by MolProbity. The Clashes column lists the number of clashes averaged over the ensemble.

Mol	Chain	Non-H	H(model)	H(added)	Clashes
1	A	812	807	807	71±8
All	All	8120	8070	8070	709

The all-atom clashscore is defined as the number of clashes found per 1000 atoms (including hydrogen atoms). The all-atom clashscore for this structure is 44.

All unique clashes are listed below, sorted by their clash magnitude.

Atom-1	Atom-2	Clash(Å)	Distance(Å)	Models	
				Worst	Total
1:A:11:PHE:CE2	1:A:63:VAL:HG13	0.92	2.00	7	10
1:A:21:VAL:HG13	1:A:51:VAL:HG12	0.90	1.44	2	4
1:A:76:LYS:HG3	1:A:86:THR:HG22	0.83	1.50	7	2
1:A:20:LEU:HD21	1:A:102:TYR:HB3	0.81	1.50	6	7
1:A:47:TYR:CD2	1:A:99:ILE:HG12	0.81	2.11	4	3
1:A:5:VAL:HG13	1:A:55:VAL:HG22	0.81	1.53	7	5
1:A:20:LEU:HD23	1:A:50:MET:CE	0.80	2.06	2	4
1:A:76:LYS:HD2	1:A:86:THR:HG22	0.80	1.51	9	2
1:A:5:VAL:CG1	1:A:55:VAL:HG22	0.80	2.07	8	5
1:A:11:PHE:CD1	1:A:55:VAL:HG21	0.79	2.11	8	5
1:A:36:ILE:HG12	1:A:90:ALA:HB1	0.79	1.54	2	9
1:A:77:VAL:O	1:A:84:VAL:HG12	0.78	1.77	2	10
1:A:64:THR:HG23	1:A:69:ILE:HB	0.78	1.56	1	9
1:A:18:ASN:OD1	1:A:21:VAL:HG22	0.78	1.79	10	1
1:A:21:VAL:HG12	1:A:53:ILE:HD11	0.77	1.55	10	2
1:A:55:VAL:HG11	1:A:63:VAL:HG11	0.76	1.56	6	10
1:A:76:LYS:CD	1:A:86:THR:HG22	0.76	2.11	5	7
1:A:14:ILE:HG22	1:A:21:VAL:HG11	0.75	1.58	3	4
1:A:21:VAL:HG13	1:A:51:VAL:CG1	0.75	2.11	2	4
1:A:99:ILE:O	1:A:103:ALA:HB3	0.74	1.82	7	10
1:A:2:VAL:HG12	1:A:54:LYS:HD2	0.73	1.59	8	3
1:A:76:LYS:CG	1:A:86:THR:HG22	0.73	2.14	4	8
1:A:15:ILE:HD12	1:A:78:TYR:CD2	0.73	2.18	3	3
1:A:22:ILE:HD12	1:A:77:VAL:HG22	0.72	1.61	8	2
1:A:71:SER:HB3	1:A:88:LEU:HD21	0.71	1.62	8	2
1:A:3:LYS:O	1:A:54:LYS:N	0.71	2.22	8	8
1:A:99:ILE:HD13	1:A:103:ALA:HB3	0.71	1.60	7	6
1:A:97:GLN:OE1	1:A:98:LEU:N	0.70	2.23	6	1
1:A:5:VAL:HG13	1:A:55:VAL:HG23	0.70	1.61	6	3
1:A:50:MET:SD	1:A:103:ALA:HB2	0.69	2.26	5	7
1:A:20:LEU:HD23	1:A:50:MET:HE1	0.69	1.63	5	3
1:A:15:ILE:HD12	1:A:78:TYR:HD2	0.68	1.49	1	2
1:A:23:VAL:HG22	1:A:53:ILE:HG13	0.68	1.64	8	1
1:A:99:ILE:HD13	1:A:103:ALA:CB	0.68	2.18	7	6

Continued on next page...

Continued from previous page...

Atom-1	Atom-2	Clash(Å)	Distance(Å)	Models	
				Worst	Total
1:A:20:LEU:HD23	1:A:50:MET:SD	0.67	2.30	3	2
1:A:47:TYR:CE2	1:A:99:ILE:CG2	0.67	2.78	9	10
1:A:23:VAL:HG13	1:A:25:PHE:CE1	0.67	2.25	7	3
1:A:23:VAL:HG22	1:A:53:ILE:CG1	0.67	2.20	8	1
1:A:76:LYS:HG2	1:A:86:THR:HG22	0.66	1.68	6	5
1:A:43:CYS:O	1:A:47:TYR:CD2	0.65	2.49	1	7
1:A:47:TYR:CE2	1:A:99:ILE:HG23	0.65	2.26	9	7
1:A:85:ASP:OD2	1:A:98:LEU:HD12	0.65	1.92	5	2
1:A:87:LEU:HB3	1:A:98:LEU:HD13	0.65	1.68	9	2
1:A:43:CYS:HB3	1:A:47:TYR:CE2	0.64	2.27	8	3
1:A:60:VAL:HB	1:A:63:VAL:HG23	0.64	1.70	8	8
1:A:85:ASP:OD2	1:A:98:LEU:HD13	0.64	1.93	1	1
1:A:8:GLN:NE2	1:A:63:VAL:HG22	0.64	2.08	10	8
1:A:98:LEU:HD12	1:A:98:LEU:O	0.64	1.92	1	1
1:A:10:GLU:O	1:A:14:ILE:HD12	0.64	1.93	5	7
1:A:53:ILE:N	1:A:53:ILE:HD13	0.64	2.08	7	2
1:A:76:LYS:HG2	1:A:78:TYR:CE1	0.63	2.28	3	1
1:A:11:PHE:O	1:A:15:ILE:HG12	0.63	1.94	7	10
1:A:50:MET:HE3	1:A:103:ALA:CA	0.62	2.24	8	2
1:A:47:TYR:CD2	1:A:99:ILE:HD12	0.62	2.28	8	5
1:A:20:LEU:HD12	1:A:78:TYR:O	0.61	1.95	3	4
1:A:36:ILE:CG1	1:A:90:ALA:HB1	0.61	2.26	5	5
1:A:17:GLN:N	1:A:17:GLN:OE1	0.61	2.34	9	1
1:A:5:VAL:HG13	1:A:55:VAL:CG2	0.60	2.25	1	2
1:A:20:LEU:HB3	1:A:50:MET:SD	0.60	2.36	10	2
1:A:100:GLU:CA	1:A:104:ALA:HB3	0.60	2.26	4	8
1:A:100:GLU:HA	1:A:104:ALA:HB3	0.60	1.73	7	8
1:A:21:VAL:CG1	1:A:53:ILE:HD11	0.60	2.26	7	2
1:A:22:ILE:HG22	1:A:50:MET:HG2	0.60	1.73	9	4
1:A:39:PHE:CE2	1:A:92:ASP:O	0.60	2.54	4	10
1:A:20:LEU:HD21	1:A:102:TYR:CB	0.60	2.26	5	3
1:A:37:ALA:N	1:A:38:PRO:HD2	0.60	2.12	8	2
1:A:95:LEU:O	1:A:99:ILE:HG22	0.60	1.96	5	3
1:A:43:CYS:HB3	1:A:47:TYR:CD2	0.59	2.32	8	8
1:A:78:TYR:HA	1:A:82:SER:O	0.59	1.98	6	10
1:A:36:ILE:HB	1:A:90:ALA:HB1	0.59	1.74	6	2
1:A:39:PHE:CE2	1:A:95:LEU:HD23	0.59	2.32	10	2
1:A:44:SER:O	1:A:48:THR:HG22	0.59	1.97	8	2
1:A:22:ILE:HD12	1:A:77:VAL:CG2	0.59	2.27	9	2
1:A:31:GLY:N	1:A:32:PRO:HD2	0.58	2.13	9	10
1:A:13:SER:O	1:A:17:GLN:OE1	0.58	2.20	9	1

Continued on next page...

Continued from previous page...

Atom-1	Atom-2	Clash(Å)	Distance(Å)	Models	
				Worst	Total
1:A:3:LYS:HB3	1:A:53:ILE:HG22	0.58	1.76	7	3
1:A:40:TYR:HA	1:A:43:CYS:SG	0.57	2.38	5	2
1:A:87:LEU:HD11	1:A:89:GLY:O	0.57	1.99	10	7
1:A:30:CYS:SG	1:A:32:PRO:HG2	0.57	2.39	5	1
1:A:47:TYR:CD2	1:A:99:ILE:HG23	0.57	2.34	8	1
1:A:102:TYR:N	1:A:102:TYR:CD1	0.57	2.73	10	3
1:A:87:LEU:CB	1:A:98:LEU:HD13	0.56	2.29	9	3
1:A:23:VAL:HG23	1:A:53:ILE:O	0.56	2.01	3	3
1:A:11:PHE:HB2	1:A:55:VAL:HG21	0.56	1.78	5	3
1:A:39:PHE:CE2	1:A:43:CYS:SG	0.56	2.99	4	8
1:A:2:VAL:HG11	1:A:54:LYS:CD	0.55	2.31	2	3
1:A:30:CYS:O	1:A:33:CYS:SG	0.55	2.64	10	5
1:A:28:GLU:HG2	1:A:28:GLU:O	0.55	2.02	6	7
1:A:96:LYS:O	1:A:100:GLU:HB2	0.55	2.01	9	7
1:A:91:ASN:OD1	1:A:94:ALA:HB3	0.54	2.02	7	1
1:A:33:CYS:O	1:A:36:ILE:HG22	0.54	2.02	10	9
1:A:43:CYS:CB	1:A:47:TYR:CD2	0.54	2.90	8	2
1:A:15:ILE:HG23	1:A:78:TYR:CD2	0.54	2.37	1	2
1:A:36:ILE:O	1:A:40:TYR:HB2	0.54	2.02	5	2
1:A:4:ILE:HG23	1:A:4:ILE:O	0.54	2.02	10	4
1:A:24:ASP:HB2	1:A:75:PHE:CE1	0.54	2.38	10	6
1:A:14:ILE:HG21	1:A:53:ILE:HG21	0.53	1.80	4	2
1:A:24:ASP:HB3	1:A:75:PHE:CE1	0.53	2.38	3	2
1:A:71:SER:CB	1:A:88:LEU:HD21	0.53	2.33	8	2
1:A:18:ASN:OD1	1:A:21:VAL:CG2	0.53	2.55	10	1
1:A:100:GLU:HA	1:A:104:ALA:CB	0.53	2.34	10	8
1:A:5:VAL:HG23	1:A:10:GLU:HG2	0.53	1.80	7	2
1:A:22:ILE:HD13	1:A:77:VAL:HG22	0.53	1.80	3	1
1:A:100:GLU:O	1:A:104:ALA:HA	0.52	2.04	1	1
1:A:23:VAL:HA	1:A:53:ILE:O	0.52	2.04	10	4
1:A:20:LEU:HA	1:A:78:TYR:O	0.51	2.05	3	1
1:A:36:ILE:CB	1:A:90:ALA:HB1	0.51	2.35	6	2
1:A:47:TYR:CD2	1:A:99:ILE:CG2	0.51	2.93	8	1
1:A:39:PHE:CD2	1:A:95:LEU:HD23	0.51	2.41	5	2
1:A:99:ILE:CD1	1:A:103:ALA:CB	0.51	2.87	7	6
1:A:57:VAL:HG11	1:A:72:MET:SD	0.51	2.45	8	1
1:A:22:ILE:HD12	1:A:23:VAL:N	0.51	2.20	10	1
1:A:47:TYR:CE2	1:A:99:ILE:HG21	0.51	2.41	5	6
1:A:91:ASN:HB2	1:A:94:ALA:HB3	0.51	1.84	5	5
1:A:4:ILE:O	1:A:4:ILE:CG2	0.50	2.59	6	1
1:A:15:ILE:HD12	1:A:78:TYR:CE2	0.50	2.41	3	1

Continued on next page...

Continued from previous page...

Atom-1	Atom-2	Clash(Å)	Distance(Å)	Models	
				Worst	Total
1:A:24:ASP:OD2	1:A:40:TYR:CE2	0.50	2.65	9	2
1:A:22:ILE:C	1:A:22:ILE:HD13	0.50	2.26	7	1
1:A:73:PRO:HG2	1:A:90:ALA:N	0.49	2.22	7	3
1:A:5:VAL:CG1	1:A:55:VAL:HG23	0.49	2.34	6	1
1:A:26:PHE:CZ	1:A:56:ASP:OD2	0.49	2.66	7	1
1:A:24:ASP:OD1	1:A:26:PHE:HB3	0.49	2.07	7	2
1:A:100:GLU:O	1:A:104:ALA:CA	0.49	2.61	1	2
1:A:15:ILE:HG23	1:A:78:TYR:HD2	0.48	1.67	1	2
1:A:37:ALA:HB3	1:A:38:PRO:HD3	0.48	1.84	4	7
1:A:99:ILE:CD1	1:A:103:ALA:HB3	0.48	2.38	10	4
1:A:85:ASP:HB2	1:A:102:TYR:CZ	0.48	2.44	6	2
1:A:85:ASP:HB2	1:A:102:TYR:OH	0.48	2.08	5	3
1:A:98:LEU:HD12	1:A:98:LEU:C	0.48	2.29	1	1
1:A:24:ASP:OD2	1:A:75:PHE:CZ	0.48	2.67	3	2
1:A:20:LEU:HD11	1:A:77:VAL:CG1	0.48	2.39	6	4
1:A:2:VAL:HG11	1:A:54:LYS:HD2	0.48	1.85	2	2
1:A:24:ASP:OD1	1:A:24:ASP:N	0.48	2.47	5	1
1:A:47:TYR:CD2	1:A:99:ILE:CG1	0.48	2.92	4	3
1:A:87:LEU:HD12	1:A:88:LEU:N	0.47	2.24	7	4
1:A:59:GLU:O	1:A:60:VAL:HG13	0.47	2.09	7	1
1:A:20:LEU:CD2	1:A:50:MET:HE1	0.47	2.39	8	1
1:A:87:LEU:HD12	1:A:87:LEU:C	0.47	2.30	4	8
1:A:5:VAL:CG1	1:A:55:VAL:CG2	0.47	2.92	7	4
1:A:74:THR:HB	1:A:76:LYS:HZ1	0.47	1.70	5	1
1:A:11:PHE:CZ	1:A:63:VAL:HG13	0.47	2.43	7	1
1:A:24:ASP:CG	1:A:75:PHE:CD1	0.47	2.88	3	2
1:A:50:MET:HE3	1:A:103:ALA:HA	0.47	1.85	8	1
1:A:43:CYS:HA	1:A:47:TYR:CE2	0.47	2.45	10	2
1:A:26:PHE:CE1	1:A:56:ASP:OD1	0.47	2.67	7	1
1:A:49:LYS:NZ	1:A:103:ALA:O	0.47	2.47	6	1
1:A:56:ASP:OD1	1:A:59:GLU:HB2	0.47	2.10	1	1
1:A:4:ILE:HB	1:A:54:LYS:HD3	0.47	1.86	10	1
1:A:20:LEU:HD11	1:A:77:VAL:HG12	0.46	1.87	10	4
1:A:25:PHE:CZ	1:A:78:TYR:OH	0.46	2.69	3	1
1:A:100:GLU:O	1:A:104:ALA:C	0.46	2.53	4	2
1:A:20:LEU:HG	1:A:50:MET:CE	0.46	2.40	8	3
1:A:45:LYS:O	1:A:48:THR:HG23	0.46	2.10	1	1
1:A:100:GLU:O	1:A:104:ALA:N	0.46	2.49	1	3
1:A:11:PHE:CZ	1:A:25:PHE:CE2	0.46	3.04	4	3
1:A:55:VAL:CG1	1:A:63:VAL:HG11	0.46	2.38	4	1
1:A:9:SER:O	1:A:13:SER:N	0.46	2.49	5	4

Continued on next page...

Continued from previous page...

Atom-1	Atom-2	Clash(Å)	Distance(Å)	Models	
				Worst	Total
1:A:20:LEU:HD13	1:A:79:LYS:HG2	0.45	1.88	6	1
1:A:20:LEU:CD2	1:A:50:MET:CE	0.45	2.95	7	2
1:A:11:PHE:CD2	1:A:63:VAL:HG13	0.45	2.46	9	3
1:A:37:ALA:N	1:A:38:PRO:CD	0.45	2.79	7	10
1:A:68:ASN:OD1	1:A:68:ASN:O	0.45	2.35	1	2
1:A:99:ILE:O	1:A:103:ALA:CB	0.45	2.60	5	5
1:A:24:ASP:OD1	1:A:54:LYS:HG3	0.45	2.11	5	3
1:A:87:LEU:HB2	1:A:98:LEU:HD13	0.45	1.87	2	1
1:A:87:LEU:CB	1:A:98:LEU:HD22	0.45	2.42	3	1
1:A:43:CYS:SG	1:A:95:LEU:HD21	0.45	2.51	5	1
1:A:62:GLU:O	1:A:66:LYS:CG	0.45	2.65	2	2
1:A:67:GLU:O	1:A:68:ASN:ND2	0.45	2.50	7	3
1:A:47:TYR:O	1:A:49:LYS:N	0.45	2.50	2	3
1:A:87:LEU:HB3	1:A:98:LEU:HD22	0.45	1.89	3	1
1:A:94:ALA:O	1:A:97:GLN:OE1	0.45	2.35	6	1
1:A:50:MET:HE3	1:A:103:ALA:HB2	0.45	1.89	8	1
1:A:22:ILE:CD1	1:A:40:TYR:OH	0.44	2.65	7	1
1:A:52:PHE:CZ	1:A:99:ILE:HD11	0.44	2.47	7	1
1:A:91:ASN:OD1	1:A:91:ASN:N	0.44	2.50	7	1
1:A:2:VAL:CG1	1:A:54:LYS:HD2	0.44	2.39	8	1
1:A:47:TYR:CG	1:A:99:ILE:CG1	0.44	3.00	2	2
1:A:24:ASP:CB	1:A:75:PHE:CE1	0.44	3.00	5	2
1:A:71:SER:O	1:A:74:THR:OG1	0.44	2.27	8	2
1:A:22:ILE:HD13	1:A:77:VAL:CG2	0.44	2.41	3	1
1:A:46:THR:HG22	1:A:47:TYR:CD1	0.44	2.47	10	1
1:A:23:VAL:HB	1:A:53:ILE:HG13	0.44	1.90	4	1
1:A:76:LYS:HB3	1:A:78:TYR:CE1	0.44	2.48	6	1
1:A:50:MET:HE3	1:A:103:ALA:CB	0.44	2.42	8	1
1:A:77:VAL:C	1:A:84:VAL:HG12	0.44	2.32	2	1
1:A:76:LYS:HD3	1:A:86:THR:HG22	0.44	1.90	1	2
1:A:23:VAL:HB	1:A:53:ILE:HG12	0.44	1.90	7	1
1:A:22:ILE:HD13	1:A:99:ILE:HG12	0.44	1.90	1	1
1:A:22:ILE:HG12	1:A:40:TYR:OH	0.44	2.13	9	1
1:A:68:ASN:O	1:A:68:ASN:CG	0.43	2.55	2	1
1:A:60:VAL:HB	1:A:63:VAL:CG2	0.43	2.42	8	1
1:A:98:LEU:HD23	1:A:99:ILE:N	0.43	2.28	9	2
1:A:24:ASP:CG	1:A:75:PHE:CE1	0.43	2.91	3	1
1:A:22:ILE:HD12	1:A:76:LYS:O	0.43	2.14	3	1
1:A:47:TYR:CG	1:A:99:ILE:HG12	0.43	2.48	2	2
1:A:11:PHE:HD1	1:A:55:VAL:HG21	0.43	1.65	8	2
1:A:24:ASP:OD2	1:A:40:TYR:CZ	0.43	2.71	3	1

Continued on next page...

Continued from previous page...

Atom-1	Atom-2	Clash(Å)	Distance(Å)	Models	
				Worst	Total
1:A:101:LYS:HE2	1:A:102:TYR:CE1	0.43	2.49	5	1
1:A:85:ASP:CB	1:A:102:TYR:OH	0.43	2.67	2	1
1:A:18:ASN:HB2	1:A:21:VAL:HG22	0.43	1.90	3	2
1:A:42:GLU:HG3	1:A:43:CYS:N	0.43	2.29	2	1
1:A:19:GLU:O	1:A:79:LYS:HA	0.43	2.14	6	2
1:A:8:GLN:HE22	1:A:63:VAL:HG22	0.43	1.72	3	1
1:A:47:TYR:HA	1:A:49:LYS:HZ3	0.43	1.74	6	1
1:A:101:LYS:HB3	1:A:102:TYR:CD1	0.43	2.48	7	2
1:A:42:GLU:O	1:A:45:LYS:HG3	0.42	2.14	9	2
1:A:11:PHE:CE1	1:A:25:PHE:CE1	0.42	3.06	5	1
1:A:22:ILE:CD1	1:A:77:VAL:HG22	0.42	2.44	9	1
1:A:41:GLU:HG3	1:A:42:GLU:N	0.42	2.28	9	1
1:A:62:GLU:O	1:A:66:LYS:HG2	0.42	2.13	1	1
1:A:23:VAL:CG2	1:A:53:ILE:CD1	0.42	2.98	9	2
1:A:39:PHE:CG	1:A:92:ASP:HB3	0.42	2.50	3	1
1:A:24:ASP:HB3	1:A:40:TYR:CZ	0.42	2.50	4	1
1:A:22:ILE:HD13	1:A:23:VAL:N	0.42	2.30	7	1
1:A:99:ILE:O	1:A:99:ILE:HD13	0.42	2.15	7	2
1:A:43:CYS:O	1:A:47:TYR:HB2	0.42	2.14	9	2
1:A:78:TYR:CA	1:A:82:SER:O	0.42	2.68	9	1
1:A:30:CYS:SG	1:A:33:CYS:N	0.42	2.93	5	1
1:A:31:GLY:N	1:A:32:PRO:CD	0.41	2.84	4	6
1:A:24:ASP:OD1	1:A:54:LYS:CG	0.41	2.68	5	2
1:A:20:LEU:HD13	1:A:79:LYS:HD2	0.41	1.92	7	1
1:A:96:LYS:O	1:A:100:GLU:CB	0.41	2.68	8	2
1:A:35:ARG:O	1:A:38:PRO:HD2	0.41	2.15	5	2
1:A:43:CYS:O	1:A:47:TYR:CG	0.41	2.74	3	1
1:A:67:GLU:OE2	1:A:78:TYR:CE1	0.41	2.74	3	1
1:A:100:GLU:O	1:A:104:ALA:HB3	0.41	2.16	6	1
1:A:77:VAL:HG11	1:A:102:TYR:CD2	0.41	2.51	7	1
1:A:85:ASP:CB	1:A:102:TYR:CZ	0.41	3.03	10	1
1:A:87:LEU:HB3	1:A:98:LEU:CD2	0.41	2.45	1	1
1:A:35:ARG:O	1:A:38:PRO:HG2	0.41	2.16	6	1
1:A:18:ASN:HB2	1:A:21:VAL:CG2	0.41	2.46	7	2
1:A:11:PHE:CZ	1:A:25:PHE:HE2	0.41	2.34	4	1
1:A:76:LYS:HD2	1:A:86:THR:CG2	0.41	2.39	5	1
1:A:14:ILE:O	1:A:18:ASN:ND2	0.41	2.54	10	1
1:A:20:LEU:HD23	1:A:50:MET:CG	0.41	2.46	10	1
1:A:11:PHE:CD2	1:A:12:ASP:OD1	0.41	2.74	5	1
1:A:11:PHE:CD2	1:A:63:VAL:HG22	0.41	2.51	7	1
1:A:84:VAL:CG1	1:A:85:ASP:N	0.41	2.83	10	1

Continued on next page...

Continued from previous page...

Atom-1	Atom-2	Clash(Å)	Distance(Å)	Models	
				Worst	Total
1:A:43:CYS:CB	1:A:47:TYR:CE2	0.40	3.04	3	1
1:A:47:TYR:HB3	1:A:103:ALA:HB1	0.40	1.94	3	1
1:A:56:ASP:OD1	1:A:57:VAL:N	0.40	2.55	4	1
1:A:60:VAL:CG2	1:A:63:VAL:CG2	0.40	2.99	7	1
1:A:39:PHE:O	1:A:43:CYS:SG	0.40	2.79	8	1
1:A:99:ILE:HG13	1:A:103:ALA:HB3	0.40	1.92	4	1
1:A:3:LYS:CB	1:A:53:ILE:HG22	0.40	2.47	6	1
1:A:11:PHE:CE2	1:A:63:VAL:CG1	0.40	2.94	9	1

6.3 Torsion angles (i)

6.3.1 Protein backbone (i)

In the following table, the Percentiles column shows the percent Ramachandran outliers of the chain as a percentile score with respect to all PDB entries followed by that with respect to all NMR entries. The Analysed column shows the number of residues for which the backbone conformation was analysed and the total number of residues.

Mol	Chain	Analysed	Favoured	Allowed	Outliers	Percentiles	
1	A	102/104 (98%)	96±1 (94±1%)	3±1 (3±1%)	3±1 (3±1%)	7	39
All	All	1020/1040 (98%)	961 (94%)	27 (3%)	32 (3%)	7	39

All 5 unique Ramachandran outliers are listed below. They are sorted by the frequency of occurrence in the ensemble.

Mol	Chain	Res	Type	Models (Total)
1	A	28	GLU	10
1	A	48	THR	10
1	A	89	GLY	8
1	A	84	VAL	2
1	A	60	VAL	2

6.3.2 Protein sidechains (i)

In the following table, the Percentiles column shows the percent sidechain outliers of the chain as a percentile score with respect to all PDB entries followed by that with respect to all NMR entries. The Analysed column shows the number of residues for which the sidechain conformation was analysed and the total number of residues.

Mol	Chain	Analysed	Rotameric	Outliers	Percentiles	
1	A	94/95 (99%)	65±4 (69±4%)	29±4 (31±4%)	1	14
All	All	940/950 (99%)	646 (69%)	294 (31%)	1	14

All 58 unique residues with a non-rotameric sidechain are listed below. They are sorted by the frequency of occurrence in the ensemble.

Mol	Chain	Res	Type	Models (Total)
1	A	8	GLN	10
1	A	51	VAL	10
1	A	74	THR	10
1	A	87	LEU	10
1	A	95	LEU	10
1	A	22	ILE	9
1	A	40	TYR	9
1	A	49	LYS	9
1	A	61	SER	9
1	A	60	VAL	9
1	A	16	SER	8
1	A	72	MET	8
1	A	45	LYS	7
1	A	82	SER	7
1	A	83	SER	7
1	A	99	ILE	7
1	A	98	LEU	7
1	A	3	LYS	6
1	A	17	GLN	6
1	A	34	LYS	6
1	A	41	GLU	6
1	A	35	ARG	6
1	A	96	LYS	6
1	A	101	LYS	6
1	A	7	SER	6
1	A	30	CYS	5
1	A	79	LYS	5
1	A	50	MET	5
1	A	33	CYS	5
1	A	66	LYS	5
1	A	54	LYS	5
1	A	20	LEU	4
1	A	46	THR	4
1	A	84	VAL	4
1	A	85	ASP	4
1	A	68	ASN	4

Continued on next page...

Continued from previous page...

Mol	Chain	Res	Type	Models (Total)
1	A	76	LYS	4
1	A	4	ILE	4
1	A	24	ASP	3
1	A	59	GLU	3
1	A	97	GLN	3
1	A	19	GLU	3
1	A	102	TYR	3
1	A	5	VAL	3
1	A	93	SER	3
1	A	53	ILE	3
1	A	71	SER	2
1	A	58	ASP	2
1	A	55	VAL	2
1	A	10	GLU	2
1	A	43	CYS	2
1	A	9	SER	2
1	A	80	ASN	1
1	A	88	LEU	1
1	A	91	ASN	1
1	A	62	GLU	1
1	A	18	ASN	1
1	A	69	ILE	1

6.3.3 RNA (i)

There are no RNA molecules in this entry.

6.4 Non-standard residues in protein, DNA, RNA chains (i)

There are no non-standard protein/DNA/RNA residues in this entry.

6.5 Carbohydrates (i)

There are no monosaccharides in this entry.

6.6 Ligand geometry (i)

There are no ligands in this entry.

6.7 Other polymers (i)

There are no such molecules in this entry.

6.8 Polymer linkage issues (i)

There are no chain breaks in this entry.

7 Chemical shift validation (i)

The completeness of assignment taking into account all chemical shift lists is 95% for the well-defined parts and 95% for the entire structure.

7.1 Chemical shift list 1

File name: working_cs.cif

Chemical shift list name: *assigned_chem_shift_list_1*

7.1.1 Bookkeeping (i)

The following table shows the results of parsing the chemical shift list and reports the number of nuclei with statistically unusual chemical shifts.

Total number of shifts	1327
Number of shifts mapped to atoms	1327
Number of unparsed shifts	0
Number of shifts with mapping errors	0
Number of shifts with mapping warnings	0
Number of shift outliers (ShiftChecker)	1

7.1.2 Chemical shift referencing (i)

The following table shows the suggested chemical shift referencing corrections.

Nucleus	# values	Correction ± precision, *ppm*	Suggested action
$^{13}C_{\alpha}$	103	-0.01 ± 0.16	None needed (< 0.5 ppm)
$^{13}C_{\beta}$	100	0.41 ± 0.15	None needed (< 0.5 ppm)
$^{13}C'$	100	0.03 ± 0.17	None needed (< 0.5 ppm)
^{15}N	101	0.13 ± 0.16	None needed (< 0.5 ppm)

7.1.3 Completeness of resonance assignments (i)

The following table shows the completeness of the chemical shift assignments for the well-defined regions of the structure. The overall completeness is 95%, i.e. 1314 atoms were assigned a chemical shift out of a possible 1384. 0 out of 16 assigned methyl groups (LEU and VAL) were assigned stereospecifically.

	Total	^{1}H	^{13}C	^{15}N
Backbone	506/512 (99%)	205/206 (100%)	201/206 (98%)	100/100 (100%)
Sidechain	711/764 (93%)	492/497 (99%)	219/247 (89%)	0/20 (0%)

Continued on next page...

Continued from previous page...

	Total	^{1}H	^{13}C	^{15}N
Aromatic	97/108 (90%)	51/52 (98%)	46/55 (84%)	0/1 (0%)
Overall	1314/1384 (95%)	748/755 (99%)	466/508 (92%)	100/121 (83%)

The following table shows the completeness of the chemical shift assignments for the full structure. The overall completeness is 95%, i.e. 1321 atoms were assigned a chemical shift out of a possible 1392. 0 out of 16 assigned methyl groups (LEU and VAL) were assigned stereospecifically.

	Total	^{1}H	^{13}C	^{15}N
Backbone	510/517 (99%)	206/208 (99%)	203/208 (98%)	101/101 (100%)
Sidechain	714/767 (93%)	494/499 (99%)	220/248 (89%)	0/20 (0%)
Aromatic	97/108 (90%)	51/52 (98%)	46/55 (84%)	0/1 (0%)
Overall	1321/1392 (95%)	751/759 (99%)	469/511 (92%)	101/122 (83%)

7.1.4 Statistically unusual chemical shifts ⓘ

The following table lists the statistically unusual chemical shifts. These are statistical measures, and large deviations from the mean do not necessarily imply incorrect assignments. Molecules containing paramagnetic centres or hemes are expected to give rise to anomalous chemical shifts.

List Id	Chain	Res	Type	Atom	Shift, *ppm*	Expected range, *ppm*	Z-score
1	A	74	THR	HG1	5.46	0.08 – 2.19	20.5

7.1.5 Random Coil Index (RCI) plots ⓘ

The image below reports *random coil index* values for the protein chains in the structure. The height of each bar gives a probability of a given residue to be disordered, as predicted from the available chemical shifts and the amino acid sequence. A value above 0.2 is an indication of significant predicted disorder. The colour of the bar shows whether the residue is in the well-defined core (black) or in the ill-defined residue ranges (cyan), as described in section 2 on ensemble composition.If well-defined core and ill-defined regions are not identified then it is shown as gray bars.

Random coil index (RCI) for chain A:

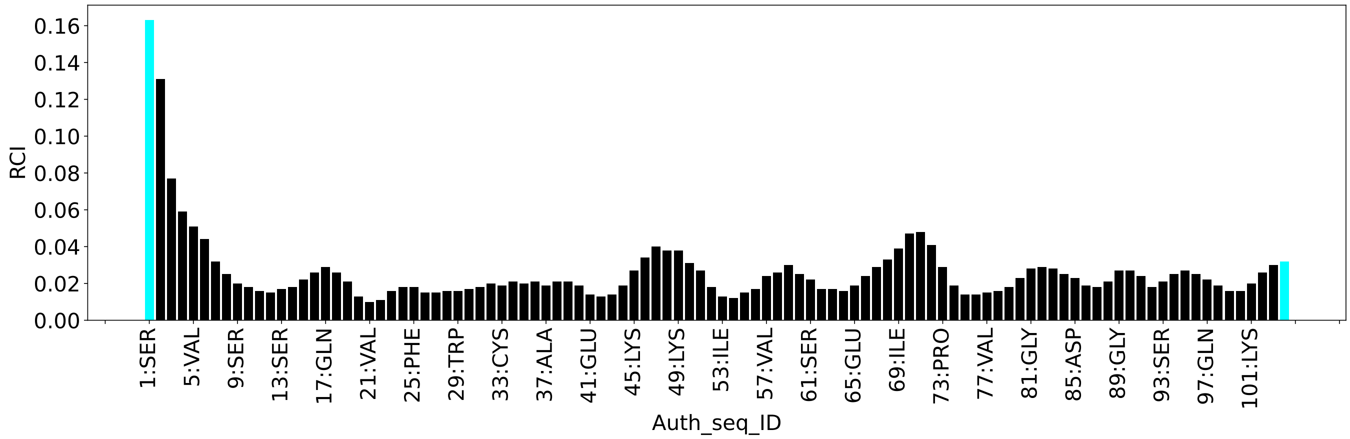
RCI
0.16
0.14
0.12
0.10
0.08
0.06
0.04
0.02
0.00
1:SER
5:VAL
9:SER
13:SER
17:GLN
21:VAL
25:PHE
29:TRP
33:CYS
37:ALA
41:GLU
45:LYS
49:LYS
53:ILE
57:VAL
61:SER
65:GLU
69:ILE
73:PRO
77:VAL
81:GLY
85:ASP
89:GLY
93:SER
97:GLN
101:LYS
Auth_seq_ID

8 NMR restraints analysis (i)

8.1 Conformationally restricting restraints (i)

The following table provides the summary of experimentally observed NMR restraints in different categories. Restraints are classified into different categories based on the sequence separation of the atoms involved.

Description	Value
Total distance restraints	2960
Intra-residue (|i-j|=0)	725
Sequential (|i-j|=1)	694
Medium range (|i-j|>1 and |i-j|<5)	561
Long range (|i-j|≥5)	980
Inter-chain	0
Hydrogen bond restraints	0
Disulfide bond restraints	0
Total dihedral-angle restraints	182
Number of unmapped restraints	0
Number of restraints per residue	30.2
Number of long range restraints per residue[1]	9.4

[1]Long range hydrogen bonds and disulfide bonds are counted as long range restraints while calculating the number of long range restraints per residue

8.2 Residual restraint violations (i)

This section provides the overview of the restraint violations analysis. The violations are binned as small, medium and large violations based on its absolute value. Average number of violations per model is calculated by dividing the total number of violations in each bin by the size of the ensemble.

8.2.1 Average number of distance violations per model (i)

Distance violations less than 0.1 Å are not included in the calculation.

Bins (Å)	Average number of violations per model	Max (Å)
0.1-0.2 (Small)	56.5	0.2
0.2-0.5 (Medium)	71.6	0.5
>0.5 (Large)	40.8	1.76

8.2.2 Average number of dihedral-angle violations per model (i)

Dihedral-angle violations less than 1° are not included in the calculation.

Bins (°)	Average number of violations per model	Max (°)
1.0-10.0 (Small)	38.7	10.0
10.0-20.0 (Medium)	2.8	14.2
>20.0 (Large)	None	None

9 Distance violation analysis (i)

9.1 Summary of distance violations (i)

The following table shows the summary of distance violations in different restraint categories based on the sequence separation of the atoms involved. Each category is further sub-divided into three sub-categories based on the atoms involved. Violations less than 0.1 Å are not included in the statistics.

Restraints type	Count	%[1]	Violated[3]			Consistently Violated[4]		
			Count	%[2]	%[1]	Count	%[2]	%[1]
Intra-residue (|i-j|=0)	725	24.5	71	9.8	2.4	24	3.3	0.8
Backbone-Backbone	92	3.1	1	1.1	0.0	1	1.1	0.0
Backbone-Sidechain	461	15.6	55	11.9	1.9	14	3.0	0.5
Sidechain-Sidechain	172	5.8	15	8.7	0.5	9	5.2	0.3
Sequential (|i-j|=1)	694	23.4	55	7.9	1.9	14	2.0	0.5
Backbone-Backbone	218	7.4	8	3.7	0.3	4	1.8	0.1
Backbone-Sidechain	397	13.4	34	8.6	1.1	8	2.0	0.3
Sidechain-Sidechain	79	2.7	13	16.5	0.4	2	2.5	0.1
Medium range (|i-j|>1 & |i-j|<5)	561	19.0	74	13.2	2.5	16	2.9	0.5
Backbone-Backbone	160	5.4	11	6.9	0.4	1	0.6	0.0
Backbone-Sidechain	273	9.2	32	11.7	1.1	11	4.0	0.4
Sidechain-Sidechain	128	4.3	31	24.2	1.0	4	3.1	0.1
Long range (|i-j|≥5)	980	33.1	136	13.9	4.6	22	2.2	0.7
Backbone-Backbone	128	4.3	7	5.5	0.2	1	0.8	0.0
Backbone-Sidechain	451	15.2	41	9.1	1.4	4	0.9	0.1
Sidechain-Sidechain	401	13.5	88	21.9	3.0	17	4.2	0.6
Inter-chain	0	0.0	0	0.0	0.0	0	0.0	0.0
Backbone-Backbone	0	0.0	0	0.0	0.0	0	0.0	0.0
Backbone-Sidechain	0	0.0	0	0.0	0.0	0	0.0	0.0
Sidechain-Sidechain	0	0.0	0	0.0	0.0	0	0.0	0.0
Hydrogen bond	0	0.0	0	0.0	0.0	0	0.0	0.0
Disulfide bond	0	0.0	0	0.0	0.0	0	0.0	0.0
Total	2960	100.0	336	11.4	11.4	76	2.6	2.6
Backbone-Backbone	598	20.2	27	4.5	0.9	7	1.2	0.2
Backbone-Sidechain	1582	53.4	162	10.2	5.5	37	2.3	1.2
Sidechain-Sidechain	780	26.4	147	18.8	5.0	32	4.1	1.1

[1] percentage calculated with respect to the total number of distance restraints, [2] percentage calculated with respect to the number of restraints in a particular restraint category, [3] violated in at least one model, [4] violated in all the models

9.1.1 Bar chart : Distribution of distance restraints and violations (i)

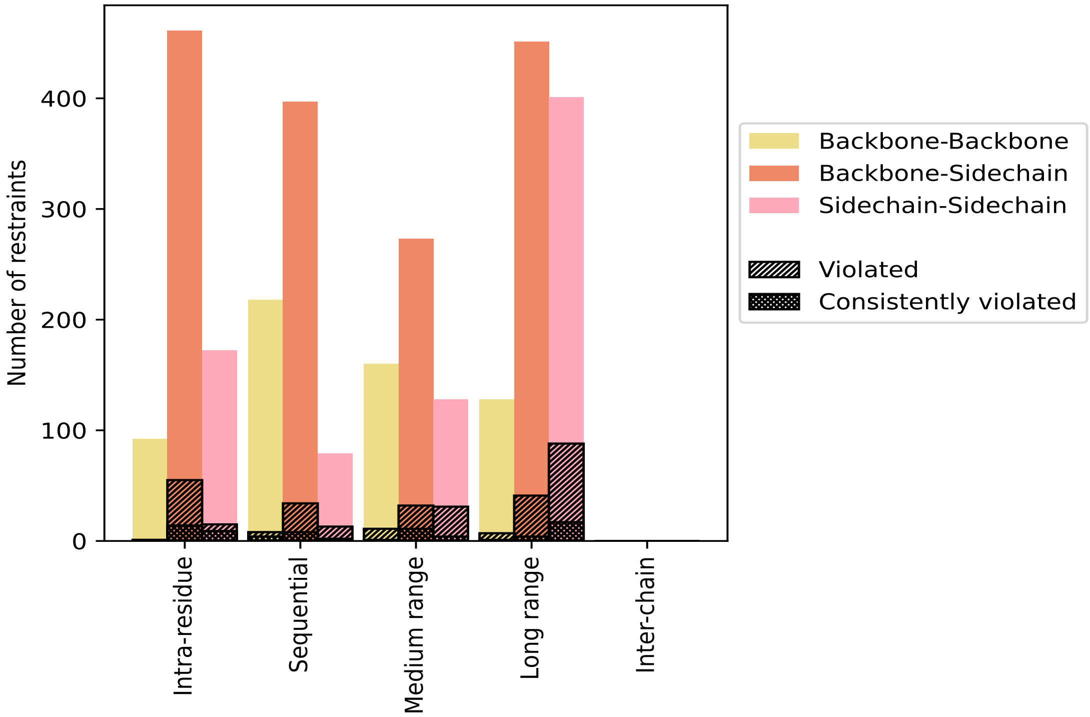

Violated and consistently violated restraints are shown using different hatch patterns in their respective categories. The hydrogen bonds and disulfied bonds are counted in their appropriate category on the x-axis

9.2 Distance violation statistics for each model (i)

The following table provides the distance violation statistics for each model in the ensemble. Violations less than 0.1 Å are not included in the statistics.

Model ID	Number of violations						Mean (Å)	Max (Å)	SD[6] (Å)	Median (Å)
	IR[1]	SQ[2]	MR[3]	LR[4]	IC[5]	Total				
1	43	21	37	64	0	165	0.39	1.71	0.3	0.31
2	45	25	34	62	0	166	0.37	1.75	0.28	0.29
3	41	22	34	61	0	158	0.42	1.73	0.3	0.37
4	48	28	40	58	0	174	0.36	1.73	0.29	0.27
5	40	26	38	62	0	166	0.37	1.73	0.3	0.28
6	39	28	42	67	0	176	0.39	1.75	0.29	0.31
7	42	29	36	69	0	176	0.38	1.75	0.29	0.3
8	44	30	39	66	0	179	0.38	1.76	0.29	0.31
9	41	23	37	57	0	158	0.4	1.72	0.28	0.32
10	45	27	35	64	0	171	0.39	1.74	0.29	0.32

[1]Intra-residue restraints, [2]Sequential restraints, [3]Medium range restraints, [4]Long range restraints,

[5]Inter-chain restraints, [6]Standard deviation

9.2.1 Bar graph : Distance Violation statistics for each model (i)

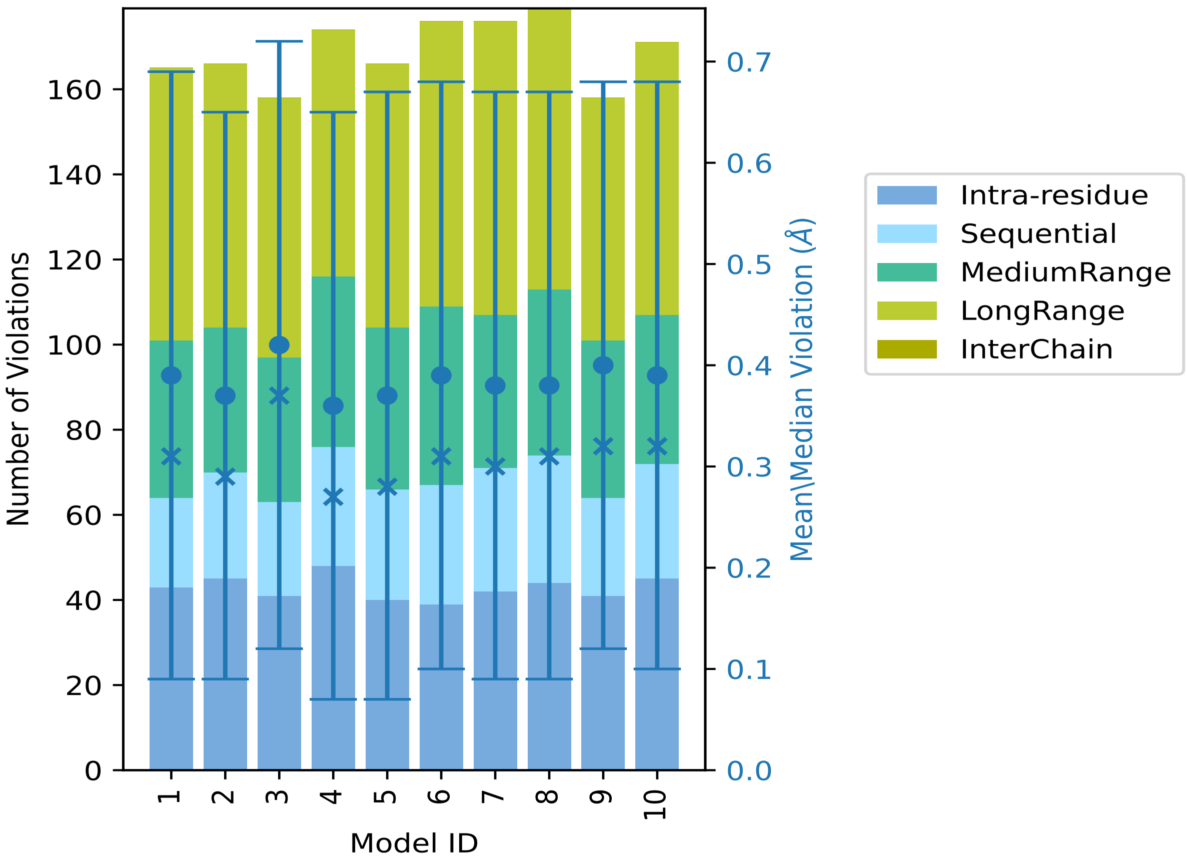

The mean(dot),median(x) and the standard deviation are shown in blue with respect to the y axis on the right

9.3 Distance violation statistics for the ensemble (i)

Violation analysis may find that some restraints are violated in few models and some are violated in most of models. The following table provides this information as number of violated restraints for a given fraction of the ensemble. In total, 2624(IR:654, SQ:639, MR:487, LR:844, IC:0) restraints are not violated in the ensemble.

Number of violated restraints						Fraction of the ensemble	
IR[1]	SQ[2]	MR[3]	LR[4]	IC[5]	Total	Count[6]	%
12	18	18	36	0	84	1	10.0
7	5	12	16	0	40	2	20.0
6	4	4	14	0	28	3	30.0
3	4	4	11	0	22	4	40.0

Continued on next page...

Continued from previous page...

Number of violated restraints						Fraction of the ensemble	
IR[1]	SQ[2]	MR[3]	LR[4]	IC[5]	Total	Count[6]	%
6	4	5	6	0	21	5	50.0
2	1	3	10	0	16	6	60.0
1	3	3	7	0	14	7	70.0
7	2	3	9	0	21	8	80.0
3	0	6	5	0	14	9	90.0
24	14	16	22	0	76	10	100.0

[1]Intra-residue restraints, [2]Sequential restraints, [3]Medium range restraints, [4]Long range restraints, [5]Inter-chain restraints, [6] Number of models with violations

9.3.1 Bar graph : Distance violation statistics for the ensemble (i)

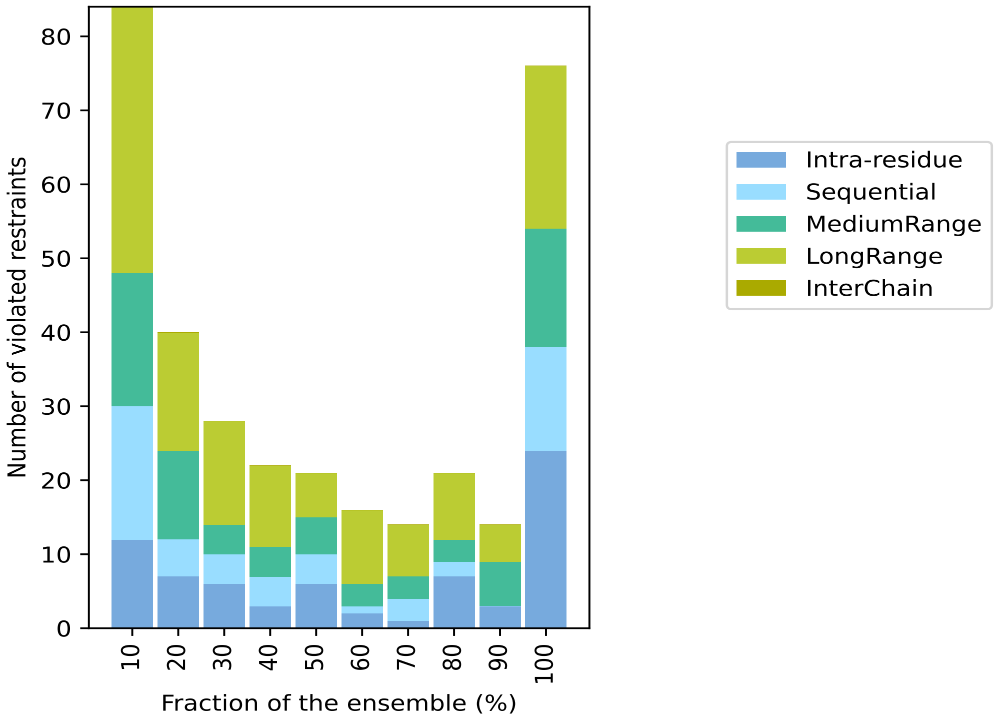

9.4 Most violated distance restraints in the ensemble (i)

9.4.1 Histogram : Distribution of mean distance violations (i)

The following histogram shows the distribution of the average value of the violation. The average is calculated for each restraint that is violated in more than one model over all the violated models in the ensemble

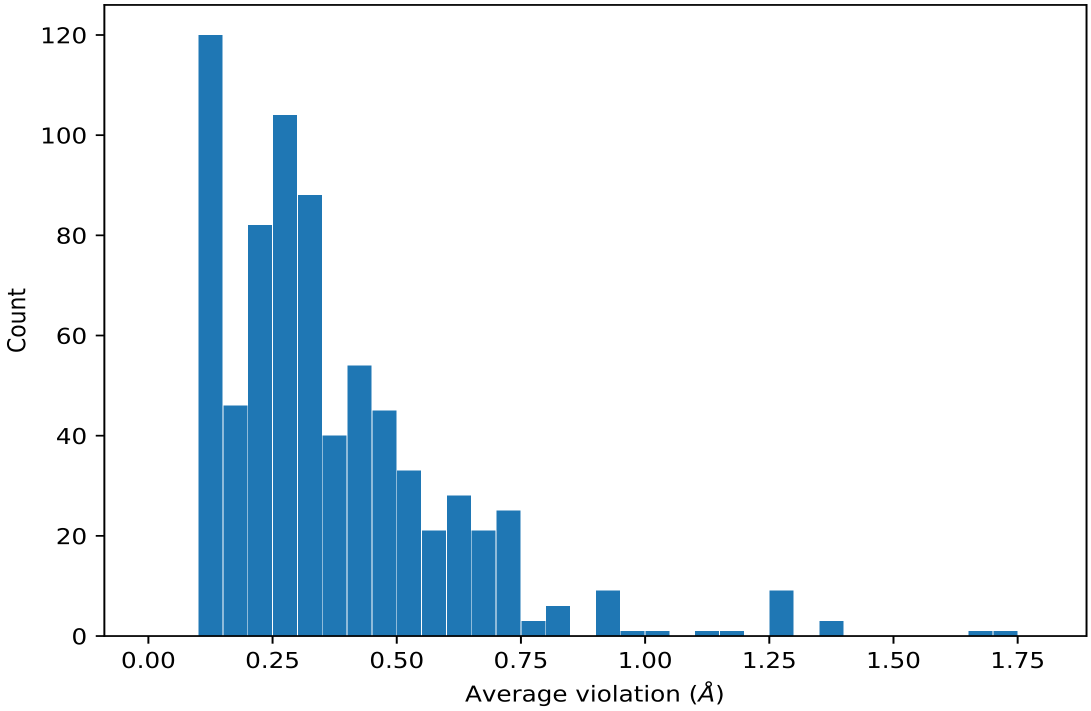

9.4.2 Table: Most violated distance restraints (i)

The following table provides the mean and the standard deviation of the violation for each restraint sorted by number of violated models and the mean value. The Key (restraint list ID, restraint ID) is the unique identifier for a given restraint. Rows with same key represent combinatorial or ambiguous restraints and are counted as a single restraint.

Key	Atom-1	Atom-2	Models[1]	Mean (Å)	SD[1] (Å)	Median (Å)
(2,397)	1:A:26:PHE:HA	1:A:27:ALA:H	10	1.74	0.01	1.74
(2,410)	1:A:29:TRP:H	1:A:29:TRP:HA	10	1.7	0.01	1.7
(2,400)	1:A:27:ALA:HB1	1:A:29:TRP:H	10	1.37	0.01	1.37
(2,400)	1:A:27:ALA:HB2	1:A:29:TRP:H	10	1.37	0.01	1.37
(2,400)	1:A:27:ALA:HB3	1:A:29:TRP:H	10	1.37	0.01	1.37
(2,406)	1:A:29:TRP:HA	1:A:30:CYS:H	10	1.16	0.02	1.16
(2,407)	1:A:29:TRP:HD1	1:A:29:TRP:H	10	1.14	0.02	1.15
(2,403)	1:A:28:GLU:HA	1:A:29:TRP:H	10	1.01	0.02	1.02
(2,405)	1:A:28:GLU:H	1:A:29:TRP:H	10	0.98	0.01	0.99
(2,238)	1:A:46:THR:HG21	1:A:104:ALA:HB1	10	0.95	0.38	1.03
(2,238)	1:A:46:THR:HG21	1:A:104:ALA:HB2	10	0.95	0.38	1.03
(2,238)	1:A:46:THR:HG21	1:A:104:ALA:HB3	10	0.95	0.38	1.03
(2,238)	1:A:46:THR:HG22	1:A:104:ALA:HB1	10	0.95	0.38	1.03
(2,238)	1:A:46:THR:HG22	1:A:104:ALA:HB2	10	0.95	0.38	1.03
(2,238)	1:A:46:THR:HG22	1:A:104:ALA:HB3	10	0.95	0.38	1.03
(2,238)	1:A:46:THR:HG23	1:A:104:ALA:HB1	10	0.95	0.38	1.03

Continued on next page...

Continued from previous page...

Key	Atom-1	Atom-2	Models[1]	Mean (Å)	SD[1] (Å)	Median (Å)
(2,238)	1:A:46:THR:HG23	1:A:104:ALA:HB2	10	0.95	0.38	1.03
(2,238)	1:A:46:THR:HG23	1:A:104:ALA:HB3	10	0.95	0.38	1.03
(2,326)	1:A:69:ILE:HG21	1:A:72:MET:HA	10	0.84	0.05	0.85
(2,326)	1:A:69:ILE:HG22	1:A:72:MET:HA	10	0.84	0.05	0.85
(2,326)	1:A:69:ILE:HG23	1:A:72:MET:HA	10	0.84	0.05	0.85
(2,49)	1:A:5:VAL:HG21	1:A:7:SER:HA	10	0.8	0.04	0.82
(2,49)	1:A:5:VAL:HG22	1:A:7:SER:HA	10	0.8	0.04	0.82
(2,49)	1:A:5:VAL:HG23	1:A:7:SER:HA	10	0.8	0.04	0.82
(2,199)	1:A:36:ILE:HG21	1:A:40:TYR:HD1	10	0.74	0.25	0.66
(2,199)	1:A:36:ILE:HG21	1:A:40:TYR:HD2	10	0.74	0.25	0.66
(2,199)	1:A:36:ILE:HG22	1:A:40:TYR:HD1	10	0.74	0.25	0.66
(2,199)	1:A:36:ILE:HG22	1:A:40:TYR:HD2	10	0.74	0.25	0.66
(2,199)	1:A:36:ILE:HG23	1:A:40:TYR:HD1	10	0.74	0.25	0.66
(2,199)	1:A:36:ILE:HG23	1:A:40:TYR:HD2	10	0.74	0.25	0.66
(2,328)	1:A:69:ILE:HG21	1:A:72:MET:HB2	10	0.74	0.18	0.84
(2,328)	1:A:69:ILE:HG22	1:A:72:MET:HB2	10	0.74	0.18	0.84
(2,328)	1:A:69:ILE:HG23	1:A:72:MET:HB2	10	0.74	0.18	0.84
(1,2542)	1:A:27:ALA:HB1	1:A:27:ALA:HA	10	0.73	0.0	0.73
(1,2542)	1:A:27:ALA:HB2	1:A:27:ALA:HA	10	0.73	0.0	0.73
(1,2542)	1:A:27:ALA:HB3	1:A:27:ALA:HA	10	0.73	0.0	0.73
(2,126)	1:A:25:PHE:HZ	1:A:69:ILE:HD11	10	0.72	0.11	0.7
(2,126)	1:A:25:PHE:HZ	1:A:69:ILE:HD12	10	0.72	0.11	0.7
(2,126)	1:A:25:PHE:HZ	1:A:69:ILE:HD13	10	0.72	0.11	0.7
(2,127)	1:A:25:PHE:HZ	1:A:69:ILE:HD11	10	0.72	0.11	0.7
(2,127)	1:A:25:PHE:HZ	1:A:69:ILE:HD12	10	0.72	0.11	0.7
(2,127)	1:A:25:PHE:HZ	1:A:69:ILE:HD13	10	0.72	0.11	0.7
(1,2546)	1:A:29:TRP:HD1	1:A:29:TRP:HA	10	0.71	0.03	0.7
(2,140)	1:A:26:PHE:HE1	1:A:54:LYS:HD2	10	0.69	0.17	0.72
(2,140)	1:A:26:PHE:HE1	1:A:54:LYS:HD3	10	0.69	0.17	0.72
(2,140)	1:A:26:PHE:HE2	1:A:54:LYS:HD2	10	0.69	0.17	0.72
(2,140)	1:A:26:PHE:HE2	1:A:54:LYS:HD3	10	0.69	0.17	0.72
(1,990)	1:A:33:CYS:HA	1:A:90:ALA:HB1	10	0.66	0.11	0.7
(1,990)	1:A:33:CYS:HA	1:A:90:ALA:HB2	10	0.66	0.11	0.7
(1,990)	1:A:33:CYS:HA	1:A:90:ALA:HB3	10	0.66	0.11	0.7
(2,257)	1:A:48:THR:HA	1:A:52:PHE:HD1	10	0.65	0.02	0.65
(2,257)	1:A:48:THR:HA	1:A:52:PHE:HD2	10	0.65	0.02	0.65
(1,1209)	1:A:40:TYR:HE1	1:A:95:LEU:HD11	10	0.63	0.15	0.7
(1,1209)	1:A:40:TYR:HE1	1:A:95:LEU:HD12	10	0.63	0.15	0.7
(1,1209)	1:A:40:TYR:HE1	1:A:95:LEU:HD13	10	0.63	0.15	0.7
(1,1209)	1:A:40:TYR:HE2	1:A:95:LEU:HD11	10	0.63	0.15	0.7
(1,1209)	1:A:40:TYR:HE2	1:A:95:LEU:HD12	10	0.63	0.15	0.7
(1,1209)	1:A:40:TYR:HE2	1:A:95:LEU:HD13	10	0.63	0.15	0.7

Continued on next page...

Continued from previous page...

Key	Atom-1	Atom-2	Models[1]	Mean (Å)	SD[1] (Å)	Median (Å)
(2,267)	1:A:51:VAL:H	1:A:52:PHE:HD1	10	0.63	0.02	0.63
(2,267)	1:A:51:VAL:H	1:A:52:PHE:HD2	10	0.63	0.02	0.63
(2,210)	1:A:40:TYR:HE1	1:A:52:PHE:HB3	10	0.61	0.02	0.61
(2,210)	1:A:40:TYR:HE2	1:A:52:PHE:HB3	10	0.61	0.02	0.61
(2,325)	1:A:69:ILE:HG21	1:A:71:SER:HA	10	0.61	0.2	0.61
(2,325)	1:A:69:ILE:HG22	1:A:71:SER:HA	10	0.61	0.2	0.61
(2,325)	1:A:69:ILE:HG23	1:A:71:SER:HA	10	0.61	0.2	0.61
(1,1392)	1:A:47:TYR:HE1	1:A:99:ILE:HG21	10	0.61	0.09	0.66
(1,1392)	1:A:47:TYR:HE1	1:A:99:ILE:HG22	10	0.61	0.09	0.66
(1,1392)	1:A:47:TYR:HE1	1:A:99:ILE:HG23	10	0.61	0.09	0.66
(1,1392)	1:A:47:TYR:HE2	1:A:99:ILE:HG21	10	0.61	0.09	0.66
(1,1392)	1:A:47:TYR:HE2	1:A:99:ILE:HG22	10	0.61	0.09	0.66
(1,1392)	1:A:47:TYR:HE2	1:A:99:ILE:HG23	10	0.61	0.09	0.66
(1,2423)	1:A:99:ILE:HG21	1:A:100:GLU:H	10	0.58	0.01	0.57
(1,2423)	1:A:99:ILE:HG22	1:A:100:GLU:H	10	0.58	0.01	0.57
(1,2423)	1:A:99:ILE:HG23	1:A:100:GLU:H	10	0.58	0.01	0.57
(2,74)	1:A:11:PHE:HD1	1:A:12:ASP:HB2	10	0.57	0.01	0.57
(2,74)	1:A:11:PHE:HD2	1:A:12:ASP:HB2	10	0.57	0.01	0.57
(2,409)	1:A:29:TRP:HE3	1:A:30:CYS:H	10	0.53	0.02	0.54
(2,118)	1:A:25:PHE:HD1	1:A:67:GLU:HG2	10	0.52	0.25	0.44
(2,118)	1:A:25:PHE:HD1	1:A:67:GLU:HG3	10	0.52	0.25	0.44
(2,118)	1:A:25:PHE:HD2	1:A:67:GLU:HG2	10	0.52	0.25	0.44
(2,118)	1:A:25:PHE:HD2	1:A:67:GLU:HG3	10	0.52	0.25	0.44
(2,224)	1:A:43:CYS:HB2	1:A:52:PHE:HD1	10	0.51	0.04	0.52
(2,224)	1:A:43:CYS:HB2	1:A:52:PHE:HD2	10	0.51	0.04	0.52
(2,163)	1:A:28:GLU:HG2	1:A:29:TRP:H	10	0.49	0.03	0.48
(2,163)	1:A:28:GLU:HG3	1:A:29:TRP:H	10	0.49	0.03	0.48
(2,359)	1:A:74:THR:H	1:A:75:PHE:HE1	10	0.49	0.1	0.55
(2,359)	1:A:74:THR:H	1:A:75:PHE:HE2	10	0.49	0.1	0.55
(1,424)	1:A:15:ILE:HG21	1:A:78:TYR:HB3	10	0.49	0.01	0.49
(1,424)	1:A:15:ILE:HG22	1:A:78:TYR:HB3	10	0.49	0.01	0.49
(1,424)	1:A:15:ILE:HG23	1:A:78:TYR:HB3	10	0.49	0.01	0.49
(1,1950)	1:A:77:VAL:HG11	1:A:84:VAL:HG11	10	0.46	0.09	0.5
(1,1950)	1:A:77:VAL:HG11	1:A:84:VAL:HG12	10	0.46	0.09	0.5
(1,1950)	1:A:77:VAL:HG11	1:A:84:VAL:HG13	10	0.46	0.09	0.5
(1,1950)	1:A:77:VAL:HG12	1:A:84:VAL:HG11	10	0.46	0.09	0.5
(1,1950)	1:A:77:VAL:HG12	1:A:84:VAL:HG12	10	0.46	0.09	0.5
(1,1950)	1:A:77:VAL:HG12	1:A:84:VAL:HG13	10	0.46	0.09	0.5
(1,1950)	1:A:77:VAL:HG13	1:A:84:VAL:HG11	10	0.46	0.09	0.5
(1,1950)	1:A:77:VAL:HG13	1:A:84:VAL:HG12	10	0.46	0.09	0.5
(1,1950)	1:A:77:VAL:HG13	1:A:84:VAL:HG13	10	0.46	0.09	0.5
(1,2544)	1:A:27:ALA:HB1	1:A:29:TRP:HE1	10	0.45	0.03	0.44

Continued on next page...

Continued from previous page...

Key	Atom-1	Atom-2	Models[1]	Mean (Å)	SD[1] (Å)	Median (Å)
(1,2544)	1:A:27:ALA:HB2	1:A:29:TRP:HE1	10	0.45	0.03	0.44
(1,2544)	1:A:27:ALA:HB3	1:A:29:TRP:HE1	10	0.45	0.03	0.44
(1,2310)	1:A:95:LEU:HD21	1:A:95:LEU:HB3	10	0.45	0.01	0.45
(1,2310)	1:A:95:LEU:HD22	1:A:95:LEU:HB3	10	0.45	0.01	0.45
(1,2310)	1:A:95:LEU:HD23	1:A:95:LEU:HB3	10	0.45	0.01	0.45
(1,577)	1:A:20:LEU:HD11	1:A:79:LYS:HA	10	0.45	0.05	0.46
(1,577)	1:A:20:LEU:HD12	1:A:79:LYS:HA	10	0.45	0.05	0.46
(1,577)	1:A:20:LEU:HD13	1:A:79:LYS:HA	10	0.45	0.05	0.46
(2,266)	1:A:50:MET:H	1:A:52:PHE:HD1	10	0.44	0.12	0.48
(2,266)	1:A:50:MET:H	1:A:52:PHE:HD2	10	0.44	0.12	0.48
(2,208)	1:A:40:TYR:HD1	1:A:41:GLU:H	10	0.44	0.03	0.42
(2,208)	1:A:40:TYR:HD2	1:A:41:GLU:H	10	0.44	0.03	0.42
(1,2123)	1:A:84:VAL:HG11	1:A:85:ASP:H	10	0.43	0.01	0.43
(1,2123)	1:A:84:VAL:HG12	1:A:85:ASP:H	10	0.43	0.01	0.43
(1,2123)	1:A:84:VAL:HG13	1:A:85:ASP:H	10	0.43	0.01	0.43
(1,1359)	1:A:47:TYR:HB3	1:A:103:ALA:HB1	10	0.42	0.04	0.42
(1,1359)	1:A:47:TYR:HB3	1:A:103:ALA:HB2	10	0.42	0.04	0.42
(1,1359)	1:A:47:TYR:HB3	1:A:103:ALA:HB3	10	0.42	0.04	0.42
(1,1916)	1:A:76:LYS:HD2	1:A:86:THR:HG21	10	0.42	0.04	0.4
(1,1916)	1:A:76:LYS:HD2	1:A:86:THR:HG22	10	0.42	0.04	0.4
(1,1916)	1:A:76:LYS:HD2	1:A:86:THR:HG23	10	0.42	0.04	0.4
(1,1916)	1:A:76:LYS:HD3	1:A:86:THR:HG21	10	0.42	0.04	0.4
(1,1916)	1:A:76:LYS:HD3	1:A:86:THR:HG22	10	0.42	0.04	0.4
(1,1916)	1:A:76:LYS:HD3	1:A:86:THR:HG23	10	0.42	0.04	0.4
(1,89)	1:A:5:VAL:HG21	1:A:7:SER:H	10	0.42	0.01	0.42
(1,89)	1:A:5:VAL:HG22	1:A:7:SER:H	10	0.42	0.01	0.42
(1,89)	1:A:5:VAL:HG23	1:A:7:SER:H	10	0.42	0.01	0.42
(1,555)	1:A:20:LEU:HA	1:A:20:LEU:HD11	10	0.41	0.05	0.44
(1,555)	1:A:20:LEU:HA	1:A:20:LEU:HD12	10	0.41	0.05	0.44
(1,555)	1:A:20:LEU:HA	1:A:20:LEU:HD13	10	0.41	0.05	0.44
(1,97)	1:A:5:VAL:H	1:A:5:VAL:HG11	10	0.41	0.04	0.42
(1,97)	1:A:5:VAL:H	1:A:5:VAL:HG12	10	0.41	0.04	0.42
(1,97)	1:A:5:VAL:H	1:A:5:VAL:HG13	10	0.41	0.04	0.42
(2,9)	1:A:1:SER:HB2	1:A:2:VAL:HA	10	0.4	0.06	0.4
(2,9)	1:A:1:SER:HB3	1:A:2:VAL:HA	10	0.4	0.06	0.4
(1,1933)	1:A:77:VAL:HB	1:A:84:VAL:HG11	10	0.4	0.04	0.42
(1,1933)	1:A:77:VAL:HB	1:A:84:VAL:HG12	10	0.4	0.04	0.42
(1,1933)	1:A:77:VAL:HB	1:A:84:VAL:HG13	10	0.4	0.04	0.42
(2,134)	1:A:26:PHE:HA	1:A:75:PHE:HE1	10	0.38	0.1	0.37
(2,134)	1:A:26:PHE:HA	1:A:75:PHE:HE2	10	0.38	0.1	0.37
(1,1851)	1:A:75:PHE:HB2	1:A:87:LEU:HD21	10	0.38	0.05	0.41
(1,1851)	1:A:75:PHE:HB2	1:A:87:LEU:HD22	10	0.38	0.05	0.41

Continued on next page...

Continued from previous page...

Key	Atom-1	Atom-2	Models[1]	Mean (Å)	SD[1] (Å)	Median (Å)
(1,1851)	1:A:75:PHE:HB2	1:A:87:LEU:HD23	10	0.38	0.05	0.41
(1,1036)	1:A:36:ILE:HA	1:A:36:ILE:HD11	10	0.38	0.03	0.38
(1,1036)	1:A:36:ILE:HA	1:A:36:ILE:HD12	10	0.38	0.03	0.38
(1,1036)	1:A:36:ILE:HA	1:A:36:ILE:HD13	10	0.38	0.03	0.38
(1,1267)	1:A:43:CYS:HB3	1:A:99:ILE:HG21	10	0.36	0.09	0.4
(1,1267)	1:A:43:CYS:HB3	1:A:99:ILE:HG22	10	0.36	0.09	0.4
(1,1267)	1:A:43:CYS:HB3	1:A:99:ILE:HG23	10	0.36	0.09	0.4
(2,408)	1:A:29:TRP:HE1	1:A:29:TRP:H	10	0.34	0.01	0.34
(1,1812)	1:A:74:THR:HG1	1:A:74:THR:HG21	10	0.3	0.05	0.31
(1,1812)	1:A:74:THR:HG1	1:A:74:THR:HG22	10	0.3	0.05	0.31
(1,1812)	1:A:74:THR:HG1	1:A:74:THR:HG23	10	0.3	0.05	0.31
(2,404)	1:A:28:GLU:HA	1:A:30:CYS:H	10	0.29	0.03	0.3
(1,154)	1:A:8:GLN:HE22	1:A:11:PHE:HD1	10	0.29	0.01	0.29
(1,154)	1:A:8:GLN:HE22	1:A:11:PHE:HD2	10	0.29	0.01	0.29
(1,30)	1:A:2:VAL:H	1:A:2:VAL:HG21	10	0.29	0.1	0.32
(1,30)	1:A:2:VAL:H	1:A:2:VAL:HG22	10	0.29	0.1	0.32
(1,30)	1:A:2:VAL:H	1:A:2:VAL:HG23	10	0.29	0.1	0.32
(1,1088)	1:A:36:ILE:HG21	1:A:75:PHE:HZ	10	0.28	0.03	0.27
(1,1088)	1:A:36:ILE:HG22	1:A:75:PHE:HZ	10	0.28	0.03	0.27
(1,1088)	1:A:36:ILE:HG23	1:A:75:PHE:HZ	10	0.28	0.03	0.27
(1,927)	1:A:27:ALA:HA	1:A:29:TRP:HE1	10	0.28	0.01	0.28
(1,2303)	1:A:95:LEU:HD11	1:A:95:LEU:HB2	10	0.27	0.03	0.27
(1,2303)	1:A:95:LEU:HD12	1:A:95:LEU:HB2	10	0.27	0.03	0.27
(1,2303)	1:A:95:LEU:HD13	1:A:95:LEU:HB2	10	0.27	0.03	0.27
(1,2304)	1:A:95:LEU:HD11	1:A:95:LEU:HB2	10	0.27	0.03	0.27
(1,2304)	1:A:95:LEU:HD12	1:A:95:LEU:HB2	10	0.27	0.03	0.27
(1,2304)	1:A:95:LEU:HD13	1:A:95:LEU:HB2	10	0.27	0.03	0.27
(2,402)	1:A:27:ALA:HB1	1:A:30:CYS:H	10	0.26	0.09	0.26
(2,402)	1:A:27:ALA:HB2	1:A:30:CYS:H	10	0.26	0.09	0.26
(2,402)	1:A:27:ALA:HB3	1:A:30:CYS:H	10	0.26	0.09	0.26
(2,398)	1:A:26:PHE:HD1	1:A:27:ALA:HB1	10	0.26	0.05	0.27
(2,398)	1:A:26:PHE:HD1	1:A:27:ALA:HB2	10	0.26	0.05	0.27
(2,398)	1:A:26:PHE:HD1	1:A:27:ALA:HB3	10	0.26	0.05	0.27
(2,398)	1:A:26:PHE:HD2	1:A:27:ALA:HB1	10	0.26	0.05	0.27
(2,398)	1:A:26:PHE:HD2	1:A:27:ALA:HB2	10	0.26	0.05	0.27
(2,398)	1:A:26:PHE:HD2	1:A:27:ALA:HB3	10	0.26	0.05	0.27
(1,1816)	1:A:74:THR:HG21	1:A:74:THR:HA	10	0.25	0.04	0.24
(1,1816)	1:A:74:THR:HG22	1:A:74:THR:HA	10	0.25	0.04	0.24
(1,1816)	1:A:74:THR:HG23	1:A:74:THR:HA	10	0.25	0.04	0.24
(1,684)	1:A:22:ILE:H	1:A:22:ILE:HG21	10	0.24	0.09	0.21
(1,684)	1:A:22:ILE:H	1:A:22:ILE:HG22	10	0.24	0.09	0.21
(1,684)	1:A:22:ILE:H	1:A:22:ILE:HG23	10	0.24	0.09	0.21

Continued on next page...

Continued from previous page...

Key	Atom-1	Atom-2	Models[1]	Mean (Å)	SD[1] (Å)	Median (Å)
(1,936)	1:A:27:ALA:H	1:A:27:ALA:HB1	10	0.22	0.01	0.22
(1,936)	1:A:27:ALA:H	1:A:27:ALA:HB2	10	0.22	0.01	0.22
(1,936)	1:A:27:ALA:H	1:A:27:ALA:HB3	10	0.22	0.01	0.22
(1,913)	1:A:26:PHE:H	1:A:26:PHE:HD1	10	0.2	0.05	0.22
(1,913)	1:A:26:PHE:H	1:A:26:PHE:HD2	10	0.2	0.05	0.22
(1,1963)	1:A:77:VAL:HG21	1:A:77:VAL:HA	10	0.18	0.03	0.19
(1,1963)	1:A:77:VAL:HG22	1:A:77:VAL:HA	10	0.18	0.03	0.19
(1,1963)	1:A:77:VAL:HG23	1:A:77:VAL:HA	10	0.18	0.03	0.19
(1,433)	1:A:15:ILE:HG21	1:A:82:SER:HA	10	0.18	0.04	0.18
(1,433)	1:A:15:ILE:HG22	1:A:82:SER:HA	10	0.18	0.04	0.18
(1,433)	1:A:15:ILE:HG23	1:A:82:SER:HA	10	0.18	0.04	0.18
(1,735)	1:A:23:VAL:HG21	1:A:23:VAL:HG11	10	0.16	0.07	0.14
(1,735)	1:A:23:VAL:HG21	1:A:23:VAL:HG12	10	0.16	0.07	0.14
(1,735)	1:A:23:VAL:HG21	1:A:23:VAL:HG13	10	0.16	0.07	0.14
(1,735)	1:A:23:VAL:HG22	1:A:23:VAL:HG11	10	0.16	0.07	0.14
(1,735)	1:A:23:VAL:HG22	1:A:23:VAL:HG12	10	0.16	0.07	0.14
(1,735)	1:A:23:VAL:HG22	1:A:23:VAL:HG13	10	0.16	0.07	0.14
(1,735)	1:A:23:VAL:HG23	1:A:23:VAL:HG11	10	0.16	0.07	0.14
(1,735)	1:A:23:VAL:HG23	1:A:23:VAL:HG12	10	0.16	0.07	0.14
(1,735)	1:A:23:VAL:HG23	1:A:23:VAL:HG13	10	0.16	0.07	0.14
(1,594)	1:A:20:LEU:H	1:A:50:MET:HA	10	0.16	0.03	0.16
(1,979)	1:A:32:PRO:HB3	1:A:36:ILE:HA	10	0.15	0.03	0.15
(1,1938)	1:A:77:VAL:HG11	1:A:77:VAL:HA	10	0.15	0.03	0.15
(1,1938)	1:A:77:VAL:HG12	1:A:77:VAL:HA	10	0.15	0.03	0.15
(1,1938)	1:A:77:VAL:HG13	1:A:77:VAL:HA	10	0.15	0.03	0.15
(1,2243)	1:A:91:ASN:HD21	1:A:91:ASN:HD22	10	0.15	0.0	0.15
(1,1940)	1:A:77:VAL:HG11	1:A:77:VAL:HG21	10	0.15	0.02	0.15
(1,1940)	1:A:77:VAL:HG11	1:A:77:VAL:HG22	10	0.15	0.02	0.15
(1,1940)	1:A:77:VAL:HG11	1:A:77:VAL:HG23	10	0.15	0.02	0.15
(1,1940)	1:A:77:VAL:HG12	1:A:77:VAL:HG21	10	0.15	0.02	0.15
(1,1940)	1:A:77:VAL:HG12	1:A:77:VAL:HG22	10	0.15	0.02	0.15
(1,1940)	1:A:77:VAL:HG12	1:A:77:VAL:HG23	10	0.15	0.02	0.15
(1,1940)	1:A:77:VAL:HG13	1:A:77:VAL:HG21	10	0.15	0.02	0.15
(1,1940)	1:A:77:VAL:HG13	1:A:77:VAL:HG22	10	0.15	0.02	0.15
(1,1940)	1:A:77:VAL:HG13	1:A:77:VAL:HG23	10	0.15	0.02	0.15
(1,400)	1:A:15:ILE:HG13	1:A:17:GLN:H	10	0.14	0.01	0.14
(1,1200)	1:A:40:TYR:HB2	1:A:40:TYR:HB3	10	0.13	0.0	0.13
(1,1625)	1:A:63:VAL:HG11	1:A:63:VAL:HB	10	0.11	0.0	0.11
(1,1625)	1:A:63:VAL:HG12	1:A:63:VAL:HB	10	0.11	0.0	0.11
(1,1625)	1:A:63:VAL:HG13	1:A:63:VAL:HB	10	0.11	0.0	0.11
(2,245)	1:A:47:TYR:HB3	1:A:50:MET:HE1	9	0.77	0.16	0.84
(2,245)	1:A:47:TYR:HB3	1:A:50:MET:HE2	9	0.77	0.16	0.84

Continued on next page...

Continued from previous page...

Key	Atom-1	Atom-2	Models[1]	Mean (Å)	SD[1] (Å)	Median (Å)
(2,245)	1:A:47:TYR:HB3	1:A:50:MET:HE3	9	0.77	0.16	0.84
(1,575)	1:A:20:LEU:HD11	1:A:77:VAL:HG11	9	0.68	0.11	0.72
(1,575)	1:A:20:LEU:HD11	1:A:77:VAL:HG12	9	0.68	0.11	0.72
(1,575)	1:A:20:LEU:HD11	1:A:77:VAL:HG13	9	0.68	0.11	0.72
(1,575)	1:A:20:LEU:HD12	1:A:77:VAL:HG11	9	0.68	0.11	0.72
(1,575)	1:A:20:LEU:HD12	1:A:77:VAL:HG12	9	0.68	0.11	0.72
(1,575)	1:A:20:LEU:HD12	1:A:77:VAL:HG13	9	0.68	0.11	0.72
(1,575)	1:A:20:LEU:HD13	1:A:77:VAL:HG11	9	0.68	0.11	0.72
(1,575)	1:A:20:LEU:HD13	1:A:77:VAL:HG12	9	0.68	0.11	0.72
(1,575)	1:A:20:LEU:HD13	1:A:77:VAL:HG13	9	0.68	0.11	0.72
(1,576)	1:A:20:LEU:HD11	1:A:78:TYR:H	9	0.45	0.14	0.46
(1,576)	1:A:20:LEU:HD12	1:A:78:TYR:H	9	0.45	0.14	0.46
(1,576)	1:A:20:LEU:HD13	1:A:78:TYR:H	9	0.45	0.14	0.46
(1,712)	1:A:23:VAL:HG11	1:A:25:PHE:HZ	9	0.44	0.05	0.46
(1,712)	1:A:23:VAL:HG12	1:A:25:PHE:HZ	9	0.44	0.05	0.46
(1,712)	1:A:23:VAL:HG13	1:A:25:PHE:HZ	9	0.44	0.05	0.46
(1,1741)	1:A:69:ILE:HD11	1:A:69:ILE:HG21	9	0.35	0.1	0.34
(1,1741)	1:A:69:ILE:HD11	1:A:69:ILE:HG22	9	0.35	0.1	0.34
(1,1741)	1:A:69:ILE:HD11	1:A:69:ILE:HG23	9	0.35	0.1	0.34
(1,1741)	1:A:69:ILE:HD12	1:A:69:ILE:HG21	9	0.35	0.1	0.34
(1,1741)	1:A:69:ILE:HD12	1:A:69:ILE:HG22	9	0.35	0.1	0.34
(1,1741)	1:A:69:ILE:HD12	1:A:69:ILE:HG23	9	0.35	0.1	0.34
(1,1741)	1:A:69:ILE:HD13	1:A:69:ILE:HG21	9	0.35	0.1	0.34
(1,1741)	1:A:69:ILE:HD13	1:A:69:ILE:HG22	9	0.35	0.1	0.34
(1,1741)	1:A:69:ILE:HD13	1:A:69:ILE:HG23	9	0.35	0.1	0.34
(2,287)	1:A:57:VAL:HG21	1:A:61:SER:HA	9	0.34	0.08	0.35
(2,287)	1:A:57:VAL:HG22	1:A:61:SER:HA	9	0.34	0.08	0.35
(2,287)	1:A:57:VAL:HG23	1:A:61:SER:HA	9	0.34	0.08	0.35
(1,1263)	1:A:43:CYS:HB3	1:A:47:TYR:HD1	9	0.33	0.06	0.34
(1,1263)	1:A:43:CYS:HB3	1:A:47:TYR:HD2	9	0.33	0.06	0.34
(1,227)	1:A:11:PHE:HE1	1:A:23:VAL:HG11	9	0.23	0.07	0.23
(1,227)	1:A:11:PHE:HE1	1:A:23:VAL:HG12	9	0.23	0.07	0.23
(1,227)	1:A:11:PHE:HE1	1:A:23:VAL:HG13	9	0.23	0.07	0.23
(1,227)	1:A:11:PHE:HE2	1:A:23:VAL:HG11	9	0.23	0.07	0.23
(1,227)	1:A:11:PHE:HE2	1:A:23:VAL:HG12	9	0.23	0.07	0.23
(1,227)	1:A:11:PHE:HE2	1:A:23:VAL:HG13	9	0.23	0.07	0.23
(1,1413)	1:A:48:THR:H	1:A:48:THR:HG21	9	0.21	0.03	0.21
(1,1413)	1:A:48:THR:H	1:A:48:THR:HG22	9	0.21	0.03	0.21
(1,1413)	1:A:48:THR:H	1:A:48:THR:HG23	9	0.21	0.03	0.21
(2,411)	1:A:33:CYS:H	1:A:36:ILE:H	9	0.19	0.02	0.19
(1,697)	1:A:22:ILE:H	1:A:53:ILE:H	9	0.15	0.02	0.14
(2,91)	1:A:15:ILE:HA	1:A:18:ASN:H	9	0.14	0.02	0.14

Continued on next page...

Continued from previous page...

Key	Atom-1	Atom-2	Models[1]	Mean (Å)	SD[1] (Å)	Median (Å)
(1,2547)	1:A:29:TRP:HE3	1:A:29:TRP:HA	9	0.14	0.02	0.13
(1,524)	1:A:19:GLU:HA	1:A:79:LYS:HA	9	0.12	0.01	0.12
(2,327)	1:A:69:ILE:HG21	1:A:72:MET:HB3	8	0.73	0.3	0.9
(2,327)	1:A:69:ILE:HG22	1:A:72:MET:HB3	8	0.73	0.3	0.9
(2,327)	1:A:69:ILE:HG23	1:A:72:MET:HB3	8	0.73	0.3	0.9
(2,32)	1:A:3:LYS:HD2	1:A:53:ILE:HA	8	0.58	0.02	0.58
(2,32)	1:A:3:LYS:HD3	1:A:53:ILE:HA	8	0.58	0.02	0.58
(2,296)	1:A:67:GLU:HB3	1:A:78:TYR:HE1	8	0.45	0.15	0.55
(2,296)	1:A:67:GLU:HB3	1:A:78:TYR:HE2	8	0.45	0.15	0.55
(1,2351)	1:A:97:GLN:HE21	1:A:97:GLN:HB2	8	0.36	0.1	0.39
(1,2351)	1:A:97:GLN:HE21	1:A:97:GLN:HB3	8	0.36	0.1	0.39
(2,247)	1:A:47:TYR:HB2	1:A:52:PHE:HD1	8	0.35	0.14	0.31
(2,247)	1:A:47:TYR:HB2	1:A:52:PHE:HD2	8	0.35	0.14	0.31
(1,366)	1:A:15:ILE:HD11	1:A:15:ILE:HG21	8	0.35	0.07	0.37
(1,366)	1:A:15:ILE:HD11	1:A:15:ILE:HG22	8	0.35	0.07	0.37
(1,366)	1:A:15:ILE:HD11	1:A:15:ILE:HG23	8	0.35	0.07	0.37
(1,366)	1:A:15:ILE:HD12	1:A:15:ILE:HG21	8	0.35	0.07	0.37
(1,366)	1:A:15:ILE:HD12	1:A:15:ILE:HG22	8	0.35	0.07	0.37
(1,366)	1:A:15:ILE:HD12	1:A:15:ILE:HG23	8	0.35	0.07	0.37
(1,366)	1:A:15:ILE:HD13	1:A:15:ILE:HG21	8	0.35	0.07	0.37
(1,366)	1:A:15:ILE:HD13	1:A:15:ILE:HG22	8	0.35	0.07	0.37
(1,366)	1:A:15:ILE:HD13	1:A:15:ILE:HG23	8	0.35	0.07	0.37
(1,412)	1:A:15:ILE:HG21	1:A:15:ILE:HD11	8	0.35	0.07	0.37
(1,412)	1:A:15:ILE:HG21	1:A:15:ILE:HD12	8	0.35	0.07	0.37
(1,412)	1:A:15:ILE:HG21	1:A:15:ILE:HD13	8	0.35	0.07	0.37
(1,412)	1:A:15:ILE:HG22	1:A:15:ILE:HD11	8	0.35	0.07	0.37
(1,412)	1:A:15:ILE:HG22	1:A:15:ILE:HD12	8	0.35	0.07	0.37
(1,412)	1:A:15:ILE:HG22	1:A:15:ILE:HD13	8	0.35	0.07	0.37
(1,412)	1:A:15:ILE:HG23	1:A:15:ILE:HD11	8	0.35	0.07	0.37
(1,412)	1:A:15:ILE:HG23	1:A:15:ILE:HD12	8	0.35	0.07	0.37
(1,412)	1:A:15:ILE:HG23	1:A:15:ILE:HD13	8	0.35	0.07	0.37
(1,749)	1:A:23:VAL:HG21	1:A:54:LYS:HA	8	0.33	0.12	0.32
(1,749)	1:A:23:VAL:HG22	1:A:54:LYS:HA	8	0.33	0.12	0.32
(1,749)	1:A:23:VAL:HG23	1:A:54:LYS:HA	8	0.33	0.12	0.32
(1,1421)	1:A:49:LYS:H	1:A:49:LYS:HD2	8	0.29	0.03	0.29
(1,1421)	1:A:49:LYS:H	1:A:49:LYS:HD3	8	0.29	0.03	0.29
(1,112)	1:A:6:THR:H	1:A:6:THR:HG21	8	0.26	0.05	0.26
(1,112)	1:A:6:THR:H	1:A:6:THR:HG22	8	0.26	0.05	0.26
(1,112)	1:A:6:THR:H	1:A:6:THR:HG23	8	0.26	0.05	0.26
(1,380)	1:A:15:ILE:HD11	1:A:23:VAL:HB	8	0.26	0.05	0.25
(1,380)	1:A:15:ILE:HD12	1:A:23:VAL:HB	8	0.26	0.05	0.25
(1,380)	1:A:15:ILE:HD13	1:A:23:VAL:HB	8	0.26	0.05	0.25

Continued on next page...

Continued from previous page...

Key	Atom-1	Atom-2	Models[1]	Mean (Å)	SD[1] (Å)	Median (Å)
(1,764)	1:A:23:VAL:H	1:A:23:VAL:HG11	8	0.23	0.08	0.2
(1,764)	1:A:23:VAL:H	1:A:23:VAL:HG12	8	0.23	0.08	0.2
(1,764)	1:A:23:VAL:H	1:A:23:VAL:HG13	8	0.23	0.08	0.2
(1,2135)	1:A:85:ASP:HA	1:A:86:THR:HG21	8	0.22	0.04	0.2
(1,2135)	1:A:85:ASP:HA	1:A:86:THR:HG22	8	0.22	0.04	0.2
(1,2135)	1:A:85:ASP:HA	1:A:86:THR:HG23	8	0.22	0.04	0.2
(1,1155)	1:A:39:PHE:HD1	1:A:95:LEU:HD21	8	0.2	0.04	0.18
(1,1155)	1:A:39:PHE:HD1	1:A:95:LEU:HD22	8	0.2	0.04	0.18
(1,1155)	1:A:39:PHE:HD1	1:A:95:LEU:HD23	8	0.2	0.04	0.18
(1,1155)	1:A:39:PHE:HD2	1:A:95:LEU:HD21	8	0.2	0.04	0.18
(1,1155)	1:A:39:PHE:HD2	1:A:95:LEU:HD22	8	0.2	0.04	0.18
(1,1155)	1:A:39:PHE:HD2	1:A:95:LEU:HD23	8	0.2	0.04	0.18
(1,688)	1:A:22:ILE:H	1:A:50:MET:HA	8	0.17	0.06	0.16
(1,2396)	1:A:98:LEU:H	1:A:98:LEU:HG	8	0.15	0.02	0.15
(1,2407)	1:A:99:ILE:HA	1:A:104:ALA:H	8	0.14	0.02	0.14
(1,612)	1:A:21:VAL:HB	1:A:78:TYR:HB2	8	0.14	0.02	0.14
(2,155)	1:A:27:ALA:H	1:A:29:TRP:H	8	0.13	0.02	0.12
(2,90)	1:A:14:ILE:H	1:A:17:GLN:H	8	0.12	0.01	0.12
(1,915)	1:A:26:PHE:H	1:A:27:ALA:H	8	0.12	0.01	0.11
(2,341)	1:A:72:MET:HA	1:A:72:MET:HE1	7	0.59	0.11	0.64
(2,341)	1:A:72:MET:HA	1:A:72:MET:HE2	7	0.59	0.11	0.64
(2,341)	1:A:72:MET:HA	1:A:72:MET:HE3	7	0.59	0.11	0.64
(1,2238)	1:A:91:ASN:HB2	1:A:94:ALA:HB1	7	0.41	0.01	0.41
(1,2238)	1:A:91:ASN:HB2	1:A:94:ALA:HB2	7	0.41	0.01	0.41
(1,2238)	1:A:91:ASN:HB2	1:A:94:ALA:HB3	7	0.41	0.01	0.41
(2,98)	1:A:20:LEU:HA	1:A:79:LYS:HE2	7	0.37	0.15	0.36
(2,98)	1:A:20:LEU:HA	1:A:79:LYS:HE3	7	0.37	0.15	0.36
(2,99)	1:A:20:LEU:HA	1:A:79:LYS:HE2	7	0.37	0.15	0.36
(2,99)	1:A:20:LEU:HA	1:A:79:LYS:HE3	7	0.37	0.15	0.36
(2,372)	1:A:77:VAL:HA	1:A:78:TYR:HE1	7	0.36	0.13	0.39
(2,372)	1:A:77:VAL:HA	1:A:78:TYR:HE2	7	0.36	0.13	0.39
(1,731)	1:A:23:VAL:HG11	1:A:78:TYR:HE1	7	0.32	0.05	0.31
(1,731)	1:A:23:VAL:HG11	1:A:78:TYR:HE2	7	0.32	0.05	0.31
(1,731)	1:A:23:VAL:HG12	1:A:78:TYR:HE1	7	0.32	0.05	0.31
(1,731)	1:A:23:VAL:HG12	1:A:78:TYR:HE2	7	0.32	0.05	0.31
(1,731)	1:A:23:VAL:HG13	1:A:78:TYR:HE1	7	0.32	0.05	0.31
(1,731)	1:A:23:VAL:HG13	1:A:78:TYR:HE2	7	0.32	0.05	0.31
(1,1358)	1:A:47:TYR:HB3	1:A:99:ILE:HD11	7	0.31	0.11	0.31
(1,1358)	1:A:47:TYR:HB3	1:A:99:ILE:HD12	7	0.31	0.11	0.31
(1,1358)	1:A:47:TYR:HB3	1:A:99:ILE:HD13	7	0.31	0.11	0.31
(1,2197)	1:A:88:LEU:HD11	1:A:89:GLY:H	7	0.3	0.08	0.28
(1,2197)	1:A:88:LEU:HD12	1:A:89:GLY:H	7	0.3	0.08	0.28

Continued on next page...

Continued from previous page...

Key	Atom-1	Atom-2	Models[1]	Mean (Å)	SD[1] (Å)	Median (Å)
(1,2197)	1:A:88:LEU:HD13	1:A:89:GLY:H	7	0.3	0.08	0.28
(1,2197)	1:A:88:LEU:HD21	1:A:89:GLY:H	7	0.3	0.08	0.28
(1,2197)	1:A:88:LEU:HD22	1:A:89:GLY:H	7	0.3	0.08	0.28
(1,2197)	1:A:88:LEU:HD23	1:A:89:GLY:H	7	0.3	0.08	0.28
(1,503)	1:A:18:ASN:HD21	1:A:21:VAL:HG21	7	0.28	0.09	0.3
(1,503)	1:A:18:ASN:HD21	1:A:21:VAL:HG22	7	0.28	0.09	0.3
(1,503)	1:A:18:ASN:HD21	1:A:21:VAL:HG23	7	0.28	0.09	0.3
(1,587)	1:A:20:LEU:HG	1:A:77:VAL:HG11	7	0.27	0.11	0.21
(1,587)	1:A:20:LEU:HG	1:A:77:VAL:HG12	7	0.27	0.11	0.21
(1,587)	1:A:20:LEU:HG	1:A:77:VAL:HG13	7	0.27	0.11	0.21
(2,284)	1:A:57:VAL:HG11	1:A:61:SER:HA	7	0.24	0.05	0.26
(2,284)	1:A:57:VAL:HG12	1:A:61:SER:HA	7	0.24	0.05	0.26
(2,284)	1:A:57:VAL:HG13	1:A:61:SER:HA	7	0.24	0.05	0.26
(1,381)	1:A:15:ILE:HD11	1:A:23:VAL:HG11	7	0.15	0.03	0.15
(1,381)	1:A:15:ILE:HD11	1:A:23:VAL:HG12	7	0.15	0.03	0.15
(1,381)	1:A:15:ILE:HD11	1:A:23:VAL:HG13	7	0.15	0.03	0.15
(1,381)	1:A:15:ILE:HD12	1:A:23:VAL:HG11	7	0.15	0.03	0.15
(1,381)	1:A:15:ILE:HD12	1:A:23:VAL:HG12	7	0.15	0.03	0.15
(1,381)	1:A:15:ILE:HD12	1:A:23:VAL:HG13	7	0.15	0.03	0.15
(1,381)	1:A:15:ILE:HD13	1:A:23:VAL:HG11	7	0.15	0.03	0.15
(1,381)	1:A:15:ILE:HD13	1:A:23:VAL:HG12	7	0.15	0.03	0.15
(1,381)	1:A:15:ILE:HD13	1:A:23:VAL:HG13	7	0.15	0.03	0.15
(1,943)	1:A:28:GLU:H	1:A:29:TRP:HD1	7	0.14	0.01	0.14
(1,1259)	1:A:43:CYS:HA	1:A:52:PHE:HZ	7	0.12	0.02	0.12
(2,131)	1:A:26:PHE:HA	1:A:72:MET:HE1	6	0.67	0.26	0.84
(2,131)	1:A:26:PHE:HA	1:A:72:MET:HE2	6	0.67	0.26	0.84
(2,131)	1:A:26:PHE:HA	1:A:72:MET:HE3	6	0.67	0.26	0.84
(1,607)	1:A:21:VAL:HA	1:A:51:VAL:HG21	6	0.53	0.01	0.53
(1,607)	1:A:21:VAL:HA	1:A:51:VAL:HG22	6	0.53	0.01	0.53
(1,607)	1:A:21:VAL:HA	1:A:51:VAL:HG23	6	0.53	0.01	0.53
(2,80)	1:A:12:ASP:HB3	1:A:66:LYS:HE2	6	0.51	0.08	0.53
(2,80)	1:A:12:ASP:HB3	1:A:66:LYS:HE3	6	0.51	0.08	0.53
(1,1981)	1:A:77:VAL:HG21	1:A:102:TYR:HE1	6	0.49	0.22	0.52
(1,1981)	1:A:77:VAL:HG21	1:A:102:TYR:HE2	6	0.49	0.22	0.52
(1,1981)	1:A:77:VAL:HG22	1:A:102:TYR:HE1	6	0.49	0.22	0.52
(1,1981)	1:A:77:VAL:HG22	1:A:102:TYR:HE2	6	0.49	0.22	0.52
(1,1981)	1:A:77:VAL:HG23	1:A:102:TYR:HE1	6	0.49	0.22	0.52
(1,1981)	1:A:77:VAL:HG23	1:A:102:TYR:HE2	6	0.49	0.22	0.52
(2,183)	1:A:29:TRP:HE1	1:A:72:MET:HE1	6	0.44	0.21	0.43
(2,183)	1:A:29:TRP:HE1	1:A:72:MET:HE2	6	0.44	0.21	0.43
(2,183)	1:A:29:TRP:HE1	1:A:72:MET:HE3	6	0.44	0.21	0.43
(2,83)	1:A:12:ASP:HB2	1:A:66:LYS:HE2	6	0.39	0.09	0.4

Continued on next page...

Continued from previous page...

Key	Atom-1	Atom-2	Models[1]	Mean (Å)	SD[1] (Å)	Median (Å)
(2,83)	1:A:12:ASP:HB2	1:A:66:LYS:HE3	6	0.39	0.09	0.4
(1,1458)	1:A:51:VAL:H	1:A:51:VAL:HG21	6	0.38	0.01	0.38
(1,1458)	1:A:51:VAL:H	1:A:51:VAL:HG22	6	0.38	0.01	0.38
(1,1458)	1:A:51:VAL:H	1:A:51:VAL:HG23	6	0.38	0.01	0.38
(2,209)	1:A:40:TYR:HD1	1:A:43:CYS:H	6	0.34	0.12	0.36
(2,209)	1:A:40:TYR:HD2	1:A:43:CYS:H	6	0.34	0.12	0.36
(1,1334)	1:A:46:THR:HG21	1:A:47:TYR:HD1	6	0.32	0.1	0.32
(1,1334)	1:A:46:THR:HG21	1:A:47:TYR:HD2	6	0.32	0.1	0.32
(1,1334)	1:A:46:THR:HG22	1:A:47:TYR:HD1	6	0.32	0.1	0.32
(1,1334)	1:A:46:THR:HG22	1:A:47:TYR:HD2	6	0.32	0.1	0.32
(1,1334)	1:A:46:THR:HG23	1:A:47:TYR:HD1	6	0.32	0.1	0.32
(1,1334)	1:A:46:THR:HG23	1:A:47:TYR:HD2	6	0.32	0.1	0.32
(1,1890)	1:A:75:PHE:HZ	1:A:90:ALA:HB1	6	0.32	0.11	0.33
(1,1890)	1:A:75:PHE:HZ	1:A:90:ALA:HB2	6	0.32	0.11	0.33
(1,1890)	1:A:75:PHE:HZ	1:A:90:ALA:HB3	6	0.32	0.11	0.33
(1,2200)	1:A:88:LEU:HD21	1:A:88:LEU:HA	6	0.25	0.08	0.25
(1,2200)	1:A:88:LEU:HD22	1:A:88:LEU:HA	6	0.25	0.08	0.25
(1,2200)	1:A:88:LEU:HD23	1:A:88:LEU:HA	6	0.25	0.08	0.25
(1,375)	1:A:15:ILE:HD11	1:A:21:VAL:HG11	6	0.21	0.07	0.19
(1,375)	1:A:15:ILE:HD11	1:A:21:VAL:HG12	6	0.21	0.07	0.19
(1,375)	1:A:15:ILE:HD11	1:A:21:VAL:HG13	6	0.21	0.07	0.19
(1,375)	1:A:15:ILE:HD12	1:A:21:VAL:HG11	6	0.21	0.07	0.19
(1,375)	1:A:15:ILE:HD12	1:A:21:VAL:HG12	6	0.21	0.07	0.19
(1,375)	1:A:15:ILE:HD12	1:A:21:VAL:HG13	6	0.21	0.07	0.19
(1,375)	1:A:15:ILE:HD13	1:A:21:VAL:HG11	6	0.21	0.07	0.19
(1,375)	1:A:15:ILE:HD13	1:A:21:VAL:HG12	6	0.21	0.07	0.19
(1,375)	1:A:15:ILE:HD13	1:A:21:VAL:HG13	6	0.21	0.07	0.19
(2,243)	1:A:47:TYR:HA	1:A:50:MET:HB2	6	0.14	0.02	0.15
(1,1090)	1:A:36:ILE:HG21	1:A:90:ALA:HB1	6	0.14	0.04	0.14
(1,1090)	1:A:36:ILE:HG21	1:A:90:ALA:HB2	6	0.14	0.04	0.14
(1,1090)	1:A:36:ILE:HG21	1:A:90:ALA:HB3	6	0.14	0.04	0.14
(1,1090)	1:A:36:ILE:HG22	1:A:90:ALA:HB1	6	0.14	0.04	0.14
(1,1090)	1:A:36:ILE:HG22	1:A:90:ALA:HB2	6	0.14	0.04	0.14
(1,1090)	1:A:36:ILE:HG22	1:A:90:ALA:HB3	6	0.14	0.04	0.14
(1,1090)	1:A:36:ILE:HG23	1:A:90:ALA:HB1	6	0.14	0.04	0.14
(1,1090)	1:A:36:ILE:HG23	1:A:90:ALA:HB2	6	0.14	0.04	0.14
(1,1090)	1:A:36:ILE:HG23	1:A:90:ALA:HB3	6	0.14	0.04	0.14
(1,47)	1:A:3:LYS:HG2	1:A:53:ILE:HA	6	0.12	0.01	0.12
(2,157)	1:A:27:ALA:H	1:A:30:CYS:H	6	0.12	0.01	0.12
(2,329)	1:A:69:ILE:HG21	1:A:72:MET:HE1	5	1.25	0.48	1.51
(2,329)	1:A:69:ILE:HG21	1:A:72:MET:HE2	5	1.25	0.48	1.51
(2,329)	1:A:69:ILE:HG21	1:A:72:MET:HE3	5	1.25	0.48	1.51

Continued on next page...

Continued from previous page...

Key	Atom-1	Atom-2	Models[1]	Mean (Å)	SD[1] (Å)	Median (Å)
(2,329)	1:A:69:ILE:HG22	1:A:72:MET:HE1	5	1.25	0.48	1.51
(2,329)	1:A:69:ILE:HG22	1:A:72:MET:HE2	5	1.25	0.48	1.51
(2,329)	1:A:69:ILE:HG22	1:A:72:MET:HE3	5	1.25	0.48	1.51
(2,329)	1:A:69:ILE:HG23	1:A:72:MET:HE1	5	1.25	0.48	1.51
(2,329)	1:A:69:ILE:HG23	1:A:72:MET:HE2	5	1.25	0.48	1.51
(2,329)	1:A:69:ILE:HG23	1:A:72:MET:HE3	5	1.25	0.48	1.51
(2,215)	1:A:42:GLU:H	1:A:45:LYS:HE2	5	0.51	0.08	0.55
(2,215)	1:A:42:GLU:H	1:A:45:LYS:HE3	5	0.51	0.08	0.55
(2,264)	1:A:50:MET:HE1	1:A:99:ILE:HA	5	0.42	0.26	0.24
(2,264)	1:A:50:MET:HE2	1:A:99:ILE:HA	5	0.42	0.26	0.24
(2,264)	1:A:50:MET:HE3	1:A:99:ILE:HA	5	0.42	0.26	0.24
(1,1896)	1:A:76:LYS:HA	1:A:76:LYS:HG2	5	0.31	0.01	0.31
(1,1896)	1:A:76:LYS:HA	1:A:76:LYS:HG3	5	0.31	0.01	0.31
(2,244)	1:A:47:TYR:HA	1:A:52:PHE:HE1	5	0.3	0.12	0.25
(2,244)	1:A:47:TYR:HA	1:A:52:PHE:HE2	5	0.3	0.12	0.25
(2,230)	1:A:44:SER:HG	1:A:45:LYS:HE2	5	0.3	0.2	0.15
(2,230)	1:A:44:SER:HG	1:A:45:LYS:HE3	5	0.3	0.2	0.15
(1,144)	1:A:8:GLN:HE21	1:A:11:PHE:HD1	5	0.27	0.04	0.3
(1,144)	1:A:8:GLN:HE21	1:A:11:PHE:HD2	5	0.27	0.04	0.3
(1,826)	1:A:25:PHE:HD1	1:A:25:PHE:H	5	0.25	0.01	0.26
(1,826)	1:A:25:PHE:HD2	1:A:25:PHE:H	5	0.25	0.01	0.26
(1,425)	1:A:15:ILE:HG21	1:A:78:TYR:HB2	5	0.23	0.05	0.23
(1,425)	1:A:15:ILE:HG22	1:A:78:TYR:HB2	5	0.23	0.05	0.23
(1,425)	1:A:15:ILE:HG23	1:A:78:TYR:HB2	5	0.23	0.05	0.23
(1,1550)	1:A:57:VAL:HG21	1:A:58:ASP:H	5	0.18	0.04	0.2
(1,1550)	1:A:57:VAL:HG22	1:A:58:ASP:H	5	0.18	0.04	0.2
(1,1550)	1:A:57:VAL:HG23	1:A:58:ASP:H	5	0.18	0.04	0.2
(1,2535)	1:A:103:ALA:H	1:A:104:ALA:HA	5	0.17	0.02	0.16
(1,153)	1:A:8:GLN:HE22	1:A:9:SER:H	5	0.15	0.02	0.17
(1,971)	1:A:30:CYS:H	1:A:32:PRO:HG3	5	0.15	0.02	0.15
(1,1486)	1:A:52:PHE:HZ	1:A:99:ILE:HA	5	0.15	0.03	0.15
(1,1505)	1:A:54:LYS:HA	1:A:54:LYS:HG3	5	0.14	0.02	0.15
(1,460)	1:A:17:GLN:HA	1:A:17:GLN:HG2	5	0.13	0.03	0.11
(1,1739)	1:A:69:ILE:HD11	1:A:69:ILE:HG13	5	0.13	0.0	0.13
(1,1739)	1:A:69:ILE:HD12	1:A:69:ILE:HG13	5	0.13	0.0	0.13
(1,1739)	1:A:69:ILE:HD13	1:A:69:ILE:HG13	5	0.13	0.0	0.13
(1,1740)	1:A:69:ILE:HD11	1:A:69:ILE:HG12	5	0.13	0.0	0.13
(1,1740)	1:A:69:ILE:HD12	1:A:69:ILE:HG12	5	0.13	0.0	0.13
(1,1740)	1:A:69:ILE:HD13	1:A:69:ILE:HG12	5	0.13	0.0	0.13
(1,1074)	1:A:36:ILE:HG12	1:A:75:PHE:HE1	5	0.12	0.02	0.11
(1,1074)	1:A:36:ILE:HG12	1:A:75:PHE:HE2	5	0.12	0.02	0.11
(1,1433)	1:A:50:MET:HG3	1:A:103:ALA:H	5	0.12	0.01	0.12

Continued on next page...

Continued from previous page...

Key	Atom-1	Atom-2	Models[1]	Mean (Å)	SD[1] (Å)	Median (Å)
(1,1133)	1:A:39:PHE:HB2	1:A:41:GLU:H	5	0.12	0.01	0.11
(2,193)	1:A:29:TRP:HZ3	1:A:72:MET:HE1	4	0.63	0.16	0.62
(2,193)	1:A:29:TRP:HZ3	1:A:72:MET:HE2	4	0.63	0.16	0.62
(2,193)	1:A:29:TRP:HZ3	1:A:72:MET:HE3	4	0.63	0.16	0.62
(1,606)	1:A:21:VAL:HA	1:A:51:VAL:HG11	4	0.57	0.01	0.57
(1,606)	1:A:21:VAL:HA	1:A:51:VAL:HG12	4	0.57	0.01	0.57
(1,606)	1:A:21:VAL:HA	1:A:51:VAL:HG13	4	0.57	0.01	0.57
(1,1980)	1:A:77:VAL:HG21	1:A:102:TYR:HD1	4	0.44	0.11	0.46
(1,1980)	1:A:77:VAL:HG21	1:A:102:TYR:HD2	4	0.44	0.11	0.46
(1,1980)	1:A:77:VAL:HG22	1:A:102:TYR:HD1	4	0.44	0.11	0.46
(1,1980)	1:A:77:VAL:HG22	1:A:102:TYR:HD2	4	0.44	0.11	0.46
(1,1980)	1:A:77:VAL:HG23	1:A:102:TYR:HD1	4	0.44	0.11	0.46
(1,1980)	1:A:77:VAL:HG23	1:A:102:TYR:HD2	4	0.44	0.11	0.46
(1,692)	1:A:22:ILE:H	1:A:51:VAL:HG11	4	0.4	0.04	0.4
(1,692)	1:A:22:ILE:H	1:A:51:VAL:HG12	4	0.4	0.04	0.4
(1,692)	1:A:22:ILE:H	1:A:51:VAL:HG13	4	0.4	0.04	0.4
(2,34)	1:A:3:LYS:HE2	1:A:5:VAL:HB	4	0.33	0.14	0.3
(2,34)	1:A:3:LYS:HE3	1:A:5:VAL:HB	4	0.33	0.14	0.3
(1,2018)	1:A:78:TYR:HD1	1:A:83:SER:HA	4	0.33	0.12	0.4
(1,2018)	1:A:78:TYR:HD2	1:A:83:SER:HA	4	0.33	0.12	0.4
(1,736)	1:A:23:VAL:HG21	1:A:24:ASP:H	4	0.32	0.08	0.34
(1,736)	1:A:23:VAL:HG22	1:A:24:ASP:H	4	0.32	0.08	0.34
(1,736)	1:A:23:VAL:HG23	1:A:24:ASP:H	4	0.32	0.08	0.34
(2,151)	1:A:27:ALA:HB1	1:A:58:ASP:HB2	4	0.3	0.07	0.3
(2,151)	1:A:27:ALA:HB2	1:A:58:ASP:HB2	4	0.3	0.07	0.3
(2,151)	1:A:27:ALA:HB3	1:A:58:ASP:HB2	4	0.3	0.07	0.3
(1,752)	1:A:23:VAL:HG21	1:A:55:VAL:HG21	4	0.28	0.02	0.26
(1,752)	1:A:23:VAL:HG21	1:A:55:VAL:HG22	4	0.28	0.02	0.26
(1,752)	1:A:23:VAL:HG21	1:A:55:VAL:HG23	4	0.28	0.02	0.26
(1,752)	1:A:23:VAL:HG22	1:A:55:VAL:HG21	4	0.28	0.02	0.26
(1,752)	1:A:23:VAL:HG22	1:A:55:VAL:HG22	4	0.28	0.02	0.26
(1,752)	1:A:23:VAL:HG22	1:A:55:VAL:HG23	4	0.28	0.02	0.26
(1,752)	1:A:23:VAL:HG23	1:A:55:VAL:HG21	4	0.28	0.02	0.26
(1,752)	1:A:23:VAL:HG23	1:A:55:VAL:HG22	4	0.28	0.02	0.26
(1,752)	1:A:23:VAL:HG23	1:A:55:VAL:HG23	4	0.28	0.02	0.26
(1,12)	1:A:2:VAL:HG11	1:A:3:LYS:H	4	0.25	0.07	0.27
(1,12)	1:A:2:VAL:HG12	1:A:3:LYS:H	4	0.25	0.07	0.27
(1,12)	1:A:2:VAL:HG13	1:A:3:LYS:H	4	0.25	0.07	0.27
(1,2388)	1:A:98:LEU:HD21	1:A:99:ILE:HA	4	0.24	0.06	0.24
(1,2388)	1:A:98:LEU:HD22	1:A:99:ILE:HA	4	0.24	0.06	0.24
(1,2388)	1:A:98:LEU:HD23	1:A:99:ILE:HA	4	0.24	0.06	0.24
(1,1915)	1:A:76:LYS:HD2	1:A:86:THR:HA	4	0.23	0.09	0.23

Continued on next page...

Continued from previous page...

Key	Atom-1	Atom-2	Models[1]	Mean (Å)	SD[1] (Å)	Median (Å)
(1,1915)	1:A:76:LYS:HD3	1:A:86:THR:HA	4	0.23	0.09	0.23
(1,665)	1:A:22:ILE:HG21	1:A:50:MET:HB3	4	0.23	0.07	0.22
(1,665)	1:A:22:ILE:HG22	1:A:50:MET:HB3	4	0.23	0.07	0.22
(1,665)	1:A:22:ILE:HG23	1:A:50:MET:HB3	4	0.23	0.07	0.22
(2,268)	1:A:51:VAL:H	1:A:52:PHE:HE1	4	0.17	0.04	0.18
(2,268)	1:A:51:VAL:H	1:A:52:PHE:HE2	4	0.17	0.04	0.18
(1,788)	1:A:24:ASP:HB2	1:A:75:PHE:HE1	4	0.16	0.02	0.17
(1,788)	1:A:24:ASP:HB2	1:A:75:PHE:HE2	4	0.16	0.02	0.17
(1,2468)	1:A:101:LYS:HB2	1:A:103:ALA:H	4	0.15	0.03	0.16
(1,932)	1:A:27:ALA:HB1	1:A:57:VAL:HG21	4	0.14	0.03	0.14
(1,932)	1:A:27:ALA:HB1	1:A:57:VAL:HG22	4	0.14	0.03	0.14
(1,932)	1:A:27:ALA:HB1	1:A:57:VAL:HG23	4	0.14	0.03	0.14
(1,932)	1:A:27:ALA:HB2	1:A:57:VAL:HG21	4	0.14	0.03	0.14
(1,932)	1:A:27:ALA:HB2	1:A:57:VAL:HG22	4	0.14	0.03	0.14
(1,932)	1:A:27:ALA:HB2	1:A:57:VAL:HG23	4	0.14	0.03	0.14
(1,932)	1:A:27:ALA:HB3	1:A:57:VAL:HG21	4	0.14	0.03	0.14
(1,932)	1:A:27:ALA:HB3	1:A:57:VAL:HG22	4	0.14	0.03	0.14
(1,932)	1:A:27:ALA:HB3	1:A:57:VAL:HG23	4	0.14	0.03	0.14
(1,2506)	1:A:101:LYS:H	1:A:103:ALA:H	4	0.14	0.03	0.14
(1,2289)	1:A:94:ALA:HA	1:A:98:LEU:H	4	0.12	0.01	0.12
(1,1097)	1:A:36:ILE:H	1:A:36:ILE:HG21	4	0.12	0.01	0.12
(1,1097)	1:A:36:ILE:H	1:A:36:ILE:HG22	4	0.12	0.01	0.12
(1,1097)	1:A:36:ILE:H	1:A:36:ILE:HG23	4	0.12	0.01	0.12
(1,2156)	1:A:86:THR:H	1:A:86:THR:HB	4	0.12	0.01	0.11
(1,439)	1:A:15:ILE:H	1:A:15:ILE:HG12	4	0.12	0.01	0.11
(1,389)	1:A:15:ILE:HD11	1:A:78:TYR:HD1	3	0.61	0.03	0.63
(1,389)	1:A:15:ILE:HD11	1:A:78:TYR:HD2	3	0.61	0.03	0.63
(1,389)	1:A:15:ILE:HD12	1:A:78:TYR:HD1	3	0.61	0.03	0.63
(1,389)	1:A:15:ILE:HD12	1:A:78:TYR:HD2	3	0.61	0.03	0.63
(1,389)	1:A:15:ILE:HD13	1:A:78:TYR:HD1	3	0.61	0.03	0.63
(1,389)	1:A:15:ILE:HD13	1:A:78:TYR:HD2	3	0.61	0.03	0.63
(1,390)	1:A:15:ILE:HD11	1:A:78:TYR:HE1	3	0.6	0.12	0.66
(1,390)	1:A:15:ILE:HD11	1:A:78:TYR:HE2	3	0.6	0.12	0.66
(1,390)	1:A:15:ILE:HD12	1:A:78:TYR:HE1	3	0.6	0.12	0.66
(1,390)	1:A:15:ILE:HD12	1:A:78:TYR:HE2	3	0.6	0.12	0.66
(1,390)	1:A:15:ILE:HD13	1:A:78:TYR:HE1	3	0.6	0.12	0.66
(1,390)	1:A:15:ILE:HD13	1:A:78:TYR:HE2	3	0.6	0.12	0.66
(1,1719)	1:A:68:ASN:HD21	1:A:70:THR:HG21	3	0.53	0.08	0.57
(1,1719)	1:A:68:ASN:HD21	1:A:70:THR:HG22	3	0.53	0.08	0.57
(1,1719)	1:A:68:ASN:HD21	1:A:70:THR:HG23	3	0.53	0.08	0.57
(1,1960)	1:A:77:VAL:HG11	1:A:102:TYR:HD1	3	0.47	0.02	0.46
(1,1960)	1:A:77:VAL:HG11	1:A:102:TYR:HD2	3	0.47	0.02	0.46

Continued on next page...

Continued from previous page...

Key	Atom-1	Atom-2	Models[1]	Mean (Å)	SD[1] (Å)	Median (Å)
(1,1960)	1:A:77:VAL:HG12	1:A:102:TYR:HD1	3	0.47	0.02	0.46
(1,1960)	1:A:77:VAL:HG12	1:A:102:TYR:HD2	3	0.47	0.02	0.46
(1,1960)	1:A:77:VAL:HG13	1:A:102:TYR:HD1	3	0.47	0.02	0.46
(1,1960)	1:A:77:VAL:HG13	1:A:102:TYR:HD2	3	0.47	0.02	0.46
(1,747)	1:A:23:VAL:HG21	1:A:53:ILE:HG12	3	0.45	0.02	0.45
(1,747)	1:A:23:VAL:HG22	1:A:53:ILE:HG12	3	0.45	0.02	0.45
(1,747)	1:A:23:VAL:HG23	1:A:53:ILE:HG12	3	0.45	0.02	0.45
(1,2429)	1:A:99:ILE:H	1:A:99:ILE:HG21	3	0.44	0.0	0.44
(1,2429)	1:A:99:ILE:H	1:A:99:ILE:HG22	3	0.44	0.0	0.44
(1,2429)	1:A:99:ILE:H	1:A:99:ILE:HG23	3	0.44	0.0	0.44
(1,2127)	1:A:84:VAL:HG21	1:A:102:TYR:HE1	3	0.39	0.23	0.37
(1,2127)	1:A:84:VAL:HG21	1:A:102:TYR:HE2	3	0.39	0.23	0.37
(1,2127)	1:A:84:VAL:HG22	1:A:102:TYR:HE1	3	0.39	0.23	0.37
(1,2127)	1:A:84:VAL:HG22	1:A:102:TYR:HE2	3	0.39	0.23	0.37
(1,2127)	1:A:84:VAL:HG23	1:A:102:TYR:HE1	3	0.39	0.23	0.37
(1,2127)	1:A:84:VAL:HG23	1:A:102:TYR:HE2	3	0.39	0.23	0.37
(1,2244)	1:A:91:ASN:HD21	1:A:94:ALA:HB1	3	0.35	0.07	0.33
(1,2244)	1:A:91:ASN:HD21	1:A:94:ALA:HB2	3	0.35	0.07	0.33
(1,2244)	1:A:91:ASN:HD21	1:A:94:ALA:HB3	3	0.35	0.07	0.33
(1,2172)	1:A:87:LEU:HD11	1:A:91:ASN:HB2	3	0.33	0.11	0.34
(1,2172)	1:A:87:LEU:HD12	1:A:91:ASN:HB2	3	0.33	0.11	0.34
(1,2172)	1:A:87:LEU:HD13	1:A:91:ASN:HB2	3	0.33	0.11	0.34
(1,1778)	1:A:70:THR:H	1:A:70:THR:HG21	3	0.31	0.0	0.31
(1,1778)	1:A:70:THR:H	1:A:70:THR:HG22	3	0.31	0.0	0.31
(1,1778)	1:A:70:THR:H	1:A:70:THR:HG23	3	0.31	0.0	0.31
(1,1376)	1:A:47:TYR:HD1	1:A:99:ILE:HG12	3	0.29	0.0	0.29
(1,1376)	1:A:47:TYR:HD2	1:A:99:ILE:HG12	3	0.29	0.0	0.29
(1,304)	1:A:14:ILE:HA	1:A:14:ILE:HD11	3	0.25	0.0	0.25
(1,304)	1:A:14:ILE:HA	1:A:14:ILE:HD12	3	0.25	0.0	0.25
(1,304)	1:A:14:ILE:HA	1:A:14:ILE:HD13	3	0.25	0.0	0.25
(1,362)	1:A:15:ILE:HD11	1:A:15:ILE:HA	3	0.23	0.03	0.24
(1,362)	1:A:15:ILE:HD12	1:A:15:ILE:HA	3	0.23	0.03	0.24
(1,362)	1:A:15:ILE:HD13	1:A:15:ILE:HA	3	0.23	0.03	0.24
(1,1336)	1:A:46:THR:HG21	1:A:47:TYR:H	3	0.22	0.04	0.2
(1,1336)	1:A:46:THR:HG22	1:A:47:TYR:H	3	0.22	0.04	0.2
(1,1336)	1:A:46:THR:HG23	1:A:47:TYR:H	3	0.22	0.04	0.2
(1,2409)	1:A:99:ILE:HD11	1:A:99:ILE:HA	3	0.22	0.0	0.22
(1,2409)	1:A:99:ILE:HD12	1:A:99:ILE:HA	3	0.22	0.0	0.22
(1,2409)	1:A:99:ILE:HD13	1:A:99:ILE:HA	3	0.22	0.0	0.22
(1,838)	1:A:25:PHE:HE1	1:A:63:VAL:HG11	3	0.2	0.06	0.21
(1,838)	1:A:25:PHE:HE1	1:A:63:VAL:HG12	3	0.2	0.06	0.21
(1,838)	1:A:25:PHE:HE1	1:A:63:VAL:HG13	3	0.2	0.06	0.21

Continued on next page...

Continued from previous page...

Key	Atom-1	Atom-2	Models[1]	Mean (Å)	SD[1] (Å)	Median (Å)
(1,838)	1:A:25:PHE:HE2	1:A:63:VAL:HG11	3	0.2	0.06	0.21
(1,838)	1:A:25:PHE:HE2	1:A:63:VAL:HG12	3	0.2	0.06	0.21
(1,838)	1:A:25:PHE:HE2	1:A:63:VAL:HG13	3	0.2	0.06	0.21
(1,168)	1:A:8:GLN:H	1:A:60:VAL:HG11	3	0.2	0.07	0.15
(1,168)	1:A:8:GLN:H	1:A:60:VAL:HG12	3	0.2	0.07	0.15
(1,168)	1:A:8:GLN:H	1:A:60:VAL:HG13	3	0.2	0.07	0.15
(1,746)	1:A:23:VAL:HG21	1:A:53:ILE:HG13	3	0.2	0.03	0.18
(1,746)	1:A:23:VAL:HG22	1:A:53:ILE:HG13	3	0.2	0.03	0.18
(1,746)	1:A:23:VAL:HG23	1:A:53:ILE:HG13	3	0.2	0.03	0.18
(1,1331)	1:A:46:THR:HG21	1:A:47:TYR:HA	3	0.19	0.07	0.15
(1,1331)	1:A:46:THR:HG22	1:A:47:TYR:HA	3	0.19	0.07	0.15
(1,1331)	1:A:46:THR:HG23	1:A:47:TYR:HA	3	0.19	0.07	0.15
(1,232)	1:A:11:PHE:HE1	1:A:63:VAL:HG11	3	0.16	0.02	0.17
(1,232)	1:A:11:PHE:HE1	1:A:63:VAL:HG12	3	0.16	0.02	0.17
(1,232)	1:A:11:PHE:HE1	1:A:63:VAL:HG13	3	0.16	0.02	0.17
(1,232)	1:A:11:PHE:HE2	1:A:63:VAL:HG11	3	0.16	0.02	0.17
(1,232)	1:A:11:PHE:HE2	1:A:63:VAL:HG12	3	0.16	0.02	0.17
(1,232)	1:A:11:PHE:HE2	1:A:63:VAL:HG13	3	0.16	0.02	0.17
(1,1488)	1:A:52:PHE:HZ	1:A:99:ILE:HG12	3	0.16	0.04	0.14
(1,105)	1:A:5:VAL:H	1:A:55:VAL:HB	3	0.15	0.02	0.15
(1,1988)	1:A:77:VAL:H	1:A:84:VAL:HB	3	0.14	0.02	0.14
(1,586)	1:A:20:LEU:HG	1:A:51:VAL:H	3	0.14	0.02	0.15
(1,1607)	1:A:62:GLU:HB3	1:A:63:VAL:H	3	0.14	0.0	0.14
(1,937)	1:A:28:GLU:HB3	1:A:29:TRP:HD1	3	0.13	0.01	0.12
(2,89)	1:A:14:ILE:H	1:A:17:GLN:HB2	3	0.12	0.0	0.12
(1,164)	1:A:8:GLN:H	1:A:8:GLN:HG2	3	0.11	0.0	0.11
(2,137)	1:A:26:PHE:HB2	1:A:72:MET:HE1	2	0.72	0.1	0.72
(2,137)	1:A:26:PHE:HB2	1:A:72:MET:HE2	2	0.72	0.1	0.72
(2,137)	1:A:26:PHE:HB2	1:A:72:MET:HE3	2	0.72	0.1	0.72
(2,10)	1:A:1:SER:HB2	1:A:3:LYS:H	2	0.6	0.02	0.6
(2,10)	1:A:1:SER:HB3	1:A:3:LYS:H	2	0.6	0.02	0.6
(1,426)	1:A:15:ILE:HG21	1:A:78:TYR:HD1	2	0.52	0.0	0.52
(1,426)	1:A:15:ILE:HG21	1:A:78:TYR:HD2	2	0.52	0.0	0.52
(1,426)	1:A:15:ILE:HG22	1:A:78:TYR:HD1	2	0.52	0.0	0.52
(1,426)	1:A:15:ILE:HG22	1:A:78:TYR:HD2	2	0.52	0.0	0.52
(1,426)	1:A:15:ILE:HG23	1:A:78:TYR:HD1	2	0.52	0.0	0.52
(1,426)	1:A:15:ILE:HG23	1:A:78:TYR:HD2	2	0.52	0.0	0.52
(1,2006)	1:A:78:TYR:HD1	1:A:15:ILE:HG21	2	0.52	0.0	0.52
(1,2006)	1:A:78:TYR:HD1	1:A:15:ILE:HG22	2	0.52	0.0	0.52
(1,2006)	1:A:78:TYR:HD1	1:A:15:ILE:HG23	2	0.52	0.0	0.52
(1,2006)	1:A:78:TYR:HD2	1:A:15:ILE:HG21	2	0.52	0.0	0.52
(1,2006)	1:A:78:TYR:HD2	1:A:15:ILE:HG22	2	0.52	0.0	0.52

Continued on next page...

Continued from previous page...

Key	Atom-1	Atom-2	Models[1]	Mean (Å)	SD[1] (Å)	Median (Å)
(1,2006)	1:A:78:TYR:HD2	1:A:15:ILE:HG23	2	0.52	0.0	0.52
(1,2475)	1:A:101:LYS:HE2	1:A:102:TYR:HE1	2	0.5	0.04	0.5
(1,2475)	1:A:101:LYS:HE2	1:A:102:TYR:HE2	2	0.5	0.04	0.5
(1,2475)	1:A:101:LYS:HE3	1:A:102:TYR:HE1	2	0.5	0.04	0.5
(1,2475)	1:A:101:LYS:HE3	1:A:102:TYR:HE2	2	0.5	0.04	0.5
(1,2232)	1:A:91:ASN:HB3	1:A:94:ALA:HB1	2	0.38	0.01	0.38
(1,2232)	1:A:91:ASN:HB3	1:A:94:ALA:HB2	2	0.38	0.01	0.38
(1,2232)	1:A:91:ASN:HB3	1:A:94:ALA:HB3	2	0.38	0.01	0.38
(2,29)	1:A:2:VAL:H	1:A:54:LYS:HE2	2	0.38	0.18	0.38
(2,29)	1:A:2:VAL:H	1:A:54:LYS:HE3	2	0.38	0.18	0.38
(2,229)	1:A:44:SER:HG	1:A:45:LYS:HD2	2	0.37	0.02	0.37
(2,229)	1:A:44:SER:HG	1:A:45:LYS:HD3	2	0.37	0.02	0.37
(1,1084)	1:A:36:ILE:HG21	1:A:40:TYR:HB2	2	0.36	0.19	0.36
(1,1084)	1:A:36:ILE:HG22	1:A:40:TYR:HB2	2	0.36	0.19	0.36
(1,1084)	1:A:36:ILE:HG23	1:A:40:TYR:HB2	2	0.36	0.19	0.36
(1,633)	1:A:22:ILE:HA	1:A:22:ILE:HD11	2	0.34	0.0	0.34
(1,633)	1:A:22:ILE:HA	1:A:22:ILE:HD12	2	0.34	0.0	0.34
(1,633)	1:A:22:ILE:HA	1:A:22:ILE:HD13	2	0.34	0.0	0.34
(1,1631)	1:A:63:VAL:HG11	1:A:66:LYS:HG2	2	0.34	0.23	0.34
(1,1631)	1:A:63:VAL:HG11	1:A:66:LYS:HG3	2	0.34	0.23	0.34
(1,1631)	1:A:63:VAL:HG12	1:A:66:LYS:HG2	2	0.34	0.23	0.34
(1,1631)	1:A:63:VAL:HG12	1:A:66:LYS:HG3	2	0.34	0.23	0.34
(1,1631)	1:A:63:VAL:HG13	1:A:66:LYS:HG2	2	0.34	0.23	0.34
(1,1631)	1:A:63:VAL:HG13	1:A:66:LYS:HG3	2	0.34	0.23	0.34
(1,1823)	1:A:74:THR:HG21	1:A:89:GLY:H	2	0.32	0.0	0.32
(1,1823)	1:A:74:THR:HG22	1:A:89:GLY:H	2	0.32	0.0	0.32
(1,1823)	1:A:74:THR:HG23	1:A:89:GLY:H	2	0.32	0.0	0.32
(1,1270)	1:A:43:CYS:HB2	1:A:47:TYR:HD1	2	0.3	0.04	0.3
(1,1270)	1:A:43:CYS:HB2	1:A:47:TYR:HD2	2	0.3	0.04	0.3
(1,1693)	1:A:67:GLU:HB2	1:A:69:ILE:HD11	2	0.3	0.11	0.3
(1,1693)	1:A:67:GLU:HB2	1:A:69:ILE:HD12	2	0.3	0.11	0.3
(1,1693)	1:A:67:GLU:HB2	1:A:69:ILE:HD13	2	0.3	0.11	0.3
(1,2389)	1:A:98:LEU:HD21	1:A:99:ILE:H	2	0.29	0.01	0.29
(1,2389)	1:A:98:LEU:HD22	1:A:99:ILE:H	2	0.29	0.01	0.29
(1,2389)	1:A:98:LEU:HD23	1:A:99:ILE:H	2	0.29	0.01	0.29
(1,1959)	1:A:77:VAL:HG11	1:A:102:TYR:HB2	2	0.29	0.0	0.29
(1,1959)	1:A:77:VAL:HG12	1:A:102:TYR:HB2	2	0.29	0.0	0.29
(1,1959)	1:A:77:VAL:HG13	1:A:102:TYR:HB2	2	0.29	0.0	0.29
(2,269)	1:A:52:PHE:HB3	1:A:54:LYS:HD2	2	0.29	0.1	0.29
(2,269)	1:A:52:PHE:HB3	1:A:54:LYS:HD3	2	0.29	0.1	0.29
(1,1976)	1:A:77:VAL:HG21	1:A:98:LEU:HD21	2	0.25	0.07	0.25
(1,1976)	1:A:77:VAL:HG21	1:A:98:LEU:HD22	2	0.25	0.07	0.25

Continued on next page...

Continued from previous page...

Key	Atom-1	Atom-2	Models[1]	Mean (Å)	SD[1] (Å)	Median (Å)
(1,1976)	1:A:77:VAL:HG21	1:A:98:LEU:HD23	2	0.25	0.07	0.25
(1,1976)	1:A:77:VAL:HG22	1:A:98:LEU:HD21	2	0.25	0.07	0.25
(1,1976)	1:A:77:VAL:HG22	1:A:98:LEU:HD22	2	0.25	0.07	0.25
(1,1976)	1:A:77:VAL:HG22	1:A:98:LEU:HD23	2	0.25	0.07	0.25
(1,1976)	1:A:77:VAL:HG23	1:A:98:LEU:HD21	2	0.25	0.07	0.25
(1,1976)	1:A:77:VAL:HG23	1:A:98:LEU:HD22	2	0.25	0.07	0.25
(1,1976)	1:A:77:VAL:HG23	1:A:98:LEU:HD23	2	0.25	0.07	0.25
(1,1100)	1:A:36:ILE:H	1:A:38:PRO:HD2	2	0.2	0.03	0.2
(1,1100)	1:A:36:ILE:H	1:A:38:PRO:HD3	2	0.2	0.03	0.2
(1,96)	1:A:5:VAL:H	1:A:5:VAL:HB	2	0.2	0.02	0.2
(1,2383)	1:A:98:LEU:HD11	1:A:102:TYR:HE1	2	0.2	0.02	0.2
(1,2383)	1:A:98:LEU:HD11	1:A:102:TYR:HE2	2	0.2	0.02	0.2
(1,2383)	1:A:98:LEU:HD12	1:A:102:TYR:HE1	2	0.2	0.02	0.2
(1,2383)	1:A:98:LEU:HD12	1:A:102:TYR:HE2	2	0.2	0.02	0.2
(1,2383)	1:A:98:LEU:HD13	1:A:102:TYR:HE1	2	0.2	0.02	0.2
(1,2383)	1:A:98:LEU:HD13	1:A:102:TYR:HE2	2	0.2	0.02	0.2
(1,2415)	1:A:99:ILE:HD11	1:A:103:ALA:H	2	0.19	0.08	0.19
(1,2415)	1:A:99:ILE:HD12	1:A:103:ALA:H	2	0.19	0.08	0.19
(1,2415)	1:A:99:ILE:HD13	1:A:103:ALA:H	2	0.19	0.08	0.19
(1,933)	1:A:27:ALA:HB1	1:A:57:VAL:H	2	0.18	0.05	0.18
(1,933)	1:A:27:ALA:HB2	1:A:57:VAL:H	2	0.18	0.05	0.18
(1,933)	1:A:27:ALA:HB3	1:A:57:VAL:H	2	0.18	0.05	0.18
(1,252)	1:A:11:PHE:HZ	1:A:67:GLU:HG2	2	0.17	0.05	0.17
(1,252)	1:A:11:PHE:HZ	1:A:67:GLU:HG3	2	0.17	0.05	0.17
(2,100)	1:A:21:VAL:HA	1:A:50:MET:HB3	2	0.16	0.01	0.16
(1,54)	1:A:3:LYS:H	1:A:3:LYS:HG2	2	0.16	0.01	0.16
(1,1762)	1:A:69:ILE:HG21	1:A:74:THR:HG1	2	0.15	0.04	0.15
(1,1762)	1:A:69:ILE:HG22	1:A:74:THR:HG1	2	0.15	0.04	0.15
(1,1762)	1:A:69:ILE:HG23	1:A:74:THR:HG1	2	0.15	0.04	0.15
(1,2004)	1:A:78:TYR:HD1	1:A:15:ILE:HG13	2	0.15	0.04	0.15
(1,2004)	1:A:78:TYR:HD2	1:A:15:ILE:HG13	2	0.15	0.04	0.15
(1,1703)	1:A:67:GLU:H	1:A:68:ASN:HB2	2	0.15	0.02	0.15
(1,1737)	1:A:69:ILE:HD11	1:A:69:ILE:HA	2	0.15	0.0	0.15
(1,1737)	1:A:69:ILE:HD12	1:A:69:ILE:HA	2	0.15	0.0	0.15
(1,1737)	1:A:69:ILE:HD13	1:A:69:ILE:HA	2	0.15	0.0	0.15
(1,2416)	1:A:99:ILE:HD11	1:A:104:ALA:H	2	0.15	0.0	0.15
(1,2416)	1:A:99:ILE:HD12	1:A:104:ALA:H	2	0.15	0.0	0.15
(1,2416)	1:A:99:ILE:HD13	1:A:104:ALA:H	2	0.15	0.0	0.15
(1,106)	1:A:5:VAL:H	1:A:55:VAL:HG21	2	0.14	0.02	0.14
(1,106)	1:A:5:VAL:H	1:A:55:VAL:HG22	2	0.14	0.02	0.14
(1,106)	1:A:5:VAL:H	1:A:55:VAL:HG23	2	0.14	0.02	0.14
(1,1448)	1:A:51:VAL:HG11	1:A:51:VAL:HA	2	0.12	0.01	0.12

Continued on next page...

Continued from previous page...

Key	Atom-1	Atom-2	Models[1]	Mean (Å)	SD[1] (Å)	Median (Å)
(1,1448)	1:A:51:VAL:HG12	1:A:51:VAL:HA	2	0.12	0.01	0.12
(1,1448)	1:A:51:VAL:HG13	1:A:51:VAL:HA	2	0.12	0.01	0.12
(1,1218)	1:A:40:TYR:H	1:A:92:ASP:HA	2	0.12	0.01	0.12
(1,2225)	1:A:90:ALA:H	1:A:90:ALA:HB1	2	0.12	0.01	0.12
(1,2225)	1:A:90:ALA:H	1:A:90:ALA:HB2	2	0.12	0.01	0.12
(1,2225)	1:A:90:ALA:H	1:A:90:ALA:HB3	2	0.12	0.01	0.12
(2,19)	1:A:2:VAL:HB	1:A:3:LYS:HA	2	0.12	0.0	0.12
(2,47)	1:A:5:VAL:HB	1:A:11:PHE:HB2	2	0.12	0.0	0.12
(1,200)	1:A:11:PHE:HA	1:A:15:ILE:H	2	0.11	0.0	0.11
(1,598)	1:A:21:VAL:HA	1:A:21:VAL:HG11	2	0.11	0.0	0.11
(1,598)	1:A:21:VAL:HA	1:A:21:VAL:HG12	2	0.11	0.0	0.11
(1,598)	1:A:21:VAL:HA	1:A:21:VAL:HG13	2	0.11	0.0	0.11
(1,1432)	1:A:50:MET:HG3	1:A:52:PHE:HZ	2	0.11	0.0	0.11

[1]Number of violated models, [2]Standard deviation

9.5 All violated distance restraints (i)

9.5.1 Histogram : Distribution of distance violations (i)

The following histogram shows the distribution of the absolute value of the violation for all violated restraints in the ensemble.

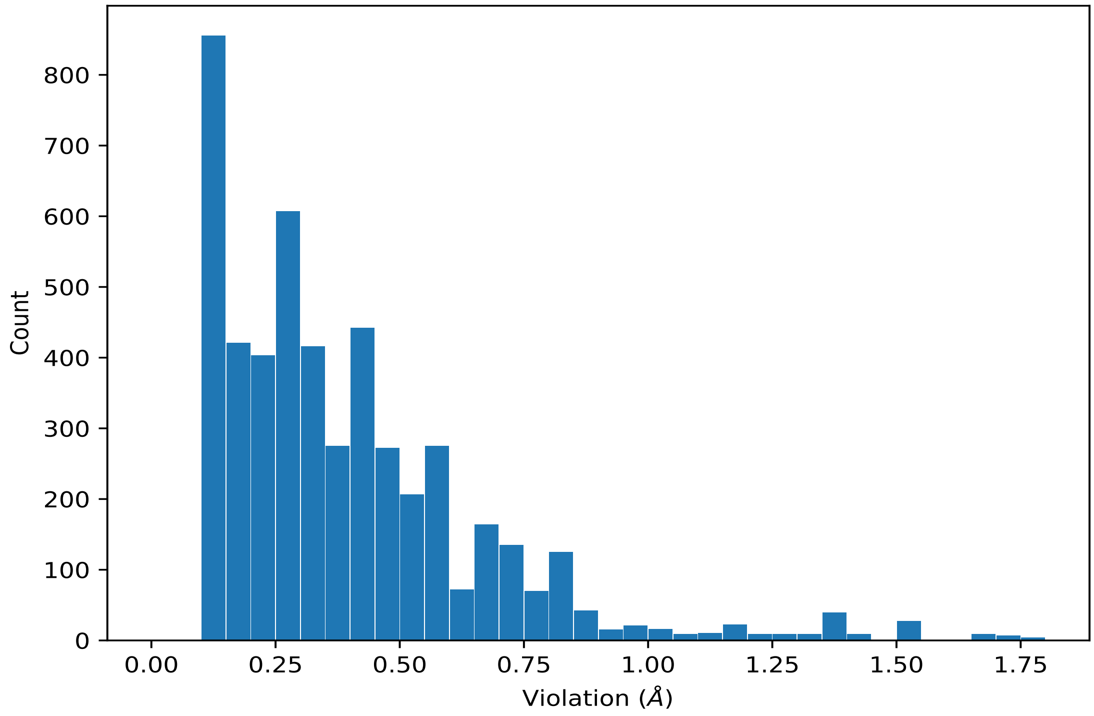

9.5.2 Table : All distance violations (i)

The following table lists the absolute value of the violation for each restraint in the ensemble sorted by its value. The Key (restraint list ID, restraint ID) is the unique identifier for a given restraint. Rows with same key represent combinatorial or ambiguous restraints and are counted as a single restraint.

Key	Atom-1	Atom-2	Model ID	Violation (Å)
(2,397)	1:A:26:PHE:HA	1:A:27:ALA:H	8	1.76
(2,397)	1:A:26:PHE:HA	1:A:27:ALA:H	2	1.75
(2,397)	1:A:26:PHE:HA	1:A:27:ALA:H	6	1.75
(2,397)	1:A:26:PHE:HA	1:A:27:ALA:H	7	1.75
(2,397)	1:A:26:PHE:HA	1:A:27:ALA:H	10	1.74
(2,397)	1:A:26:PHE:HA	1:A:27:ALA:H	3	1.73
(2,397)	1:A:26:PHE:HA	1:A:27:ALA:H	4	1.73
(2,397)	1:A:26:PHE:HA	1:A:27:ALA:H	5	1.73
(2,397)	1:A:26:PHE:HA	1:A:27:ALA:H	9	1.72
(2,410)	1:A:29:TRP:H	1:A:29:TRP:HA	5	1.71
(2,397)	1:A:26:PHE:HA	1:A:27:ALA:H	1	1.71
(2,410)	1:A:29:TRP:H	1:A:29:TRP:HA	2	1.7
(2,410)	1:A:29:TRP:H	1:A:29:TRP:HA	3	1.7
(2,410)	1:A:29:TRP:H	1:A:29:TRP:HA	6	1.7
(2,410)	1:A:29:TRP:H	1:A:29:TRP:HA	7	1.7
(2,410)	1:A:29:TRP:H	1:A:29:TRP:HA	8	1.7
(2,410)	1:A:29:TRP:H	1:A:29:TRP:HA	10	1.7
(2,410)	1:A:29:TRP:H	1:A:29:TRP:HA	1	1.69
(2,410)	1:A:29:TRP:H	1:A:29:TRP:HA	4	1.69
(2,410)	1:A:29:TRP:H	1:A:29:TRP:HA	9	1.69
(2,329)	1:A:69:ILE:HG21	1:A:72:MET:HE1	4	1.54
(2,329)	1:A:69:ILE:HG21	1:A:72:MET:HE2	4	1.54
(2,329)	1:A:69:ILE:HG21	1:A:72:MET:HE3	4	1.54
(2,329)	1:A:69:ILE:HG22	1:A:72:MET:HE1	4	1.54
(2,329)	1:A:69:ILE:HG22	1:A:72:MET:HE2	4	1.54
(2,329)	1:A:69:ILE:HG22	1:A:72:MET:HE3	4	1.54
(2,329)	1:A:69:ILE:HG23	1:A:72:MET:HE1	4	1.54
(2,329)	1:A:69:ILE:HG23	1:A:72:MET:HE2	4	1.54
(2,329)	1:A:69:ILE:HG23	1:A:72:MET:HE3	4	1.54
(2,329)	1:A:69:ILE:HG21	1:A:72:MET:HE1	5	1.53
(2,329)	1:A:69:ILE:HG21	1:A:72:MET:HE2	5	1.53
(2,329)	1:A:69:ILE:HG21	1:A:72:MET:HE3	5	1.53
(2,329)	1:A:69:ILE:HG22	1:A:72:MET:HE1	5	1.53
(2,329)	1:A:69:ILE:HG22	1:A:72:MET:HE2	5	1.53
(2,329)	1:A:69:ILE:HG22	1:A:72:MET:HE3	5	1.53
(2,329)	1:A:69:ILE:HG23	1:A:72:MET:HE1	5	1.53
(2,329)	1:A:69:ILE:HG23	1:A:72:MET:HE2	5	1.53

Continued on next page...

Continued from previous page...

Key	Atom-1	Atom-2	Model ID	Violation (Å)
(2,329)	1:A:69:ILE:HG23	1:A:72:MET:HE3	5	1.53
(2,329)	1:A:69:ILE:HG21	1:A:72:MET:HE1	8	1.51
(2,329)	1:A:69:ILE:HG21	1:A:72:MET:HE2	8	1.51
(2,329)	1:A:69:ILE:HG21	1:A:72:MET:HE3	8	1.51
(2,329)	1:A:69:ILE:HG22	1:A:72:MET:HE1	8	1.51
(2,329)	1:A:69:ILE:HG22	1:A:72:MET:HE2	8	1.51
(2,329)	1:A:69:ILE:HG22	1:A:72:MET:HE3	8	1.51
(2,329)	1:A:69:ILE:HG23	1:A:72:MET:HE1	8	1.51
(2,329)	1:A:69:ILE:HG23	1:A:72:MET:HE2	8	1.51
(2,329)	1:A:69:ILE:HG23	1:A:72:MET:HE3	8	1.51
(2,238)	1:A:46:THR:HG21	1:A:104:ALA:HB1	7	1.42
(2,238)	1:A:46:THR:HG21	1:A:104:ALA:HB2	7	1.42
(2,238)	1:A:46:THR:HG21	1:A:104:ALA:HB3	7	1.42
(2,238)	1:A:46:THR:HG22	1:A:104:ALA:HB1	7	1.42
(2,238)	1:A:46:THR:HG22	1:A:104:ALA:HB2	7	1.42
(2,238)	1:A:46:THR:HG22	1:A:104:ALA:HB3	7	1.42
(2,238)	1:A:46:THR:HG23	1:A:104:ALA:HB1	7	1.42
(2,238)	1:A:46:THR:HG23	1:A:104:ALA:HB2	7	1.42
(2,238)	1:A:46:THR:HG23	1:A:104:ALA:HB3	7	1.42
(2,329)	1:A:69:ILE:HG21	1:A:72:MET:HE1	1	1.4
(2,329)	1:A:69:ILE:HG21	1:A:72:MET:HE2	1	1.4
(2,329)	1:A:69:ILE:HG21	1:A:72:MET:HE3	1	1.4
(2,329)	1:A:69:ILE:HG22	1:A:72:MET:HE1	1	1.4
(2,329)	1:A:69:ILE:HG22	1:A:72:MET:HE2	1	1.4
(2,329)	1:A:69:ILE:HG22	1:A:72:MET:HE3	1	1.4
(2,329)	1:A:69:ILE:HG23	1:A:72:MET:HE1	1	1.4
(2,329)	1:A:69:ILE:HG23	1:A:72:MET:HE2	1	1.4
(2,329)	1:A:69:ILE:HG23	1:A:72:MET:HE3	1	1.4
(2,400)	1:A:27:ALA:HB1	1:A:29:TRP:H	4	1.39
(2,400)	1:A:27:ALA:HB2	1:A:29:TRP:H	4	1.39
(2,400)	1:A:27:ALA:HB3	1:A:29:TRP:H	4	1.39
(2,400)	1:A:27:ALA:HB1	1:A:29:TRP:H	1	1.38
(2,400)	1:A:27:ALA:HB2	1:A:29:TRP:H	1	1.38
(2,400)	1:A:27:ALA:HB3	1:A:29:TRP:H	1	1.38
(2,400)	1:A:27:ALA:HB1	1:A:29:TRP:H	2	1.38
(2,400)	1:A:27:ALA:HB2	1:A:29:TRP:H	2	1.38
(2,400)	1:A:27:ALA:HB3	1:A:29:TRP:H	2	1.38
(2,400)	1:A:27:ALA:HB1	1:A:29:TRP:H	9	1.38
(2,400)	1:A:27:ALA:HB2	1:A:29:TRP:H	9	1.38
(2,400)	1:A:27:ALA:HB3	1:A:29:TRP:H	9	1.38
(2,400)	1:A:27:ALA:HB1	1:A:29:TRP:H	5	1.37
(2,400)	1:A:27:ALA:HB2	1:A:29:TRP:H	5	1.37

Continued on next page...

Continued from previous page...

Key	Atom-1	Atom-2	Model ID	Violation (Å)
(2,400)	1:A:27:ALA:HB3	1:A:29:TRP:H	5	1.37
(2,400)	1:A:27:ALA:HB1	1:A:29:TRP:H	7	1.37
(2,400)	1:A:27:ALA:HB2	1:A:29:TRP:H	7	1.37
(2,400)	1:A:27:ALA:HB3	1:A:29:TRP:H	7	1.37
(2,400)	1:A:27:ALA:HB1	1:A:29:TRP:H	10	1.37
(2,400)	1:A:27:ALA:HB2	1:A:29:TRP:H	10	1.37
(2,400)	1:A:27:ALA:HB3	1:A:29:TRP:H	10	1.37
(2,400)	1:A:27:ALA:HB1	1:A:29:TRP:H	3	1.36
(2,400)	1:A:27:ALA:HB2	1:A:29:TRP:H	3	1.36
(2,400)	1:A:27:ALA:HB3	1:A:29:TRP:H	3	1.36
(2,400)	1:A:27:ALA:HB1	1:A:29:TRP:H	8	1.36
(2,400)	1:A:27:ALA:HB2	1:A:29:TRP:H	8	1.36
(2,400)	1:A:27:ALA:HB3	1:A:29:TRP:H	8	1.36
(2,400)	1:A:27:ALA:HB1	1:A:29:TRP:H	6	1.35
(2,400)	1:A:27:ALA:HB2	1:A:29:TRP:H	6	1.35
(2,400)	1:A:27:ALA:HB3	1:A:29:TRP:H	6	1.35
(2,238)	1:A:46:THR:HG21	1:A:104:ALA:HB1	10	1.3
(2,238)	1:A:46:THR:HG21	1:A:104:ALA:HB2	10	1.3
(2,238)	1:A:46:THR:HG21	1:A:104:ALA:HB3	10	1.3
(2,238)	1:A:46:THR:HG22	1:A:104:ALA:HB1	10	1.3
(2,238)	1:A:46:THR:HG22	1:A:104:ALA:HB2	10	1.3
(2,238)	1:A:46:THR:HG22	1:A:104:ALA:HB3	10	1.3
(2,238)	1:A:46:THR:HG23	1:A:104:ALA:HB1	10	1.3
(2,238)	1:A:46:THR:HG23	1:A:104:ALA:HB2	10	1.3
(2,238)	1:A:46:THR:HG23	1:A:104:ALA:HB3	10	1.3
(2,238)	1:A:46:THR:HG21	1:A:104:ALA:HB1	3	1.28
(2,238)	1:A:46:THR:HG21	1:A:104:ALA:HB2	3	1.28
(2,238)	1:A:46:THR:HG21	1:A:104:ALA:HB3	3	1.28
(2,238)	1:A:46:THR:HG22	1:A:104:ALA:HB1	3	1.28
(2,238)	1:A:46:THR:HG22	1:A:104:ALA:HB2	3	1.28
(2,238)	1:A:46:THR:HG22	1:A:104:ALA:HB3	3	1.28
(2,238)	1:A:46:THR:HG23	1:A:104:ALA:HB1	3	1.28
(2,238)	1:A:46:THR:HG23	1:A:104:ALA:HB2	3	1.28
(2,238)	1:A:46:THR:HG23	1:A:104:ALA:HB3	3	1.28
(2,238)	1:A:46:THR:HG21	1:A:104:ALA:HB1	8	1.23
(2,238)	1:A:46:THR:HG21	1:A:104:ALA:HB2	8	1.23
(2,238)	1:A:46:THR:HG21	1:A:104:ALA:HB3	8	1.23
(2,238)	1:A:46:THR:HG22	1:A:104:ALA:HB1	8	1.23
(2,238)	1:A:46:THR:HG22	1:A:104:ALA:HB2	8	1.23
(2,238)	1:A:46:THR:HG22	1:A:104:ALA:HB3	8	1.23
(2,238)	1:A:46:THR:HG23	1:A:104:ALA:HB1	8	1.23
(2,238)	1:A:46:THR:HG23	1:A:104:ALA:HB2	8	1.23

Continued on next page...

Continued from previous page...

Key	Atom-1	Atom-2	Model ID	Violation (Å)
(2,238)	1:A:46:THR:HG23	1:A:104:ALA:HB3	8	1.23
(2,407)	1:A:29:TRP:HD1	1:A:29:TRP:H	7	1.19
(2,406)	1:A:29:TRP:HA	1:A:30:CYS:H	1	1.19
(2,406)	1:A:29:TRP:HA	1:A:30:CYS:H	9	1.19
(2,199)	1:A:36:ILE:HG21	1:A:40:TYR:HD1	5	1.19
(2,199)	1:A:36:ILE:HG21	1:A:40:TYR:HD2	5	1.19
(2,199)	1:A:36:ILE:HG22	1:A:40:TYR:HD1	5	1.19
(2,199)	1:A:36:ILE:HG22	1:A:40:TYR:HD2	5	1.19
(2,199)	1:A:36:ILE:HG23	1:A:40:TYR:HD1	5	1.19
(2,199)	1:A:36:ILE:HG23	1:A:40:TYR:HD2	5	1.19
(2,406)	1:A:29:TRP:HA	1:A:30:CYS:H	4	1.17
(2,199)	1:A:36:ILE:HG21	1:A:40:TYR:HD1	1	1.17
(2,199)	1:A:36:ILE:HG21	1:A:40:TYR:HD2	1	1.17
(2,199)	1:A:36:ILE:HG22	1:A:40:TYR:HD1	1	1.17
(2,199)	1:A:36:ILE:HG22	1:A:40:TYR:HD2	1	1.17
(2,199)	1:A:36:ILE:HG23	1:A:40:TYR:HD1	1	1.17
(2,199)	1:A:36:ILE:HG23	1:A:40:TYR:HD2	1	1.17
(2,407)	1:A:29:TRP:HD1	1:A:29:TRP:H	8	1.16
(2,407)	1:A:29:TRP:HD1	1:A:29:TRP:H	10	1.16
(2,406)	1:A:29:TRP:HA	1:A:30:CYS:H	2	1.16
(2,406)	1:A:29:TRP:HA	1:A:30:CYS:H	3	1.16
(2,406)	1:A:29:TRP:HA	1:A:30:CYS:H	6	1.16
(2,406)	1:A:29:TRP:HA	1:A:30:CYS:H	8	1.16
(2,407)	1:A:29:TRP:HD1	1:A:29:TRP:H	2	1.15
(2,407)	1:A:29:TRP:HD1	1:A:29:TRP:H	5	1.15
(2,407)	1:A:29:TRP:HD1	1:A:29:TRP:H	6	1.15
(2,406)	1:A:29:TRP:HA	1:A:30:CYS:H	10	1.15
(2,407)	1:A:29:TRP:HD1	1:A:29:TRP:H	3	1.14
(2,406)	1:A:29:TRP:HA	1:A:30:CYS:H	7	1.14
(2,407)	1:A:29:TRP:HD1	1:A:29:TRP:H	4	1.12
(2,406)	1:A:29:TRP:HA	1:A:30:CYS:H	5	1.12
(2,407)	1:A:29:TRP:HD1	1:A:29:TRP:H	1	1.11
(2,407)	1:A:29:TRP:HD1	1:A:29:TRP:H	9	1.11
(2,238)	1:A:46:THR:HG21	1:A:104:ALA:HB1	5	1.07
(2,238)	1:A:46:THR:HG21	1:A:104:ALA:HB2	5	1.07
(2,238)	1:A:46:THR:HG21	1:A:104:ALA:HB3	5	1.07
(2,238)	1:A:46:THR:HG22	1:A:104:ALA:HB1	5	1.07
(2,238)	1:A:46:THR:HG22	1:A:104:ALA:HB2	5	1.07
(2,238)	1:A:46:THR:HG22	1:A:104:ALA:HB3	5	1.07
(2,238)	1:A:46:THR:HG23	1:A:104:ALA:HB1	5	1.07
(2,238)	1:A:46:THR:HG23	1:A:104:ALA:HB2	5	1.07
(2,238)	1:A:46:THR:HG23	1:A:104:ALA:HB3	5	1.07

Continued on next page...

Continued from previous page...

Key	Atom-1	Atom-2	Model ID	Violation (Å)
(2,140)	1:A:26:PHE:HE1	1:A:54:LYS:HD2	3	1.04
(2,140)	1:A:26:PHE:HE1	1:A:54:LYS:HD3	3	1.04
(2,140)	1:A:26:PHE:HE2	1:A:54:LYS:HD2	3	1.04
(2,140)	1:A:26:PHE:HE2	1:A:54:LYS:HD3	3	1.04
(2,403)	1:A:28:GLU:HA	1:A:29:TRP:H	5	1.03
(2,403)	1:A:28:GLU:HA	1:A:29:TRP:H	2	1.02
(2,403)	1:A:28:GLU:HA	1:A:29:TRP:H	3	1.02
(2,403)	1:A:28:GLU:HA	1:A:29:TRP:H	6	1.02
(2,403)	1:A:28:GLU:HA	1:A:29:TRP:H	7	1.02
(2,403)	1:A:28:GLU:HA	1:A:29:TRP:H	8	1.02
(2,403)	1:A:28:GLU:HA	1:A:29:TRP:H	10	1.02
(2,405)	1:A:28:GLU:H	1:A:29:TRP:H	7	1.0
(2,118)	1:A:25:PHE:HD1	1:A:67:GLU:HG2	6	1.0
(2,118)	1:A:25:PHE:HD1	1:A:67:GLU:HG3	6	1.0
(2,118)	1:A:25:PHE:HD2	1:A:67:GLU:HG2	6	1.0
(2,118)	1:A:25:PHE:HD2	1:A:67:GLU:HG3	6	1.0
(2,405)	1:A:28:GLU:H	1:A:29:TRP:H	2	0.99
(2,405)	1:A:28:GLU:H	1:A:29:TRP:H	3	0.99
(2,405)	1:A:28:GLU:H	1:A:29:TRP:H	5	0.99
(2,405)	1:A:28:GLU:H	1:A:29:TRP:H	8	0.99
(2,405)	1:A:28:GLU:H	1:A:29:TRP:H	10	0.99
(2,238)	1:A:46:THR:HG21	1:A:104:ALA:HB1	6	0.99
(2,238)	1:A:46:THR:HG21	1:A:104:ALA:HB2	6	0.99
(2,238)	1:A:46:THR:HG21	1:A:104:ALA:HB3	6	0.99
(2,238)	1:A:46:THR:HG22	1:A:104:ALA:HB1	6	0.99
(2,238)	1:A:46:THR:HG22	1:A:104:ALA:HB2	6	0.99
(2,238)	1:A:46:THR:HG22	1:A:104:ALA:HB3	6	0.99
(2,238)	1:A:46:THR:HG23	1:A:104:ALA:HB1	6	0.99
(2,238)	1:A:46:THR:HG23	1:A:104:ALA:HB2	6	0.99
(2,238)	1:A:46:THR:HG23	1:A:104:ALA:HB3	6	0.99
(2,405)	1:A:28:GLU:H	1:A:29:TRP:H	6	0.98
(2,403)	1:A:28:GLU:HA	1:A:29:TRP:H	1	0.98
(2,403)	1:A:28:GLU:HA	1:A:29:TRP:H	4	0.98
(2,403)	1:A:28:GLU:HA	1:A:29:TRP:H	9	0.98
(2,405)	1:A:28:GLU:H	1:A:29:TRP:H	1	0.97
(2,405)	1:A:28:GLU:H	1:A:29:TRP:H	9	0.97
(2,405)	1:A:28:GLU:H	1:A:29:TRP:H	4	0.96
(2,327)	1:A:69:ILE:HG21	1:A:72:MET:HB3	3	0.91
(2,327)	1:A:69:ILE:HG22	1:A:72:MET:HB3	3	0.91
(2,327)	1:A:69:ILE:HG23	1:A:72:MET:HB3	3	0.91
(2,327)	1:A:69:ILE:HG21	1:A:72:MET:HB3	6	0.91
(2,327)	1:A:69:ILE:HG22	1:A:72:MET:HB3	6	0.91

Continued on next page...

Continued from previous page...

Key	Atom-1	Atom-2	Model ID	Violation (Å)
(2,327)	1:A:69:ILE:HG23	1:A:72:MET:HB3	6	0.91
(2,327)	1:A:69:ILE:HG21	1:A:72:MET:HB3	9	0.91
(2,327)	1:A:69:ILE:HG22	1:A:72:MET:HB3	9	0.91
(2,327)	1:A:69:ILE:HG23	1:A:72:MET:HB3	9	0.91
(2,327)	1:A:69:ILE:HG21	1:A:72:MET:HB3	2	0.9
(2,327)	1:A:69:ILE:HG22	1:A:72:MET:HB3	2	0.9
(2,327)	1:A:69:ILE:HG23	1:A:72:MET:HB3	2	0.9
(2,326)	1:A:69:ILE:HG21	1:A:72:MET:HA	1	0.9
(2,326)	1:A:69:ILE:HG22	1:A:72:MET:HA	1	0.9
(2,326)	1:A:69:ILE:HG23	1:A:72:MET:HA	1	0.9
(2,327)	1:A:69:ILE:HG21	1:A:72:MET:HB3	7	0.89
(2,327)	1:A:69:ILE:HG22	1:A:72:MET:HB3	7	0.89
(2,327)	1:A:69:ILE:HG23	1:A:72:MET:HB3	7	0.89
(2,326)	1:A:69:ILE:HG21	1:A:72:MET:HA	4	0.89
(2,326)	1:A:69:ILE:HG22	1:A:72:MET:HA	4	0.89
(2,326)	1:A:69:ILE:HG23	1:A:72:MET:HA	4	0.89
(2,328)	1:A:69:ILE:HG21	1:A:72:MET:HB2	5	0.88
(2,328)	1:A:69:ILE:HG22	1:A:72:MET:HB2	5	0.88
(2,328)	1:A:69:ILE:HG23	1:A:72:MET:HB2	5	0.88
(2,326)	1:A:69:ILE:HG21	1:A:72:MET:HA	8	0.88
(2,326)	1:A:69:ILE:HG22	1:A:72:MET:HA	8	0.88
(2,326)	1:A:69:ILE:HG23	1:A:72:MET:HA	8	0.88
(2,326)	1:A:69:ILE:HG21	1:A:72:MET:HA	10	0.88
(2,326)	1:A:69:ILE:HG22	1:A:72:MET:HA	10	0.88
(2,326)	1:A:69:ILE:HG23	1:A:72:MET:HA	10	0.88
(2,245)	1:A:47:TYR:HB3	1:A:50:MET:HE1	10	0.88
(2,245)	1:A:47:TYR:HB3	1:A:50:MET:HE2	10	0.88
(2,245)	1:A:47:TYR:HB3	1:A:50:MET:HE3	10	0.88
(2,328)	1:A:69:ILE:HG21	1:A:72:MET:HB2	1	0.87
(2,328)	1:A:69:ILE:HG22	1:A:72:MET:HB2	1	0.87
(2,328)	1:A:69:ILE:HG23	1:A:72:MET:HB2	1	0.87
(2,328)	1:A:69:ILE:HG21	1:A:72:MET:HB2	7	0.87
(2,328)	1:A:69:ILE:HG22	1:A:72:MET:HB2	7	0.87
(2,328)	1:A:69:ILE:HG23	1:A:72:MET:HB2	7	0.87
(2,327)	1:A:69:ILE:HG21	1:A:72:MET:HB3	10	0.87
(2,327)	1:A:69:ILE:HG22	1:A:72:MET:HB3	10	0.87
(2,327)	1:A:69:ILE:HG23	1:A:72:MET:HB3	10	0.87
(2,326)	1:A:69:ILE:HG21	1:A:72:MET:HA	5	0.87
(2,326)	1:A:69:ILE:HG22	1:A:72:MET:HA	5	0.87
(2,326)	1:A:69:ILE:HG23	1:A:72:MET:HA	5	0.87
(2,245)	1:A:47:TYR:HB3	1:A:50:MET:HE1	3	0.87
(2,245)	1:A:47:TYR:HB3	1:A:50:MET:HE2	3	0.87

Continued on next page...

Continued from previous page...

Key	Atom-1	Atom-2	Model ID	Violation (Å)
(2,245)	1:A:47:TYR:HB3	1:A:50:MET:HE3	3	0.87
(2,328)	1:A:69:ILE:HG21	1:A:72:MET:HB2	2	0.86
(2,328)	1:A:69:ILE:HG22	1:A:72:MET:HB2	2	0.86
(2,328)	1:A:69:ILE:HG23	1:A:72:MET:HB2	2	0.86
(2,325)	1:A:69:ILE:HG21	1:A:71:SER:HA	3	0.86
(2,325)	1:A:69:ILE:HG22	1:A:71:SER:HA	3	0.86
(2,325)	1:A:69:ILE:HG23	1:A:71:SER:HA	3	0.86
(2,245)	1:A:47:TYR:HB3	1:A:50:MET:HE1	1	0.86
(2,245)	1:A:47:TYR:HB3	1:A:50:MET:HE2	1	0.86
(2,245)	1:A:47:TYR:HB3	1:A:50:MET:HE3	1	0.86
(2,49)	1:A:5:VAL:HG21	1:A:7:SER:HA	4	0.85
(2,49)	1:A:5:VAL:HG22	1:A:7:SER:HA	4	0.85
(2,49)	1:A:5:VAL:HG23	1:A:7:SER:HA	4	0.85
(2,49)	1:A:5:VAL:HG21	1:A:7:SER:HA	6	0.85
(2,49)	1:A:5:VAL:HG22	1:A:7:SER:HA	6	0.85
(2,49)	1:A:5:VAL:HG23	1:A:7:SER:HA	6	0.85
(2,328)	1:A:69:ILE:HG21	1:A:72:MET:HB2	8	0.85
(2,328)	1:A:69:ILE:HG22	1:A:72:MET:HB2	8	0.85
(2,328)	1:A:69:ILE:HG23	1:A:72:MET:HB2	8	0.85
(2,325)	1:A:69:ILE:HG21	1:A:71:SER:HA	6	0.85
(2,325)	1:A:69:ILE:HG22	1:A:71:SER:HA	6	0.85
(2,325)	1:A:69:ILE:HG23	1:A:71:SER:HA	6	0.85
(2,264)	1:A:50:MET:HE1	1:A:99:ILE:HA	7	0.85
(2,264)	1:A:50:MET:HE2	1:A:99:ILE:HA	7	0.85
(2,264)	1:A:50:MET:HE3	1:A:99:ILE:HA	7	0.85
(2,245)	1:A:47:TYR:HB3	1:A:50:MET:HE1	9	0.85
(2,245)	1:A:47:TYR:HB3	1:A:50:MET:HE2	9	0.85
(2,245)	1:A:47:TYR:HB3	1:A:50:MET:HE3	9	0.85
(2,131)	1:A:26:PHE:HA	1:A:72:MET:HE1	1	0.85
(2,131)	1:A:26:PHE:HA	1:A:72:MET:HE2	1	0.85
(2,131)	1:A:26:PHE:HA	1:A:72:MET:HE3	1	0.85
(2,131)	1:A:26:PHE:HA	1:A:72:MET:HE1	5	0.85
(2,131)	1:A:26:PHE:HA	1:A:72:MET:HE2	5	0.85
(2,131)	1:A:26:PHE:HA	1:A:72:MET:HE3	5	0.85
(2,131)	1:A:26:PHE:HA	1:A:72:MET:HE1	6	0.85
(2,131)	1:A:26:PHE:HA	1:A:72:MET:HE2	6	0.85
(2,131)	1:A:26:PHE:HA	1:A:72:MET:HE3	6	0.85
(2,127)	1:A:25:PHE:HZ	1:A:69:ILE:HD11	10	0.85
(2,127)	1:A:25:PHE:HZ	1:A:69:ILE:HD12	10	0.85
(2,127)	1:A:25:PHE:HZ	1:A:69:ILE:HD13	10	0.85
(2,126)	1:A:25:PHE:HZ	1:A:69:ILE:HD11	10	0.85
(2,126)	1:A:25:PHE:HZ	1:A:69:ILE:HD12	10	0.85

Continued on next page...

Continued from previous page...

Key	Atom-1	Atom-2	Model ID	Violation (Å)
(2,126)	1:A:25:PHE:HZ	1:A:69:ILE:HD13	10	0.85
(2,49)	1:A:5:VAL:HG21	1:A:7:SER:HA	3	0.84
(2,49)	1:A:5:VAL:HG22	1:A:7:SER:HA	3	0.84
(2,49)	1:A:5:VAL:HG23	1:A:7:SER:HA	3	0.84
(2,49)	1:A:5:VAL:HG21	1:A:7:SER:HA	9	0.84
(2,49)	1:A:5:VAL:HG22	1:A:7:SER:HA	9	0.84
(2,49)	1:A:5:VAL:HG23	1:A:7:SER:HA	9	0.84
(2,328)	1:A:69:ILE:HG21	1:A:72:MET:HB2	9	0.84
(2,328)	1:A:69:ILE:HG22	1:A:72:MET:HB2	9	0.84
(2,328)	1:A:69:ILE:HG23	1:A:72:MET:HB2	9	0.84
(2,325)	1:A:69:ILE:HG21	1:A:71:SER:HA	9	0.84
(2,325)	1:A:69:ILE:HG22	1:A:71:SER:HA	9	0.84
(2,325)	1:A:69:ILE:HG23	1:A:71:SER:HA	9	0.84
(2,245)	1:A:47:TYR:HB3	1:A:50:MET:HE1	2	0.84
(2,245)	1:A:47:TYR:HB3	1:A:50:MET:HE2	2	0.84
(2,245)	1:A:47:TYR:HB3	1:A:50:MET:HE3	2	0.84
(2,199)	1:A:36:ILE:HG21	1:A:40:TYR:HD1	8	0.84
(2,199)	1:A:36:ILE:HG21	1:A:40:TYR:HD2	8	0.84
(2,199)	1:A:36:ILE:HG22	1:A:40:TYR:HD1	8	0.84
(2,199)	1:A:36:ILE:HG22	1:A:40:TYR:HD2	8	0.84
(2,199)	1:A:36:ILE:HG23	1:A:40:TYR:HD1	8	0.84
(2,199)	1:A:36:ILE:HG23	1:A:40:TYR:HD2	8	0.84
(2,193)	1:A:29:TRP:HZ3	1:A:72:MET:HE1	2	0.84
(2,193)	1:A:29:TRP:HZ3	1:A:72:MET:HE2	2	0.84
(2,193)	1:A:29:TRP:HZ3	1:A:72:MET:HE3	2	0.84
(2,140)	1:A:26:PHE:HE1	1:A:54:LYS:HD2	2	0.84
(2,140)	1:A:26:PHE:HE1	1:A:54:LYS:HD3	2	0.84
(2,140)	1:A:26:PHE:HE2	1:A:54:LYS:HD2	2	0.84
(2,140)	1:A:26:PHE:HE2	1:A:54:LYS:HD3	2	0.84
(2,131)	1:A:26:PHE:HA	1:A:72:MET:HE1	3	0.84
(2,131)	1:A:26:PHE:HA	1:A:72:MET:HE2	3	0.84
(2,131)	1:A:26:PHE:HA	1:A:72:MET:HE3	3	0.84
(2,127)	1:A:25:PHE:HZ	1:A:69:ILE:HD11	3	0.84
(2,127)	1:A:25:PHE:HZ	1:A:69:ILE:HD12	3	0.84
(2,127)	1:A:25:PHE:HZ	1:A:69:ILE:HD13	3	0.84
(2,127)	1:A:25:PHE:HZ	1:A:69:ILE:HD11	6	0.84
(2,127)	1:A:25:PHE:HZ	1:A:69:ILE:HD12	6	0.84
(2,127)	1:A:25:PHE:HZ	1:A:69:ILE:HD13	6	0.84
(2,126)	1:A:25:PHE:HZ	1:A:69:ILE:HD11	3	0.84
(2,126)	1:A:25:PHE:HZ	1:A:69:ILE:HD12	3	0.84
(2,126)	1:A:25:PHE:HZ	1:A:69:ILE:HD13	3	0.84
(2,126)	1:A:25:PHE:HZ	1:A:69:ILE:HD11	6	0.84

Continued on next page...

Continued from previous page...

Key	Atom-1	Atom-2	Model ID	Violation (Å)
(2,126)	1:A:25:PHE:HZ	1:A:69:ILE:HD12	6	0.84
(2,126)	1:A:25:PHE:HZ	1:A:69:ILE:HD13	6	0.84
(2,326)	1:A:69:ILE:HG21	1:A:72:MET:HA	6	0.83
(2,326)	1:A:69:ILE:HG22	1:A:72:MET:HA	6	0.83
(2,326)	1:A:69:ILE:HG23	1:A:72:MET:HA	6	0.83
(2,49)	1:A:5:VAL:HG21	1:A:7:SER:HA	10	0.82
(2,49)	1:A:5:VAL:HG22	1:A:7:SER:HA	10	0.82
(2,49)	1:A:5:VAL:HG23	1:A:7:SER:HA	10	0.82
(2,326)	1:A:69:ILE:HG21	1:A:72:MET:HA	2	0.82
(2,326)	1:A:69:ILE:HG22	1:A:72:MET:HA	2	0.82
(2,326)	1:A:69:ILE:HG23	1:A:72:MET:HA	2	0.82
(2,326)	1:A:69:ILE:HG21	1:A:72:MET:HA	7	0.82
(2,326)	1:A:69:ILE:HG22	1:A:72:MET:HA	7	0.82
(2,326)	1:A:69:ILE:HG23	1:A:72:MET:HA	7	0.82
(2,326)	1:A:69:ILE:HG21	1:A:72:MET:HA	9	0.82
(2,326)	1:A:69:ILE:HG22	1:A:72:MET:HA	9	0.82
(2,326)	1:A:69:ILE:HG23	1:A:72:MET:HA	9	0.82
(2,245)	1:A:47:TYR:HB3	1:A:50:MET:HE1	4	0.82
(2,245)	1:A:47:TYR:HB3	1:A:50:MET:HE2	4	0.82
(2,245)	1:A:47:TYR:HB3	1:A:50:MET:HE3	4	0.82
(2,245)	1:A:47:TYR:HB3	1:A:50:MET:HE1	5	0.82
(2,245)	1:A:47:TYR:HB3	1:A:50:MET:HE2	5	0.82
(2,245)	1:A:47:TYR:HB3	1:A:50:MET:HE3	5	0.82
(2,49)	1:A:5:VAL:HG21	1:A:7:SER:HA	1	0.81
(2,49)	1:A:5:VAL:HG22	1:A:7:SER:HA	1	0.81
(2,49)	1:A:5:VAL:HG23	1:A:7:SER:HA	1	0.81
(2,199)	1:A:36:ILE:HG21	1:A:40:TYR:HD1	7	0.81
(2,199)	1:A:36:ILE:HG21	1:A:40:TYR:HD2	7	0.81
(2,199)	1:A:36:ILE:HG22	1:A:40:TYR:HD1	7	0.81
(2,199)	1:A:36:ILE:HG22	1:A:40:TYR:HD2	7	0.81
(2,199)	1:A:36:ILE:HG23	1:A:40:TYR:HD1	7	0.81
(2,199)	1:A:36:ILE:HG23	1:A:40:TYR:HD2	7	0.81
(2,137)	1:A:26:PHE:HB2	1:A:72:MET:HE1	6	0.81
(2,137)	1:A:26:PHE:HB2	1:A:72:MET:HE2	6	0.81
(2,137)	1:A:26:PHE:HB2	1:A:72:MET:HE3	6	0.81
(2,118)	1:A:25:PHE:HD1	1:A:67:GLU:HG2	4	0.81
(2,118)	1:A:25:PHE:HD1	1:A:67:GLU:HG3	4	0.81
(2,118)	1:A:25:PHE:HD2	1:A:67:GLU:HG2	4	0.81
(2,118)	1:A:25:PHE:HD2	1:A:67:GLU:HG3	4	0.81
(2,183)	1:A:29:TRP:HE1	1:A:72:MET:HE1	7	0.8
(2,183)	1:A:29:TRP:HE1	1:A:72:MET:HE2	7	0.8
(2,183)	1:A:29:TRP:HE1	1:A:72:MET:HE3	7	0.8

Continued on next page...

Continued from previous page...

Key	Atom-1	Atom-2	Model ID	Violation (Å)
(2,150)	1:A:27:ALA:HB1	1:A:58:ASP:HB3	1	0.8
(2,150)	1:A:27:ALA:HB2	1:A:58:ASP:HB3	1	0.8
(2,150)	1:A:27:ALA:HB3	1:A:58:ASP:HB3	1	0.8
(2,127)	1:A:25:PHE:HZ	1:A:69:ILE:HD11	1	0.8
(2,127)	1:A:25:PHE:HZ	1:A:69:ILE:HD12	1	0.8
(2,127)	1:A:25:PHE:HZ	1:A:69:ILE:HD13	1	0.8
(2,126)	1:A:25:PHE:HZ	1:A:69:ILE:HD11	1	0.8
(2,126)	1:A:25:PHE:HZ	1:A:69:ILE:HD12	1	0.8
(2,126)	1:A:25:PHE:HZ	1:A:69:ILE:HD13	1	0.8
(2,49)	1:A:5:VAL:HG21	1:A:7:SER:HA	2	0.79
(2,49)	1:A:5:VAL:HG22	1:A:7:SER:HA	2	0.79
(2,49)	1:A:5:VAL:HG23	1:A:7:SER:HA	2	0.79
(2,238)	1:A:46:THR:HG21	1:A:104:ALA:HB1	4	0.78
(2,238)	1:A:46:THR:HG21	1:A:104:ALA:HB2	4	0.78
(2,238)	1:A:46:THR:HG21	1:A:104:ALA:HB3	4	0.78
(2,238)	1:A:46:THR:HG22	1:A:104:ALA:HB1	4	0.78
(2,238)	1:A:46:THR:HG22	1:A:104:ALA:HB2	4	0.78
(2,238)	1:A:46:THR:HG22	1:A:104:ALA:HB3	4	0.78
(2,238)	1:A:46:THR:HG23	1:A:104:ALA:HB1	4	0.78
(2,238)	1:A:46:THR:HG23	1:A:104:ALA:HB2	4	0.78
(2,238)	1:A:46:THR:HG23	1:A:104:ALA:HB3	4	0.78
(1,575)	1:A:20:LEU:HD11	1:A:77:VAL:HG11	1	0.78
(1,575)	1:A:20:LEU:HD11	1:A:77:VAL:HG12	1	0.78
(1,575)	1:A:20:LEU:HD11	1:A:77:VAL:HG13	1	0.78
(1,575)	1:A:20:LEU:HD12	1:A:77:VAL:HG11	1	0.78
(1,575)	1:A:20:LEU:HD12	1:A:77:VAL:HG12	1	0.78
(1,575)	1:A:20:LEU:HD12	1:A:77:VAL:HG13	1	0.78
(1,575)	1:A:20:LEU:HD13	1:A:77:VAL:HG11	1	0.78
(1,575)	1:A:20:LEU:HD13	1:A:77:VAL:HG12	1	0.78
(1,575)	1:A:20:LEU:HD13	1:A:77:VAL:HG13	1	0.78
(1,575)	1:A:20:LEU:HD11	1:A:77:VAL:HG11	6	0.78
(1,575)	1:A:20:LEU:HD11	1:A:77:VAL:HG12	6	0.78
(1,575)	1:A:20:LEU:HD11	1:A:77:VAL:HG13	6	0.78
(1,575)	1:A:20:LEU:HD12	1:A:77:VAL:HG11	6	0.78
(1,575)	1:A:20:LEU:HD12	1:A:77:VAL:HG12	6	0.78
(1,575)	1:A:20:LEU:HD12	1:A:77:VAL:HG13	6	0.78
(1,575)	1:A:20:LEU:HD13	1:A:77:VAL:HG11	6	0.78
(1,575)	1:A:20:LEU:HD13	1:A:77:VAL:HG12	6	0.78
(1,575)	1:A:20:LEU:HD13	1:A:77:VAL:HG13	6	0.78
(1,2546)	1:A:29:TRP:HD1	1:A:29:TRP:HA	9	0.77
(2,49)	1:A:5:VAL:HG21	1:A:7:SER:HA	7	0.76
(2,49)	1:A:5:VAL:HG22	1:A:7:SER:HA	7	0.76

Continued on next page...

Continued from previous page...

Key	Atom-1	Atom-2	Model ID	Violation (Å)
(2,49)	1:A:5:VAL:HG23	1:A:7:SER:HA	7	0.76
(2,238)	1:A:46:THR:HG21	1:A:104:ALA:HB1	2	0.76
(2,238)	1:A:46:THR:HG21	1:A:104:ALA:HB2	2	0.76
(2,238)	1:A:46:THR:HG21	1:A:104:ALA:HB3	2	0.76
(2,238)	1:A:46:THR:HG22	1:A:104:ALA:HB1	2	0.76
(2,238)	1:A:46:THR:HG22	1:A:104:ALA:HB2	2	0.76
(2,238)	1:A:46:THR:HG22	1:A:104:ALA:HB3	2	0.76
(2,238)	1:A:46:THR:HG23	1:A:104:ALA:HB1	2	0.76
(2,238)	1:A:46:THR:HG23	1:A:104:ALA:HB2	2	0.76
(2,238)	1:A:46:THR:HG23	1:A:104:ALA:HB3	2	0.76
(1,575)	1:A:20:LEU:HD11	1:A:77:VAL:HG11	5	0.76
(1,575)	1:A:20:LEU:HD11	1:A:77:VAL:HG12	5	0.76
(1,575)	1:A:20:LEU:HD11	1:A:77:VAL:HG13	5	0.76
(1,575)	1:A:20:LEU:HD12	1:A:77:VAL:HG11	5	0.76
(1,575)	1:A:20:LEU:HD12	1:A:77:VAL:HG12	5	0.76
(1,575)	1:A:20:LEU:HD12	1:A:77:VAL:HG13	5	0.76
(1,575)	1:A:20:LEU:HD13	1:A:77:VAL:HG11	5	0.76
(1,575)	1:A:20:LEU:HD13	1:A:77:VAL:HG12	5	0.76
(1,575)	1:A:20:LEU:HD13	1:A:77:VAL:HG13	5	0.76
(1,2546)	1:A:29:TRP:HD1	1:A:29:TRP:HA	1	0.76
(1,2546)	1:A:29:TRP:HD1	1:A:29:TRP:HA	4	0.76
(2,49)	1:A:5:VAL:HG21	1:A:7:SER:HA	5	0.75
(2,49)	1:A:5:VAL:HG22	1:A:7:SER:HA	5	0.75
(2,49)	1:A:5:VAL:HG23	1:A:7:SER:HA	5	0.75
(2,140)	1:A:26:PHE:HE1	1:A:54:LYS:HD2	7	0.75
(2,140)	1:A:26:PHE:HE1	1:A:54:LYS:HD3	7	0.75
(2,140)	1:A:26:PHE:HE2	1:A:54:LYS:HD2	7	0.75
(2,140)	1:A:26:PHE:HE2	1:A:54:LYS:HD3	7	0.75
(1,575)	1:A:20:LEU:HD11	1:A:77:VAL:HG11	9	0.75
(1,575)	1:A:20:LEU:HD11	1:A:77:VAL:HG12	9	0.75
(1,575)	1:A:20:LEU:HD11	1:A:77:VAL:HG13	9	0.75
(1,575)	1:A:20:LEU:HD12	1:A:77:VAL:HG11	9	0.75
(1,575)	1:A:20:LEU:HD12	1:A:77:VAL:HG12	9	0.75
(1,575)	1:A:20:LEU:HD12	1:A:77:VAL:HG13	9	0.75
(1,575)	1:A:20:LEU:HD13	1:A:77:VAL:HG11	9	0.75
(1,575)	1:A:20:LEU:HD13	1:A:77:VAL:HG12	9	0.75
(1,575)	1:A:20:LEU:HD13	1:A:77:VAL:HG13	9	0.75
(2,341)	1:A:72:MET:HA	1:A:72:MET:HE1	4	0.74
(2,341)	1:A:72:MET:HA	1:A:72:MET:HE2	4	0.74
(2,341)	1:A:72:MET:HA	1:A:72:MET:HE3	4	0.74
(1,990)	1:A:33:CYS:HA	1:A:90:ALA:HB1	9	0.74
(1,990)	1:A:33:CYS:HA	1:A:90:ALA:HB2	9	0.74

Continued on next page...

Continued from previous page...

Key	Atom-1	Atom-2	Model ID	Violation (Å)
(1,990)	1:A:33:CYS:HA	1:A:90:ALA:HB3	9	0.74
(1,2542)	1:A:27:ALA:HB1	1:A:27:ALA:HA	1	0.74
(1,2542)	1:A:27:ALA:HB2	1:A:27:ALA:HA	1	0.74
(1,2542)	1:A:27:ALA:HB3	1:A:27:ALA:HA	1	0.74
(1,2542)	1:A:27:ALA:HB1	1:A:27:ALA:HA	4	0.74
(1,2542)	1:A:27:ALA:HB2	1:A:27:ALA:HA	4	0.74
(1,2542)	1:A:27:ALA:HB3	1:A:27:ALA:HA	4	0.74
(1,2542)	1:A:27:ALA:HB1	1:A:27:ALA:HA	9	0.74
(1,2542)	1:A:27:ALA:HB2	1:A:27:ALA:HA	9	0.74
(1,2542)	1:A:27:ALA:HB3	1:A:27:ALA:HA	9	0.74
(1,1209)	1:A:40:TYR:HE1	1:A:95:LEU:HD11	4	0.74
(1,1209)	1:A:40:TYR:HE1	1:A:95:LEU:HD12	4	0.74
(1,1209)	1:A:40:TYR:HE1	1:A:95:LEU:HD13	4	0.74
(1,1209)	1:A:40:TYR:HE2	1:A:95:LEU:HD11	4	0.74
(1,1209)	1:A:40:TYR:HE2	1:A:95:LEU:HD12	4	0.74
(1,1209)	1:A:40:TYR:HE2	1:A:95:LEU:HD13	4	0.74
(2,328)	1:A:69:ILE:HG21	1:A:72:MET:HB2	6	0.73
(2,328)	1:A:69:ILE:HG22	1:A:72:MET:HB2	6	0.73
(2,328)	1:A:69:ILE:HG23	1:A:72:MET:HB2	6	0.73
(2,199)	1:A:36:ILE:HG21	1:A:40:TYR:HD1	6	0.73
(2,199)	1:A:36:ILE:HG21	1:A:40:TYR:HD2	6	0.73
(2,199)	1:A:36:ILE:HG22	1:A:40:TYR:HD1	6	0.73
(2,199)	1:A:36:ILE:HG22	1:A:40:TYR:HD2	6	0.73
(2,199)	1:A:36:ILE:HG23	1:A:40:TYR:HD1	6	0.73
(2,199)	1:A:36:ILE:HG23	1:A:40:TYR:HD2	6	0.73
(2,140)	1:A:26:PHE:HE1	1:A:54:LYS:HD2	10	0.73
(2,140)	1:A:26:PHE:HE1	1:A:54:LYS:HD3	10	0.73
(2,140)	1:A:26:PHE:HE2	1:A:54:LYS:HD2	10	0.73
(2,140)	1:A:26:PHE:HE2	1:A:54:LYS:HD3	10	0.73
(1,990)	1:A:33:CYS:HA	1:A:90:ALA:HB1	1	0.73
(1,990)	1:A:33:CYS:HA	1:A:90:ALA:HB2	1	0.73
(1,990)	1:A:33:CYS:HA	1:A:90:ALA:HB3	1	0.73
(1,2542)	1:A:27:ALA:HB1	1:A:27:ALA:HA	2	0.73
(1,2542)	1:A:27:ALA:HB2	1:A:27:ALA:HA	2	0.73
(1,2542)	1:A:27:ALA:HB3	1:A:27:ALA:HA	2	0.73
(1,2542)	1:A:27:ALA:HB1	1:A:27:ALA:HA	3	0.73
(1,2542)	1:A:27:ALA:HB2	1:A:27:ALA:HA	3	0.73
(1,2542)	1:A:27:ALA:HB3	1:A:27:ALA:HA	3	0.73
(1,2542)	1:A:27:ALA:HB1	1:A:27:ALA:HA	5	0.73
(1,2542)	1:A:27:ALA:HB2	1:A:27:ALA:HA	5	0.73
(1,2542)	1:A:27:ALA:HB3	1:A:27:ALA:HA	5	0.73
(1,2542)	1:A:27:ALA:HB1	1:A:27:ALA:HA	6	0.73

Continued on next page...

Continued from previous page...

Key	Atom-1	Atom-2	Model ID	Violation (Å)
(1,2542)	1:A:27:ALA:HB2	1:A:27:ALA:HA	6	0.73
(1,2542)	1:A:27:ALA:HB3	1:A:27:ALA:HA	6	0.73
(1,2542)	1:A:27:ALA:HB1	1:A:27:ALA:HA	7	0.73
(1,2542)	1:A:27:ALA:HB2	1:A:27:ALA:HA	7	0.73
(1,2542)	1:A:27:ALA:HB3	1:A:27:ALA:HA	7	0.73
(1,2542)	1:A:27:ALA:HB1	1:A:27:ALA:HA	8	0.73
(1,2542)	1:A:27:ALA:HB2	1:A:27:ALA:HA	8	0.73
(1,2542)	1:A:27:ALA:HB3	1:A:27:ALA:HA	8	0.73
(1,2542)	1:A:27:ALA:HB1	1:A:27:ALA:HA	10	0.73
(1,2542)	1:A:27:ALA:HB2	1:A:27:ALA:HA	10	0.73
(1,2542)	1:A:27:ALA:HB3	1:A:27:ALA:HA	10	0.73
(2,49)	1:A:5:VAL:HG21	1:A:7:SER:HA	8	0.72
(2,49)	1:A:5:VAL:HG22	1:A:7:SER:HA	8	0.72
(2,49)	1:A:5:VAL:HG23	1:A:7:SER:HA	8	0.72
(2,325)	1:A:69:ILE:HG21	1:A:71:SER:HA	10	0.72
(2,325)	1:A:69:ILE:HG22	1:A:71:SER:HA	10	0.72
(2,325)	1:A:69:ILE:HG23	1:A:71:SER:HA	10	0.72
(2,193)	1:A:29:TRP:HZ3	1:A:72:MET:HE1	7	0.72
(2,193)	1:A:29:TRP:HZ3	1:A:72:MET:HE2	7	0.72
(2,193)	1:A:29:TRP:HZ3	1:A:72:MET:HE3	7	0.72
(2,140)	1:A:26:PHE:HE1	1:A:54:LYS:HD2	5	0.72
(2,140)	1:A:26:PHE:HE1	1:A:54:LYS:HD3	5	0.72
(2,140)	1:A:26:PHE:HE2	1:A:54:LYS:HD2	5	0.72
(2,140)	1:A:26:PHE:HE2	1:A:54:LYS:HD3	5	0.72
(2,127)	1:A:25:PHE:HZ	1:A:69:ILE:HD11	7	0.72
(2,127)	1:A:25:PHE:HZ	1:A:69:ILE:HD12	7	0.72
(2,127)	1:A:25:PHE:HZ	1:A:69:ILE:HD13	7	0.72
(2,126)	1:A:25:PHE:HZ	1:A:69:ILE:HD11	7	0.72
(2,126)	1:A:25:PHE:HZ	1:A:69:ILE:HD12	7	0.72
(2,126)	1:A:25:PHE:HZ	1:A:69:ILE:HD13	7	0.72
(1,575)	1:A:20:LEU:HD11	1:A:77:VAL:HG11	3	0.72
(1,575)	1:A:20:LEU:HD11	1:A:77:VAL:HG12	3	0.72
(1,575)	1:A:20:LEU:HD11	1:A:77:VAL:HG13	3	0.72
(1,575)	1:A:20:LEU:HD12	1:A:77:VAL:HG11	3	0.72
(1,575)	1:A:20:LEU:HD12	1:A:77:VAL:HG12	3	0.72
(1,575)	1:A:20:LEU:HD12	1:A:77:VAL:HG13	3	0.72
(1,575)	1:A:20:LEU:HD13	1:A:77:VAL:HG11	3	0.72
(1,575)	1:A:20:LEU:HD13	1:A:77:VAL:HG12	3	0.72
(1,575)	1:A:20:LEU:HD13	1:A:77:VAL:HG13	3	0.72
(1,1209)	1:A:40:TYR:HE1	1:A:95:LEU:HD11	1	0.72
(1,1209)	1:A:40:TYR:HE1	1:A:95:LEU:HD12	1	0.72
(1,1209)	1:A:40:TYR:HE1	1:A:95:LEU:HD13	1	0.72

Continued on next page...

Continued from previous page...

Key	Atom-1	Atom-2	Model ID	Violation (Å)
(1,1209)	1:A:40:TYR:HE2	1:A:95:LEU:HD11	1	0.72
(1,1209)	1:A:40:TYR:HE2	1:A:95:LEU:HD12	1	0.72
(1,1209)	1:A:40:TYR:HE2	1:A:95:LEU:HD13	1	0.72
(2,341)	1:A:72:MET:HA	1:A:72:MET:HE1	8	0.71
(2,341)	1:A:72:MET:HA	1:A:72:MET:HE2	8	0.71
(2,341)	1:A:72:MET:HA	1:A:72:MET:HE3	8	0.71
(2,326)	1:A:69:ILE:HG21	1:A:72:MET:HA	3	0.71
(2,326)	1:A:69:ILE:HG22	1:A:72:MET:HA	3	0.71
(2,326)	1:A:69:ILE:HG23	1:A:72:MET:HA	3	0.71
(2,140)	1:A:26:PHE:HE1	1:A:54:LYS:HD2	9	0.71
(2,140)	1:A:26:PHE:HE1	1:A:54:LYS:HD3	9	0.71
(2,140)	1:A:26:PHE:HE2	1:A:54:LYS:HD2	9	0.71
(2,140)	1:A:26:PHE:HE2	1:A:54:LYS:HD3	9	0.71
(1,990)	1:A:33:CYS:HA	1:A:90:ALA:HB1	10	0.71
(1,990)	1:A:33:CYS:HA	1:A:90:ALA:HB2	10	0.71
(1,990)	1:A:33:CYS:HA	1:A:90:ALA:HB3	10	0.71
(1,1981)	1:A:77:VAL:HG21	1:A:102:TYR:HE1	7	0.71
(1,1981)	1:A:77:VAL:HG21	1:A:102:TYR:HE2	7	0.71
(1,1981)	1:A:77:VAL:HG22	1:A:102:TYR:HE1	7	0.71
(1,1981)	1:A:77:VAL:HG22	1:A:102:TYR:HE2	7	0.71
(1,1981)	1:A:77:VAL:HG23	1:A:102:TYR:HE1	7	0.71
(1,1981)	1:A:77:VAL:HG23	1:A:102:TYR:HE2	7	0.71
(1,1981)	1:A:77:VAL:HG21	1:A:102:TYR:HE1	10	0.71
(1,1981)	1:A:77:VAL:HG21	1:A:102:TYR:HE2	10	0.71
(1,1981)	1:A:77:VAL:HG22	1:A:102:TYR:HE1	10	0.71
(1,1981)	1:A:77:VAL:HG22	1:A:102:TYR:HE2	10	0.71
(1,1981)	1:A:77:VAL:HG23	1:A:102:TYR:HE1	10	0.71
(1,1981)	1:A:77:VAL:HG23	1:A:102:TYR:HE2	10	0.71
(1,1209)	1:A:40:TYR:HE1	1:A:95:LEU:HD11	2	0.71
(1,1209)	1:A:40:TYR:HE1	1:A:95:LEU:HD12	2	0.71
(1,1209)	1:A:40:TYR:HE1	1:A:95:LEU:HD13	2	0.71
(1,1209)	1:A:40:TYR:HE2	1:A:95:LEU:HD11	2	0.71
(1,1209)	1:A:40:TYR:HE2	1:A:95:LEU:HD12	2	0.71
(1,1209)	1:A:40:TYR:HE2	1:A:95:LEU:HD13	2	0.71
(1,1209)	1:A:40:TYR:HE1	1:A:95:LEU:HD11	6	0.71
(1,1209)	1:A:40:TYR:HE1	1:A:95:LEU:HD12	6	0.71
(1,1209)	1:A:40:TYR:HE1	1:A:95:LEU:HD13	6	0.71
(1,1209)	1:A:40:TYR:HE2	1:A:95:LEU:HD11	6	0.71
(1,1209)	1:A:40:TYR:HE2	1:A:95:LEU:HD12	6	0.71
(1,1209)	1:A:40:TYR:HE2	1:A:95:LEU:HD13	6	0.71
(1,1209)	1:A:40:TYR:HE1	1:A:95:LEU:HD11	8	0.71
(1,1209)	1:A:40:TYR:HE1	1:A:95:LEU:HD12	8	0.71

Continued on next page...

Continued from previous page...

Key	Atom-1	Atom-2	Model ID	Violation (Å)
(1,1209)	1:A:40:TYR:HE1	1:A:95:LEU:HD13	8	0.71
(1,1209)	1:A:40:TYR:HE2	1:A:95:LEU:HD11	8	0.71
(1,1209)	1:A:40:TYR:HE2	1:A:95:LEU:HD12	8	0.71
(1,1209)	1:A:40:TYR:HE2	1:A:95:LEU:HD13	8	0.71
(1,990)	1:A:33:CYS:HA	1:A:90:ALA:HB1	3	0.7
(1,990)	1:A:33:CYS:HA	1:A:90:ALA:HB2	3	0.7
(1,990)	1:A:33:CYS:HA	1:A:90:ALA:HB3	3	0.7
(1,990)	1:A:33:CYS:HA	1:A:90:ALA:HB1	4	0.7
(1,990)	1:A:33:CYS:HA	1:A:90:ALA:HB2	4	0.7
(1,990)	1:A:33:CYS:HA	1:A:90:ALA:HB3	4	0.7
(1,990)	1:A:33:CYS:HA	1:A:90:ALA:HB1	7	0.7
(1,990)	1:A:33:CYS:HA	1:A:90:ALA:HB2	7	0.7
(1,990)	1:A:33:CYS:HA	1:A:90:ALA:HB3	7	0.7
(1,390)	1:A:15:ILE:HD11	1:A:78:TYR:HE1	3	0.7
(1,390)	1:A:15:ILE:HD11	1:A:78:TYR:HE2	3	0.7
(1,390)	1:A:15:ILE:HD12	1:A:78:TYR:HE1	3	0.7
(1,390)	1:A:15:ILE:HD12	1:A:78:TYR:HE2	3	0.7
(1,390)	1:A:15:ILE:HD13	1:A:78:TYR:HE1	3	0.7
(1,390)	1:A:15:ILE:HD13	1:A:78:TYR:HE2	3	0.7
(1,2546)	1:A:29:TRP:HD1	1:A:29:TRP:HA	2	0.7
(1,2546)	1:A:29:TRP:HD1	1:A:29:TRP:HA	3	0.7
(1,2546)	1:A:29:TRP:HD1	1:A:29:TRP:HA	5	0.7
(1,2546)	1:A:29:TRP:HD1	1:A:29:TRP:HA	6	0.7
(1,1981)	1:A:77:VAL:HG21	1:A:102:TYR:HE1	3	0.7
(1,1981)	1:A:77:VAL:HG21	1:A:102:TYR:HE2	3	0.7
(1,1981)	1:A:77:VAL:HG22	1:A:102:TYR:HE1	3	0.7
(1,1981)	1:A:77:VAL:HG22	1:A:102:TYR:HE2	3	0.7
(1,1981)	1:A:77:VAL:HG23	1:A:102:TYR:HE1	3	0.7
(1,1981)	1:A:77:VAL:HG23	1:A:102:TYR:HE2	3	0.7
(1,1209)	1:A:40:TYR:HE1	1:A:95:LEU:HD11	9	0.7
(1,1209)	1:A:40:TYR:HE1	1:A:95:LEU:HD12	9	0.7
(1,1209)	1:A:40:TYR:HE1	1:A:95:LEU:HD13	9	0.7
(1,1209)	1:A:40:TYR:HE2	1:A:95:LEU:HD11	9	0.7
(1,1209)	1:A:40:TYR:HE2	1:A:95:LEU:HD12	9	0.7
(1,1209)	1:A:40:TYR:HE2	1:A:95:LEU:HD13	9	0.7
(1,1209)	1:A:40:TYR:HE1	1:A:95:LEU:HD11	10	0.7
(1,1209)	1:A:40:TYR:HE1	1:A:95:LEU:HD12	10	0.7
(1,1209)	1:A:40:TYR:HE1	1:A:95:LEU:HD13	10	0.7
(1,1209)	1:A:40:TYR:HE2	1:A:95:LEU:HD11	10	0.7
(1,1209)	1:A:40:TYR:HE2	1:A:95:LEU:HD12	10	0.7
(1,1209)	1:A:40:TYR:HE2	1:A:95:LEU:HD13	10	0.7
(2,118)	1:A:25:PHE:HD1	1:A:67:GLU:HG2	8	0.69

Continued on next page...

Continued from previous page...

Key	Atom-1	Atom-2	Model ID	Violation (Å)
(2,118)	1:A:25:PHE:HD1	1:A:67:GLU:HG3	8	0.69
(2,118)	1:A:25:PHE:HD2	1:A:67:GLU:HG2	8	0.69
(2,118)	1:A:25:PHE:HD2	1:A:67:GLU:HG3	8	0.69
(1,990)	1:A:33:CYS:HA	1:A:90:ALA:HB1	8	0.69
(1,990)	1:A:33:CYS:HA	1:A:90:ALA:HB2	8	0.69
(1,990)	1:A:33:CYS:HA	1:A:90:ALA:HB3	8	0.69
(1,575)	1:A:20:LEU:HD11	1:A:77:VAL:HG11	10	0.69
(1,575)	1:A:20:LEU:HD11	1:A:77:VAL:HG12	10	0.69
(1,575)	1:A:20:LEU:HD11	1:A:77:VAL:HG13	10	0.69
(1,575)	1:A:20:LEU:HD12	1:A:77:VAL:HG11	10	0.69
(1,575)	1:A:20:LEU:HD12	1:A:77:VAL:HG12	10	0.69
(1,575)	1:A:20:LEU:HD12	1:A:77:VAL:HG13	10	0.69
(1,575)	1:A:20:LEU:HD13	1:A:77:VAL:HG11	10	0.69
(1,575)	1:A:20:LEU:HD13	1:A:77:VAL:HG12	10	0.69
(1,575)	1:A:20:LEU:HD13	1:A:77:VAL:HG13	10	0.69
(1,2546)	1:A:29:TRP:HD1	1:A:29:TRP:HA	8	0.69
(1,2546)	1:A:29:TRP:HD1	1:A:29:TRP:HA	10	0.69
(1,2127)	1:A:84:VAL:HG21	1:A:102:TYR:HE1	1	0.69
(1,2127)	1:A:84:VAL:HG21	1:A:102:TYR:HE2	1	0.69
(1,2127)	1:A:84:VAL:HG22	1:A:102:TYR:HE1	1	0.69
(1,2127)	1:A:84:VAL:HG22	1:A:102:TYR:HE2	1	0.69
(1,2127)	1:A:84:VAL:HG23	1:A:102:TYR:HE1	1	0.69
(1,2127)	1:A:84:VAL:HG23	1:A:102:TYR:HE2	1	0.69
(2,328)	1:A:69:ILE:HG21	1:A:72:MET:HB2	4	0.68
(2,328)	1:A:69:ILE:HG22	1:A:72:MET:HB2	4	0.68
(2,328)	1:A:69:ILE:HG23	1:A:72:MET:HB2	4	0.68
(2,127)	1:A:25:PHE:HZ	1:A:69:ILE:HD11	2	0.68
(2,127)	1:A:25:PHE:HZ	1:A:69:ILE:HD12	2	0.68
(2,127)	1:A:25:PHE:HZ	1:A:69:ILE:HD13	2	0.68
(2,127)	1:A:25:PHE:HZ	1:A:69:ILE:HD11	4	0.68
(2,127)	1:A:25:PHE:HZ	1:A:69:ILE:HD12	4	0.68
(2,127)	1:A:25:PHE:HZ	1:A:69:ILE:HD13	4	0.68
(2,126)	1:A:25:PHE:HZ	1:A:69:ILE:HD11	2	0.68
(2,126)	1:A:25:PHE:HZ	1:A:69:ILE:HD12	2	0.68
(2,126)	1:A:25:PHE:HZ	1:A:69:ILE:HD13	2	0.68
(2,126)	1:A:25:PHE:HZ	1:A:69:ILE:HD11	4	0.68
(2,126)	1:A:25:PHE:HZ	1:A:69:ILE:HD12	4	0.68
(2,126)	1:A:25:PHE:HZ	1:A:69:ILE:HD13	4	0.68
(1,990)	1:A:33:CYS:HA	1:A:90:ALA:HB1	6	0.68
(1,990)	1:A:33:CYS:HA	1:A:90:ALA:HB2	6	0.68
(1,990)	1:A:33:CYS:HA	1:A:90:ALA:HB3	6	0.68
(1,1392)	1:A:47:TYR:HE1	1:A:99:ILE:HG21	10	0.68

Continued on next page...

Continued from previous page...

Key	Atom-1	Atom-2	Model ID	Violation (Å)
(1,1392)	1:A:47:TYR:HE1	1:A:99:ILE:HG22	10	0.68
(1,1392)	1:A:47:TYR:HE1	1:A:99:ILE:HG23	10	0.68
(1,1392)	1:A:47:TYR:HE2	1:A:99:ILE:HG21	10	0.68
(1,1392)	1:A:47:TYR:HE2	1:A:99:ILE:HG22	10	0.68
(1,1392)	1:A:47:TYR:HE2	1:A:99:ILE:HG23	10	0.68
(2,341)	1:A:72:MET:HA	1:A:72:MET:HE1	5	0.67
(2,341)	1:A:72:MET:HA	1:A:72:MET:HE2	5	0.67
(2,341)	1:A:72:MET:HA	1:A:72:MET:HE3	5	0.67
(2,127)	1:A:25:PHE:HZ	1:A:69:ILE:HD11	9	0.67
(2,127)	1:A:25:PHE:HZ	1:A:69:ILE:HD12	9	0.67
(2,127)	1:A:25:PHE:HZ	1:A:69:ILE:HD13	9	0.67
(2,126)	1:A:25:PHE:HZ	1:A:69:ILE:HD11	9	0.67
(2,126)	1:A:25:PHE:HZ	1:A:69:ILE:HD12	9	0.67
(2,126)	1:A:25:PHE:HZ	1:A:69:ILE:HD13	9	0.67
(1,2546)	1:A:29:TRP:HD1	1:A:29:TRP:HA	7	0.67
(1,1392)	1:A:47:TYR:HE1	1:A:99:ILE:HG21	1	0.67
(1,1392)	1:A:47:TYR:HE1	1:A:99:ILE:HG22	1	0.67
(1,1392)	1:A:47:TYR:HE1	1:A:99:ILE:HG23	1	0.67
(1,1392)	1:A:47:TYR:HE2	1:A:99:ILE:HG21	1	0.67
(1,1392)	1:A:47:TYR:HE2	1:A:99:ILE:HG22	1	0.67
(1,1392)	1:A:47:TYR:HE2	1:A:99:ILE:HG23	1	0.67
(1,1392)	1:A:47:TYR:HE1	1:A:99:ILE:HG21	3	0.67
(1,1392)	1:A:47:TYR:HE1	1:A:99:ILE:HG22	3	0.67
(1,1392)	1:A:47:TYR:HE1	1:A:99:ILE:HG23	3	0.67
(1,1392)	1:A:47:TYR:HE2	1:A:99:ILE:HG21	3	0.67
(1,1392)	1:A:47:TYR:HE2	1:A:99:ILE:HG22	3	0.67
(1,1392)	1:A:47:TYR:HE2	1:A:99:ILE:HG23	3	0.67
(1,1392)	1:A:47:TYR:HE1	1:A:99:ILE:HG21	7	0.67
(1,1392)	1:A:47:TYR:HE1	1:A:99:ILE:HG22	7	0.67
(1,1392)	1:A:47:TYR:HE1	1:A:99:ILE:HG23	7	0.67
(1,1392)	1:A:47:TYR:HE2	1:A:99:ILE:HG21	7	0.67
(1,1392)	1:A:47:TYR:HE2	1:A:99:ILE:HG22	7	0.67
(1,1392)	1:A:47:TYR:HE2	1:A:99:ILE:HG23	7	0.67
(2,267)	1:A:51:VAL:H	1:A:52:PHE:HD1	3	0.66
(2,267)	1:A:51:VAL:H	1:A:52:PHE:HD2	3	0.66
(2,267)	1:A:51:VAL:H	1:A:52:PHE:HD1	7	0.66
(2,267)	1:A:51:VAL:H	1:A:52:PHE:HD2	7	0.66
(2,257)	1:A:48:THR:HA	1:A:52:PHE:HD1	2	0.66
(2,257)	1:A:48:THR:HA	1:A:52:PHE:HD2	2	0.66
(2,257)	1:A:48:THR:HA	1:A:52:PHE:HD1	5	0.66
(2,257)	1:A:48:THR:HA	1:A:52:PHE:HD2	5	0.66
(2,257)	1:A:48:THR:HA	1:A:52:PHE:HD1	6	0.66

Continued on next page...

Continued from previous page...

Key	Atom-1	Atom-2	Model ID	Violation (Å)
(2,257)	1:A:48:THR:HA	1:A:52:PHE:HD2	6	0.66
(2,257)	1:A:48:THR:HA	1:A:52:PHE:HD1	10	0.66
(2,257)	1:A:48:THR:HA	1:A:52:PHE:HD2	10	0.66
(2,245)	1:A:47:TYR:HB3	1:A:50:MET:HE1	8	0.66
(2,245)	1:A:47:TYR:HB3	1:A:50:MET:HE2	8	0.66
(2,245)	1:A:47:TYR:HB3	1:A:50:MET:HE3	8	0.66
(2,118)	1:A:25:PHE:HD1	1:A:67:GLU:HG2	9	0.66
(2,118)	1:A:25:PHE:HD1	1:A:67:GLU:HG3	9	0.66
(2,118)	1:A:25:PHE:HD2	1:A:67:GLU:HG2	9	0.66
(2,118)	1:A:25:PHE:HD2	1:A:67:GLU:HG3	9	0.66
(1,990)	1:A:33:CYS:HA	1:A:90:ALA:HB1	2	0.66
(1,990)	1:A:33:CYS:HA	1:A:90:ALA:HB2	2	0.66
(1,990)	1:A:33:CYS:HA	1:A:90:ALA:HB3	2	0.66
(1,390)	1:A:15:ILE:HD11	1:A:78:TYR:HE1	1	0.66
(1,390)	1:A:15:ILE:HD11	1:A:78:TYR:HE2	1	0.66
(1,390)	1:A:15:ILE:HD12	1:A:78:TYR:HE1	1	0.66
(1,390)	1:A:15:ILE:HD12	1:A:78:TYR:HE2	1	0.66
(1,390)	1:A:15:ILE:HD13	1:A:78:TYR:HE1	1	0.66
(1,390)	1:A:15:ILE:HD13	1:A:78:TYR:HE2	1	0.66
(1,1392)	1:A:47:TYR:HE1	1:A:99:ILE:HG21	6	0.66
(1,1392)	1:A:47:TYR:HE1	1:A:99:ILE:HG22	6	0.66
(1,1392)	1:A:47:TYR:HE1	1:A:99:ILE:HG23	6	0.66
(1,1392)	1:A:47:TYR:HE2	1:A:99:ILE:HG21	6	0.66
(1,1392)	1:A:47:TYR:HE2	1:A:99:ILE:HG22	6	0.66
(1,1392)	1:A:47:TYR:HE2	1:A:99:ILE:HG23	6	0.66
(2,325)	1:A:69:ILE:HG21	1:A:71:SER:HA	4	0.65
(2,325)	1:A:69:ILE:HG22	1:A:71:SER:HA	4	0.65
(2,325)	1:A:69:ILE:HG23	1:A:71:SER:HA	4	0.65
(2,257)	1:A:48:THR:HA	1:A:52:PHE:HD1	3	0.65
(2,257)	1:A:48:THR:HA	1:A:52:PHE:HD2	3	0.65
(2,257)	1:A:48:THR:HA	1:A:52:PHE:HD1	4	0.65
(2,257)	1:A:48:THR:HA	1:A:52:PHE:HD2	4	0.65
(2,257)	1:A:48:THR:HA	1:A:52:PHE:HD1	7	0.65
(2,257)	1:A:48:THR:HA	1:A:52:PHE:HD2	7	0.65
(2,210)	1:A:40:TYR:HE1	1:A:52:PHE:HB3	3	0.65
(2,210)	1:A:40:TYR:HE2	1:A:52:PHE:HB3	3	0.65
(1,1392)	1:A:47:TYR:HE1	1:A:99:ILE:HG21	9	0.65
(1,1392)	1:A:47:TYR:HE1	1:A:99:ILE:HG22	9	0.65
(1,1392)	1:A:47:TYR:HE1	1:A:99:ILE:HG23	9	0.65
(1,1392)	1:A:47:TYR:HE2	1:A:99:ILE:HG21	9	0.65
(1,1392)	1:A:47:TYR:HE2	1:A:99:ILE:HG22	9	0.65
(1,1392)	1:A:47:TYR:HE2	1:A:99:ILE:HG23	9	0.65

Continued on next page...

Continued from previous page...

Key	Atom-1	Atom-2	Model ID	Violation (Å)
(2,341)	1:A:72:MET:HA	1:A:72:MET:HE1	1	0.64
(2,341)	1:A:72:MET:HA	1:A:72:MET:HE2	1	0.64
(2,341)	1:A:72:MET:HA	1:A:72:MET:HE3	1	0.64
(2,267)	1:A:51:VAL:H	1:A:52:PHE:HD1	10	0.64
(2,267)	1:A:51:VAL:H	1:A:52:PHE:HD2	10	0.64
(1,389)	1:A:15:ILE:HD11	1:A:78:TYR:HD1	1	0.64
(1,389)	1:A:15:ILE:HD11	1:A:78:TYR:HD2	1	0.64
(1,389)	1:A:15:ILE:HD12	1:A:78:TYR:HD1	1	0.64
(1,389)	1:A:15:ILE:HD12	1:A:78:TYR:HD2	1	0.64
(1,389)	1:A:15:ILE:HD13	1:A:78:TYR:HD1	1	0.64
(1,389)	1:A:15:ILE:HD13	1:A:78:TYR:HD2	1	0.64
(1,1209)	1:A:40:TYR:HE1	1:A:95:LEU:HD11	3	0.64
(1,1209)	1:A:40:TYR:HE1	1:A:95:LEU:HD12	3	0.64
(1,1209)	1:A:40:TYR:HE1	1:A:95:LEU:HD13	3	0.64
(1,1209)	1:A:40:TYR:HE2	1:A:95:LEU:HD11	3	0.64
(1,1209)	1:A:40:TYR:HE2	1:A:95:LEU:HD12	3	0.64
(1,1209)	1:A:40:TYR:HE2	1:A:95:LEU:HD13	3	0.64
(2,267)	1:A:51:VAL:H	1:A:52:PHE:HD1	2	0.63
(2,267)	1:A:51:VAL:H	1:A:52:PHE:HD2	2	0.63
(2,267)	1:A:51:VAL:H	1:A:52:PHE:HD1	5	0.63
(2,267)	1:A:51:VAL:H	1:A:52:PHE:HD2	5	0.63
(2,267)	1:A:51:VAL:H	1:A:52:PHE:HD1	6	0.63
(2,267)	1:A:51:VAL:H	1:A:52:PHE:HD2	6	0.63
(2,257)	1:A:48:THR:HA	1:A:52:PHE:HD1	8	0.63
(2,257)	1:A:48:THR:HA	1:A:52:PHE:HD2	8	0.63
(2,210)	1:A:40:TYR:HE1	1:A:52:PHE:HB3	9	0.63
(2,210)	1:A:40:TYR:HE2	1:A:52:PHE:HB3	9	0.63
(1,389)	1:A:15:ILE:HD11	1:A:78:TYR:HD1	6	0.63
(1,389)	1:A:15:ILE:HD11	1:A:78:TYR:HD2	6	0.63
(1,389)	1:A:15:ILE:HD12	1:A:78:TYR:HD1	6	0.63
(1,389)	1:A:15:ILE:HD12	1:A:78:TYR:HD2	6	0.63
(1,389)	1:A:15:ILE:HD13	1:A:78:TYR:HD1	6	0.63
(1,389)	1:A:15:ILE:HD13	1:A:78:TYR:HD2	6	0.63
(1,1392)	1:A:47:TYR:HE1	1:A:99:ILE:HG21	8	0.63
(1,1392)	1:A:47:TYR:HE1	1:A:99:ILE:HG22	8	0.63
(1,1392)	1:A:47:TYR:HE1	1:A:99:ILE:HG23	8	0.63
(1,1392)	1:A:47:TYR:HE2	1:A:99:ILE:HG21	8	0.63
(1,1392)	1:A:47:TYR:HE2	1:A:99:ILE:HG22	8	0.63
(1,1392)	1:A:47:TYR:HE2	1:A:99:ILE:HG23	8	0.63
(2,267)	1:A:51:VAL:H	1:A:52:PHE:HD1	4	0.62
(2,267)	1:A:51:VAL:H	1:A:52:PHE:HD2	4	0.62
(2,257)	1:A:48:THR:HA	1:A:52:PHE:HD1	1	0.62

Continued on next page...

Continued from previous page...

Key	Atom-1	Atom-2	Model ID	Violation (Å)
(2,257)	1:A:48:THR:HA	1:A:52:PHE:HD2	1	0.62
(2,257)	1:A:48:THR:HA	1:A:52:PHE:HD1	9	0.62
(2,257)	1:A:48:THR:HA	1:A:52:PHE:HD2	9	0.62
(2,210)	1:A:40:TYR:HE1	1:A:52:PHE:HB3	4	0.62
(2,210)	1:A:40:TYR:HE2	1:A:52:PHE:HB3	4	0.62
(2,210)	1:A:40:TYR:HE1	1:A:52:PHE:HB3	5	0.62
(2,210)	1:A:40:TYR:HE2	1:A:52:PHE:HB3	5	0.62
(2,137)	1:A:26:PHE:HB2	1:A:72:MET:HE1	3	0.62
(2,137)	1:A:26:PHE:HB2	1:A:72:MET:HE2	3	0.62
(2,137)	1:A:26:PHE:HB2	1:A:72:MET:HE3	3	0.62
(2,10)	1:A:1:SER:HB2	1:A:3:LYS:H	3	0.62
(2,10)	1:A:1:SER:HB3	1:A:3:LYS:H	3	0.62
(1,576)	1:A:20:LEU:HD11	1:A:78:TYR:H	9	0.62
(1,576)	1:A:20:LEU:HD12	1:A:78:TYR:H	9	0.62
(1,576)	1:A:20:LEU:HD13	1:A:78:TYR:H	9	0.62
(2,267)	1:A:51:VAL:H	1:A:52:PHE:HD1	8	0.61
(2,267)	1:A:51:VAL:H	1:A:52:PHE:HD2	8	0.61
(2,264)	1:A:50:MET:HE1	1:A:99:ILE:HA	8	0.61
(2,264)	1:A:50:MET:HE2	1:A:99:ILE:HA	8	0.61
(2,264)	1:A:50:MET:HE3	1:A:99:ILE:HA	8	0.61
(2,210)	1:A:40:TYR:HE1	1:A:52:PHE:HB3	1	0.61
(2,210)	1:A:40:TYR:HE2	1:A:52:PHE:HB3	1	0.61
(2,210)	1:A:40:TYR:HE1	1:A:52:PHE:HB3	8	0.61
(2,210)	1:A:40:TYR:HE2	1:A:52:PHE:HB3	8	0.61
(1,576)	1:A:20:LEU:HD11	1:A:78:TYR:H	1	0.61
(1,576)	1:A:20:LEU:HD12	1:A:78:TYR:H	1	0.61
(1,576)	1:A:20:LEU:HD13	1:A:78:TYR:H	1	0.61
(1,2423)	1:A:99:ILE:HG21	1:A:100:GLU:H	5	0.61
(1,2423)	1:A:99:ILE:HG22	1:A:100:GLU:H	5	0.61
(1,2423)	1:A:99:ILE:HG23	1:A:100:GLU:H	5	0.61
(2,267)	1:A:51:VAL:H	1:A:52:PHE:HD1	9	0.6
(2,267)	1:A:51:VAL:H	1:A:52:PHE:HD2	9	0.6
(2,210)	1:A:40:TYR:HE1	1:A:52:PHE:HB3	2	0.6
(2,210)	1:A:40:TYR:HE2	1:A:52:PHE:HB3	2	0.6
(2,210)	1:A:40:TYR:HE1	1:A:52:PHE:HB3	10	0.6
(2,210)	1:A:40:TYR:HE2	1:A:52:PHE:HB3	10	0.6
(2,140)	1:A:26:PHE:HE1	1:A:54:LYS:HD2	6	0.6
(2,140)	1:A:26:PHE:HE1	1:A:54:LYS:HD3	6	0.6
(2,140)	1:A:26:PHE:HE2	1:A:54:LYS:HD2	6	0.6
(2,140)	1:A:26:PHE:HE2	1:A:54:LYS:HD3	6	0.6
(2,80)	1:A:12:ASP:HB3	1:A:66:LYS:HE2	7	0.59
(2,80)	1:A:12:ASP:HB3	1:A:66:LYS:HE3	7	0.59

Continued on next page...

Continued from previous page...

Key	Atom-1	Atom-2	Model ID	Violation (Å)
(2,32)	1:A:3:LYS:HD2	1:A:53:ILE:HA	2	0.59
(2,32)	1:A:3:LYS:HD3	1:A:53:ILE:HA	2	0.59
(2,32)	1:A:3:LYS:HD2	1:A:53:ILE:HA	4	0.59
(2,32)	1:A:3:LYS:HD3	1:A:53:ILE:HA	4	0.59
(2,32)	1:A:3:LYS:HD2	1:A:53:ILE:HA	10	0.59
(2,32)	1:A:3:LYS:HD3	1:A:53:ILE:HA	10	0.59
(2,267)	1:A:51:VAL:H	1:A:52:PHE:HD1	1	0.59
(2,267)	1:A:51:VAL:H	1:A:52:PHE:HD2	1	0.59
(2,210)	1:A:40:TYR:HE1	1:A:52:PHE:HB3	6	0.59
(2,210)	1:A:40:TYR:HE2	1:A:52:PHE:HB3	6	0.59
(1,575)	1:A:20:LEU:HD11	1:A:77:VAL:HG11	4	0.59
(1,575)	1:A:20:LEU:HD11	1:A:77:VAL:HG12	4	0.59
(1,575)	1:A:20:LEU:HD11	1:A:77:VAL:HG13	4	0.59
(1,575)	1:A:20:LEU:HD12	1:A:77:VAL:HG11	4	0.59
(1,575)	1:A:20:LEU:HD12	1:A:77:VAL:HG12	4	0.59
(1,575)	1:A:20:LEU:HD12	1:A:77:VAL:HG13	4	0.59
(1,575)	1:A:20:LEU:HD13	1:A:77:VAL:HG11	4	0.59
(1,575)	1:A:20:LEU:HD13	1:A:77:VAL:HG12	4	0.59
(1,575)	1:A:20:LEU:HD13	1:A:77:VAL:HG13	4	0.59
(1,2423)	1:A:99:ILE:HG21	1:A:100:GLU:H	7	0.59
(1,2423)	1:A:99:ILE:HG22	1:A:100:GLU:H	7	0.59
(1,2423)	1:A:99:ILE:HG23	1:A:100:GLU:H	7	0.59
(1,2423)	1:A:99:ILE:HG21	1:A:100:GLU:H	8	0.59
(1,2423)	1:A:99:ILE:HG22	1:A:100:GLU:H	8	0.59
(1,2423)	1:A:99:ILE:HG23	1:A:100:GLU:H	8	0.59
(1,1719)	1:A:68:ASN:HD21	1:A:70:THR:HG21	4	0.59
(1,1719)	1:A:68:ASN:HD21	1:A:70:THR:HG22	4	0.59
(1,1719)	1:A:68:ASN:HD21	1:A:70:THR:HG23	4	0.59
(2,74)	1:A:11:PHE:HD1	1:A:12:ASP:HB2	2	0.58
(2,74)	1:A:11:PHE:HD2	1:A:12:ASP:HB2	2	0.58
(2,74)	1:A:11:PHE:HD1	1:A:12:ASP:HB2	4	0.58
(2,74)	1:A:11:PHE:HD2	1:A:12:ASP:HB2	4	0.58
(2,74)	1:A:11:PHE:HD1	1:A:12:ASP:HB2	6	0.58
(2,74)	1:A:11:PHE:HD2	1:A:12:ASP:HB2	6	0.58
(2,74)	1:A:11:PHE:HD1	1:A:12:ASP:HB2	7	0.58
(2,74)	1:A:11:PHE:HD2	1:A:12:ASP:HB2	7	0.58
(2,32)	1:A:3:LYS:HD2	1:A:53:ILE:HA	1	0.58
(2,32)	1:A:3:LYS:HD3	1:A:53:ILE:HA	1	0.58
(2,32)	1:A:3:LYS:HD2	1:A:53:ILE:HA	6	0.58
(2,32)	1:A:3:LYS:HD3	1:A:53:ILE:HA	6	0.58
(2,32)	1:A:3:LYS:HD2	1:A:53:ILE:HA	7	0.58
(2,32)	1:A:3:LYS:HD3	1:A:53:ILE:HA	7	0.58

Continued on next page...

Continued from previous page...

Key	Atom-1	Atom-2	Model ID	Violation (Å)
(2,210)	1:A:40:TYR:HE1	1:A:52:PHE:HB3	7	0.58
(2,210)	1:A:40:TYR:HE2	1:A:52:PHE:HB3	7	0.58
(2,199)	1:A:36:ILE:HG21	1:A:40:TYR:HD1	3	0.58
(2,199)	1:A:36:ILE:HG21	1:A:40:TYR:HD2	3	0.58
(2,199)	1:A:36:ILE:HG22	1:A:40:TYR:HD1	3	0.58
(2,199)	1:A:36:ILE:HG22	1:A:40:TYR:HD2	3	0.58
(2,199)	1:A:36:ILE:HG23	1:A:40:TYR:HD1	3	0.58
(2,199)	1:A:36:ILE:HG23	1:A:40:TYR:HD2	3	0.58
(2,10)	1:A:1:SER:HB2	1:A:3:LYS:H	9	0.58
(2,10)	1:A:1:SER:HB3	1:A:3:LYS:H	9	0.58
(1,606)	1:A:21:VAL:HA	1:A:51:VAL:HG11	6	0.58
(1,606)	1:A:21:VAL:HA	1:A:51:VAL:HG12	6	0.58
(1,606)	1:A:21:VAL:HA	1:A:51:VAL:HG13	6	0.58
(1,606)	1:A:21:VAL:HA	1:A:51:VAL:HG11	9	0.58
(1,606)	1:A:21:VAL:HA	1:A:51:VAL:HG12	9	0.58
(1,606)	1:A:21:VAL:HA	1:A:51:VAL:HG13	9	0.58
(1,2423)	1:A:99:ILE:HG21	1:A:100:GLU:H	4	0.58
(1,2423)	1:A:99:ILE:HG22	1:A:100:GLU:H	4	0.58
(1,2423)	1:A:99:ILE:HG23	1:A:100:GLU:H	4	0.58
(1,2423)	1:A:99:ILE:HG21	1:A:100:GLU:H	10	0.58
(1,2423)	1:A:99:ILE:HG22	1:A:100:GLU:H	10	0.58
(1,2423)	1:A:99:ILE:HG23	1:A:100:GLU:H	10	0.58
(1,1950)	1:A:77:VAL:HG11	1:A:84:VAL:HG11	9	0.58
(1,1950)	1:A:77:VAL:HG11	1:A:84:VAL:HG12	9	0.58
(1,1950)	1:A:77:VAL:HG11	1:A:84:VAL:HG13	9	0.58
(1,1950)	1:A:77:VAL:HG12	1:A:84:VAL:HG11	9	0.58
(1,1950)	1:A:77:VAL:HG12	1:A:84:VAL:HG12	9	0.58
(1,1950)	1:A:77:VAL:HG12	1:A:84:VAL:HG13	9	0.58
(1,1950)	1:A:77:VAL:HG13	1:A:84:VAL:HG11	9	0.58
(1,1950)	1:A:77:VAL:HG13	1:A:84:VAL:HG12	9	0.58
(1,1950)	1:A:77:VAL:HG13	1:A:84:VAL:HG13	9	0.58
(2,74)	1:A:11:PHE:HD1	1:A:12:ASP:HB2	1	0.57
(2,74)	1:A:11:PHE:HD2	1:A:12:ASP:HB2	1	0.57
(2,74)	1:A:11:PHE:HD1	1:A:12:ASP:HB2	10	0.57
(2,74)	1:A:11:PHE:HD2	1:A:12:ASP:HB2	10	0.57
(2,325)	1:A:69:ILE:HG21	1:A:71:SER:HA	2	0.57
(2,325)	1:A:69:ILE:HG22	1:A:71:SER:HA	2	0.57
(2,325)	1:A:69:ILE:HG23	1:A:71:SER:HA	2	0.57
(2,32)	1:A:3:LYS:HD2	1:A:53:ILE:HA	8	0.57
(2,32)	1:A:3:LYS:HD3	1:A:53:ILE:HA	8	0.57
(2,296)	1:A:67:GLU:HB3	1:A:78:TYR:HE1	6	0.57
(2,296)	1:A:67:GLU:HB3	1:A:78:TYR:HE2	6	0.57

Continued on next page...

Continued from previous page...

Key	Atom-1	Atom-2	Model ID	Violation (Å)
(2,266)	1:A:50:MET:H	1:A:52:PHE:HD1	3	0.57
(2,266)	1:A:50:MET:H	1:A:52:PHE:HD2	3	0.57
(2,238)	1:A:46:THR:HG21	1:A:104:ALA:HB1	1	0.57
(2,238)	1:A:46:THR:HG21	1:A:104:ALA:HB2	1	0.57
(2,238)	1:A:46:THR:HG21	1:A:104:ALA:HB3	1	0.57
(2,238)	1:A:46:THR:HG22	1:A:104:ALA:HB1	1	0.57
(2,238)	1:A:46:THR:HG22	1:A:104:ALA:HB2	1	0.57
(2,238)	1:A:46:THR:HG22	1:A:104:ALA:HB3	1	0.57
(2,238)	1:A:46:THR:HG23	1:A:104:ALA:HB1	1	0.57
(2,238)	1:A:46:THR:HG23	1:A:104:ALA:HB2	1	0.57
(2,238)	1:A:46:THR:HG23	1:A:104:ALA:HB3	1	0.57
(2,230)	1:A:44:SER:HG	1:A:45:LYS:HE2	2	0.57
(2,230)	1:A:44:SER:HG	1:A:45:LYS:HE3	2	0.57
(2,224)	1:A:43:CYS:HB2	1:A:52:PHE:HD1	1	0.57
(2,224)	1:A:43:CYS:HB2	1:A:52:PHE:HD2	1	0.57
(2,183)	1:A:29:TRP:HE1	1:A:72:MET:HE1	9	0.57
(2,183)	1:A:29:TRP:HE1	1:A:72:MET:HE2	9	0.57
(2,183)	1:A:29:TRP:HE1	1:A:72:MET:HE3	9	0.57
(1,606)	1:A:21:VAL:HA	1:A:51:VAL:HG11	2	0.57
(1,606)	1:A:21:VAL:HA	1:A:51:VAL:HG12	2	0.57
(1,606)	1:A:21:VAL:HA	1:A:51:VAL:HG13	2	0.57
(1,576)	1:A:20:LEU:HD11	1:A:78:TYR:H	3	0.57
(1,576)	1:A:20:LEU:HD12	1:A:78:TYR:H	3	0.57
(1,576)	1:A:20:LEU:HD13	1:A:78:TYR:H	3	0.57
(1,575)	1:A:20:LEU:HD11	1:A:77:VAL:HG11	2	0.57
(1,575)	1:A:20:LEU:HD11	1:A:77:VAL:HG12	2	0.57
(1,575)	1:A:20:LEU:HD11	1:A:77:VAL:HG13	2	0.57
(1,575)	1:A:20:LEU:HD12	1:A:77:VAL:HG11	2	0.57
(1,575)	1:A:20:LEU:HD12	1:A:77:VAL:HG12	2	0.57
(1,575)	1:A:20:LEU:HD12	1:A:77:VAL:HG13	2	0.57
(1,575)	1:A:20:LEU:HD13	1:A:77:VAL:HG11	2	0.57
(1,575)	1:A:20:LEU:HD13	1:A:77:VAL:HG12	2	0.57
(1,575)	1:A:20:LEU:HD13	1:A:77:VAL:HG13	2	0.57
(1,389)	1:A:15:ILE:HD11	1:A:78:TYR:HD1	3	0.57
(1,389)	1:A:15:ILE:HD11	1:A:78:TYR:HD2	3	0.57
(1,389)	1:A:15:ILE:HD12	1:A:78:TYR:HD1	3	0.57
(1,389)	1:A:15:ILE:HD12	1:A:78:TYR:HD2	3	0.57
(1,389)	1:A:15:ILE:HD13	1:A:78:TYR:HD1	3	0.57
(1,389)	1:A:15:ILE:HD13	1:A:78:TYR:HD2	3	0.57
(1,2423)	1:A:99:ILE:HG21	1:A:100:GLU:H	2	0.57
(1,2423)	1:A:99:ILE:HG22	1:A:100:GLU:H	2	0.57
(1,2423)	1:A:99:ILE:HG23	1:A:100:GLU:H	2	0.57

Continued on next page...

Continued from previous page...

Key	Atom-1	Atom-2	Model ID	Violation (Å)
(1,2423)	1:A:99:ILE:HG21	1:A:100:GLU:H	6	0.57
(1,2423)	1:A:99:ILE:HG22	1:A:100:GLU:H	6	0.57
(1,2423)	1:A:99:ILE:HG23	1:A:100:GLU:H	6	0.57
(1,2423)	1:A:99:ILE:HG21	1:A:100:GLU:H	9	0.57
(1,2423)	1:A:99:ILE:HG22	1:A:100:GLU:H	9	0.57
(1,2423)	1:A:99:ILE:HG23	1:A:100:GLU:H	9	0.57
(1,1741)	1:A:69:ILE:HD11	1:A:69:ILE:HG21	10	0.57
(1,1741)	1:A:69:ILE:HD11	1:A:69:ILE:HG22	10	0.57
(1,1741)	1:A:69:ILE:HD11	1:A:69:ILE:HG23	10	0.57
(1,1741)	1:A:69:ILE:HD12	1:A:69:ILE:HG21	10	0.57
(1,1741)	1:A:69:ILE:HD12	1:A:69:ILE:HG22	10	0.57
(1,1741)	1:A:69:ILE:HD12	1:A:69:ILE:HG23	10	0.57
(1,1741)	1:A:69:ILE:HD13	1:A:69:ILE:HG21	10	0.57
(1,1741)	1:A:69:ILE:HD13	1:A:69:ILE:HG22	10	0.57
(1,1741)	1:A:69:ILE:HD13	1:A:69:ILE:HG23	10	0.57
(1,1719)	1:A:68:ASN:HD21	1:A:70:THR:HG21	6	0.57
(1,1719)	1:A:68:ASN:HD21	1:A:70:THR:HG22	6	0.57
(1,1719)	1:A:68:ASN:HD21	1:A:70:THR:HG23	6	0.57
(1,1631)	1:A:63:VAL:HG11	1:A:66:LYS:HG2	7	0.57
(1,1631)	1:A:63:VAL:HG11	1:A:66:LYS:HG3	7	0.57
(1,1631)	1:A:63:VAL:HG12	1:A:66:LYS:HG2	7	0.57
(1,1631)	1:A:63:VAL:HG12	1:A:66:LYS:HG3	7	0.57
(1,1631)	1:A:63:VAL:HG13	1:A:66:LYS:HG2	7	0.57
(1,1631)	1:A:63:VAL:HG13	1:A:66:LYS:HG3	7	0.57
(2,80)	1:A:12:ASP:HB3	1:A:66:LYS:HE2	10	0.56
(2,80)	1:A:12:ASP:HB3	1:A:66:LYS:HE3	10	0.56
(2,74)	1:A:11:PHE:HD1	1:A:12:ASP:HB2	3	0.56
(2,74)	1:A:11:PHE:HD2	1:A:12:ASP:HB2	3	0.56
(2,74)	1:A:11:PHE:HD1	1:A:12:ASP:HB2	5	0.56
(2,74)	1:A:11:PHE:HD2	1:A:12:ASP:HB2	5	0.56
(2,74)	1:A:11:PHE:HD1	1:A:12:ASP:HB2	8	0.56
(2,74)	1:A:11:PHE:HD2	1:A:12:ASP:HB2	8	0.56
(2,74)	1:A:11:PHE:HD1	1:A:12:ASP:HB2	9	0.56
(2,74)	1:A:11:PHE:HD2	1:A:12:ASP:HB2	9	0.56
(2,359)	1:A:74:THR:H	1:A:75:PHE:HE1	3	0.56
(2,359)	1:A:74:THR:H	1:A:75:PHE:HE2	3	0.56
(2,359)	1:A:74:THR:H	1:A:75:PHE:HE1	9	0.56
(2,359)	1:A:74:THR:H	1:A:75:PHE:HE2	9	0.56
(2,34)	1:A:3:LYS:HE2	1:A:5:VAL:HB	7	0.56
(2,34)	1:A:3:LYS:HE3	1:A:5:VAL:HB	7	0.56
(2,296)	1:A:67:GLU:HB3	1:A:78:TYR:HE1	8	0.56
(2,296)	1:A:67:GLU:HB3	1:A:78:TYR:HE2	8	0.56

Continued on next page...

Continued from previous page...

Key	Atom-1	Atom-2	Model ID	Violation (Å)
(2,29)	1:A:2:VAL:H	1:A:54:LYS:HE2	8	0.56
(2,29)	1:A:2:VAL:H	1:A:54:LYS:HE3	8	0.56
(2,266)	1:A:50:MET:H	1:A:52:PHE:HD1	10	0.56
(2,266)	1:A:50:MET:H	1:A:52:PHE:HD2	10	0.56
(2,224)	1:A:43:CYS:HB2	1:A:52:PHE:HD1	10	0.56
(2,224)	1:A:43:CYS:HB2	1:A:52:PHE:HD2	10	0.56
(2,199)	1:A:36:ILE:HG21	1:A:40:TYR:HD1	10	0.56
(2,199)	1:A:36:ILE:HG21	1:A:40:TYR:HD2	10	0.56
(2,199)	1:A:36:ILE:HG22	1:A:40:TYR:HD1	10	0.56
(2,199)	1:A:36:ILE:HG22	1:A:40:TYR:HD2	10	0.56
(2,199)	1:A:36:ILE:HG23	1:A:40:TYR:HD1	10	0.56
(2,199)	1:A:36:ILE:HG23	1:A:40:TYR:HD2	10	0.56
(2,140)	1:A:26:PHE:HE1	1:A:54:LYS:HD2	4	0.56
(2,140)	1:A:26:PHE:HE1	1:A:54:LYS:HD3	4	0.56
(2,140)	1:A:26:PHE:HE2	1:A:54:LYS:HD2	4	0.56
(2,140)	1:A:26:PHE:HE2	1:A:54:LYS:HD3	4	0.56
(2,134)	1:A:26:PHE:HA	1:A:75:PHE:HE1	3	0.56
(2,134)	1:A:26:PHE:HA	1:A:75:PHE:HE2	3	0.56
(1,607)	1:A:21:VAL:HA	1:A:51:VAL:HG21	3	0.56
(1,607)	1:A:21:VAL:HA	1:A:51:VAL:HG22	3	0.56
(1,607)	1:A:21:VAL:HA	1:A:51:VAL:HG23	3	0.56
(1,606)	1:A:21:VAL:HA	1:A:51:VAL:HG11	7	0.56
(1,606)	1:A:21:VAL:HA	1:A:51:VAL:HG12	7	0.56
(1,606)	1:A:21:VAL:HA	1:A:51:VAL:HG13	7	0.56
(1,576)	1:A:20:LEU:HD11	1:A:78:TYR:H	6	0.56
(1,576)	1:A:20:LEU:HD12	1:A:78:TYR:H	6	0.56
(1,576)	1:A:20:LEU:HD13	1:A:78:TYR:H	6	0.56
(1,2423)	1:A:99:ILE:HG21	1:A:100:GLU:H	1	0.56
(1,2423)	1:A:99:ILE:HG22	1:A:100:GLU:H	1	0.56
(1,2423)	1:A:99:ILE:HG23	1:A:100:GLU:H	1	0.56
(1,2423)	1:A:99:ILE:HG21	1:A:100:GLU:H	3	0.56
(1,2423)	1:A:99:ILE:HG22	1:A:100:GLU:H	3	0.56
(1,2423)	1:A:99:ILE:HG23	1:A:100:GLU:H	3	0.56
(1,1980)	1:A:77:VAL:HG21	1:A:102:TYR:HD1	7	0.56
(1,1980)	1:A:77:VAL:HG21	1:A:102:TYR:HD2	7	0.56
(1,1980)	1:A:77:VAL:HG22	1:A:102:TYR:HD1	7	0.56
(1,1980)	1:A:77:VAL:HG22	1:A:102:TYR:HD2	7	0.56
(1,1980)	1:A:77:VAL:HG23	1:A:102:TYR:HD1	7	0.56
(1,1980)	1:A:77:VAL:HG23	1:A:102:TYR:HD2	7	0.56
(2,99)	1:A:20:LEU:HA	1:A:79:LYS:HE2	8	0.55
(2,99)	1:A:20:LEU:HA	1:A:79:LYS:HE3	8	0.55
(2,98)	1:A:20:LEU:HA	1:A:79:LYS:HE2	8	0.55

Continued on next page...

Continued from previous page...

Key	Atom-1	Atom-2	Model ID	Violation (Å)
(2,98)	1:A:20:LEU:HA	1:A:79:LYS:HE3	8	0.55
(2,9)	1:A:1:SER:HB2	1:A:2:VAL:HA	4	0.55
(2,9)	1:A:1:SER:HB3	1:A:2:VAL:HA	4	0.55
(2,409)	1:A:29:TRP:HE3	1:A:30:CYS:H	10	0.55
(2,372)	1:A:77:VAL:HA	1:A:78:TYR:HE1	7	0.55
(2,372)	1:A:77:VAL:HA	1:A:78:TYR:HE2	7	0.55
(2,359)	1:A:74:THR:H	1:A:75:PHE:HE1	2	0.55
(2,359)	1:A:74:THR:H	1:A:75:PHE:HE2	2	0.55
(2,359)	1:A:74:THR:H	1:A:75:PHE:HE1	4	0.55
(2,359)	1:A:74:THR:H	1:A:75:PHE:HE2	4	0.55
(2,359)	1:A:74:THR:H	1:A:75:PHE:HE1	6	0.55
(2,359)	1:A:74:THR:H	1:A:75:PHE:HE2	6	0.55
(2,359)	1:A:74:THR:H	1:A:75:PHE:HE1	7	0.55
(2,359)	1:A:74:THR:H	1:A:75:PHE:HE2	7	0.55
(2,359)	1:A:74:THR:H	1:A:75:PHE:HE1	10	0.55
(2,359)	1:A:74:THR:H	1:A:75:PHE:HE2	10	0.55
(2,296)	1:A:67:GLU:HB3	1:A:78:TYR:HE1	1	0.55
(2,296)	1:A:67:GLU:HB3	1:A:78:TYR:HE2	1	0.55
(2,296)	1:A:67:GLU:HB3	1:A:78:TYR:HE1	3	0.55
(2,296)	1:A:67:GLU:HB3	1:A:78:TYR:HE2	3	0.55
(2,296)	1:A:67:GLU:HB3	1:A:78:TYR:HE1	9	0.55
(2,296)	1:A:67:GLU:HB3	1:A:78:TYR:HE2	9	0.55
(2,266)	1:A:50:MET:H	1:A:52:PHE:HD1	9	0.55
(2,266)	1:A:50:MET:H	1:A:52:PHE:HD2	9	0.55
(2,247)	1:A:47:TYR:HB2	1:A:52:PHE:HD1	1	0.55
(2,247)	1:A:47:TYR:HB2	1:A:52:PHE:HD2	1	0.55
(2,224)	1:A:43:CYS:HB2	1:A:52:PHE:HD1	6	0.55
(2,224)	1:A:43:CYS:HB2	1:A:52:PHE:HD2	6	0.55
(2,215)	1:A:42:GLU:H	1:A:45:LYS:HE2	4	0.55
(2,215)	1:A:42:GLU:H	1:A:45:LYS:HE3	4	0.55
(2,215)	1:A:42:GLU:H	1:A:45:LYS:HE2	6	0.55
(2,215)	1:A:42:GLU:H	1:A:45:LYS:HE3	6	0.55
(2,215)	1:A:42:GLU:H	1:A:45:LYS:HE2	8	0.55
(2,215)	1:A:42:GLU:H	1:A:45:LYS:HE3	8	0.55
(2,209)	1:A:40:TYR:HD1	1:A:43:CYS:H	9	0.55
(2,209)	1:A:40:TYR:HD2	1:A:43:CYS:H	9	0.55
(2,199)	1:A:36:ILE:HG21	1:A:40:TYR:HD1	9	0.55
(2,199)	1:A:36:ILE:HG21	1:A:40:TYR:HD2	9	0.55
(2,199)	1:A:36:ILE:HG22	1:A:40:TYR:HD1	9	0.55
(2,199)	1:A:36:ILE:HG22	1:A:40:TYR:HD2	9	0.55
(2,199)	1:A:36:ILE:HG23	1:A:40:TYR:HD1	9	0.55
(2,199)	1:A:36:ILE:HG23	1:A:40:TYR:HD2	9	0.55

Continued on next page...

Continued from previous page...

Key	Atom-1	Atom-2	Model ID	Violation (Å)
(2,163)	1:A:28:GLU:HG2	1:A:29:TRP:H	4	0.55
(2,163)	1:A:28:GLU:HG3	1:A:29:TRP:H	4	0.55
(1,1392)	1:A:47:TYR:HE1	1:A:99:ILE:HG21	4	0.55
(1,1392)	1:A:47:TYR:HE1	1:A:99:ILE:HG22	4	0.55
(1,1392)	1:A:47:TYR:HE1	1:A:99:ILE:HG23	4	0.55
(1,1392)	1:A:47:TYR:HE2	1:A:99:ILE:HG21	4	0.55
(1,1392)	1:A:47:TYR:HE2	1:A:99:ILE:HG22	4	0.55
(1,1392)	1:A:47:TYR:HE2	1:A:99:ILE:HG23	4	0.55
(1,1084)	1:A:36:ILE:HG21	1:A:40:TYR:HB2	5	0.55
(1,1084)	1:A:36:ILE:HG22	1:A:40:TYR:HB2	5	0.55
(1,1084)	1:A:36:ILE:HG23	1:A:40:TYR:HB2	5	0.55
(2,99)	1:A:20:LEU:HA	1:A:79:LYS:HE2	3	0.54
(2,99)	1:A:20:LEU:HA	1:A:79:LYS:HE3	3	0.54
(2,98)	1:A:20:LEU:HA	1:A:79:LYS:HE2	3	0.54
(2,98)	1:A:20:LEU:HA	1:A:79:LYS:HE3	3	0.54
(2,409)	1:A:29:TRP:HE3	1:A:30:CYS:H	3	0.54
(2,409)	1:A:29:TRP:HE3	1:A:30:CYS:H	5	0.54
(2,409)	1:A:29:TRP:HE3	1:A:30:CYS:H	6	0.54
(2,409)	1:A:29:TRP:HE3	1:A:30:CYS:H	7	0.54
(2,409)	1:A:29:TRP:HE3	1:A:30:CYS:H	8	0.54
(2,32)	1:A:3:LYS:HD2	1:A:53:ILE:HA	5	0.54
(2,32)	1:A:3:LYS:HD3	1:A:53:ILE:HA	5	0.54
(2,247)	1:A:47:TYR:HB2	1:A:52:PHE:HD1	9	0.54
(2,247)	1:A:47:TYR:HB2	1:A:52:PHE:HD2	9	0.54
(2,224)	1:A:43:CYS:HB2	1:A:52:PHE:HD1	2	0.54
(2,224)	1:A:43:CYS:HB2	1:A:52:PHE:HD2	2	0.54
(2,224)	1:A:43:CYS:HB2	1:A:52:PHE:HD1	3	0.54
(2,224)	1:A:43:CYS:HB2	1:A:52:PHE:HD2	3	0.54
(2,163)	1:A:28:GLU:HG2	1:A:29:TRP:H	9	0.54
(2,163)	1:A:28:GLU:HG3	1:A:29:TRP:H	9	0.54
(2,140)	1:A:26:PHE:HE1	1:A:54:LYS:HD2	1	0.54
(2,140)	1:A:26:PHE:HE1	1:A:54:LYS:HD3	1	0.54
(2,140)	1:A:26:PHE:HE2	1:A:54:LYS:HD2	1	0.54
(2,140)	1:A:26:PHE:HE2	1:A:54:LYS:HD3	1	0.54
(2,134)	1:A:26:PHE:HA	1:A:75:PHE:HE1	9	0.54
(2,134)	1:A:26:PHE:HA	1:A:75:PHE:HE2	9	0.54
(2,127)	1:A:25:PHE:HZ	1:A:69:ILE:HD11	5	0.54
(2,127)	1:A:25:PHE:HZ	1:A:69:ILE:HD12	5	0.54
(2,127)	1:A:25:PHE:HZ	1:A:69:ILE:HD13	5	0.54
(2,126)	1:A:25:PHE:HZ	1:A:69:ILE:HD11	5	0.54
(2,126)	1:A:25:PHE:HZ	1:A:69:ILE:HD12	5	0.54
(2,126)	1:A:25:PHE:HZ	1:A:69:ILE:HD13	5	0.54

Continued on next page...

Continued from previous page...

Key	Atom-1	Atom-2	Model ID	Violation (Å)
(1,607)	1:A:21:VAL:HA	1:A:51:VAL:HG21	8	0.54
(1,607)	1:A:21:VAL:HA	1:A:51:VAL:HG22	8	0.54
(1,607)	1:A:21:VAL:HA	1:A:51:VAL:HG23	8	0.54
(1,2475)	1:A:101:LYS:HE2	1:A:102:TYR:HE1	10	0.54
(1,2475)	1:A:101:LYS:HE2	1:A:102:TYR:HE2	10	0.54
(1,2475)	1:A:101:LYS:HE3	1:A:102:TYR:HE1	10	0.54
(1,2475)	1:A:101:LYS:HE3	1:A:102:TYR:HE2	10	0.54
(1,1980)	1:A:77:VAL:HG21	1:A:102:TYR:HD1	10	0.54
(1,1980)	1:A:77:VAL:HG21	1:A:102:TYR:HD2	10	0.54
(1,1980)	1:A:77:VAL:HG22	1:A:102:TYR:HD1	10	0.54
(1,1980)	1:A:77:VAL:HG22	1:A:102:TYR:HD2	10	0.54
(1,1980)	1:A:77:VAL:HG23	1:A:102:TYR:HD1	10	0.54
(1,1980)	1:A:77:VAL:HG23	1:A:102:TYR:HD2	10	0.54
(2,80)	1:A:12:ASP:HB3	1:A:66:LYS:HE2	6	0.53
(2,80)	1:A:12:ASP:HB3	1:A:66:LYS:HE3	6	0.53
(2,80)	1:A:12:ASP:HB3	1:A:66:LYS:HE2	8	0.53
(2,80)	1:A:12:ASP:HB3	1:A:66:LYS:HE3	8	0.53
(2,409)	1:A:29:TRP:HE3	1:A:30:CYS:H	2	0.53
(2,215)	1:A:42:GLU:H	1:A:45:LYS:HE2	10	0.53
(2,215)	1:A:42:GLU:H	1:A:45:LYS:HE3	10	0.53
(2,199)	1:A:36:ILE:HG21	1:A:40:TYR:HD1	4	0.53
(2,199)	1:A:36:ILE:HG21	1:A:40:TYR:HD2	4	0.53
(2,199)	1:A:36:ILE:HG22	1:A:40:TYR:HD1	4	0.53
(2,199)	1:A:36:ILE:HG22	1:A:40:TYR:HD2	4	0.53
(2,199)	1:A:36:ILE:HG23	1:A:40:TYR:HD1	4	0.53
(2,199)	1:A:36:ILE:HG23	1:A:40:TYR:HD2	4	0.53
(2,163)	1:A:28:GLU:HG2	1:A:29:TRP:H	1	0.53
(2,163)	1:A:28:GLU:HG3	1:A:29:TRP:H	1	0.53
(2,127)	1:A:25:PHE:HZ	1:A:69:ILE:HD11	8	0.53
(2,127)	1:A:25:PHE:HZ	1:A:69:ILE:HD12	8	0.53
(2,127)	1:A:25:PHE:HZ	1:A:69:ILE:HD13	8	0.53
(2,126)	1:A:25:PHE:HZ	1:A:69:ILE:HD11	8	0.53
(2,126)	1:A:25:PHE:HZ	1:A:69:ILE:HD12	8	0.53
(2,126)	1:A:25:PHE:HZ	1:A:69:ILE:HD13	8	0.53
(1,607)	1:A:21:VAL:HA	1:A:51:VAL:HG21	1	0.53
(1,607)	1:A:21:VAL:HA	1:A:51:VAL:HG22	1	0.53
(1,607)	1:A:21:VAL:HA	1:A:51:VAL:HG23	1	0.53
(1,607)	1:A:21:VAL:HA	1:A:51:VAL:HG21	10	0.53
(1,607)	1:A:21:VAL:HA	1:A:51:VAL:HG22	10	0.53
(1,607)	1:A:21:VAL:HA	1:A:51:VAL:HG23	10	0.53
(1,1950)	1:A:77:VAL:HG11	1:A:84:VAL:HG11	5	0.53
(1,1950)	1:A:77:VAL:HG11	1:A:84:VAL:HG12	5	0.53

Continued on next page...

Continued from previous page...

Key	Atom-1	Atom-2	Model ID	Violation (Å)
(1,1950)	1:A:77:VAL:HG11	1:A:84:VAL:HG13	5	0.53
(1,1950)	1:A:77:VAL:HG12	1:A:84:VAL:HG11	5	0.53
(1,1950)	1:A:77:VAL:HG12	1:A:84:VAL:HG12	5	0.53
(1,1950)	1:A:77:VAL:HG12	1:A:84:VAL:HG13	5	0.53
(1,1950)	1:A:77:VAL:HG13	1:A:84:VAL:HG11	5	0.53
(1,1950)	1:A:77:VAL:HG13	1:A:84:VAL:HG12	5	0.53
(1,1950)	1:A:77:VAL:HG13	1:A:84:VAL:HG13	5	0.53
(2,80)	1:A:12:ASP:HB3	1:A:66:LYS:HE2	3	0.52
(2,80)	1:A:12:ASP:HB3	1:A:66:LYS:HE3	3	0.52
(2,266)	1:A:50:MET:H	1:A:52:PHE:HD1	2	0.52
(2,266)	1:A:50:MET:H	1:A:52:PHE:HD2	2	0.52
(2,266)	1:A:50:MET:H	1:A:52:PHE:HD1	6	0.52
(2,266)	1:A:50:MET:H	1:A:52:PHE:HD2	6	0.52
(2,230)	1:A:44:SER:HG	1:A:45:LYS:HE2	10	0.52
(2,230)	1:A:44:SER:HG	1:A:45:LYS:HE3	10	0.52
(2,208)	1:A:40:TYR:HD1	1:A:41:GLU:H	7	0.52
(2,208)	1:A:40:TYR:HD2	1:A:41:GLU:H	7	0.52
(2,193)	1:A:29:TRP:HZ3	1:A:72:MET:HE1	10	0.52
(2,193)	1:A:29:TRP:HZ3	1:A:72:MET:HE2	10	0.52
(2,193)	1:A:29:TRP:HZ3	1:A:72:MET:HE3	10	0.52
(1,607)	1:A:21:VAL:HA	1:A:51:VAL:HG21	4	0.52
(1,607)	1:A:21:VAL:HA	1:A:51:VAL:HG22	4	0.52
(1,607)	1:A:21:VAL:HA	1:A:51:VAL:HG23	4	0.52
(1,607)	1:A:21:VAL:HA	1:A:51:VAL:HG21	5	0.52
(1,607)	1:A:21:VAL:HA	1:A:51:VAL:HG22	5	0.52
(1,607)	1:A:21:VAL:HA	1:A:51:VAL:HG23	5	0.52
(1,426)	1:A:15:ILE:HG21	1:A:78:TYR:HD1	1	0.52
(1,426)	1:A:15:ILE:HG21	1:A:78:TYR:HD2	1	0.52
(1,426)	1:A:15:ILE:HG22	1:A:78:TYR:HD1	1	0.52
(1,426)	1:A:15:ILE:HG22	1:A:78:TYR:HD2	1	0.52
(1,426)	1:A:15:ILE:HG23	1:A:78:TYR:HD1	1	0.52
(1,426)	1:A:15:ILE:HG23	1:A:78:TYR:HD2	1	0.52
(1,426)	1:A:15:ILE:HG21	1:A:78:TYR:HD1	6	0.52
(1,426)	1:A:15:ILE:HG21	1:A:78:TYR:HD2	6	0.52
(1,426)	1:A:15:ILE:HG22	1:A:78:TYR:HD1	6	0.52
(1,426)	1:A:15:ILE:HG22	1:A:78:TYR:HD2	6	0.52
(1,426)	1:A:15:ILE:HG23	1:A:78:TYR:HD1	6	0.52
(1,426)	1:A:15:ILE:HG23	1:A:78:TYR:HD2	6	0.52
(1,2351)	1:A:97:GLN:HE21	1:A:97:GLN:HB2	6	0.52
(1,2351)	1:A:97:GLN:HE21	1:A:97:GLN:HB3	6	0.52
(1,2006)	1:A:78:TYR:HD1	1:A:15:ILE:HG21	1	0.52
(1,2006)	1:A:78:TYR:HD1	1:A:15:ILE:HG22	1	0.52

Continued on next page...

Continued from previous page...

Key	Atom-1	Atom-2	Model ID	Violation (Å)
(1,2006)	1:A:78:TYR:HD1	1:A:15:ILE:HG23	1	0.52
(1,2006)	1:A:78:TYR:HD2	1:A:15:ILE:HG21	1	0.52
(1,2006)	1:A:78:TYR:HD2	1:A:15:ILE:HG22	1	0.52
(1,2006)	1:A:78:TYR:HD2	1:A:15:ILE:HG23	1	0.52
(1,2006)	1:A:78:TYR:HD1	1:A:15:ILE:HG21	6	0.52
(1,2006)	1:A:78:TYR:HD1	1:A:15:ILE:HG22	6	0.52
(1,2006)	1:A:78:TYR:HD1	1:A:15:ILE:HG23	6	0.52
(1,2006)	1:A:78:TYR:HD2	1:A:15:ILE:HG21	6	0.52
(1,2006)	1:A:78:TYR:HD2	1:A:15:ILE:HG22	6	0.52
(1,2006)	1:A:78:TYR:HD2	1:A:15:ILE:HG23	6	0.52
(1,1950)	1:A:77:VAL:HG11	1:A:84:VAL:HG11	6	0.52
(1,1950)	1:A:77:VAL:HG11	1:A:84:VAL:HG12	6	0.52
(1,1950)	1:A:77:VAL:HG11	1:A:84:VAL:HG13	6	0.52
(1,1950)	1:A:77:VAL:HG12	1:A:84:VAL:HG11	6	0.52
(1,1950)	1:A:77:VAL:HG12	1:A:84:VAL:HG12	6	0.52
(1,1950)	1:A:77:VAL:HG12	1:A:84:VAL:HG13	6	0.52
(1,1950)	1:A:77:VAL:HG13	1:A:84:VAL:HG11	6	0.52
(1,1950)	1:A:77:VAL:HG13	1:A:84:VAL:HG12	6	0.52
(1,1950)	1:A:77:VAL:HG13	1:A:84:VAL:HG13	6	0.52
(2,83)	1:A:12:ASP:HB2	1:A:66:LYS:HE2	10	0.51
(2,83)	1:A:12:ASP:HB2	1:A:66:LYS:HE3	10	0.51
(2,325)	1:A:69:ILE:HG21	1:A:71:SER:HA	7	0.51
(2,325)	1:A:69:ILE:HG22	1:A:71:SER:HA	7	0.51
(2,325)	1:A:69:ILE:HG23	1:A:71:SER:HA	7	0.51
(2,325)	1:A:69:ILE:HG21	1:A:71:SER:HA	8	0.51
(2,325)	1:A:69:ILE:HG22	1:A:71:SER:HA	8	0.51
(2,325)	1:A:69:ILE:HG23	1:A:71:SER:HA	8	0.51
(2,287)	1:A:57:VAL:HG21	1:A:61:SER:HA	3	0.51
(2,287)	1:A:57:VAL:HG22	1:A:61:SER:HA	3	0.51
(2,287)	1:A:57:VAL:HG23	1:A:61:SER:HA	3	0.51
(1,424)	1:A:15:ILE:HG21	1:A:78:TYR:HB3	8	0.51
(1,424)	1:A:15:ILE:HG22	1:A:78:TYR:HB3	8	0.51
(1,424)	1:A:15:ILE:HG23	1:A:78:TYR:HB3	8	0.51
(1,1950)	1:A:77:VAL:HG11	1:A:84:VAL:HG11	1	0.51
(1,1950)	1:A:77:VAL:HG11	1:A:84:VAL:HG12	1	0.51
(1,1950)	1:A:77:VAL:HG11	1:A:84:VAL:HG13	1	0.51
(1,1950)	1:A:77:VAL:HG12	1:A:84:VAL:HG11	1	0.51
(1,1950)	1:A:77:VAL:HG12	1:A:84:VAL:HG12	1	0.51
(1,1950)	1:A:77:VAL:HG12	1:A:84:VAL:HG13	1	0.51
(1,1950)	1:A:77:VAL:HG13	1:A:84:VAL:HG11	1	0.51
(1,1950)	1:A:77:VAL:HG13	1:A:84:VAL:HG12	1	0.51
(1,1950)	1:A:77:VAL:HG13	1:A:84:VAL:HG13	1	0.51

Continued on next page...

Continued from previous page...

Key	Atom-1	Atom-2	Model ID	Violation (Å)
(1,1950)	1:A:77:VAL:HG11	1:A:84:VAL:HG11	4	0.51
(1,1950)	1:A:77:VAL:HG11	1:A:84:VAL:HG12	4	0.51
(1,1950)	1:A:77:VAL:HG11	1:A:84:VAL:HG13	4	0.51
(1,1950)	1:A:77:VAL:HG12	1:A:84:VAL:HG11	4	0.51
(1,1950)	1:A:77:VAL:HG12	1:A:84:VAL:HG12	4	0.51
(1,1950)	1:A:77:VAL:HG12	1:A:84:VAL:HG13	4	0.51
(1,1950)	1:A:77:VAL:HG13	1:A:84:VAL:HG11	4	0.51
(1,1950)	1:A:77:VAL:HG13	1:A:84:VAL:HG12	4	0.51
(1,1950)	1:A:77:VAL:HG13	1:A:84:VAL:HG13	4	0.51
(1,1916)	1:A:76:LYS:HD2	1:A:86:THR:HG21	5	0.51
(1,1916)	1:A:76:LYS:HD2	1:A:86:THR:HG22	5	0.51
(1,1916)	1:A:76:LYS:HD2	1:A:86:THR:HG23	5	0.51
(1,1916)	1:A:76:LYS:HD3	1:A:86:THR:HG21	5	0.51
(1,1916)	1:A:76:LYS:HD3	1:A:86:THR:HG22	5	0.51
(1,1916)	1:A:76:LYS:HD3	1:A:86:THR:HG23	5	0.51
(2,99)	1:A:20:LEU:HA	1:A:79:LYS:HE2	7	0.5
(2,99)	1:A:20:LEU:HA	1:A:79:LYS:HE3	7	0.5
(2,98)	1:A:20:LEU:HA	1:A:79:LYS:HE2	7	0.5
(2,98)	1:A:20:LEU:HA	1:A:79:LYS:HE3	7	0.5
(2,409)	1:A:29:TRP:HE3	1:A:30:CYS:H	1	0.5
(2,409)	1:A:29:TRP:HE3	1:A:30:CYS:H	4	0.5
(2,409)	1:A:29:TRP:HE3	1:A:30:CYS:H	9	0.5
(2,371)	1:A:76:LYS:HG2	1:A:78:TYR:H	3	0.5
(2,371)	1:A:76:LYS:HG3	1:A:78:TYR:H	3	0.5
(2,328)	1:A:69:ILE:HG21	1:A:72:MET:HB2	3	0.5
(2,328)	1:A:69:ILE:HG22	1:A:72:MET:HB2	3	0.5
(2,328)	1:A:69:ILE:HG23	1:A:72:MET:HB2	3	0.5
(2,258)	1:A:48:THR:HB	1:A:49:LYS:HD2	3	0.5
(2,258)	1:A:48:THR:HB	1:A:49:LYS:HD3	3	0.5
(2,224)	1:A:43:CYS:HB2	1:A:52:PHE:HD1	4	0.5
(2,224)	1:A:43:CYS:HB2	1:A:52:PHE:HD2	4	0.5
(2,109)	1:A:22:ILE:H	1:A:50:MET:HE1	7	0.5
(2,109)	1:A:22:ILE:H	1:A:50:MET:HE2	7	0.5
(2,109)	1:A:22:ILE:H	1:A:50:MET:HE3	7	0.5
(1,424)	1:A:15:ILE:HG21	1:A:78:TYR:HB3	2	0.5
(1,424)	1:A:15:ILE:HG22	1:A:78:TYR:HB3	2	0.5
(1,424)	1:A:15:ILE:HG23	1:A:78:TYR:HB3	2	0.5
(1,424)	1:A:15:ILE:HG21	1:A:78:TYR:HB3	9	0.5
(1,424)	1:A:15:ILE:HG22	1:A:78:TYR:HB3	9	0.5
(1,424)	1:A:15:ILE:HG23	1:A:78:TYR:HB3	9	0.5
(1,1950)	1:A:77:VAL:HG11	1:A:84:VAL:HG11	3	0.5
(1,1950)	1:A:77:VAL:HG11	1:A:84:VAL:HG12	3	0.5

Continued on next page...

Continued from previous page...

Key	Atom-1	Atom-2	Model ID	Violation (Å)
(1,1950)	1:A:77:VAL:HG11	1:A:84:VAL:HG13	3	0.5
(1,1950)	1:A:77:VAL:HG12	1:A:84:VAL:HG11	3	0.5
(1,1950)	1:A:77:VAL:HG12	1:A:84:VAL:HG12	3	0.5
(1,1950)	1:A:77:VAL:HG12	1:A:84:VAL:HG13	3	0.5
(1,1950)	1:A:77:VAL:HG13	1:A:84:VAL:HG11	3	0.5
(1,1950)	1:A:77:VAL:HG13	1:A:84:VAL:HG12	3	0.5
(1,1950)	1:A:77:VAL:HG13	1:A:84:VAL:HG13	3	0.5
(2,341)	1:A:72:MET:HA	1:A:72:MET:HE1	3	0.49
(2,341)	1:A:72:MET:HA	1:A:72:MET:HE2	3	0.49
(2,341)	1:A:72:MET:HA	1:A:72:MET:HE3	3	0.49
(1,424)	1:A:15:ILE:HG21	1:A:78:TYR:HB3	3	0.49
(1,424)	1:A:15:ILE:HG22	1:A:78:TYR:HB3	3	0.49
(1,424)	1:A:15:ILE:HG23	1:A:78:TYR:HB3	3	0.49
(1,424)	1:A:15:ILE:HG21	1:A:78:TYR:HB3	4	0.49
(1,424)	1:A:15:ILE:HG22	1:A:78:TYR:HB3	4	0.49
(1,424)	1:A:15:ILE:HG23	1:A:78:TYR:HB3	4	0.49
(1,424)	1:A:15:ILE:HG21	1:A:78:TYR:HB3	5	0.49
(1,424)	1:A:15:ILE:HG22	1:A:78:TYR:HB3	5	0.49
(1,424)	1:A:15:ILE:HG23	1:A:78:TYR:HB3	5	0.49
(1,424)	1:A:15:ILE:HG21	1:A:78:TYR:HB3	7	0.49
(1,424)	1:A:15:ILE:HG22	1:A:78:TYR:HB3	7	0.49
(1,424)	1:A:15:ILE:HG23	1:A:78:TYR:HB3	7	0.49
(1,1960)	1:A:77:VAL:HG11	1:A:102:TYR:HD1	7	0.49
(1,1960)	1:A:77:VAL:HG11	1:A:102:TYR:HD2	7	0.49
(1,1960)	1:A:77:VAL:HG12	1:A:102:TYR:HD1	7	0.49
(1,1960)	1:A:77:VAL:HG12	1:A:102:TYR:HD2	7	0.49
(1,1960)	1:A:77:VAL:HG13	1:A:102:TYR:HD1	7	0.49
(1,1960)	1:A:77:VAL:HG13	1:A:102:TYR:HD2	7	0.49
(2,244)	1:A:47:TYR:HA	1:A:52:PHE:HE1	1	0.48
(2,244)	1:A:47:TYR:HA	1:A:52:PHE:HE2	1	0.48
(2,163)	1:A:28:GLU:HG2	1:A:29:TRP:H	2	0.48
(2,163)	1:A:28:GLU:HG3	1:A:29:TRP:H	2	0.48
(2,163)	1:A:28:GLU:HG2	1:A:29:TRP:H	8	0.48
(2,163)	1:A:28:GLU:HG3	1:A:29:TRP:H	8	0.48
(1,749)	1:A:23:VAL:HG21	1:A:54:LYS:HA	4	0.48
(1,749)	1:A:23:VAL:HG22	1:A:54:LYS:HA	4	0.48
(1,749)	1:A:23:VAL:HG23	1:A:54:LYS:HA	4	0.48
(1,747)	1:A:23:VAL:HG21	1:A:53:ILE:HG12	8	0.48
(1,747)	1:A:23:VAL:HG22	1:A:53:ILE:HG12	8	0.48
(1,747)	1:A:23:VAL:HG23	1:A:53:ILE:HG12	8	0.48
(1,577)	1:A:20:LEU:HD11	1:A:79:LYS:HA	4	0.48
(1,577)	1:A:20:LEU:HD12	1:A:79:LYS:HA	4	0.48

Continued on next page...

Continued from previous page...

Key	Atom-1	Atom-2	Model ID	Violation (Å)
(1,577)	1:A:20:LEU:HD13	1:A:79:LYS:HA	4	0.48
(1,577)	1:A:20:LEU:HD11	1:A:79:LYS:HA	7	0.48
(1,577)	1:A:20:LEU:HD12	1:A:79:LYS:HA	7	0.48
(1,577)	1:A:20:LEU:HD13	1:A:79:LYS:HA	7	0.48
(1,577)	1:A:20:LEU:HD11	1:A:79:LYS:HA	8	0.48
(1,577)	1:A:20:LEU:HD12	1:A:79:LYS:HA	8	0.48
(1,577)	1:A:20:LEU:HD13	1:A:79:LYS:HA	8	0.48
(1,424)	1:A:15:ILE:HG21	1:A:78:TYR:HB3	6	0.48
(1,424)	1:A:15:ILE:HG22	1:A:78:TYR:HB3	6	0.48
(1,424)	1:A:15:ILE:HG23	1:A:78:TYR:HB3	6	0.48
(1,424)	1:A:15:ILE:HG21	1:A:78:TYR:HB3	10	0.48
(1,424)	1:A:15:ILE:HG22	1:A:78:TYR:HB3	10	0.48
(1,424)	1:A:15:ILE:HG23	1:A:78:TYR:HB3	10	0.48
(1,2544)	1:A:27:ALA:HB1	1:A:29:TRP:HE1	1	0.48
(1,2544)	1:A:27:ALA:HB2	1:A:29:TRP:HE1	1	0.48
(1,2544)	1:A:27:ALA:HB3	1:A:29:TRP:HE1	1	0.48
(1,2544)	1:A:27:ALA:HB1	1:A:29:TRP:HE1	3	0.48
(1,2544)	1:A:27:ALA:HB2	1:A:29:TRP:HE1	3	0.48
(1,2544)	1:A:27:ALA:HB3	1:A:29:TRP:HE1	3	0.48
(1,2544)	1:A:27:ALA:HB1	1:A:29:TRP:HE1	9	0.48
(1,2544)	1:A:27:ALA:HB2	1:A:29:TRP:HE1	9	0.48
(1,2544)	1:A:27:ALA:HB3	1:A:29:TRP:HE1	9	0.48
(2,224)	1:A:43:CYS:HB2	1:A:52:PHE:HD1	7	0.47
(2,224)	1:A:43:CYS:HB2	1:A:52:PHE:HD2	7	0.47
(2,224)	1:A:43:CYS:HB2	1:A:52:PHE:HD1	9	0.47
(2,224)	1:A:43:CYS:HB2	1:A:52:PHE:HD2	9	0.47
(2,163)	1:A:28:GLU:HG2	1:A:29:TRP:H	3	0.47
(2,163)	1:A:28:GLU:HG3	1:A:29:TRP:H	3	0.47
(2,163)	1:A:28:GLU:HG2	1:A:29:TRP:H	5	0.47
(2,163)	1:A:28:GLU:HG3	1:A:29:TRP:H	5	0.47
(2,163)	1:A:28:GLU:HG2	1:A:29:TRP:H	6	0.47
(2,163)	1:A:28:GLU:HG3	1:A:29:TRP:H	6	0.47
(2,163)	1:A:28:GLU:HG2	1:A:29:TRP:H	7	0.47
(2,163)	1:A:28:GLU:HG3	1:A:29:TRP:H	7	0.47
(1,749)	1:A:23:VAL:HG21	1:A:54:LYS:HA	6	0.47
(1,749)	1:A:23:VAL:HG22	1:A:54:LYS:HA	6	0.47
(1,749)	1:A:23:VAL:HG23	1:A:54:LYS:HA	6	0.47
(1,712)	1:A:23:VAL:HG11	1:A:25:PHE:HZ	3	0.47
(1,712)	1:A:23:VAL:HG12	1:A:25:PHE:HZ	3	0.47
(1,712)	1:A:23:VAL:HG13	1:A:25:PHE:HZ	3	0.47
(1,692)	1:A:22:ILE:H	1:A:51:VAL:HG11	9	0.47
(1,692)	1:A:22:ILE:H	1:A:51:VAL:HG12	9	0.47

Continued on next page...

Continued from previous page...

Key	Atom-1	Atom-2	Model ID	Violation (Å)
(1,692)	1:A:22:ILE:H	1:A:51:VAL:HG13	9	0.47
(1,577)	1:A:20:LEU:HD11	1:A:79:LYS:HA	1	0.47
(1,577)	1:A:20:LEU:HD12	1:A:79:LYS:HA	1	0.47
(1,577)	1:A:20:LEU:HD13	1:A:79:LYS:HA	1	0.47
(1,577)	1:A:20:LEU:HD11	1:A:79:LYS:HA	3	0.47
(1,577)	1:A:20:LEU:HD12	1:A:79:LYS:HA	3	0.47
(1,577)	1:A:20:LEU:HD13	1:A:79:LYS:HA	3	0.47
(1,2197)	1:A:88:LEU:HD11	1:A:89:GLY:H	7	0.47
(1,2197)	1:A:88:LEU:HD12	1:A:89:GLY:H	7	0.47
(1,2197)	1:A:88:LEU:HD13	1:A:89:GLY:H	7	0.47
(1,2197)	1:A:88:LEU:HD21	1:A:89:GLY:H	7	0.47
(1,2197)	1:A:88:LEU:HD22	1:A:89:GLY:H	7	0.47
(1,2197)	1:A:88:LEU:HD23	1:A:89:GLY:H	7	0.47
(2,341)	1:A:72:MET:HA	1:A:72:MET:HE1	10	0.46
(2,341)	1:A:72:MET:HA	1:A:72:MET:HE2	10	0.46
(2,341)	1:A:72:MET:HA	1:A:72:MET:HE3	10	0.46
(2,247)	1:A:47:TYR:HB2	1:A:52:PHE:HD1	4	0.46
(2,247)	1:A:47:TYR:HB2	1:A:52:PHE:HD2	4	0.46
(2,224)	1:A:43:CYS:HB2	1:A:52:PHE:HD1	5	0.46
(2,224)	1:A:43:CYS:HB2	1:A:52:PHE:HD2	5	0.46
(2,208)	1:A:40:TYR:HD1	1:A:41:GLU:H	2	0.46
(2,208)	1:A:40:TYR:HD2	1:A:41:GLU:H	2	0.46
(2,199)	1:A:36:ILE:HG21	1:A:40:TYR:HD1	2	0.46
(2,199)	1:A:36:ILE:HG21	1:A:40:TYR:HD2	2	0.46
(2,199)	1:A:36:ILE:HG22	1:A:40:TYR:HD1	2	0.46
(2,199)	1:A:36:ILE:HG22	1:A:40:TYR:HD2	2	0.46
(2,199)	1:A:36:ILE:HG23	1:A:40:TYR:HD1	2	0.46
(2,199)	1:A:36:ILE:HG23	1:A:40:TYR:HD2	2	0.46
(2,163)	1:A:28:GLU:HG2	1:A:29:TRP:H	10	0.46
(2,163)	1:A:28:GLU:HG3	1:A:29:TRP:H	10	0.46
(1,712)	1:A:23:VAL:HG11	1:A:25:PHE:HZ	2	0.46
(1,712)	1:A:23:VAL:HG12	1:A:25:PHE:HZ	2	0.46
(1,712)	1:A:23:VAL:HG13	1:A:25:PHE:HZ	2	0.46
(1,712)	1:A:23:VAL:HG11	1:A:25:PHE:HZ	5	0.46
(1,712)	1:A:23:VAL:HG12	1:A:25:PHE:HZ	5	0.46
(1,712)	1:A:23:VAL:HG13	1:A:25:PHE:HZ	5	0.46
(1,712)	1:A:23:VAL:HG11	1:A:25:PHE:HZ	9	0.46
(1,712)	1:A:23:VAL:HG12	1:A:25:PHE:HZ	9	0.46
(1,712)	1:A:23:VAL:HG13	1:A:25:PHE:HZ	9	0.46
(1,712)	1:A:23:VAL:HG11	1:A:25:PHE:HZ	10	0.46
(1,712)	1:A:23:VAL:HG12	1:A:25:PHE:HZ	10	0.46
(1,712)	1:A:23:VAL:HG13	1:A:25:PHE:HZ	10	0.46

Continued on next page...

Continued from previous page...

Key	Atom-1	Atom-2	Model ID	Violation (Å)
(1,587)	1:A:20:LEU:HG	1:A:77:VAL:HG11	3	0.46
(1,587)	1:A:20:LEU:HG	1:A:77:VAL:HG12	3	0.46
(1,587)	1:A:20:LEU:HG	1:A:77:VAL:HG13	3	0.46
(1,577)	1:A:20:LEU:HD11	1:A:79:LYS:HA	6	0.46
(1,577)	1:A:20:LEU:HD12	1:A:79:LYS:HA	6	0.46
(1,577)	1:A:20:LEU:HD13	1:A:79:LYS:HA	6	0.46
(1,577)	1:A:20:LEU:HD11	1:A:79:LYS:HA	9	0.46
(1,577)	1:A:20:LEU:HD12	1:A:79:LYS:HA	9	0.46
(1,577)	1:A:20:LEU:HD13	1:A:79:LYS:HA	9	0.46
(1,577)	1:A:20:LEU:HD11	1:A:79:LYS:HA	10	0.46
(1,577)	1:A:20:LEU:HD12	1:A:79:LYS:HA	10	0.46
(1,577)	1:A:20:LEU:HD13	1:A:79:LYS:HA	10	0.46
(1,576)	1:A:20:LEU:HD11	1:A:78:TYR:H	5	0.46
(1,576)	1:A:20:LEU:HD12	1:A:78:TYR:H	5	0.46
(1,576)	1:A:20:LEU:HD13	1:A:78:TYR:H	5	0.46
(1,555)	1:A:20:LEU:HA	1:A:20:LEU:HD11	4	0.46
(1,555)	1:A:20:LEU:HA	1:A:20:LEU:HD12	4	0.46
(1,555)	1:A:20:LEU:HA	1:A:20:LEU:HD13	4	0.46
(1,424)	1:A:15:ILE:HG21	1:A:78:TYR:HB3	1	0.46
(1,424)	1:A:15:ILE:HG22	1:A:78:TYR:HB3	1	0.46
(1,424)	1:A:15:ILE:HG23	1:A:78:TYR:HB3	1	0.46
(1,2544)	1:A:27:ALA:HB1	1:A:29:TRP:HE1	6	0.46
(1,2544)	1:A:27:ALA:HB2	1:A:29:TRP:HE1	6	0.46
(1,2544)	1:A:27:ALA:HB3	1:A:29:TRP:HE1	6	0.46
(1,2475)	1:A:101:LYS:HE2	1:A:102:TYR:HE1	8	0.46
(1,2475)	1:A:101:LYS:HE2	1:A:102:TYR:HE2	8	0.46
(1,2475)	1:A:101:LYS:HE3	1:A:102:TYR:HE1	8	0.46
(1,2475)	1:A:101:LYS:HE3	1:A:102:TYR:HE2	8	0.46
(1,2310)	1:A:95:LEU:HD21	1:A:95:LEU:HB3	5	0.46
(1,2310)	1:A:95:LEU:HD22	1:A:95:LEU:HB3	5	0.46
(1,2310)	1:A:95:LEU:HD23	1:A:95:LEU:HB3	5	0.46
(1,2310)	1:A:95:LEU:HD21	1:A:95:LEU:HB3	8	0.46
(1,2310)	1:A:95:LEU:HD22	1:A:95:LEU:HB3	8	0.46
(1,2310)	1:A:95:LEU:HD23	1:A:95:LEU:HB3	8	0.46
(1,1960)	1:A:77:VAL:HG11	1:A:102:TYR:HD1	3	0.46
(1,1960)	1:A:77:VAL:HG11	1:A:102:TYR:HD2	3	0.46
(1,1960)	1:A:77:VAL:HG12	1:A:102:TYR:HD1	3	0.46
(1,1960)	1:A:77:VAL:HG12	1:A:102:TYR:HD2	3	0.46
(1,1960)	1:A:77:VAL:HG13	1:A:102:TYR:HD1	3	0.46
(1,1960)	1:A:77:VAL:HG13	1:A:102:TYR:HD2	3	0.46
(1,1392)	1:A:47:TYR:HE1	1:A:99:ILE:HG21	2	0.46
(1,1392)	1:A:47:TYR:HE1	1:A:99:ILE:HG22	2	0.46

Continued on next page...

Continued from previous page...

Key	Atom-1	Atom-2	Model ID	Violation (Å)
(1,1392)	1:A:47:TYR:HE1	1:A:99:ILE:HG23	2	0.46
(1,1392)	1:A:47:TYR:HE2	1:A:99:ILE:HG21	2	0.46
(1,1392)	1:A:47:TYR:HE2	1:A:99:ILE:HG22	2	0.46
(1,1392)	1:A:47:TYR:HE2	1:A:99:ILE:HG23	2	0.46
(1,1359)	1:A:47:TYR:HB3	1:A:103:ALA:HB1	1	0.46
(1,1359)	1:A:47:TYR:HB3	1:A:103:ALA:HB2	1	0.46
(1,1359)	1:A:47:TYR:HB3	1:A:103:ALA:HB3	1	0.46
(2,9)	1:A:1:SER:HB2	1:A:2:VAL:HA	10	0.45
(2,9)	1:A:1:SER:HB3	1:A:2:VAL:HA	10	0.45
(2,83)	1:A:12:ASP:HB2	1:A:66:LYS:HE2	3	0.45
(2,83)	1:A:12:ASP:HB2	1:A:66:LYS:HE3	3	0.45
(2,341)	1:A:72:MET:HA	1:A:72:MET:HE1	9	0.45
(2,341)	1:A:72:MET:HA	1:A:72:MET:HE2	9	0.45
(2,341)	1:A:72:MET:HA	1:A:72:MET:HE3	9	0.45
(2,266)	1:A:50:MET:H	1:A:52:PHE:HD1	8	0.45
(2,266)	1:A:50:MET:H	1:A:52:PHE:HD2	8	0.45
(2,224)	1:A:43:CYS:HB2	1:A:52:PHE:HD1	8	0.45
(2,224)	1:A:43:CYS:HB2	1:A:52:PHE:HD2	8	0.45
(2,208)	1:A:40:TYR:HD1	1:A:41:GLU:H	9	0.45
(2,208)	1:A:40:TYR:HD2	1:A:41:GLU:H	9	0.45
(2,193)	1:A:29:TRP:HZ3	1:A:72:MET:HE1	6	0.45
(2,193)	1:A:29:TRP:HZ3	1:A:72:MET:HE2	6	0.45
(2,193)	1:A:29:TRP:HZ3	1:A:72:MET:HE3	6	0.45
(2,118)	1:A:25:PHE:HD1	1:A:67:GLU:HG2	5	0.45
(2,118)	1:A:25:PHE:HD1	1:A:67:GLU:HG3	5	0.45
(2,118)	1:A:25:PHE:HD2	1:A:67:GLU:HG2	5	0.45
(2,118)	1:A:25:PHE:HD2	1:A:67:GLU:HG3	5	0.45
(1,747)	1:A:23:VAL:HG21	1:A:53:ILE:HG12	10	0.45
(1,747)	1:A:23:VAL:HG22	1:A:53:ILE:HG12	10	0.45
(1,747)	1:A:23:VAL:HG23	1:A:53:ILE:HG12	10	0.45
(1,712)	1:A:23:VAL:HG11	1:A:25:PHE:HZ	1	0.45
(1,712)	1:A:23:VAL:HG12	1:A:25:PHE:HZ	1	0.45
(1,712)	1:A:23:VAL:HG13	1:A:25:PHE:HZ	1	0.45
(1,712)	1:A:23:VAL:HG11	1:A:25:PHE:HZ	6	0.45
(1,712)	1:A:23:VAL:HG12	1:A:25:PHE:HZ	6	0.45
(1,712)	1:A:23:VAL:HG13	1:A:25:PHE:HZ	6	0.45
(1,575)	1:A:20:LEU:HD11	1:A:77:VAL:HG11	8	0.45
(1,575)	1:A:20:LEU:HD11	1:A:77:VAL:HG12	8	0.45
(1,575)	1:A:20:LEU:HD11	1:A:77:VAL:HG13	8	0.45
(1,575)	1:A:20:LEU:HD12	1:A:77:VAL:HG11	8	0.45
(1,575)	1:A:20:LEU:HD12	1:A:77:VAL:HG12	8	0.45
(1,575)	1:A:20:LEU:HD12	1:A:77:VAL:HG13	8	0.45

Continued on next page...

Continued from previous page...

Key	Atom-1	Atom-2	Model ID	Violation (Å)
(1,575)	1:A:20:LEU:HD13	1:A:77:VAL:HG11	8	0.45
(1,575)	1:A:20:LEU:HD13	1:A:77:VAL:HG12	8	0.45
(1,575)	1:A:20:LEU:HD13	1:A:77:VAL:HG13	8	0.45
(1,555)	1:A:20:LEU:HA	1:A:20:LEU:HD11	1	0.45
(1,555)	1:A:20:LEU:HA	1:A:20:LEU:HD12	1	0.45
(1,555)	1:A:20:LEU:HA	1:A:20:LEU:HD13	1	0.45
(1,555)	1:A:20:LEU:HA	1:A:20:LEU:HD11	9	0.45
(1,555)	1:A:20:LEU:HA	1:A:20:LEU:HD12	9	0.45
(1,555)	1:A:20:LEU:HA	1:A:20:LEU:HD13	9	0.45
(1,2544)	1:A:27:ALA:HB1	1:A:29:TRP:HE1	2	0.45
(1,2544)	1:A:27:ALA:HB2	1:A:29:TRP:HE1	2	0.45
(1,2544)	1:A:27:ALA:HB3	1:A:29:TRP:HE1	2	0.45
(1,2429)	1:A:99:ILE:H	1:A:99:ILE:HG21	5	0.45
(1,2429)	1:A:99:ILE:H	1:A:99:ILE:HG22	5	0.45
(1,2429)	1:A:99:ILE:H	1:A:99:ILE:HG23	5	0.45
(1,2310)	1:A:95:LEU:HD21	1:A:95:LEU:HB3	1	0.45
(1,2310)	1:A:95:LEU:HD22	1:A:95:LEU:HB3	1	0.45
(1,2310)	1:A:95:LEU:HD23	1:A:95:LEU:HB3	1	0.45
(1,2310)	1:A:95:LEU:HD21	1:A:95:LEU:HB3	2	0.45
(1,2310)	1:A:95:LEU:HD22	1:A:95:LEU:HB3	2	0.45
(1,2310)	1:A:95:LEU:HD23	1:A:95:LEU:HB3	2	0.45
(1,2310)	1:A:95:LEU:HD21	1:A:95:LEU:HB3	3	0.45
(1,2310)	1:A:95:LEU:HD22	1:A:95:LEU:HB3	3	0.45
(1,2310)	1:A:95:LEU:HD23	1:A:95:LEU:HB3	3	0.45
(1,2310)	1:A:95:LEU:HD21	1:A:95:LEU:HB3	7	0.45
(1,2310)	1:A:95:LEU:HD22	1:A:95:LEU:HB3	7	0.45
(1,2310)	1:A:95:LEU:HD23	1:A:95:LEU:HB3	7	0.45
(1,2244)	1:A:91:ASN:HD21	1:A:94:ALA:HB1	10	0.45
(1,2244)	1:A:91:ASN:HD21	1:A:94:ALA:HB2	10	0.45
(1,2244)	1:A:91:ASN:HD21	1:A:94:ALA:HB3	10	0.45
(1,2172)	1:A:87:LEU:HD11	1:A:91:ASN:HB2	5	0.45
(1,2172)	1:A:87:LEU:HD12	1:A:91:ASN:HB2	5	0.45
(1,2172)	1:A:87:LEU:HD13	1:A:91:ASN:HB2	5	0.45
(1,2123)	1:A:84:VAL:HG11	1:A:85:ASP:H	2	0.45
(1,2123)	1:A:84:VAL:HG12	1:A:85:ASP:H	2	0.45
(1,2123)	1:A:84:VAL:HG13	1:A:85:ASP:H	2	0.45
(1,1960)	1:A:77:VAL:HG11	1:A:102:TYR:HD1	10	0.45
(1,1960)	1:A:77:VAL:HG11	1:A:102:TYR:HD2	10	0.45
(1,1960)	1:A:77:VAL:HG12	1:A:102:TYR:HD1	10	0.45
(1,1960)	1:A:77:VAL:HG12	1:A:102:TYR:HD2	10	0.45
(1,1960)	1:A:77:VAL:HG13	1:A:102:TYR:HD1	10	0.45
(1,1960)	1:A:77:VAL:HG13	1:A:102:TYR:HD2	10	0.45

Continued on next page...

Continued from previous page...

Key	Atom-1	Atom-2	Model ID	Violation (Å)
(1,1950)	1:A:77:VAL:HG11	1:A:84:VAL:HG11	2	0.45
(1,1950)	1:A:77:VAL:HG11	1:A:84:VAL:HG12	2	0.45
(1,1950)	1:A:77:VAL:HG11	1:A:84:VAL:HG13	2	0.45
(1,1950)	1:A:77:VAL:HG12	1:A:84:VAL:HG11	2	0.45
(1,1950)	1:A:77:VAL:HG12	1:A:84:VAL:HG12	2	0.45
(1,1950)	1:A:77:VAL:HG12	1:A:84:VAL:HG13	2	0.45
(1,1950)	1:A:77:VAL:HG13	1:A:84:VAL:HG11	2	0.45
(1,1950)	1:A:77:VAL:HG13	1:A:84:VAL:HG12	2	0.45
(1,1950)	1:A:77:VAL:HG13	1:A:84:VAL:HG13	2	0.45
(1,1916)	1:A:76:LYS:HD2	1:A:86:THR:HG21	7	0.45
(1,1916)	1:A:76:LYS:HD2	1:A:86:THR:HG22	7	0.45
(1,1916)	1:A:76:LYS:HD2	1:A:86:THR:HG23	7	0.45
(1,1916)	1:A:76:LYS:HD3	1:A:86:THR:HG21	7	0.45
(1,1916)	1:A:76:LYS:HD3	1:A:86:THR:HG22	7	0.45
(1,1916)	1:A:76:LYS:HD3	1:A:86:THR:HG23	7	0.45
(1,1916)	1:A:76:LYS:HD2	1:A:86:THR:HG21	10	0.45
(1,1916)	1:A:76:LYS:HD2	1:A:86:THR:HG22	10	0.45
(1,1916)	1:A:76:LYS:HD2	1:A:86:THR:HG23	10	0.45
(1,1916)	1:A:76:LYS:HD3	1:A:86:THR:HG21	10	0.45
(1,1916)	1:A:76:LYS:HD3	1:A:86:THR:HG22	10	0.45
(1,1916)	1:A:76:LYS:HD3	1:A:86:THR:HG23	10	0.45
(1,1890)	1:A:75:PHE:HZ	1:A:90:ALA:HB1	9	0.45
(1,1890)	1:A:75:PHE:HZ	1:A:90:ALA:HB2	9	0.45
(1,1890)	1:A:75:PHE:HZ	1:A:90:ALA:HB3	9	0.45
(1,1359)	1:A:47:TYR:HB3	1:A:103:ALA:HB1	3	0.45
(1,1359)	1:A:47:TYR:HB3	1:A:103:ALA:HB2	3	0.45
(1,1359)	1:A:47:TYR:HB3	1:A:103:ALA:HB3	3	0.45
(2,402)	1:A:27:ALA:HB1	1:A:30:CYS:H	7	0.44
(2,402)	1:A:27:ALA:HB2	1:A:30:CYS:H	7	0.44
(2,402)	1:A:27:ALA:HB3	1:A:30:CYS:H	7	0.44
(2,372)	1:A:77:VAL:HA	1:A:78:TYR:HE1	2	0.44
(2,372)	1:A:77:VAL:HA	1:A:78:TYR:HE2	2	0.44
(2,372)	1:A:77:VAL:HA	1:A:78:TYR:HE1	8	0.44
(2,372)	1:A:77:VAL:HA	1:A:78:TYR:HE2	8	0.44
(2,208)	1:A:40:TYR:HD1	1:A:41:GLU:H	3	0.44
(2,208)	1:A:40:TYR:HD2	1:A:41:GLU:H	3	0.44
(2,183)	1:A:29:TRP:HE1	1:A:72:MET:HE1	2	0.44
(2,183)	1:A:29:TRP:HE1	1:A:72:MET:HE2	2	0.44
(2,183)	1:A:29:TRP:HE1	1:A:72:MET:HE3	2	0.44
(2,118)	1:A:25:PHE:HD1	1:A:67:GLU:HG2	10	0.44
(2,118)	1:A:25:PHE:HD1	1:A:67:GLU:HG3	10	0.44
(2,118)	1:A:25:PHE:HD2	1:A:67:GLU:HG2	10	0.44

Continued on next page...

Continued from previous page...

Key	Atom-1	Atom-2	Model ID	Violation (Å)
(2,118)	1:A:25:PHE:HD2	1:A:67:GLU:HG3	10	0.44
(1,97)	1:A:5:VAL:H	1:A:5:VAL:HG11	1	0.44
(1,97)	1:A:5:VAL:H	1:A:5:VAL:HG12	1	0.44
(1,97)	1:A:5:VAL:H	1:A:5:VAL:HG13	1	0.44
(1,97)	1:A:5:VAL:H	1:A:5:VAL:HG11	4	0.44
(1,97)	1:A:5:VAL:H	1:A:5:VAL:HG12	4	0.44
(1,97)	1:A:5:VAL:H	1:A:5:VAL:HG13	4	0.44
(1,89)	1:A:5:VAL:HG21	1:A:7:SER:H	5	0.44
(1,89)	1:A:5:VAL:HG22	1:A:7:SER:H	5	0.44
(1,89)	1:A:5:VAL:HG23	1:A:7:SER:H	5	0.44
(1,555)	1:A:20:LEU:HA	1:A:20:LEU:HD11	6	0.44
(1,555)	1:A:20:LEU:HA	1:A:20:LEU:HD12	6	0.44
(1,555)	1:A:20:LEU:HA	1:A:20:LEU:HD13	6	0.44
(1,555)	1:A:20:LEU:HA	1:A:20:LEU:HD11	8	0.44
(1,555)	1:A:20:LEU:HA	1:A:20:LEU:HD12	8	0.44
(1,555)	1:A:20:LEU:HA	1:A:20:LEU:HD13	8	0.44
(1,503)	1:A:18:ASN:HD21	1:A:21:VAL:HG21	9	0.44
(1,503)	1:A:18:ASN:HD21	1:A:21:VAL:HG22	9	0.44
(1,503)	1:A:18:ASN:HD21	1:A:21:VAL:HG23	9	0.44
(1,2544)	1:A:27:ALA:HB1	1:A:29:TRP:HE1	5	0.44
(1,2544)	1:A:27:ALA:HB2	1:A:29:TRP:HE1	5	0.44
(1,2544)	1:A:27:ALA:HB3	1:A:29:TRP:HE1	5	0.44
(1,2544)	1:A:27:ALA:HB1	1:A:29:TRP:HE1	8	0.44
(1,2544)	1:A:27:ALA:HB2	1:A:29:TRP:HE1	8	0.44
(1,2544)	1:A:27:ALA:HB3	1:A:29:TRP:HE1	8	0.44
(1,2429)	1:A:99:ILE:H	1:A:99:ILE:HG21	2	0.44
(1,2429)	1:A:99:ILE:H	1:A:99:ILE:HG22	2	0.44
(1,2429)	1:A:99:ILE:H	1:A:99:ILE:HG23	2	0.44
(1,2429)	1:A:99:ILE:H	1:A:99:ILE:HG21	4	0.44
(1,2429)	1:A:99:ILE:H	1:A:99:ILE:HG22	4	0.44
(1,2429)	1:A:99:ILE:H	1:A:99:ILE:HG23	4	0.44
(1,2310)	1:A:95:LEU:HD21	1:A:95:LEU:HB3	6	0.44
(1,2310)	1:A:95:LEU:HD22	1:A:95:LEU:HB3	6	0.44
(1,2310)	1:A:95:LEU:HD23	1:A:95:LEU:HB3	6	0.44
(1,2310)	1:A:95:LEU:HD21	1:A:95:LEU:HB3	9	0.44
(1,2310)	1:A:95:LEU:HD22	1:A:95:LEU:HB3	9	0.44
(1,2310)	1:A:95:LEU:HD23	1:A:95:LEU:HB3	9	0.44
(1,2310)	1:A:95:LEU:HD21	1:A:95:LEU:HB3	10	0.44
(1,2310)	1:A:95:LEU:HD22	1:A:95:LEU:HB3	10	0.44
(1,2310)	1:A:95:LEU:HD23	1:A:95:LEU:HB3	10	0.44
(1,2123)	1:A:84:VAL:HG11	1:A:85:ASP:H	1	0.44
(1,2123)	1:A:84:VAL:HG12	1:A:85:ASP:H	1	0.44

Continued on next page...

Continued from previous page...

Key	Atom-1	Atom-2	Model ID	Violation (Å)
(1,2123)	1:A:84:VAL:HG13	1:A:85:ASP:H	1	0.44
(1,2123)	1:A:84:VAL:HG11	1:A:85:ASP:H	5	0.44
(1,2123)	1:A:84:VAL:HG12	1:A:85:ASP:H	5	0.44
(1,2123)	1:A:84:VAL:HG13	1:A:85:ASP:H	5	0.44
(1,2123)	1:A:84:VAL:HG11	1:A:85:ASP:H	6	0.44
(1,2123)	1:A:84:VAL:HG12	1:A:85:ASP:H	6	0.44
(1,2123)	1:A:84:VAL:HG13	1:A:85:ASP:H	6	0.44
(1,1916)	1:A:76:LYS:HD2	1:A:86:THR:HG21	9	0.44
(1,1916)	1:A:76:LYS:HD2	1:A:86:THR:HG22	9	0.44
(1,1916)	1:A:76:LYS:HD2	1:A:86:THR:HG23	9	0.44
(1,1916)	1:A:76:LYS:HD3	1:A:86:THR:HG21	9	0.44
(1,1916)	1:A:76:LYS:HD3	1:A:86:THR:HG22	9	0.44
(1,1916)	1:A:76:LYS:HD3	1:A:86:THR:HG23	9	0.44
(1,1890)	1:A:75:PHE:HZ	1:A:90:ALA:HB1	3	0.44
(1,1890)	1:A:75:PHE:HZ	1:A:90:ALA:HB2	3	0.44
(1,1890)	1:A:75:PHE:HZ	1:A:90:ALA:HB3	3	0.44
(1,1359)	1:A:47:TYR:HB3	1:A:103:ALA:HB1	8	0.44
(1,1359)	1:A:47:TYR:HB3	1:A:103:ALA:HB2	8	0.44
(1,1359)	1:A:47:TYR:HB3	1:A:103:ALA:HB3	8	0.44
(1,1359)	1:A:47:TYR:HB3	1:A:103:ALA:HB1	9	0.44
(1,1359)	1:A:47:TYR:HB3	1:A:103:ALA:HB2	9	0.44
(1,1359)	1:A:47:TYR:HB3	1:A:103:ALA:HB3	9	0.44
(1,1334)	1:A:46:THR:HG21	1:A:47:TYR:HD1	10	0.44
(1,1334)	1:A:46:THR:HG21	1:A:47:TYR:HD2	10	0.44
(1,1334)	1:A:46:THR:HG22	1:A:47:TYR:HD1	10	0.44
(1,1334)	1:A:46:THR:HG22	1:A:47:TYR:HD2	10	0.44
(1,1334)	1:A:46:THR:HG23	1:A:47:TYR:HD1	10	0.44
(1,1334)	1:A:46:THR:HG23	1:A:47:TYR:HD2	10	0.44
(1,1267)	1:A:43:CYS:HB3	1:A:99:ILE:HG21	3	0.44
(1,1267)	1:A:43:CYS:HB3	1:A:99:ILE:HG22	3	0.44
(1,1267)	1:A:43:CYS:HB3	1:A:99:ILE:HG23	3	0.44
(1,1267)	1:A:43:CYS:HB3	1:A:99:ILE:HG21	7	0.44
(1,1267)	1:A:43:CYS:HB3	1:A:99:ILE:HG22	7	0.44
(1,1267)	1:A:43:CYS:HB3	1:A:99:ILE:HG23	7	0.44
(1,1267)	1:A:43:CYS:HB3	1:A:99:ILE:HG21	9	0.44
(1,1267)	1:A:43:CYS:HB3	1:A:99:ILE:HG22	9	0.44
(1,1267)	1:A:43:CYS:HB3	1:A:99:ILE:HG23	9	0.44
(2,266)	1:A:50:MET:H	1:A:52:PHE:HD1	4	0.43
(2,266)	1:A:50:MET:H	1:A:52:PHE:HD2	4	0.43
(2,208)	1:A:40:TYR:HD1	1:A:41:GLU:H	6	0.43
(2,208)	1:A:40:TYR:HD2	1:A:41:GLU:H	6	0.43
(2,118)	1:A:25:PHE:HD1	1:A:67:GLU:HG2	7	0.43

Continued on next page...

Continued from previous page...

Key	Atom-1	Atom-2	Model ID	Violation (Å)
(2,118)	1:A:25:PHE:HD1	1:A:67:GLU:HG3	7	0.43
(2,118)	1:A:25:PHE:HD2	1:A:67:GLU:HG2	7	0.43
(2,118)	1:A:25:PHE:HD2	1:A:67:GLU:HG3	7	0.43
(1,97)	1:A:5:VAL:H	1:A:5:VAL:HG11	3	0.43
(1,97)	1:A:5:VAL:H	1:A:5:VAL:HG12	3	0.43
(1,97)	1:A:5:VAL:H	1:A:5:VAL:HG13	3	0.43
(1,89)	1:A:5:VAL:HG21	1:A:7:SER:H	4	0.43
(1,89)	1:A:5:VAL:HG22	1:A:7:SER:H	4	0.43
(1,89)	1:A:5:VAL:HG23	1:A:7:SER:H	4	0.43
(1,747)	1:A:23:VAL:HG21	1:A:53:ILE:HG12	7	0.43
(1,747)	1:A:23:VAL:HG22	1:A:53:ILE:HG12	7	0.43
(1,747)	1:A:23:VAL:HG23	1:A:53:ILE:HG12	7	0.43
(1,587)	1:A:20:LEU:HG	1:A:77:VAL:HG11	10	0.43
(1,587)	1:A:20:LEU:HG	1:A:77:VAL:HG12	10	0.43
(1,587)	1:A:20:LEU:HG	1:A:77:VAL:HG13	10	0.43
(1,555)	1:A:20:LEU:HA	1:A:20:LEU:HD11	5	0.43
(1,555)	1:A:20:LEU:HA	1:A:20:LEU:HD12	5	0.43
(1,555)	1:A:20:LEU:HA	1:A:20:LEU:HD13	5	0.43
(1,390)	1:A:15:ILE:HD11	1:A:78:TYR:HE1	6	0.43
(1,390)	1:A:15:ILE:HD11	1:A:78:TYR:HE2	6	0.43
(1,390)	1:A:15:ILE:HD12	1:A:78:TYR:HE1	6	0.43
(1,390)	1:A:15:ILE:HD12	1:A:78:TYR:HE2	6	0.43
(1,390)	1:A:15:ILE:HD13	1:A:78:TYR:HE1	6	0.43
(1,390)	1:A:15:ILE:HD13	1:A:78:TYR:HE2	6	0.43
(1,2544)	1:A:27:ALA:HB1	1:A:29:TRP:HE1	4	0.43
(1,2544)	1:A:27:ALA:HB2	1:A:29:TRP:HE1	4	0.43
(1,2544)	1:A:27:ALA:HB3	1:A:29:TRP:HE1	4	0.43
(1,2544)	1:A:27:ALA:HB1	1:A:29:TRP:HE1	10	0.43
(1,2544)	1:A:27:ALA:HB2	1:A:29:TRP:HE1	10	0.43
(1,2544)	1:A:27:ALA:HB3	1:A:29:TRP:HE1	10	0.43
(1,2310)	1:A:95:LEU:HD21	1:A:95:LEU:HB3	4	0.43
(1,2310)	1:A:95:LEU:HD22	1:A:95:LEU:HB3	4	0.43
(1,2310)	1:A:95:LEU:HD23	1:A:95:LEU:HB3	4	0.43
(1,2123)	1:A:84:VAL:HG11	1:A:85:ASP:H	3	0.43
(1,2123)	1:A:84:VAL:HG12	1:A:85:ASP:H	3	0.43
(1,2123)	1:A:84:VAL:HG13	1:A:85:ASP:H	3	0.43
(1,2123)	1:A:84:VAL:HG11	1:A:85:ASP:H	4	0.43
(1,2123)	1:A:84:VAL:HG12	1:A:85:ASP:H	4	0.43
(1,2123)	1:A:84:VAL:HG13	1:A:85:ASP:H	4	0.43
(1,2123)	1:A:84:VAL:HG11	1:A:85:ASP:H	8	0.43
(1,2123)	1:A:84:VAL:HG12	1:A:85:ASP:H	8	0.43
(1,2123)	1:A:84:VAL:HG13	1:A:85:ASP:H	8	0.43

Continued on next page...

Continued from previous page...

Key	Atom-1	Atom-2	Model ID	Violation (Å)
(1,1933)	1:A:77:VAL:HB	1:A:84:VAL:HG11	4	0.43
(1,1933)	1:A:77:VAL:HB	1:A:84:VAL:HG12	4	0.43
(1,1933)	1:A:77:VAL:HB	1:A:84:VAL:HG13	4	0.43
(1,1933)	1:A:77:VAL:HB	1:A:84:VAL:HG11	6	0.43
(1,1933)	1:A:77:VAL:HB	1:A:84:VAL:HG12	6	0.43
(1,1933)	1:A:77:VAL:HB	1:A:84:VAL:HG13	6	0.43
(1,1359)	1:A:47:TYR:HB3	1:A:103:ALA:HB1	6	0.43
(1,1359)	1:A:47:TYR:HB3	1:A:103:ALA:HB2	6	0.43
(1,1359)	1:A:47:TYR:HB3	1:A:103:ALA:HB3	6	0.43
(1,1358)	1:A:47:TYR:HB3	1:A:99:ILE:HD11	2	0.43
(1,1358)	1:A:47:TYR:HB3	1:A:99:ILE:HD12	2	0.43
(1,1358)	1:A:47:TYR:HB3	1:A:99:ILE:HD13	2	0.43
(1,1334)	1:A:46:THR:HG21	1:A:47:TYR:HD1	8	0.43
(1,1334)	1:A:46:THR:HG21	1:A:47:TYR:HD2	8	0.43
(1,1334)	1:A:46:THR:HG22	1:A:47:TYR:HD1	8	0.43
(1,1334)	1:A:46:THR:HG22	1:A:47:TYR:HD2	8	0.43
(1,1334)	1:A:46:THR:HG23	1:A:47:TYR:HD1	8	0.43
(1,1334)	1:A:46:THR:HG23	1:A:47:TYR:HD2	8	0.43
(2,83)	1:A:12:ASP:HB2	1:A:66:LYS:HE2	5	0.42
(2,83)	1:A:12:ASP:HB2	1:A:66:LYS:HE3	5	0.42
(2,296)	1:A:67:GLU:HB3	1:A:78:TYR:HE1	4	0.42
(2,296)	1:A:67:GLU:HB3	1:A:78:TYR:HE2	4	0.42
(2,208)	1:A:40:TYR:HD1	1:A:41:GLU:H	4	0.42
(2,208)	1:A:40:TYR:HD2	1:A:41:GLU:H	4	0.42
(2,208)	1:A:40:TYR:HD1	1:A:41:GLU:H	5	0.42
(2,208)	1:A:40:TYR:HD2	1:A:41:GLU:H	5	0.42
(2,208)	1:A:40:TYR:HD1	1:A:41:GLU:H	10	0.42
(2,208)	1:A:40:TYR:HD2	1:A:41:GLU:H	10	0.42
(2,183)	1:A:29:TRP:HE1	1:A:72:MET:HE1	6	0.42
(2,183)	1:A:29:TRP:HE1	1:A:72:MET:HE2	6	0.42
(2,183)	1:A:29:TRP:HE1	1:A:72:MET:HE3	6	0.42
(2,134)	1:A:26:PHE:HA	1:A:75:PHE:HE1	10	0.42
(2,134)	1:A:26:PHE:HA	1:A:75:PHE:HE2	10	0.42
(1,97)	1:A:5:VAL:H	1:A:5:VAL:HG11	6	0.42
(1,97)	1:A:5:VAL:H	1:A:5:VAL:HG12	6	0.42
(1,97)	1:A:5:VAL:H	1:A:5:VAL:HG13	6	0.42
(1,97)	1:A:5:VAL:H	1:A:5:VAL:HG11	9	0.42
(1,97)	1:A:5:VAL:H	1:A:5:VAL:HG12	9	0.42
(1,97)	1:A:5:VAL:H	1:A:5:VAL:HG13	9	0.42
(1,89)	1:A:5:VAL:HG21	1:A:7:SER:H	2	0.42
(1,89)	1:A:5:VAL:HG22	1:A:7:SER:H	2	0.42
(1,89)	1:A:5:VAL:HG23	1:A:7:SER:H	2	0.42

Continued on next page...

Continued from previous page...

Key	Atom-1	Atom-2	Model ID	Violation (Å)
(1,89)	1:A:5:VAL:HG21	1:A:7:SER:H	6	0.42
(1,89)	1:A:5:VAL:HG22	1:A:7:SER:H	6	0.42
(1,89)	1:A:5:VAL:HG23	1:A:7:SER:H	6	0.42
(1,89)	1:A:5:VAL:HG21	1:A:7:SER:H	10	0.42
(1,89)	1:A:5:VAL:HG22	1:A:7:SER:H	10	0.42
(1,89)	1:A:5:VAL:HG23	1:A:7:SER:H	10	0.42
(1,749)	1:A:23:VAL:HG21	1:A:54:LYS:HA	3	0.42
(1,749)	1:A:23:VAL:HG22	1:A:54:LYS:HA	3	0.42
(1,749)	1:A:23:VAL:HG23	1:A:54:LYS:HA	3	0.42
(1,712)	1:A:23:VAL:HG11	1:A:25:PHE:HZ	7	0.42
(1,712)	1:A:23:VAL:HG12	1:A:25:PHE:HZ	7	0.42
(1,712)	1:A:23:VAL:HG13	1:A:25:PHE:HZ	7	0.42
(1,555)	1:A:20:LEU:HA	1:A:20:LEU:HD11	2	0.42
(1,555)	1:A:20:LEU:HA	1:A:20:LEU:HD12	2	0.42
(1,555)	1:A:20:LEU:HA	1:A:20:LEU:HD13	2	0.42
(1,412)	1:A:15:ILE:HG21	1:A:15:ILE:HD11	9	0.42
(1,412)	1:A:15:ILE:HG21	1:A:15:ILE:HD12	9	0.42
(1,412)	1:A:15:ILE:HG21	1:A:15:ILE:HD13	9	0.42
(1,412)	1:A:15:ILE:HG22	1:A:15:ILE:HD11	9	0.42
(1,412)	1:A:15:ILE:HG22	1:A:15:ILE:HD12	9	0.42
(1,412)	1:A:15:ILE:HG22	1:A:15:ILE:HD13	9	0.42
(1,412)	1:A:15:ILE:HG23	1:A:15:ILE:HD11	9	0.42
(1,412)	1:A:15:ILE:HG23	1:A:15:ILE:HD12	9	0.42
(1,412)	1:A:15:ILE:HG23	1:A:15:ILE:HD13	9	0.42
(1,366)	1:A:15:ILE:HD11	1:A:15:ILE:HG21	9	0.42
(1,366)	1:A:15:ILE:HD11	1:A:15:ILE:HG22	9	0.42
(1,366)	1:A:15:ILE:HD11	1:A:15:ILE:HG23	9	0.42
(1,366)	1:A:15:ILE:HD12	1:A:15:ILE:HG21	9	0.42
(1,366)	1:A:15:ILE:HD12	1:A:15:ILE:HG22	9	0.42
(1,366)	1:A:15:ILE:HD12	1:A:15:ILE:HG23	9	0.42
(1,366)	1:A:15:ILE:HD13	1:A:15:ILE:HG21	9	0.42
(1,366)	1:A:15:ILE:HD13	1:A:15:ILE:HG22	9	0.42
(1,366)	1:A:15:ILE:HD13	1:A:15:ILE:HG23	9	0.42
(1,2238)	1:A:91:ASN:HB2	1:A:94:ALA:HB1	1	0.42
(1,2238)	1:A:91:ASN:HB2	1:A:94:ALA:HB2	1	0.42
(1,2238)	1:A:91:ASN:HB2	1:A:94:ALA:HB3	1	0.42
(1,2123)	1:A:84:VAL:HG11	1:A:85:ASP:H	7	0.42
(1,2123)	1:A:84:VAL:HG12	1:A:85:ASP:H	7	0.42
(1,2123)	1:A:84:VAL:HG13	1:A:85:ASP:H	7	0.42
(1,2123)	1:A:84:VAL:HG11	1:A:85:ASP:H	9	0.42
(1,2123)	1:A:84:VAL:HG12	1:A:85:ASP:H	9	0.42
(1,2123)	1:A:84:VAL:HG13	1:A:85:ASP:H	9	0.42

Continued on next page...

Continued from previous page...

Key	Atom-1	Atom-2	Model ID	Violation (Å)
(1,1933)	1:A:77:VAL:HB	1:A:84:VAL:HG11	1	0.42
(1,1933)	1:A:77:VAL:HB	1:A:84:VAL:HG12	1	0.42
(1,1933)	1:A:77:VAL:HB	1:A:84:VAL:HG13	1	0.42
(1,1933)	1:A:77:VAL:HB	1:A:84:VAL:HG11	8	0.42
(1,1933)	1:A:77:VAL:HB	1:A:84:VAL:HG12	8	0.42
(1,1933)	1:A:77:VAL:HB	1:A:84:VAL:HG13	8	0.42
(1,1933)	1:A:77:VAL:HB	1:A:84:VAL:HG11	9	0.42
(1,1933)	1:A:77:VAL:HB	1:A:84:VAL:HG12	9	0.42
(1,1933)	1:A:77:VAL:HB	1:A:84:VAL:HG13	9	0.42
(1,1719)	1:A:68:ASN:HD21	1:A:70:THR:HG21	10	0.42
(1,1719)	1:A:68:ASN:HD21	1:A:70:THR:HG22	10	0.42
(1,1719)	1:A:68:ASN:HD21	1:A:70:THR:HG23	10	0.42
(1,1359)	1:A:47:TYR:HB3	1:A:103:ALA:HB1	4	0.42
(1,1359)	1:A:47:TYR:HB3	1:A:103:ALA:HB2	4	0.42
(1,1359)	1:A:47:TYR:HB3	1:A:103:ALA:HB3	4	0.42
(1,1359)	1:A:47:TYR:HB3	1:A:103:ALA:HB1	7	0.42
(1,1359)	1:A:47:TYR:HB3	1:A:103:ALA:HB2	7	0.42
(1,1359)	1:A:47:TYR:HB3	1:A:103:ALA:HB3	7	0.42
(1,1359)	1:A:47:TYR:HB3	1:A:103:ALA:HB1	10	0.42
(1,1359)	1:A:47:TYR:HB3	1:A:103:ALA:HB2	10	0.42
(1,1359)	1:A:47:TYR:HB3	1:A:103:ALA:HB3	10	0.42
(1,1358)	1:A:47:TYR:HB3	1:A:99:ILE:HD11	5	0.42
(1,1358)	1:A:47:TYR:HB3	1:A:99:ILE:HD12	5	0.42
(1,1358)	1:A:47:TYR:HB3	1:A:99:ILE:HD13	5	0.42
(1,1358)	1:A:47:TYR:HB3	1:A:99:ILE:HD11	8	0.42
(1,1358)	1:A:47:TYR:HB3	1:A:99:ILE:HD12	8	0.42
(1,1358)	1:A:47:TYR:HB3	1:A:99:ILE:HD13	8	0.42
(1,1267)	1:A:43:CYS:HB3	1:A:99:ILE:HG21	6	0.42
(1,1267)	1:A:43:CYS:HB3	1:A:99:ILE:HG22	6	0.42
(1,1267)	1:A:43:CYS:HB3	1:A:99:ILE:HG23	6	0.42
(1,1036)	1:A:36:ILE:HA	1:A:36:ILE:HD11	5	0.42
(1,1036)	1:A:36:ILE:HA	1:A:36:ILE:HD12	5	0.42
(1,1036)	1:A:36:ILE:HA	1:A:36:ILE:HD13	5	0.42
(2,9)	1:A:1:SER:HB2	1:A:2:VAL:HA	6	0.41
(2,9)	1:A:1:SER:HB3	1:A:2:VAL:HA	6	0.41
(2,287)	1:A:57:VAL:HG21	1:A:61:SER:HA	6	0.41
(2,287)	1:A:57:VAL:HG22	1:A:61:SER:HA	6	0.41
(2,287)	1:A:57:VAL:HG23	1:A:61:SER:HA	6	0.41
(2,244)	1:A:47:TYR:HA	1:A:52:PHE:HE1	9	0.41
(2,244)	1:A:47:TYR:HA	1:A:52:PHE:HE2	9	0.41
(2,208)	1:A:40:TYR:HD1	1:A:41:GLU:H	1	0.41
(2,208)	1:A:40:TYR:HD2	1:A:41:GLU:H	1	0.41

Continued on next page...

Continued from previous page...

Key	Atom-1	Atom-2	Model ID	Violation (Å)
(1,97)	1:A:5:VAL:H	1:A:5:VAL:HG11	2	0.41
(1,97)	1:A:5:VAL:H	1:A:5:VAL:HG12	2	0.41
(1,97)	1:A:5:VAL:H	1:A:5:VAL:HG13	2	0.41
(1,97)	1:A:5:VAL:H	1:A:5:VAL:HG11	5	0.41
(1,97)	1:A:5:VAL:H	1:A:5:VAL:HG12	5	0.41
(1,97)	1:A:5:VAL:H	1:A:5:VAL:HG13	5	0.41
(1,97)	1:A:5:VAL:H	1:A:5:VAL:HG11	7	0.41
(1,97)	1:A:5:VAL:H	1:A:5:VAL:HG12	7	0.41
(1,97)	1:A:5:VAL:H	1:A:5:VAL:HG13	7	0.41
(1,97)	1:A:5:VAL:H	1:A:5:VAL:HG11	8	0.41
(1,97)	1:A:5:VAL:H	1:A:5:VAL:HG12	8	0.41
(1,97)	1:A:5:VAL:H	1:A:5:VAL:HG13	8	0.41
(1,89)	1:A:5:VAL:HG21	1:A:7:SER:H	1	0.41
(1,89)	1:A:5:VAL:HG22	1:A:7:SER:H	1	0.41
(1,89)	1:A:5:VAL:HG23	1:A:7:SER:H	1	0.41
(1,89)	1:A:5:VAL:HG21	1:A:7:SER:H	8	0.41
(1,89)	1:A:5:VAL:HG22	1:A:7:SER:H	8	0.41
(1,89)	1:A:5:VAL:HG23	1:A:7:SER:H	8	0.41
(1,89)	1:A:5:VAL:HG21	1:A:7:SER:H	9	0.41
(1,89)	1:A:5:VAL:HG22	1:A:7:SER:H	9	0.41
(1,89)	1:A:5:VAL:HG23	1:A:7:SER:H	9	0.41
(1,731)	1:A:23:VAL:HG11	1:A:78:TYR:HE1	3	0.41
(1,731)	1:A:23:VAL:HG11	1:A:78:TYR:HE2	3	0.41
(1,731)	1:A:23:VAL:HG12	1:A:78:TYR:HE1	3	0.41
(1,731)	1:A:23:VAL:HG12	1:A:78:TYR:HE2	3	0.41
(1,731)	1:A:23:VAL:HG13	1:A:78:TYR:HE1	3	0.41
(1,731)	1:A:23:VAL:HG13	1:A:78:TYR:HE2	3	0.41
(1,707)	1:A:23:VAL:HG11	1:A:24:ASP:H	8	0.41
(1,707)	1:A:23:VAL:HG12	1:A:24:ASP:H	8	0.41
(1,707)	1:A:23:VAL:HG13	1:A:24:ASP:H	8	0.41
(1,412)	1:A:15:ILE:HG21	1:A:15:ILE:HD11	7	0.41
(1,412)	1:A:15:ILE:HG21	1:A:15:ILE:HD12	7	0.41
(1,412)	1:A:15:ILE:HG21	1:A:15:ILE:HD13	7	0.41
(1,412)	1:A:15:ILE:HG22	1:A:15:ILE:HD11	7	0.41
(1,412)	1:A:15:ILE:HG22	1:A:15:ILE:HD12	7	0.41
(1,412)	1:A:15:ILE:HG22	1:A:15:ILE:HD13	7	0.41
(1,412)	1:A:15:ILE:HG23	1:A:15:ILE:HD11	7	0.41
(1,412)	1:A:15:ILE:HG23	1:A:15:ILE:HD12	7	0.41
(1,412)	1:A:15:ILE:HG23	1:A:15:ILE:HD13	7	0.41
(1,366)	1:A:15:ILE:HD11	1:A:15:ILE:HG21	7	0.41
(1,366)	1:A:15:ILE:HD11	1:A:15:ILE:HG22	7	0.41
(1,366)	1:A:15:ILE:HD11	1:A:15:ILE:HG23	7	0.41

Continued on next page...

Continued from previous page...

Key	Atom-1	Atom-2	Model ID	Violation (Å)
(1,366)	1:A:15:ILE:HD12	1:A:15:ILE:HG21	7	0.41
(1,366)	1:A:15:ILE:HD12	1:A:15:ILE:HG22	7	0.41
(1,366)	1:A:15:ILE:HD12	1:A:15:ILE:HG23	7	0.41
(1,366)	1:A:15:ILE:HD13	1:A:15:ILE:HG21	7	0.41
(1,366)	1:A:15:ILE:HD13	1:A:15:ILE:HG22	7	0.41
(1,366)	1:A:15:ILE:HD13	1:A:15:ILE:HG23	7	0.41
(1,30)	1:A:2:VAL:H	1:A:2:VAL:HG21	7	0.41
(1,30)	1:A:2:VAL:H	1:A:2:VAL:HG22	7	0.41
(1,30)	1:A:2:VAL:H	1:A:2:VAL:HG23	7	0.41
(1,30)	1:A:2:VAL:H	1:A:2:VAL:HG21	8	0.41
(1,30)	1:A:2:VAL:H	1:A:2:VAL:HG22	8	0.41
(1,30)	1:A:2:VAL:H	1:A:2:VAL:HG23	8	0.41
(1,2238)	1:A:91:ASN:HB2	1:A:94:ALA:HB1	4	0.41
(1,2238)	1:A:91:ASN:HB2	1:A:94:ALA:HB2	4	0.41
(1,2238)	1:A:91:ASN:HB2	1:A:94:ALA:HB3	4	0.41
(1,2238)	1:A:91:ASN:HB2	1:A:94:ALA:HB1	5	0.41
(1,2238)	1:A:91:ASN:HB2	1:A:94:ALA:HB2	5	0.41
(1,2238)	1:A:91:ASN:HB2	1:A:94:ALA:HB3	5	0.41
(1,2238)	1:A:91:ASN:HB2	1:A:94:ALA:HB1	6	0.41
(1,2238)	1:A:91:ASN:HB2	1:A:94:ALA:HB2	6	0.41
(1,2238)	1:A:91:ASN:HB2	1:A:94:ALA:HB3	6	0.41
(1,2238)	1:A:91:ASN:HB2	1:A:94:ALA:HB1	9	0.41
(1,2238)	1:A:91:ASN:HB2	1:A:94:ALA:HB2	9	0.41
(1,2238)	1:A:91:ASN:HB2	1:A:94:ALA:HB3	9	0.41
(1,2238)	1:A:91:ASN:HB2	1:A:94:ALA:HB1	10	0.41
(1,2238)	1:A:91:ASN:HB2	1:A:94:ALA:HB2	10	0.41
(1,2238)	1:A:91:ASN:HB2	1:A:94:ALA:HB3	10	0.41
(1,2123)	1:A:84:VAL:HG11	1:A:85:ASP:H	10	0.41
(1,2123)	1:A:84:VAL:HG12	1:A:85:ASP:H	10	0.41
(1,2123)	1:A:84:VAL:HG13	1:A:85:ASP:H	10	0.41
(1,1933)	1:A:77:VAL:HB	1:A:84:VAL:HG11	3	0.41
(1,1933)	1:A:77:VAL:HB	1:A:84:VAL:HG12	3	0.41
(1,1933)	1:A:77:VAL:HB	1:A:84:VAL:HG13	3	0.41
(1,1933)	1:A:77:VAL:HB	1:A:84:VAL:HG11	5	0.41
(1,1933)	1:A:77:VAL:HB	1:A:84:VAL:HG12	5	0.41
(1,1933)	1:A:77:VAL:HB	1:A:84:VAL:HG13	5	0.41
(1,1916)	1:A:76:LYS:HD2	1:A:86:THR:HG21	3	0.41
(1,1916)	1:A:76:LYS:HD2	1:A:86:THR:HG22	3	0.41
(1,1916)	1:A:76:LYS:HD2	1:A:86:THR:HG23	3	0.41
(1,1916)	1:A:76:LYS:HD3	1:A:86:THR:HG21	3	0.41
(1,1916)	1:A:76:LYS:HD3	1:A:86:THR:HG22	3	0.41
(1,1916)	1:A:76:LYS:HD3	1:A:86:THR:HG23	3	0.41

Continued on next page...

Continued from previous page...

Key	Atom-1	Atom-2	Model ID	Violation (Å)
(1,1851)	1:A:75:PHE:HB2	1:A:87:LEU:HD21	1	0.41
(1,1851)	1:A:75:PHE:HB2	1:A:87:LEU:HD22	1	0.41
(1,1851)	1:A:75:PHE:HB2	1:A:87:LEU:HD23	1	0.41
(1,1851)	1:A:75:PHE:HB2	1:A:87:LEU:HD21	2	0.41
(1,1851)	1:A:75:PHE:HB2	1:A:87:LEU:HD22	2	0.41
(1,1851)	1:A:75:PHE:HB2	1:A:87:LEU:HD23	2	0.41
(1,1851)	1:A:75:PHE:HB2	1:A:87:LEU:HD21	3	0.41
(1,1851)	1:A:75:PHE:HB2	1:A:87:LEU:HD22	3	0.41
(1,1851)	1:A:75:PHE:HB2	1:A:87:LEU:HD23	3	0.41
(1,1851)	1:A:75:PHE:HB2	1:A:87:LEU:HD21	4	0.41
(1,1851)	1:A:75:PHE:HB2	1:A:87:LEU:HD22	4	0.41
(1,1851)	1:A:75:PHE:HB2	1:A:87:LEU:HD23	4	0.41
(1,1851)	1:A:75:PHE:HB2	1:A:87:LEU:HD21	6	0.41
(1,1851)	1:A:75:PHE:HB2	1:A:87:LEU:HD22	6	0.41
(1,1851)	1:A:75:PHE:HB2	1:A:87:LEU:HD23	6	0.41
(1,1851)	1:A:75:PHE:HB2	1:A:87:LEU:HD21	8	0.41
(1,1851)	1:A:75:PHE:HB2	1:A:87:LEU:HD22	8	0.41
(1,1851)	1:A:75:PHE:HB2	1:A:87:LEU:HD23	8	0.41
(1,1392)	1:A:47:TYR:HE1	1:A:99:ILE:HG21	5	0.41
(1,1392)	1:A:47:TYR:HE1	1:A:99:ILE:HG22	5	0.41
(1,1392)	1:A:47:TYR:HE1	1:A:99:ILE:HG23	5	0.41
(1,1392)	1:A:47:TYR:HE2	1:A:99:ILE:HG21	5	0.41
(1,1392)	1:A:47:TYR:HE2	1:A:99:ILE:HG22	5	0.41
(1,1392)	1:A:47:TYR:HE2	1:A:99:ILE:HG23	5	0.41
(1,1267)	1:A:43:CYS:HB3	1:A:99:ILE:HG21	1	0.41
(1,1267)	1:A:43:CYS:HB3	1:A:99:ILE:HG22	1	0.41
(1,1267)	1:A:43:CYS:HB3	1:A:99:ILE:HG23	1	0.41
(1,1036)	1:A:36:ILE:HA	1:A:36:ILE:HD11	1	0.41
(1,1036)	1:A:36:ILE:HA	1:A:36:ILE:HD12	1	0.41
(1,1036)	1:A:36:ILE:HA	1:A:36:ILE:HD13	1	0.41
(1,1036)	1:A:36:ILE:HA	1:A:36:ILE:HD11	9	0.41
(1,1036)	1:A:36:ILE:HA	1:A:36:ILE:HD12	9	0.41
(1,1036)	1:A:36:ILE:HA	1:A:36:ILE:HD13	9	0.41
(2,9)	1:A:1:SER:HB2	1:A:2:VAL:HA	1	0.4
(2,9)	1:A:1:SER:HB3	1:A:2:VAL:HA	1	0.4
(2,9)	1:A:1:SER:HB2	1:A:2:VAL:HA	8	0.4
(2,9)	1:A:1:SER:HB3	1:A:2:VAL:HA	8	0.4
(2,359)	1:A:74:THR:H	1:A:75:PHE:HE1	1	0.4
(2,359)	1:A:74:THR:H	1:A:75:PHE:HE2	1	0.4
(2,208)	1:A:40:TYR:HD1	1:A:41:GLU:H	8	0.4
(2,208)	1:A:40:TYR:HD2	1:A:41:GLU:H	8	0.4
(2,151)	1:A:27:ALA:HB1	1:A:58:ASP:HB2	2	0.4

Continued on next page...

Continued from previous page...

Key	Atom-1	Atom-2	Model ID	Violation (Å)
(2,151)	1:A:27:ALA:HB2	1:A:58:ASP:HB2	2	0.4
(2,151)	1:A:27:ALA:HB3	1:A:58:ASP:HB2	2	0.4
(2,134)	1:A:26:PHE:HA	1:A:75:PHE:HE1	4	0.4
(2,134)	1:A:26:PHE:HA	1:A:75:PHE:HE2	4	0.4
(1,89)	1:A:5:VAL:HG21	1:A:7:SER:H	3	0.4
(1,89)	1:A:5:VAL:HG22	1:A:7:SER:H	3	0.4
(1,89)	1:A:5:VAL:HG23	1:A:7:SER:H	3	0.4
(1,89)	1:A:5:VAL:HG21	1:A:7:SER:H	7	0.4
(1,89)	1:A:5:VAL:HG22	1:A:7:SER:H	7	0.4
(1,89)	1:A:5:VAL:HG23	1:A:7:SER:H	7	0.4
(1,736)	1:A:23:VAL:HG21	1:A:24:ASP:H	4	0.4
(1,736)	1:A:23:VAL:HG22	1:A:24:ASP:H	4	0.4
(1,736)	1:A:23:VAL:HG23	1:A:24:ASP:H	4	0.4
(1,692)	1:A:22:ILE:H	1:A:51:VAL:HG11	7	0.4
(1,692)	1:A:22:ILE:H	1:A:51:VAL:HG12	7	0.4
(1,692)	1:A:22:ILE:H	1:A:51:VAL:HG13	7	0.4
(1,577)	1:A:20:LEU:HD11	1:A:79:LYS:HA	5	0.4
(1,577)	1:A:20:LEU:HD12	1:A:79:LYS:HA	5	0.4
(1,577)	1:A:20:LEU:HD13	1:A:79:LYS:HA	5	0.4
(1,2351)	1:A:97:GLN:HE21	1:A:97:GLN:HB2	5	0.4
(1,2351)	1:A:97:GLN:HE21	1:A:97:GLN:HB3	5	0.4
(1,2238)	1:A:91:ASN:HB2	1:A:94:ALA:HB1	2	0.4
(1,2238)	1:A:91:ASN:HB2	1:A:94:ALA:HB2	2	0.4
(1,2238)	1:A:91:ASN:HB2	1:A:94:ALA:HB3	2	0.4
(1,2018)	1:A:78:TYR:HD1	1:A:83:SER:HA	1	0.4
(1,2018)	1:A:78:TYR:HD2	1:A:83:SER:HA	1	0.4
(1,2018)	1:A:78:TYR:HD1	1:A:83:SER:HA	6	0.4
(1,2018)	1:A:78:TYR:HD2	1:A:83:SER:HA	6	0.4
(1,1933)	1:A:77:VAL:HB	1:A:84:VAL:HG11	2	0.4
(1,1933)	1:A:77:VAL:HB	1:A:84:VAL:HG12	2	0.4
(1,1933)	1:A:77:VAL:HB	1:A:84:VAL:HG13	2	0.4
(1,1916)	1:A:76:LYS:HD2	1:A:86:THR:HG21	6	0.4
(1,1916)	1:A:76:LYS:HD2	1:A:86:THR:HG22	6	0.4
(1,1916)	1:A:76:LYS:HD2	1:A:86:THR:HG23	6	0.4
(1,1916)	1:A:76:LYS:HD3	1:A:86:THR:HG21	6	0.4
(1,1916)	1:A:76:LYS:HD3	1:A:86:THR:HG22	6	0.4
(1,1916)	1:A:76:LYS:HD3	1:A:86:THR:HG23	6	0.4
(1,1851)	1:A:75:PHE:HB2	1:A:87:LEU:HD21	5	0.4
(1,1851)	1:A:75:PHE:HB2	1:A:87:LEU:HD22	5	0.4
(1,1851)	1:A:75:PHE:HB2	1:A:87:LEU:HD23	5	0.4
(1,1812)	1:A:74:THR:HG1	1:A:74:THR:HG21	3	0.4
(1,1812)	1:A:74:THR:HG1	1:A:74:THR:HG22	3	0.4

Continued on next page...

Continued from previous page...

Key	Atom-1	Atom-2	Model ID	Violation (Å)
(1,1812)	1:A:74:THR:HG1	1:A:74:THR:HG23	3	0.4
(1,1693)	1:A:67:GLU:HB2	1:A:69:ILE:HD11	1	0.4
(1,1693)	1:A:67:GLU:HB2	1:A:69:ILE:HD12	1	0.4
(1,1693)	1:A:67:GLU:HB2	1:A:69:ILE:HD13	1	0.4
(1,1036)	1:A:36:ILE:HA	1:A:36:ILE:HD11	7	0.4
(1,1036)	1:A:36:ILE:HA	1:A:36:ILE:HD12	7	0.4
(1,1036)	1:A:36:ILE:HA	1:A:36:ILE:HD13	7	0.4
(2,9)	1:A:1:SER:HB2	1:A:2:VAL:HA	3	0.39
(2,9)	1:A:1:SER:HB3	1:A:2:VAL:HA	3	0.39
(2,83)	1:A:12:ASP:HB2	1:A:66:LYS:HE2	7	0.39
(2,83)	1:A:12:ASP:HB2	1:A:66:LYS:HE3	7	0.39
(2,372)	1:A:77:VAL:HA	1:A:78:TYR:HE1	9	0.39
(2,372)	1:A:77:VAL:HA	1:A:78:TYR:HE2	9	0.39
(2,229)	1:A:44:SER:HG	1:A:45:LYS:HD2	6	0.39
(2,229)	1:A:44:SER:HG	1:A:45:LYS:HD3	6	0.39
(2,134)	1:A:26:PHE:HA	1:A:75:PHE:HE1	2	0.39
(2,134)	1:A:26:PHE:HA	1:A:75:PHE:HE2	2	0.39
(1,692)	1:A:22:ILE:H	1:A:51:VAL:HG11	2	0.39
(1,692)	1:A:22:ILE:H	1:A:51:VAL:HG12	2	0.39
(1,692)	1:A:22:ILE:H	1:A:51:VAL:HG13	2	0.39
(1,652)	1:A:22:ILE:HD11	1:A:23:VAL:H	10	0.39
(1,652)	1:A:22:ILE:HD12	1:A:23:VAL:H	10	0.39
(1,652)	1:A:22:ILE:HD13	1:A:23:VAL:H	10	0.39
(1,555)	1:A:20:LEU:HA	1:A:20:LEU:HD11	7	0.39
(1,555)	1:A:20:LEU:HA	1:A:20:LEU:HD12	7	0.39
(1,555)	1:A:20:LEU:HA	1:A:20:LEU:HD13	7	0.39
(1,2544)	1:A:27:ALA:HB1	1:A:29:TRP:HE1	7	0.39
(1,2544)	1:A:27:ALA:HB2	1:A:29:TRP:HE1	7	0.39
(1,2544)	1:A:27:ALA:HB3	1:A:29:TRP:HE1	7	0.39
(1,2351)	1:A:97:GLN:HE21	1:A:97:GLN:HB2	1	0.39
(1,2351)	1:A:97:GLN:HE21	1:A:97:GLN:HB3	1	0.39
(1,2351)	1:A:97:GLN:HE21	1:A:97:GLN:HB2	7	0.39
(1,2351)	1:A:97:GLN:HE21	1:A:97:GLN:HB3	7	0.39
(1,2351)	1:A:97:GLN:HE21	1:A:97:GLN:HB2	8	0.39
(1,2351)	1:A:97:GLN:HE21	1:A:97:GLN:HB3	8	0.39
(1,2232)	1:A:91:ASN:HB3	1:A:94:ALA:HB1	8	0.39
(1,2232)	1:A:91:ASN:HB3	1:A:94:ALA:HB2	8	0.39
(1,2232)	1:A:91:ASN:HB3	1:A:94:ALA:HB3	8	0.39
(1,2018)	1:A:78:TYR:HD1	1:A:83:SER:HA	3	0.39
(1,2018)	1:A:78:TYR:HD2	1:A:83:SER:HA	3	0.39
(1,1980)	1:A:77:VAL:HG21	1:A:102:TYR:HD1	8	0.39
(1,1980)	1:A:77:VAL:HG21	1:A:102:TYR:HD2	8	0.39

Continued on next page...

Continued from previous page...

Key	Atom-1	Atom-2	Model ID	Violation (Å)
(1,1980)	1:A:77:VAL:HG22	1:A:102:TYR:HD1	8	0.39
(1,1980)	1:A:77:VAL:HG22	1:A:102:TYR:HD2	8	0.39
(1,1980)	1:A:77:VAL:HG23	1:A:102:TYR:HD1	8	0.39
(1,1980)	1:A:77:VAL:HG23	1:A:102:TYR:HD2	8	0.39
(1,1950)	1:A:77:VAL:HG11	1:A:84:VAL:HG11	7	0.39
(1,1950)	1:A:77:VAL:HG11	1:A:84:VAL:HG12	7	0.39
(1,1950)	1:A:77:VAL:HG11	1:A:84:VAL:HG13	7	0.39
(1,1950)	1:A:77:VAL:HG12	1:A:84:VAL:HG11	7	0.39
(1,1950)	1:A:77:VAL:HG12	1:A:84:VAL:HG12	7	0.39
(1,1950)	1:A:77:VAL:HG12	1:A:84:VAL:HG13	7	0.39
(1,1950)	1:A:77:VAL:HG13	1:A:84:VAL:HG11	7	0.39
(1,1950)	1:A:77:VAL:HG13	1:A:84:VAL:HG12	7	0.39
(1,1950)	1:A:77:VAL:HG13	1:A:84:VAL:HG13	7	0.39
(1,1916)	1:A:76:LYS:HD2	1:A:86:THR:HG21	1	0.39
(1,1916)	1:A:76:LYS:HD2	1:A:86:THR:HG22	1	0.39
(1,1916)	1:A:76:LYS:HD2	1:A:86:THR:HG23	1	0.39
(1,1916)	1:A:76:LYS:HD3	1:A:86:THR:HG21	1	0.39
(1,1916)	1:A:76:LYS:HD3	1:A:86:THR:HG22	1	0.39
(1,1916)	1:A:76:LYS:HD3	1:A:86:THR:HG23	1	0.39
(1,1916)	1:A:76:LYS:HD2	1:A:86:THR:HG21	2	0.39
(1,1916)	1:A:76:LYS:HD2	1:A:86:THR:HG22	2	0.39
(1,1916)	1:A:76:LYS:HD2	1:A:86:THR:HG23	2	0.39
(1,1916)	1:A:76:LYS:HD3	1:A:86:THR:HG21	2	0.39
(1,1916)	1:A:76:LYS:HD3	1:A:86:THR:HG22	2	0.39
(1,1916)	1:A:76:LYS:HD3	1:A:86:THR:HG23	2	0.39
(1,1741)	1:A:69:ILE:HD11	1:A:69:ILE:HG21	9	0.39
(1,1741)	1:A:69:ILE:HD11	1:A:69:ILE:HG22	9	0.39
(1,1741)	1:A:69:ILE:HD11	1:A:69:ILE:HG23	9	0.39
(1,1741)	1:A:69:ILE:HD12	1:A:69:ILE:HG21	9	0.39
(1,1741)	1:A:69:ILE:HD12	1:A:69:ILE:HG22	9	0.39
(1,1741)	1:A:69:ILE:HD12	1:A:69:ILE:HG23	9	0.39
(1,1741)	1:A:69:ILE:HD13	1:A:69:ILE:HG21	9	0.39
(1,1741)	1:A:69:ILE:HD13	1:A:69:ILE:HG22	9	0.39
(1,1741)	1:A:69:ILE:HD13	1:A:69:ILE:HG23	9	0.39
(1,1458)	1:A:51:VAL:H	1:A:51:VAL:HG21	3	0.39
(1,1458)	1:A:51:VAL:H	1:A:51:VAL:HG22	3	0.39
(1,1458)	1:A:51:VAL:H	1:A:51:VAL:HG23	3	0.39
(1,1267)	1:A:43:CYS:HB3	1:A:99:ILE:HG21	10	0.39
(1,1267)	1:A:43:CYS:HB3	1:A:99:ILE:HG22	10	0.39
(1,1267)	1:A:43:CYS:HB3	1:A:99:ILE:HG23	10	0.39
(1,1036)	1:A:36:ILE:HA	1:A:36:ILE:HD11	10	0.39
(1,1036)	1:A:36:ILE:HA	1:A:36:ILE:HD12	10	0.39

Continued on next page...

Continued from previous page...

Key	Atom-1	Atom-2	Model ID	Violation (Å)
(1,1036)	1:A:36:ILE:HA	1:A:36:ILE:HD13	10	0.39
(2,9)	1:A:1:SER:HB2	1:A:2:VAL:HA	9	0.38
(2,9)	1:A:1:SER:HB3	1:A:2:VAL:HA	9	0.38
(2,269)	1:A:52:PHE:HB3	1:A:54:LYS:HD2	8	0.38
(2,269)	1:A:52:PHE:HB3	1:A:54:LYS:HD3	8	0.38
(2,247)	1:A:47:TYR:HB2	1:A:52:PHE:HD1	7	0.38
(2,247)	1:A:47:TYR:HB2	1:A:52:PHE:HD2	7	0.38
(2,209)	1:A:40:TYR:HD1	1:A:43:CYS:H	1	0.38
(2,209)	1:A:40:TYR:HD2	1:A:43:CYS:H	1	0.38
(2,140)	1:A:26:PHE:HE1	1:A:54:LYS:HD2	8	0.38
(2,140)	1:A:26:PHE:HE1	1:A:54:LYS:HD3	8	0.38
(2,140)	1:A:26:PHE:HE2	1:A:54:LYS:HD2	8	0.38
(2,140)	1:A:26:PHE:HE2	1:A:54:LYS:HD3	8	0.38
(1,764)	1:A:23:VAL:H	1:A:23:VAL:HG11	10	0.38
(1,764)	1:A:23:VAL:H	1:A:23:VAL:HG12	10	0.38
(1,764)	1:A:23:VAL:H	1:A:23:VAL:HG13	10	0.38
(1,736)	1:A:23:VAL:HG21	1:A:24:ASP:H	7	0.38
(1,736)	1:A:23:VAL:HG22	1:A:24:ASP:H	7	0.38
(1,736)	1:A:23:VAL:HG23	1:A:24:ASP:H	7	0.38
(1,731)	1:A:23:VAL:HG11	1:A:78:TYR:HE1	7	0.38
(1,731)	1:A:23:VAL:HG11	1:A:78:TYR:HE2	7	0.38
(1,731)	1:A:23:VAL:HG12	1:A:78:TYR:HE1	7	0.38
(1,731)	1:A:23:VAL:HG12	1:A:78:TYR:HE2	7	0.38
(1,731)	1:A:23:VAL:HG13	1:A:78:TYR:HE1	7	0.38
(1,731)	1:A:23:VAL:HG13	1:A:78:TYR:HE2	7	0.38
(1,684)	1:A:22:ILE:H	1:A:22:ILE:HG21	9	0.38
(1,684)	1:A:22:ILE:H	1:A:22:ILE:HG22	9	0.38
(1,684)	1:A:22:ILE:H	1:A:22:ILE:HG23	9	0.38
(1,412)	1:A:15:ILE:HG21	1:A:15:ILE:HD11	8	0.38
(1,412)	1:A:15:ILE:HG21	1:A:15:ILE:HD12	8	0.38
(1,412)	1:A:15:ILE:HG21	1:A:15:ILE:HD13	8	0.38
(1,412)	1:A:15:ILE:HG22	1:A:15:ILE:HD11	8	0.38
(1,412)	1:A:15:ILE:HG22	1:A:15:ILE:HD12	8	0.38
(1,412)	1:A:15:ILE:HG22	1:A:15:ILE:HD13	8	0.38
(1,412)	1:A:15:ILE:HG23	1:A:15:ILE:HD11	8	0.38
(1,412)	1:A:15:ILE:HG23	1:A:15:ILE:HD12	8	0.38
(1,412)	1:A:15:ILE:HG23	1:A:15:ILE:HD13	8	0.38
(1,412)	1:A:15:ILE:HG21	1:A:15:ILE:HD11	10	0.38
(1,412)	1:A:15:ILE:HG21	1:A:15:ILE:HD12	10	0.38
(1,412)	1:A:15:ILE:HG21	1:A:15:ILE:HD13	10	0.38
(1,412)	1:A:15:ILE:HG22	1:A:15:ILE:HD11	10	0.38
(1,412)	1:A:15:ILE:HG22	1:A:15:ILE:HD12	10	0.38

Continued on next page...

Continued from previous page...

Key	Atom-1	Atom-2	Model ID	Violation (Å)
(1,412)	1:A:15:ILE:HG22	1:A:15:ILE:HD13	10	0.38
(1,412)	1:A:15:ILE:HG23	1:A:15:ILE:HD11	10	0.38
(1,412)	1:A:15:ILE:HG23	1:A:15:ILE:HD12	10	0.38
(1,412)	1:A:15:ILE:HG23	1:A:15:ILE:HD13	10	0.38
(1,366)	1:A:15:ILE:HD11	1:A:15:ILE:HG21	8	0.38
(1,366)	1:A:15:ILE:HD11	1:A:15:ILE:HG22	8	0.38
(1,366)	1:A:15:ILE:HD11	1:A:15:ILE:HG23	8	0.38
(1,366)	1:A:15:ILE:HD12	1:A:15:ILE:HG21	8	0.38
(1,366)	1:A:15:ILE:HD12	1:A:15:ILE:HG22	8	0.38
(1,366)	1:A:15:ILE:HD12	1:A:15:ILE:HG23	8	0.38
(1,366)	1:A:15:ILE:HD13	1:A:15:ILE:HG21	8	0.38
(1,366)	1:A:15:ILE:HD13	1:A:15:ILE:HG22	8	0.38
(1,366)	1:A:15:ILE:HD13	1:A:15:ILE:HG23	8	0.38
(1,366)	1:A:15:ILE:HD11	1:A:15:ILE:HG21	10	0.38
(1,366)	1:A:15:ILE:HD11	1:A:15:ILE:HG22	10	0.38
(1,366)	1:A:15:ILE:HD11	1:A:15:ILE:HG23	10	0.38
(1,366)	1:A:15:ILE:HD12	1:A:15:ILE:HG21	10	0.38
(1,366)	1:A:15:ILE:HD12	1:A:15:ILE:HG22	10	0.38
(1,366)	1:A:15:ILE:HD12	1:A:15:ILE:HG23	10	0.38
(1,366)	1:A:15:ILE:HD13	1:A:15:ILE:HG21	10	0.38
(1,366)	1:A:15:ILE:HD13	1:A:15:ILE:HG22	10	0.38
(1,366)	1:A:15:ILE:HD13	1:A:15:ILE:HG23	10	0.38
(1,2351)	1:A:97:GLN:HE21	1:A:97:GLN:HB2	10	0.38
(1,2351)	1:A:97:GLN:HE21	1:A:97:GLN:HB3	10	0.38
(1,2200)	1:A:88:LEU:HD21	1:A:88:LEU:HA	3	0.38
(1,2200)	1:A:88:LEU:HD22	1:A:88:LEU:HA	3	0.38
(1,2200)	1:A:88:LEU:HD23	1:A:88:LEU:HA	3	0.38
(1,1458)	1:A:51:VAL:H	1:A:51:VAL:HG21	1	0.38
(1,1458)	1:A:51:VAL:H	1:A:51:VAL:HG22	1	0.38
(1,1458)	1:A:51:VAL:H	1:A:51:VAL:HG23	1	0.38
(1,1458)	1:A:51:VAL:H	1:A:51:VAL:HG21	8	0.38
(1,1458)	1:A:51:VAL:H	1:A:51:VAL:HG22	8	0.38
(1,1458)	1:A:51:VAL:H	1:A:51:VAL:HG23	8	0.38
(1,1209)	1:A:40:TYR:HE1	1:A:95:LEU:HD11	5	0.38
(1,1209)	1:A:40:TYR:HE1	1:A:95:LEU:HD12	5	0.38
(1,1209)	1:A:40:TYR:HE1	1:A:95:LEU:HD13	5	0.38
(1,1209)	1:A:40:TYR:HE2	1:A:95:LEU:HD11	5	0.38
(1,1209)	1:A:40:TYR:HE2	1:A:95:LEU:HD12	5	0.38
(1,1209)	1:A:40:TYR:HE2	1:A:95:LEU:HD13	5	0.38
(2,245)	1:A:47:TYR:HB3	1:A:50:MET:HE1	6	0.37
(2,245)	1:A:47:TYR:HB3	1:A:50:MET:HE2	6	0.37
(2,245)	1:A:47:TYR:HB3	1:A:50:MET:HE3	6	0.37

Continued on next page...

Continued from previous page...

Key	Atom-1	Atom-2	Model ID	Violation (Å)
(2,209)	1:A:40:TYR:HD1	1:A:43:CYS:H	8	0.37
(2,209)	1:A:40:TYR:HD2	1:A:43:CYS:H	8	0.37
(2,131)	1:A:26:PHE:HA	1:A:72:MET:HE1	8	0.37
(2,131)	1:A:26:PHE:HA	1:A:72:MET:HE2	8	0.37
(2,131)	1:A:26:PHE:HA	1:A:72:MET:HE3	8	0.37
(1,720)	1:A:23:VAL:HG11	1:A:54:LYS:HA	8	0.37
(1,720)	1:A:23:VAL:HG12	1:A:54:LYS:HA	8	0.37
(1,720)	1:A:23:VAL:HG13	1:A:54:LYS:HA	8	0.37
(1,684)	1:A:22:ILE:H	1:A:22:ILE:HG21	1	0.37
(1,684)	1:A:22:ILE:H	1:A:22:ILE:HG22	1	0.37
(1,684)	1:A:22:ILE:H	1:A:22:ILE:HG23	1	0.37
(1,2232)	1:A:91:ASN:HB3	1:A:94:ALA:HB1	3	0.37
(1,2232)	1:A:91:ASN:HB3	1:A:94:ALA:HB2	3	0.37
(1,2232)	1:A:91:ASN:HB3	1:A:94:ALA:HB3	3	0.37
(1,2127)	1:A:84:VAL:HG21	1:A:102:TYR:HE1	6	0.37
(1,2127)	1:A:84:VAL:HG21	1:A:102:TYR:HE2	6	0.37
(1,2127)	1:A:84:VAL:HG22	1:A:102:TYR:HE1	6	0.37
(1,2127)	1:A:84:VAL:HG22	1:A:102:TYR:HE2	6	0.37
(1,2127)	1:A:84:VAL:HG23	1:A:102:TYR:HE1	6	0.37
(1,2127)	1:A:84:VAL:HG23	1:A:102:TYR:HE2	6	0.37
(1,1950)	1:A:77:VAL:HG11	1:A:84:VAL:HG11	10	0.37
(1,1950)	1:A:77:VAL:HG11	1:A:84:VAL:HG12	10	0.37
(1,1950)	1:A:77:VAL:HG11	1:A:84:VAL:HG13	10	0.37
(1,1950)	1:A:77:VAL:HG12	1:A:84:VAL:HG11	10	0.37
(1,1950)	1:A:77:VAL:HG12	1:A:84:VAL:HG12	10	0.37
(1,1950)	1:A:77:VAL:HG12	1:A:84:VAL:HG13	10	0.37
(1,1950)	1:A:77:VAL:HG13	1:A:84:VAL:HG11	10	0.37
(1,1950)	1:A:77:VAL:HG13	1:A:84:VAL:HG12	10	0.37
(1,1950)	1:A:77:VAL:HG13	1:A:84:VAL:HG13	10	0.37
(1,1916)	1:A:76:LYS:HD2	1:A:86:THR:HG21	8	0.37
(1,1916)	1:A:76:LYS:HD2	1:A:86:THR:HG22	8	0.37
(1,1916)	1:A:76:LYS:HD2	1:A:86:THR:HG23	8	0.37
(1,1916)	1:A:76:LYS:HD3	1:A:86:THR:HG21	8	0.37
(1,1916)	1:A:76:LYS:HD3	1:A:86:THR:HG22	8	0.37
(1,1916)	1:A:76:LYS:HD3	1:A:86:THR:HG23	8	0.37
(1,1851)	1:A:75:PHE:HB2	1:A:87:LEU:HD21	9	0.37
(1,1851)	1:A:75:PHE:HB2	1:A:87:LEU:HD22	9	0.37
(1,1851)	1:A:75:PHE:HB2	1:A:87:LEU:HD23	9	0.37
(1,1741)	1:A:69:ILE:HD11	1:A:69:ILE:HG21	3	0.37
(1,1741)	1:A:69:ILE:HD11	1:A:69:ILE:HG22	3	0.37
(1,1741)	1:A:69:ILE:HD11	1:A:69:ILE:HG23	3	0.37
(1,1741)	1:A:69:ILE:HD12	1:A:69:ILE:HG21	3	0.37

Continued on next page...

Continued from previous page...

Key	Atom-1	Atom-2	Model ID	Violation (Å)
(1,1741)	1:A:69:ILE:HD12	1:A:69:ILE:HG22	3	0.37
(1,1741)	1:A:69:ILE:HD12	1:A:69:ILE:HG23	3	0.37
(1,1741)	1:A:69:ILE:HD13	1:A:69:ILE:HG21	3	0.37
(1,1741)	1:A:69:ILE:HD13	1:A:69:ILE:HG22	3	0.37
(1,1741)	1:A:69:ILE:HD13	1:A:69:ILE:HG23	3	0.37
(1,1458)	1:A:51:VAL:H	1:A:51:VAL:HG21	4	0.37
(1,1458)	1:A:51:VAL:H	1:A:51:VAL:HG22	4	0.37
(1,1458)	1:A:51:VAL:H	1:A:51:VAL:HG23	4	0.37
(1,1458)	1:A:51:VAL:H	1:A:51:VAL:HG21	5	0.37
(1,1458)	1:A:51:VAL:H	1:A:51:VAL:HG22	5	0.37
(1,1458)	1:A:51:VAL:H	1:A:51:VAL:HG23	5	0.37
(1,1458)	1:A:51:VAL:H	1:A:51:VAL:HG21	10	0.37
(1,1458)	1:A:51:VAL:H	1:A:51:VAL:HG22	10	0.37
(1,1458)	1:A:51:VAL:H	1:A:51:VAL:HG23	10	0.37
(1,1263)	1:A:43:CYS:HB3	1:A:47:TYR:HD1	1	0.37
(1,1263)	1:A:43:CYS:HB3	1:A:47:TYR:HD2	1	0.37
(1,1263)	1:A:43:CYS:HB3	1:A:47:TYR:HD1	9	0.37
(1,1263)	1:A:43:CYS:HB3	1:A:47:TYR:HD2	9	0.37
(1,1036)	1:A:36:ILE:HA	1:A:36:ILE:HD11	3	0.37
(1,1036)	1:A:36:ILE:HA	1:A:36:ILE:HD12	3	0.37
(1,1036)	1:A:36:ILE:HA	1:A:36:ILE:HD13	3	0.37
(1,1036)	1:A:36:ILE:HA	1:A:36:ILE:HD11	8	0.37
(1,1036)	1:A:36:ILE:HA	1:A:36:ILE:HD12	8	0.37
(1,1036)	1:A:36:ILE:HA	1:A:36:ILE:HD13	8	0.37
(2,99)	1:A:20:LEU:HA	1:A:79:LYS:HE2	2	0.36
(2,99)	1:A:20:LEU:HA	1:A:79:LYS:HE3	2	0.36
(2,98)	1:A:20:LEU:HA	1:A:79:LYS:HE2	2	0.36
(2,98)	1:A:20:LEU:HA	1:A:79:LYS:HE3	2	0.36
(2,9)	1:A:1:SER:HB2	1:A:2:VAL:HA	2	0.36
(2,9)	1:A:1:SER:HB3	1:A:2:VAL:HA	2	0.36
(1,692)	1:A:22:ILE:H	1:A:51:VAL:HG11	6	0.36
(1,692)	1:A:22:ILE:H	1:A:51:VAL:HG12	6	0.36
(1,692)	1:A:22:ILE:H	1:A:51:VAL:HG13	6	0.36
(1,412)	1:A:15:ILE:HG21	1:A:15:ILE:HD11	2	0.36
(1,412)	1:A:15:ILE:HG21	1:A:15:ILE:HD12	2	0.36
(1,412)	1:A:15:ILE:HG21	1:A:15:ILE:HD13	2	0.36
(1,412)	1:A:15:ILE:HG22	1:A:15:ILE:HD11	2	0.36
(1,412)	1:A:15:ILE:HG22	1:A:15:ILE:HD12	2	0.36
(1,412)	1:A:15:ILE:HG22	1:A:15:ILE:HD13	2	0.36
(1,412)	1:A:15:ILE:HG23	1:A:15:ILE:HD11	2	0.36
(1,412)	1:A:15:ILE:HG23	1:A:15:ILE:HD12	2	0.36
(1,412)	1:A:15:ILE:HG23	1:A:15:ILE:HD13	2	0.36

Continued on next page...

Continued from previous page...

Key	Atom-1	Atom-2	Model ID	Violation (Å)
(1,366)	1:A:15:ILE:HD11	1:A:15:ILE:HG21	2	0.36
(1,366)	1:A:15:ILE:HD11	1:A:15:ILE:HG22	2	0.36
(1,366)	1:A:15:ILE:HD11	1:A:15:ILE:HG23	2	0.36
(1,366)	1:A:15:ILE:HD12	1:A:15:ILE:HG21	2	0.36
(1,366)	1:A:15:ILE:HD12	1:A:15:ILE:HG22	2	0.36
(1,366)	1:A:15:ILE:HD12	1:A:15:ILE:HG23	2	0.36
(1,366)	1:A:15:ILE:HD13	1:A:15:ILE:HG21	2	0.36
(1,366)	1:A:15:ILE:HD13	1:A:15:ILE:HG22	2	0.36
(1,366)	1:A:15:ILE:HD13	1:A:15:ILE:HG23	2	0.36
(1,30)	1:A:2:VAL:H	1:A:2:VAL:HG21	10	0.36
(1,30)	1:A:2:VAL:H	1:A:2:VAL:HG22	10	0.36
(1,30)	1:A:2:VAL:H	1:A:2:VAL:HG23	10	0.36
(1,1916)	1:A:76:LYS:HD2	1:A:86:THR:HG21	4	0.36
(1,1916)	1:A:76:LYS:HD2	1:A:86:THR:HG22	4	0.36
(1,1916)	1:A:76:LYS:HD2	1:A:86:THR:HG23	4	0.36
(1,1916)	1:A:76:LYS:HD3	1:A:86:THR:HG21	4	0.36
(1,1916)	1:A:76:LYS:HD3	1:A:86:THR:HG22	4	0.36
(1,1916)	1:A:76:LYS:HD3	1:A:86:THR:HG23	4	0.36
(1,1741)	1:A:69:ILE:HD11	1:A:69:ILE:HG21	7	0.36
(1,1741)	1:A:69:ILE:HD11	1:A:69:ILE:HG22	7	0.36
(1,1741)	1:A:69:ILE:HD11	1:A:69:ILE:HG23	7	0.36
(1,1741)	1:A:69:ILE:HD12	1:A:69:ILE:HG21	7	0.36
(1,1741)	1:A:69:ILE:HD12	1:A:69:ILE:HG22	7	0.36
(1,1741)	1:A:69:ILE:HD12	1:A:69:ILE:HG23	7	0.36
(1,1741)	1:A:69:ILE:HD13	1:A:69:ILE:HG21	7	0.36
(1,1741)	1:A:69:ILE:HD13	1:A:69:ILE:HG22	7	0.36
(1,1741)	1:A:69:ILE:HD13	1:A:69:ILE:HG23	7	0.36
(1,1036)	1:A:36:ILE:HA	1:A:36:ILE:HD11	2	0.36
(1,1036)	1:A:36:ILE:HA	1:A:36:ILE:HD12	2	0.36
(1,1036)	1:A:36:ILE:HA	1:A:36:ILE:HD13	2	0.36
(2,408)	1:A:29:TRP:HE1	1:A:29:TRP:H	1	0.35
(2,408)	1:A:29:TRP:HE1	1:A:29:TRP:H	9	0.35
(2,402)	1:A:27:ALA:HB1	1:A:30:CYS:H	10	0.35
(2,402)	1:A:27:ALA:HB2	1:A:30:CYS:H	10	0.35
(2,402)	1:A:27:ALA:HB3	1:A:30:CYS:H	10	0.35
(2,398)	1:A:26:PHE:HD1	1:A:27:ALA:HB1	1	0.35
(2,398)	1:A:26:PHE:HD1	1:A:27:ALA:HB2	1	0.35
(2,398)	1:A:26:PHE:HD1	1:A:27:ALA:HB3	1	0.35
(2,398)	1:A:26:PHE:HD2	1:A:27:ALA:HB1	1	0.35
(2,398)	1:A:26:PHE:HD2	1:A:27:ALA:HB2	1	0.35
(2,398)	1:A:26:PHE:HD2	1:A:27:ALA:HB3	1	0.35
(2,287)	1:A:57:VAL:HG21	1:A:61:SER:HA	4	0.35

Continued on next page...

Continued from previous page...

Key	Atom-1	Atom-2	Model ID	Violation (Å)
(2,287)	1:A:57:VAL:HG22	1:A:61:SER:HA	4	0.35
(2,287)	1:A:57:VAL:HG23	1:A:61:SER:HA	4	0.35
(2,287)	1:A:57:VAL:HG21	1:A:61:SER:HA	9	0.35
(2,287)	1:A:57:VAL:HG22	1:A:61:SER:HA	9	0.35
(2,287)	1:A:57:VAL:HG23	1:A:61:SER:HA	9	0.35
(2,287)	1:A:57:VAL:HG21	1:A:61:SER:HA	10	0.35
(2,287)	1:A:57:VAL:HG22	1:A:61:SER:HA	10	0.35
(2,287)	1:A:57:VAL:HG23	1:A:61:SER:HA	10	0.35
(2,229)	1:A:44:SER:HG	1:A:45:LYS:HD2	9	0.35
(2,229)	1:A:44:SER:HG	1:A:45:LYS:HD3	9	0.35
(2,215)	1:A:42:GLU:H	1:A:45:LYS:HE2	3	0.35
(2,215)	1:A:42:GLU:H	1:A:45:LYS:HE3	3	0.35
(2,209)	1:A:40:TYR:HD1	1:A:43:CYS:H	3	0.35
(2,209)	1:A:40:TYR:HD2	1:A:43:CYS:H	3	0.35
(2,134)	1:A:26:PHE:HA	1:A:75:PHE:HE1	1	0.35
(2,134)	1:A:26:PHE:HA	1:A:75:PHE:HE2	1	0.35
(1,749)	1:A:23:VAL:HG21	1:A:54:LYS:HA	7	0.35
(1,749)	1:A:23:VAL:HG22	1:A:54:LYS:HA	7	0.35
(1,749)	1:A:23:VAL:HG23	1:A:54:LYS:HA	7	0.35
(1,633)	1:A:22:ILE:HA	1:A:22:ILE:HD11	3	0.35
(1,633)	1:A:22:ILE:HA	1:A:22:ILE:HD12	3	0.35
(1,633)	1:A:22:ILE:HA	1:A:22:ILE:HD13	3	0.35
(1,382)	1:A:15:ILE:HD11	1:A:23:VAL:HG21	8	0.35
(1,382)	1:A:15:ILE:HD11	1:A:23:VAL:HG22	8	0.35
(1,382)	1:A:15:ILE:HD11	1:A:23:VAL:HG23	8	0.35
(1,382)	1:A:15:ILE:HD12	1:A:23:VAL:HG21	8	0.35
(1,382)	1:A:15:ILE:HD12	1:A:23:VAL:HG22	8	0.35
(1,382)	1:A:15:ILE:HD12	1:A:23:VAL:HG23	8	0.35
(1,382)	1:A:15:ILE:HD13	1:A:23:VAL:HG21	8	0.35
(1,382)	1:A:15:ILE:HD13	1:A:23:VAL:HG22	8	0.35
(1,382)	1:A:15:ILE:HD13	1:A:23:VAL:HG23	8	0.35
(1,380)	1:A:15:ILE:HD11	1:A:23:VAL:HB	2	0.35
(1,380)	1:A:15:ILE:HD12	1:A:23:VAL:HB	2	0.35
(1,380)	1:A:15:ILE:HD13	1:A:23:VAL:HB	2	0.35
(1,375)	1:A:15:ILE:HD11	1:A:21:VAL:HG11	7	0.35
(1,375)	1:A:15:ILE:HD11	1:A:21:VAL:HG12	7	0.35
(1,375)	1:A:15:ILE:HD11	1:A:21:VAL:HG13	7	0.35
(1,375)	1:A:15:ILE:HD12	1:A:21:VAL:HG11	7	0.35
(1,375)	1:A:15:ILE:HD12	1:A:21:VAL:HG12	7	0.35
(1,375)	1:A:15:ILE:HD12	1:A:21:VAL:HG13	7	0.35
(1,375)	1:A:15:ILE:HD13	1:A:21:VAL:HG11	7	0.35
(1,375)	1:A:15:ILE:HD13	1:A:21:VAL:HG12	7	0.35

Continued on next page...

Continued from previous page...

Key	Atom-1	Atom-2	Model ID	Violation (Å)
(1,375)	1:A:15:ILE:HD13	1:A:21:VAL:HG13	7	0.35
(1,30)	1:A:2:VAL:H	1:A:2:VAL:HG21	3	0.35
(1,30)	1:A:2:VAL:H	1:A:2:VAL:HG22	3	0.35
(1,30)	1:A:2:VAL:H	1:A:2:VAL:HG23	3	0.35
(1,30)	1:A:2:VAL:H	1:A:2:VAL:HG21	9	0.35
(1,30)	1:A:2:VAL:H	1:A:2:VAL:HG22	9	0.35
(1,30)	1:A:2:VAL:H	1:A:2:VAL:HG23	9	0.35
(1,2197)	1:A:88:LEU:HD11	1:A:89:GLY:H	1	0.35
(1,2197)	1:A:88:LEU:HD12	1:A:89:GLY:H	1	0.35
(1,2197)	1:A:88:LEU:HD13	1:A:89:GLY:H	1	0.35
(1,2197)	1:A:88:LEU:HD21	1:A:89:GLY:H	1	0.35
(1,2197)	1:A:88:LEU:HD22	1:A:89:GLY:H	1	0.35
(1,2197)	1:A:88:LEU:HD23	1:A:89:GLY:H	1	0.35
(1,1933)	1:A:77:VAL:HB	1:A:84:VAL:HG11	10	0.35
(1,1933)	1:A:77:VAL:HB	1:A:84:VAL:HG12	10	0.35
(1,1933)	1:A:77:VAL:HB	1:A:84:VAL:HG13	10	0.35
(1,1816)	1:A:74:THR:HG21	1:A:74:THR:HA	3	0.35
(1,1816)	1:A:74:THR:HG22	1:A:74:THR:HA	3	0.35
(1,1816)	1:A:74:THR:HG23	1:A:74:THR:HA	3	0.35
(1,1359)	1:A:47:TYR:HB3	1:A:103:ALA:HB1	5	0.35
(1,1359)	1:A:47:TYR:HB3	1:A:103:ALA:HB2	5	0.35
(1,1359)	1:A:47:TYR:HB3	1:A:103:ALA:HB3	5	0.35
(1,1334)	1:A:46:THR:HG21	1:A:47:TYR:HD1	3	0.35
(1,1334)	1:A:46:THR:HG21	1:A:47:TYR:HD2	3	0.35
(1,1334)	1:A:46:THR:HG22	1:A:47:TYR:HD1	3	0.35
(1,1334)	1:A:46:THR:HG22	1:A:47:TYR:HD2	3	0.35
(1,1334)	1:A:46:THR:HG23	1:A:47:TYR:HD1	3	0.35
(1,1334)	1:A:46:THR:HG23	1:A:47:TYR:HD2	3	0.35
(1,1263)	1:A:43:CYS:HB3	1:A:47:TYR:HD1	4	0.35
(1,1263)	1:A:43:CYS:HB3	1:A:47:TYR:HD2	4	0.35
(1,1263)	1:A:43:CYS:HB3	1:A:47:TYR:HD1	6	0.35
(1,1263)	1:A:43:CYS:HB3	1:A:47:TYR:HD2	6	0.35
(1,1036)	1:A:36:ILE:HA	1:A:36:ILE:HD11	6	0.35
(1,1036)	1:A:36:ILE:HA	1:A:36:ILE:HD12	6	0.35
(1,1036)	1:A:36:ILE:HA	1:A:36:ILE:HD13	6	0.35
(2,9)	1:A:1:SER:HB2	1:A:2:VAL:HA	5	0.34
(2,9)	1:A:1:SER:HB3	1:A:2:VAL:HA	5	0.34
(2,9)	1:A:1:SER:HB2	1:A:2:VAL:HA	7	0.34
(2,9)	1:A:1:SER:HB3	1:A:2:VAL:HA	7	0.34
(2,80)	1:A:12:ASP:HB3	1:A:66:LYS:HE2	5	0.34
(2,80)	1:A:12:ASP:HB3	1:A:66:LYS:HE3	5	0.34
(2,408)	1:A:29:TRP:HE1	1:A:29:TRP:H	3	0.34

Continued on next page...

Continued from previous page...

Key	Atom-1	Atom-2	Model ID	Violation (Å)
(2,408)	1:A:29:TRP:HE1	1:A:29:TRP:H	4	0.34
(2,408)	1:A:29:TRP:HE1	1:A:29:TRP:H	6	0.34
(2,408)	1:A:29:TRP:HE1	1:A:29:TRP:H	8	0.34
(2,408)	1:A:29:TRP:HE1	1:A:29:TRP:H	10	0.34
(2,372)	1:A:77:VAL:HA	1:A:78:TYR:HE1	4	0.34
(2,372)	1:A:77:VAL:HA	1:A:78:TYR:HE2	4	0.34
(2,359)	1:A:74:THR:H	1:A:75:PHE:HE1	8	0.34
(2,359)	1:A:74:THR:H	1:A:75:PHE:HE2	8	0.34
(2,287)	1:A:57:VAL:HG21	1:A:61:SER:HA	5	0.34
(2,287)	1:A:57:VAL:HG22	1:A:61:SER:HA	5	0.34
(2,287)	1:A:57:VAL:HG23	1:A:61:SER:HA	5	0.34
(2,134)	1:A:26:PHE:HA	1:A:75:PHE:HE1	7	0.34
(2,134)	1:A:26:PHE:HA	1:A:75:PHE:HE2	7	0.34
(1,990)	1:A:33:CYS:HA	1:A:90:ALA:HB1	5	0.34
(1,990)	1:A:33:CYS:HA	1:A:90:ALA:HB2	5	0.34
(1,990)	1:A:33:CYS:HA	1:A:90:ALA:HB3	5	0.34
(1,684)	1:A:22:ILE:H	1:A:22:ILE:HG21	8	0.34
(1,684)	1:A:22:ILE:H	1:A:22:ILE:HG22	8	0.34
(1,684)	1:A:22:ILE:H	1:A:22:ILE:HG23	8	0.34
(1,633)	1:A:22:ILE:HA	1:A:22:ILE:HD11	10	0.34
(1,633)	1:A:22:ILE:HA	1:A:22:ILE:HD12	10	0.34
(1,633)	1:A:22:ILE:HA	1:A:22:ILE:HD13	10	0.34
(1,576)	1:A:20:LEU:HD11	1:A:78:TYR:H	2	0.34
(1,576)	1:A:20:LEU:HD12	1:A:78:TYR:H	2	0.34
(1,576)	1:A:20:LEU:HD13	1:A:78:TYR:H	2	0.34
(1,555)	1:A:20:LEU:HA	1:A:20:LEU:HD11	3	0.34
(1,555)	1:A:20:LEU:HA	1:A:20:LEU:HD12	3	0.34
(1,555)	1:A:20:LEU:HA	1:A:20:LEU:HD13	3	0.34
(1,2172)	1:A:87:LEU:HD11	1:A:91:ASN:HB2	6	0.34
(1,2172)	1:A:87:LEU:HD12	1:A:91:ASN:HB2	6	0.34
(1,2172)	1:A:87:LEU:HD13	1:A:91:ASN:HB2	6	0.34
(1,1981)	1:A:77:VAL:HG21	1:A:102:TYR:HE1	4	0.34
(1,1981)	1:A:77:VAL:HG21	1:A:102:TYR:HE2	4	0.34
(1,1981)	1:A:77:VAL:HG22	1:A:102:TYR:HE1	4	0.34
(1,1981)	1:A:77:VAL:HG22	1:A:102:TYR:HE2	4	0.34
(1,1981)	1:A:77:VAL:HG23	1:A:102:TYR:HE1	4	0.34
(1,1981)	1:A:77:VAL:HG23	1:A:102:TYR:HE2	4	0.34
(1,1915)	1:A:76:LYS:HD2	1:A:86:THR:HA	5	0.34
(1,1915)	1:A:76:LYS:HD3	1:A:86:THR:HA	5	0.34
(1,1890)	1:A:75:PHE:HZ	1:A:90:ALA:HB1	1	0.34
(1,1890)	1:A:75:PHE:HZ	1:A:90:ALA:HB2	1	0.34
(1,1890)	1:A:75:PHE:HZ	1:A:90:ALA:HB3	1	0.34

Continued on next page...

Continued from previous page...

Key	Atom-1	Atom-2	Model ID	Violation (Å)
(1,1741)	1:A:69:ILE:HD11	1:A:69:ILE:HG21	6	0.34
(1,1741)	1:A:69:ILE:HD11	1:A:69:ILE:HG22	6	0.34
(1,1741)	1:A:69:ILE:HD11	1:A:69:ILE:HG23	6	0.34
(1,1741)	1:A:69:ILE:HD12	1:A:69:ILE:HG21	6	0.34
(1,1741)	1:A:69:ILE:HD12	1:A:69:ILE:HG22	6	0.34
(1,1741)	1:A:69:ILE:HD12	1:A:69:ILE:HG23	6	0.34
(1,1741)	1:A:69:ILE:HD13	1:A:69:ILE:HG21	6	0.34
(1,1741)	1:A:69:ILE:HD13	1:A:69:ILE:HG22	6	0.34
(1,1741)	1:A:69:ILE:HD13	1:A:69:ILE:HG23	6	0.34
(1,1421)	1:A:49:LYS:H	1:A:49:LYS:HD2	6	0.34
(1,1421)	1:A:49:LYS:H	1:A:49:LYS:HD3	6	0.34
(1,1359)	1:A:47:TYR:HB3	1:A:103:ALA:HB1	2	0.34
(1,1359)	1:A:47:TYR:HB3	1:A:103:ALA:HB2	2	0.34
(1,1359)	1:A:47:TYR:HB3	1:A:103:ALA:HB3	2	0.34
(1,1270)	1:A:43:CYS:HB2	1:A:47:TYR:HD1	5	0.34
(1,1270)	1:A:43:CYS:HB2	1:A:47:TYR:HD2	5	0.34
(1,1263)	1:A:43:CYS:HB3	1:A:47:TYR:HD1	2	0.34
(1,1263)	1:A:43:CYS:HB3	1:A:47:TYR:HD2	2	0.34
(1,1263)	1:A:43:CYS:HB3	1:A:47:TYR:HD1	3	0.34
(1,1263)	1:A:43:CYS:HB3	1:A:47:TYR:HD2	3	0.34
(1,1263)	1:A:43:CYS:HB3	1:A:47:TYR:HD1	7	0.34
(1,1263)	1:A:43:CYS:HB3	1:A:47:TYR:HD2	7	0.34
(1,1263)	1:A:43:CYS:HB3	1:A:47:TYR:HD1	10	0.34
(1,1263)	1:A:43:CYS:HB3	1:A:47:TYR:HD2	10	0.34
(1,112)	1:A:6:THR:H	1:A:6:THR:HG21	6	0.34
(1,112)	1:A:6:THR:H	1:A:6:THR:HG22	6	0.34
(1,112)	1:A:6:THR:H	1:A:6:THR:HG23	6	0.34
(1,1088)	1:A:36:ILE:HG21	1:A:75:PHE:HZ	1	0.34
(1,1088)	1:A:36:ILE:HG22	1:A:75:PHE:HZ	1	0.34
(1,1088)	1:A:36:ILE:HG23	1:A:75:PHE:HZ	1	0.34
(2,408)	1:A:29:TRP:HE1	1:A:29:TRP:H	2	0.33
(2,408)	1:A:29:TRP:HE1	1:A:29:TRP:H	5	0.33
(2,408)	1:A:29:TRP:HE1	1:A:29:TRP:H	7	0.33
(2,404)	1:A:28:GLU:HA	1:A:30:CYS:H	3	0.33
(2,134)	1:A:26:PHE:HA	1:A:75:PHE:HE1	8	0.33
(2,134)	1:A:26:PHE:HA	1:A:75:PHE:HE2	8	0.33
(2,118)	1:A:25:PHE:HD1	1:A:67:GLU:HG2	1	0.33
(2,118)	1:A:25:PHE:HD1	1:A:67:GLU:HG3	1	0.33
(2,118)	1:A:25:PHE:HD2	1:A:67:GLU:HG2	1	0.33
(2,118)	1:A:25:PHE:HD2	1:A:67:GLU:HG3	1	0.33
(1,731)	1:A:23:VAL:HG11	1:A:78:TYR:HE1	2	0.33
(1,731)	1:A:23:VAL:HG11	1:A:78:TYR:HE2	2	0.33

Continued on next page...

Continued from previous page...

Key	Atom-1	Atom-2	Model ID	Violation (Å)
(1,731)	1:A:23:VAL:HG12	1:A:78:TYR:HE1	2	0.33
(1,731)	1:A:23:VAL:HG12	1:A:78:TYR:HE2	2	0.33
(1,731)	1:A:23:VAL:HG13	1:A:78:TYR:HE1	2	0.33
(1,731)	1:A:23:VAL:HG13	1:A:78:TYR:HE2	2	0.33
(1,665)	1:A:22:ILE:HG21	1:A:50:MET:HB3	10	0.33
(1,665)	1:A:22:ILE:HG22	1:A:50:MET:HB3	10	0.33
(1,665)	1:A:22:ILE:HG23	1:A:50:MET:HB3	10	0.33
(1,576)	1:A:20:LEU:HD11	1:A:78:TYR:H	10	0.33
(1,576)	1:A:20:LEU:HD12	1:A:78:TYR:H	10	0.33
(1,576)	1:A:20:LEU:HD13	1:A:78:TYR:H	10	0.33
(1,412)	1:A:15:ILE:HG21	1:A:15:ILE:HD11	5	0.33
(1,412)	1:A:15:ILE:HG21	1:A:15:ILE:HD12	5	0.33
(1,412)	1:A:15:ILE:HG21	1:A:15:ILE:HD13	5	0.33
(1,412)	1:A:15:ILE:HG22	1:A:15:ILE:HD11	5	0.33
(1,412)	1:A:15:ILE:HG22	1:A:15:ILE:HD12	5	0.33
(1,412)	1:A:15:ILE:HG22	1:A:15:ILE:HD13	5	0.33
(1,412)	1:A:15:ILE:HG23	1:A:15:ILE:HD11	5	0.33
(1,412)	1:A:15:ILE:HG23	1:A:15:ILE:HD12	5	0.33
(1,412)	1:A:15:ILE:HG23	1:A:15:ILE:HD13	5	0.33
(1,366)	1:A:15:ILE:HD11	1:A:15:ILE:HG21	5	0.33
(1,366)	1:A:15:ILE:HD11	1:A:15:ILE:HG22	5	0.33
(1,366)	1:A:15:ILE:HD11	1:A:15:ILE:HG23	5	0.33
(1,366)	1:A:15:ILE:HD12	1:A:15:ILE:HG21	5	0.33
(1,366)	1:A:15:ILE:HD12	1:A:15:ILE:HG22	5	0.33
(1,366)	1:A:15:ILE:HD12	1:A:15:ILE:HG23	5	0.33
(1,366)	1:A:15:ILE:HD13	1:A:15:ILE:HG21	5	0.33
(1,366)	1:A:15:ILE:HD13	1:A:15:ILE:HG22	5	0.33
(1,366)	1:A:15:ILE:HD13	1:A:15:ILE:HG23	5	0.33
(1,2304)	1:A:95:LEU:HD11	1:A:95:LEU:HB2	4	0.33
(1,2304)	1:A:95:LEU:HD12	1:A:95:LEU:HB2	4	0.33
(1,2304)	1:A:95:LEU:HD13	1:A:95:LEU:HB2	4	0.33
(1,2303)	1:A:95:LEU:HD11	1:A:95:LEU:HB2	4	0.33
(1,2303)	1:A:95:LEU:HD12	1:A:95:LEU:HB2	4	0.33
(1,2303)	1:A:95:LEU:HD13	1:A:95:LEU:HB2	4	0.33
(1,2244)	1:A:91:ASN:HD21	1:A:94:ALA:HB1	9	0.33
(1,2244)	1:A:91:ASN:HD21	1:A:94:ALA:HB2	9	0.33
(1,2244)	1:A:91:ASN:HD21	1:A:94:ALA:HB3	9	0.33
(1,2199)	1:A:88:LEU:HD11	1:A:88:LEU:H	10	0.33
(1,2199)	1:A:88:LEU:HD12	1:A:88:LEU:H	10	0.33
(1,2199)	1:A:88:LEU:HD13	1:A:88:LEU:H	10	0.33
(1,1981)	1:A:77:VAL:HG21	1:A:102:TYR:HE1	8	0.33
(1,1981)	1:A:77:VAL:HG21	1:A:102:TYR:HE2	8	0.33

Continued on next page...

Continued from previous page...

Key	Atom-1	Atom-2	Model ID	Violation (Å)
(1,1981)	1:A:77:VAL:HG22	1:A:102:TYR:HE1	8	0.33
(1,1981)	1:A:77:VAL:HG22	1:A:102:TYR:HE2	8	0.33
(1,1981)	1:A:77:VAL:HG23	1:A:102:TYR:HE1	8	0.33
(1,1981)	1:A:77:VAL:HG23	1:A:102:TYR:HE2	8	0.33
(1,1812)	1:A:74:THR:HG1	1:A:74:THR:HG21	2	0.33
(1,1812)	1:A:74:THR:HG1	1:A:74:THR:HG22	2	0.33
(1,1812)	1:A:74:THR:HG1	1:A:74:THR:HG23	2	0.33
(1,1812)	1:A:74:THR:HG1	1:A:74:THR:HG21	6	0.33
(1,1812)	1:A:74:THR:HG1	1:A:74:THR:HG22	6	0.33
(1,1812)	1:A:74:THR:HG1	1:A:74:THR:HG23	6	0.33
(1,1741)	1:A:69:ILE:HD11	1:A:69:ILE:HG21	5	0.33
(1,1741)	1:A:69:ILE:HD11	1:A:69:ILE:HG22	5	0.33
(1,1741)	1:A:69:ILE:HD11	1:A:69:ILE:HG23	5	0.33
(1,1741)	1:A:69:ILE:HD12	1:A:69:ILE:HG21	5	0.33
(1,1741)	1:A:69:ILE:HD12	1:A:69:ILE:HG22	5	0.33
(1,1741)	1:A:69:ILE:HD12	1:A:69:ILE:HG23	5	0.33
(1,1741)	1:A:69:ILE:HD13	1:A:69:ILE:HG21	5	0.33
(1,1741)	1:A:69:ILE:HD13	1:A:69:ILE:HG22	5	0.33
(1,1741)	1:A:69:ILE:HD13	1:A:69:ILE:HG23	5	0.33
(1,12)	1:A:2:VAL:HG11	1:A:3:LYS:H	4	0.33
(1,12)	1:A:2:VAL:HG12	1:A:3:LYS:H	4	0.33
(1,12)	1:A:2:VAL:HG13	1:A:3:LYS:H	4	0.33
(1,1036)	1:A:36:ILE:HA	1:A:36:ILE:HD11	4	0.33
(1,1036)	1:A:36:ILE:HA	1:A:36:ILE:HD12	4	0.33
(1,1036)	1:A:36:ILE:HA	1:A:36:ILE:HD13	4	0.33
(2,404)	1:A:28:GLU:HA	1:A:30:CYS:H	8	0.32
(2,328)	1:A:69:ILE:HG21	1:A:72:MET:HB2	10	0.32
(2,328)	1:A:69:ILE:HG22	1:A:72:MET:HB2	10	0.32
(2,328)	1:A:69:ILE:HG23	1:A:72:MET:HB2	10	0.32
(2,151)	1:A:27:ALA:HB1	1:A:58:ASP:HB2	6	0.32
(2,151)	1:A:27:ALA:HB2	1:A:58:ASP:HB2	6	0.32
(2,151)	1:A:27:ALA:HB3	1:A:58:ASP:HB2	6	0.32
(1,684)	1:A:22:ILE:H	1:A:22:ILE:HG21	4	0.32
(1,684)	1:A:22:ILE:H	1:A:22:ILE:HG22	4	0.32
(1,684)	1:A:22:ILE:H	1:A:22:ILE:HG23	4	0.32
(1,576)	1:A:20:LEU:HD11	1:A:78:TYR:H	4	0.32
(1,576)	1:A:20:LEU:HD12	1:A:78:TYR:H	4	0.32
(1,576)	1:A:20:LEU:HD13	1:A:78:TYR:H	4	0.32
(1,503)	1:A:18:ASN:HD21	1:A:21:VAL:HG21	6	0.32
(1,503)	1:A:18:ASN:HD21	1:A:21:VAL:HG22	6	0.32
(1,503)	1:A:18:ASN:HD21	1:A:21:VAL:HG23	6	0.32
(1,503)	1:A:18:ASN:HD21	1:A:21:VAL:HG21	7	0.32

Continued on next page...

Continued from previous page...

Key	Atom-1	Atom-2	Model ID	Violation (Å)
(1,503)	1:A:18:ASN:HD21	1:A:21:VAL:HG22	7	0.32
(1,503)	1:A:18:ASN:HD21	1:A:21:VAL:HG23	7	0.32
(1,227)	1:A:11:PHE:HE1	1:A:23:VAL:HG11	9	0.32
(1,227)	1:A:11:PHE:HE1	1:A:23:VAL:HG12	9	0.32
(1,227)	1:A:11:PHE:HE1	1:A:23:VAL:HG13	9	0.32
(1,227)	1:A:11:PHE:HE2	1:A:23:VAL:HG11	9	0.32
(1,227)	1:A:11:PHE:HE2	1:A:23:VAL:HG12	9	0.32
(1,227)	1:A:11:PHE:HE2	1:A:23:VAL:HG13	9	0.32
(1,1976)	1:A:77:VAL:HG21	1:A:98:LEU:HD21	5	0.32
(1,1976)	1:A:77:VAL:HG21	1:A:98:LEU:HD22	5	0.32
(1,1976)	1:A:77:VAL:HG21	1:A:98:LEU:HD23	5	0.32
(1,1976)	1:A:77:VAL:HG22	1:A:98:LEU:HD21	5	0.32
(1,1976)	1:A:77:VAL:HG22	1:A:98:LEU:HD22	5	0.32
(1,1976)	1:A:77:VAL:HG22	1:A:98:LEU:HD23	5	0.32
(1,1976)	1:A:77:VAL:HG23	1:A:98:LEU:HD21	5	0.32
(1,1976)	1:A:77:VAL:HG23	1:A:98:LEU:HD22	5	0.32
(1,1976)	1:A:77:VAL:HG23	1:A:98:LEU:HD23	5	0.32
(1,1896)	1:A:76:LYS:HA	1:A:76:LYS:HG2	1	0.32
(1,1896)	1:A:76:LYS:HA	1:A:76:LYS:HG3	1	0.32
(1,1896)	1:A:76:LYS:HA	1:A:76:LYS:HG2	4	0.32
(1,1896)	1:A:76:LYS:HA	1:A:76:LYS:HG3	4	0.32
(1,1890)	1:A:75:PHE:HZ	1:A:90:ALA:HB1	2	0.32
(1,1890)	1:A:75:PHE:HZ	1:A:90:ALA:HB2	2	0.32
(1,1890)	1:A:75:PHE:HZ	1:A:90:ALA:HB3	2	0.32
(1,1823)	1:A:74:THR:HG21	1:A:89:GLY:H	7	0.32
(1,1823)	1:A:74:THR:HG22	1:A:89:GLY:H	7	0.32
(1,1823)	1:A:74:THR:HG23	1:A:89:GLY:H	7	0.32
(1,1823)	1:A:74:THR:HG21	1:A:89:GLY:H	10	0.32
(1,1823)	1:A:74:THR:HG22	1:A:89:GLY:H	10	0.32
(1,1823)	1:A:74:THR:HG23	1:A:89:GLY:H	10	0.32
(1,1812)	1:A:74:THR:HG1	1:A:74:THR:HG21	9	0.32
(1,1812)	1:A:74:THR:HG1	1:A:74:THR:HG22	9	0.32
(1,1812)	1:A:74:THR:HG1	1:A:74:THR:HG23	9	0.32
(1,1745)	1:A:69:ILE:HD11	1:A:74:THR:HG1	7	0.32
(1,1745)	1:A:69:ILE:HD12	1:A:74:THR:HG1	7	0.32
(1,1745)	1:A:69:ILE:HD13	1:A:74:THR:HG1	7	0.32
(1,1741)	1:A:69:ILE:HD11	1:A:69:ILE:HG21	2	0.32
(1,1741)	1:A:69:ILE:HD11	1:A:69:ILE:HG22	2	0.32
(1,1741)	1:A:69:ILE:HD11	1:A:69:ILE:HG23	2	0.32
(1,1741)	1:A:69:ILE:HD12	1:A:69:ILE:HG21	2	0.32
(1,1741)	1:A:69:ILE:HD12	1:A:69:ILE:HG22	2	0.32
(1,1741)	1:A:69:ILE:HD12	1:A:69:ILE:HG23	2	0.32

Continued on next page...

Continued from previous page...

Key	Atom-1	Atom-2	Model ID	Violation (Å)
(1,1741)	1:A:69:ILE:HD13	1:A:69:ILE:HG21	2	0.32
(1,1741)	1:A:69:ILE:HD13	1:A:69:ILE:HG22	2	0.32
(1,1741)	1:A:69:ILE:HD13	1:A:69:ILE:HG23	2	0.32
(1,154)	1:A:8:GLN:HE22	1:A:11:PHE:HD1	5	0.32
(1,154)	1:A:8:GLN:HE22	1:A:11:PHE:HD2	5	0.32
(1,144)	1:A:8:GLN:HE21	1:A:11:PHE:HD1	8	0.32
(1,144)	1:A:8:GLN:HE21	1:A:11:PHE:HD2	8	0.32
(1,1389)	1:A:47:TYR:HE1	1:A:96:LYS:HA	8	0.32
(1,1389)	1:A:47:TYR:HE2	1:A:96:LYS:HA	8	0.32
(2,404)	1:A:28:GLU:HA	1:A:30:CYS:H	2	0.31
(2,404)	1:A:28:GLU:HA	1:A:30:CYS:H	6	0.31
(2,402)	1:A:27:ALA:HB1	1:A:30:CYS:H	8	0.31
(2,402)	1:A:27:ALA:HB2	1:A:30:CYS:H	8	0.31
(2,402)	1:A:27:ALA:HB3	1:A:30:CYS:H	8	0.31
(2,34)	1:A:3:LYS:HE2	1:A:5:VAL:HB	1	0.31
(2,34)	1:A:3:LYS:HE3	1:A:5:VAL:HB	1	0.31
(2,287)	1:A:57:VAL:HG21	1:A:61:SER:HA	8	0.31
(2,287)	1:A:57:VAL:HG22	1:A:61:SER:HA	8	0.31
(2,287)	1:A:57:VAL:HG23	1:A:61:SER:HA	8	0.31
(1,752)	1:A:23:VAL:HG21	1:A:55:VAL:HG21	9	0.31
(1,752)	1:A:23:VAL:HG21	1:A:55:VAL:HG22	9	0.31
(1,752)	1:A:23:VAL:HG21	1:A:55:VAL:HG23	9	0.31
(1,752)	1:A:23:VAL:HG22	1:A:55:VAL:HG21	9	0.31
(1,752)	1:A:23:VAL:HG22	1:A:55:VAL:HG22	9	0.31
(1,752)	1:A:23:VAL:HG22	1:A:55:VAL:HG23	9	0.31
(1,752)	1:A:23:VAL:HG23	1:A:55:VAL:HG21	9	0.31
(1,752)	1:A:23:VAL:HG23	1:A:55:VAL:HG22	9	0.31
(1,752)	1:A:23:VAL:HG23	1:A:55:VAL:HG23	9	0.31
(1,736)	1:A:23:VAL:HG21	1:A:24:ASP:H	3	0.31
(1,736)	1:A:23:VAL:HG22	1:A:24:ASP:H	3	0.31
(1,736)	1:A:23:VAL:HG23	1:A:24:ASP:H	3	0.31
(1,735)	1:A:23:VAL:HG21	1:A:23:VAL:HG11	8	0.31
(1,735)	1:A:23:VAL:HG21	1:A:23:VAL:HG12	8	0.31
(1,735)	1:A:23:VAL:HG21	1:A:23:VAL:HG13	8	0.31
(1,735)	1:A:23:VAL:HG22	1:A:23:VAL:HG11	8	0.31
(1,735)	1:A:23:VAL:HG22	1:A:23:VAL:HG12	8	0.31
(1,735)	1:A:23:VAL:HG22	1:A:23:VAL:HG13	8	0.31
(1,735)	1:A:23:VAL:HG23	1:A:23:VAL:HG11	8	0.31
(1,735)	1:A:23:VAL:HG23	1:A:23:VAL:HG12	8	0.31
(1,735)	1:A:23:VAL:HG23	1:A:23:VAL:HG13	8	0.31
(1,731)	1:A:23:VAL:HG11	1:A:78:TYR:HE1	5	0.31
(1,731)	1:A:23:VAL:HG11	1:A:78:TYR:HE2	5	0.31

Continued on next page...

Continued from previous page...

Key	Atom-1	Atom-2	Model ID	Violation (Å)
(1,731)	1:A:23:VAL:HG12	1:A:78:TYR:HE1	5	0.31
(1,731)	1:A:23:VAL:HG12	1:A:78:TYR:HE2	5	0.31
(1,731)	1:A:23:VAL:HG13	1:A:78:TYR:HE1	5	0.31
(1,731)	1:A:23:VAL:HG13	1:A:78:TYR:HE2	5	0.31
(1,555)	1:A:20:LEU:HA	1:A:20:LEU:HD11	10	0.31
(1,555)	1:A:20:LEU:HA	1:A:20:LEU:HD12	10	0.31
(1,555)	1:A:20:LEU:HA	1:A:20:LEU:HD13	10	0.31
(1,380)	1:A:15:ILE:HD11	1:A:23:VAL:HB	4	0.31
(1,380)	1:A:15:ILE:HD12	1:A:23:VAL:HB	4	0.31
(1,380)	1:A:15:ILE:HD13	1:A:23:VAL:HB	4	0.31
(1,2388)	1:A:98:LEU:HD21	1:A:99:ILE:HA	9	0.31
(1,2388)	1:A:98:LEU:HD22	1:A:99:ILE:HA	9	0.31
(1,2388)	1:A:98:LEU:HD23	1:A:99:ILE:HA	9	0.31
(1,227)	1:A:11:PHE:HE1	1:A:23:VAL:HG11	5	0.31
(1,227)	1:A:11:PHE:HE1	1:A:23:VAL:HG12	5	0.31
(1,227)	1:A:11:PHE:HE1	1:A:23:VAL:HG13	5	0.31
(1,227)	1:A:11:PHE:HE2	1:A:23:VAL:HG11	5	0.31
(1,227)	1:A:11:PHE:HE2	1:A:23:VAL:HG12	5	0.31
(1,227)	1:A:11:PHE:HE2	1:A:23:VAL:HG13	5	0.31
(1,2200)	1:A:88:LEU:HD21	1:A:88:LEU:HA	6	0.31
(1,2200)	1:A:88:LEU:HD22	1:A:88:LEU:HA	6	0.31
(1,2200)	1:A:88:LEU:HD23	1:A:88:LEU:HA	6	0.31
(1,2052)	1:A:79:LYS:HD2	1:A:80:ASN:H	6	0.31
(1,2052)	1:A:79:LYS:HD3	1:A:80:ASN:H	6	0.31
(1,1896)	1:A:76:LYS:HA	1:A:76:LYS:HG2	2	0.31
(1,1896)	1:A:76:LYS:HA	1:A:76:LYS:HG3	2	0.31
(1,1896)	1:A:76:LYS:HA	1:A:76:LYS:HG2	6	0.31
(1,1896)	1:A:76:LYS:HA	1:A:76:LYS:HG3	6	0.31
(1,1812)	1:A:74:THR:HG1	1:A:74:THR:HG21	1	0.31
(1,1812)	1:A:74:THR:HG1	1:A:74:THR:HG22	1	0.31
(1,1812)	1:A:74:THR:HG1	1:A:74:THR:HG23	1	0.31
(1,1812)	1:A:74:THR:HG1	1:A:74:THR:HG21	10	0.31
(1,1812)	1:A:74:THR:HG1	1:A:74:THR:HG22	10	0.31
(1,1812)	1:A:74:THR:HG1	1:A:74:THR:HG23	10	0.31
(1,1778)	1:A:70:THR:H	1:A:70:THR:HG21	2	0.31
(1,1778)	1:A:70:THR:H	1:A:70:THR:HG22	2	0.31
(1,1778)	1:A:70:THR:H	1:A:70:THR:HG23	2	0.31
(1,1778)	1:A:70:THR:H	1:A:70:THR:HG21	10	0.31
(1,1778)	1:A:70:THR:H	1:A:70:THR:HG22	10	0.31
(1,1778)	1:A:70:THR:H	1:A:70:THR:HG23	10	0.31
(1,1741)	1:A:69:ILE:HD11	1:A:69:ILE:HG21	4	0.31
(1,1741)	1:A:69:ILE:HD11	1:A:69:ILE:HG22	4	0.31

Continued on next page...

Continued from previous page...

Key	Atom-1	Atom-2	Model ID	Violation (Å)
(1,1741)	1:A:69:ILE:HD11	1:A:69:ILE:HG23	4	0.31
(1,1741)	1:A:69:ILE:HD12	1:A:69:ILE:HG21	4	0.31
(1,1741)	1:A:69:ILE:HD12	1:A:69:ILE:HG22	4	0.31
(1,1741)	1:A:69:ILE:HD12	1:A:69:ILE:HG23	4	0.31
(1,1741)	1:A:69:ILE:HD13	1:A:69:ILE:HG21	4	0.31
(1,1741)	1:A:69:ILE:HD13	1:A:69:ILE:HG22	4	0.31
(1,1741)	1:A:69:ILE:HD13	1:A:69:ILE:HG23	4	0.31
(1,1666)	1:A:64:THR:HG21	1:A:69:ILE:H	1	0.31
(1,1666)	1:A:64:THR:HG22	1:A:69:ILE:H	1	0.31
(1,1666)	1:A:64:THR:HG23	1:A:69:ILE:H	1	0.31
(1,144)	1:A:8:GLN:HE21	1:A:11:PHE:HD1	7	0.31
(1,144)	1:A:8:GLN:HE21	1:A:11:PHE:HD2	7	0.31
(1,1421)	1:A:49:LYS:H	1:A:49:LYS:HD2	1	0.31
(1,1421)	1:A:49:LYS:H	1:A:49:LYS:HD3	1	0.31
(1,1358)	1:A:47:TYR:HB3	1:A:99:ILE:HD11	3	0.31
(1,1358)	1:A:47:TYR:HB3	1:A:99:ILE:HD12	3	0.31
(1,1358)	1:A:47:TYR:HB3	1:A:99:ILE:HD13	3	0.31
(1,1267)	1:A:43:CYS:HB3	1:A:99:ILE:HG21	8	0.31
(1,1267)	1:A:43:CYS:HB3	1:A:99:ILE:HG22	8	0.31
(1,1267)	1:A:43:CYS:HB3	1:A:99:ILE:HG23	8	0.31
(1,112)	1:A:6:THR:H	1:A:6:THR:HG21	3	0.31
(1,112)	1:A:6:THR:H	1:A:6:THR:HG22	3	0.31
(1,112)	1:A:6:THR:H	1:A:6:THR:HG23	3	0.31
(1,1088)	1:A:36:ILE:HG21	1:A:75:PHE:HZ	8	0.31
(1,1088)	1:A:36:ILE:HG22	1:A:75:PHE:HZ	8	0.31
(1,1088)	1:A:36:ILE:HG23	1:A:75:PHE:HZ	8	0.31
(2,83)	1:A:12:ASP:HB2	1:A:66:LYS:HE2	8	0.3
(2,83)	1:A:12:ASP:HB2	1:A:66:LYS:HE3	8	0.3
(2,404)	1:A:28:GLU:HA	1:A:30:CYS:H	10	0.3
(2,402)	1:A:27:ALA:HB1	1:A:30:CYS:H	2	0.3
(2,402)	1:A:27:ALA:HB2	1:A:30:CYS:H	2	0.3
(2,402)	1:A:27:ALA:HB3	1:A:30:CYS:H	2	0.3
(2,398)	1:A:26:PHE:HD1	1:A:27:ALA:HB1	9	0.3
(2,398)	1:A:26:PHE:HD1	1:A:27:ALA:HB2	9	0.3
(2,398)	1:A:26:PHE:HD1	1:A:27:ALA:HB3	9	0.3
(2,398)	1:A:26:PHE:HD2	1:A:27:ALA:HB1	9	0.3
(2,398)	1:A:26:PHE:HD2	1:A:27:ALA:HB2	9	0.3
(2,398)	1:A:26:PHE:HD2	1:A:27:ALA:HB3	9	0.3
(2,359)	1:A:74:THR:H	1:A:75:PHE:HE1	5	0.3
(2,359)	1:A:74:THR:H	1:A:75:PHE:HE2	5	0.3
(2,34)	1:A:3:LYS:HE2	1:A:5:VAL:HB	6	0.3
(2,34)	1:A:3:LYS:HE3	1:A:5:VAL:HB	6	0.3

Continued on next page...

Continued from previous page...

Key	Atom-1	Atom-2	Model ID	Violation (Å)
(2,325)	1:A:69:ILE:HG21	1:A:71:SER:HA	1	0.3
(2,325)	1:A:69:ILE:HG22	1:A:71:SER:HA	1	0.3
(2,325)	1:A:69:ILE:HG23	1:A:71:SER:HA	1	0.3
(1,97)	1:A:5:VAL:H	1:A:5:VAL:HG11	10	0.3
(1,97)	1:A:5:VAL:H	1:A:5:VAL:HG12	10	0.3
(1,97)	1:A:5:VAL:H	1:A:5:VAL:HG13	10	0.3
(1,764)	1:A:23:VAL:H	1:A:23:VAL:HG11	9	0.3
(1,764)	1:A:23:VAL:H	1:A:23:VAL:HG12	9	0.3
(1,764)	1:A:23:VAL:H	1:A:23:VAL:HG13	9	0.3
(1,731)	1:A:23:VAL:HG11	1:A:78:TYR:HE1	9	0.3
(1,731)	1:A:23:VAL:HG11	1:A:78:TYR:HE2	9	0.3
(1,731)	1:A:23:VAL:HG12	1:A:78:TYR:HE1	9	0.3
(1,731)	1:A:23:VAL:HG12	1:A:78:TYR:HE2	9	0.3
(1,731)	1:A:23:VAL:HG13	1:A:78:TYR:HE1	9	0.3
(1,731)	1:A:23:VAL:HG13	1:A:78:TYR:HE2	9	0.3
(1,723)	1:A:23:VAL:HG11	1:A:55:VAL:HG21	8	0.3
(1,723)	1:A:23:VAL:HG11	1:A:55:VAL:HG22	8	0.3
(1,723)	1:A:23:VAL:HG11	1:A:55:VAL:HG23	8	0.3
(1,723)	1:A:23:VAL:HG12	1:A:55:VAL:HG21	8	0.3
(1,723)	1:A:23:VAL:HG12	1:A:55:VAL:HG22	8	0.3
(1,723)	1:A:23:VAL:HG12	1:A:55:VAL:HG23	8	0.3
(1,723)	1:A:23:VAL:HG13	1:A:55:VAL:HG21	8	0.3
(1,723)	1:A:23:VAL:HG13	1:A:55:VAL:HG22	8	0.3
(1,723)	1:A:23:VAL:HG13	1:A:55:VAL:HG23	8	0.3
(1,712)	1:A:23:VAL:HG11	1:A:25:PHE:HZ	4	0.3
(1,712)	1:A:23:VAL:HG12	1:A:25:PHE:HZ	4	0.3
(1,712)	1:A:23:VAL:HG13	1:A:25:PHE:HZ	4	0.3
(1,577)	1:A:20:LEU:HD11	1:A:79:LYS:HA	2	0.3
(1,577)	1:A:20:LEU:HD12	1:A:79:LYS:HA	2	0.3
(1,577)	1:A:20:LEU:HD13	1:A:79:LYS:HA	2	0.3
(1,503)	1:A:18:ASN:HD21	1:A:21:VAL:HG21	3	0.3
(1,503)	1:A:18:ASN:HD21	1:A:21:VAL:HG22	3	0.3
(1,503)	1:A:18:ASN:HD21	1:A:21:VAL:HG23	3	0.3
(1,425)	1:A:15:ILE:HG21	1:A:78:TYR:HB2	7	0.3
(1,425)	1:A:15:ILE:HG22	1:A:78:TYR:HB2	7	0.3
(1,425)	1:A:15:ILE:HG23	1:A:78:TYR:HB2	7	0.3
(1,2389)	1:A:98:LEU:HD21	1:A:99:ILE:H	9	0.3
(1,2389)	1:A:98:LEU:HD22	1:A:99:ILE:H	9	0.3
(1,2389)	1:A:98:LEU:HD23	1:A:99:ILE:H	9	0.3
(1,2304)	1:A:95:LEU:HD11	1:A:95:LEU:HB2	3	0.3
(1,2304)	1:A:95:LEU:HD12	1:A:95:LEU:HB2	3	0.3
(1,2304)	1:A:95:LEU:HD13	1:A:95:LEU:HB2	3	0.3

Continued on next page...

Continued from previous page...

Key	Atom-1	Atom-2	Model ID	Violation (Å)
(1,2303)	1:A:95:LEU:HD11	1:A:95:LEU:HB2	3	0.3
(1,2303)	1:A:95:LEU:HD12	1:A:95:LEU:HB2	3	0.3
(1,2303)	1:A:95:LEU:HD13	1:A:95:LEU:HB2	3	0.3
(1,2197)	1:A:88:LEU:HD11	1:A:89:GLY:H	8	0.3
(1,2197)	1:A:88:LEU:HD12	1:A:89:GLY:H	8	0.3
(1,2197)	1:A:88:LEU:HD13	1:A:89:GLY:H	8	0.3
(1,2197)	1:A:88:LEU:HD21	1:A:89:GLY:H	8	0.3
(1,2197)	1:A:88:LEU:HD22	1:A:89:GLY:H	8	0.3
(1,2197)	1:A:88:LEU:HD23	1:A:89:GLY:H	8	0.3
(1,1915)	1:A:76:LYS:HD2	1:A:86:THR:HA	9	0.3
(1,1915)	1:A:76:LYS:HD3	1:A:86:THR:HA	9	0.3
(1,1896)	1:A:76:LYS:HA	1:A:76:LYS:HG2	8	0.3
(1,1896)	1:A:76:LYS:HA	1:A:76:LYS:HG3	8	0.3
(1,1851)	1:A:75:PHE:HB2	1:A:87:LEU:HD21	7	0.3
(1,1851)	1:A:75:PHE:HB2	1:A:87:LEU:HD22	7	0.3
(1,1851)	1:A:75:PHE:HB2	1:A:87:LEU:HD23	7	0.3
(1,1778)	1:A:70:THR:H	1:A:70:THR:HG21	7	0.3
(1,1778)	1:A:70:THR:H	1:A:70:THR:HG22	7	0.3
(1,1778)	1:A:70:THR:H	1:A:70:THR:HG23	7	0.3
(1,168)	1:A:8:GLN:H	1:A:60:VAL:HG11	6	0.3
(1,168)	1:A:8:GLN:H	1:A:60:VAL:HG12	6	0.3
(1,168)	1:A:8:GLN:H	1:A:60:VAL:HG13	6	0.3
(1,154)	1:A:8:GLN:HE22	1:A:11:PHE:HD1	2	0.3
(1,154)	1:A:8:GLN:HE22	1:A:11:PHE:HD2	2	0.3
(1,154)	1:A:8:GLN:HE22	1:A:11:PHE:HD1	9	0.3
(1,154)	1:A:8:GLN:HE22	1:A:11:PHE:HD2	9	0.3
(1,144)	1:A:8:GLN:HE21	1:A:11:PHE:HD1	6	0.3
(1,144)	1:A:8:GLN:HE21	1:A:11:PHE:HD2	6	0.3
(1,1421)	1:A:49:LYS:H	1:A:49:LYS:HD2	2	0.3
(1,1421)	1:A:49:LYS:H	1:A:49:LYS:HD3	2	0.3
(1,1421)	1:A:49:LYS:H	1:A:49:LYS:HD2	4	0.3
(1,1421)	1:A:49:LYS:H	1:A:49:LYS:HD3	4	0.3
(1,1376)	1:A:47:TYR:HD1	1:A:99:ILE:HG12	5	0.3
(1,1376)	1:A:47:TYR:HD2	1:A:99:ILE:HG12	5	0.3
(1,1334)	1:A:46:THR:HG21	1:A:47:TYR:HD1	7	0.3
(1,1334)	1:A:46:THR:HG21	1:A:47:TYR:HD2	7	0.3
(1,1334)	1:A:46:THR:HG22	1:A:47:TYR:HD1	7	0.3
(1,1334)	1:A:46:THR:HG22	1:A:47:TYR:HD2	7	0.3
(1,1334)	1:A:46:THR:HG23	1:A:47:TYR:HD1	7	0.3
(1,1334)	1:A:46:THR:HG23	1:A:47:TYR:HD2	7	0.3
(1,12)	1:A:2:VAL:HG11	1:A:3:LYS:H	6	0.3
(1,12)	1:A:2:VAL:HG12	1:A:3:LYS:H	6	0.3

Continued on next page...

Continued from previous page...

Key	Atom-1	Atom-2	Model ID	Violation (Å)
(1,12)	1:A:2:VAL:HG13	1:A:3:LYS:H	6	0.3
(1,112)	1:A:6:THR:H	1:A:6:THR:HG21	10	0.3
(1,112)	1:A:6:THR:H	1:A:6:THR:HG22	10	0.3
(1,112)	1:A:6:THR:H	1:A:6:THR:HG23	10	0.3
(2,82)	1:A:12:ASP:HB2	1:A:66:LYS:HD2	4	0.29
(2,82)	1:A:12:ASP:HB2	1:A:66:LYS:HD3	4	0.29
(2,404)	1:A:28:GLU:HA	1:A:30:CYS:H	1	0.29
(2,404)	1:A:28:GLU:HA	1:A:30:CYS:H	9	0.29
(2,329)	1:A:69:ILE:HG21	1:A:72:MET:HE1	9	0.29
(2,329)	1:A:69:ILE:HG21	1:A:72:MET:HE2	9	0.29
(2,329)	1:A:69:ILE:HG21	1:A:72:MET:HE3	9	0.29
(2,329)	1:A:69:ILE:HG22	1:A:72:MET:HE1	9	0.29
(2,329)	1:A:69:ILE:HG22	1:A:72:MET:HE2	9	0.29
(2,329)	1:A:69:ILE:HG22	1:A:72:MET:HE3	9	0.29
(2,329)	1:A:69:ILE:HG23	1:A:72:MET:HE1	9	0.29
(2,329)	1:A:69:ILE:HG23	1:A:72:MET:HE2	9	0.29
(2,329)	1:A:69:ILE:HG23	1:A:72:MET:HE3	9	0.29
(2,287)	1:A:57:VAL:HG21	1:A:61:SER:HA	2	0.29
(2,287)	1:A:57:VAL:HG22	1:A:61:SER:HA	2	0.29
(2,287)	1:A:57:VAL:HG23	1:A:61:SER:HA	2	0.29
(2,284)	1:A:57:VAL:HG11	1:A:61:SER:HA	5	0.29
(2,284)	1:A:57:VAL:HG12	1:A:61:SER:HA	5	0.29
(2,284)	1:A:57:VAL:HG13	1:A:61:SER:HA	5	0.29
(2,266)	1:A:50:MET:H	1:A:52:PHE:HD1	7	0.29
(2,266)	1:A:50:MET:H	1:A:52:PHE:HD2	7	0.29
(2,183)	1:A:29:TRP:HE1	1:A:72:MET:HE1	1	0.29
(2,183)	1:A:29:TRP:HE1	1:A:72:MET:HE2	1	0.29
(2,183)	1:A:29:TRP:HE1	1:A:72:MET:HE3	1	0.29
(1,927)	1:A:27:ALA:HA	1:A:29:TRP:HE1	6	0.29
(1,927)	1:A:27:ALA:HA	1:A:29:TRP:HE1	7	0.29
(1,927)	1:A:27:ALA:HA	1:A:29:TRP:HE1	8	0.29
(1,764)	1:A:23:VAL:H	1:A:23:VAL:HG11	1	0.29
(1,764)	1:A:23:VAL:H	1:A:23:VAL:HG12	1	0.29
(1,764)	1:A:23:VAL:H	1:A:23:VAL:HG13	1	0.29
(1,688)	1:A:22:ILE:H	1:A:50:MET:HA	10	0.29
(1,412)	1:A:15:ILE:HG21	1:A:15:ILE:HD11	4	0.29
(1,412)	1:A:15:ILE:HG21	1:A:15:ILE:HD12	4	0.29
(1,412)	1:A:15:ILE:HG21	1:A:15:ILE:HD13	4	0.29
(1,412)	1:A:15:ILE:HG22	1:A:15:ILE:HD11	4	0.29
(1,412)	1:A:15:ILE:HG22	1:A:15:ILE:HD12	4	0.29
(1,412)	1:A:15:ILE:HG22	1:A:15:ILE:HD13	4	0.29
(1,412)	1:A:15:ILE:HG23	1:A:15:ILE:HD11	4	0.29

Continued on next page...

Continued from previous page...

Key	Atom-1	Atom-2	Model ID	Violation (Å)
(1,412)	1:A:15:ILE:HG23	1:A:15:ILE:HD12	4	0.29
(1,412)	1:A:15:ILE:HG23	1:A:15:ILE:HD13	4	0.29
(1,380)	1:A:15:ILE:HD11	1:A:23:VAL:HB	5	0.29
(1,380)	1:A:15:ILE:HD12	1:A:23:VAL:HB	5	0.29
(1,380)	1:A:15:ILE:HD13	1:A:23:VAL:HB	5	0.29
(1,366)	1:A:15:ILE:HD11	1:A:15:ILE:HG21	4	0.29
(1,366)	1:A:15:ILE:HD11	1:A:15:ILE:HG22	4	0.29
(1,366)	1:A:15:ILE:HD11	1:A:15:ILE:HG23	4	0.29
(1,366)	1:A:15:ILE:HD12	1:A:15:ILE:HG21	4	0.29
(1,366)	1:A:15:ILE:HD12	1:A:15:ILE:HG22	4	0.29
(1,366)	1:A:15:ILE:HD12	1:A:15:ILE:HG23	4	0.29
(1,366)	1:A:15:ILE:HD13	1:A:15:ILE:HG21	4	0.29
(1,366)	1:A:15:ILE:HD13	1:A:15:ILE:HG22	4	0.29
(1,366)	1:A:15:ILE:HD13	1:A:15:ILE:HG23	4	0.29
(1,30)	1:A:2:VAL:H	1:A:2:VAL:HG21	5	0.29
(1,30)	1:A:2:VAL:H	1:A:2:VAL:HG22	5	0.29
(1,30)	1:A:2:VAL:H	1:A:2:VAL:HG23	5	0.29
(1,227)	1:A:11:PHE:HE1	1:A:23:VAL:HG11	6	0.29
(1,227)	1:A:11:PHE:HE1	1:A:23:VAL:HG12	6	0.29
(1,227)	1:A:11:PHE:HE1	1:A:23:VAL:HG13	6	0.29
(1,227)	1:A:11:PHE:HE2	1:A:23:VAL:HG11	6	0.29
(1,227)	1:A:11:PHE:HE2	1:A:23:VAL:HG12	6	0.29
(1,227)	1:A:11:PHE:HE2	1:A:23:VAL:HG13	6	0.29
(1,2135)	1:A:85:ASP:HA	1:A:86:THR:HG21	7	0.29
(1,2135)	1:A:85:ASP:HA	1:A:86:THR:HG22	7	0.29
(1,2135)	1:A:85:ASP:HA	1:A:86:THR:HG23	7	0.29
(1,1959)	1:A:77:VAL:HG11	1:A:102:TYR:HB2	6	0.29
(1,1959)	1:A:77:VAL:HG12	1:A:102:TYR:HB2	6	0.29
(1,1959)	1:A:77:VAL:HG13	1:A:102:TYR:HB2	6	0.29
(1,1959)	1:A:77:VAL:HG11	1:A:102:TYR:HB2	8	0.29
(1,1959)	1:A:77:VAL:HG12	1:A:102:TYR:HB2	8	0.29
(1,1959)	1:A:77:VAL:HG13	1:A:102:TYR:HB2	8	0.29
(1,1851)	1:A:75:PHE:HB2	1:A:87:LEU:HD21	10	0.29
(1,1851)	1:A:75:PHE:HB2	1:A:87:LEU:HD22	10	0.29
(1,1851)	1:A:75:PHE:HB2	1:A:87:LEU:HD23	10	0.29
(1,154)	1:A:8:GLN:HE22	1:A:11:PHE:HD1	3	0.29
(1,154)	1:A:8:GLN:HE22	1:A:11:PHE:HD2	3	0.29
(1,154)	1:A:8:GLN:HE22	1:A:11:PHE:HD1	7	0.29
(1,154)	1:A:8:GLN:HE22	1:A:11:PHE:HD2	7	0.29
(1,154)	1:A:8:GLN:HE22	1:A:11:PHE:HD1	10	0.29
(1,154)	1:A:8:GLN:HE22	1:A:11:PHE:HD2	10	0.29
(1,1378)	1:A:47:TYR:HD1	1:A:99:ILE:HG21	8	0.29

Continued on next page...

Continued from previous page...

Key	Atom-1	Atom-2	Model ID	Violation (Å)
(1,1378)	1:A:47:TYR:HD1	1:A:99:ILE:HG22	8	0.29
(1,1378)	1:A:47:TYR:HD1	1:A:99:ILE:HG23	8	0.29
(1,1378)	1:A:47:TYR:HD2	1:A:99:ILE:HG21	8	0.29
(1,1378)	1:A:47:TYR:HD2	1:A:99:ILE:HG22	8	0.29
(1,1378)	1:A:47:TYR:HD2	1:A:99:ILE:HG23	8	0.29
(1,1377)	1:A:47:TYR:HD1	1:A:99:ILE:HG21	8	0.29
(1,1377)	1:A:47:TYR:HD1	1:A:99:ILE:HG22	8	0.29
(1,1377)	1:A:47:TYR:HD1	1:A:99:ILE:HG23	8	0.29
(1,1377)	1:A:47:TYR:HD2	1:A:99:ILE:HG21	8	0.29
(1,1377)	1:A:47:TYR:HD2	1:A:99:ILE:HG22	8	0.29
(1,1377)	1:A:47:TYR:HD2	1:A:99:ILE:HG23	8	0.29
(1,1376)	1:A:47:TYR:HD1	1:A:99:ILE:HG12	2	0.29
(1,1376)	1:A:47:TYR:HD2	1:A:99:ILE:HG12	2	0.29
(1,1376)	1:A:47:TYR:HD1	1:A:99:ILE:HG12	4	0.29
(1,1376)	1:A:47:TYR:HD2	1:A:99:ILE:HG12	4	0.29
(1,1334)	1:A:46:THR:HG21	1:A:47:TYR:HD1	6	0.29
(1,1334)	1:A:46:THR:HG21	1:A:47:TYR:HD2	6	0.29
(1,1334)	1:A:46:THR:HG22	1:A:47:TYR:HD1	6	0.29
(1,1334)	1:A:46:THR:HG22	1:A:47:TYR:HD2	6	0.29
(1,1334)	1:A:46:THR:HG23	1:A:47:TYR:HD1	6	0.29
(1,1334)	1:A:46:THR:HG23	1:A:47:TYR:HD2	6	0.29
(1,1209)	1:A:40:TYR:HE1	1:A:95:LEU:HD11	7	0.29
(1,1209)	1:A:40:TYR:HE1	1:A:95:LEU:HD12	7	0.29
(1,1209)	1:A:40:TYR:HE1	1:A:95:LEU:HD13	7	0.29
(1,1209)	1:A:40:TYR:HE2	1:A:95:LEU:HD11	7	0.29
(1,1209)	1:A:40:TYR:HE2	1:A:95:LEU:HD12	7	0.29
(1,1209)	1:A:40:TYR:HE2	1:A:95:LEU:HD13	7	0.29
(1,1088)	1:A:36:ILE:HG21	1:A:75:PHE:HZ	2	0.29
(1,1088)	1:A:36:ILE:HG22	1:A:75:PHE:HZ	2	0.29
(1,1088)	1:A:36:ILE:HG23	1:A:75:PHE:HZ	2	0.29
(1,1088)	1:A:36:ILE:HG21	1:A:75:PHE:HZ	3	0.29
(1,1088)	1:A:36:ILE:HG22	1:A:75:PHE:HZ	3	0.29
(1,1088)	1:A:36:ILE:HG23	1:A:75:PHE:HZ	3	0.29
(2,404)	1:A:28:GLU:HA	1:A:30:CYS:H	7	0.28
(2,398)	1:A:26:PHE:HD1	1:A:27:ALA:HB1	8	0.28
(2,398)	1:A:26:PHE:HD1	1:A:27:ALA:HB2	8	0.28
(2,398)	1:A:26:PHE:HD1	1:A:27:ALA:HB3	8	0.28
(2,398)	1:A:26:PHE:HD2	1:A:27:ALA:HB1	8	0.28
(2,398)	1:A:26:PHE:HD2	1:A:27:ALA:HB2	8	0.28
(2,398)	1:A:26:PHE:HD2	1:A:27:ALA:HB3	8	0.28
(2,296)	1:A:67:GLU:HB3	1:A:78:TYR:HE1	10	0.28
(2,296)	1:A:67:GLU:HB3	1:A:78:TYR:HE2	10	0.28

Continued on next page...

Continued from previous page...

Key	Atom-1	Atom-2	Model ID	Violation (Å)
(2,284)	1:A:57:VAL:HG11	1:A:61:SER:HA	9	0.28
(2,284)	1:A:57:VAL:HG12	1:A:61:SER:HA	9	0.28
(2,284)	1:A:57:VAL:HG13	1:A:61:SER:HA	9	0.28
(2,266)	1:A:50:MET:H	1:A:52:PHE:HD1	5	0.28
(2,266)	1:A:50:MET:H	1:A:52:PHE:HD2	5	0.28
(2,134)	1:A:26:PHE:HA	1:A:75:PHE:HE1	5	0.28
(2,134)	1:A:26:PHE:HA	1:A:75:PHE:HE2	5	0.28
(1,927)	1:A:27:ALA:HA	1:A:29:TRP:HE1	5	0.28
(1,927)	1:A:27:ALA:HA	1:A:29:TRP:HE1	10	0.28
(1,749)	1:A:23:VAL:HG21	1:A:54:LYS:HA	2	0.28
(1,749)	1:A:23:VAL:HG22	1:A:54:LYS:HA	2	0.28
(1,749)	1:A:23:VAL:HG23	1:A:54:LYS:HA	2	0.28
(1,735)	1:A:23:VAL:HG21	1:A:23:VAL:HG11	7	0.28
(1,735)	1:A:23:VAL:HG21	1:A:23:VAL:HG12	7	0.28
(1,735)	1:A:23:VAL:HG21	1:A:23:VAL:HG13	7	0.28
(1,735)	1:A:23:VAL:HG22	1:A:23:VAL:HG11	7	0.28
(1,735)	1:A:23:VAL:HG22	1:A:23:VAL:HG12	7	0.28
(1,735)	1:A:23:VAL:HG22	1:A:23:VAL:HG13	7	0.28
(1,735)	1:A:23:VAL:HG23	1:A:23:VAL:HG11	7	0.28
(1,735)	1:A:23:VAL:HG23	1:A:23:VAL:HG12	7	0.28
(1,735)	1:A:23:VAL:HG23	1:A:23:VAL:HG13	7	0.28
(1,654)	1:A:22:ILE:HD11	1:A:75:PHE:HB3	5	0.28
(1,654)	1:A:22:ILE:HD12	1:A:75:PHE:HB3	5	0.28
(1,654)	1:A:22:ILE:HD13	1:A:75:PHE:HB3	5	0.28
(1,2389)	1:A:98:LEU:HD21	1:A:99:ILE:H	8	0.28
(1,2389)	1:A:98:LEU:HD22	1:A:99:ILE:H	8	0.28
(1,2389)	1:A:98:LEU:HD23	1:A:99:ILE:H	8	0.28
(1,2304)	1:A:95:LEU:HD11	1:A:95:LEU:HB2	9	0.28
(1,2304)	1:A:95:LEU:HD12	1:A:95:LEU:HB2	9	0.28
(1,2304)	1:A:95:LEU:HD13	1:A:95:LEU:HB2	9	0.28
(1,2303)	1:A:95:LEU:HD11	1:A:95:LEU:HB2	9	0.28
(1,2303)	1:A:95:LEU:HD12	1:A:95:LEU:HB2	9	0.28
(1,2303)	1:A:95:LEU:HD13	1:A:95:LEU:HB2	9	0.28
(1,2197)	1:A:88:LEU:HD11	1:A:89:GLY:H	2	0.28
(1,2197)	1:A:88:LEU:HD12	1:A:89:GLY:H	2	0.28
(1,2197)	1:A:88:LEU:HD13	1:A:89:GLY:H	2	0.28
(1,2197)	1:A:88:LEU:HD21	1:A:89:GLY:H	2	0.28
(1,2197)	1:A:88:LEU:HD22	1:A:89:GLY:H	2	0.28
(1,2197)	1:A:88:LEU:HD23	1:A:89:GLY:H	2	0.28
(1,2197)	1:A:88:LEU:HD11	1:A:89:GLY:H	3	0.28
(1,2197)	1:A:88:LEU:HD12	1:A:89:GLY:H	3	0.28
(1,2197)	1:A:88:LEU:HD13	1:A:89:GLY:H	3	0.28

Continued on next page...

Continued from previous page...

Key	Atom-1	Atom-2	Model ID	Violation (Å)
(1,2197)	1:A:88:LEU:HD21	1:A:89:GLY:H	3	0.28
(1,2197)	1:A:88:LEU:HD22	1:A:89:GLY:H	3	0.28
(1,2197)	1:A:88:LEU:HD23	1:A:89:GLY:H	3	0.28
(1,1980)	1:A:77:VAL:HG21	1:A:102:TYR:HD1	3	0.28
(1,1980)	1:A:77:VAL:HG21	1:A:102:TYR:HD2	3	0.28
(1,1980)	1:A:77:VAL:HG22	1:A:102:TYR:HD1	3	0.28
(1,1980)	1:A:77:VAL:HG22	1:A:102:TYR:HD2	3	0.28
(1,1980)	1:A:77:VAL:HG23	1:A:102:TYR:HD1	3	0.28
(1,1980)	1:A:77:VAL:HG23	1:A:102:TYR:HD2	3	0.28
(1,1933)	1:A:77:VAL:HB	1:A:84:VAL:HG11	7	0.28
(1,1933)	1:A:77:VAL:HB	1:A:84:VAL:HG12	7	0.28
(1,1933)	1:A:77:VAL:HB	1:A:84:VAL:HG13	7	0.28
(1,1812)	1:A:74:THR:HG1	1:A:74:THR:HG21	8	0.28
(1,1812)	1:A:74:THR:HG1	1:A:74:THR:HG22	8	0.28
(1,1812)	1:A:74:THR:HG1	1:A:74:THR:HG23	8	0.28
(1,154)	1:A:8:GLN:HE22	1:A:11:PHE:HD1	1	0.28
(1,154)	1:A:8:GLN:HE22	1:A:11:PHE:HD2	1	0.28
(1,154)	1:A:8:GLN:HE22	1:A:11:PHE:HD1	4	0.28
(1,154)	1:A:8:GLN:HE22	1:A:11:PHE:HD2	4	0.28
(1,154)	1:A:8:GLN:HE22	1:A:11:PHE:HD1	6	0.28
(1,154)	1:A:8:GLN:HE22	1:A:11:PHE:HD2	6	0.28
(1,154)	1:A:8:GLN:HE22	1:A:11:PHE:HD1	8	0.28
(1,154)	1:A:8:GLN:HE22	1:A:11:PHE:HD2	8	0.28
(1,1421)	1:A:49:LYS:H	1:A:49:LYS:HD2	9	0.28
(1,1421)	1:A:49:LYS:H	1:A:49:LYS:HD3	9	0.28
(1,1413)	1:A:48:THR:H	1:A:48:THR:HG21	8	0.28
(1,1413)	1:A:48:THR:H	1:A:48:THR:HG22	8	0.28
(1,1413)	1:A:48:THR:H	1:A:48:THR:HG23	8	0.28
(1,1336)	1:A:46:THR:HG21	1:A:47:TYR:H	5	0.28
(1,1336)	1:A:46:THR:HG22	1:A:47:TYR:H	5	0.28
(1,1336)	1:A:46:THR:HG23	1:A:47:TYR:H	5	0.28
(1,1331)	1:A:46:THR:HG21	1:A:47:TYR:HA	5	0.28
(1,1331)	1:A:46:THR:HG22	1:A:47:TYR:HA	5	0.28
(1,1331)	1:A:46:THR:HG23	1:A:47:TYR:HA	5	0.28
(1,1267)	1:A:43:CYS:HB3	1:A:99:ILE:HG21	4	0.28
(1,1267)	1:A:43:CYS:HB3	1:A:99:ILE:HG22	4	0.28
(1,1267)	1:A:43:CYS:HB3	1:A:99:ILE:HG23	4	0.28
(2,99)	1:A:20:LEU:HA	1:A:79:LYS:HE2	9	0.27
(2,99)	1:A:20:LEU:HA	1:A:79:LYS:HE3	9	0.27
(2,98)	1:A:20:LEU:HA	1:A:79:LYS:HE2	9	0.27
(2,98)	1:A:20:LEU:HA	1:A:79:LYS:HE3	9	0.27
(2,398)	1:A:26:PHE:HD1	1:A:27:ALA:HB1	2	0.27

Continued on next page...

Continued from previous page...

Key	Atom-1	Atom-2	Model ID	Violation (Å)
(2,398)	1:A:26:PHE:HD1	1:A:27:ALA:HB2	2	0.27
(2,398)	1:A:26:PHE:HD1	1:A:27:ALA:HB3	2	0.27
(2,398)	1:A:26:PHE:HD2	1:A:27:ALA:HB1	2	0.27
(2,398)	1:A:26:PHE:HD2	1:A:27:ALA:HB2	2	0.27
(2,398)	1:A:26:PHE:HD2	1:A:27:ALA:HB3	2	0.27
(2,398)	1:A:26:PHE:HD1	1:A:27:ALA:HB1	3	0.27
(2,398)	1:A:26:PHE:HD1	1:A:27:ALA:HB2	3	0.27
(2,398)	1:A:26:PHE:HD1	1:A:27:ALA:HB3	3	0.27
(2,398)	1:A:26:PHE:HD2	1:A:27:ALA:HB1	3	0.27
(2,398)	1:A:26:PHE:HD2	1:A:27:ALA:HB2	3	0.27
(2,398)	1:A:26:PHE:HD2	1:A:27:ALA:HB3	3	0.27
(2,398)	1:A:26:PHE:HD1	1:A:27:ALA:HB1	4	0.27
(2,398)	1:A:26:PHE:HD1	1:A:27:ALA:HB2	4	0.27
(2,398)	1:A:26:PHE:HD1	1:A:27:ALA:HB3	4	0.27
(2,398)	1:A:26:PHE:HD2	1:A:27:ALA:HB1	4	0.27
(2,398)	1:A:26:PHE:HD2	1:A:27:ALA:HB2	4	0.27
(2,398)	1:A:26:PHE:HD2	1:A:27:ALA:HB3	4	0.27
(2,398)	1:A:26:PHE:HD1	1:A:27:ALA:HB1	6	0.27
(2,398)	1:A:26:PHE:HD1	1:A:27:ALA:HB2	6	0.27
(2,398)	1:A:26:PHE:HD1	1:A:27:ALA:HB3	6	0.27
(2,398)	1:A:26:PHE:HD2	1:A:27:ALA:HB1	6	0.27
(2,398)	1:A:26:PHE:HD2	1:A:27:ALA:HB2	6	0.27
(2,398)	1:A:26:PHE:HD2	1:A:27:ALA:HB3	6	0.27
(2,325)	1:A:69:ILE:HG21	1:A:71:SER:HA	5	0.27
(2,325)	1:A:69:ILE:HG22	1:A:71:SER:HA	5	0.27
(2,325)	1:A:69:ILE:HG23	1:A:71:SER:HA	5	0.27
(2,151)	1:A:27:ALA:HB1	1:A:58:ASP:HB2	9	0.27
(2,151)	1:A:27:ALA:HB2	1:A:58:ASP:HB2	9	0.27
(2,151)	1:A:27:ALA:HB3	1:A:58:ASP:HB2	9	0.27
(1,927)	1:A:27:ALA:HA	1:A:29:TRP:HE1	1	0.27
(1,927)	1:A:27:ALA:HA	1:A:29:TRP:HE1	2	0.27
(1,927)	1:A:27:ALA:HA	1:A:29:TRP:HE1	4	0.27
(1,845)	1:A:25:PHE:HE1	1:A:76:LYS:HE2	5	0.27
(1,845)	1:A:25:PHE:HE2	1:A:76:LYS:HE2	5	0.27
(1,838)	1:A:25:PHE:HE1	1:A:63:VAL:HG11	7	0.27
(1,838)	1:A:25:PHE:HE1	1:A:63:VAL:HG12	7	0.27
(1,838)	1:A:25:PHE:HE1	1:A:63:VAL:HG13	7	0.27
(1,838)	1:A:25:PHE:HE2	1:A:63:VAL:HG11	7	0.27
(1,838)	1:A:25:PHE:HE2	1:A:63:VAL:HG12	7	0.27
(1,838)	1:A:25:PHE:HE2	1:A:63:VAL:HG13	7	0.27
(1,826)	1:A:25:PHE:HD1	1:A:25:PHE:H	7	0.27
(1,826)	1:A:25:PHE:HD2	1:A:25:PHE:H	7	0.27

Continued on next page...

Continued from previous page...

Key	Atom-1	Atom-2	Model ID	Violation (Å)
(1,752)	1:A:23:VAL:HG21	1:A:55:VAL:HG21	2	0.27
(1,752)	1:A:23:VAL:HG21	1:A:55:VAL:HG22	2	0.27
(1,752)	1:A:23:VAL:HG21	1:A:55:VAL:HG23	2	0.27
(1,752)	1:A:23:VAL:HG22	1:A:55:VAL:HG21	2	0.27
(1,752)	1:A:23:VAL:HG22	1:A:55:VAL:HG22	2	0.27
(1,752)	1:A:23:VAL:HG22	1:A:55:VAL:HG23	2	0.27
(1,752)	1:A:23:VAL:HG23	1:A:55:VAL:HG21	2	0.27
(1,752)	1:A:23:VAL:HG23	1:A:55:VAL:HG22	2	0.27
(1,752)	1:A:23:VAL:HG23	1:A:55:VAL:HG23	2	0.27
(1,731)	1:A:23:VAL:HG11	1:A:78:TYR:HE1	4	0.27
(1,731)	1:A:23:VAL:HG11	1:A:78:TYR:HE2	4	0.27
(1,731)	1:A:23:VAL:HG12	1:A:78:TYR:HE1	4	0.27
(1,731)	1:A:23:VAL:HG12	1:A:78:TYR:HE2	4	0.27
(1,731)	1:A:23:VAL:HG13	1:A:78:TYR:HE1	4	0.27
(1,731)	1:A:23:VAL:HG13	1:A:78:TYR:HE2	4	0.27
(1,731)	1:A:23:VAL:HG11	1:A:78:TYR:HE1	10	0.27
(1,731)	1:A:23:VAL:HG11	1:A:78:TYR:HE2	10	0.27
(1,731)	1:A:23:VAL:HG12	1:A:78:TYR:HE1	10	0.27
(1,731)	1:A:23:VAL:HG12	1:A:78:TYR:HE2	10	0.27
(1,731)	1:A:23:VAL:HG13	1:A:78:TYR:HE1	10	0.27
(1,731)	1:A:23:VAL:HG13	1:A:78:TYR:HE2	10	0.27
(1,380)	1:A:15:ILE:HD11	1:A:23:VAL:HB	7	0.27
(1,380)	1:A:15:ILE:HD12	1:A:23:VAL:HB	7	0.27
(1,380)	1:A:15:ILE:HD13	1:A:23:VAL:HB	7	0.27
(1,2415)	1:A:99:ILE:HD11	1:A:103:ALA:H	1	0.27
(1,2415)	1:A:99:ILE:HD12	1:A:103:ALA:H	1	0.27
(1,2415)	1:A:99:ILE:HD13	1:A:103:ALA:H	1	0.27
(1,2351)	1:A:97:GLN:HE21	1:A:97:GLN:HB2	3	0.27
(1,2351)	1:A:97:GLN:HE21	1:A:97:GLN:HB3	3	0.27
(1,2304)	1:A:95:LEU:HD11	1:A:95:LEU:HB2	2	0.27
(1,2304)	1:A:95:LEU:HD12	1:A:95:LEU:HB2	2	0.27
(1,2304)	1:A:95:LEU:HD13	1:A:95:LEU:HB2	2	0.27
(1,2304)	1:A:95:LEU:HD11	1:A:95:LEU:HB2	5	0.27
(1,2304)	1:A:95:LEU:HD12	1:A:95:LEU:HB2	5	0.27
(1,2304)	1:A:95:LEU:HD13	1:A:95:LEU:HB2	5	0.27
(1,2304)	1:A:95:LEU:HD11	1:A:95:LEU:HB2	10	0.27
(1,2304)	1:A:95:LEU:HD12	1:A:95:LEU:HB2	10	0.27
(1,2304)	1:A:95:LEU:HD13	1:A:95:LEU:HB2	10	0.27
(1,2303)	1:A:95:LEU:HD11	1:A:95:LEU:HB2	2	0.27
(1,2303)	1:A:95:LEU:HD12	1:A:95:LEU:HB2	2	0.27
(1,2303)	1:A:95:LEU:HD13	1:A:95:LEU:HB2	2	0.27
(1,2303)	1:A:95:LEU:HD11	1:A:95:LEU:HB2	5	0.27

Continued on next page...

Continued from previous page...

Key	Atom-1	Atom-2	Model ID	Violation (Å)
(1,2303)	1:A:95:LEU:HD12	1:A:95:LEU:HB2	5	0.27
(1,2303)	1:A:95:LEU:HD13	1:A:95:LEU:HB2	5	0.27
(1,2303)	1:A:95:LEU:HD11	1:A:95:LEU:HB2	10	0.27
(1,2303)	1:A:95:LEU:HD12	1:A:95:LEU:HB2	10	0.27
(1,2303)	1:A:95:LEU:HD13	1:A:95:LEU:HB2	10	0.27
(1,2244)	1:A:91:ASN:HD21	1:A:94:ALA:HB1	6	0.27
(1,2244)	1:A:91:ASN:HD21	1:A:94:ALA:HB2	6	0.27
(1,2244)	1:A:91:ASN:HD21	1:A:94:ALA:HB3	6	0.27
(1,2200)	1:A:88:LEU:HD21	1:A:88:LEU:HA	2	0.27
(1,2200)	1:A:88:LEU:HD22	1:A:88:LEU:HA	2	0.27
(1,2200)	1:A:88:LEU:HD23	1:A:88:LEU:HA	2	0.27
(1,2135)	1:A:85:ASP:HA	1:A:86:THR:HG21	10	0.27
(1,2135)	1:A:85:ASP:HA	1:A:86:THR:HG22	10	0.27
(1,2135)	1:A:85:ASP:HA	1:A:86:THR:HG23	10	0.27
(1,1950)	1:A:77:VAL:HG11	1:A:84:VAL:HG11	8	0.27
(1,1950)	1:A:77:VAL:HG11	1:A:84:VAL:HG12	8	0.27
(1,1950)	1:A:77:VAL:HG11	1:A:84:VAL:HG13	8	0.27
(1,1950)	1:A:77:VAL:HG12	1:A:84:VAL:HG11	8	0.27
(1,1950)	1:A:77:VAL:HG12	1:A:84:VAL:HG12	8	0.27
(1,1950)	1:A:77:VAL:HG12	1:A:84:VAL:HG13	8	0.27
(1,1950)	1:A:77:VAL:HG13	1:A:84:VAL:HG11	8	0.27
(1,1950)	1:A:77:VAL:HG13	1:A:84:VAL:HG12	8	0.27
(1,1950)	1:A:77:VAL:HG13	1:A:84:VAL:HG13	8	0.27
(1,1421)	1:A:49:LYS:H	1:A:49:LYS:HD2	8	0.27
(1,1421)	1:A:49:LYS:H	1:A:49:LYS:HD3	8	0.27
(1,1421)	1:A:49:LYS:H	1:A:49:LYS:HD2	10	0.27
(1,1421)	1:A:49:LYS:H	1:A:49:LYS:HD3	10	0.27
(1,1267)	1:A:43:CYS:HB3	1:A:99:ILE:HG21	5	0.27
(1,1267)	1:A:43:CYS:HB3	1:A:99:ILE:HG22	5	0.27
(1,1267)	1:A:43:CYS:HB3	1:A:99:ILE:HG23	5	0.27
(1,1155)	1:A:39:PHE:HD1	1:A:95:LEU:HD21	5	0.27
(1,1155)	1:A:39:PHE:HD1	1:A:95:LEU:HD22	5	0.27
(1,1155)	1:A:39:PHE:HD1	1:A:95:LEU:HD23	5	0.27
(1,1155)	1:A:39:PHE:HD2	1:A:95:LEU:HD21	5	0.27
(1,1155)	1:A:39:PHE:HD2	1:A:95:LEU:HD22	5	0.27
(1,1155)	1:A:39:PHE:HD2	1:A:95:LEU:HD23	5	0.27
(1,112)	1:A:6:THR:H	1:A:6:THR:HG21	4	0.27
(1,112)	1:A:6:THR:H	1:A:6:THR:HG22	4	0.27
(1,112)	1:A:6:THR:H	1:A:6:THR:HG23	4	0.27
(1,1088)	1:A:36:ILE:HG21	1:A:75:PHE:HZ	7	0.27
(1,1088)	1:A:36:ILE:HG22	1:A:75:PHE:HZ	7	0.27
(1,1088)	1:A:36:ILE:HG23	1:A:75:PHE:HZ	7	0.27

Continued on next page...

Continued from previous page...

Key	Atom-1	Atom-2	Model ID	Violation (Å)
(1,1088)	1:A:36:ILE:HG21	1:A:75:PHE:HZ	9	0.27
(1,1088)	1:A:36:ILE:HG22	1:A:75:PHE:HZ	9	0.27
(1,1088)	1:A:36:ILE:HG23	1:A:75:PHE:HZ	9	0.27
(2,83)	1:A:12:ASP:HB2	1:A:66:LYS:HE2	6	0.26
(2,83)	1:A:12:ASP:HB2	1:A:66:LYS:HE3	6	0.26
(2,402)	1:A:27:ALA:HB1	1:A:30:CYS:H	6	0.26
(2,402)	1:A:27:ALA:HB2	1:A:30:CYS:H	6	0.26
(2,402)	1:A:27:ALA:HB3	1:A:30:CYS:H	6	0.26
(2,284)	1:A:57:VAL:HG11	1:A:61:SER:HA	2	0.26
(2,284)	1:A:57:VAL:HG12	1:A:61:SER:HA	2	0.26
(2,284)	1:A:57:VAL:HG13	1:A:61:SER:HA	2	0.26
(2,284)	1:A:57:VAL:HG11	1:A:61:SER:HA	6	0.26
(2,284)	1:A:57:VAL:HG12	1:A:61:SER:HA	6	0.26
(2,284)	1:A:57:VAL:HG13	1:A:61:SER:HA	6	0.26
(2,265)	1:A:50:MET:HE1	1:A:102:TYR:HE1	7	0.26
(2,265)	1:A:50:MET:HE1	1:A:102:TYR:HE2	7	0.26
(2,265)	1:A:50:MET:HE2	1:A:102:TYR:HE1	7	0.26
(2,265)	1:A:50:MET:HE2	1:A:102:TYR:HE2	7	0.26
(2,265)	1:A:50:MET:HE3	1:A:102:TYR:HE1	7	0.26
(2,265)	1:A:50:MET:HE3	1:A:102:TYR:HE2	7	0.26
(1,927)	1:A:27:ALA:HA	1:A:29:TRP:HE1	3	0.26
(1,927)	1:A:27:ALA:HA	1:A:29:TRP:HE1	9	0.26
(1,826)	1:A:25:PHE:HD1	1:A:25:PHE:H	4	0.26
(1,826)	1:A:25:PHE:HD2	1:A:25:PHE:H	4	0.26
(1,826)	1:A:25:PHE:HD1	1:A:25:PHE:H	8	0.26
(1,826)	1:A:25:PHE:HD2	1:A:25:PHE:H	8	0.26
(1,798)	1:A:25:PHE:HA	1:A:25:PHE:HD1	9	0.26
(1,798)	1:A:25:PHE:HA	1:A:25:PHE:HD2	9	0.26
(1,752)	1:A:23:VAL:HG21	1:A:55:VAL:HG21	3	0.26
(1,752)	1:A:23:VAL:HG21	1:A:55:VAL:HG22	3	0.26
(1,752)	1:A:23:VAL:HG21	1:A:55:VAL:HG23	3	0.26
(1,752)	1:A:23:VAL:HG22	1:A:55:VAL:HG21	3	0.26
(1,752)	1:A:23:VAL:HG22	1:A:55:VAL:HG22	3	0.26
(1,752)	1:A:23:VAL:HG22	1:A:55:VAL:HG23	3	0.26
(1,752)	1:A:23:VAL:HG23	1:A:55:VAL:HG21	3	0.26
(1,752)	1:A:23:VAL:HG23	1:A:55:VAL:HG22	3	0.26
(1,752)	1:A:23:VAL:HG23	1:A:55:VAL:HG23	3	0.26
(1,752)	1:A:23:VAL:HG21	1:A:55:VAL:HG21	7	0.26
(1,752)	1:A:23:VAL:HG21	1:A:55:VAL:HG22	7	0.26
(1,752)	1:A:23:VAL:HG21	1:A:55:VAL:HG23	7	0.26
(1,752)	1:A:23:VAL:HG22	1:A:55:VAL:HG21	7	0.26
(1,752)	1:A:23:VAL:HG22	1:A:55:VAL:HG22	7	0.26

Continued on next page...

Continued from previous page...

Key	Atom-1	Atom-2	Model ID	Violation (Å)
(1,752)	1:A:23:VAL:HG22	1:A:55:VAL:HG23	7	0.26
(1,752)	1:A:23:VAL:HG23	1:A:55:VAL:HG21	7	0.26
(1,752)	1:A:23:VAL:HG23	1:A:55:VAL:HG22	7	0.26
(1,752)	1:A:23:VAL:HG23	1:A:55:VAL:HG23	7	0.26
(1,749)	1:A:23:VAL:HG21	1:A:54:LYS:HA	10	0.26
(1,749)	1:A:23:VAL:HG22	1:A:54:LYS:HA	10	0.26
(1,749)	1:A:23:VAL:HG23	1:A:54:LYS:HA	10	0.26
(1,425)	1:A:15:ILE:HG21	1:A:78:TYR:HB2	9	0.26
(1,425)	1:A:15:ILE:HG22	1:A:78:TYR:HB2	9	0.26
(1,425)	1:A:15:ILE:HG23	1:A:78:TYR:HB2	9	0.26
(1,30)	1:A:2:VAL:H	1:A:2:VAL:HG21	2	0.26
(1,30)	1:A:2:VAL:H	1:A:2:VAL:HG22	2	0.26
(1,30)	1:A:2:VAL:H	1:A:2:VAL:HG23	2	0.26
(1,2388)	1:A:98:LEU:HD21	1:A:99:ILE:HA	8	0.26
(1,2388)	1:A:98:LEU:HD22	1:A:99:ILE:HA	8	0.26
(1,2388)	1:A:98:LEU:HD23	1:A:99:ILE:HA	8	0.26
(1,2304)	1:A:95:LEU:HD11	1:A:95:LEU:HB2	7	0.26
(1,2304)	1:A:95:LEU:HD12	1:A:95:LEU:HB2	7	0.26
(1,2304)	1:A:95:LEU:HD13	1:A:95:LEU:HB2	7	0.26
(1,2303)	1:A:95:LEU:HD11	1:A:95:LEU:HB2	7	0.26
(1,2303)	1:A:95:LEU:HD12	1:A:95:LEU:HB2	7	0.26
(1,2303)	1:A:95:LEU:HD13	1:A:95:LEU:HB2	7	0.26
(1,1816)	1:A:74:THR:HG21	1:A:74:THR:HA	6	0.26
(1,1816)	1:A:74:THR:HG22	1:A:74:THR:HA	6	0.26
(1,1816)	1:A:74:THR:HG23	1:A:74:THR:HA	6	0.26
(1,1816)	1:A:74:THR:HG21	1:A:74:THR:HA	9	0.26
(1,1816)	1:A:74:THR:HG22	1:A:74:THR:HA	9	0.26
(1,1816)	1:A:74:THR:HG23	1:A:74:THR:HA	9	0.26
(1,1270)	1:A:43:CYS:HB2	1:A:47:TYR:HD1	8	0.26
(1,1270)	1:A:43:CYS:HB2	1:A:47:TYR:HD2	8	0.26
(1,1088)	1:A:36:ILE:HG21	1:A:75:PHE:HZ	10	0.26
(1,1088)	1:A:36:ILE:HG22	1:A:75:PHE:HZ	10	0.26
(1,1088)	1:A:36:ILE:HG23	1:A:75:PHE:HZ	10	0.26
(2,404)	1:A:28:GLU:HA	1:A:30:CYS:H	5	0.25
(2,402)	1:A:27:ALA:HB1	1:A:30:CYS:H	3	0.25
(2,402)	1:A:27:ALA:HB2	1:A:30:CYS:H	3	0.25
(2,402)	1:A:27:ALA:HB3	1:A:30:CYS:H	3	0.25
(2,401)	1:A:27:ALA:HB1	1:A:30:CYS:HB3	5	0.25
(2,401)	1:A:27:ALA:HB2	1:A:30:CYS:HB3	5	0.25
(2,401)	1:A:27:ALA:HB3	1:A:30:CYS:HB3	5	0.25
(2,398)	1:A:26:PHE:HD1	1:A:27:ALA:HB1	5	0.25
(2,398)	1:A:26:PHE:HD1	1:A:27:ALA:HB2	5	0.25

Continued on next page...

Continued from previous page...

Key	Atom-1	Atom-2	Model ID	Violation (Å)
(2,398)	1:A:26:PHE:HD1	1:A:27:ALA:HB3	5	0.25
(2,398)	1:A:26:PHE:HD2	1:A:27:ALA:HB1	5	0.25
(2,398)	1:A:26:PHE:HD2	1:A:27:ALA:HB2	5	0.25
(2,398)	1:A:26:PHE:HD2	1:A:27:ALA:HB3	5	0.25
(2,244)	1:A:47:TYR:HA	1:A:52:PHE:HE1	4	0.25
(2,244)	1:A:47:TYR:HA	1:A:52:PHE:HE2	4	0.25
(1,913)	1:A:26:PHE:H	1:A:26:PHE:HD1	7	0.25
(1,913)	1:A:26:PHE:H	1:A:26:PHE:HD2	7	0.25
(1,826)	1:A:25:PHE:HD1	1:A:25:PHE:H	6	0.25
(1,826)	1:A:25:PHE:HD2	1:A:25:PHE:H	6	0.25
(1,665)	1:A:22:ILE:HG21	1:A:50:MET:HB3	3	0.25
(1,665)	1:A:22:ILE:HG22	1:A:50:MET:HB3	3	0.25
(1,665)	1:A:22:ILE:HG23	1:A:50:MET:HB3	3	0.25
(1,375)	1:A:15:ILE:HD11	1:A:21:VAL:HG11	9	0.25
(1,375)	1:A:15:ILE:HD11	1:A:21:VAL:HG12	9	0.25
(1,375)	1:A:15:ILE:HD11	1:A:21:VAL:HG13	9	0.25
(1,375)	1:A:15:ILE:HD12	1:A:21:VAL:HG11	9	0.25
(1,375)	1:A:15:ILE:HD12	1:A:21:VAL:HG12	9	0.25
(1,375)	1:A:15:ILE:HD12	1:A:21:VAL:HG13	9	0.25
(1,375)	1:A:15:ILE:HD13	1:A:21:VAL:HG11	9	0.25
(1,375)	1:A:15:ILE:HD13	1:A:21:VAL:HG12	9	0.25
(1,375)	1:A:15:ILE:HD13	1:A:21:VAL:HG13	9	0.25
(1,362)	1:A:15:ILE:HD11	1:A:15:ILE:HA	1	0.25
(1,362)	1:A:15:ILE:HD12	1:A:15:ILE:HA	1	0.25
(1,362)	1:A:15:ILE:HD13	1:A:15:ILE:HA	1	0.25
(1,304)	1:A:14:ILE:HA	1:A:14:ILE:HD11	1	0.25
(1,304)	1:A:14:ILE:HA	1:A:14:ILE:HD12	1	0.25
(1,304)	1:A:14:ILE:HA	1:A:14:ILE:HD13	1	0.25
(1,304)	1:A:14:ILE:HA	1:A:14:ILE:HD11	8	0.25
(1,304)	1:A:14:ILE:HA	1:A:14:ILE:HD12	8	0.25
(1,304)	1:A:14:ILE:HA	1:A:14:ILE:HD13	8	0.25
(1,304)	1:A:14:ILE:HA	1:A:14:ILE:HD11	10	0.25
(1,304)	1:A:14:ILE:HA	1:A:14:ILE:HD12	10	0.25
(1,304)	1:A:14:ILE:HA	1:A:14:ILE:HD13	10	0.25
(1,2304)	1:A:95:LEU:HD11	1:A:95:LEU:HB2	8	0.25
(1,2304)	1:A:95:LEU:HD12	1:A:95:LEU:HB2	8	0.25
(1,2304)	1:A:95:LEU:HD13	1:A:95:LEU:HB2	8	0.25
(1,2303)	1:A:95:LEU:HD11	1:A:95:LEU:HB2	8	0.25
(1,2303)	1:A:95:LEU:HD12	1:A:95:LEU:HB2	8	0.25
(1,2303)	1:A:95:LEU:HD13	1:A:95:LEU:HB2	8	0.25
(1,227)	1:A:11:PHE:HE1	1:A:23:VAL:HG11	1	0.25
(1,227)	1:A:11:PHE:HE1	1:A:23:VAL:HG12	1	0.25

Continued on next page...

Continued from previous page...

Key	Atom-1	Atom-2	Model ID	Violation (Å)
(1,227)	1:A:11:PHE:HE1	1:A:23:VAL:HG13	1	0.25
(1,227)	1:A:11:PHE:HE2	1:A:23:VAL:HG11	1	0.25
(1,227)	1:A:11:PHE:HE2	1:A:23:VAL:HG12	1	0.25
(1,227)	1:A:11:PHE:HE2	1:A:23:VAL:HG13	1	0.25
(1,2197)	1:A:88:LEU:HD11	1:A:89:GLY:H	9	0.25
(1,2197)	1:A:88:LEU:HD12	1:A:89:GLY:H	9	0.25
(1,2197)	1:A:88:LEU:HD13	1:A:89:GLY:H	9	0.25
(1,2197)	1:A:88:LEU:HD21	1:A:89:GLY:H	9	0.25
(1,2197)	1:A:88:LEU:HD22	1:A:89:GLY:H	9	0.25
(1,2197)	1:A:88:LEU:HD23	1:A:89:GLY:H	9	0.25
(1,2054)	1:A:79:LYS:HE2	1:A:80:ASN:HD22	1	0.25
(1,2054)	1:A:79:LYS:HE3	1:A:80:ASN:HD22	1	0.25
(1,1963)	1:A:77:VAL:HG21	1:A:77:VAL:HA	3	0.25
(1,1963)	1:A:77:VAL:HG22	1:A:77:VAL:HA	3	0.25
(1,1963)	1:A:77:VAL:HG23	1:A:77:VAL:HA	3	0.25
(1,1816)	1:A:74:THR:HG21	1:A:74:THR:HA	1	0.25
(1,1816)	1:A:74:THR:HG22	1:A:74:THR:HA	1	0.25
(1,1816)	1:A:74:THR:HG23	1:A:74:THR:HA	1	0.25
(1,1816)	1:A:74:THR:HG21	1:A:74:THR:HA	2	0.25
(1,1816)	1:A:74:THR:HG22	1:A:74:THR:HA	2	0.25
(1,1816)	1:A:74:THR:HG23	1:A:74:THR:HA	2	0.25
(1,1812)	1:A:74:THR:HG1	1:A:74:THR:HG21	5	0.25
(1,1812)	1:A:74:THR:HG1	1:A:74:THR:HG22	5	0.25
(1,1812)	1:A:74:THR:HG1	1:A:74:THR:HG23	5	0.25
(1,1413)	1:A:48:THR:H	1:A:48:THR:HG21	4	0.25
(1,1413)	1:A:48:THR:H	1:A:48:THR:HG22	4	0.25
(1,1413)	1:A:48:THR:H	1:A:48:THR:HG23	4	0.25
(1,1155)	1:A:39:PHE:HD1	1:A:95:LEU:HD21	2	0.25
(1,1155)	1:A:39:PHE:HD1	1:A:95:LEU:HD22	2	0.25
(1,1155)	1:A:39:PHE:HD1	1:A:95:LEU:HD23	2	0.25
(1,1155)	1:A:39:PHE:HD2	1:A:95:LEU:HD21	2	0.25
(1,1155)	1:A:39:PHE:HD2	1:A:95:LEU:HD22	2	0.25
(1,1155)	1:A:39:PHE:HD2	1:A:95:LEU:HD23	2	0.25
(1,112)	1:A:6:THR:H	1:A:6:THR:HG21	2	0.25
(1,112)	1:A:6:THR:H	1:A:6:THR:HG22	2	0.25
(1,112)	1:A:6:THR:H	1:A:6:THR:HG23	2	0.25
(1,112)	1:A:6:THR:H	1:A:6:THR:HG21	9	0.25
(1,112)	1:A:6:THR:H	1:A:6:THR:HG22	9	0.25
(1,112)	1:A:6:THR:H	1:A:6:THR:HG23	9	0.25
(1,1088)	1:A:36:ILE:HG21	1:A:75:PHE:HZ	4	0.25
(1,1088)	1:A:36:ILE:HG22	1:A:75:PHE:HZ	4	0.25
(1,1088)	1:A:36:ILE:HG23	1:A:75:PHE:HZ	4	0.25

Continued on next page...

Continued from previous page...

Key	Atom-1	Atom-2	Model ID	Violation (Å)
(1,1088)	1:A:36:ILE:HG21	1:A:75:PHE:HZ	5	0.25
(1,1088)	1:A:36:ILE:HG22	1:A:75:PHE:HZ	5	0.25
(1,1088)	1:A:36:ILE:HG23	1:A:75:PHE:HZ	5	0.25
(2,79)	1:A:12:ASP:HB3	1:A:66:LYS:HD2	4	0.24
(2,79)	1:A:12:ASP:HB3	1:A:66:LYS:HD3	4	0.24
(2,404)	1:A:28:GLU:HA	1:A:30:CYS:H	4	0.24
(2,402)	1:A:27:ALA:HB1	1:A:30:CYS:H	5	0.24
(2,402)	1:A:27:ALA:HB2	1:A:30:CYS:H	5	0.24
(2,402)	1:A:27:ALA:HB3	1:A:30:CYS:H	5	0.24
(2,264)	1:A:50:MET:HE1	1:A:99:ILE:HA	4	0.24
(2,264)	1:A:50:MET:HE2	1:A:99:ILE:HA	4	0.24
(2,264)	1:A:50:MET:HE3	1:A:99:ILE:HA	4	0.24
(2,247)	1:A:47:TYR:HB2	1:A:52:PHE:HD1	3	0.24
(2,247)	1:A:47:TYR:HB2	1:A:52:PHE:HD2	3	0.24
(2,244)	1:A:47:TYR:HA	1:A:52:PHE:HE1	8	0.24
(2,244)	1:A:47:TYR:HA	1:A:52:PHE:HE2	8	0.24
(2,131)	1:A:26:PHE:HA	1:A:72:MET:HE1	4	0.24
(2,131)	1:A:26:PHE:HA	1:A:72:MET:HE2	4	0.24
(2,131)	1:A:26:PHE:HA	1:A:72:MET:HE3	4	0.24
(1,936)	1:A:27:ALA:H	1:A:27:ALA:HB1	3	0.24
(1,936)	1:A:27:ALA:H	1:A:27:ALA:HB2	3	0.24
(1,936)	1:A:27:ALA:H	1:A:27:ALA:HB3	3	0.24
(1,936)	1:A:27:ALA:H	1:A:27:ALA:HB1	8	0.24
(1,936)	1:A:27:ALA:H	1:A:27:ALA:HB2	8	0.24
(1,936)	1:A:27:ALA:H	1:A:27:ALA:HB3	8	0.24
(1,933)	1:A:27:ALA:HB1	1:A:57:VAL:H	6	0.24
(1,933)	1:A:27:ALA:HB2	1:A:57:VAL:H	6	0.24
(1,933)	1:A:27:ALA:HB3	1:A:57:VAL:H	6	0.24
(1,913)	1:A:26:PHE:H	1:A:26:PHE:HD1	6	0.24
(1,913)	1:A:26:PHE:H	1:A:26:PHE:HD2	6	0.24
(1,749)	1:A:23:VAL:HG21	1:A:54:LYS:HA	5	0.24
(1,749)	1:A:23:VAL:HG22	1:A:54:LYS:HA	5	0.24
(1,749)	1:A:23:VAL:HG23	1:A:54:LYS:HA	5	0.24
(1,746)	1:A:23:VAL:HG21	1:A:53:ILE:HG13	8	0.24
(1,746)	1:A:23:VAL:HG22	1:A:53:ILE:HG13	8	0.24
(1,746)	1:A:23:VAL:HG23	1:A:53:ILE:HG13	8	0.24
(1,745)	1:A:23:VAL:HG21	1:A:53:ILE:HB	7	0.24
(1,745)	1:A:23:VAL:HG22	1:A:53:ILE:HB	7	0.24
(1,745)	1:A:23:VAL:HG23	1:A:53:ILE:HB	7	0.24
(1,684)	1:A:22:ILE:H	1:A:22:ILE:HG21	7	0.24
(1,684)	1:A:22:ILE:H	1:A:22:ILE:HG22	7	0.24
(1,684)	1:A:22:ILE:H	1:A:22:ILE:HG23	7	0.24

Continued on next page...

Continued from previous page...

Key	Atom-1	Atom-2	Model ID	Violation (Å)
(1,433)	1:A:15:ILE:HG21	1:A:82:SER:HA	2	0.24
(1,433)	1:A:15:ILE:HG22	1:A:82:SER:HA	2	0.24
(1,433)	1:A:15:ILE:HG23	1:A:82:SER:HA	2	0.24
(1,362)	1:A:15:ILE:HD11	1:A:15:ILE:HA	6	0.24
(1,362)	1:A:15:ILE:HD12	1:A:15:ILE:HA	6	0.24
(1,362)	1:A:15:ILE:HD13	1:A:15:ILE:HA	6	0.24
(1,343)	1:A:14:ILE:HG21	1:A:18:ASN:HD22	10	0.24
(1,343)	1:A:14:ILE:HG22	1:A:18:ASN:HD22	10	0.24
(1,343)	1:A:14:ILE:HG23	1:A:18:ASN:HD22	10	0.24
(1,2304)	1:A:95:LEU:HD11	1:A:95:LEU:HB2	1	0.24
(1,2304)	1:A:95:LEU:HD12	1:A:95:LEU:HB2	1	0.24
(1,2304)	1:A:95:LEU:HD13	1:A:95:LEU:HB2	1	0.24
(1,2303)	1:A:95:LEU:HD11	1:A:95:LEU:HB2	1	0.24
(1,2303)	1:A:95:LEU:HD12	1:A:95:LEU:HB2	1	0.24
(1,2303)	1:A:95:LEU:HD13	1:A:95:LEU:HB2	1	0.24
(1,2139)	1:A:85:ASP:HB3	1:A:98:LEU:HD11	1	0.24
(1,2139)	1:A:85:ASP:HB3	1:A:98:LEU:HD12	1	0.24
(1,2139)	1:A:85:ASP:HB3	1:A:98:LEU:HD13	1	0.24
(1,2135)	1:A:85:ASP:HA	1:A:86:THR:HG21	5	0.24
(1,2135)	1:A:85:ASP:HA	1:A:86:THR:HG22	5	0.24
(1,2135)	1:A:85:ASP:HA	1:A:86:THR:HG23	5	0.24
(1,2053)	1:A:79:LYS:HE2	1:A:80:ASN:HD21	6	0.24
(1,2053)	1:A:79:LYS:HE3	1:A:80:ASN:HD21	6	0.24
(1,1816)	1:A:74:THR:HG21	1:A:74:THR:HA	7	0.24
(1,1816)	1:A:74:THR:HG22	1:A:74:THR:HA	7	0.24
(1,1816)	1:A:74:THR:HG23	1:A:74:THR:HA	7	0.24
(1,1816)	1:A:74:THR:HG21	1:A:74:THR:HA	10	0.24
(1,1816)	1:A:74:THR:HG22	1:A:74:THR:HA	10	0.24
(1,1816)	1:A:74:THR:HG23	1:A:74:THR:HA	10	0.24
(1,1358)	1:A:47:TYR:HB3	1:A:99:ILE:HD11	4	0.24
(1,1358)	1:A:47:TYR:HB3	1:A:99:ILE:HD12	4	0.24
(1,1358)	1:A:47:TYR:HB3	1:A:99:ILE:HD13	4	0.24
(1,12)	1:A:2:VAL:HG11	1:A:3:LYS:H	10	0.24
(1,12)	1:A:2:VAL:HG12	1:A:3:LYS:H	10	0.24
(1,12)	1:A:2:VAL:HG13	1:A:3:LYS:H	10	0.24
(1,1100)	1:A:36:ILE:H	1:A:38:PRO:HD2	6	0.24
(1,1100)	1:A:36:ILE:H	1:A:38:PRO:HD3	6	0.24
(1,1088)	1:A:36:ILE:HG21	1:A:75:PHE:HZ	6	0.24
(1,1088)	1:A:36:ILE:HG22	1:A:75:PHE:HZ	6	0.24
(1,1088)	1:A:36:ILE:HG23	1:A:75:PHE:HZ	6	0.24
(2,402)	1:A:27:ALA:HB1	1:A:30:CYS:H	4	0.23
(2,402)	1:A:27:ALA:HB2	1:A:30:CYS:H	4	0.23

Continued on next page...

Continued from previous page...

Key	Atom-1	Atom-2	Model ID	Violation (Å)
(2,402)	1:A:27:ALA:HB3	1:A:30:CYS:H	4	0.23
(2,284)	1:A:57:VAL:HG11	1:A:61:SER:HA	1	0.23
(2,284)	1:A:57:VAL:HG12	1:A:61:SER:HA	1	0.23
(2,284)	1:A:57:VAL:HG13	1:A:61:SER:HA	1	0.23
(2,284)	1:A:57:VAL:HG11	1:A:61:SER:HA	4	0.23
(2,284)	1:A:57:VAL:HG12	1:A:61:SER:HA	4	0.23
(2,284)	1:A:57:VAL:HG13	1:A:61:SER:HA	4	0.23
(2,266)	1:A:50:MET:H	1:A:52:PHE:HD1	1	0.23
(2,266)	1:A:50:MET:H	1:A:52:PHE:HD2	1	0.23
(2,247)	1:A:47:TYR:HB2	1:A:52:PHE:HD1	10	0.23
(2,247)	1:A:47:TYR:HB2	1:A:52:PHE:HD2	10	0.23
(2,134)	1:A:26:PHE:HA	1:A:75:PHE:HE1	6	0.23
(2,134)	1:A:26:PHE:HA	1:A:75:PHE:HE2	6	0.23
(1,936)	1:A:27:ALA:H	1:A:27:ALA:HB1	5	0.23
(1,936)	1:A:27:ALA:H	1:A:27:ALA:HB2	5	0.23
(1,936)	1:A:27:ALA:H	1:A:27:ALA:HB3	5	0.23
(1,936)	1:A:27:ALA:H	1:A:27:ALA:HB1	7	0.23
(1,936)	1:A:27:ALA:H	1:A:27:ALA:HB2	7	0.23
(1,936)	1:A:27:ALA:H	1:A:27:ALA:HB3	7	0.23
(1,936)	1:A:27:ALA:H	1:A:27:ALA:HB1	10	0.23
(1,936)	1:A:27:ALA:H	1:A:27:ALA:HB2	10	0.23
(1,936)	1:A:27:ALA:H	1:A:27:ALA:HB3	10	0.23
(1,913)	1:A:26:PHE:H	1:A:26:PHE:HD1	8	0.23
(1,913)	1:A:26:PHE:H	1:A:26:PHE:HD2	8	0.23
(1,913)	1:A:26:PHE:H	1:A:26:PHE:HD1	10	0.23
(1,913)	1:A:26:PHE:H	1:A:26:PHE:HD2	10	0.23
(1,841)	1:A:25:PHE:HE1	1:A:69:ILE:HD11	9	0.23
(1,841)	1:A:25:PHE:HE1	1:A:69:ILE:HD12	9	0.23
(1,841)	1:A:25:PHE:HE1	1:A:69:ILE:HD13	9	0.23
(1,841)	1:A:25:PHE:HE2	1:A:69:ILE:HD11	9	0.23
(1,841)	1:A:25:PHE:HE2	1:A:69:ILE:HD12	9	0.23
(1,841)	1:A:25:PHE:HE2	1:A:69:ILE:HD13	9	0.23
(1,826)	1:A:25:PHE:HD1	1:A:25:PHE:H	2	0.23
(1,826)	1:A:25:PHE:HD2	1:A:25:PHE:H	2	0.23
(1,718)	1:A:23:VAL:HG11	1:A:53:ILE:HG13	8	0.23
(1,718)	1:A:23:VAL:HG12	1:A:53:ILE:HG13	8	0.23
(1,718)	1:A:23:VAL:HG13	1:A:53:ILE:HG13	8	0.23
(1,688)	1:A:22:ILE:H	1:A:50:MET:HA	3	0.23
(1,668)	1:A:22:ILE:HG21	1:A:52:PHE:HA	10	0.23
(1,668)	1:A:22:ILE:HG22	1:A:52:PHE:HA	10	0.23
(1,668)	1:A:22:ILE:HG23	1:A:52:PHE:HA	10	0.23
(1,503)	1:A:18:ASN:HD21	1:A:21:VAL:HG21	5	0.23

Continued on next page...

Continued from previous page...

Key	Atom-1	Atom-2	Model ID	Violation (Å)
(1,503)	1:A:18:ASN:HD21	1:A:21:VAL:HG22	5	0.23
(1,503)	1:A:18:ASN:HD21	1:A:21:VAL:HG23	5	0.23
(1,425)	1:A:15:ILE:HG21	1:A:78:TYR:HB2	2	0.23
(1,425)	1:A:15:ILE:HG22	1:A:78:TYR:HB2	2	0.23
(1,425)	1:A:15:ILE:HG23	1:A:78:TYR:HB2	2	0.23
(1,380)	1:A:15:ILE:HD11	1:A:23:VAL:HB	1	0.23
(1,380)	1:A:15:ILE:HD12	1:A:23:VAL:HB	1	0.23
(1,380)	1:A:15:ILE:HD13	1:A:23:VAL:HB	1	0.23
(1,380)	1:A:15:ILE:HD11	1:A:23:VAL:HB	9	0.23
(1,380)	1:A:15:ILE:HD12	1:A:23:VAL:HB	9	0.23
(1,380)	1:A:15:ILE:HD13	1:A:23:VAL:HB	9	0.23
(1,2304)	1:A:95:LEU:HD11	1:A:95:LEU:HB2	6	0.23
(1,2304)	1:A:95:LEU:HD12	1:A:95:LEU:HB2	6	0.23
(1,2304)	1:A:95:LEU:HD13	1:A:95:LEU:HB2	6	0.23
(1,2303)	1:A:95:LEU:HD11	1:A:95:LEU:HB2	6	0.23
(1,2303)	1:A:95:LEU:HD12	1:A:95:LEU:HB2	6	0.23
(1,2303)	1:A:95:LEU:HD13	1:A:95:LEU:HB2	6	0.23
(1,228)	1:A:11:PHE:HE1	1:A:23:VAL:HG21	6	0.23
(1,228)	1:A:11:PHE:HE1	1:A:23:VAL:HG22	6	0.23
(1,228)	1:A:11:PHE:HE1	1:A:23:VAL:HG23	6	0.23
(1,228)	1:A:11:PHE:HE2	1:A:23:VAL:HG21	6	0.23
(1,228)	1:A:11:PHE:HE2	1:A:23:VAL:HG22	6	0.23
(1,228)	1:A:11:PHE:HE2	1:A:23:VAL:HG23	6	0.23
(1,227)	1:A:11:PHE:HE1	1:A:23:VAL:HG11	4	0.23
(1,227)	1:A:11:PHE:HE1	1:A:23:VAL:HG12	4	0.23
(1,227)	1:A:11:PHE:HE1	1:A:23:VAL:HG13	4	0.23
(1,227)	1:A:11:PHE:HE2	1:A:23:VAL:HG11	4	0.23
(1,227)	1:A:11:PHE:HE2	1:A:23:VAL:HG12	4	0.23
(1,227)	1:A:11:PHE:HE2	1:A:23:VAL:HG13	4	0.23
(1,2200)	1:A:88:LEU:HD21	1:A:88:LEU:HA	9	0.23
(1,2200)	1:A:88:LEU:HD22	1:A:88:LEU:HA	9	0.23
(1,2200)	1:A:88:LEU:HD23	1:A:88:LEU:HA	9	0.23
(1,144)	1:A:8:GLN:HE21	1:A:11:PHE:HD1	3	0.23
(1,144)	1:A:8:GLN:HE21	1:A:11:PHE:HD2	3	0.23
(1,1413)	1:A:48:THR:H	1:A:48:THR:HG21	9	0.23
(1,1413)	1:A:48:THR:H	1:A:48:THR:HG22	9	0.23
(1,1413)	1:A:48:THR:H	1:A:48:THR:HG23	9	0.23
(2,411)	1:A:33:CYS:H	1:A:36:ILE:H	4	0.22
(2,411)	1:A:33:CYS:H	1:A:36:ILE:H	9	0.22
(2,268)	1:A:51:VAL:H	1:A:52:PHE:HE1	2	0.22
(2,268)	1:A:51:VAL:H	1:A:52:PHE:HE2	2	0.22
(2,264)	1:A:50:MET:HE1	1:A:99:ILE:HA	9	0.22

Continued on next page...

Continued from previous page...

Key	Atom-1	Atom-2	Model ID	Violation (Å)
(2,264)	1:A:50:MET:HE2	1:A:99:ILE:HA	9	0.22
(2,264)	1:A:50:MET:HE3	1:A:99:ILE:HA	9	0.22
(2,151)	1:A:27:ALA:HB1	1:A:58:ASP:HB2	4	0.22
(2,151)	1:A:27:ALA:HB2	1:A:58:ASP:HB2	4	0.22
(2,151)	1:A:27:ALA:HB3	1:A:58:ASP:HB2	4	0.22
(1,96)	1:A:5:VAL:H	1:A:5:VAL:HB	10	0.22
(1,936)	1:A:27:ALA:H	1:A:27:ALA:HB1	1	0.22
(1,936)	1:A:27:ALA:H	1:A:27:ALA:HB2	1	0.22
(1,936)	1:A:27:ALA:H	1:A:27:ALA:HB3	1	0.22
(1,936)	1:A:27:ALA:H	1:A:27:ALA:HB1	2	0.22
(1,936)	1:A:27:ALA:H	1:A:27:ALA:HB2	2	0.22
(1,936)	1:A:27:ALA:H	1:A:27:ALA:HB3	2	0.22
(1,913)	1:A:26:PHE:H	1:A:26:PHE:HD1	3	0.22
(1,913)	1:A:26:PHE:H	1:A:26:PHE:HD2	3	0.22
(1,913)	1:A:26:PHE:H	1:A:26:PHE:HD1	5	0.22
(1,913)	1:A:26:PHE:H	1:A:26:PHE:HD2	5	0.22
(1,913)	1:A:26:PHE:H	1:A:26:PHE:HD1	9	0.22
(1,913)	1:A:26:PHE:H	1:A:26:PHE:HD2	9	0.22
(1,594)	1:A:20:LEU:H	1:A:50:MET:HA	3	0.22
(1,587)	1:A:20:LEU:HG	1:A:77:VAL:HG11	7	0.22
(1,587)	1:A:20:LEU:HG	1:A:77:VAL:HG12	7	0.22
(1,587)	1:A:20:LEU:HG	1:A:77:VAL:HG13	7	0.22
(1,576)	1:A:20:LEU:HD11	1:A:78:TYR:H	8	0.22
(1,576)	1:A:20:LEU:HD12	1:A:78:TYR:H	8	0.22
(1,576)	1:A:20:LEU:HD13	1:A:78:TYR:H	8	0.22
(1,433)	1:A:15:ILE:HG21	1:A:82:SER:HA	8	0.22
(1,433)	1:A:15:ILE:HG22	1:A:82:SER:HA	8	0.22
(1,433)	1:A:15:ILE:HG23	1:A:82:SER:HA	8	0.22
(1,252)	1:A:11:PHE:HZ	1:A:67:GLU:HG2	3	0.22
(1,252)	1:A:11:PHE:HZ	1:A:67:GLU:HG3	3	0.22
(1,2409)	1:A:99:ILE:HD11	1:A:99:ILE:HA	2	0.22
(1,2409)	1:A:99:ILE:HD12	1:A:99:ILE:HA	2	0.22
(1,2409)	1:A:99:ILE:HD13	1:A:99:ILE:HA	2	0.22
(1,2409)	1:A:99:ILE:HD11	1:A:99:ILE:HA	5	0.22
(1,2409)	1:A:99:ILE:HD12	1:A:99:ILE:HA	5	0.22
(1,2409)	1:A:99:ILE:HD13	1:A:99:ILE:HA	5	0.22
(1,2388)	1:A:98:LEU:HD21	1:A:99:ILE:HA	6	0.22
(1,2388)	1:A:98:LEU:HD22	1:A:99:ILE:HA	6	0.22
(1,2388)	1:A:98:LEU:HD23	1:A:99:ILE:HA	6	0.22
(1,2383)	1:A:98:LEU:HD11	1:A:102:TYR:HE1	4	0.22
(1,2383)	1:A:98:LEU:HD11	1:A:102:TYR:HE2	4	0.22
(1,2383)	1:A:98:LEU:HD12	1:A:102:TYR:HE1	4	0.22

Continued on next page...

Continued from previous page...

Key	Atom-1	Atom-2	Model ID	Violation (Å)
(1,2383)	1:A:98:LEU:HD12	1:A:102:TYR:HE2	4	0.22
(1,2383)	1:A:98:LEU:HD13	1:A:102:TYR:HE1	4	0.22
(1,2383)	1:A:98:LEU:HD13	1:A:102:TYR:HE2	4	0.22
(1,227)	1:A:11:PHE:HE1	1:A:23:VAL:HG11	2	0.22
(1,227)	1:A:11:PHE:HE1	1:A:23:VAL:HG12	2	0.22
(1,227)	1:A:11:PHE:HE1	1:A:23:VAL:HG13	2	0.22
(1,227)	1:A:11:PHE:HE2	1:A:23:VAL:HG11	2	0.22
(1,227)	1:A:11:PHE:HE2	1:A:23:VAL:HG12	2	0.22
(1,227)	1:A:11:PHE:HE2	1:A:23:VAL:HG13	2	0.22
(1,2135)	1:A:85:ASP:HA	1:A:86:THR:HG21	8	0.22
(1,2135)	1:A:85:ASP:HA	1:A:86:THR:HG22	8	0.22
(1,2135)	1:A:85:ASP:HA	1:A:86:THR:HG23	8	0.22
(1,1816)	1:A:74:THR:HG21	1:A:74:THR:HA	5	0.22
(1,1816)	1:A:74:THR:HG22	1:A:74:THR:HA	5	0.22
(1,1816)	1:A:74:THR:HG23	1:A:74:THR:HA	5	0.22
(1,1816)	1:A:74:THR:HG21	1:A:74:THR:HA	8	0.22
(1,1816)	1:A:74:THR:HG22	1:A:74:THR:HA	8	0.22
(1,1816)	1:A:74:THR:HG23	1:A:74:THR:HA	8	0.22
(1,1812)	1:A:74:THR:HG1	1:A:74:THR:HG21	7	0.22
(1,1812)	1:A:74:THR:HG1	1:A:74:THR:HG22	7	0.22
(1,1812)	1:A:74:THR:HG1	1:A:74:THR:HG23	7	0.22
(1,1550)	1:A:57:VAL:HG21	1:A:58:ASP:H	5	0.22
(1,1550)	1:A:57:VAL:HG22	1:A:58:ASP:H	5	0.22
(1,1550)	1:A:57:VAL:HG23	1:A:58:ASP:H	5	0.22
(1,1421)	1:A:49:LYS:H	1:A:49:LYS:HD2	7	0.22
(1,1421)	1:A:49:LYS:H	1:A:49:LYS:HD3	7	0.22
(1,1413)	1:A:48:THR:H	1:A:48:THR:HG21	1	0.22
(1,1413)	1:A:48:THR:H	1:A:48:THR:HG22	1	0.22
(1,1413)	1:A:48:THR:H	1:A:48:THR:HG23	1	0.22
(1,112)	1:A:6:THR:H	1:A:6:THR:HG21	7	0.22
(1,112)	1:A:6:THR:H	1:A:6:THR:HG22	7	0.22
(1,112)	1:A:6:THR:H	1:A:6:THR:HG23	7	0.22
(1,1090)	1:A:36:ILE:HG21	1:A:90:ALA:HB1	2	0.22
(1,1090)	1:A:36:ILE:HG21	1:A:90:ALA:HB2	2	0.22
(1,1090)	1:A:36:ILE:HG21	1:A:90:ALA:HB3	2	0.22
(1,1090)	1:A:36:ILE:HG22	1:A:90:ALA:HB1	2	0.22
(1,1090)	1:A:36:ILE:HG22	1:A:90:ALA:HB2	2	0.22
(1,1090)	1:A:36:ILE:HG22	1:A:90:ALA:HB3	2	0.22
(1,1090)	1:A:36:ILE:HG23	1:A:90:ALA:HB1	2	0.22
(1,1090)	1:A:36:ILE:HG23	1:A:90:ALA:HB2	2	0.22
(1,1090)	1:A:36:ILE:HG23	1:A:90:ALA:HB3	2	0.22
(2,327)	1:A:69:ILE:HG21	1:A:72:MET:HB3	1	0.21

Continued on next page...

Continued from previous page...

Key	Atom-1	Atom-2	Model ID	Violation (Å)
(2,327)	1:A:69:ILE:HG22	1:A:72:MET:HB3	1	0.21
(2,327)	1:A:69:ILE:HG23	1:A:72:MET:HB3	1	0.21
(2,327)	1:A:69:ILE:HG21	1:A:72:MET:HB3	5	0.21
(2,327)	1:A:69:ILE:HG22	1:A:72:MET:HB3	5	0.21
(2,327)	1:A:69:ILE:HG23	1:A:72:MET:HB3	5	0.21
(2,118)	1:A:25:PHE:HD1	1:A:67:GLU:HG2	2	0.21
(2,118)	1:A:25:PHE:HD1	1:A:67:GLU:HG3	2	0.21
(2,118)	1:A:25:PHE:HD2	1:A:67:GLU:HG2	2	0.21
(2,118)	1:A:25:PHE:HD2	1:A:67:GLU:HG3	2	0.21
(1,979)	1:A:32:PRO:HB3	1:A:36:ILE:HA	2	0.21
(1,936)	1:A:27:ALA:H	1:A:27:ALA:HB1	6	0.21
(1,936)	1:A:27:ALA:H	1:A:27:ALA:HB2	6	0.21
(1,936)	1:A:27:ALA:H	1:A:27:ALA:HB3	6	0.21
(1,936)	1:A:27:ALA:H	1:A:27:ALA:HB1	9	0.21
(1,936)	1:A:27:ALA:H	1:A:27:ALA:HB2	9	0.21
(1,936)	1:A:27:ALA:H	1:A:27:ALA:HB3	9	0.21
(1,913)	1:A:26:PHE:H	1:A:26:PHE:HD1	2	0.21
(1,913)	1:A:26:PHE:H	1:A:26:PHE:HD2	2	0.21
(1,838)	1:A:25:PHE:HE1	1:A:63:VAL:HG11	2	0.21
(1,838)	1:A:25:PHE:HE1	1:A:63:VAL:HG12	2	0.21
(1,838)	1:A:25:PHE:HE1	1:A:63:VAL:HG13	2	0.21
(1,838)	1:A:25:PHE:HE2	1:A:63:VAL:HG11	2	0.21
(1,838)	1:A:25:PHE:HE2	1:A:63:VAL:HG12	2	0.21
(1,838)	1:A:25:PHE:HE2	1:A:63:VAL:HG13	2	0.21
(1,764)	1:A:23:VAL:H	1:A:23:VAL:HG11	6	0.21
(1,764)	1:A:23:VAL:H	1:A:23:VAL:HG12	6	0.21
(1,764)	1:A:23:VAL:H	1:A:23:VAL:HG13	6	0.21
(1,587)	1:A:20:LEU:HG	1:A:77:VAL:HG11	6	0.21
(1,587)	1:A:20:LEU:HG	1:A:77:VAL:HG12	6	0.21
(1,587)	1:A:20:LEU:HG	1:A:77:VAL:HG13	6	0.21
(1,587)	1:A:20:LEU:HG	1:A:77:VAL:HG11	8	0.21
(1,587)	1:A:20:LEU:HG	1:A:77:VAL:HG12	8	0.21
(1,587)	1:A:20:LEU:HG	1:A:77:VAL:HG13	8	0.21
(1,433)	1:A:15:ILE:HG21	1:A:82:SER:HA	3	0.21
(1,433)	1:A:15:ILE:HG22	1:A:82:SER:HA	3	0.21
(1,433)	1:A:15:ILE:HG23	1:A:82:SER:HA	3	0.21
(1,381)	1:A:15:ILE:HD11	1:A:23:VAL:HG11	9	0.21
(1,381)	1:A:15:ILE:HD11	1:A:23:VAL:HG12	9	0.21
(1,381)	1:A:15:ILE:HD11	1:A:23:VAL:HG13	9	0.21
(1,381)	1:A:15:ILE:HD12	1:A:23:VAL:HG11	9	0.21
(1,381)	1:A:15:ILE:HD12	1:A:23:VAL:HG12	9	0.21
(1,381)	1:A:15:ILE:HD12	1:A:23:VAL:HG13	9	0.21

Continued on next page...

Continued from previous page...

Key	Atom-1	Atom-2	Model ID	Violation (Å)
(1,381)	1:A:15:ILE:HD13	1:A:23:VAL:HG11	9	0.21
(1,381)	1:A:15:ILE:HD13	1:A:23:VAL:HG12	9	0.21
(1,381)	1:A:15:ILE:HD13	1:A:23:VAL:HG13	9	0.21
(1,2409)	1:A:99:ILE:HD11	1:A:99:ILE:HA	4	0.21
(1,2409)	1:A:99:ILE:HD12	1:A:99:ILE:HA	4	0.21
(1,2409)	1:A:99:ILE:HD13	1:A:99:ILE:HA	4	0.21
(1,1890)	1:A:75:PHE:HZ	1:A:90:ALA:HB1	10	0.21
(1,1890)	1:A:75:PHE:HZ	1:A:90:ALA:HB2	10	0.21
(1,1890)	1:A:75:PHE:HZ	1:A:90:ALA:HB3	10	0.21
(1,1812)	1:A:74:THR:HG1	1:A:74:THR:HG21	4	0.21
(1,1812)	1:A:74:THR:HG1	1:A:74:THR:HG22	4	0.21
(1,1812)	1:A:74:THR:HG1	1:A:74:THR:HG23	4	0.21
(1,1488)	1:A:52:PHE:HZ	1:A:99:ILE:HG12	4	0.21
(1,144)	1:A:8:GLN:HE21	1:A:11:PHE:HD1	10	0.21
(1,144)	1:A:8:GLN:HE21	1:A:11:PHE:HD2	10	0.21
(1,1413)	1:A:48:THR:H	1:A:48:THR:HG21	7	0.21
(1,1413)	1:A:48:THR:H	1:A:48:THR:HG22	7	0.21
(1,1413)	1:A:48:THR:H	1:A:48:THR:HG23	7	0.21
(2,99)	1:A:20:LEU:HA	1:A:79:LYS:HE2	5	0.2
(2,99)	1:A:20:LEU:HA	1:A:79:LYS:HE3	5	0.2
(2,98)	1:A:20:LEU:HA	1:A:79:LYS:HE2	5	0.2
(2,98)	1:A:20:LEU:HA	1:A:79:LYS:HE3	5	0.2
(2,411)	1:A:33:CYS:H	1:A:36:ILE:H	2	0.2
(2,29)	1:A:2:VAL:H	1:A:54:LYS:HE2	6	0.2
(2,29)	1:A:2:VAL:H	1:A:54:LYS:HE3	6	0.2
(2,247)	1:A:47:TYR:HB2	1:A:52:PHE:HD1	8	0.2
(2,247)	1:A:47:TYR:HB2	1:A:52:PHE:HD2	8	0.2
(2,209)	1:A:40:TYR:HD1	1:A:43:CYS:H	6	0.2
(2,209)	1:A:40:TYR:HD2	1:A:43:CYS:H	6	0.2
(1,936)	1:A:27:ALA:H	1:A:27:ALA:HB1	4	0.2
(1,936)	1:A:27:ALA:H	1:A:27:ALA:HB2	4	0.2
(1,936)	1:A:27:ALA:H	1:A:27:ALA:HB3	4	0.2
(1,697)	1:A:22:ILE:H	1:A:53:ILE:H	7	0.2
(1,425)	1:A:15:ILE:HG21	1:A:78:TYR:HB2	6	0.2
(1,425)	1:A:15:ILE:HG22	1:A:78:TYR:HB2	6	0.2
(1,425)	1:A:15:ILE:HG23	1:A:78:TYR:HB2	6	0.2
(1,412)	1:A:15:ILE:HG21	1:A:15:ILE:HD11	3	0.2
(1,412)	1:A:15:ILE:HG21	1:A:15:ILE:HD12	3	0.2
(1,412)	1:A:15:ILE:HG21	1:A:15:ILE:HD13	3	0.2
(1,412)	1:A:15:ILE:HG22	1:A:15:ILE:HD11	3	0.2
(1,412)	1:A:15:ILE:HG22	1:A:15:ILE:HD12	3	0.2
(1,412)	1:A:15:ILE:HG22	1:A:15:ILE:HD13	3	0.2

Continued on next page...

Continued from previous page...

Key	Atom-1	Atom-2	Model ID	Violation (Å)
(1,412)	1:A:15:ILE:HG23	1:A:15:ILE:HD11	3	0.2
(1,412)	1:A:15:ILE:HG23	1:A:15:ILE:HD12	3	0.2
(1,412)	1:A:15:ILE:HG23	1:A:15:ILE:HD13	3	0.2
(1,380)	1:A:15:ILE:HD11	1:A:23:VAL:HB	10	0.2
(1,380)	1:A:15:ILE:HD12	1:A:23:VAL:HB	10	0.2
(1,380)	1:A:15:ILE:HD13	1:A:23:VAL:HB	10	0.2
(1,375)	1:A:15:ILE:HD11	1:A:21:VAL:HG11	6	0.2
(1,375)	1:A:15:ILE:HD11	1:A:21:VAL:HG12	6	0.2
(1,375)	1:A:15:ILE:HD11	1:A:21:VAL:HG13	6	0.2
(1,375)	1:A:15:ILE:HD12	1:A:21:VAL:HG11	6	0.2
(1,375)	1:A:15:ILE:HD12	1:A:21:VAL:HG12	6	0.2
(1,375)	1:A:15:ILE:HD12	1:A:21:VAL:HG13	6	0.2
(1,375)	1:A:15:ILE:HD13	1:A:21:VAL:HG11	6	0.2
(1,375)	1:A:15:ILE:HD13	1:A:21:VAL:HG12	6	0.2
(1,375)	1:A:15:ILE:HD13	1:A:21:VAL:HG13	6	0.2
(1,366)	1:A:15:ILE:HD11	1:A:15:ILE:HG21	3	0.2
(1,366)	1:A:15:ILE:HD11	1:A:15:ILE:HG22	3	0.2
(1,366)	1:A:15:ILE:HD11	1:A:15:ILE:HG23	3	0.2
(1,366)	1:A:15:ILE:HD12	1:A:15:ILE:HG21	3	0.2
(1,366)	1:A:15:ILE:HD12	1:A:15:ILE:HG22	3	0.2
(1,366)	1:A:15:ILE:HD12	1:A:15:ILE:HG23	3	0.2
(1,366)	1:A:15:ILE:HD13	1:A:15:ILE:HG21	3	0.2
(1,366)	1:A:15:ILE:HD13	1:A:15:ILE:HG22	3	0.2
(1,366)	1:A:15:ILE:HD13	1:A:15:ILE:HG23	3	0.2
(1,30)	1:A:2:VAL:H	1:A:2:VAL:HG21	1	0.2
(1,30)	1:A:2:VAL:H	1:A:2:VAL:HG22	1	0.2
(1,30)	1:A:2:VAL:H	1:A:2:VAL:HG23	1	0.2
(1,2535)	1:A:103:ALA:H	1:A:104:ALA:HA	4	0.2
(1,1969)	1:A:77:VAL:HG21	1:A:85:ASP:HB3	9	0.2
(1,1969)	1:A:77:VAL:HG22	1:A:85:ASP:HB3	9	0.2
(1,1969)	1:A:77:VAL:HG23	1:A:85:ASP:HB3	9	0.2
(1,1963)	1:A:77:VAL:HG21	1:A:77:VAL:HA	1	0.2
(1,1963)	1:A:77:VAL:HG22	1:A:77:VAL:HA	1	0.2
(1,1963)	1:A:77:VAL:HG23	1:A:77:VAL:HA	1	0.2
(1,1963)	1:A:77:VAL:HG21	1:A:77:VAL:HA	4	0.2
(1,1963)	1:A:77:VAL:HG22	1:A:77:VAL:HA	4	0.2
(1,1963)	1:A:77:VAL:HG23	1:A:77:VAL:HA	4	0.2
(1,1963)	1:A:77:VAL:HG21	1:A:77:VAL:HA	7	0.2
(1,1963)	1:A:77:VAL:HG22	1:A:77:VAL:HA	7	0.2
(1,1963)	1:A:77:VAL:HG23	1:A:77:VAL:HA	7	0.2
(1,1940)	1:A:77:VAL:HG11	1:A:77:VAL:HG21	7	0.2
(1,1940)	1:A:77:VAL:HG11	1:A:77:VAL:HG22	7	0.2

Continued on next page...

Continued from previous page...

Key	Atom-1	Atom-2	Model ID	Violation (Å)
(1,1940)	1:A:77:VAL:HG11	1:A:77:VAL:HG23	7	0.2
(1,1940)	1:A:77:VAL:HG12	1:A:77:VAL:HG21	7	0.2
(1,1940)	1:A:77:VAL:HG12	1:A:77:VAL:HG22	7	0.2
(1,1940)	1:A:77:VAL:HG12	1:A:77:VAL:HG23	7	0.2
(1,1940)	1:A:77:VAL:HG13	1:A:77:VAL:HG21	7	0.2
(1,1940)	1:A:77:VAL:HG13	1:A:77:VAL:HG22	7	0.2
(1,1940)	1:A:77:VAL:HG13	1:A:77:VAL:HG23	7	0.2
(1,1550)	1:A:57:VAL:HG21	1:A:58:ASP:H	4	0.2
(1,1550)	1:A:57:VAL:HG22	1:A:58:ASP:H	4	0.2
(1,1550)	1:A:57:VAL:HG23	1:A:58:ASP:H	4	0.2
(1,1550)	1:A:57:VAL:HG21	1:A:58:ASP:H	7	0.2
(1,1550)	1:A:57:VAL:HG22	1:A:58:ASP:H	7	0.2
(1,1550)	1:A:57:VAL:HG23	1:A:58:ASP:H	7	0.2
(1,1486)	1:A:52:PHE:HZ	1:A:99:ILE:HA	8	0.2
(1,1413)	1:A:48:THR:H	1:A:48:THR:HG21	5	0.2
(1,1413)	1:A:48:THR:H	1:A:48:THR:HG22	5	0.2
(1,1413)	1:A:48:THR:H	1:A:48:THR:HG23	5	0.2
(1,1336)	1:A:46:THR:HG21	1:A:47:TYR:H	4	0.2
(1,1336)	1:A:46:THR:HG22	1:A:47:TYR:H	4	0.2
(1,1336)	1:A:46:THR:HG23	1:A:47:TYR:H	4	0.2
(1,1225)	1:A:41:GLU:HG2	1:A:42:GLU:H	6	0.2
(1,1225)	1:A:41:GLU:HG3	1:A:42:GLU:H	6	0.2
(1,1155)	1:A:39:PHE:HD1	1:A:95:LEU:HD21	10	0.2
(1,1155)	1:A:39:PHE:HD1	1:A:95:LEU:HD22	10	0.2
(1,1155)	1:A:39:PHE:HD1	1:A:95:LEU:HD23	10	0.2
(1,1155)	1:A:39:PHE:HD2	1:A:95:LEU:HD21	10	0.2
(1,1155)	1:A:39:PHE:HD2	1:A:95:LEU:HD22	10	0.2
(1,1155)	1:A:39:PHE:HD2	1:A:95:LEU:HD23	10	0.2
(2,411)	1:A:33:CYS:H	1:A:36:ILE:H	6	0.19
(2,411)	1:A:33:CYS:H	1:A:36:ILE:H	7	0.19
(2,411)	1:A:33:CYS:H	1:A:36:ILE:H	8	0.19
(2,411)	1:A:33:CYS:H	1:A:36:ILE:H	10	0.19
(2,398)	1:A:26:PHE:HD1	1:A:27:ALA:HB1	7	0.19
(2,398)	1:A:26:PHE:HD1	1:A:27:ALA:HB2	7	0.19
(2,398)	1:A:26:PHE:HD1	1:A:27:ALA:HB3	7	0.19
(2,398)	1:A:26:PHE:HD2	1:A:27:ALA:HB1	7	0.19
(2,398)	1:A:26:PHE:HD2	1:A:27:ALA:HB2	7	0.19
(2,398)	1:A:26:PHE:HD2	1:A:27:ALA:HB3	7	0.19
(2,372)	1:A:77:VAL:HA	1:A:78:TYR:HE1	5	0.19
(2,372)	1:A:77:VAL:HA	1:A:78:TYR:HE2	5	0.19
(2,295)	1:A:67:GLU:HA	1:A:78:TYR:HE1	9	0.19
(2,295)	1:A:67:GLU:HA	1:A:78:TYR:HE2	9	0.19

Continued on next page...

Continued from previous page...

Key	Atom-1	Atom-2	Model ID	Violation (Å)
(2,287)	1:A:57:VAL:HG21	1:A:61:SER:HA	1	0.19
(2,287)	1:A:57:VAL:HG22	1:A:61:SER:HA	1	0.19
(2,287)	1:A:57:VAL:HG23	1:A:61:SER:HA	1	0.19
(2,269)	1:A:52:PHE:HB3	1:A:54:LYS:HD2	9	0.19
(2,269)	1:A:52:PHE:HB3	1:A:54:LYS:HD3	9	0.19
(2,268)	1:A:51:VAL:H	1:A:52:PHE:HE1	5	0.19
(2,268)	1:A:51:VAL:H	1:A:52:PHE:HE2	5	0.19
(1,979)	1:A:32:PRO:HB3	1:A:36:ILE:HA	4	0.19
(1,932)	1:A:27:ALA:HB1	1:A:57:VAL:HG21	1	0.19
(1,932)	1:A:27:ALA:HB1	1:A:57:VAL:HG22	1	0.19
(1,932)	1:A:27:ALA:HB1	1:A:57:VAL:HG23	1	0.19
(1,932)	1:A:27:ALA:HB2	1:A:57:VAL:HG21	1	0.19
(1,932)	1:A:27:ALA:HB2	1:A:57:VAL:HG22	1	0.19
(1,932)	1:A:27:ALA:HB2	1:A:57:VAL:HG23	1	0.19
(1,932)	1:A:27:ALA:HB3	1:A:57:VAL:HG21	1	0.19
(1,932)	1:A:27:ALA:HB3	1:A:57:VAL:HG22	1	0.19
(1,932)	1:A:27:ALA:HB3	1:A:57:VAL:HG23	1	0.19
(1,820)	1:A:25:PHE:HB2	1:A:69:ILE:HD11	7	0.19
(1,820)	1:A:25:PHE:HB2	1:A:69:ILE:HD12	7	0.19
(1,820)	1:A:25:PHE:HB2	1:A:69:ILE:HD13	7	0.19
(1,788)	1:A:24:ASP:HB2	1:A:75:PHE:HE1	1	0.19
(1,788)	1:A:24:ASP:HB2	1:A:75:PHE:HE2	1	0.19
(1,764)	1:A:23:VAL:H	1:A:23:VAL:HG11	7	0.19
(1,764)	1:A:23:VAL:H	1:A:23:VAL:HG12	7	0.19
(1,764)	1:A:23:VAL:H	1:A:23:VAL:HG13	7	0.19
(1,736)	1:A:23:VAL:HG21	1:A:24:ASP:H	10	0.19
(1,736)	1:A:23:VAL:HG22	1:A:24:ASP:H	10	0.19
(1,736)	1:A:23:VAL:HG23	1:A:24:ASP:H	10	0.19
(1,594)	1:A:20:LEU:H	1:A:50:MET:HA	8	0.19
(1,460)	1:A:17:GLN:HA	1:A:17:GLN:HG2	9	0.19
(1,433)	1:A:15:ILE:HG21	1:A:82:SER:HA	1	0.19
(1,433)	1:A:15:ILE:HG22	1:A:82:SER:HA	1	0.19
(1,433)	1:A:15:ILE:HG23	1:A:82:SER:HA	1	0.19
(1,381)	1:A:15:ILE:HD11	1:A:23:VAL:HG11	4	0.19
(1,381)	1:A:15:ILE:HD11	1:A:23:VAL:HG12	4	0.19
(1,381)	1:A:15:ILE:HD11	1:A:23:VAL:HG13	4	0.19
(1,381)	1:A:15:ILE:HD12	1:A:23:VAL:HG11	4	0.19
(1,381)	1:A:15:ILE:HD12	1:A:23:VAL:HG12	4	0.19
(1,381)	1:A:15:ILE:HD12	1:A:23:VAL:HG13	4	0.19
(1,381)	1:A:15:ILE:HD13	1:A:23:VAL:HG11	4	0.19
(1,381)	1:A:15:ILE:HD13	1:A:23:VAL:HG12	4	0.19
(1,381)	1:A:15:ILE:HD13	1:A:23:VAL:HG13	4	0.19

Continued on next page...

Continued from previous page...

Key	Atom-1	Atom-2	Model ID	Violation (Å)
(1,362)	1:A:15:ILE:HD11	1:A:15:ILE:HA	3	0.19
(1,362)	1:A:15:ILE:HD12	1:A:15:ILE:HA	3	0.19
(1,362)	1:A:15:ILE:HD13	1:A:15:ILE:HA	3	0.19
(1,2197)	1:A:88:LEU:HD11	1:A:89:GLY:H	6	0.19
(1,2197)	1:A:88:LEU:HD12	1:A:89:GLY:H	6	0.19
(1,2197)	1:A:88:LEU:HD13	1:A:89:GLY:H	6	0.19
(1,2197)	1:A:88:LEU:HD21	1:A:89:GLY:H	6	0.19
(1,2197)	1:A:88:LEU:HD22	1:A:89:GLY:H	6	0.19
(1,2197)	1:A:88:LEU:HD23	1:A:89:GLY:H	6	0.19
(1,2172)	1:A:87:LEU:HD11	1:A:91:ASN:HB2	10	0.19
(1,2172)	1:A:87:LEU:HD12	1:A:91:ASN:HB2	10	0.19
(1,2172)	1:A:87:LEU:HD13	1:A:91:ASN:HB2	10	0.19
(1,2135)	1:A:85:ASP:HA	1:A:86:THR:HG21	2	0.19
(1,2135)	1:A:85:ASP:HA	1:A:86:THR:HG22	2	0.19
(1,2135)	1:A:85:ASP:HA	1:A:86:THR:HG23	2	0.19
(1,2135)	1:A:85:ASP:HA	1:A:86:THR:HG21	6	0.19
(1,2135)	1:A:85:ASP:HA	1:A:86:THR:HG22	6	0.19
(1,2135)	1:A:85:ASP:HA	1:A:86:THR:HG23	6	0.19
(1,2004)	1:A:78:TYR:HD1	1:A:15:ILE:HG13	6	0.19
(1,2004)	1:A:78:TYR:HD2	1:A:15:ILE:HG13	6	0.19
(1,1963)	1:A:77:VAL:HG21	1:A:77:VAL:HA	5	0.19
(1,1963)	1:A:77:VAL:HG22	1:A:77:VAL:HA	5	0.19
(1,1963)	1:A:77:VAL:HG23	1:A:77:VAL:HA	5	0.19
(1,1963)	1:A:77:VAL:HG21	1:A:77:VAL:HA	6	0.19
(1,1963)	1:A:77:VAL:HG22	1:A:77:VAL:HA	6	0.19
(1,1963)	1:A:77:VAL:HG23	1:A:77:VAL:HA	6	0.19
(1,1938)	1:A:77:VAL:HG11	1:A:77:VAL:HA	8	0.19
(1,1938)	1:A:77:VAL:HG12	1:A:77:VAL:HA	8	0.19
(1,1938)	1:A:77:VAL:HG13	1:A:77:VAL:HA	8	0.19
(1,1816)	1:A:74:THR:HG21	1:A:74:THR:HA	4	0.19
(1,1816)	1:A:74:THR:HG22	1:A:74:THR:HA	4	0.19
(1,1816)	1:A:74:THR:HG23	1:A:74:THR:HA	4	0.19
(1,1762)	1:A:69:ILE:HG21	1:A:74:THR:HG1	7	0.19
(1,1762)	1:A:69:ILE:HG22	1:A:74:THR:HG1	7	0.19
(1,1762)	1:A:69:ILE:HG23	1:A:74:THR:HG1	7	0.19
(1,1693)	1:A:67:GLU:HB2	1:A:69:ILE:HD11	8	0.19
(1,1693)	1:A:67:GLU:HB2	1:A:69:ILE:HD12	8	0.19
(1,1693)	1:A:67:GLU:HB2	1:A:69:ILE:HD13	8	0.19
(1,1413)	1:A:48:THR:H	1:A:48:THR:HG21	2	0.19
(1,1413)	1:A:48:THR:H	1:A:48:THR:HG22	2	0.19
(1,1413)	1:A:48:THR:H	1:A:48:THR:HG23	2	0.19
(1,1155)	1:A:39:PHE:HD1	1:A:95:LEU:HD21	6	0.19

Continued on next page...

Continued from previous page...

Key	Atom-1	Atom-2	Model ID	Violation (Å)
(1,1155)	1:A:39:PHE:HD1	1:A:95:LEU:HD22	6	0.19
(1,1155)	1:A:39:PHE:HD1	1:A:95:LEU:HD23	6	0.19
(1,1155)	1:A:39:PHE:HD2	1:A:95:LEU:HD21	6	0.19
(1,1155)	1:A:39:PHE:HD2	1:A:95:LEU:HD22	6	0.19
(1,1155)	1:A:39:PHE:HD2	1:A:95:LEU:HD23	6	0.19
(2,99)	1:A:20:LEU:HA	1:A:79:LYS:HE2	1	0.18
(2,99)	1:A:20:LEU:HA	1:A:79:LYS:HE3	1	0.18
(2,98)	1:A:20:LEU:HA	1:A:79:LYS:HE2	1	0.18
(2,98)	1:A:20:LEU:HA	1:A:79:LYS:HE3	1	0.18
(2,411)	1:A:33:CYS:H	1:A:36:ILE:H	3	0.18
(2,398)	1:A:26:PHE:HD1	1:A:27:ALA:HB1	10	0.18
(2,398)	1:A:26:PHE:HD1	1:A:27:ALA:HB2	10	0.18
(2,398)	1:A:26:PHE:HD1	1:A:27:ALA:HB3	10	0.18
(2,398)	1:A:26:PHE:HD2	1:A:27:ALA:HB1	10	0.18
(2,398)	1:A:26:PHE:HD2	1:A:27:ALA:HB2	10	0.18
(2,398)	1:A:26:PHE:HD2	1:A:27:ALA:HB3	10	0.18
(2,264)	1:A:50:MET:HE1	1:A:99:ILE:HA	1	0.18
(2,264)	1:A:50:MET:HE2	1:A:99:ILE:HA	1	0.18
(2,264)	1:A:50:MET:HE3	1:A:99:ILE:HA	1	0.18
(2,247)	1:A:47:TYR:HB2	1:A:52:PHE:HD1	6	0.18
(2,247)	1:A:47:TYR:HB2	1:A:52:PHE:HD2	6	0.18
(2,218)	1:A:43:CYS:HB3	1:A:46:THR:HG21	4	0.18
(2,218)	1:A:43:CYS:HB3	1:A:46:THR:HG22	4	0.18
(2,218)	1:A:43:CYS:HB3	1:A:46:THR:HG23	4	0.18
(2,209)	1:A:40:TYR:HD1	1:A:43:CYS:H	10	0.18
(2,209)	1:A:40:TYR:HD2	1:A:43:CYS:H	10	0.18
(1,971)	1:A:30:CYS:H	1:A:32:PRO:HG3	5	0.18
(1,96)	1:A:5:VAL:H	1:A:5:VAL:HB	5	0.18
(1,788)	1:A:24:ASP:HB2	1:A:75:PHE:HE1	4	0.18
(1,788)	1:A:24:ASP:HB2	1:A:75:PHE:HE2	4	0.18
(1,753)	1:A:23:VAL:HG21	1:A:55:VAL:H	3	0.18
(1,753)	1:A:23:VAL:HG22	1:A:55:VAL:H	3	0.18
(1,753)	1:A:23:VAL:HG23	1:A:55:VAL:H	3	0.18
(1,746)	1:A:23:VAL:HG21	1:A:53:ILE:HG13	1	0.18
(1,746)	1:A:23:VAL:HG22	1:A:53:ILE:HG13	1	0.18
(1,746)	1:A:23:VAL:HG23	1:A:53:ILE:HG13	1	0.18
(1,697)	1:A:22:ILE:H	1:A:53:ILE:H	9	0.18
(1,688)	1:A:22:ILE:H	1:A:50:MET:HA	4	0.18
(1,684)	1:A:22:ILE:H	1:A:22:ILE:HG21	6	0.18
(1,684)	1:A:22:ILE:H	1:A:22:ILE:HG22	6	0.18
(1,684)	1:A:22:ILE:H	1:A:22:ILE:HG23	6	0.18
(1,684)	1:A:22:ILE:H	1:A:22:ILE:HG21	10	0.18

Continued on next page...

Continued from previous page...

Key	Atom-1	Atom-2	Model ID	Violation (Å)
(1,684)	1:A:22:ILE:H	1:A:22:ILE:HG22	10	0.18
(1,684)	1:A:22:ILE:H	1:A:22:ILE:HG23	10	0.18
(1,665)	1:A:22:ILE:HG21	1:A:50:MET:HB3	5	0.18
(1,665)	1:A:22:ILE:HG22	1:A:50:MET:HB3	5	0.18
(1,665)	1:A:22:ILE:HG23	1:A:50:MET:HB3	5	0.18
(1,587)	1:A:20:LEU:HG	1:A:77:VAL:HG11	5	0.18
(1,587)	1:A:20:LEU:HG	1:A:77:VAL:HG12	5	0.18
(1,587)	1:A:20:LEU:HG	1:A:77:VAL:HG13	5	0.18
(1,433)	1:A:15:ILE:HG21	1:A:82:SER:HA	4	0.18
(1,433)	1:A:15:ILE:HG22	1:A:82:SER:HA	4	0.18
(1,433)	1:A:15:ILE:HG23	1:A:82:SER:HA	4	0.18
(1,433)	1:A:15:ILE:HG21	1:A:82:SER:HA	6	0.18
(1,433)	1:A:15:ILE:HG22	1:A:82:SER:HA	6	0.18
(1,433)	1:A:15:ILE:HG23	1:A:82:SER:HA	6	0.18
(1,380)	1:A:15:ILE:HD11	1:A:23:VAL:HB	6	0.18
(1,380)	1:A:15:ILE:HD12	1:A:23:VAL:HB	6	0.18
(1,380)	1:A:15:ILE:HD13	1:A:23:VAL:HB	6	0.18
(1,375)	1:A:15:ILE:HD11	1:A:21:VAL:HG11	2	0.18
(1,375)	1:A:15:ILE:HD11	1:A:21:VAL:HG12	2	0.18
(1,375)	1:A:15:ILE:HD11	1:A:21:VAL:HG13	2	0.18
(1,375)	1:A:15:ILE:HD12	1:A:21:VAL:HG11	2	0.18
(1,375)	1:A:15:ILE:HD12	1:A:21:VAL:HG12	2	0.18
(1,375)	1:A:15:ILE:HD12	1:A:21:VAL:HG13	2	0.18
(1,375)	1:A:15:ILE:HD13	1:A:21:VAL:HG11	2	0.18
(1,375)	1:A:15:ILE:HD13	1:A:21:VAL:HG12	2	0.18
(1,375)	1:A:15:ILE:HD13	1:A:21:VAL:HG13	2	0.18
(1,2535)	1:A:103:ALA:H	1:A:104:ALA:HA	7	0.18
(1,2468)	1:A:101:LYS:HB2	1:A:103:ALA:H	4	0.18
(1,2383)	1:A:98:LEU:HD11	1:A:102:TYR:HE1	1	0.18
(1,2383)	1:A:98:LEU:HD11	1:A:102:TYR:HE2	1	0.18
(1,2383)	1:A:98:LEU:HD12	1:A:102:TYR:HE1	1	0.18
(1,2383)	1:A:98:LEU:HD12	1:A:102:TYR:HE2	1	0.18
(1,2383)	1:A:98:LEU:HD13	1:A:102:TYR:HE1	1	0.18
(1,2383)	1:A:98:LEU:HD13	1:A:102:TYR:HE2	1	0.18
(1,232)	1:A:11:PHE:HE1	1:A:63:VAL:HG11	4	0.18
(1,232)	1:A:11:PHE:HE1	1:A:63:VAL:HG12	4	0.18
(1,232)	1:A:11:PHE:HE1	1:A:63:VAL:HG13	4	0.18
(1,232)	1:A:11:PHE:HE2	1:A:63:VAL:HG11	4	0.18
(1,232)	1:A:11:PHE:HE2	1:A:63:VAL:HG12	4	0.18
(1,232)	1:A:11:PHE:HE2	1:A:63:VAL:HG13	4	0.18
(1,227)	1:A:11:PHE:HE1	1:A:23:VAL:HG11	3	0.18
(1,227)	1:A:11:PHE:HE1	1:A:23:VAL:HG12	3	0.18

Continued on next page...

Continued from previous page...

Key	Atom-1	Atom-2	Model ID	Violation (Å)
(1,227)	1:A:11:PHE:HE1	1:A:23:VAL:HG13	3	0.18
(1,227)	1:A:11:PHE:HE2	1:A:23:VAL:HG11	3	0.18
(1,227)	1:A:11:PHE:HE2	1:A:23:VAL:HG12	3	0.18
(1,227)	1:A:11:PHE:HE2	1:A:23:VAL:HG13	3	0.18
(1,2200)	1:A:88:LEU:HD21	1:A:88:LEU:HA	7	0.18
(1,2200)	1:A:88:LEU:HD22	1:A:88:LEU:HA	7	0.18
(1,2200)	1:A:88:LEU:HD23	1:A:88:LEU:HA	7	0.18
(1,2135)	1:A:85:ASP:HA	1:A:86:THR:HG21	1	0.18
(1,2135)	1:A:85:ASP:HA	1:A:86:THR:HG22	1	0.18
(1,2135)	1:A:85:ASP:HA	1:A:86:THR:HG23	1	0.18
(1,1976)	1:A:77:VAL:HG21	1:A:98:LEU:HD21	2	0.18
(1,1976)	1:A:77:VAL:HG21	1:A:98:LEU:HD22	2	0.18
(1,1976)	1:A:77:VAL:HG21	1:A:98:LEU:HD23	2	0.18
(1,1976)	1:A:77:VAL:HG22	1:A:98:LEU:HD21	2	0.18
(1,1976)	1:A:77:VAL:HG22	1:A:98:LEU:HD22	2	0.18
(1,1976)	1:A:77:VAL:HG22	1:A:98:LEU:HD23	2	0.18
(1,1976)	1:A:77:VAL:HG23	1:A:98:LEU:HD21	2	0.18
(1,1976)	1:A:77:VAL:HG23	1:A:98:LEU:HD22	2	0.18
(1,1976)	1:A:77:VAL:HG23	1:A:98:LEU:HD23	2	0.18
(1,1963)	1:A:77:VAL:HG21	1:A:77:VAL:HA	9	0.18
(1,1963)	1:A:77:VAL:HG22	1:A:77:VAL:HA	9	0.18
(1,1963)	1:A:77:VAL:HG23	1:A:77:VAL:HA	9	0.18
(1,1963)	1:A:77:VAL:HG21	1:A:77:VAL:HA	10	0.18
(1,1963)	1:A:77:VAL:HG22	1:A:77:VAL:HA	10	0.18
(1,1963)	1:A:77:VAL:HG23	1:A:77:VAL:HA	10	0.18
(1,1938)	1:A:77:VAL:HG11	1:A:77:VAL:HA	2	0.18
(1,1938)	1:A:77:VAL:HG12	1:A:77:VAL:HA	2	0.18
(1,1938)	1:A:77:VAL:HG13	1:A:77:VAL:HA	2	0.18
(1,1938)	1:A:77:VAL:HG11	1:A:77:VAL:HA	5	0.18
(1,1938)	1:A:77:VAL:HG12	1:A:77:VAL:HA	5	0.18
(1,1938)	1:A:77:VAL:HG13	1:A:77:VAL:HA	5	0.18
(1,1938)	1:A:77:VAL:HG11	1:A:77:VAL:HA	6	0.18
(1,1938)	1:A:77:VAL:HG12	1:A:77:VAL:HA	6	0.18
(1,1938)	1:A:77:VAL:HG13	1:A:77:VAL:HA	6	0.18
(1,1741)	1:A:69:ILE:HD11	1:A:69:ILE:HG21	8	0.18
(1,1741)	1:A:69:ILE:HD11	1:A:69:ILE:HG22	8	0.18
(1,1741)	1:A:69:ILE:HD11	1:A:69:ILE:HG23	8	0.18
(1,1741)	1:A:69:ILE:HD12	1:A:69:ILE:HG21	8	0.18
(1,1741)	1:A:69:ILE:HD12	1:A:69:ILE:HG22	8	0.18
(1,1741)	1:A:69:ILE:HD12	1:A:69:ILE:HG23	8	0.18
(1,1741)	1:A:69:ILE:HD13	1:A:69:ILE:HG21	8	0.18
(1,1741)	1:A:69:ILE:HD13	1:A:69:ILE:HG22	8	0.18

Continued on next page...

Continued from previous page...

Key	Atom-1	Atom-2	Model ID	Violation (Å)
(1,1741)	1:A:69:ILE:HD13	1:A:69:ILE:HG23	8	0.18
(1,1423)	1:A:49:LYS:H	1:A:49:LYS:HG2	6	0.18
(1,1413)	1:A:48:THR:H	1:A:48:THR:HG21	10	0.18
(1,1413)	1:A:48:THR:H	1:A:48:THR:HG22	10	0.18
(1,1413)	1:A:48:THR:H	1:A:48:THR:HG23	10	0.18
(1,1358)	1:A:47:TYR:HB3	1:A:99:ILE:HD11	6	0.18
(1,1358)	1:A:47:TYR:HB3	1:A:99:ILE:HD12	6	0.18
(1,1358)	1:A:47:TYR:HB3	1:A:99:ILE:HD13	6	0.18
(1,1336)	1:A:46:THR:HG21	1:A:47:TYR:H	2	0.18
(1,1336)	1:A:46:THR:HG22	1:A:47:TYR:H	2	0.18
(1,1336)	1:A:46:THR:HG23	1:A:47:TYR:H	2	0.18
(1,1207)	1:A:40:TYR:HD1	1:A:95:LEU:HD21	4	0.18
(1,1207)	1:A:40:TYR:HD1	1:A:95:LEU:HD22	4	0.18
(1,1207)	1:A:40:TYR:HD1	1:A:95:LEU:HD23	4	0.18
(1,1207)	1:A:40:TYR:HD2	1:A:95:LEU:HD21	4	0.18
(1,1207)	1:A:40:TYR:HD2	1:A:95:LEU:HD22	4	0.18
(1,1207)	1:A:40:TYR:HD2	1:A:95:LEU:HD23	4	0.18
(1,1155)	1:A:39:PHE:HD1	1:A:95:LEU:HD21	4	0.18
(1,1155)	1:A:39:PHE:HD1	1:A:95:LEU:HD22	4	0.18
(1,1155)	1:A:39:PHE:HD1	1:A:95:LEU:HD23	4	0.18
(1,1155)	1:A:39:PHE:HD2	1:A:95:LEU:HD21	4	0.18
(1,1155)	1:A:39:PHE:HD2	1:A:95:LEU:HD22	4	0.18
(1,1155)	1:A:39:PHE:HD2	1:A:95:LEU:HD23	4	0.18
(1,112)	1:A:6:THR:H	1:A:6:THR:HG21	5	0.18
(1,112)	1:A:6:THR:H	1:A:6:THR:HG22	5	0.18
(1,112)	1:A:6:THR:H	1:A:6:THR:HG23	5	0.18
(2,268)	1:A:51:VAL:H	1:A:52:PHE:HE1	6	0.17
(2,268)	1:A:51:VAL:H	1:A:52:PHE:HE2	6	0.17
(2,100)	1:A:21:VAL:HA	1:A:50:MET:HB3	10	0.17
(1,764)	1:A:23:VAL:H	1:A:23:VAL:HG11	4	0.17
(1,764)	1:A:23:VAL:H	1:A:23:VAL:HG12	4	0.17
(1,764)	1:A:23:VAL:H	1:A:23:VAL:HG13	4	0.17
(1,746)	1:A:23:VAL:HG21	1:A:53:ILE:HG13	5	0.17
(1,746)	1:A:23:VAL:HG22	1:A:53:ILE:HG13	5	0.17
(1,746)	1:A:23:VAL:HG23	1:A:53:ILE:HG13	5	0.17
(1,684)	1:A:22:ILE:H	1:A:22:ILE:HG21	5	0.17
(1,684)	1:A:22:ILE:H	1:A:22:ILE:HG22	5	0.17
(1,684)	1:A:22:ILE:H	1:A:22:ILE:HG23	5	0.17
(1,612)	1:A:21:VAL:HB	1:A:78:TYR:HB2	10	0.17
(1,594)	1:A:20:LEU:H	1:A:50:MET:HA	7	0.17
(1,587)	1:A:20:LEU:HG	1:A:77:VAL:HG11	2	0.17
(1,587)	1:A:20:LEU:HG	1:A:77:VAL:HG12	2	0.17

Continued on next page...

Continued from previous page...

Key	Atom-1	Atom-2	Model ID	Violation (Å)
(1,587)	1:A:20:LEU:HG	1:A:77:VAL:HG13	2	0.17
(1,433)	1:A:15:ILE:HG21	1:A:82:SER:HA	7	0.17
(1,433)	1:A:15:ILE:HG22	1:A:82:SER:HA	7	0.17
(1,433)	1:A:15:ILE:HG23	1:A:82:SER:HA	7	0.17
(1,2547)	1:A:29:TRP:HE3	1:A:29:TRP:HA	9	0.17
(1,2506)	1:A:101:LYS:H	1:A:103:ALA:H	9	0.17
(1,2471)	1:A:101:LYS:HD2	1:A:102:TYR:HE1	7	0.17
(1,2471)	1:A:101:LYS:HD2	1:A:102:TYR:HE2	7	0.17
(1,2468)	1:A:101:LYS:HB2	1:A:103:ALA:H	5	0.17
(1,2407)	1:A:99:ILE:HA	1:A:104:ALA:H	3	0.17
(1,2407)	1:A:99:ILE:HA	1:A:104:ALA:H	8	0.17
(1,232)	1:A:11:PHE:HE1	1:A:63:VAL:HG11	5	0.17
(1,232)	1:A:11:PHE:HE1	1:A:63:VAL:HG12	5	0.17
(1,232)	1:A:11:PHE:HE1	1:A:63:VAL:HG13	5	0.17
(1,232)	1:A:11:PHE:HE2	1:A:63:VAL:HG11	5	0.17
(1,232)	1:A:11:PHE:HE2	1:A:63:VAL:HG12	5	0.17
(1,232)	1:A:11:PHE:HE2	1:A:63:VAL:HG13	5	0.17
(1,2135)	1:A:85:ASP:HA	1:A:86:THR:HG21	4	0.17
(1,2135)	1:A:85:ASP:HA	1:A:86:THR:HG22	4	0.17
(1,2135)	1:A:85:ASP:HA	1:A:86:THR:HG23	4	0.17
(1,1940)	1:A:77:VAL:HG11	1:A:77:VAL:HG21	1	0.17
(1,1940)	1:A:77:VAL:HG11	1:A:77:VAL:HG22	1	0.17
(1,1940)	1:A:77:VAL:HG11	1:A:77:VAL:HG23	1	0.17
(1,1940)	1:A:77:VAL:HG12	1:A:77:VAL:HG21	1	0.17
(1,1940)	1:A:77:VAL:HG12	1:A:77:VAL:HG22	1	0.17
(1,1940)	1:A:77:VAL:HG12	1:A:77:VAL:HG23	1	0.17
(1,1940)	1:A:77:VAL:HG13	1:A:77:VAL:HG21	1	0.17
(1,1940)	1:A:77:VAL:HG13	1:A:77:VAL:HG22	1	0.17
(1,1940)	1:A:77:VAL:HG13	1:A:77:VAL:HG23	1	0.17
(1,1630)	1:A:63:VAL:HG11	1:A:66:LYS:HE2	8	0.17
(1,1630)	1:A:63:VAL:HG11	1:A:66:LYS:HE3	8	0.17
(1,1630)	1:A:63:VAL:HG12	1:A:66:LYS:HE2	8	0.17
(1,1630)	1:A:63:VAL:HG12	1:A:66:LYS:HE3	8	0.17
(1,1630)	1:A:63:VAL:HG13	1:A:66:LYS:HE2	8	0.17
(1,1630)	1:A:63:VAL:HG13	1:A:66:LYS:HE3	8	0.17
(1,153)	1:A:8:GLN:HE22	1:A:9:SER:H	3	0.17
(1,153)	1:A:8:GLN:HE22	1:A:9:SER:H	7	0.17
(1,153)	1:A:8:GLN:HE22	1:A:9:SER:H	8	0.17
(1,1413)	1:A:48:THR:H	1:A:48:THR:HG21	6	0.17
(1,1413)	1:A:48:THR:H	1:A:48:THR:HG22	6	0.17
(1,1413)	1:A:48:THR:H	1:A:48:THR:HG23	6	0.17
(1,1155)	1:A:39:PHE:HD1	1:A:95:LEU:HD21	1	0.17

Continued on next page...

Continued from previous page...

Key	Atom-1	Atom-2	Model ID	Violation (Å)
(1,1155)	1:A:39:PHE:HD1	1:A:95:LEU:HD22	1	0.17
(1,1155)	1:A:39:PHE:HD1	1:A:95:LEU:HD23	1	0.17
(1,1155)	1:A:39:PHE:HD2	1:A:95:LEU:HD21	1	0.17
(1,1155)	1:A:39:PHE:HD2	1:A:95:LEU:HD22	1	0.17
(1,1155)	1:A:39:PHE:HD2	1:A:95:LEU:HD23	1	0.17
(1,1155)	1:A:39:PHE:HD1	1:A:95:LEU:HD21	8	0.17
(1,1155)	1:A:39:PHE:HD1	1:A:95:LEU:HD22	8	0.17
(1,1155)	1:A:39:PHE:HD1	1:A:95:LEU:HD23	8	0.17
(1,1155)	1:A:39:PHE:HD2	1:A:95:LEU:HD21	8	0.17
(1,1155)	1:A:39:PHE:HD2	1:A:95:LEU:HD22	8	0.17
(1,1155)	1:A:39:PHE:HD2	1:A:95:LEU:HD23	8	0.17
(1,1100)	1:A:36:ILE:H	1:A:38:PRO:HD2	8	0.17
(1,1100)	1:A:36:ILE:H	1:A:38:PRO:HD3	8	0.17
(1,1084)	1:A:36:ILE:HG21	1:A:40:TYR:HB2	7	0.17
(1,1084)	1:A:36:ILE:HG22	1:A:40:TYR:HB2	7	0.17
(1,1084)	1:A:36:ILE:HG23	1:A:40:TYR:HB2	7	0.17
(1,105)	1:A:5:VAL:H	1:A:55:VAL:HB	8	0.17
(2,91)	1:A:15:ILE:HA	1:A:18:ASN:H	1	0.16
(2,91)	1:A:15:ILE:HA	1:A:18:ASN:H	8	0.16
(2,411)	1:A:33:CYS:H	1:A:36:ILE:H	1	0.16
(2,34)	1:A:3:LYS:HE2	1:A:5:VAL:HB	4	0.16
(2,34)	1:A:3:LYS:HE3	1:A:5:VAL:HB	4	0.16
(2,243)	1:A:47:TYR:HA	1:A:50:MET:HB2	2	0.16
(2,243)	1:A:47:TYR:HA	1:A:50:MET:HB2	10	0.16
(2,228)	1:A:43:CYS:H	1:A:45:LYS:HE2	8	0.16
(2,228)	1:A:43:CYS:H	1:A:45:LYS:HE3	8	0.16
(2,155)	1:A:27:ALA:H	1:A:29:TRP:H	1	0.16
(2,118)	1:A:25:PHE:HD1	1:A:67:GLU:HG2	3	0.16
(2,118)	1:A:25:PHE:HD1	1:A:67:GLU:HG3	3	0.16
(2,118)	1:A:25:PHE:HD2	1:A:67:GLU:HG2	3	0.16
(2,118)	1:A:25:PHE:HD2	1:A:67:GLU:HG3	3	0.16
(2,100)	1:A:21:VAL:HA	1:A:50:MET:HB3	3	0.16
(1,979)	1:A:32:PRO:HB3	1:A:36:ILE:HA	1	0.16
(1,971)	1:A:30:CYS:H	1:A:32:PRO:HG3	2	0.16
(1,788)	1:A:24:ASP:HB2	1:A:75:PHE:HE1	5	0.16
(1,788)	1:A:24:ASP:HB2	1:A:75:PHE:HE2	5	0.16
(1,735)	1:A:23:VAL:HG21	1:A:23:VAL:HG11	4	0.16
(1,735)	1:A:23:VAL:HG21	1:A:23:VAL:HG12	4	0.16
(1,735)	1:A:23:VAL:HG21	1:A:23:VAL:HG13	4	0.16
(1,735)	1:A:23:VAL:HG22	1:A:23:VAL:HG11	4	0.16
(1,735)	1:A:23:VAL:HG22	1:A:23:VAL:HG12	4	0.16
(1,735)	1:A:23:VAL:HG22	1:A:23:VAL:HG13	4	0.16

Continued on next page...

Continued from previous page...

Key	Atom-1	Atom-2	Model ID	Violation (Å)
(1,735)	1:A:23:VAL:HG23	1:A:23:VAL:HG11	4	0.16
(1,735)	1:A:23:VAL:HG23	1:A:23:VAL:HG12	4	0.16
(1,735)	1:A:23:VAL:HG23	1:A:23:VAL:HG13	4	0.16
(1,733)	1:A:23:VAL:HG21	1:A:23:VAL:HA	7	0.16
(1,733)	1:A:23:VAL:HG22	1:A:23:VAL:HA	7	0.16
(1,733)	1:A:23:VAL:HG23	1:A:23:VAL:HA	7	0.16
(1,688)	1:A:22:ILE:H	1:A:50:MET:HA	8	0.16
(1,612)	1:A:21:VAL:HB	1:A:78:TYR:HB2	3	0.16
(1,594)	1:A:20:LEU:H	1:A:50:MET:HA	2	0.16
(1,594)	1:A:20:LEU:H	1:A:50:MET:HA	4	0.16
(1,54)	1:A:3:LYS:H	1:A:3:LYS:HG2	9	0.16
(1,503)	1:A:18:ASN:HD21	1:A:21:VAL:HG21	2	0.16
(1,503)	1:A:18:ASN:HD21	1:A:21:VAL:HG22	2	0.16
(1,503)	1:A:18:ASN:HD21	1:A:21:VAL:HG23	2	0.16
(1,503)	1:A:18:ASN:HD21	1:A:21:VAL:HG21	8	0.16
(1,503)	1:A:18:ASN:HD21	1:A:21:VAL:HG22	8	0.16
(1,503)	1:A:18:ASN:HD21	1:A:21:VAL:HG23	8	0.16
(1,400)	1:A:15:ILE:HG13	1:A:17:GLN:H	8	0.16
(1,381)	1:A:15:ILE:HD11	1:A:23:VAL:HG11	5	0.16
(1,381)	1:A:15:ILE:HD11	1:A:23:VAL:HG12	5	0.16
(1,381)	1:A:15:ILE:HD11	1:A:23:VAL:HG13	5	0.16
(1,381)	1:A:15:ILE:HD12	1:A:23:VAL:HG11	5	0.16
(1,381)	1:A:15:ILE:HD12	1:A:23:VAL:HG12	5	0.16
(1,381)	1:A:15:ILE:HD12	1:A:23:VAL:HG13	5	0.16
(1,381)	1:A:15:ILE:HD13	1:A:23:VAL:HG11	5	0.16
(1,381)	1:A:15:ILE:HD13	1:A:23:VAL:HG12	5	0.16
(1,381)	1:A:15:ILE:HD13	1:A:23:VAL:HG13	5	0.16
(1,30)	1:A:2:VAL:H	1:A:2:VAL:HG21	6	0.16
(1,30)	1:A:2:VAL:H	1:A:2:VAL:HG22	6	0.16
(1,30)	1:A:2:VAL:H	1:A:2:VAL:HG23	6	0.16
(1,2547)	1:A:29:TRP:HE3	1:A:29:TRP:HA	1	0.16
(1,2547)	1:A:29:TRP:HE3	1:A:29:TRP:HA	4	0.16
(1,2535)	1:A:103:ALA:H	1:A:104:ALA:HA	5	0.16
(1,2506)	1:A:101:LYS:H	1:A:103:ALA:H	1	0.16
(1,2396)	1:A:98:LEU:H	1:A:98:LEU:HG	2	0.16
(1,2396)	1:A:98:LEU:H	1:A:98:LEU:HG	5	0.16
(1,1988)	1:A:77:VAL:H	1:A:84:VAL:HB	10	0.16
(1,1981)	1:A:77:VAL:HG21	1:A:102:TYR:HE1	2	0.16
(1,1981)	1:A:77:VAL:HG21	1:A:102:TYR:HE2	2	0.16
(1,1981)	1:A:77:VAL:HG22	1:A:102:TYR:HE1	2	0.16
(1,1981)	1:A:77:VAL:HG22	1:A:102:TYR:HE2	2	0.16
(1,1981)	1:A:77:VAL:HG23	1:A:102:TYR:HE1	2	0.16

Continued on next page...

Continued from previous page...

Key	Atom-1	Atom-2	Model ID	Violation (Å)
(1,1981)	1:A:77:VAL:HG23	1:A:102:TYR:HE2	2	0.16
(1,1940)	1:A:77:VAL:HG11	1:A:77:VAL:HG21	8	0.16
(1,1940)	1:A:77:VAL:HG11	1:A:77:VAL:HG22	8	0.16
(1,1940)	1:A:77:VAL:HG11	1:A:77:VAL:HG23	8	0.16
(1,1940)	1:A:77:VAL:HG12	1:A:77:VAL:HG21	8	0.16
(1,1940)	1:A:77:VAL:HG12	1:A:77:VAL:HG22	8	0.16
(1,1940)	1:A:77:VAL:HG12	1:A:77:VAL:HG23	8	0.16
(1,1940)	1:A:77:VAL:HG13	1:A:77:VAL:HG21	8	0.16
(1,1940)	1:A:77:VAL:HG13	1:A:77:VAL:HG22	8	0.16
(1,1940)	1:A:77:VAL:HG13	1:A:77:VAL:HG23	8	0.16
(1,1915)	1:A:76:LYS:HD2	1:A:86:THR:HA	7	0.16
(1,1915)	1:A:76:LYS:HD3	1:A:86:THR:HA	7	0.16
(1,1703)	1:A:67:GLU:H	1:A:68:ASN:HB2	7	0.16
(1,1550)	1:A:57:VAL:HG21	1:A:58:ASP:H	6	0.16
(1,1550)	1:A:57:VAL:HG22	1:A:58:ASP:H	6	0.16
(1,1550)	1:A:57:VAL:HG23	1:A:58:ASP:H	6	0.16
(1,1505)	1:A:54:LYS:HA	1:A:54:LYS:HG3	1	0.16
(1,1505)	1:A:54:LYS:HA	1:A:54:LYS:HG3	7	0.16
(1,1267)	1:A:43:CYS:HB3	1:A:99:ILE:HG21	2	0.16
(1,1267)	1:A:43:CYS:HB3	1:A:99:ILE:HG22	2	0.16
(1,1267)	1:A:43:CYS:HB3	1:A:99:ILE:HG23	2	0.16
(1,1263)	1:A:43:CYS:HB3	1:A:47:TYR:HD1	8	0.16
(1,1263)	1:A:43:CYS:HB3	1:A:47:TYR:HD2	8	0.16
(1,1259)	1:A:43:CYS:HA	1:A:52:PHE:HZ	1	0.16
(1,1074)	1:A:36:ILE:HG12	1:A:75:PHE:HE1	4	0.16
(1,1074)	1:A:36:ILE:HG12	1:A:75:PHE:HE2	4	0.16
(1,1053)	1:A:36:ILE:HB	1:A:90:ALA:HB1	5	0.16
(1,1053)	1:A:36:ILE:HB	1:A:90:ALA:HB2	5	0.16
(1,1053)	1:A:36:ILE:HB	1:A:90:ALA:HB3	5	0.16
(2,91)	1:A:15:ILE:HA	1:A:18:ASN:H	4	0.15
(2,90)	1:A:14:ILE:H	1:A:17:GLN:H	7	0.15
(2,73)	1:A:11:PHE:HD1	1:A:12:ASP:HB3	6	0.15
(2,73)	1:A:11:PHE:HD2	1:A:12:ASP:HB3	6	0.15
(2,243)	1:A:47:TYR:HA	1:A:50:MET:HB2	5	0.15
(2,230)	1:A:44:SER:HG	1:A:45:LYS:HE2	4	0.15
(2,230)	1:A:44:SER:HG	1:A:45:LYS:HE3	4	0.15
(2,155)	1:A:27:ALA:H	1:A:29:TRP:H	9	0.15
(1,979)	1:A:32:PRO:HB3	1:A:36:ILE:HA	6	0.15
(1,979)	1:A:32:PRO:HB3	1:A:36:ILE:HA	7	0.15
(1,979)	1:A:32:PRO:HB3	1:A:36:ILE:HA	8	0.15
(1,979)	1:A:32:PRO:HB3	1:A:36:ILE:HA	9	0.15
(1,971)	1:A:30:CYS:H	1:A:32:PRO:HG3	4	0.15

Continued on next page...

Continued from previous page...

Key	Atom-1	Atom-2	Model ID	Violation (Å)
(1,943)	1:A:28:GLU:H	1:A:29:TRP:HD1	3	0.15
(1,943)	1:A:28:GLU:H	1:A:29:TRP:HD1	10	0.15
(1,932)	1:A:27:ALA:HB1	1:A:57:VAL:HG21	5	0.15
(1,932)	1:A:27:ALA:HB1	1:A:57:VAL:HG22	5	0.15
(1,932)	1:A:27:ALA:HB1	1:A:57:VAL:HG23	5	0.15
(1,932)	1:A:27:ALA:HB2	1:A:57:VAL:HG21	5	0.15
(1,932)	1:A:27:ALA:HB2	1:A:57:VAL:HG22	5	0.15
(1,932)	1:A:27:ALA:HB2	1:A:57:VAL:HG23	5	0.15
(1,932)	1:A:27:ALA:HB3	1:A:57:VAL:HG21	5	0.15
(1,932)	1:A:27:ALA:HB3	1:A:57:VAL:HG22	5	0.15
(1,932)	1:A:27:ALA:HB3	1:A:57:VAL:HG23	5	0.15
(1,783)	1:A:24:ASP:HB3	1:A:75:PHE:HE1	3	0.15
(1,783)	1:A:24:ASP:HB3	1:A:75:PHE:HE2	3	0.15
(1,764)	1:A:23:VAL:H	1:A:23:VAL:HG11	2	0.15
(1,764)	1:A:23:VAL:H	1:A:23:VAL:HG12	2	0.15
(1,764)	1:A:23:VAL:H	1:A:23:VAL:HG13	2	0.15
(1,764)	1:A:23:VAL:H	1:A:23:VAL:HG11	3	0.15
(1,764)	1:A:23:VAL:H	1:A:23:VAL:HG12	3	0.15
(1,764)	1:A:23:VAL:H	1:A:23:VAL:HG13	3	0.15
(1,735)	1:A:23:VAL:HG21	1:A:23:VAL:HG11	3	0.15
(1,735)	1:A:23:VAL:HG21	1:A:23:VAL:HG12	3	0.15
(1,735)	1:A:23:VAL:HG21	1:A:23:VAL:HG13	3	0.15
(1,735)	1:A:23:VAL:HG22	1:A:23:VAL:HG11	3	0.15
(1,735)	1:A:23:VAL:HG22	1:A:23:VAL:HG12	3	0.15
(1,735)	1:A:23:VAL:HG22	1:A:23:VAL:HG13	3	0.15
(1,735)	1:A:23:VAL:HG23	1:A:23:VAL:HG11	3	0.15
(1,735)	1:A:23:VAL:HG23	1:A:23:VAL:HG12	3	0.15
(1,735)	1:A:23:VAL:HG23	1:A:23:VAL:HG13	3	0.15
(1,697)	1:A:22:ILE:H	1:A:53:ILE:H	10	0.15
(1,688)	1:A:22:ILE:H	1:A:50:MET:HA	1	0.15
(1,684)	1:A:22:ILE:H	1:A:22:ILE:HG21	2	0.15
(1,684)	1:A:22:ILE:H	1:A:22:ILE:HG22	2	0.15
(1,684)	1:A:22:ILE:H	1:A:22:ILE:HG23	2	0.15
(1,612)	1:A:21:VAL:HB	1:A:78:TYR:HB2	2	0.15
(1,594)	1:A:20:LEU:H	1:A:50:MET:HA	1	0.15
(1,594)	1:A:20:LEU:H	1:A:50:MET:HA	5	0.15
(1,594)	1:A:20:LEU:H	1:A:50:MET:HA	6	0.15
(1,586)	1:A:20:LEU:HG	1:A:51:VAL:H	4	0.15
(1,586)	1:A:20:LEU:HG	1:A:51:VAL:H	8	0.15
(1,54)	1:A:3:LYS:H	1:A:3:LYS:HG2	3	0.15
(1,524)	1:A:19:GLU:HA	1:A:79:LYS:HA	7	0.15
(1,433)	1:A:15:ILE:HG21	1:A:82:SER:HA	5	0.15

Continued on next page...

Continued from previous page...

Key	Atom-1	Atom-2	Model ID	Violation (Å)
(1,433)	1:A:15:ILE:HG22	1:A:82:SER:HA	5	0.15
(1,433)	1:A:15:ILE:HG23	1:A:82:SER:HA	5	0.15
(1,425)	1:A:15:ILE:HG21	1:A:78:TYR:HB2	10	0.15
(1,425)	1:A:15:ILE:HG22	1:A:78:TYR:HB2	10	0.15
(1,425)	1:A:15:ILE:HG23	1:A:78:TYR:HB2	10	0.15
(1,400)	1:A:15:ILE:HG13	1:A:17:GLN:H	1	0.15
(1,400)	1:A:15:ILE:HG13	1:A:17:GLN:H	6	0.15
(1,381)	1:A:15:ILE:HD11	1:A:23:VAL:HG11	2	0.15
(1,381)	1:A:15:ILE:HD11	1:A:23:VAL:HG12	2	0.15
(1,381)	1:A:15:ILE:HD11	1:A:23:VAL:HG13	2	0.15
(1,381)	1:A:15:ILE:HD12	1:A:23:VAL:HG11	2	0.15
(1,381)	1:A:15:ILE:HD12	1:A:23:VAL:HG12	2	0.15
(1,381)	1:A:15:ILE:HD12	1:A:23:VAL:HG13	2	0.15
(1,381)	1:A:15:ILE:HD13	1:A:23:VAL:HG11	2	0.15
(1,381)	1:A:15:ILE:HD13	1:A:23:VAL:HG12	2	0.15
(1,381)	1:A:15:ILE:HD13	1:A:23:VAL:HG13	2	0.15
(1,2535)	1:A:103:ALA:H	1:A:104:ALA:HA	8	0.15
(1,2468)	1:A:101:LYS:HB2	1:A:103:ALA:H	8	0.15
(1,2416)	1:A:99:ILE:HD11	1:A:104:ALA:H	9	0.15
(1,2416)	1:A:99:ILE:HD12	1:A:104:ALA:H	9	0.15
(1,2416)	1:A:99:ILE:HD13	1:A:104:ALA:H	9	0.15
(1,2396)	1:A:98:LEU:H	1:A:98:LEU:HG	6	0.15
(1,2396)	1:A:98:LEU:H	1:A:98:LEU:HG	7	0.15
(1,2396)	1:A:98:LEU:H	1:A:98:LEU:HG	10	0.15
(1,2388)	1:A:98:LEU:HD21	1:A:99:ILE:HA	10	0.15
(1,2388)	1:A:98:LEU:HD22	1:A:99:ILE:HA	10	0.15
(1,2388)	1:A:98:LEU:HD23	1:A:99:ILE:HA	10	0.15
(1,227)	1:A:11:PHE:HE1	1:A:23:VAL:HG11	10	0.15
(1,227)	1:A:11:PHE:HE1	1:A:23:VAL:HG12	10	0.15
(1,227)	1:A:11:PHE:HE1	1:A:23:VAL:HG13	10	0.15
(1,227)	1:A:11:PHE:HE2	1:A:23:VAL:HG11	10	0.15
(1,227)	1:A:11:PHE:HE2	1:A:23:VAL:HG12	10	0.15
(1,227)	1:A:11:PHE:HE2	1:A:23:VAL:HG13	10	0.15
(1,2243)	1:A:91:ASN:HD21	1:A:91:ASN:HD22	1	0.15
(1,2243)	1:A:91:ASN:HD21	1:A:91:ASN:HD22	2	0.15
(1,2243)	1:A:91:ASN:HD21	1:A:91:ASN:HD22	3	0.15
(1,2243)	1:A:91:ASN:HD21	1:A:91:ASN:HD22	4	0.15
(1,2243)	1:A:91:ASN:HD21	1:A:91:ASN:HD22	5	0.15
(1,2243)	1:A:91:ASN:HD21	1:A:91:ASN:HD22	6	0.15
(1,2243)	1:A:91:ASN:HD21	1:A:91:ASN:HD22	7	0.15
(1,2243)	1:A:91:ASN:HD21	1:A:91:ASN:HD22	8	0.15
(1,2243)	1:A:91:ASN:HD21	1:A:91:ASN:HD22	9	0.15

Continued on next page...

Continued from previous page...

Key	Atom-1	Atom-2	Model ID	Violation (Å)
(1,2243)	1:A:91:ASN:HD21	1:A:91:ASN:HD22	10	0.15
(1,2200)	1:A:88:LEU:HD21	1:A:88:LEU:HA	1	0.15
(1,2200)	1:A:88:LEU:HD22	1:A:88:LEU:HA	1	0.15
(1,2200)	1:A:88:LEU:HD23	1:A:88:LEU:HA	1	0.15
(1,1975)	1:A:77:VAL:HG21	1:A:98:LEU:HD11	1	0.15
(1,1975)	1:A:77:VAL:HG21	1:A:98:LEU:HD12	1	0.15
(1,1975)	1:A:77:VAL:HG21	1:A:98:LEU:HD13	1	0.15
(1,1975)	1:A:77:VAL:HG22	1:A:98:LEU:HD11	1	0.15
(1,1975)	1:A:77:VAL:HG22	1:A:98:LEU:HD12	1	0.15
(1,1975)	1:A:77:VAL:HG22	1:A:98:LEU:HD13	1	0.15
(1,1975)	1:A:77:VAL:HG23	1:A:98:LEU:HD11	1	0.15
(1,1975)	1:A:77:VAL:HG23	1:A:98:LEU:HD12	1	0.15
(1,1975)	1:A:77:VAL:HG23	1:A:98:LEU:HD13	1	0.15
(1,1940)	1:A:77:VAL:HG11	1:A:77:VAL:HG21	9	0.15
(1,1940)	1:A:77:VAL:HG11	1:A:77:VAL:HG22	9	0.15
(1,1940)	1:A:77:VAL:HG11	1:A:77:VAL:HG23	9	0.15
(1,1940)	1:A:77:VAL:HG12	1:A:77:VAL:HG21	9	0.15
(1,1940)	1:A:77:VAL:HG12	1:A:77:VAL:HG22	9	0.15
(1,1940)	1:A:77:VAL:HG12	1:A:77:VAL:HG23	9	0.15
(1,1940)	1:A:77:VAL:HG13	1:A:77:VAL:HG21	9	0.15
(1,1940)	1:A:77:VAL:HG13	1:A:77:VAL:HG22	9	0.15
(1,1940)	1:A:77:VAL:HG13	1:A:77:VAL:HG23	9	0.15
(1,1940)	1:A:77:VAL:HG11	1:A:77:VAL:HG21	10	0.15
(1,1940)	1:A:77:VAL:HG11	1:A:77:VAL:HG22	10	0.15
(1,1940)	1:A:77:VAL:HG11	1:A:77:VAL:HG23	10	0.15
(1,1940)	1:A:77:VAL:HG12	1:A:77:VAL:HG21	10	0.15
(1,1940)	1:A:77:VAL:HG12	1:A:77:VAL:HG22	10	0.15
(1,1940)	1:A:77:VAL:HG12	1:A:77:VAL:HG23	10	0.15
(1,1940)	1:A:77:VAL:HG13	1:A:77:VAL:HG21	10	0.15
(1,1940)	1:A:77:VAL:HG13	1:A:77:VAL:HG22	10	0.15
(1,1940)	1:A:77:VAL:HG13	1:A:77:VAL:HG23	10	0.15
(1,1938)	1:A:77:VAL:HG11	1:A:77:VAL:HA	1	0.15
(1,1938)	1:A:77:VAL:HG12	1:A:77:VAL:HA	1	0.15
(1,1938)	1:A:77:VAL:HG13	1:A:77:VAL:HA	1	0.15
(1,1737)	1:A:69:ILE:HD11	1:A:69:ILE:HA	1	0.15
(1,1737)	1:A:69:ILE:HD12	1:A:69:ILE:HA	1	0.15
(1,1737)	1:A:69:ILE:HD13	1:A:69:ILE:HA	1	0.15
(1,1685)	1:A:66:LYS:HG2	1:A:67:GLU:H	7	0.15
(1,1685)	1:A:66:LYS:HG3	1:A:67:GLU:H	7	0.15
(1,168)	1:A:8:GLN:H	1:A:60:VAL:HG11	7	0.15
(1,168)	1:A:8:GLN:H	1:A:60:VAL:HG12	7	0.15
(1,168)	1:A:8:GLN:H	1:A:60:VAL:HG13	7	0.15

Continued on next page...

Continued from previous page...

Key	Atom-1	Atom-2	Model ID	Violation (Å)
(1,168)	1:A:8:GLN:H	1:A:60:VAL:HG11	8	0.15
(1,168)	1:A:8:GLN:H	1:A:60:VAL:HG12	8	0.15
(1,168)	1:A:8:GLN:H	1:A:60:VAL:HG13	8	0.15
(1,1505)	1:A:54:LYS:HA	1:A:54:LYS:HG3	2	0.15
(1,1486)	1:A:52:PHE:HZ	1:A:99:ILE:HA	3	0.15
(1,1486)	1:A:52:PHE:HZ	1:A:99:ILE:HA	9	0.15
(1,1437)	1:A:50:MET:HG2	1:A:103:ALA:H	7	0.15
(1,1331)	1:A:46:THR:HG21	1:A:47:TYR:HA	2	0.15
(1,1331)	1:A:46:THR:HG22	1:A:47:TYR:HA	2	0.15
(1,1331)	1:A:46:THR:HG23	1:A:47:TYR:HA	2	0.15
(1,1155)	1:A:39:PHE:HD1	1:A:95:LEU:HD21	7	0.15
(1,1155)	1:A:39:PHE:HD1	1:A:95:LEU:HD22	7	0.15
(1,1155)	1:A:39:PHE:HD1	1:A:95:LEU:HD23	7	0.15
(1,1155)	1:A:39:PHE:HD2	1:A:95:LEU:HD21	7	0.15
(1,1155)	1:A:39:PHE:HD2	1:A:95:LEU:HD22	7	0.15
(1,1155)	1:A:39:PHE:HD2	1:A:95:LEU:HD23	7	0.15
(1,106)	1:A:5:VAL:H	1:A:55:VAL:HG21	3	0.15
(1,106)	1:A:5:VAL:H	1:A:55:VAL:HG22	3	0.15
(1,106)	1:A:5:VAL:H	1:A:55:VAL:HG23	3	0.15
(1,105)	1:A:5:VAL:H	1:A:55:VAL:HB	7	0.15
(2,91)	1:A:15:ILE:HA	1:A:18:ASN:H	6	0.14
(2,91)	1:A:15:ILE:HA	1:A:18:ASN:H	7	0.14
(2,372)	1:A:77:VAL:HA	1:A:78:TYR:HE1	10	0.14
(2,372)	1:A:77:VAL:HA	1:A:78:TYR:HE2	10	0.14
(2,244)	1:A:47:TYR:HA	1:A:52:PHE:HE1	7	0.14
(2,244)	1:A:47:TYR:HA	1:A:52:PHE:HE2	7	0.14
(2,243)	1:A:47:TYR:HA	1:A:50:MET:HB2	3	0.14
(2,238)	1:A:46:THR:HG21	1:A:104:ALA:HB1	9	0.14
(2,238)	1:A:46:THR:HG21	1:A:104:ALA:HB2	9	0.14
(2,238)	1:A:46:THR:HG21	1:A:104:ALA:HB3	9	0.14
(2,238)	1:A:46:THR:HG22	1:A:104:ALA:HB1	9	0.14
(2,238)	1:A:46:THR:HG22	1:A:104:ALA:HB2	9	0.14
(2,238)	1:A:46:THR:HG22	1:A:104:ALA:HB3	9	0.14
(2,238)	1:A:46:THR:HG23	1:A:104:ALA:HB1	9	0.14
(2,238)	1:A:46:THR:HG23	1:A:104:ALA:HB2	9	0.14
(2,238)	1:A:46:THR:HG23	1:A:104:ALA:HB3	9	0.14
(2,157)	1:A:27:ALA:H	1:A:30:CYS:H	8	0.14
(2,155)	1:A:27:ALA:H	1:A:29:TRP:H	6	0.14
(1,979)	1:A:32:PRO:HB3	1:A:36:ILE:HA	3	0.14
(1,971)	1:A:30:CYS:H	1:A:32:PRO:HG3	1	0.14
(1,943)	1:A:28:GLU:H	1:A:29:TRP:HD1	2	0.14
(1,943)	1:A:28:GLU:H	1:A:29:TRP:HD1	6	0.14

Continued on next page...

Continued from previous page...

Key	Atom-1	Atom-2	Model ID	Violation (Å)
(1,943)	1:A:28:GLU:H	1:A:29:TRP:HD1	7	0.14
(1,943)	1:A:28:GLU:H	1:A:29:TRP:HD1	8	0.14
(1,937)	1:A:28:GLU:HB3	1:A:29:TRP:HD1	4	0.14
(1,735)	1:A:23:VAL:HG21	1:A:23:VAL:HG11	6	0.14
(1,735)	1:A:23:VAL:HG21	1:A:23:VAL:HG12	6	0.14
(1,735)	1:A:23:VAL:HG21	1:A:23:VAL:HG13	6	0.14
(1,735)	1:A:23:VAL:HG22	1:A:23:VAL:HG11	6	0.14
(1,735)	1:A:23:VAL:HG22	1:A:23:VAL:HG12	6	0.14
(1,735)	1:A:23:VAL:HG22	1:A:23:VAL:HG13	6	0.14
(1,735)	1:A:23:VAL:HG23	1:A:23:VAL:HG11	6	0.14
(1,735)	1:A:23:VAL:HG23	1:A:23:VAL:HG12	6	0.14
(1,735)	1:A:23:VAL:HG23	1:A:23:VAL:HG13	6	0.14
(1,697)	1:A:22:ILE:H	1:A:53:ILE:H	1	0.14
(1,697)	1:A:22:ILE:H	1:A:53:ILE:H	3	0.14
(1,697)	1:A:22:ILE:H	1:A:53:ILE:H	5	0.14
(1,665)	1:A:22:ILE:HG21	1:A:50:MET:HB3	2	0.14
(1,665)	1:A:22:ILE:HG22	1:A:50:MET:HB3	2	0.14
(1,665)	1:A:22:ILE:HG23	1:A:50:MET:HB3	2	0.14
(1,612)	1:A:21:VAL:HB	1:A:78:TYR:HB2	8	0.14
(1,594)	1:A:20:LEU:H	1:A:50:MET:HA	10	0.14
(1,524)	1:A:19:GLU:HA	1:A:79:LYS:HA	10	0.14
(1,47)	1:A:3:LYS:HG2	1:A:53:ILE:HA	5	0.14
(1,400)	1:A:15:ILE:HG13	1:A:17:GLN:H	2	0.14
(1,400)	1:A:15:ILE:HG13	1:A:17:GLN:H	3	0.14
(1,400)	1:A:15:ILE:HG13	1:A:17:GLN:H	4	0.14
(1,400)	1:A:15:ILE:HG13	1:A:17:GLN:H	5	0.14
(1,375)	1:A:15:ILE:HD11	1:A:21:VAL:HG11	5	0.14
(1,375)	1:A:15:ILE:HD11	1:A:21:VAL:HG12	5	0.14
(1,375)	1:A:15:ILE:HD11	1:A:21:VAL:HG13	5	0.14
(1,375)	1:A:15:ILE:HD12	1:A:21:VAL:HG11	5	0.14
(1,375)	1:A:15:ILE:HD12	1:A:21:VAL:HG12	5	0.14
(1,375)	1:A:15:ILE:HD12	1:A:21:VAL:HG13	5	0.14
(1,375)	1:A:15:ILE:HD13	1:A:21:VAL:HG11	5	0.14
(1,375)	1:A:15:ILE:HD13	1:A:21:VAL:HG12	5	0.14
(1,375)	1:A:15:ILE:HD13	1:A:21:VAL:HG13	5	0.14
(1,375)	1:A:15:ILE:HD11	1:A:21:VAL:HG11	10	0.14
(1,375)	1:A:15:ILE:HD11	1:A:21:VAL:HG12	10	0.14
(1,375)	1:A:15:ILE:HD11	1:A:21:VAL:HG13	10	0.14
(1,375)	1:A:15:ILE:HD12	1:A:21:VAL:HG11	10	0.14
(1,375)	1:A:15:ILE:HD12	1:A:21:VAL:HG12	10	0.14
(1,375)	1:A:15:ILE:HD12	1:A:21:VAL:HG13	10	0.14
(1,375)	1:A:15:ILE:HD13	1:A:21:VAL:HG11	10	0.14

Continued on next page...

Continued from previous page...

Key	Atom-1	Atom-2	Model ID	Violation (Å)
(1,375)	1:A:15:ILE:HD13	1:A:21:VAL:HG12	10	0.14
(1,375)	1:A:15:ILE:HD13	1:A:21:VAL:HG13	10	0.14
(1,2535)	1:A:103:ALA:H	1:A:104:ALA:HA	2	0.14
(1,2463)	1:A:101:LYS:HA	1:A:104:ALA:H	9	0.14
(1,245)	1:A:11:PHE:HZ	1:A:15:ILE:HD11	1	0.14
(1,245)	1:A:11:PHE:HZ	1:A:15:ILE:HD12	1	0.14
(1,245)	1:A:11:PHE:HZ	1:A:15:ILE:HD13	1	0.14
(1,2416)	1:A:99:ILE:HD11	1:A:104:ALA:H	7	0.14
(1,2416)	1:A:99:ILE:HD12	1:A:104:ALA:H	7	0.14
(1,2416)	1:A:99:ILE:HD13	1:A:104:ALA:H	7	0.14
(1,2407)	1:A:99:ILE:HA	1:A:104:ALA:H	4	0.14
(1,2407)	1:A:99:ILE:HA	1:A:104:ALA:H	6	0.14
(1,2396)	1:A:98:LEU:H	1:A:98:LEU:HG	8	0.14
(1,2396)	1:A:98:LEU:H	1:A:98:LEU:HG	9	0.14
(1,2351)	1:A:97:GLN:HE21	1:A:97:GLN:HB2	4	0.14
(1,2351)	1:A:97:GLN:HE21	1:A:97:GLN:HB3	4	0.14
(1,232)	1:A:11:PHE:HE1	1:A:63:VAL:HG11	10	0.14
(1,232)	1:A:11:PHE:HE1	1:A:63:VAL:HG12	10	0.14
(1,232)	1:A:11:PHE:HE1	1:A:63:VAL:HG13	10	0.14
(1,232)	1:A:11:PHE:HE2	1:A:63:VAL:HG11	10	0.14
(1,232)	1:A:11:PHE:HE2	1:A:63:VAL:HG12	10	0.14
(1,232)	1:A:11:PHE:HE2	1:A:63:VAL:HG13	10	0.14
(1,2156)	1:A:86:THR:H	1:A:86:THR:HB	5	0.14
(1,1988)	1:A:77:VAL:H	1:A:84:VAL:HB	7	0.14
(1,1963)	1:A:77:VAL:HG21	1:A:77:VAL:HA	2	0.14
(1,1963)	1:A:77:VAL:HG22	1:A:77:VAL:HA	2	0.14
(1,1963)	1:A:77:VAL:HG23	1:A:77:VAL:HA	2	0.14
(1,1940)	1:A:77:VAL:HG11	1:A:77:VAL:HG21	2	0.14
(1,1940)	1:A:77:VAL:HG11	1:A:77:VAL:HG22	2	0.14
(1,1940)	1:A:77:VAL:HG11	1:A:77:VAL:HG23	2	0.14
(1,1940)	1:A:77:VAL:HG12	1:A:77:VAL:HG21	2	0.14
(1,1940)	1:A:77:VAL:HG12	1:A:77:VAL:HG22	2	0.14
(1,1940)	1:A:77:VAL:HG12	1:A:77:VAL:HG23	2	0.14
(1,1940)	1:A:77:VAL:HG13	1:A:77:VAL:HG21	2	0.14
(1,1940)	1:A:77:VAL:HG13	1:A:77:VAL:HG22	2	0.14
(1,1940)	1:A:77:VAL:HG13	1:A:77:VAL:HG23	2	0.14
(1,1938)	1:A:77:VAL:HG11	1:A:77:VAL:HA	3	0.14
(1,1938)	1:A:77:VAL:HG12	1:A:77:VAL:HA	3	0.14
(1,1938)	1:A:77:VAL:HG13	1:A:77:VAL:HA	3	0.14
(1,1938)	1:A:77:VAL:HG11	1:A:77:VAL:HA	4	0.14
(1,1938)	1:A:77:VAL:HG12	1:A:77:VAL:HA	4	0.14
(1,1938)	1:A:77:VAL:HG13	1:A:77:VAL:HA	4	0.14

Continued on next page...

Continued from previous page...

Key	Atom-1	Atom-2	Model ID	Violation (Å)
(1,1938)	1:A:77:VAL:HG11	1:A:77:VAL:HA	9	0.14
(1,1938)	1:A:77:VAL:HG12	1:A:77:VAL:HA	9	0.14
(1,1938)	1:A:77:VAL:HG13	1:A:77:VAL:HA	9	0.14
(1,1890)	1:A:75:PHE:HZ	1:A:90:ALA:HB1	8	0.14
(1,1890)	1:A:75:PHE:HZ	1:A:90:ALA:HB2	8	0.14
(1,1890)	1:A:75:PHE:HZ	1:A:90:ALA:HB3	8	0.14
(1,1737)	1:A:69:ILE:HD11	1:A:69:ILE:HA	8	0.14
(1,1737)	1:A:69:ILE:HD12	1:A:69:ILE:HA	8	0.14
(1,1737)	1:A:69:ILE:HD13	1:A:69:ILE:HA	8	0.14
(1,1607)	1:A:62:GLU:HB3	1:A:63:VAL:H	4	0.14
(1,1607)	1:A:62:GLU:HB3	1:A:63:VAL:H	9	0.14
(1,153)	1:A:8:GLN:HE22	1:A:9:SER:H	6	0.14
(1,1505)	1:A:54:LYS:HA	1:A:54:LYS:HG3	5	0.14
(1,1488)	1:A:52:PHE:HZ	1:A:99:ILE:HG12	2	0.14
(1,1486)	1:A:52:PHE:HZ	1:A:99:ILE:HA	6	0.14
(1,1433)	1:A:50:MET:HG3	1:A:103:ALA:H	5	0.14
(1,1358)	1:A:47:TYR:HB3	1:A:99:ILE:HD11	9	0.14
(1,1358)	1:A:47:TYR:HB3	1:A:99:ILE:HD12	9	0.14
(1,1358)	1:A:47:TYR:HB3	1:A:99:ILE:HD13	9	0.14
(1,12)	1:A:2:VAL:HG11	1:A:3:LYS:H	8	0.14
(1,12)	1:A:2:VAL:HG12	1:A:3:LYS:H	8	0.14
(1,12)	1:A:2:VAL:HG13	1:A:3:LYS:H	8	0.14
(1,1090)	1:A:36:ILE:HG21	1:A:90:ALA:HB1	1	0.14
(1,1090)	1:A:36:ILE:HG21	1:A:90:ALA:HB2	1	0.14
(1,1090)	1:A:36:ILE:HG21	1:A:90:ALA:HB3	1	0.14
(1,1090)	1:A:36:ILE:HG22	1:A:90:ALA:HB1	1	0.14
(1,1090)	1:A:36:ILE:HG22	1:A:90:ALA:HB2	1	0.14
(1,1090)	1:A:36:ILE:HG22	1:A:90:ALA:HB3	1	0.14
(1,1090)	1:A:36:ILE:HG23	1:A:90:ALA:HB1	1	0.14
(1,1090)	1:A:36:ILE:HG23	1:A:90:ALA:HB2	1	0.14
(1,1090)	1:A:36:ILE:HG23	1:A:90:ALA:HB3	1	0.14
(1,1090)	1:A:36:ILE:HG21	1:A:90:ALA:HB1	4	0.14
(1,1090)	1:A:36:ILE:HG21	1:A:90:ALA:HB2	4	0.14
(1,1090)	1:A:36:ILE:HG21	1:A:90:ALA:HB3	4	0.14
(1,1090)	1:A:36:ILE:HG22	1:A:90:ALA:HB1	4	0.14
(1,1090)	1:A:36:ILE:HG22	1:A:90:ALA:HB2	4	0.14
(1,1090)	1:A:36:ILE:HG22	1:A:90:ALA:HB3	4	0.14
(1,1090)	1:A:36:ILE:HG23	1:A:90:ALA:HB1	4	0.14
(1,1090)	1:A:36:ILE:HG23	1:A:90:ALA:HB2	4	0.14
(1,1090)	1:A:36:ILE:HG23	1:A:90:ALA:HB3	4	0.14
(1,107)	1:A:5:VAL:H	1:A:55:VAL:H	10	0.14
(2,91)	1:A:15:ILE:HA	1:A:18:ASN:H	2	0.13

Continued on next page...

Continued from previous page...

Key	Atom-1	Atom-2	Model ID	Violation (Å)
(2,90)	1:A:14:ILE:H	1:A:17:GLN:H	1	0.13
(2,90)	1:A:14:ILE:H	1:A:17:GLN:H	8	0.13
(2,90)	1:A:14:ILE:H	1:A:17:GLN:H	9	0.13
(2,402)	1:A:27:ALA:HB1	1:A:30:CYS:H	1	0.13
(2,402)	1:A:27:ALA:HB2	1:A:30:CYS:H	1	0.13
(2,402)	1:A:27:ALA:HB3	1:A:30:CYS:H	1	0.13
(2,402)	1:A:27:ALA:HB1	1:A:30:CYS:H	9	0.13
(2,402)	1:A:27:ALA:HB2	1:A:30:CYS:H	9	0.13
(2,402)	1:A:27:ALA:HB3	1:A:30:CYS:H	9	0.13
(2,385)	1:A:79:LYS:HD2	1:A:81:GLY:H	6	0.13
(2,385)	1:A:79:LYS:HD3	1:A:81:GLY:H	6	0.13
(2,320)	1:A:69:ILE:HG12	1:A:70:THR:H	10	0.13
(2,296)	1:A:67:GLU:HB3	1:A:78:TYR:HE1	2	0.13
(2,296)	1:A:67:GLU:HB3	1:A:78:TYR:HE2	2	0.13
(2,243)	1:A:47:TYR:HA	1:A:50:MET:HB2	1	0.13
(2,242)	1:A:47:TYR:HA	1:A:50:MET:HB3	7	0.13
(2,230)	1:A:44:SER:HG	1:A:45:LYS:HE2	5	0.13
(2,230)	1:A:44:SER:HG	1:A:45:LYS:HE3	5	0.13
(2,230)	1:A:44:SER:HG	1:A:45:LYS:HE2	8	0.13
(2,230)	1:A:44:SER:HG	1:A:45:LYS:HE3	8	0.13
(2,157)	1:A:27:ALA:H	1:A:30:CYS:H	6	0.13
(2,157)	1:A:27:ALA:H	1:A:30:CYS:H	7	0.13
(2,155)	1:A:27:ALA:H	1:A:29:TRP:H	3	0.13
(1,979)	1:A:32:PRO:HB3	1:A:36:ILE:HA	10	0.13
(1,971)	1:A:30:CYS:H	1:A:32:PRO:HG3	9	0.13
(1,943)	1:A:28:GLU:H	1:A:29:TRP:HD1	5	0.13
(1,933)	1:A:27:ALA:HB1	1:A:57:VAL:H	8	0.13
(1,933)	1:A:27:ALA:HB2	1:A:57:VAL:H	8	0.13
(1,933)	1:A:27:ALA:HB3	1:A:57:VAL:H	8	0.13
(1,932)	1:A:27:ALA:HB1	1:A:57:VAL:HG21	9	0.13
(1,932)	1:A:27:ALA:HB1	1:A:57:VAL:HG22	9	0.13
(1,932)	1:A:27:ALA:HB1	1:A:57:VAL:HG23	9	0.13
(1,932)	1:A:27:ALA:HB2	1:A:57:VAL:HG21	9	0.13
(1,932)	1:A:27:ALA:HB2	1:A:57:VAL:HG22	9	0.13
(1,932)	1:A:27:ALA:HB2	1:A:57:VAL:HG23	9	0.13
(1,932)	1:A:27:ALA:HB3	1:A:57:VAL:HG21	9	0.13
(1,932)	1:A:27:ALA:HB3	1:A:57:VAL:HG22	9	0.13
(1,932)	1:A:27:ALA:HB3	1:A:57:VAL:HG23	9	0.13
(1,915)	1:A:26:PHE:H	1:A:27:ALA:H	4	0.13
(1,838)	1:A:25:PHE:HE1	1:A:63:VAL:HG11	1	0.13
(1,838)	1:A:25:PHE:HE1	1:A:63:VAL:HG12	1	0.13
(1,838)	1:A:25:PHE:HE1	1:A:63:VAL:HG13	1	0.13

Continued on next page...

Continued from previous page...

Key	Atom-1	Atom-2	Model ID	Violation (Å)
(1,838)	1:A:25:PHE:HE2	1:A:63:VAL:HG11	1	0.13
(1,838)	1:A:25:PHE:HE2	1:A:63:VAL:HG12	1	0.13
(1,838)	1:A:25:PHE:HE2	1:A:63:VAL:HG13	1	0.13
(1,788)	1:A:24:ASP:HB2	1:A:75:PHE:HE1	8	0.13
(1,788)	1:A:24:ASP:HB2	1:A:75:PHE:HE2	8	0.13
(1,735)	1:A:23:VAL:HG21	1:A:23:VAL:HG11	5	0.13
(1,735)	1:A:23:VAL:HG21	1:A:23:VAL:HG12	5	0.13
(1,735)	1:A:23:VAL:HG21	1:A:23:VAL:HG13	5	0.13
(1,735)	1:A:23:VAL:HG22	1:A:23:VAL:HG11	5	0.13
(1,735)	1:A:23:VAL:HG22	1:A:23:VAL:HG12	5	0.13
(1,735)	1:A:23:VAL:HG22	1:A:23:VAL:HG13	5	0.13
(1,735)	1:A:23:VAL:HG23	1:A:23:VAL:HG11	5	0.13
(1,735)	1:A:23:VAL:HG23	1:A:23:VAL:HG12	5	0.13
(1,735)	1:A:23:VAL:HG23	1:A:23:VAL:HG13	5	0.13
(1,697)	1:A:22:ILE:H	1:A:53:ILE:H	2	0.13
(1,697)	1:A:22:ILE:H	1:A:53:ILE:H	6	0.13
(1,648)	1:A:22:ILE:HB	1:A:23:VAL:H	7	0.13
(1,612)	1:A:21:VAL:HB	1:A:78:TYR:HB2	7	0.13
(1,524)	1:A:19:GLU:HA	1:A:79:LYS:HA	2	0.13
(1,47)	1:A:3:LYS:HG2	1:A:53:ILE:HA	7	0.13
(1,47)	1:A:3:LYS:HG2	1:A:53:ILE:HA	10	0.13
(1,460)	1:A:17:GLN:HA	1:A:17:GLN:HG2	3	0.13
(1,439)	1:A:15:ILE:H	1:A:15:ILE:HG12	7	0.13
(1,433)	1:A:15:ILE:HG21	1:A:82:SER:HA	9	0.13
(1,433)	1:A:15:ILE:HG22	1:A:82:SER:HA	9	0.13
(1,433)	1:A:15:ILE:HG23	1:A:82:SER:HA	9	0.13
(1,400)	1:A:15:ILE:HG13	1:A:17:GLN:H	7	0.13
(1,400)	1:A:15:ILE:HG13	1:A:17:GLN:H	9	0.13
(1,400)	1:A:15:ILE:HG13	1:A:17:GLN:H	10	0.13
(1,381)	1:A:15:ILE:HD11	1:A:23:VAL:HG11	1	0.13
(1,381)	1:A:15:ILE:HD11	1:A:23:VAL:HG12	1	0.13
(1,381)	1:A:15:ILE:HD11	1:A:23:VAL:HG13	1	0.13
(1,381)	1:A:15:ILE:HD12	1:A:23:VAL:HG11	1	0.13
(1,381)	1:A:15:ILE:HD12	1:A:23:VAL:HG12	1	0.13
(1,381)	1:A:15:ILE:HD12	1:A:23:VAL:HG13	1	0.13
(1,381)	1:A:15:ILE:HD13	1:A:23:VAL:HG11	1	0.13
(1,381)	1:A:15:ILE:HD13	1:A:23:VAL:HG12	1	0.13
(1,381)	1:A:15:ILE:HD13	1:A:23:VAL:HG13	1	0.13
(1,2547)	1:A:29:TRP:HE3	1:A:29:TRP:HA	3	0.13
(1,2547)	1:A:29:TRP:HE3	1:A:29:TRP:HA	6	0.13
(1,2465)	1:A:101:LYS:HB3	1:A:103:ALA:H	7	0.13
(1,2407)	1:A:99:ILE:HA	1:A:104:ALA:H	2	0.13

Continued on next page...

Continued from previous page...

Key	Atom-1	Atom-2	Model ID	Violation (Å)
(1,2407)	1:A:99:ILE:HA	1:A:104:ALA:H	10	0.13
(1,2289)	1:A:94:ALA:HA	1:A:98:LEU:H	2	0.13
(1,2289)	1:A:94:ALA:HA	1:A:98:LEU:H	5	0.13
(1,2225)	1:A:90:ALA:H	1:A:90:ALA:HB1	4	0.13
(1,2225)	1:A:90:ALA:H	1:A:90:ALA:HB2	4	0.13
(1,2225)	1:A:90:ALA:H	1:A:90:ALA:HB3	4	0.13
(1,1940)	1:A:77:VAL:HG11	1:A:77:VAL:HG21	5	0.13
(1,1940)	1:A:77:VAL:HG11	1:A:77:VAL:HG22	5	0.13
(1,1940)	1:A:77:VAL:HG11	1:A:77:VAL:HG23	5	0.13
(1,1940)	1:A:77:VAL:HG12	1:A:77:VAL:HG21	5	0.13
(1,1940)	1:A:77:VAL:HG12	1:A:77:VAL:HG22	5	0.13
(1,1940)	1:A:77:VAL:HG12	1:A:77:VAL:HG23	5	0.13
(1,1940)	1:A:77:VAL:HG13	1:A:77:VAL:HG21	5	0.13
(1,1940)	1:A:77:VAL:HG13	1:A:77:VAL:HG22	5	0.13
(1,1940)	1:A:77:VAL:HG13	1:A:77:VAL:HG23	5	0.13
(1,1940)	1:A:77:VAL:HG11	1:A:77:VAL:HG21	6	0.13
(1,1940)	1:A:77:VAL:HG11	1:A:77:VAL:HG22	6	0.13
(1,1940)	1:A:77:VAL:HG11	1:A:77:VAL:HG23	6	0.13
(1,1940)	1:A:77:VAL:HG12	1:A:77:VAL:HG21	6	0.13
(1,1940)	1:A:77:VAL:HG12	1:A:77:VAL:HG22	6	0.13
(1,1940)	1:A:77:VAL:HG12	1:A:77:VAL:HG23	6	0.13
(1,1940)	1:A:77:VAL:HG13	1:A:77:VAL:HG21	6	0.13
(1,1940)	1:A:77:VAL:HG13	1:A:77:VAL:HG22	6	0.13
(1,1940)	1:A:77:VAL:HG13	1:A:77:VAL:HG23	6	0.13
(1,1938)	1:A:77:VAL:HG11	1:A:77:VAL:HA	10	0.13
(1,1938)	1:A:77:VAL:HG12	1:A:77:VAL:HA	10	0.13
(1,1938)	1:A:77:VAL:HG13	1:A:77:VAL:HA	10	0.13
(1,1915)	1:A:76:LYS:HD2	1:A:86:THR:HA	10	0.13
(1,1915)	1:A:76:LYS:HD3	1:A:86:THR:HA	10	0.13
(1,1740)	1:A:69:ILE:HD11	1:A:69:ILE:HG12	4	0.13
(1,1740)	1:A:69:ILE:HD12	1:A:69:ILE:HG12	4	0.13
(1,1740)	1:A:69:ILE:HD13	1:A:69:ILE:HG12	4	0.13
(1,1740)	1:A:69:ILE:HD11	1:A:69:ILE:HG12	7	0.13
(1,1740)	1:A:69:ILE:HD12	1:A:69:ILE:HG12	7	0.13
(1,1740)	1:A:69:ILE:HD13	1:A:69:ILE:HG12	7	0.13
(1,1740)	1:A:69:ILE:HD11	1:A:69:ILE:HG12	8	0.13
(1,1740)	1:A:69:ILE:HD12	1:A:69:ILE:HG12	8	0.13
(1,1740)	1:A:69:ILE:HD13	1:A:69:ILE:HG12	8	0.13
(1,1739)	1:A:69:ILE:HD11	1:A:69:ILE:HG13	4	0.13
(1,1739)	1:A:69:ILE:HD12	1:A:69:ILE:HG13	4	0.13
(1,1739)	1:A:69:ILE:HD13	1:A:69:ILE:HG13	4	0.13
(1,1739)	1:A:69:ILE:HD11	1:A:69:ILE:HG13	7	0.13

Continued on next page...

Continued from previous page...

Key	Atom-1	Atom-2	Model ID	Violation (Å)
(1,1739)	1:A:69:ILE:HD12	1:A:69:ILE:HG13	7	0.13
(1,1739)	1:A:69:ILE:HD13	1:A:69:ILE:HG13	7	0.13
(1,1739)	1:A:69:ILE:HD11	1:A:69:ILE:HG13	8	0.13
(1,1739)	1:A:69:ILE:HD12	1:A:69:ILE:HG13	8	0.13
(1,1739)	1:A:69:ILE:HD13	1:A:69:ILE:HG13	8	0.13
(1,1703)	1:A:67:GLU:H	1:A:68:ASN:HB2	1	0.13
(1,1607)	1:A:62:GLU:HB3	1:A:63:VAL:H	5	0.13
(1,1488)	1:A:52:PHE:HZ	1:A:99:ILE:HG12	5	0.13
(1,1448)	1:A:51:VAL:HG11	1:A:51:VAL:HA	4	0.13
(1,1448)	1:A:51:VAL:HG12	1:A:51:VAL:HA	4	0.13
(1,1448)	1:A:51:VAL:HG13	1:A:51:VAL:HA	4	0.13
(1,1433)	1:A:50:MET:HG3	1:A:103:ALA:H	4	0.13
(1,1334)	1:A:46:THR:HG21	1:A:47:TYR:HD1	1	0.13
(1,1334)	1:A:46:THR:HG21	1:A:47:TYR:HD2	1	0.13
(1,1334)	1:A:46:THR:HG22	1:A:47:TYR:HD1	1	0.13
(1,1334)	1:A:46:THR:HG22	1:A:47:TYR:HD2	1	0.13
(1,1334)	1:A:46:THR:HG23	1:A:47:TYR:HD1	1	0.13
(1,1334)	1:A:46:THR:HG23	1:A:47:TYR:HD2	1	0.13
(1,1331)	1:A:46:THR:HG21	1:A:47:TYR:HA	4	0.13
(1,1331)	1:A:46:THR:HG22	1:A:47:TYR:HA	4	0.13
(1,1331)	1:A:46:THR:HG23	1:A:47:TYR:HA	4	0.13
(1,1259)	1:A:43:CYS:HA	1:A:52:PHE:HZ	3	0.13
(1,1259)	1:A:43:CYS:HA	1:A:52:PHE:HZ	10	0.13
(1,1218)	1:A:40:TYR:H	1:A:92:ASP:HA	4	0.13
(1,1200)	1:A:40:TYR:HB2	1:A:40:TYR:HB3	1	0.13
(1,1200)	1:A:40:TYR:HB2	1:A:40:TYR:HB3	2	0.13
(1,1200)	1:A:40:TYR:HB2	1:A:40:TYR:HB3	3	0.13
(1,1200)	1:A:40:TYR:HB2	1:A:40:TYR:HB3	4	0.13
(1,1200)	1:A:40:TYR:HB2	1:A:40:TYR:HB3	5	0.13
(1,1200)	1:A:40:TYR:HB2	1:A:40:TYR:HB3	6	0.13
(1,1200)	1:A:40:TYR:HB2	1:A:40:TYR:HB3	7	0.13
(1,1200)	1:A:40:TYR:HB2	1:A:40:TYR:HB3	8	0.13
(1,1200)	1:A:40:TYR:HB2	1:A:40:TYR:HB3	9	0.13
(1,1200)	1:A:40:TYR:HB2	1:A:40:TYR:HB3	10	0.13
(1,1133)	1:A:39:PHE:HB2	1:A:41:GLU:H	8	0.13
(1,1097)	1:A:36:ILE:H	1:A:36:ILE:HG21	8	0.13
(1,1097)	1:A:36:ILE:H	1:A:36:ILE:HG22	8	0.13
(1,1097)	1:A:36:ILE:H	1:A:36:ILE:HG23	8	0.13
(1,1090)	1:A:36:ILE:HG21	1:A:90:ALA:HB1	8	0.13
(1,1090)	1:A:36:ILE:HG21	1:A:90:ALA:HB2	8	0.13
(1,1090)	1:A:36:ILE:HG21	1:A:90:ALA:HB3	8	0.13
(1,1090)	1:A:36:ILE:HG22	1:A:90:ALA:HB1	8	0.13

Continued on next page...

Continued from previous page...

Key	Atom-1	Atom-2	Model ID	Violation (Å)
(1,1090)	1:A:36:ILE:HG22	1:A:90:ALA:HB2	8	0.13
(1,1090)	1:A:36:ILE:HG22	1:A:90:ALA:HB3	8	0.13
(1,1090)	1:A:36:ILE:HG23	1:A:90:ALA:HB1	8	0.13
(1,1090)	1:A:36:ILE:HG23	1:A:90:ALA:HB2	8	0.13
(1,1090)	1:A:36:ILE:HG23	1:A:90:ALA:HB3	8	0.13
(1,1059)	1:A:36:ILE:HD11	1:A:92:ASP:H	1	0.13
(1,1059)	1:A:36:ILE:HD12	1:A:92:ASP:H	1	0.13
(1,1059)	1:A:36:ILE:HD13	1:A:92:ASP:H	1	0.13
(1,105)	1:A:5:VAL:H	1:A:55:VAL:HB	2	0.13
(2,91)	1:A:15:ILE:HA	1:A:18:ASN:H	3	0.12
(2,91)	1:A:15:ILE:HA	1:A:18:ASN:H	5	0.12
(2,91)	1:A:15:ILE:HA	1:A:18:ASN:H	9	0.12
(2,90)	1:A:14:ILE:H	1:A:17:GLN:H	2	0.12
(2,90)	1:A:14:ILE:H	1:A:17:GLN:H	10	0.12
(2,89)	1:A:14:ILE:H	1:A:17:GLN:HB2	4	0.12
(2,89)	1:A:14:ILE:H	1:A:17:GLN:HB2	5	0.12
(2,89)	1:A:14:ILE:H	1:A:17:GLN:HB2	10	0.12
(2,47)	1:A:5:VAL:HB	1:A:11:PHE:HB2	3	0.12
(2,284)	1:A:57:VAL:HG11	1:A:61:SER:HA	7	0.12
(2,284)	1:A:57:VAL:HG12	1:A:61:SER:HA	7	0.12
(2,284)	1:A:57:VAL:HG13	1:A:61:SER:HA	7	0.12
(2,241)	1:A:47:TYR:HA	1:A:49:LYS:HB2	6	0.12
(2,19)	1:A:2:VAL:HB	1:A:3:LYS:HA	8	0.12
(2,183)	1:A:29:TRP:HE1	1:A:72:MET:HE1	5	0.12
(2,183)	1:A:29:TRP:HE1	1:A:72:MET:HE2	5	0.12
(2,183)	1:A:29:TRP:HE1	1:A:72:MET:HE3	5	0.12
(2,157)	1:A:27:ALA:H	1:A:30:CYS:H	2	0.12
(2,157)	1:A:27:ALA:H	1:A:30:CYS:H	3	0.12
(2,155)	1:A:27:ALA:H	1:A:29:TRP:H	4	0.12
(2,155)	1:A:27:ALA:H	1:A:29:TRP:H	5	0.12
(2,155)	1:A:27:ALA:H	1:A:29:TRP:H	8	0.12
(1,937)	1:A:28:GLU:HB3	1:A:29:TRP:HD1	1	0.12
(1,937)	1:A:28:GLU:HB3	1:A:29:TRP:HD1	9	0.12
(1,915)	1:A:26:PHE:H	1:A:27:ALA:H	7	0.12
(1,915)	1:A:26:PHE:H	1:A:27:ALA:H	10	0.12
(1,913)	1:A:26:PHE:H	1:A:26:PHE:HD1	1	0.12
(1,913)	1:A:26:PHE:H	1:A:26:PHE:HD2	1	0.12
(1,697)	1:A:22:ILE:H	1:A:53:ILE:H	4	0.12
(1,612)	1:A:21:VAL:HB	1:A:78:TYR:HB2	5	0.12
(1,612)	1:A:21:VAL:HB	1:A:78:TYR:HB2	9	0.12
(1,524)	1:A:19:GLU:HA	1:A:79:LYS:HA	3	0.12
(1,524)	1:A:19:GLU:HA	1:A:79:LYS:HA	9	0.12

Continued on next page...

Continued from previous page...

Key	Atom-1	Atom-2	Model ID	Violation (Å)
(1,47)	1:A:3:LYS:HG2	1:A:53:ILE:HA	1	0.12
(1,47)	1:A:3:LYS:HG2	1:A:53:ILE:HA	6	0.12
(1,433)	1:A:15:ILE:HG21	1:A:82:SER:HA	10	0.12
(1,433)	1:A:15:ILE:HG22	1:A:82:SER:HA	10	0.12
(1,433)	1:A:15:ILE:HG23	1:A:82:SER:HA	10	0.12
(1,381)	1:A:15:ILE:HD11	1:A:23:VAL:HG11	6	0.12
(1,381)	1:A:15:ILE:HD11	1:A:23:VAL:HG12	6	0.12
(1,381)	1:A:15:ILE:HD11	1:A:23:VAL:HG13	6	0.12
(1,381)	1:A:15:ILE:HD12	1:A:23:VAL:HG11	6	0.12
(1,381)	1:A:15:ILE:HD12	1:A:23:VAL:HG12	6	0.12
(1,381)	1:A:15:ILE:HD12	1:A:23:VAL:HG13	6	0.12
(1,381)	1:A:15:ILE:HD13	1:A:23:VAL:HG11	6	0.12
(1,381)	1:A:15:ILE:HD13	1:A:23:VAL:HG12	6	0.12
(1,381)	1:A:15:ILE:HD13	1:A:23:VAL:HG13	6	0.12
(1,327)	1:A:14:ILE:HB	1:A:15:ILE:HD11	3	0.12
(1,327)	1:A:14:ILE:HB	1:A:15:ILE:HD12	3	0.12
(1,327)	1:A:14:ILE:HB	1:A:15:ILE:HD13	3	0.12
(1,2547)	1:A:29:TRP:HE3	1:A:29:TRP:HA	2	0.12
(1,2547)	1:A:29:TRP:HE3	1:A:29:TRP:HA	5	0.12
(1,2547)	1:A:29:TRP:HE3	1:A:29:TRP:HA	8	0.12
(1,2547)	1:A:29:TRP:HE3	1:A:29:TRP:HA	10	0.12
(1,252)	1:A:11:PHE:HZ	1:A:67:GLU:HG2	6	0.12
(1,252)	1:A:11:PHE:HZ	1:A:67:GLU:HG3	6	0.12
(1,2506)	1:A:101:LYS:H	1:A:103:ALA:H	4	0.12
(1,2407)	1:A:99:ILE:HA	1:A:104:ALA:H	9	0.12
(1,2289)	1:A:94:ALA:HA	1:A:98:LEU:H	4	0.12
(1,2289)	1:A:94:ALA:HA	1:A:98:LEU:H	8	0.12
(1,2275)	1:A:93:SER:HB2	1:A:96:LYS:H	7	0.12
(1,2271)	1:A:93:SER:HB2	1:A:97:GLN:HE22	4	0.12
(1,2271)	1:A:93:SER:HB3	1:A:97:GLN:HE22	4	0.12
(1,227)	1:A:11:PHE:HE1	1:A:23:VAL:HG11	7	0.12
(1,227)	1:A:11:PHE:HE1	1:A:23:VAL:HG12	7	0.12
(1,227)	1:A:11:PHE:HE1	1:A:23:VAL:HG13	7	0.12
(1,227)	1:A:11:PHE:HE2	1:A:23:VAL:HG11	7	0.12
(1,227)	1:A:11:PHE:HE2	1:A:23:VAL:HG12	7	0.12
(1,227)	1:A:11:PHE:HE2	1:A:23:VAL:HG13	7	0.12
(1,2127)	1:A:84:VAL:HG21	1:A:102:TYR:HE1	2	0.12
(1,2127)	1:A:84:VAL:HG21	1:A:102:TYR:HE2	2	0.12
(1,2127)	1:A:84:VAL:HG22	1:A:102:TYR:HE1	2	0.12
(1,2127)	1:A:84:VAL:HG22	1:A:102:TYR:HE2	2	0.12
(1,2127)	1:A:84:VAL:HG23	1:A:102:TYR:HE1	2	0.12
(1,2127)	1:A:84:VAL:HG23	1:A:102:TYR:HE2	2	0.12

Continued on next page...

Continued from previous page...

Key	Atom-1	Atom-2	Model ID	Violation (Å)
(1,2018)	1:A:78:TYR:HD1	1:A:83:SER:HA	7	0.12
(1,2018)	1:A:78:TYR:HD2	1:A:83:SER:HA	7	0.12
(1,1988)	1:A:77:VAL:H	1:A:84:VAL:HB	5	0.12
(1,1963)	1:A:77:VAL:HG21	1:A:77:VAL:HA	8	0.12
(1,1963)	1:A:77:VAL:HG22	1:A:77:VAL:HA	8	0.12
(1,1963)	1:A:77:VAL:HG23	1:A:77:VAL:HA	8	0.12
(1,1940)	1:A:77:VAL:HG11	1:A:77:VAL:HG21	3	0.12
(1,1940)	1:A:77:VAL:HG11	1:A:77:VAL:HG22	3	0.12
(1,1940)	1:A:77:VAL:HG11	1:A:77:VAL:HG23	3	0.12
(1,1940)	1:A:77:VAL:HG12	1:A:77:VAL:HG21	3	0.12
(1,1940)	1:A:77:VAL:HG12	1:A:77:VAL:HG22	3	0.12
(1,1940)	1:A:77:VAL:HG12	1:A:77:VAL:HG23	3	0.12
(1,1940)	1:A:77:VAL:HG13	1:A:77:VAL:HG21	3	0.12
(1,1940)	1:A:77:VAL:HG13	1:A:77:VAL:HG22	3	0.12
(1,1940)	1:A:77:VAL:HG13	1:A:77:VAL:HG23	3	0.12
(1,1940)	1:A:77:VAL:HG11	1:A:77:VAL:HG21	4	0.12
(1,1940)	1:A:77:VAL:HG11	1:A:77:VAL:HG22	4	0.12
(1,1940)	1:A:77:VAL:HG11	1:A:77:VAL:HG23	4	0.12
(1,1940)	1:A:77:VAL:HG12	1:A:77:VAL:HG21	4	0.12
(1,1940)	1:A:77:VAL:HG12	1:A:77:VAL:HG22	4	0.12
(1,1940)	1:A:77:VAL:HG12	1:A:77:VAL:HG23	4	0.12
(1,1940)	1:A:77:VAL:HG13	1:A:77:VAL:HG21	4	0.12
(1,1940)	1:A:77:VAL:HG13	1:A:77:VAL:HG22	4	0.12
(1,1940)	1:A:77:VAL:HG13	1:A:77:VAL:HG23	4	0.12
(1,1757)	1:A:69:ILE:HG21	1:A:69:ILE:HA	10	0.12
(1,1757)	1:A:69:ILE:HG22	1:A:69:ILE:HA	10	0.12
(1,1757)	1:A:69:ILE:HG23	1:A:69:ILE:HA	10	0.12
(1,1740)	1:A:69:ILE:HD11	1:A:69:ILE:HG12	1	0.12
(1,1740)	1:A:69:ILE:HD12	1:A:69:ILE:HG12	1	0.12
(1,1740)	1:A:69:ILE:HD13	1:A:69:ILE:HG12	1	0.12
(1,1740)	1:A:69:ILE:HD11	1:A:69:ILE:HG12	2	0.12
(1,1740)	1:A:69:ILE:HD12	1:A:69:ILE:HG12	2	0.12
(1,1740)	1:A:69:ILE:HD13	1:A:69:ILE:HG12	2	0.12
(1,1739)	1:A:69:ILE:HD11	1:A:69:ILE:HG13	1	0.12
(1,1739)	1:A:69:ILE:HD12	1:A:69:ILE:HG13	1	0.12
(1,1739)	1:A:69:ILE:HD13	1:A:69:ILE:HG13	1	0.12
(1,1739)	1:A:69:ILE:HD11	1:A:69:ILE:HG13	2	0.12
(1,1739)	1:A:69:ILE:HD12	1:A:69:ILE:HG13	2	0.12
(1,1739)	1:A:69:ILE:HD13	1:A:69:ILE:HG13	2	0.12
(1,164)	1:A:8:GLN:H	1:A:8:GLN:HG2	2	0.12
(1,1625)	1:A:63:VAL:HG11	1:A:63:VAL:HB	1	0.12
(1,1625)	1:A:63:VAL:HG12	1:A:63:VAL:HB	1	0.12

Continued on next page...

Continued from previous page...

Key	Atom-1	Atom-2	Model ID	Violation (Å)
(1,1625)	1:A:63:VAL:HG13	1:A:63:VAL:HB	1	0.12
(1,1625)	1:A:63:VAL:HG11	1:A:63:VAL:HB	2	0.12
(1,1625)	1:A:63:VAL:HG12	1:A:63:VAL:HB	2	0.12
(1,1625)	1:A:63:VAL:HG13	1:A:63:VAL:HB	2	0.12
(1,1625)	1:A:63:VAL:HG11	1:A:63:VAL:HB	7	0.12
(1,1625)	1:A:63:VAL:HG12	1:A:63:VAL:HB	7	0.12
(1,1625)	1:A:63:VAL:HG13	1:A:63:VAL:HB	7	0.12
(1,1625)	1:A:63:VAL:HG11	1:A:63:VAL:HB	9	0.12
(1,1625)	1:A:63:VAL:HG12	1:A:63:VAL:HB	9	0.12
(1,1625)	1:A:63:VAL:HG13	1:A:63:VAL:HB	9	0.12
(1,1557)	1:A:57:VAL:H	1:A:58:ASP:HA	4	0.12
(1,1486)	1:A:52:PHE:HZ	1:A:99:ILE:HA	7	0.12
(1,1448)	1:A:51:VAL:HG11	1:A:51:VAL:HA	1	0.12
(1,1448)	1:A:51:VAL:HG12	1:A:51:VAL:HA	1	0.12
(1,1448)	1:A:51:VAL:HG13	1:A:51:VAL:HA	1	0.12
(1,1433)	1:A:50:MET:HG3	1:A:103:ALA:H	6	0.12
(1,1259)	1:A:43:CYS:HA	1:A:52:PHE:HZ	6	0.12
(1,1133)	1:A:39:PHE:HB2	1:A:41:GLU:H	5	0.12
(1,1097)	1:A:36:ILE:H	1:A:36:ILE:HG21	3	0.12
(1,1097)	1:A:36:ILE:H	1:A:36:ILE:HG22	3	0.12
(1,1097)	1:A:36:ILE:H	1:A:36:ILE:HG23	3	0.12
(1,1097)	1:A:36:ILE:H	1:A:36:ILE:HG21	9	0.12
(1,1097)	1:A:36:ILE:H	1:A:36:ILE:HG22	9	0.12
(1,1097)	1:A:36:ILE:H	1:A:36:ILE:HG23	9	0.12
(1,1074)	1:A:36:ILE:HG12	1:A:75:PHE:HE1	10	0.12
(1,1074)	1:A:36:ILE:HG12	1:A:75:PHE:HE2	10	0.12
(1,106)	1:A:5:VAL:H	1:A:55:VAL:HG21	10	0.12
(1,106)	1:A:5:VAL:H	1:A:55:VAL:HG22	10	0.12
(1,106)	1:A:5:VAL:H	1:A:55:VAL:HG23	10	0.12
(1,1029)	1:A:35:ARG:H	1:A:36:ILE:HB	5	0.12
(2,90)	1:A:14:ILE:H	1:A:17:GLN:H	3	0.11
(2,90)	1:A:14:ILE:H	1:A:17:GLN:H	4	0.11
(2,47)	1:A:5:VAL:HB	1:A:11:PHE:HB2	10	0.11
(2,268)	1:A:51:VAL:H	1:A:52:PHE:HE1	4	0.11
(2,268)	1:A:51:VAL:H	1:A:52:PHE:HE2	4	0.11
(2,243)	1:A:47:TYR:HA	1:A:50:MET:HB2	6	0.11
(2,19)	1:A:2:VAL:HB	1:A:3:LYS:HA	3	0.11
(2,157)	1:A:27:ALA:H	1:A:30:CYS:H	10	0.11
(2,155)	1:A:27:ALA:H	1:A:29:TRP:H	2	0.11
(2,136)	1:A:26:PHE:HB3	1:A:74:THR:H	7	0.11
(2,114)	1:A:24:ASP:H	1:A:25:PHE:HA	8	0.11
(1,979)	1:A:32:PRO:HB3	1:A:36:ILE:HA	5	0.11

Continued on next page...

Continued from previous page...

Key	Atom-1	Atom-2	Model ID	Violation (Å)
(1,932)	1:A:27:ALA:HB1	1:A:57:VAL:HG21	8	0.11
(1,932)	1:A:27:ALA:HB1	1:A:57:VAL:HG22	8	0.11
(1,932)	1:A:27:ALA:HB1	1:A:57:VAL:HG23	8	0.11
(1,932)	1:A:27:ALA:HB2	1:A:57:VAL:HG21	8	0.11
(1,932)	1:A:27:ALA:HB2	1:A:57:VAL:HG22	8	0.11
(1,932)	1:A:27:ALA:HB2	1:A:57:VAL:HG23	8	0.11
(1,932)	1:A:27:ALA:HB3	1:A:57:VAL:HG21	8	0.11
(1,932)	1:A:27:ALA:HB3	1:A:57:VAL:HG22	8	0.11
(1,932)	1:A:27:ALA:HB3	1:A:57:VAL:HG23	8	0.11
(1,915)	1:A:26:PHE:H	1:A:27:ALA:H	1	0.11
(1,915)	1:A:26:PHE:H	1:A:27:ALA:H	2	0.11
(1,915)	1:A:26:PHE:H	1:A:27:ALA:H	5	0.11
(1,915)	1:A:26:PHE:H	1:A:27:ALA:H	8	0.11
(1,915)	1:A:26:PHE:H	1:A:27:ALA:H	9	0.11
(1,913)	1:A:26:PHE:H	1:A:26:PHE:HD1	4	0.11
(1,913)	1:A:26:PHE:H	1:A:26:PHE:HD2	4	0.11
(1,749)	1:A:23:VAL:HG21	1:A:54:LYS:HA	1	0.11
(1,749)	1:A:23:VAL:HG22	1:A:54:LYS:HA	1	0.11
(1,749)	1:A:23:VAL:HG23	1:A:54:LYS:HA	1	0.11
(1,735)	1:A:23:VAL:HG21	1:A:23:VAL:HG11	1	0.11
(1,735)	1:A:23:VAL:HG21	1:A:23:VAL:HG12	1	0.11
(1,735)	1:A:23:VAL:HG21	1:A:23:VAL:HG13	1	0.11
(1,735)	1:A:23:VAL:HG22	1:A:23:VAL:HG11	1	0.11
(1,735)	1:A:23:VAL:HG22	1:A:23:VAL:HG12	1	0.11
(1,735)	1:A:23:VAL:HG22	1:A:23:VAL:HG13	1	0.11
(1,735)	1:A:23:VAL:HG23	1:A:23:VAL:HG11	1	0.11
(1,735)	1:A:23:VAL:HG23	1:A:23:VAL:HG12	1	0.11
(1,735)	1:A:23:VAL:HG23	1:A:23:VAL:HG13	1	0.11
(1,735)	1:A:23:VAL:HG21	1:A:23:VAL:HG11	2	0.11
(1,735)	1:A:23:VAL:HG21	1:A:23:VAL:HG12	2	0.11
(1,735)	1:A:23:VAL:HG21	1:A:23:VAL:HG13	2	0.11
(1,735)	1:A:23:VAL:HG22	1:A:23:VAL:HG11	2	0.11
(1,735)	1:A:23:VAL:HG22	1:A:23:VAL:HG12	2	0.11
(1,735)	1:A:23:VAL:HG22	1:A:23:VAL:HG13	2	0.11
(1,735)	1:A:23:VAL:HG23	1:A:23:VAL:HG11	2	0.11
(1,735)	1:A:23:VAL:HG23	1:A:23:VAL:HG12	2	0.11
(1,735)	1:A:23:VAL:HG23	1:A:23:VAL:HG13	2	0.11
(1,735)	1:A:23:VAL:HG21	1:A:23:VAL:HG11	9	0.11
(1,735)	1:A:23:VAL:HG21	1:A:23:VAL:HG12	9	0.11
(1,735)	1:A:23:VAL:HG21	1:A:23:VAL:HG13	9	0.11
(1,735)	1:A:23:VAL:HG22	1:A:23:VAL:HG11	9	0.11
(1,735)	1:A:23:VAL:HG22	1:A:23:VAL:HG12	9	0.11

Continued on next page...

Continued from previous page...

Key	Atom-1	Atom-2	Model ID	Violation (Å)
(1,735)	1:A:23:VAL:HG22	1:A:23:VAL:HG13	9	0.11
(1,735)	1:A:23:VAL:HG23	1:A:23:VAL:HG11	9	0.11
(1,735)	1:A:23:VAL:HG23	1:A:23:VAL:HG12	9	0.11
(1,735)	1:A:23:VAL:HG23	1:A:23:VAL:HG13	9	0.11
(1,735)	1:A:23:VAL:HG21	1:A:23:VAL:HG11	10	0.11
(1,735)	1:A:23:VAL:HG21	1:A:23:VAL:HG12	10	0.11
(1,735)	1:A:23:VAL:HG21	1:A:23:VAL:HG13	10	0.11
(1,735)	1:A:23:VAL:HG22	1:A:23:VAL:HG11	10	0.11
(1,735)	1:A:23:VAL:HG22	1:A:23:VAL:HG12	10	0.11
(1,735)	1:A:23:VAL:HG22	1:A:23:VAL:HG13	10	0.11
(1,735)	1:A:23:VAL:HG23	1:A:23:VAL:HG11	10	0.11
(1,735)	1:A:23:VAL:HG23	1:A:23:VAL:HG12	10	0.11
(1,735)	1:A:23:VAL:HG23	1:A:23:VAL:HG13	10	0.11
(1,705)	1:A:23:VAL:HG11	1:A:23:VAL:HA	8	0.11
(1,705)	1:A:23:VAL:HG12	1:A:23:VAL:HA	8	0.11
(1,705)	1:A:23:VAL:HG13	1:A:23:VAL:HA	8	0.11
(1,688)	1:A:22:ILE:H	1:A:50:MET:HA	5	0.11
(1,688)	1:A:22:ILE:H	1:A:50:MET:HA	6	0.11
(1,688)	1:A:22:ILE:H	1:A:50:MET:HA	9	0.11
(1,684)	1:A:22:ILE:H	1:A:22:ILE:HG21	3	0.11
(1,684)	1:A:22:ILE:H	1:A:22:ILE:HG22	3	0.11
(1,684)	1:A:22:ILE:H	1:A:22:ILE:HG23	3	0.11
(1,612)	1:A:21:VAL:HB	1:A:78:TYR:HB2	4	0.11
(1,611)	1:A:21:VAL:HB	1:A:78:TYR:HB3	10	0.11
(1,598)	1:A:21:VAL:HA	1:A:21:VAL:HG11	1	0.11
(1,598)	1:A:21:VAL:HA	1:A:21:VAL:HG12	1	0.11
(1,598)	1:A:21:VAL:HA	1:A:21:VAL:HG13	1	0.11
(1,598)	1:A:21:VAL:HA	1:A:21:VAL:HG11	10	0.11
(1,598)	1:A:21:VAL:HA	1:A:21:VAL:HG12	10	0.11
(1,598)	1:A:21:VAL:HA	1:A:21:VAL:HG13	10	0.11
(1,594)	1:A:20:LEU:H	1:A:50:MET:HA	9	0.11
(1,586)	1:A:20:LEU:HG	1:A:51:VAL:H	1	0.11
(1,524)	1:A:19:GLU:HA	1:A:79:LYS:HA	1	0.11
(1,524)	1:A:19:GLU:HA	1:A:79:LYS:HA	4	0.11
(1,524)	1:A:19:GLU:HA	1:A:79:LYS:HA	5	0.11
(1,524)	1:A:19:GLU:HA	1:A:79:LYS:HA	8	0.11
(1,47)	1:A:3:LYS:HG2	1:A:53:ILE:HA	4	0.11
(1,460)	1:A:17:GLN:HA	1:A:17:GLN:HG2	4	0.11
(1,460)	1:A:17:GLN:HA	1:A:17:GLN:HG2	5	0.11
(1,460)	1:A:17:GLN:HA	1:A:17:GLN:HG2	10	0.11
(1,439)	1:A:15:ILE:H	1:A:15:ILE:HG12	2	0.11
(1,439)	1:A:15:ILE:H	1:A:15:ILE:HG12	9	0.11

Continued on next page...

Continued from previous page...

Key	Atom-1	Atom-2	Model ID	Violation (Å)
(1,439)	1:A:15:ILE:H	1:A:15:ILE:HG12	10	0.11
(1,43)	1:A:3:LYS:HG3	1:A:4:ILE:H	7	0.11
(1,401)	1:A:15:ILE:HG13	1:A:21:VAL:HB	2	0.11
(1,381)	1:A:15:ILE:HD11	1:A:23:VAL:HG11	7	0.11
(1,381)	1:A:15:ILE:HD11	1:A:23:VAL:HG12	7	0.11
(1,381)	1:A:15:ILE:HD11	1:A:23:VAL:HG13	7	0.11
(1,381)	1:A:15:ILE:HD12	1:A:23:VAL:HG11	7	0.11
(1,381)	1:A:15:ILE:HD12	1:A:23:VAL:HG12	7	0.11
(1,381)	1:A:15:ILE:HD12	1:A:23:VAL:HG13	7	0.11
(1,381)	1:A:15:ILE:HD13	1:A:23:VAL:HG11	7	0.11
(1,381)	1:A:15:ILE:HD13	1:A:23:VAL:HG12	7	0.11
(1,381)	1:A:15:ILE:HD13	1:A:23:VAL:HG13	7	0.11
(1,320)	1:A:14:ILE:HA	1:A:18:ASN:HB3	7	0.11
(1,317)	1:A:14:ILE:HA	1:A:17:GLN:HG3	7	0.11
(1,30)	1:A:2:VAL:H	1:A:2:VAL:HG21	4	0.11
(1,30)	1:A:2:VAL:H	1:A:2:VAL:HG22	4	0.11
(1,30)	1:A:2:VAL:H	1:A:2:VAL:HG23	4	0.11
(1,2548)	1:A:32:PRO:HA	1:A:35:ARG:H	5	0.11
(1,2543)	1:A:27:ALA:HB1	1:A:29:TRP:HD1	6	0.11
(1,2543)	1:A:27:ALA:HB2	1:A:29:TRP:HD1	6	0.11
(1,2543)	1:A:27:ALA:HB3	1:A:29:TRP:HD1	6	0.11
(1,2534)	1:A:103:ALA:H	1:A:103:ALA:HB1	4	0.11
(1,2534)	1:A:103:ALA:H	1:A:103:ALA:HB2	4	0.11
(1,2534)	1:A:103:ALA:H	1:A:103:ALA:HB3	4	0.11
(1,2506)	1:A:101:LYS:H	1:A:103:ALA:H	7	0.11
(1,2468)	1:A:101:LYS:HB2	1:A:103:ALA:H	2	0.11
(1,2415)	1:A:99:ILE:HD11	1:A:103:ALA:H	4	0.11
(1,2415)	1:A:99:ILE:HD12	1:A:103:ALA:H	4	0.11
(1,2415)	1:A:99:ILE:HD13	1:A:103:ALA:H	4	0.11
(1,2407)	1:A:99:ILE:HA	1:A:104:ALA:H	1	0.11
(1,2399)	1:A:98:LEU:H	1:A:99:ILE:HG12	2	0.11
(1,2396)	1:A:98:LEU:H	1:A:98:LEU:HG	4	0.11
(1,2225)	1:A:90:ALA:H	1:A:90:ALA:HB1	8	0.11
(1,2225)	1:A:90:ALA:H	1:A:90:ALA:HB2	8	0.11
(1,2225)	1:A:90:ALA:H	1:A:90:ALA:HB3	8	0.11
(1,2156)	1:A:86:THR:H	1:A:86:THR:HB	2	0.11
(1,2156)	1:A:86:THR:H	1:A:86:THR:HB	6	0.11
(1,2156)	1:A:86:THR:H	1:A:86:THR:HB	8	0.11
(1,2004)	1:A:78:TYR:HD1	1:A:15:ILE:HG13	1	0.11
(1,2004)	1:A:78:TYR:HD2	1:A:15:ILE:HG13	1	0.11
(1,200)	1:A:11:PHE:HA	1:A:15:ILE:H	4	0.11
(1,200)	1:A:11:PHE:HA	1:A:15:ILE:H	5	0.11

Continued on next page...

Continued from previous page...

Key	Atom-1	Atom-2	Model ID	Violation (Å)
(1,1938)	1:A:77:VAL:HG11	1:A:77:VAL:HA	7	0.11
(1,1938)	1:A:77:VAL:HG12	1:A:77:VAL:HA	7	0.11
(1,1938)	1:A:77:VAL:HG13	1:A:77:VAL:HA	7	0.11
(1,1762)	1:A:69:ILE:HG21	1:A:74:THR:HG1	6	0.11
(1,1762)	1:A:69:ILE:HG22	1:A:74:THR:HG1	6	0.11
(1,1762)	1:A:69:ILE:HG23	1:A:74:THR:HG1	6	0.11
(1,1689)	1:A:66:LYS:H	1:A:66:LYS:HG2	4	0.11
(1,1689)	1:A:66:LYS:H	1:A:66:LYS:HG3	4	0.11
(1,1647)	1:A:63:VAL:H	1:A:63:VAL:HG21	1	0.11
(1,1647)	1:A:63:VAL:H	1:A:63:VAL:HG22	1	0.11
(1,1647)	1:A:63:VAL:H	1:A:63:VAL:HG23	1	0.11
(1,164)	1:A:8:GLN:H	1:A:8:GLN:HG2	1	0.11
(1,164)	1:A:8:GLN:H	1:A:8:GLN:HG2	4	0.11
(1,1631)	1:A:63:VAL:HG11	1:A:66:LYS:HG2	6	0.11
(1,1631)	1:A:63:VAL:HG11	1:A:66:LYS:HG3	6	0.11
(1,1631)	1:A:63:VAL:HG12	1:A:66:LYS:HG2	6	0.11
(1,1631)	1:A:63:VAL:HG12	1:A:66:LYS:HG3	6	0.11
(1,1631)	1:A:63:VAL:HG13	1:A:66:LYS:HG2	6	0.11
(1,1631)	1:A:63:VAL:HG13	1:A:66:LYS:HG3	6	0.11
(1,1625)	1:A:63:VAL:HG11	1:A:63:VAL:HB	3	0.11
(1,1625)	1:A:63:VAL:HG12	1:A:63:VAL:HB	3	0.11
(1,1625)	1:A:63:VAL:HG13	1:A:63:VAL:HB	3	0.11
(1,1625)	1:A:63:VAL:HG11	1:A:63:VAL:HB	4	0.11
(1,1625)	1:A:63:VAL:HG12	1:A:63:VAL:HB	4	0.11
(1,1625)	1:A:63:VAL:HG13	1:A:63:VAL:HB	4	0.11
(1,1625)	1:A:63:VAL:HG11	1:A:63:VAL:HB	5	0.11
(1,1625)	1:A:63:VAL:HG12	1:A:63:VAL:HB	5	0.11
(1,1625)	1:A:63:VAL:HG13	1:A:63:VAL:HB	5	0.11
(1,1625)	1:A:63:VAL:HG11	1:A:63:VAL:HB	6	0.11
(1,1625)	1:A:63:VAL:HG12	1:A:63:VAL:HB	6	0.11
(1,1625)	1:A:63:VAL:HG13	1:A:63:VAL:HB	6	0.11
(1,1625)	1:A:63:VAL:HG11	1:A:63:VAL:HB	8	0.11
(1,1625)	1:A:63:VAL:HG12	1:A:63:VAL:HB	8	0.11
(1,1625)	1:A:63:VAL:HG13	1:A:63:VAL:HB	8	0.11
(1,1625)	1:A:63:VAL:HG11	1:A:63:VAL:HB	10	0.11
(1,1625)	1:A:63:VAL:HG12	1:A:63:VAL:HB	10	0.11
(1,1625)	1:A:63:VAL:HG13	1:A:63:VAL:HB	10	0.11
(1,1555)	1:A:57:VAL:H	1:A:57:VAL:HG11	3	0.11
(1,1555)	1:A:57:VAL:H	1:A:57:VAL:HG12	3	0.11
(1,1555)	1:A:57:VAL:H	1:A:57:VAL:HG13	3	0.11
(1,1550)	1:A:57:VAL:HG21	1:A:58:ASP:H	9	0.11
(1,1550)	1:A:57:VAL:HG22	1:A:58:ASP:H	9	0.11

Continued on next page...

Continued from previous page...

Key	Atom-1	Atom-2	Model ID	Violation (Å)
(1,1550)	1:A:57:VAL:HG23	1:A:58:ASP:H	9	0.11
(1,153)	1:A:8:GLN:HE22	1:A:9:SER:H	10	0.11
(1,1518)	1:A:54:LYS:H	1:A:54:LYS:HB2	9	0.11
(1,1505)	1:A:54:LYS:HA	1:A:54:LYS:HG3	3	0.11
(1,1487)	1:A:52:PHE:HZ	1:A:99:ILE:HG13	8	0.11
(1,1433)	1:A:50:MET:HG3	1:A:103:ALA:H	2	0.11
(1,1433)	1:A:50:MET:HG3	1:A:103:ALA:H	8	0.11
(1,1432)	1:A:50:MET:HG3	1:A:52:PHE:HZ	2	0.11
(1,1432)	1:A:50:MET:HG3	1:A:52:PHE:HZ	9	0.11
(1,1419)	1:A:49:LYS:H	1:A:49:LYS:HB3	6	0.11
(1,1259)	1:A:43:CYS:HA	1:A:52:PHE:HZ	2	0.11
(1,1259)	1:A:43:CYS:HA	1:A:52:PHE:HZ	5	0.11
(1,1259)	1:A:43:CYS:HA	1:A:52:PHE:HZ	9	0.11
(1,1218)	1:A:40:TYR:H	1:A:92:ASP:HA	5	0.11
(1,1133)	1:A:39:PHE:HB2	1:A:41:GLU:H	6	0.11
(1,1133)	1:A:39:PHE:HB2	1:A:41:GLU:H	7	0.11
(1,1133)	1:A:39:PHE:HB2	1:A:41:GLU:H	10	0.11
(1,1097)	1:A:36:ILE:H	1:A:36:ILE:HG21	7	0.11
(1,1097)	1:A:36:ILE:H	1:A:36:ILE:HG22	7	0.11
(1,1097)	1:A:36:ILE:H	1:A:36:ILE:HG23	7	0.11
(1,1090)	1:A:36:ILE:HG21	1:A:90:ALA:HB1	3	0.11
(1,1090)	1:A:36:ILE:HG21	1:A:90:ALA:HB2	3	0.11
(1,1090)	1:A:36:ILE:HG21	1:A:90:ALA:HB3	3	0.11
(1,1090)	1:A:36:ILE:HG22	1:A:90:ALA:HB1	3	0.11
(1,1090)	1:A:36:ILE:HG22	1:A:90:ALA:HB2	3	0.11
(1,1090)	1:A:36:ILE:HG22	1:A:90:ALA:HB3	3	0.11
(1,1090)	1:A:36:ILE:HG23	1:A:90:ALA:HB1	3	0.11
(1,1090)	1:A:36:ILE:HG23	1:A:90:ALA:HB2	3	0.11
(1,1090)	1:A:36:ILE:HG23	1:A:90:ALA:HB3	3	0.11
(1,1090)	1:A:36:ILE:HG21	1:A:90:ALA:HB1	10	0.11
(1,1090)	1:A:36:ILE:HG21	1:A:90:ALA:HB2	10	0.11
(1,1090)	1:A:36:ILE:HG21	1:A:90:ALA:HB3	10	0.11
(1,1090)	1:A:36:ILE:HG22	1:A:90:ALA:HB1	10	0.11
(1,1090)	1:A:36:ILE:HG22	1:A:90:ALA:HB2	10	0.11
(1,1090)	1:A:36:ILE:HG22	1:A:90:ALA:HB3	10	0.11
(1,1090)	1:A:36:ILE:HG23	1:A:90:ALA:HB1	10	0.11
(1,1090)	1:A:36:ILE:HG23	1:A:90:ALA:HB2	10	0.11
(1,1090)	1:A:36:ILE:HG23	1:A:90:ALA:HB3	10	0.11
(1,1074)	1:A:36:ILE:HG12	1:A:75:PHE:HE1	2	0.11
(1,1074)	1:A:36:ILE:HG12	1:A:75:PHE:HE2	2	0.11
(1,1074)	1:A:36:ILE:HG12	1:A:75:PHE:HE1	6	0.11
(1,1074)	1:A:36:ILE:HG12	1:A:75:PHE:HE2	6	0.11

Continued on next page...

Continued from previous page...

Key	Atom-1	Atom-2	Model ID	Violation (Å)
(1,1074)	1:A:36:ILE:HG12	1:A:75:PHE:HE1	9	0.11
(1,1074)	1:A:36:ILE:HG12	1:A:75:PHE:HE2	9	0.11

10 Dihedral-angle violation analysis (i)

10.1 Summary of dihedral-angle violations (i)

The following table provides the summary of dihedral-angle violations in different dihedral-angle types. Violations less than 1° are not included in the calculation.

Angle type	Count	%[1]	Violated[3]			Consistently Violated[4]		
			Count	%[2]	%[1]	Count	%[2]	%[1]
PSI	91	50.0	30	33.0	16.5	11	12.1	6.0
PHI	91	50.0	36	39.6	19.8	11	12.1	6.0
Total	182	100.0	66	36.3	36.3	22	12.1	12.1

[1] percentage calculated with respect to total number of dihedral-angle restraints, [2] percentage calculated with respect to number of restraints in a particular dihedral-angle type, [3] violated in at least one model, [4] violated in all the models

10.1.1 Bar chart : Distribution of dihedral-angles and violations (i)

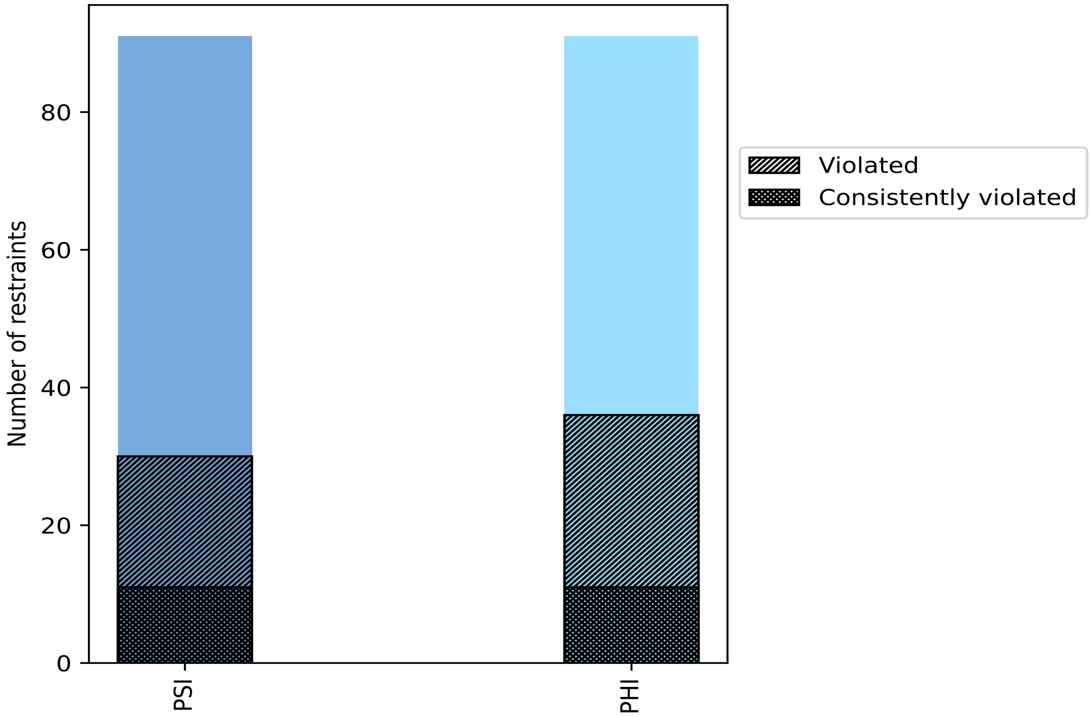

Violated and consistently violated restraints are shown using different hatch patterns in their respective categories

10.2 Dihedral-angle violation statistics for each model (i)

The following table provides the dihedral-angle violation statistics for each model in the ensemble. Violations less than 1° are not included in the statistics.

Model ID	Number of violations			Mean (°)	Max (°)	SD (°)	Median (°)
	PSI	PHI	Total				
1	20	21	41	3.11	13.7	2.99	1.8
2	20	23	43	3.02	12.1	3.01	1.9
3	23	23	46	2.87	11.8	2.83	1.75
4	21	22	43	3.15	14.2	3.03	1.9
5	19	21	40	3.28	12.1	2.99	1.9
6	20	20	40	3.17	11.9	3.02	1.9
7	19	21	40	3.02	12.5	2.93	1.7
8	21	22	43	3.05	12.0	2.83	1.8
9	17	20	37	3.3	13.8	3.13	1.9
10	18	24	42	2.99	12.1	2.85	1.85

10.2.1 Bar graph : Dihedral violation statistics for each model (i)

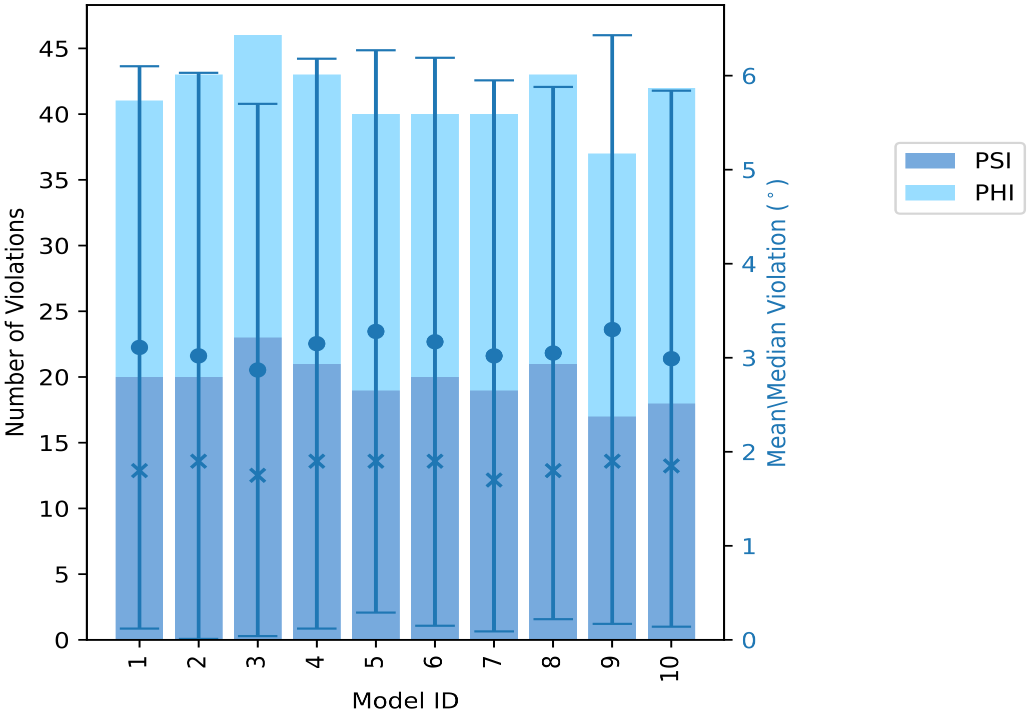

The mean(dot),median(x) and the standard deviation are shown in blue with respect to the y axis on the right

10.3 Dihedral-angle violation statistics for the ensemble (i)

Violation analysis may find that some restraints are violated in very few models and some are violated in most of models. The following table provides this information as number of violated restraints for a given fraction of ensemble.

Number of violated restraints			Fraction of the ensemble	
PSI	PHI	Total	Count[1]	%
4	7	11	1	10.0
2	2	4	2	20.0
4	3	7	3	30.0
1	2	3	4	40.0
1	3	4	5	50.0
1	0	1	6	60.0
0	3	3	7	70.0
1	2	3	8	80.0
5	3	8	9	90.0
11	11	22	10	100.0

[1] Number of models with violations

10.3.1 Bar graph : Dihedral-angle Violation statistics for the ensemble (i)

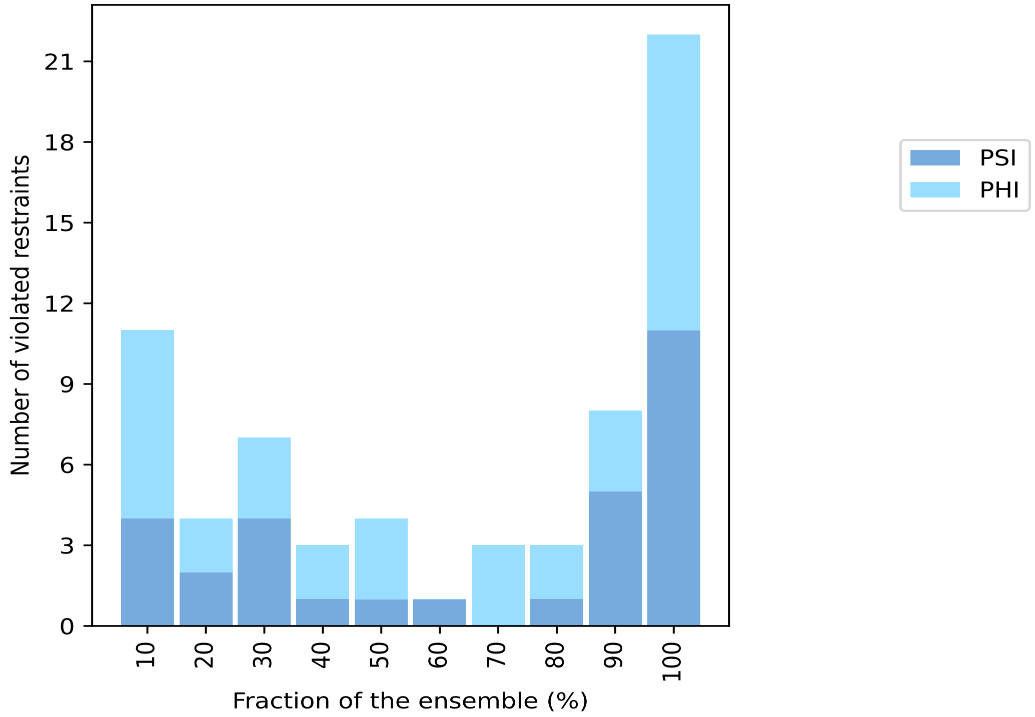

10.4 Most violated dihedral-angle restraints in the ensemble (i)

10.4.1 Histogram : Distribution of mean dihedral-angle violations (i)

The following histogram shows the distribution of the average value of the violation. The average is calculated for each restraint that is violated in more than one model over all the violated models in the ensemble

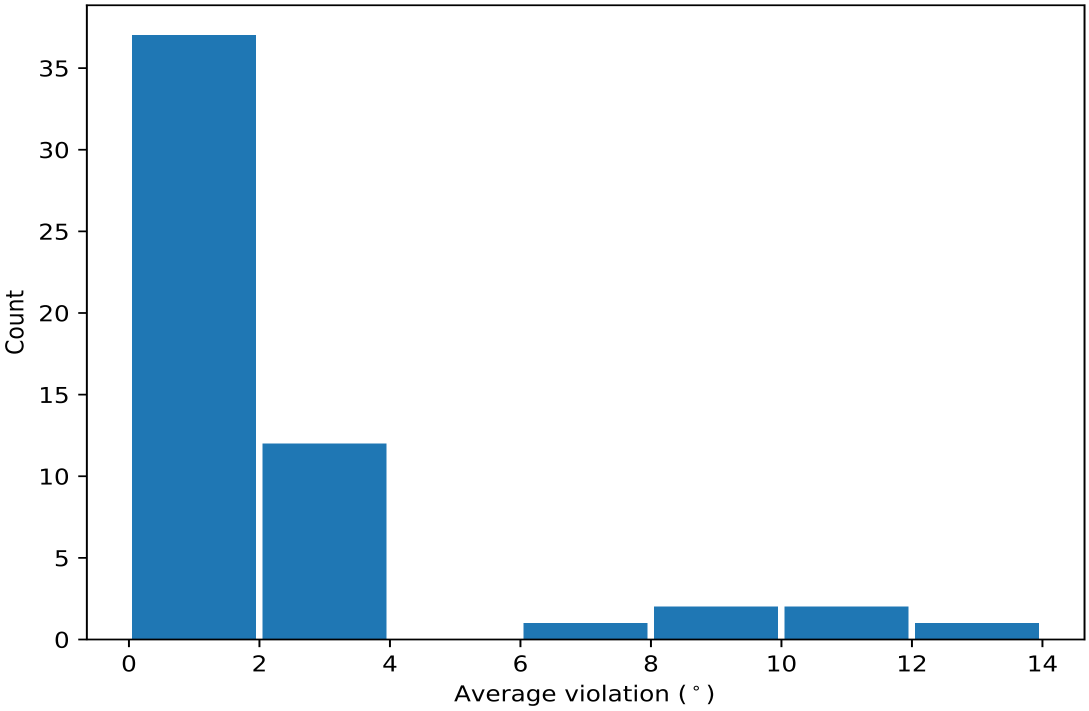

10.4.2 Table: Most violated dihedral-angle restraints (i)

The following table provides the mean and the standard deviation of the violation for each restraint sorted by number of violated models and the mean value. The Key (restraint list ID, restraint ID) is the unique identifier for a given restraint.

Key	Atom-1	Atom-2	Atom-3	Atom-4	Models[1]	Mean	SD[2]	Median
(1,53)	1:A:28:GLU:C	1:A:29:TRP:N	1:A:29:TRP:CA	1:A:29:TRP:C	10	12.62	0.86	12.1
(1,52)	1:A:28:GLU:N	1:A:28:GLU:CA	1:A:28:GLU:C	1:A:29:TRP:N	10	10.82	0.42	11.0
(1,88)	1:A:47:TYR:N	1:A:47:TYR:CA	1:A:47:TYR:C	1:A:48:THR:N	10	10.1	0.67	9.8
(1,48)	1:A:26:PHE:N	1:A:26:PHE:CA	1:A:26:PHE:C	1:A:27:ALA:N	10	9.85	0.62	9.8
(1,89)	1:A:47:TYR:C	1:A:48:THR:N	1:A:48:THR:CA	1:A:48:THR:C	10	8.67	0.71	8.4
(1,51)	1:A:27:ALA:C	1:A:28:GLU:N	1:A:28:GLU:CA	1:A:28:GLU:C	10	7.08	0.29	7.0
(1,61)	1:A:33:CYS:C	1:A:34:LYS:N	1:A:34:LYS:CA	1:A:34:LYS:C	10	3.77	0.86	3.5
(1,158)	1:A:90:ALA:N	1:A:90:ALA:CA	1:A:90:ALA:C	1:A:91:ASN:N	10	3.58	0.39	3.55
(1,131)	1:A:72:MET:C	1:A:73:PRO:N	1:A:73:PRO:CA	1:A:73:PRO:C	10	3.15	0.73	3.55
(1,163)	1:A:92:ASP:C	1:A:93:SER:N	1:A:93:SER:CA	1:A:93:SER:C	10	2.89	0.47	2.9
(1,159)	1:A:90:ALA:C	1:A:91:ASN:N	1:A:91:ASN:CA	1:A:91:ASN:C	10	2.81	0.34	2.9
(1,162)	1:A:92:ASP:N	1:A:92:ASP:CA	1:A:92:ASP:C	1:A:93:SER:N	10	2.76	0.36	2.8
(1,180)	1:A:101:LYS:N	1:A:101:LYS:CA	1:A:101:LYS:C	1:A:102:TYR:N	10	2.63	0.62	2.65

Continued on next page...

Continued from previous page...

Key	Atom-1	Atom-2	Atom-3	Atom-4	Models[1]	Mean	SD[2]	Median
(1,73)	1:A:39:PHE:C	1:A:40:TYR:N	1:A:40:TYR:CA	1:A:40:TYR:C	10	2.55	0.37	2.5
(1,46)	1:A:25:PHE:N	1:A:25:PHE:CA	1:A:25:PHE:C	1:A:26:PHE:N	10	2.4	0.79	2.35
(1,45)	1:A:24:ASP:C	1:A:25:PHE:N	1:A:25:PHE:CA	1:A:25:PHE:C	10	1.93	0.51	1.8
(1,173)	1:A:97:GLN:C	1:A:98:LEU:N	1:A:98:LEU:CA	1:A:98:LEU:C	10	1.76	0.36	1.7
(1,12)	1:A:8:GLN:N	1:A:8:GLN:CA	1:A:8:GLN:C	1:A:9:SER:N	10	1.71	0.32	1.65
(1,169)	1:A:95:LEU:C	1:A:96:LYS:N	1:A:96:LYS:CA	1:A:96:LYS:C	10	1.67	0.33	1.75
(1,64)	1:A:35:ARG:N	1:A:35:ARG:CA	1:A:35:ARG:C	1:A:36:ILE:N	10	1.66	0.28	1.5
(1,60)	1:A:33:CYS:N	1:A:33:CYS:CA	1:A:33:CYS:C	1:A:34:LYS:N	10	1.65	0.99	1.4
(1,18)	1:A:11:PHE:N	1:A:11:PHE:CA	1:A:11:PHE:C	1:A:12:ASP:N	10	1.49	0.27	1.4
(1,178)	1:A:100:GLU:N	1:A:100:GLU:CA	1:A:100:GLU:C	1:A:101:LYS:N	9	2.38	0.78	2.3
(1,39)	1:A:21:VAL:C	1:A:22:ILE:N	1:A:22:ILE:CA	1:A:22:ILE:C	9	2.23	0.66	2.2
(1,74)	1:A:40:TYR:N	1:A:40:TYR:CA	1:A:40:TYR:C	1:A:41:GLU:N	9	1.79	0.37	1.9
(1,30)	1:A:17:GLN:N	1:A:17:GLN:CA	1:A:17:GLN:C	1:A:18:ASN:N	9	1.67	0.25	1.7
(1,27)	1:A:15:ILE:C	1:A:16:SER:N	1:A:16:SER:CA	1:A:16:SER:C	9	1.63	0.21	1.6
(1,174)	1:A:98:LEU:N	1:A:98:LEU:CA	1:A:98:LEU:C	1:A:99:ILE:N	9	1.56	0.28	1.4
(1,175)	1:A:98:LEU:C	1:A:99:ILE:N	1:A:99:ILE:CA	1:A:99:ILE:C	9	1.54	0.44	1.4
(1,146)	1:A:80:ASN:N	1:A:80:ASN:CA	1:A:80:ASN:C	1:A:81:GLY:N	9	1.43	0.16	1.4
(1,40)	1:A:22:ILE:N	1:A:22:ILE:CA	1:A:22:ILE:C	1:A:23:VAL:N	8	1.67	0.18	1.7
(1,181)	1:A:101:LYS:C	1:A:102:TYR:N	1:A:102:TYR:CA	1:A:102:TYR:C	8	1.64	0.32	1.6
(1,69)	1:A:37:ALA:C	1:A:38:PRO:N	1:A:38:PRO:CA	1:A:38:PRO:C	8	1.49	0.2	1.5
(1,93)	1:A:50:MET:C	1:A:51:VAL:N	1:A:51:VAL:CA	1:A:51:VAL:C	7	1.43	0.23	1.4
(1,1)	1:A:2:VAL:C	1:A:3:LYS:N	1:A:3:LYS:CA	1:A:3:LYS:C	7	1.41	0.22	1.4
(1,59)	1:A:32:PRO:C	1:A:33:CYS:N	1:A:33:CYS:CA	1:A:33:CYS:C	7	1.33	0.2	1.3
(1,10)	1:A:7:SER:N	1:A:7:SER:CA	1:A:7:SER:C	1:A:8:GLN:N	6	1.38	0.25	1.35
(1,55)	1:A:30:CYS:C	1:A:31:GLY:N	1:A:31:GLY:CA	1:A:31:GLY:C	5	2.16	0.08	2.2
(1,177)	1:A:99:ILE:C	1:A:100:GLU:N	1:A:100:GLU:CA	1:A:100:GLU:C	5	1.7	0.46	1.4
(1,90)	1:A:48:THR:N	1:A:48:THR:CA	1:A:48:THR:C	1:A:49:LYS:N	5	1.4	0.25	1.3
(1,171)	1:A:96:LYS:C	1:A:97:GLN:N	1:A:97:GLN:CA	1:A:97:GLN:C	5	1.4	0.23	1.4
(1,49)	1:A:26:PHE:C	1:A:27:ALA:N	1:A:27:ALA:CA	1:A:27:ALA:C	4	1.62	0.45	1.4
(1,149)	1:A:84:VAL:C	1:A:85:ASP:N	1:A:85:ASP:CA	1:A:85:ASP:C	4	1.4	0.41	1.2
(1,56)	1:A:31:GLY:N	1:A:31:GLY:CA	1:A:31:GLY:C	1:A:32:PRO:N	4	1.2	0.07	1.2
(1,75)	1:A:40:TYR:C	1:A:41:GLU:N	1:A:41:GLU:CA	1:A:41:GLU:C	3	1.53	0.24	1.7
(1,41)	1:A:22:ILE:C	1:A:23:VAL:N	1:A:23:VAL:CA	1:A:23:VAL:C	3	1.43	0.19	1.3
(1,2)	1:A:3:LYS:N	1:A:3:LYS:CA	1:A:3:LYS:C	1:A:4:ILE:N	3	1.3	0.14	1.2
(1,28)	1:A:16:SER:N	1:A:16:SER:CA	1:A:16:SER:C	1:A:17:GLN:N	3	1.27	0.09	1.2
(1,36)	1:A:20:LEU:N	1:A:20:LEU:CA	1:A:20:LEU:C	1:A:21:VAL:N	3	1.27	0.05	1.3
(1,29)	1:A:16:SER:C	1:A:17:GLN:N	1:A:17:GLN:CA	1:A:17:GLN:C	3	1.17	0.05	1.2
(1,164)	1:A:93:SER:N	1:A:93:SER:CA	1:A:93:SER:C	1:A:94:ALA:N	3	1.13	0.05	1.1
(1,37)	1:A:20:LEU:C	1:A:21:VAL:N	1:A:21:VAL:CA	1:A:21:VAL:C	2	1.4	0.3	1.4
(1,119)	1:A:64:THR:C	1:A:65:GLU:N	1:A:65:GLU:CA	1:A:65:GLU:C	2	1.4	0.2	1.4
(1,102)	1:A:55:VAL:N	1:A:55:VAL:CA	1:A:55:VAL:C	1:A:56:ASP:N	2	1.3	0.2	1.3
(1,80)	1:A:43:CYS:N	1:A:43:CYS:CA	1:A:43:CYS:C	1:A:44:SER:N	2	1.15	0.05	1.15

[1] Number of violated models, [2]Standard deviation, All angle values are in degree (°)

10.5 All violated dihedral-angle restraints (i)

10.5.1 Histogram : Distribution of violations (i)

The following histogram shows the distribution of the absolute value of the violation for all violated restraints in the ensemble.

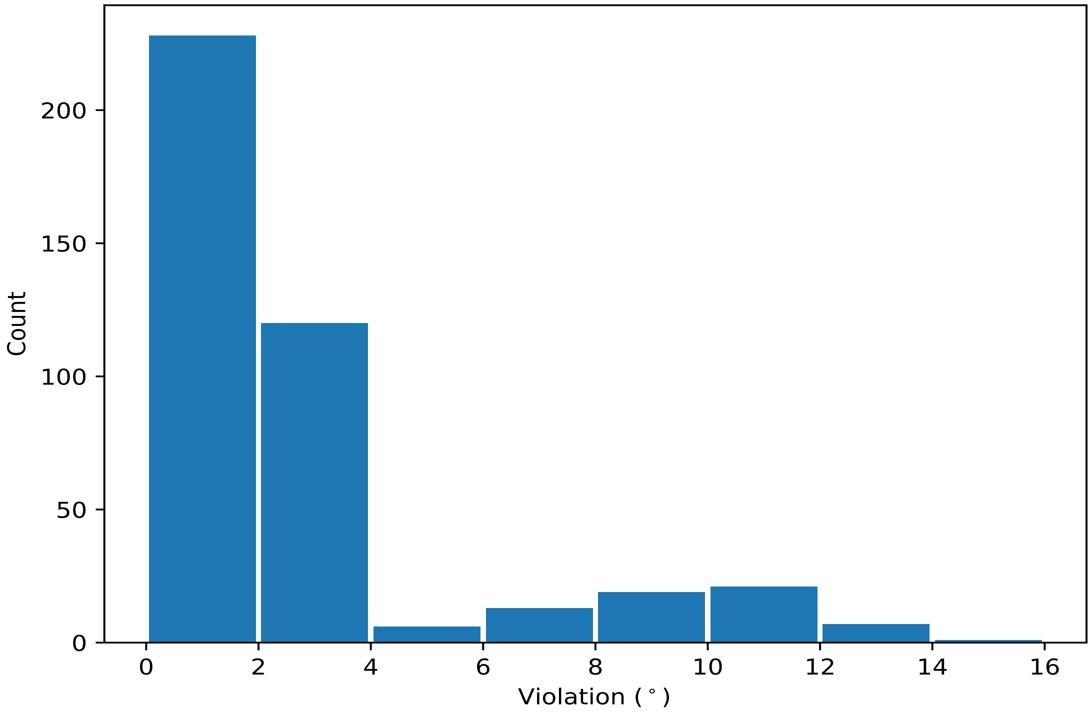

10.5.2 Table: All violated dihedral-angle restraints (i)

The following table lists the absolute value of the violation for each restraint in the ensemble sorted by its value. The Key (restraint list ID, restraint ID) is the unique identifier for a given restraint.

Key	Atom-1	Atom-2	Atom-3	Atom-4	Model ID	Violation (°)
(1,53)	1:A:28:GLU:C	1:A:29:TRP:N	1:A:29:TRP:CA	1:A:29:TRP:C	4	14.2
(1,53)	1:A:28:GLU:C	1:A:29:TRP:N	1:A:29:TRP:CA	1:A:29:TRP:C	9	13.8
(1,53)	1:A:28:GLU:C	1:A:29:TRP:N	1:A:29:TRP:CA	1:A:29:TRP:C	1	13.7
(1,53)	1:A:28:GLU:C	1:A:29:TRP:N	1:A:29:TRP:CA	1:A:29:TRP:C	7	12.5
(1,53)	1:A:28:GLU:C	1:A:29:TRP:N	1:A:29:TRP:CA	1:A:29:TRP:C	2	12.1
(1,53)	1:A:28:GLU:C	1:A:29:TRP:N	1:A:29:TRP:CA	1:A:29:TRP:C	5	12.1
(1,53)	1:A:28:GLU:C	1:A:29:TRP:N	1:A:29:TRP:CA	1:A:29:TRP:C	10	12.1
(1,53)	1:A:28:GLU:C	1:A:29:TRP:N	1:A:29:TRP:CA	1:A:29:TRP:C	8	12.0
(1,53)	1:A:28:GLU:C	1:A:29:TRP:N	1:A:29:TRP:CA	1:A:29:TRP:C	6	11.9
(1,53)	1:A:28:GLU:C	1:A:29:TRP:N	1:A:29:TRP:CA	1:A:29:TRP:C	3	11.8
(1,88)	1:A:47:TYR:N	1:A:47:TYR:CA	1:A:47:TYR:C	1:A:48:THR:N	2	11.4
(1,52)	1:A:28:GLU:N	1:A:28:GLU:CA	1:A:28:GLU:C	1:A:29:TRP:N	5	11.3
(1,52)	1:A:28:GLU:N	1:A:28:GLU:CA	1:A:28:GLU:C	1:A:29:TRP:N	3	11.2
(1,52)	1:A:28:GLU:N	1:A:28:GLU:CA	1:A:28:GLU:C	1:A:29:TRP:N	6	11.2

Continued on next page...

Continued from previous page...

Key	Atom-1	Atom-2	Atom-3	Atom-4	Model ID	Violation (°)
(1,52)	1:A:28:GLU:N	1:A:28:GLU:CA	1:A:28:GLU:C	1:A:29:TRP:N	8	11.1
(1,52)	1:A:28:GLU:N	1:A:28:GLU:CA	1:A:28:GLU:C	1:A:29:TRP:N	10	11.1
(1,88)	1:A:47:TYR:N	1:A:47:TYR:CA	1:A:47:TYR:C	1:A:48:THR:N	4	11.0
(1,52)	1:A:28:GLU:N	1:A:28:GLU:CA	1:A:28:GLU:C	1:A:29:TRP:N	2	10.9
(1,88)	1:A:47:TYR:N	1:A:47:TYR:CA	1:A:47:TYR:C	1:A:48:THR:N	6	10.7
(1,52)	1:A:28:GLU:N	1:A:28:GLU:CA	1:A:28:GLU:C	1:A:29:TRP:N	7	10.7
(1,48)	1:A:26:PHE:N	1:A:26:PHE:CA	1:A:26:PHE:C	1:A:27:ALA:N	8	10.7
(1,48)	1:A:26:PHE:N	1:A:26:PHE:CA	1:A:26:PHE:C	1:A:27:ALA:N	3	10.6
(1,48)	1:A:26:PHE:N	1:A:26:PHE:CA	1:A:26:PHE:C	1:A:27:ALA:N	1	10.4
(1,52)	1:A:28:GLU:N	1:A:28:GLU:CA	1:A:28:GLU:C	1:A:29:TRP:N	4	10.3
(1,88)	1:A:47:TYR:N	1:A:47:TYR:CA	1:A:47:TYR:C	1:A:48:THR:N	5	10.2
(1,52)	1:A:28:GLU:N	1:A:28:GLU:CA	1:A:28:GLU:C	1:A:29:TRP:N	1	10.2
(1,52)	1:A:28:GLU:N	1:A:28:GLU:CA	1:A:28:GLU:C	1:A:29:TRP:N	9	10.2
(1,48)	1:A:26:PHE:N	1:A:26:PHE:CA	1:A:26:PHE:C	1:A:27:ALA:N	9	10.1
(1,89)	1:A:47:TYR:C	1:A:48:THR:N	1:A:48:THR:CA	1:A:48:THR:C	2	10.0
(1,48)	1:A:26:PHE:N	1:A:26:PHE:CA	1:A:26:PHE:C	1:A:27:ALA:N	6	9.9
(1,88)	1:A:47:TYR:N	1:A:47:TYR:CA	1:A:47:TYR:C	1:A:48:THR:N	7	9.8
(1,88)	1:A:47:TYR:N	1:A:47:TYR:CA	1:A:47:TYR:C	1:A:48:THR:N	9	9.8
(1,88)	1:A:47:TYR:N	1:A:47:TYR:CA	1:A:47:TYR:C	1:A:48:THR:N	3	9.7
(1,48)	1:A:26:PHE:N	1:A:26:PHE:CA	1:A:26:PHE:C	1:A:27:ALA:N	2	9.7
(1,48)	1:A:26:PHE:N	1:A:26:PHE:CA	1:A:26:PHE:C	1:A:27:ALA:N	10	9.7
(1,88)	1:A:47:TYR:N	1:A:47:TYR:CA	1:A:47:TYR:C	1:A:48:THR:N	10	9.6
(1,48)	1:A:26:PHE:N	1:A:26:PHE:CA	1:A:26:PHE:C	1:A:27:ALA:N	5	9.6
(1,88)	1:A:47:TYR:N	1:A:47:TYR:CA	1:A:47:TYR:C	1:A:48:THR:N	1	9.5
(1,89)	1:A:47:TYR:C	1:A:48:THR:N	1:A:48:THR:CA	1:A:48:THR:C	6	9.4
(1,89)	1:A:47:TYR:C	1:A:48:THR:N	1:A:48:THR:CA	1:A:48:THR:C	4	9.3
(1,88)	1:A:47:TYR:N	1:A:47:TYR:CA	1:A:47:TYR:C	1:A:48:THR:N	8	9.3
(1,48)	1:A:26:PHE:N	1:A:26:PHE:CA	1:A:26:PHE:C	1:A:27:ALA:N	7	9.3
(1,89)	1:A:47:TYR:C	1:A:48:THR:N	1:A:48:THR:CA	1:A:48:THR:C	5	9.1
(1,89)	1:A:47:TYR:C	1:A:48:THR:N	1:A:48:THR:CA	1:A:48:THR:C	9	8.5
(1,48)	1:A:26:PHE:N	1:A:26:PHE:CA	1:A:26:PHE:C	1:A:27:ALA:N	4	8.5
(1,89)	1:A:47:TYR:C	1:A:48:THR:N	1:A:48:THR:CA	1:A:48:THR:C	3	8.3
(1,89)	1:A:47:TYR:C	1:A:48:THR:N	1:A:48:THR:CA	1:A:48:THR:C	7	8.3
(1,89)	1:A:47:TYR:C	1:A:48:THR:N	1:A:48:THR:CA	1:A:48:THR:C	10	8.3
(1,89)	1:A:47:TYR:C	1:A:48:THR:N	1:A:48:THR:CA	1:A:48:THR:C	1	7.9
(1,89)	1:A:47:TYR:C	1:A:48:THR:N	1:A:48:THR:CA	1:A:48:THR:C	8	7.6
(1,51)	1:A:27:ALA:C	1:A:28:GLU:N	1:A:28:GLU:CA	1:A:28:GLU:C	1	7.5
(1,51)	1:A:27:ALA:C	1:A:28:GLU:N	1:A:28:GLU:CA	1:A:28:GLU:C	4	7.5
(1,51)	1:A:27:ALA:C	1:A:28:GLU:N	1:A:28:GLU:CA	1:A:28:GLU:C	9	7.5
(1,51)	1:A:27:ALA:C	1:A:28:GLU:N	1:A:28:GLU:CA	1:A:28:GLU:C	2	7.0
(1,51)	1:A:27:ALA:C	1:A:28:GLU:N	1:A:28:GLU:CA	1:A:28:GLU:C	3	7.0
(1,51)	1:A:27:ALA:C	1:A:28:GLU:N	1:A:28:GLU:CA	1:A:28:GLU:C	6	7.0
(1,51)	1:A:27:ALA:C	1:A:28:GLU:N	1:A:28:GLU:CA	1:A:28:GLU:C	7	6.9
(1,51)	1:A:27:ALA:C	1:A:28:GLU:N	1:A:28:GLU:CA	1:A:28:GLU:C	8	6.9
(1,51)	1:A:27:ALA:C	1:A:28:GLU:N	1:A:28:GLU:CA	1:A:28:GLU:C	10	6.8
(1,51)	1:A:27:ALA:C	1:A:28:GLU:N	1:A:28:GLU:CA	1:A:28:GLU:C	5	6.7
(1,61)	1:A:33:CYS:C	1:A:34:LYS:N	1:A:34:LYS:CA	1:A:34:LYS:C	5	6.0
(1,60)	1:A:33:CYS:N	1:A:33:CYS:CA	1:A:33:CYS:C	1:A:34:LYS:N	5	4.6
(1,61)	1:A:33:CYS:C	1:A:34:LYS:N	1:A:34:LYS:CA	1:A:34:LYS:C	1	4.4
(1,158)	1:A:90:ALA:N	1:A:90:ALA:CA	1:A:90:ALA:C	1:A:91:ASN:N	7	4.4
(1,46)	1:A:25:PHE:N	1:A:25:PHE:CA	1:A:25:PHE:C	1:A:26:PHE:N	8	4.1

Continued on next page...

Continued from previous page...

Key	Atom-1	Atom-2	Atom-3	Atom-4	Model ID	Violation (°)
(1,131)	1:A:72:MET:C	1:A:73:PRO:N	1:A:73:PRO:CA	1:A:73:PRO:C	6	4.1
(1,61)	1:A:33:CYS:C	1:A:34:LYS:N	1:A:34:LYS:CA	1:A:34:LYS:C	4	4.0
(1,163)	1:A:92:ASP:C	1:A:93:SER:N	1:A:93:SER:CA	1:A:93:SER:C	5	3.9
(1,158)	1:A:90:ALA:N	1:A:90:ALA:CA	1:A:90:ALA:C	1:A:91:ASN:N	3	3.9
(1,61)	1:A:33:CYS:C	1:A:34:LYS:N	1:A:34:LYS:CA	1:A:34:LYS:C	2	3.8
(1,178)	1:A:100:GLU:N	1:A:100:GLU:CA	1:A:100:GLU:C	1:A:101:LYS:N	4	3.8
(1,158)	1:A:90:ALA:N	1:A:90:ALA:CA	1:A:90:ALA:C	1:A:91:ASN:N	1	3.8
(1,158)	1:A:90:ALA:N	1:A:90:ALA:CA	1:A:90:ALA:C	1:A:91:ASN:N	6	3.8
(1,61)	1:A:33:CYS:C	1:A:34:LYS:N	1:A:34:LYS:CA	1:A:34:LYS:C	9	3.7
(1,39)	1:A:21:VAL:C	1:A:22:ILE:N	1:A:22:ILE:CA	1:A:22:ILE:C	10	3.7
(1,131)	1:A:72:MET:C	1:A:73:PRO:N	1:A:73:PRO:CA	1:A:73:PRO:C	3	3.7
(1,131)	1:A:72:MET:C	1:A:73:PRO:N	1:A:73:PRO:CA	1:A:73:PRO:C	4	3.7
(1,180)	1:A:101:LYS:N	1:A:101:LYS:CA	1:A:101:LYS:C	1:A:102:TYR:N	9	3.6
(1,158)	1:A:90:ALA:N	1:A:90:ALA:CA	1:A:90:ALA:C	1:A:91:ASN:N	10	3.6
(1,131)	1:A:72:MET:C	1:A:73:PRO:N	1:A:73:PRO:CA	1:A:73:PRO:C	7	3.6
(1,131)	1:A:72:MET:C	1:A:73:PRO:N	1:A:73:PRO:CA	1:A:73:PRO:C	9	3.6
(1,158)	1:A:90:ALA:N	1:A:90:ALA:CA	1:A:90:ALA:C	1:A:91:ASN:N	8	3.5
(1,131)	1:A:72:MET:C	1:A:73:PRO:N	1:A:73:PRO:CA	1:A:73:PRO:C	2	3.5
(1,163)	1:A:92:ASP:C	1:A:93:SER:N	1:A:93:SER:CA	1:A:93:SER:C	8	3.4
(1,159)	1:A:90:ALA:C	1:A:91:ASN:N	1:A:91:ASN:CA	1:A:91:ASN:C	7	3.4
(1,61)	1:A:33:CYS:C	1:A:34:LYS:N	1:A:34:LYS:CA	1:A:34:LYS:C	3	3.3
(1,61)	1:A:33:CYS:C	1:A:34:LYS:N	1:A:34:LYS:CA	1:A:34:LYS:C	7	3.3
(1,45)	1:A:24:ASP:C	1:A:25:PHE:N	1:A:25:PHE:CA	1:A:25:PHE:C	8	3.3
(1,162)	1:A:92:ASP:N	1:A:92:ASP:CA	1:A:92:ASP:C	1:A:93:SER:N	8	3.3
(1,158)	1:A:90:ALA:N	1:A:90:ALA:CA	1:A:90:ALA:C	1:A:91:ASN:N	4	3.3
(1,158)	1:A:90:ALA:N	1:A:90:ALA:CA	1:A:90:ALA:C	1:A:91:ASN:N	9	3.3
(1,73)	1:A:39:PHE:C	1:A:40:TYR:N	1:A:40:TYR:CA	1:A:40:TYR:C	8	3.2
(1,61)	1:A:33:CYS:C	1:A:34:LYS:N	1:A:34:LYS:CA	1:A:34:LYS:C	8	3.2
(1,180)	1:A:101:LYS:N	1:A:101:LYS:CA	1:A:101:LYS:C	1:A:102:TYR:N	4	3.2
(1,180)	1:A:101:LYS:N	1:A:101:LYS:CA	1:A:101:LYS:C	1:A:102:TYR:N	5	3.2
(1,178)	1:A:100:GLU:N	1:A:100:GLU:CA	1:A:100:GLU:C	1:A:101:LYS:N	1	3.2
(1,162)	1:A:92:ASP:N	1:A:92:ASP:CA	1:A:92:ASP:C	1:A:93:SER:N	4	3.2
(1,61)	1:A:33:CYS:C	1:A:34:LYS:N	1:A:34:LYS:CA	1:A:34:LYS:C	10	3.1
(1,180)	1:A:101:LYS:N	1:A:101:LYS:CA	1:A:101:LYS:C	1:A:102:TYR:N	1	3.1
(1,162)	1:A:92:ASP:N	1:A:92:ASP:CA	1:A:92:ASP:C	1:A:93:SER:N	9	3.1
(1,159)	1:A:90:ALA:C	1:A:91:ASN:N	1:A:91:ASN:CA	1:A:91:ASN:C	3	3.1
(1,158)	1:A:90:ALA:N	1:A:90:ALA:CA	1:A:90:ALA:C	1:A:91:ASN:N	2	3.1
(1,158)	1:A:90:ALA:N	1:A:90:ALA:CA	1:A:90:ALA:C	1:A:91:ASN:N	5	3.1
(1,73)	1:A:39:PHE:C	1:A:40:TYR:N	1:A:40:TYR:CA	1:A:40:TYR:C	3	3.0
(1,178)	1:A:100:GLU:N	1:A:100:GLU:CA	1:A:100:GLU:C	1:A:101:LYS:N	9	3.0
(1,163)	1:A:92:ASP:C	1:A:93:SER:N	1:A:93:SER:CA	1:A:93:SER:C	4	3.0
(1,159)	1:A:90:ALA:C	1:A:91:ASN:N	1:A:91:ASN:CA	1:A:91:ASN:C	1	3.0
(1,159)	1:A:90:ALA:C	1:A:91:ASN:N	1:A:91:ASN:CA	1:A:91:ASN:C	6	3.0
(1,159)	1:A:90:ALA:C	1:A:91:ASN:N	1:A:91:ASN:CA	1:A:91:ASN:C	10	3.0
(1,73)	1:A:39:PHE:C	1:A:40:TYR:N	1:A:40:TYR:CA	1:A:40:TYR:C	5	2.9
(1,61)	1:A:33:CYS:C	1:A:34:LYS:N	1:A:34:LYS:CA	1:A:34:LYS:C	6	2.9
(1,46)	1:A:25:PHE:N	1:A:25:PHE:CA	1:A:25:PHE:C	1:A:26:PHE:N	1	2.9
(1,46)	1:A:25:PHE:N	1:A:25:PHE:CA	1:A:25:PHE:C	1:A:26:PHE:N	4	2.9
(1,163)	1:A:92:ASP:C	1:A:93:SER:N	1:A:93:SER:CA	1:A:93:SER:C	6	2.9
(1,163)	1:A:92:ASP:C	1:A:93:SER:N	1:A:93:SER:CA	1:A:93:SER:C	9	2.9
(1,163)	1:A:92:ASP:C	1:A:93:SER:N	1:A:93:SER:CA	1:A:93:SER:C	10	2.9

Continued on next page...

Continued from previous page...

Key	Atom-1	Atom-2	Atom-3	Atom-4	Model ID	Violation (°)
(1,162)	1:A:92:ASP:N	1:A:92:ASP:CA	1:A:92:ASP:C	1:A:93:SER:N	3	2.9
(1,46)	1:A:25:PHE:N	1:A:25:PHE:CA	1:A:25:PHE:C	1:A:26:PHE:N	5	2.8
(1,46)	1:A:25:PHE:N	1:A:25:PHE:CA	1:A:25:PHE:C	1:A:26:PHE:N	6	2.8
(1,163)	1:A:92:ASP:C	1:A:93:SER:N	1:A:93:SER:CA	1:A:93:SER:C	7	2.8
(1,162)	1:A:92:ASP:N	1:A:92:ASP:CA	1:A:92:ASP:C	1:A:93:SER:N	5	2.8
(1,162)	1:A:92:ASP:N	1:A:92:ASP:CA	1:A:92:ASP:C	1:A:93:SER:N	6	2.8
(1,159)	1:A:90:ALA:C	1:A:91:ASN:N	1:A:91:ASN:CA	1:A:91:ASN:C	9	2.8
(1,131)	1:A:72:MET:C	1:A:73:PRO:N	1:A:73:PRO:CA	1:A:73:PRO:C	8	2.8
(1,73)	1:A:39:PHE:C	1:A:40:TYR:N	1:A:40:TYR:CA	1:A:40:TYR:C	4	2.7
(1,39)	1:A:21:VAL:C	1:A:22:ILE:N	1:A:22:ILE:CA	1:A:22:ILE:C	3	2.7
(1,180)	1:A:101:LYS:N	1:A:101:LYS:CA	1:A:101:LYS:C	1:A:102:TYR:N	2	2.7
(1,159)	1:A:90:ALA:C	1:A:91:ASN:N	1:A:91:ASN:CA	1:A:91:ASN:C	8	2.7
(1,73)	1:A:39:PHE:C	1:A:40:TYR:N	1:A:40:TYR:CA	1:A:40:TYR:C	10	2.6
(1,180)	1:A:101:LYS:N	1:A:101:LYS:CA	1:A:101:LYS:C	1:A:102:TYR:N	8	2.6
(1,175)	1:A:98:LEU:C	1:A:99:ILE:N	1:A:99:ILE:CA	1:A:99:ILE:C	3	2.6
(1,173)	1:A:97:GLN:C	1:A:98:LEU:N	1:A:98:LEU:CA	1:A:98:LEU:C	6	2.6
(1,163)	1:A:92:ASP:C	1:A:93:SER:N	1:A:93:SER:CA	1:A:93:SER:C	3	2.6
(1,39)	1:A:21:VAL:C	1:A:22:ILE:N	1:A:22:ILE:CA	1:A:22:ILE:C	4	2.5
(1,162)	1:A:92:ASP:N	1:A:92:ASP:CA	1:A:92:ASP:C	1:A:93:SER:N	7	2.5
(1,162)	1:A:92:ASP:N	1:A:92:ASP:CA	1:A:92:ASP:C	1:A:93:SER:N	10	2.5
(1,159)	1:A:90:ALA:C	1:A:91:ASN:N	1:A:91:ASN:CA	1:A:91:ASN:C	2	2.5
(1,131)	1:A:72:MET:C	1:A:73:PRO:N	1:A:73:PRO:CA	1:A:73:PRO:C	10	2.5
(1,73)	1:A:39:PHE:C	1:A:40:TYR:N	1:A:40:TYR:CA	1:A:40:TYR:C	2	2.4
(1,49)	1:A:26:PHE:C	1:A:27:ALA:N	1:A:27:ALA:CA	1:A:27:ALA:C	4	2.4
(1,180)	1:A:101:LYS:N	1:A:101:LYS:CA	1:A:101:LYS:C	1:A:102:TYR:N	6	2.4
(1,177)	1:A:99:ILE:C	1:A:100:GLU:N	1:A:100:GLU:CA	1:A:100:GLU:C	10	2.4
(1,159)	1:A:90:ALA:C	1:A:91:ASN:N	1:A:91:ASN:CA	1:A:91:ASN:C	4	2.4
(1,122)	1:A:66:LYS:N	1:A:66:LYS:CA	1:A:66:LYS:C	1:A:67:GLU:N	8	2.4
(1,73)	1:A:39:PHE:C	1:A:40:TYR:N	1:A:40:TYR:CA	1:A:40:TYR:C	6	2.3
(1,39)	1:A:21:VAL:C	1:A:22:ILE:N	1:A:22:ILE:CA	1:A:22:ILE:C	1	2.3
(1,178)	1:A:100:GLU:N	1:A:100:GLU:CA	1:A:100:GLU:C	1:A:101:LYS:N	5	2.3
(1,178)	1:A:100:GLU:N	1:A:100:GLU:CA	1:A:100:GLU:C	1:A:101:LYS:N	8	2.3
(1,163)	1:A:92:ASP:C	1:A:93:SER:N	1:A:93:SER:CA	1:A:93:SER:C	2	2.3
(1,162)	1:A:92:ASP:N	1:A:92:ASP:CA	1:A:92:ASP:C	1:A:93:SER:N	1	2.3
(1,12)	1:A:8:GLN:N	1:A:8:GLN:CA	1:A:8:GLN:C	1:A:9:SER:N	7	2.3
(1,74)	1:A:40:TYR:N	1:A:40:TYR:CA	1:A:40:TYR:C	1:A:41:GLU:N	3	2.2
(1,74)	1:A:40:TYR:N	1:A:40:TYR:CA	1:A:40:TYR:C	1:A:41:GLU:N	6	2.2
(1,73)	1:A:39:PHE:C	1:A:40:TYR:N	1:A:40:TYR:CA	1:A:40:TYR:C	9	2.2
(1,64)	1:A:35:ARG:N	1:A:35:ARG:CA	1:A:35:ARG:C	1:A:36:ILE:N	2	2.2
(1,55)	1:A:30:CYS:C	1:A:31:GLY:N	1:A:31:GLY:CA	1:A:31:GLY:C	3	2.2
(1,55)	1:A:30:CYS:C	1:A:31:GLY:N	1:A:31:GLY:CA	1:A:31:GLY:C	6	2.2
(1,55)	1:A:30:CYS:C	1:A:31:GLY:N	1:A:31:GLY:CA	1:A:31:GLY:C	7	2.2
(1,55)	1:A:30:CYS:C	1:A:31:GLY:N	1:A:31:GLY:CA	1:A:31:GLY:C	10	2.2
(1,45)	1:A:24:ASP:C	1:A:25:PHE:N	1:A:25:PHE:CA	1:A:25:PHE:C	1	2.2
(1,39)	1:A:21:VAL:C	1:A:22:ILE:N	1:A:22:ILE:CA	1:A:22:ILE:C	6	2.2
(1,181)	1:A:101:LYS:C	1:A:102:TYR:N	1:A:102:TYR:CA	1:A:102:TYR:C	5	2.2
(1,178)	1:A:100:GLU:N	1:A:100:GLU:CA	1:A:100:GLU:C	1:A:101:LYS:N	2	2.2
(1,169)	1:A:95:LEU:C	1:A:96:LYS:N	1:A:96:LYS:CA	1:A:96:LYS:C	2	2.2
(1,163)	1:A:92:ASP:C	1:A:93:SER:N	1:A:93:SER:CA	1:A:93:SER:C	1	2.2
(1,162)	1:A:92:ASP:N	1:A:92:ASP:CA	1:A:92:ASP:C	1:A:93:SER:N	2	2.2
(1,159)	1:A:90:ALA:C	1:A:91:ASN:N	1:A:91:ASN:CA	1:A:91:ASN:C	5	2.2

Continued on next page...

Continued from previous page...

Key	Atom-1	Atom-2	Atom-3	Atom-4	Model ID	Violation (°)
(1,131)	1:A:72:MET:C	1:A:73:PRO:N	1:A:73:PRO:CA	1:A:73:PRO:C	5	2.2
(1,74)	1:A:40:TYR:N	1:A:40:TYR:CA	1:A:40:TYR:C	1:A:41:GLU:N	10	2.1
(1,73)	1:A:39:PHE:C	1:A:40:TYR:N	1:A:40:TYR:CA	1:A:40:TYR:C	1	2.1
(1,73)	1:A:39:PHE:C	1:A:40:TYR:N	1:A:40:TYR:CA	1:A:40:TYR:C	7	2.1
(1,45)	1:A:24:ASP:C	1:A:25:PHE:N	1:A:25:PHE:CA	1:A:25:PHE:C	9	2.1
(1,27)	1:A:15:ILE:C	1:A:16:SER:N	1:A:16:SER:CA	1:A:16:SER:C	10	2.1
(1,180)	1:A:101:LYS:N	1:A:101:LYS:CA	1:A:101:LYS:C	1:A:102:TYR:N	7	2.1
(1,177)	1:A:99:ILE:C	1:A:100:GLU:N	1:A:100:GLU:CA	1:A:100:GLU:C	7	2.1
(1,173)	1:A:97:GLN:C	1:A:98:LEU:N	1:A:98:LEU:CA	1:A:98:LEU:C	1	2.1
(1,149)	1:A:84:VAL:C	1:A:85:ASP:N	1:A:85:ASP:CA	1:A:85:ASP:C	2	2.1
(1,12)	1:A:8:GLN:N	1:A:8:GLN:CA	1:A:8:GLN:C	1:A:9:SER:N	3	2.1
(1,74)	1:A:40:TYR:N	1:A:40:TYR:CA	1:A:40:TYR:C	1:A:41:GLU:N	9	2.0
(1,64)	1:A:35:ARG:N	1:A:35:ARG:CA	1:A:35:ARG:C	1:A:36:ILE:N	1	2.0
(1,55)	1:A:30:CYS:C	1:A:31:GLY:N	1:A:31:GLY:CA	1:A:31:GLY:C	8	2.0
(1,30)	1:A:17:GLN:N	1:A:17:GLN:CA	1:A:17:GLN:C	1:A:18:ASN:N	4	2.0
(1,30)	1:A:17:GLN:N	1:A:17:GLN:CA	1:A:17:GLN:C	1:A:18:ASN:N	6	2.0
(1,18)	1:A:11:PHE:N	1:A:11:PHE:CA	1:A:11:PHE:C	1:A:12:ASP:N	5	2.0
(1,18)	1:A:11:PHE:N	1:A:11:PHE:CA	1:A:11:PHE:C	1:A:12:ASP:N	9	2.0
(1,174)	1:A:98:LEU:N	1:A:98:LEU:CA	1:A:98:LEU:C	1:A:99:ILE:N	3	2.0
(1,12)	1:A:8:GLN:N	1:A:8:GLN:CA	1:A:8:GLN:C	1:A:9:SER:N	8	2.0
(1,93)	1:A:50:MET:C	1:A:51:VAL:N	1:A:51:VAL:CA	1:A:51:VAL:C	10	1.9
(1,90)	1:A:48:THR:N	1:A:48:THR:CA	1:A:48:THR:C	1:A:49:LYS:N	2	1.9
(1,74)	1:A:40:TYR:N	1:A:40:TYR:CA	1:A:40:TYR:C	1:A:41:GLU:N	2	1.9
(1,64)	1:A:35:ARG:N	1:A:35:ARG:CA	1:A:35:ARG:C	1:A:36:ILE:N	9	1.9
(1,46)	1:A:25:PHE:N	1:A:25:PHE:CA	1:A:25:PHE:C	1:A:26:PHE:N	10	1.9
(1,45)	1:A:24:ASP:C	1:A:25:PHE:N	1:A:25:PHE:CA	1:A:25:PHE:C	5	1.9
(1,40)	1:A:22:ILE:N	1:A:22:ILE:CA	1:A:22:ILE:C	1:A:23:VAL:N	9	1.9
(1,39)	1:A:21:VAL:C	1:A:22:ILE:N	1:A:22:ILE:CA	1:A:22:ILE:C	2	1.9
(1,39)	1:A:21:VAL:C	1:A:22:ILE:N	1:A:22:ILE:CA	1:A:22:ILE:C	5	1.9
(1,181)	1:A:101:LYS:C	1:A:102:TYR:N	1:A:102:TYR:CA	1:A:102:TYR:C	4	1.9
(1,178)	1:A:100:GLU:N	1:A:100:GLU:CA	1:A:100:GLU:C	1:A:101:LYS:N	7	1.9
(1,175)	1:A:98:LEU:C	1:A:99:ILE:N	1:A:99:ILE:CA	1:A:99:ILE:C	10	1.9
(1,174)	1:A:98:LEU:N	1:A:98:LEU:CA	1:A:98:LEU:C	1:A:99:ILE:N	2	1.9
(1,173)	1:A:97:GLN:C	1:A:98:LEU:N	1:A:98:LEU:CA	1:A:98:LEU:C	4	1.9
(1,169)	1:A:95:LEU:C	1:A:96:LYS:N	1:A:96:LYS:CA	1:A:96:LYS:C	4	1.9
(1,169)	1:A:95:LEU:C	1:A:96:LYS:N	1:A:96:LYS:CA	1:A:96:LYS:C	5	1.9
(1,169)	1:A:95:LEU:C	1:A:96:LYS:N	1:A:96:LYS:CA	1:A:96:LYS:C	8	1.9
(1,64)	1:A:35:ARG:N	1:A:35:ARG:CA	1:A:35:ARG:C	1:A:36:ILE:N	4	1.8
(1,46)	1:A:25:PHE:N	1:A:25:PHE:CA	1:A:25:PHE:C	1:A:26:PHE:N	3	1.8
(1,45)	1:A:24:ASP:C	1:A:25:PHE:N	1:A:25:PHE:CA	1:A:25:PHE:C	3	1.8
(1,45)	1:A:24:ASP:C	1:A:25:PHE:N	1:A:25:PHE:CA	1:A:25:PHE:C	6	1.8
(1,40)	1:A:22:ILE:N	1:A:22:ILE:CA	1:A:22:ILE:C	1:A:23:VAL:N	2	1.8
(1,40)	1:A:22:ILE:N	1:A:22:ILE:CA	1:A:22:ILE:C	1:A:23:VAL:N	3	1.8
(1,30)	1:A:17:GLN:N	1:A:17:GLN:CA	1:A:17:GLN:C	1:A:18:ASN:N	1	1.8
(1,30)	1:A:17:GLN:N	1:A:17:GLN:CA	1:A:17:GLN:C	1:A:18:ASN:N	2	1.8
(1,27)	1:A:15:ILE:C	1:A:16:SER:N	1:A:16:SER:CA	1:A:16:SER:C	5	1.8
(1,181)	1:A:101:LYS:C	1:A:102:TYR:N	1:A:102:TYR:CA	1:A:102:TYR:C	9	1.8
(1,180)	1:A:101:LYS:N	1:A:101:LYS:CA	1:A:101:LYS:C	1:A:102:TYR:N	3	1.8
(1,174)	1:A:98:LEU:N	1:A:98:LEU:CA	1:A:98:LEU:C	1:A:99:ILE:N	4	1.8
(1,173)	1:A:97:GLN:C	1:A:98:LEU:N	1:A:98:LEU:CA	1:A:98:LEU:C	8	1.8
(1,173)	1:A:97:GLN:C	1:A:98:LEU:N	1:A:98:LEU:CA	1:A:98:LEU:C	10	1.8

Continued on next page...

Continued from previous page...

Key	Atom-1	Atom-2	Atom-3	Atom-4	Model ID	Violation (°)
(1,171)	1:A:96:LYS:C	1:A:97:GLN:N	1:A:97:GLN:CA	1:A:97:GLN:C	7	1.8
(1,169)	1:A:95:LEU:C	1:A:96:LYS:N	1:A:96:LYS:CA	1:A:96:LYS:C	1	1.8
(1,131)	1:A:72:MET:C	1:A:73:PRO:N	1:A:73:PRO:CA	1:A:73:PRO:C	1	1.8
(1,12)	1:A:8:GLN:N	1:A:8:GLN:CA	1:A:8:GLN:C	1:A:9:SER:N	2	1.8
(1,10)	1:A:7:SER:N	1:A:7:SER:CA	1:A:7:SER:C	1:A:8:GLN:N	7	1.8
(1,1)	1:A:2:VAL:C	1:A:3:LYS:N	1:A:3:LYS:CA	1:A:3:LYS:C	1	1.8
(1,75)	1:A:40:TYR:C	1:A:41:GLU:N	1:A:41:GLU:CA	1:A:41:GLU:C	5	1.7
(1,75)	1:A:40:TYR:C	1:A:41:GLU:N	1:A:41:GLU:CA	1:A:41:GLU:C	8	1.7
(1,74)	1:A:40:TYR:N	1:A:40:TYR:CA	1:A:40:TYR:C	1:A:41:GLU:N	1	1.7
(1,69)	1:A:37:ALA:C	1:A:38:PRO:N	1:A:38:PRO:CA	1:A:38:PRO:C	1	1.7
(1,69)	1:A:37:ALA:C	1:A:38:PRO:N	1:A:38:PRO:CA	1:A:38:PRO:C	5	1.7
(1,69)	1:A:37:ALA:C	1:A:38:PRO:N	1:A:38:PRO:CA	1:A:38:PRO:C	10	1.7
(1,46)	1:A:25:PHE:N	1:A:25:PHE:CA	1:A:25:PHE:C	1:A:26:PHE:N	2	1.7
(1,45)	1:A:24:ASP:C	1:A:25:PHE:N	1:A:25:PHE:CA	1:A:25:PHE:C	10	1.7
(1,41)	1:A:22:ILE:C	1:A:23:VAL:N	1:A:23:VAL:CA	1:A:23:VAL:C	3	1.7
(1,40)	1:A:22:ILE:N	1:A:22:ILE:CA	1:A:22:ILE:C	1:A:23:VAL:N	1	1.7
(1,40)	1:A:22:ILE:N	1:A:22:ILE:CA	1:A:22:ILE:C	1:A:23:VAL:N	5	1.7
(1,40)	1:A:22:ILE:N	1:A:22:ILE:CA	1:A:22:ILE:C	1:A:23:VAL:N	10	1.7
(1,37)	1:A:20:LEU:C	1:A:21:VAL:N	1:A:21:VAL:CA	1:A:21:VAL:C	1	1.7
(1,30)	1:A:17:GLN:N	1:A:17:GLN:CA	1:A:17:GLN:C	1:A:18:ASN:N	8	1.7
(1,27)	1:A:15:ILE:C	1:A:16:SER:N	1:A:16:SER:CA	1:A:16:SER:C	9	1.7
(1,181)	1:A:101:LYS:C	1:A:102:TYR:N	1:A:102:TYR:CA	1:A:102:TYR:C	8	1.7
(1,174)	1:A:98:LEU:N	1:A:98:LEU:CA	1:A:98:LEU:C	1:A:99:ILE:N	1	1.7
(1,169)	1:A:95:LEU:C	1:A:96:LYS:N	1:A:96:LYS:CA	1:A:96:LYS:C	9	1.7
(1,146)	1:A:80:ASN:N	1:A:80:ASN:CA	1:A:80:ASN:C	1:A:81:GLY:N	5	1.7
(1,12)	1:A:8:GLN:N	1:A:8:GLN:CA	1:A:8:GLN:C	1:A:9:SER:N	6	1.7
(1,93)	1:A:50:MET:C	1:A:51:VAL:N	1:A:51:VAL:CA	1:A:51:VAL:C	4	1.6
(1,69)	1:A:37:ALA:C	1:A:38:PRO:N	1:A:38:PRO:CA	1:A:38:PRO:C	4	1.6
(1,59)	1:A:32:PRO:C	1:A:33:CYS:N	1:A:33:CYS:CA	1:A:33:CYS:C	3	1.6
(1,59)	1:A:32:PRO:C	1:A:33:CYS:N	1:A:33:CYS:CA	1:A:33:CYS:C	10	1.6
(1,46)	1:A:25:PHE:N	1:A:25:PHE:CA	1:A:25:PHE:C	1:A:26:PHE:N	7	1.6
(1,45)	1:A:24:ASP:C	1:A:25:PHE:N	1:A:25:PHE:CA	1:A:25:PHE:C	7	1.6
(1,4)	1:A:4:ILE:N	1:A:4:ILE:CA	1:A:4:ILE:C	1:A:5:VAL:N	4	1.6
(1,30)	1:A:17:GLN:N	1:A:17:GLN:CA	1:A:17:GLN:C	1:A:18:ASN:N	7	1.6
(1,27)	1:A:15:ILE:C	1:A:16:SER:N	1:A:16:SER:CA	1:A:16:SER:C	3	1.6
(1,27)	1:A:15:ILE:C	1:A:16:SER:N	1:A:16:SER:CA	1:A:16:SER:C	6	1.6
(1,27)	1:A:15:ILE:C	1:A:16:SER:N	1:A:16:SER:CA	1:A:16:SER:C	8	1.6
(1,180)	1:A:101:LYS:N	1:A:101:LYS:CA	1:A:101:LYS:C	1:A:102:TYR:N	10	1.6
(1,175)	1:A:98:LEU:C	1:A:99:ILE:N	1:A:99:ILE:CA	1:A:99:ILE:C	5	1.6
(1,173)	1:A:97:GLN:C	1:A:98:LEU:N	1:A:98:LEU:CA	1:A:98:LEU:C	5	1.6
(1,173)	1:A:97:GLN:C	1:A:98:LEU:N	1:A:98:LEU:CA	1:A:98:LEU:C	9	1.6
(1,169)	1:A:95:LEU:C	1:A:96:LYS:N	1:A:96:LYS:CA	1:A:96:LYS:C	10	1.6
(1,146)	1:A:80:ASN:N	1:A:80:ASN:CA	1:A:80:ASN:C	1:A:81:GLY:N	4	1.6
(1,123)	1:A:66:LYS:C	1:A:67:GLU:N	1:A:67:GLU:CA	1:A:67:GLU:C	1	1.6
(1,12)	1:A:8:GLN:N	1:A:8:GLN:CA	1:A:8:GLN:C	1:A:9:SER:N	9	1.6
(1,119)	1:A:64:THR:C	1:A:65:GLU:N	1:A:65:GLU:CA	1:A:65:GLU:C	6	1.6
(1,10)	1:A:7:SER:N	1:A:7:SER:CA	1:A:7:SER:C	1:A:8:GLN:N	3	1.6
(1,1)	1:A:2:VAL:C	1:A:3:LYS:N	1:A:3:LYS:CA	1:A:3:LYS:C	8	1.6
(1,74)	1:A:40:TYR:N	1:A:40:TYR:CA	1:A:40:TYR:C	1:A:41:GLU:N	4	1.5
(1,64)	1:A:35:ARG:N	1:A:35:ARG:CA	1:A:35:ARG:C	1:A:36:ILE:N	5	1.5
(1,64)	1:A:35:ARG:N	1:A:35:ARG:CA	1:A:35:ARG:C	1:A:36:ILE:N	6	1.5

Continued on next page...

Continued from previous page...

Key	Atom-1	Atom-2	Atom-3	Atom-4	Model ID	Violation (°)
(1,64)	1:A:35:ARG:N	1:A:35:ARG:CA	1:A:35:ARG:C	1:A:36:ILE:N	10	1.5
(1,60)	1:A:33:CYS:N	1:A:33:CYS:CA	1:A:33:CYS:C	1:A:34:LYS:N	1	1.5
(1,60)	1:A:33:CYS:N	1:A:33:CYS:CA	1:A:33:CYS:C	1:A:34:LYS:N	4	1.5
(1,46)	1:A:25:PHE:N	1:A:25:PHE:CA	1:A:25:PHE:C	1:A:26:PHE:N	9	1.5
(1,45)	1:A:24:ASP:C	1:A:25:PHE:N	1:A:25:PHE:CA	1:A:25:PHE:C	4	1.5
(1,40)	1:A:22:ILE:N	1:A:22:ILE:CA	1:A:22:ILE:C	1:A:23:VAL:N	4	1.5
(1,39)	1:A:21:VAL:C	1:A:22:ILE:N	1:A:22:ILE:CA	1:A:22:ILE:C	8	1.5
(1,30)	1:A:17:GLN:N	1:A:17:GLN:CA	1:A:17:GLN:C	1:A:18:ASN:N	3	1.5
(1,27)	1:A:15:ILE:C	1:A:16:SER:N	1:A:16:SER:CA	1:A:16:SER:C	4	1.5
(1,2)	1:A:3:LYS:N	1:A:3:LYS:CA	1:A:3:LYS:C	1:A:4:ILE:N	7	1.5
(1,181)	1:A:101:LYS:C	1:A:102:TYR:N	1:A:102:TYR:CA	1:A:102:TYR:C	1	1.5
(1,181)	1:A:101:LYS:C	1:A:102:TYR:N	1:A:102:TYR:CA	1:A:102:TYR:C	2	1.5
(1,18)	1:A:11:PHE:N	1:A:11:PHE:CA	1:A:11:PHE:C	1:A:12:ASP:N	1	1.5
(1,18)	1:A:11:PHE:N	1:A:11:PHE:CA	1:A:11:PHE:C	1:A:12:ASP:N	2	1.5
(1,178)	1:A:100:GLU:N	1:A:100:GLU:CA	1:A:100:GLU:C	1:A:101:LYS:N	6	1.5
(1,175)	1:A:98:LEU:C	1:A:99:ILE:N	1:A:99:ILE:CA	1:A:99:ILE:C	8	1.5
(1,173)	1:A:97:GLN:C	1:A:98:LEU:N	1:A:98:LEU:CA	1:A:98:LEU:C	2	1.5
(1,146)	1:A:80:ASN:N	1:A:80:ASN:CA	1:A:80:ASN:C	1:A:81:GLY:N	6	1.5
(1,146)	1:A:80:ASN:N	1:A:80:ASN:CA	1:A:80:ASN:C	1:A:81:GLY:N	7	1.5
(1,12)	1:A:8:GLN:N	1:A:8:GLN:CA	1:A:8:GLN:C	1:A:9:SER:N	10	1.5
(1,102)	1:A:55:VAL:N	1:A:55:VAL:CA	1:A:55:VAL:C	1:A:56:ASP:N	8	1.5
(1,93)	1:A:50:MET:C	1:A:51:VAL:N	1:A:51:VAL:CA	1:A:51:VAL:C	7	1.4
(1,93)	1:A:50:MET:C	1:A:51:VAL:N	1:A:51:VAL:CA	1:A:51:VAL:C	9	1.4
(1,74)	1:A:40:TYR:N	1:A:40:TYR:CA	1:A:40:TYR:C	1:A:41:GLU:N	8	1.4
(1,69)	1:A:37:ALA:C	1:A:38:PRO:N	1:A:38:PRO:CA	1:A:38:PRO:C	7	1.4
(1,65)	1:A:35:ARG:C	1:A:36:ILE:N	1:A:36:ILE:CA	1:A:36:ILE:C	2	1.4
(1,64)	1:A:35:ARG:N	1:A:35:ARG:CA	1:A:35:ARG:C	1:A:36:ILE:N	3	1.4
(1,64)	1:A:35:ARG:N	1:A:35:ARG:CA	1:A:35:ARG:C	1:A:36:ILE:N	7	1.4
(1,64)	1:A:35:ARG:N	1:A:35:ARG:CA	1:A:35:ARG:C	1:A:36:ILE:N	8	1.4
(1,60)	1:A:33:CYS:N	1:A:33:CYS:CA	1:A:33:CYS:C	1:A:34:LYS:N	3	1.4
(1,60)	1:A:33:CYS:N	1:A:33:CYS:CA	1:A:33:CYS:C	1:A:34:LYS:N	7	1.4
(1,60)	1:A:33:CYS:N	1:A:33:CYS:CA	1:A:33:CYS:C	1:A:34:LYS:N	8	1.4
(1,59)	1:A:32:PRO:C	1:A:33:CYS:N	1:A:33:CYS:CA	1:A:33:CYS:C	6	1.4
(1,49)	1:A:26:PHE:C	1:A:27:ALA:N	1:A:27:ALA:CA	1:A:27:ALA:C	2	1.4
(1,49)	1:A:26:PHE:C	1:A:27:ALA:N	1:A:27:ALA:CA	1:A:27:ALA:C	7	1.4
(1,45)	1:A:24:ASP:C	1:A:25:PHE:N	1:A:25:PHE:CA	1:A:25:PHE:C	2	1.4
(1,39)	1:A:21:VAL:C	1:A:22:ILE:N	1:A:22:ILE:CA	1:A:22:ILE:C	9	1.4
(1,30)	1:A:17:GLN:N	1:A:17:GLN:CA	1:A:17:GLN:C	1:A:18:ASN:N	5	1.4
(1,28)	1:A:16:SER:N	1:A:16:SER:CA	1:A:16:SER:C	1:A:17:GLN:N	5	1.4
(1,27)	1:A:15:ILE:C	1:A:16:SER:N	1:A:16:SER:CA	1:A:16:SER:C	1	1.4
(1,27)	1:A:15:ILE:C	1:A:16:SER:N	1:A:16:SER:CA	1:A:16:SER:C	2	1.4
(1,181)	1:A:101:LYS:C	1:A:102:TYR:N	1:A:102:TYR:CA	1:A:102:TYR:C	6	1.4
(1,18)	1:A:11:PHE:N	1:A:11:PHE:CA	1:A:11:PHE:C	1:A:12:ASP:N	4	1.4
(1,18)	1:A:11:PHE:N	1:A:11:PHE:CA	1:A:11:PHE:C	1:A:12:ASP:N	7	1.4
(1,177)	1:A:99:ILE:C	1:A:100:GLU:N	1:A:100:GLU:CA	1:A:100:GLU:C	6	1.4
(1,175)	1:A:98:LEU:C	1:A:99:ILE:N	1:A:99:ILE:CA	1:A:99:ILE:C	6	1.4
(1,175)	1:A:98:LEU:C	1:A:99:ILE:N	1:A:99:ILE:CA	1:A:99:ILE:C	9	1.4
(1,174)	1:A:98:LEU:N	1:A:98:LEU:CA	1:A:98:LEU:C	1:A:99:ILE:N	5	1.4
(1,174)	1:A:98:LEU:N	1:A:98:LEU:CA	1:A:98:LEU:C	1:A:99:ILE:N	6	1.4
(1,174)	1:A:98:LEU:N	1:A:98:LEU:CA	1:A:98:LEU:C	1:A:99:ILE:N	10	1.4
(1,173)	1:A:97:GLN:C	1:A:98:LEU:N	1:A:98:LEU:CA	1:A:98:LEU:C	7	1.4

Continued on next page...

Continued from previous page...

Key	Atom-1	Atom-2	Atom-3	Atom-4	Model ID	Violation (°)
(1,171)	1:A:96:LYS:C	1:A:97:GLN:N	1:A:97:GLN:CA	1:A:97:GLN:C	2	1.4
(1,171)	1:A:96:LYS:C	1:A:97:GLN:N	1:A:97:GLN:CA	1:A:97:GLN:C	10	1.4
(1,169)	1:A:95:LEU:C	1:A:96:LYS:N	1:A:96:LYS:CA	1:A:96:LYS:C	6	1.4
(1,146)	1:A:80:ASN:N	1:A:80:ASN:CA	1:A:80:ASN:C	1:A:81:GLY:N	2	1.4
(1,146)	1:A:80:ASN:N	1:A:80:ASN:CA	1:A:80:ASN:C	1:A:81:GLY:N	3	1.4
(1,146)	1:A:80:ASN:N	1:A:80:ASN:CA	1:A:80:ASN:C	1:A:81:GLY:N	10	1.4
(1,12)	1:A:8:GLN:N	1:A:8:GLN:CA	1:A:8:GLN:C	1:A:9:SER:N	4	1.4
(1,12)	1:A:8:GLN:N	1:A:8:GLN:CA	1:A:8:GLN:C	1:A:9:SER:N	5	1.4
(1,10)	1:A:7:SER:N	1:A:7:SER:CA	1:A:7:SER:C	1:A:8:GLN:N	2	1.4
(1,1)	1:A:2:VAL:C	1:A:3:LYS:N	1:A:3:LYS:CA	1:A:3:LYS:C	2	1.4
(1,1)	1:A:2:VAL:C	1:A:3:LYS:N	1:A:3:LYS:CA	1:A:3:LYS:C	5	1.4
(1,1)	1:A:2:VAL:C	1:A:3:LYS:N	1:A:3:LYS:CA	1:A:3:LYS:C	9	1.4
(1,93)	1:A:50:MET:C	1:A:51:VAL:N	1:A:51:VAL:CA	1:A:51:VAL:C	6	1.3
(1,90)	1:A:48:THR:N	1:A:48:THR:CA	1:A:48:THR:C	1:A:49:LYS:N	1	1.3
(1,90)	1:A:48:THR:N	1:A:48:THR:CA	1:A:48:THR:C	1:A:49:LYS:N	3	1.3
(1,90)	1:A:48:THR:N	1:A:48:THR:CA	1:A:48:THR:C	1:A:49:LYS:N	10	1.3
(1,69)	1:A:37:ALA:C	1:A:38:PRO:N	1:A:38:PRO:CA	1:A:38:PRO:C	2	1.3
(1,69)	1:A:37:ALA:C	1:A:38:PRO:N	1:A:38:PRO:CA	1:A:38:PRO:C	3	1.3
(1,60)	1:A:33:CYS:N	1:A:33:CYS:CA	1:A:33:CYS:C	1:A:34:LYS:N	9	1.3
(1,59)	1:A:32:PRO:C	1:A:33:CYS:N	1:A:33:CYS:CA	1:A:33:CYS:C	7	1.3
(1,56)	1:A:31:GLY:N	1:A:31:GLY:CA	1:A:31:GLY:C	1:A:32:PRO:N	6	1.3
(1,49)	1:A:26:PHE:C	1:A:27:ALA:N	1:A:27:ALA:CA	1:A:27:ALA:C	9	1.3
(1,41)	1:A:22:ILE:C	1:A:23:VAL:N	1:A:23:VAL:CA	1:A:23:VAL:C	5	1.3
(1,41)	1:A:22:ILE:C	1:A:23:VAL:N	1:A:23:VAL:CA	1:A:23:VAL:C	10	1.3
(1,40)	1:A:22:ILE:N	1:A:22:ILE:CA	1:A:22:ILE:C	1:A:23:VAL:N	6	1.3
(1,36)	1:A:20:LEU:N	1:A:20:LEU:CA	1:A:20:LEU:C	1:A:21:VAL:N	2	1.3
(1,36)	1:A:20:LEU:N	1:A:20:LEU:CA	1:A:20:LEU:C	1:A:21:VAL:N	4	1.3
(1,35)	1:A:19:GLU:C	1:A:20:LEU:N	1:A:20:LEU:CA	1:A:20:LEU:C	10	1.3
(1,18)	1:A:11:PHE:N	1:A:11:PHE:CA	1:A:11:PHE:C	1:A:12:ASP:N	6	1.3
(1,18)	1:A:11:PHE:N	1:A:11:PHE:CA	1:A:11:PHE:C	1:A:12:ASP:N	8	1.3
(1,18)	1:A:11:PHE:N	1:A:11:PHE:CA	1:A:11:PHE:C	1:A:12:ASP:N	10	1.3
(1,177)	1:A:99:ILE:C	1:A:100:GLU:N	1:A:100:GLU:CA	1:A:100:GLU:C	3	1.3
(1,177)	1:A:99:ILE:C	1:A:100:GLU:N	1:A:100:GLU:CA	1:A:100:GLU:C	8	1.3
(1,173)	1:A:97:GLN:C	1:A:98:LEU:N	1:A:98:LEU:CA	1:A:98:LEU:C	3	1.3
(1,171)	1:A:96:LYS:C	1:A:97:GLN:N	1:A:97:GLN:CA	1:A:97:GLN:C	1	1.3
(1,149)	1:A:84:VAL:C	1:A:85:ASP:N	1:A:85:ASP:CA	1:A:85:ASP:C	8	1.3
(1,146)	1:A:80:ASN:N	1:A:80:ASN:CA	1:A:80:ASN:C	1:A:81:GLY:N	9	1.3
(1,12)	1:A:8:GLN:N	1:A:8:GLN:CA	1:A:8:GLN:C	1:A:9:SER:N	1	1.3
(1,10)	1:A:7:SER:N	1:A:7:SER:CA	1:A:7:SER:C	1:A:8:GLN:N	6	1.3
(1,93)	1:A:50:MET:C	1:A:51:VAL:N	1:A:51:VAL:CA	1:A:51:VAL:C	2	1.2
(1,93)	1:A:50:MET:C	1:A:51:VAL:N	1:A:51:VAL:CA	1:A:51:VAL:C	3	1.2
(1,90)	1:A:48:THR:N	1:A:48:THR:CA	1:A:48:THR:C	1:A:49:LYS:N	4	1.2
(1,80)	1:A:43:CYS:N	1:A:43:CYS:CA	1:A:43:CYS:C	1:A:44:SER:N	6	1.2
(1,75)	1:A:40:TYR:C	1:A:41:GLU:N	1:A:41:GLU:CA	1:A:41:GLU:C	7	1.2
(1,69)	1:A:37:ALA:C	1:A:38:PRO:N	1:A:38:PRO:CA	1:A:38:PRO:C	9	1.2
(1,60)	1:A:33:CYS:N	1:A:33:CYS:CA	1:A:33:CYS:C	1:A:34:LYS:N	2	1.2
(1,59)	1:A:32:PRO:C	1:A:33:CYS:N	1:A:33:CYS:CA	1:A:33:CYS:C	8	1.2
(1,56)	1:A:31:GLY:N	1:A:31:GLY:CA	1:A:31:GLY:C	1:A:32:PRO:N	3	1.2
(1,56)	1:A:31:GLY:N	1:A:31:GLY:CA	1:A:31:GLY:C	1:A:32:PRO:N	10	1.2
(1,36)	1:A:20:LEU:N	1:A:20:LEU:CA	1:A:20:LEU:C	1:A:21:VAL:N	7	1.2
(1,30)	1:A:17:GLN:N	1:A:17:GLN:CA	1:A:17:GLN:C	1:A:18:ASN:N	9	1.2

Continued on next page...

Continued from previous page...

Key	Atom-1	Atom-2	Atom-3	Atom-4	Model ID	Violation (°)
(1,29)	1:A:16:SER:C	1:A:17:GLN:N	1:A:17:GLN:CA	1:A:17:GLN:C	4	1.2
(1,29)	1:A:16:SER:C	1:A:17:GLN:N	1:A:17:GLN:CA	1:A:17:GLN:C	5	1.2
(1,28)	1:A:16:SER:N	1:A:16:SER:CA	1:A:16:SER:C	1:A:17:GLN:N	3	1.2
(1,28)	1:A:16:SER:N	1:A:16:SER:CA	1:A:16:SER:C	1:A:17:GLN:N	4	1.2
(1,24)	1:A:14:ILE:N	1:A:14:ILE:CA	1:A:14:ILE:C	1:A:15:ILE:N	7	1.2
(1,2)	1:A:3:LYS:N	1:A:3:LYS:CA	1:A:3:LYS:C	1:A:4:ILE:N	5	1.2
(1,2)	1:A:3:LYS:N	1:A:3:LYS:CA	1:A:3:LYS:C	1:A:4:ILE:N	8	1.2
(1,18)	1:A:11:PHE:N	1:A:11:PHE:CA	1:A:11:PHE:C	1:A:12:ASP:N	3	1.2
(1,178)	1:A:100:GLU:N	1:A:100:GLU:CA	1:A:100:GLU:C	1:A:101:LYS:N	3	1.2
(1,175)	1:A:98:LEU:C	1:A:99:ILE:N	1:A:99:ILE:CA	1:A:99:ILE:C	2	1.2
(1,175)	1:A:98:LEU:C	1:A:99:ILE:N	1:A:99:ILE:CA	1:A:99:ILE:C	4	1.2
(1,174)	1:A:98:LEU:N	1:A:98:LEU:CA	1:A:98:LEU:C	1:A:99:ILE:N	8	1.2
(1,174)	1:A:98:LEU:N	1:A:98:LEU:CA	1:A:98:LEU:C	1:A:99:ILE:N	9	1.2
(1,169)	1:A:95:LEU:C	1:A:96:LYS:N	1:A:96:LYS:CA	1:A:96:LYS:C	3	1.2
(1,164)	1:A:93:SER:N	1:A:93:SER:CA	1:A:93:SER:C	1:A:94:ALA:N	1	1.2
(1,119)	1:A:64:THR:C	1:A:65:GLU:N	1:A:65:GLU:CA	1:A:65:GLU:C	8	1.2
(1,1)	1:A:2:VAL:C	1:A:3:LYS:N	1:A:3:LYS:CA	1:A:3:LYS:C	7	1.2
(1,80)	1:A:43:CYS:N	1:A:43:CYS:CA	1:A:43:CYS:C	1:A:44:SER:N	8	1.1
(1,74)	1:A:40:TYR:N	1:A:40:TYR:CA	1:A:40:TYR:C	1:A:41:GLU:N	5	1.1
(1,60)	1:A:33:CYS:N	1:A:33:CYS:CA	1:A:33:CYS:C	1:A:34:LYS:N	6	1.1
(1,60)	1:A:33:CYS:N	1:A:33:CYS:CA	1:A:33:CYS:C	1:A:34:LYS:N	10	1.1
(1,59)	1:A:32:PRO:C	1:A:33:CYS:N	1:A:33:CYS:CA	1:A:33:CYS:C	1	1.1
(1,59)	1:A:32:PRO:C	1:A:33:CYS:N	1:A:33:CYS:CA	1:A:33:CYS:C	2	1.1
(1,56)	1:A:31:GLY:N	1:A:31:GLY:CA	1:A:31:GLY:C	1:A:32:PRO:N	8	1.1
(1,5)	1:A:4:ILE:C	1:A:5:VAL:N	1:A:5:VAL:CA	1:A:5:VAL:C	4	1.1
(1,37)	1:A:20:LEU:C	1:A:21:VAL:N	1:A:21:VAL:CA	1:A:21:VAL:C	4	1.1
(1,29)	1:A:16:SER:C	1:A:17:GLN:N	1:A:17:GLN:CA	1:A:17:GLN:C	3	1.1
(1,181)	1:A:101:LYS:C	1:A:102:TYR:N	1:A:102:TYR:CA	1:A:102:TYR:C	3	1.1
(1,175)	1:A:98:LEU:C	1:A:99:ILE:N	1:A:99:ILE:CA	1:A:99:ILE:C	1	1.1
(1,171)	1:A:96:LYS:C	1:A:97:GLN:N	1:A:97:GLN:CA	1:A:97:GLN:C	9	1.1
(1,169)	1:A:95:LEU:C	1:A:96:LYS:N	1:A:96:LYS:CA	1:A:96:LYS:C	7	1.1
(1,164)	1:A:93:SER:N	1:A:93:SER:CA	1:A:93:SER:C	1:A:94:ALA:N	3	1.1
(1,164)	1:A:93:SER:N	1:A:93:SER:CA	1:A:93:SER:C	1:A:94:ALA:N	10	1.1
(1,149)	1:A:84:VAL:C	1:A:85:ASP:N	1:A:85:ASP:CA	1:A:85:ASP:C	3	1.1
(1,149)	1:A:84:VAL:C	1:A:85:ASP:N	1:A:85:ASP:CA	1:A:85:ASP:C	4	1.1
(1,146)	1:A:80:ASN:N	1:A:80:ASN:CA	1:A:80:ASN:C	1:A:81:GLY:N	1	1.1
(1,130)	1:A:72:MET:N	1:A:72:MET:CA	1:A:72:MET:C	1:A:73:PRO:N	3	1.1
(1,129)	1:A:71:SER:C	1:A:72:MET:N	1:A:72:MET:CA	1:A:72:MET:C	10	1.1
(1,127)	1:A:69:ILE:C	1:A:70:THR:N	1:A:70:THR:CA	1:A:70:THR:C	5	1.1
(1,11)	1:A:7:SER:C	1:A:8:GLN:N	1:A:8:GLN:CA	1:A:8:GLN:C	7	1.1
(1,102)	1:A:55:VAL:N	1:A:55:VAL:CA	1:A:55:VAL:C	1:A:56:ASP:N	7	1.1
(1,10)	1:A:7:SER:N	1:A:7:SER:CA	1:A:7:SER:C	1:A:8:GLN:N	1	1.1
(1,10)	1:A:7:SER:N	1:A:7:SER:CA	1:A:7:SER:C	1:A:8:GLN:N	8	1.1
(1,1)	1:A:2:VAL:C	1:A:3:LYS:N	1:A:3:LYS:CA	1:A:3:LYS:C	10	1.1
