# Supplementary material for: Solution structure and pressure response of thioredoxin-1 of Plasmodium falciparum
Source: PLoS One. 2024 Apr 18;19(4):e0301579. doi: 10.1371/journal.pone.0301579 (PMC11025842; doi:10.1371/journal.pone.0301579)
Supplement: S3 File — (PDF) [file pone.0301579.s003.pdf]

- ***Pf*Trx – reduced state**

**Transitions** i - j	K_{ij} (at 0.1 MPa)	ΔG^0_{ij} [kJ mol^{-1}]	ΔV^0_{ij} [mL mol^{-1}]	$\Delta \beta^{0'}_{ij}$ [mL MPa^{-1} mol^{-1}]
1 – 2		2.81 $\pm$ 0.21	-28.69 $\pm$ 0.99	-0.0488 $\pm$ 0.0085
1 – 3		5.87 ± 0.52	-63.0 ± 2.7	-0.022 ± 0.034

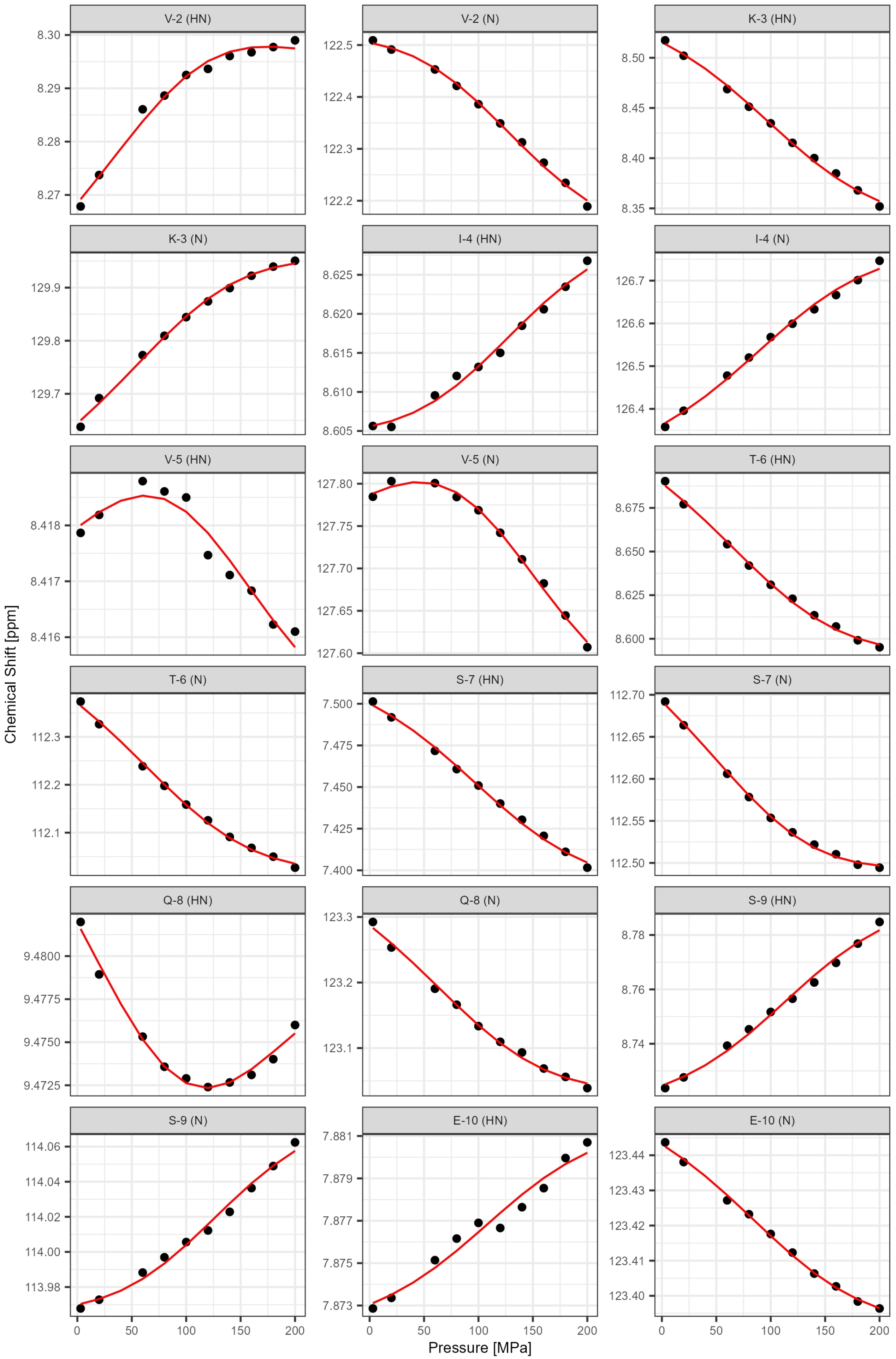
V-2 (HN)
V-2 (N)
K-3 (HN)
K-3 (N)
I-4 (HN)
I-4 (N)
V-5 (HN)
V-5 (N)
T-6 (HN)
T-6 (N)
S-7 (HN)
S-7 (N)
Q-8 (HN)
Q-8 (N)
S-9 (HN)
S-9 (N)
E-10 (HN)
E-10 (N)
Chemical Shift [ppm]
Pressure [MPa]

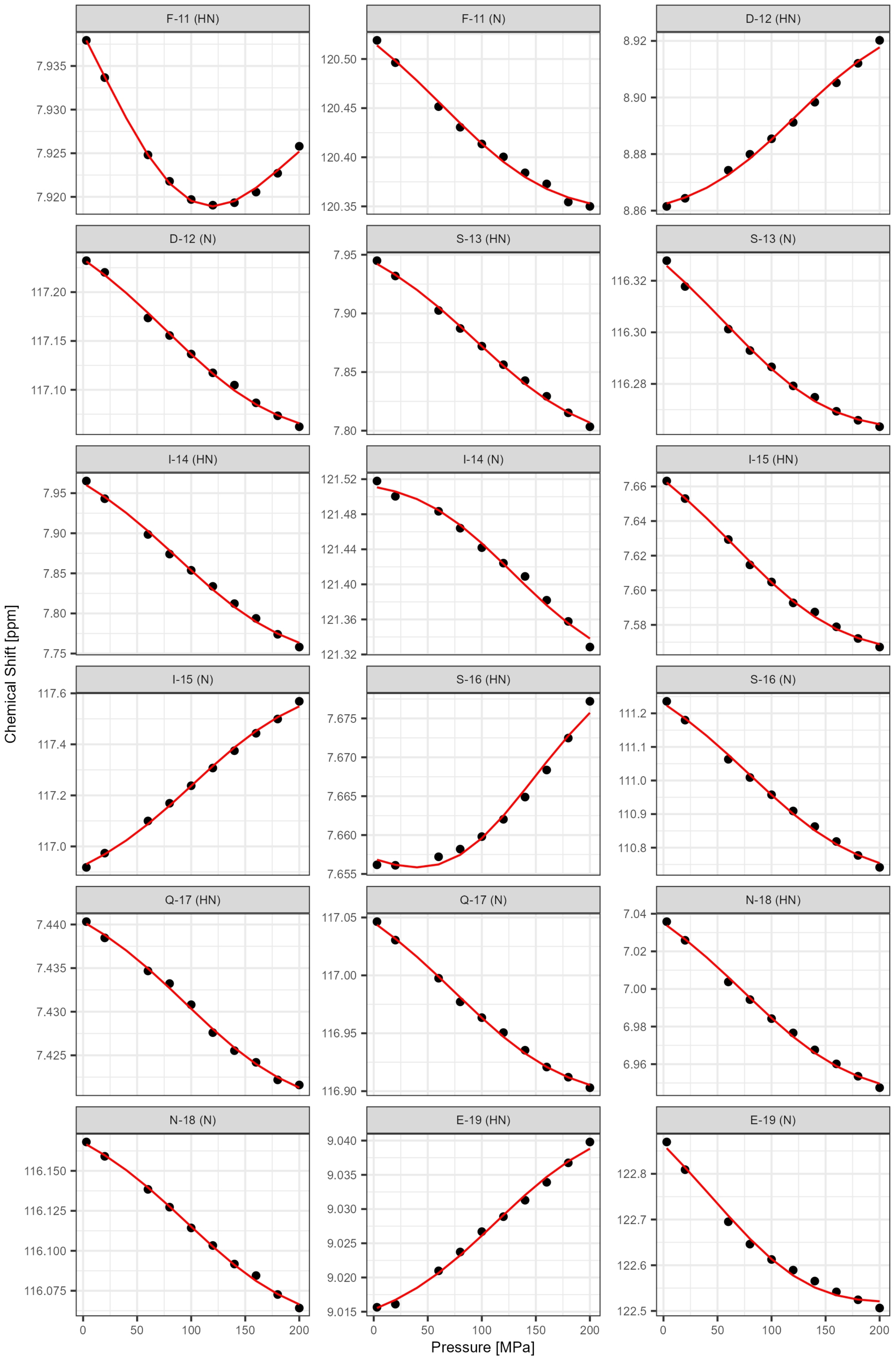
F-11 (HN)
F-11 (N)
D-12 (HN)
D-12 (N)
S-13 (HN)
S-13 (N)
I-14 (HN)
I-14 (N)
I-15 (HN)
I-15 (N)
S-16 (HN)
S-16 (N)
Q-17 (HN)
Q-17 (N)
N-18 (HN)
N-18 (N)
E-19 (HN)
E-19 (N)
Chemical Shift [ppm]
Pressure [MPa]

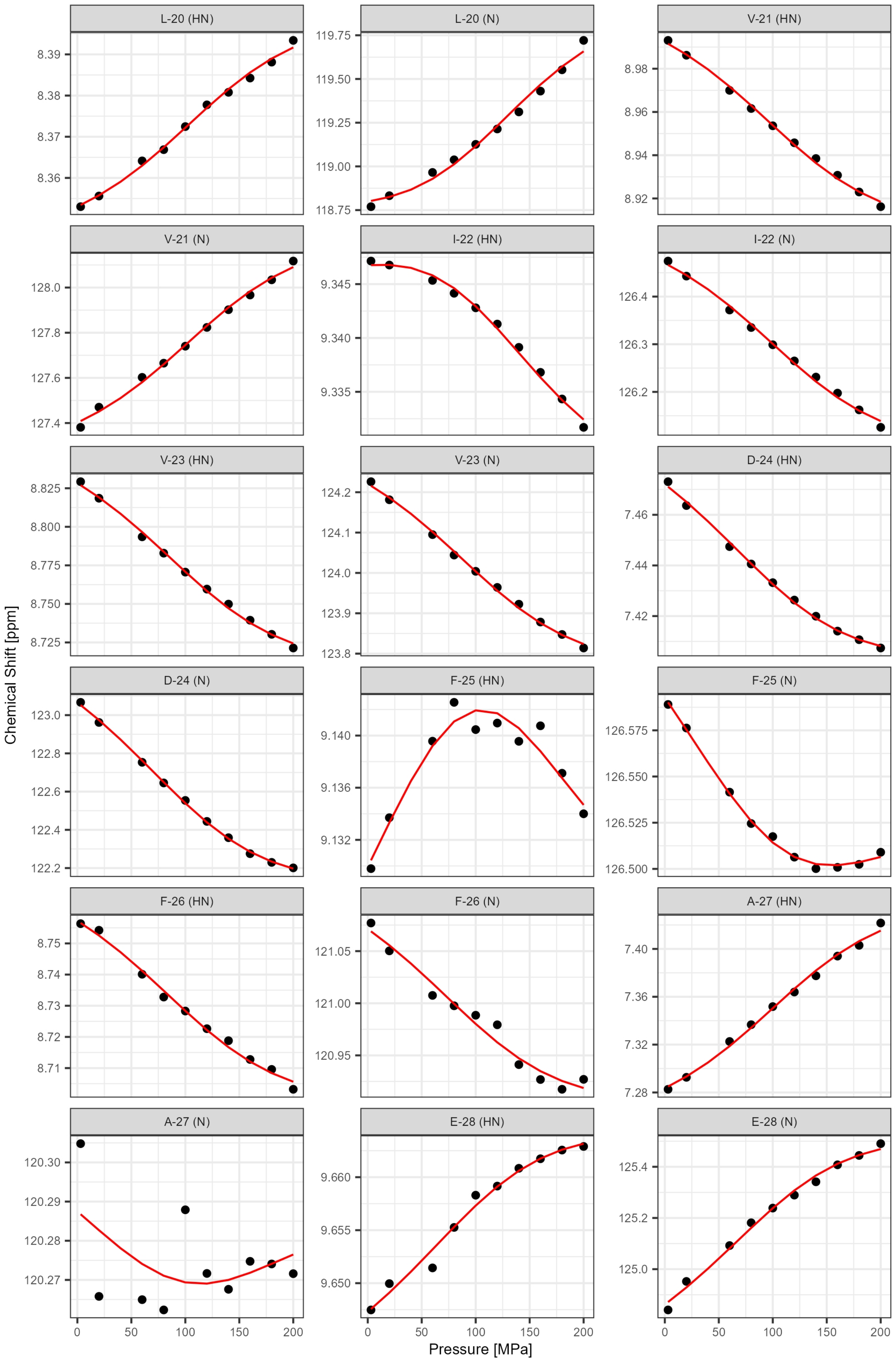
L-20 (HN)
L-20 (N)
V-21 (HN)
V-21 (N)
I-22 (HN)
I-22 (N)
V-23 (HN)
V-23 (N)
D-24 (HN)
D-24 (N)
F-25 (HN)
F-25 (N)
F-26 (HN)
F-26 (N)
A-27 (HN)
A-27 (N)
E-28 (HN)
E-28 (N)
Chemical Shift [ppm]
Pressure [MPa]
0
50
100
150
200

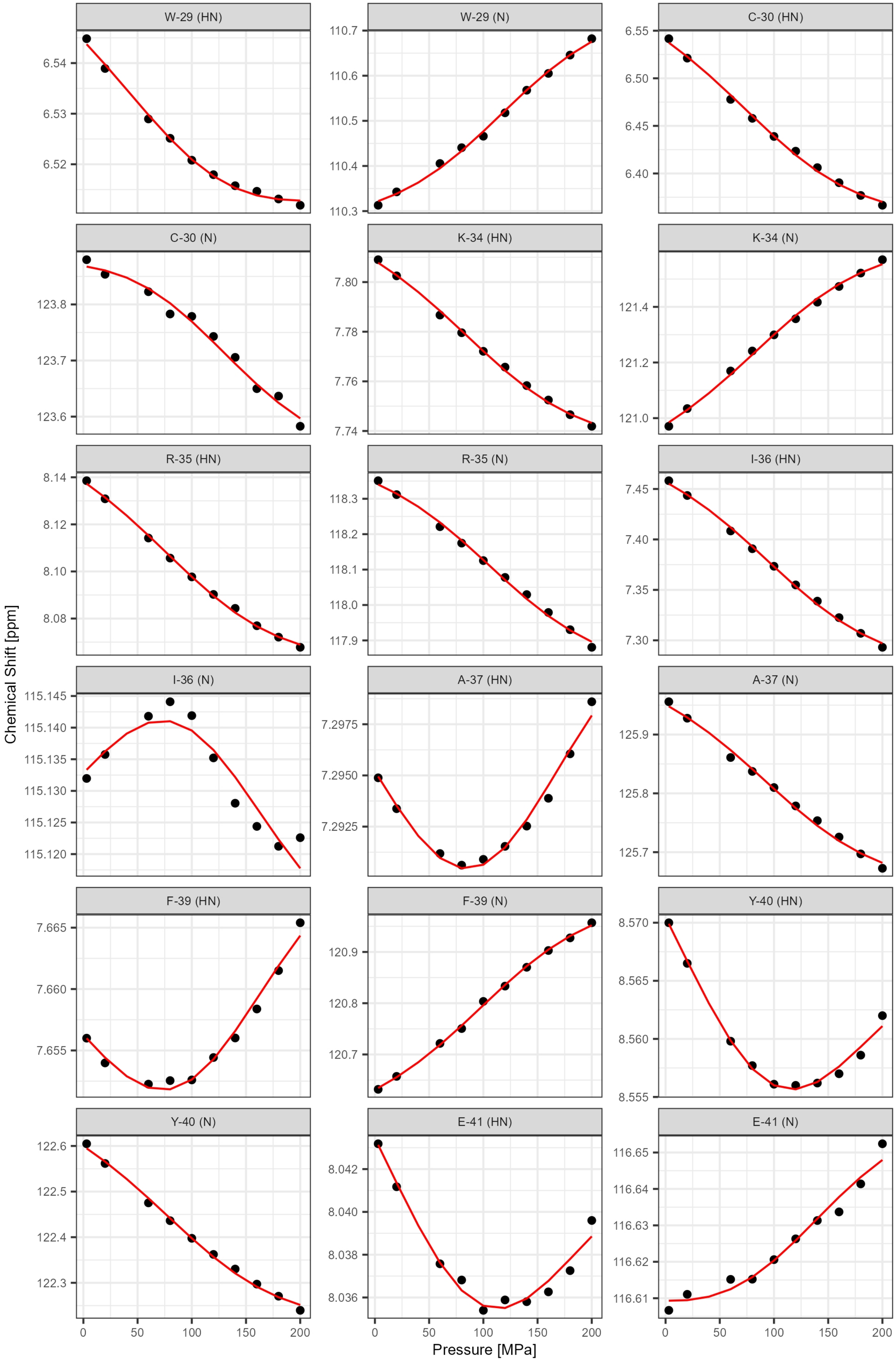
W-29 (HN)
W-29 (N)
C-30 (HN)
C-30 (N)
K-34 (HN)
K-34 (N)
R-35 (HN)
R-35 (N)
I-36 (HN)
I-36 (N)
A-37 (HN)
A-37 (N)
F-39 (HN)
F-39 (N)
Y-40 (HN)
Y-40 (N)
E-41 (HN)
E-41 (N)
Chemical Shift [ppm]
Pressure [MPa]

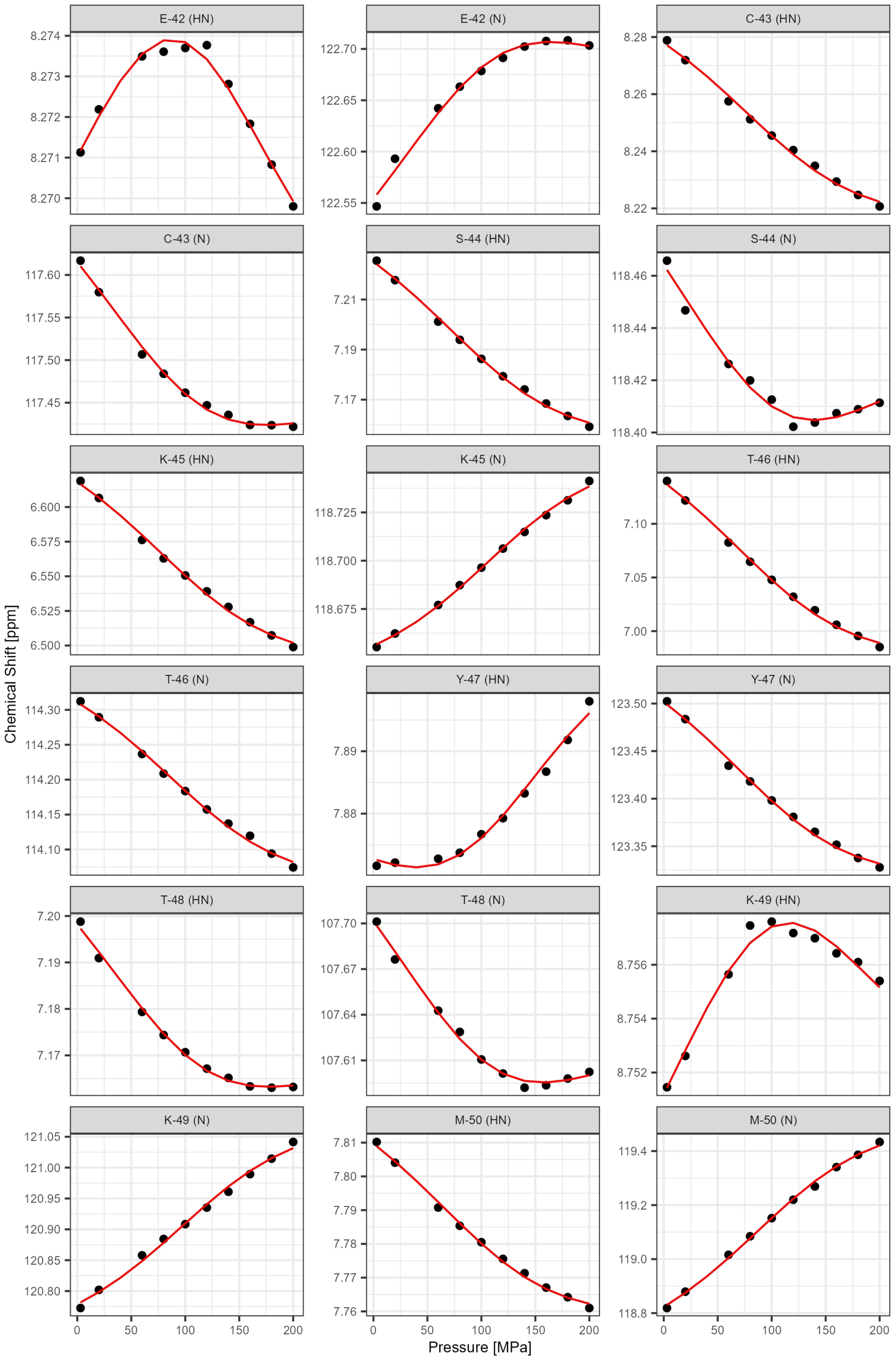
E-42 (HN)
E-42 (N)
C-43 (HN)
C-43 (N)
S-44 (HN)
S-44 (N)
K-45 (HN)
K-45 (N)
T-46 (HN)
T-46 (N)
Y-47 (HN)
Y-47 (N)
T-48 (HN)
T-48 (N)
K-49 (HN)
K-49 (N)
M-50 (HN)
M-50 (N)
Chemical Shift [ppm]
Pressure [MPa]

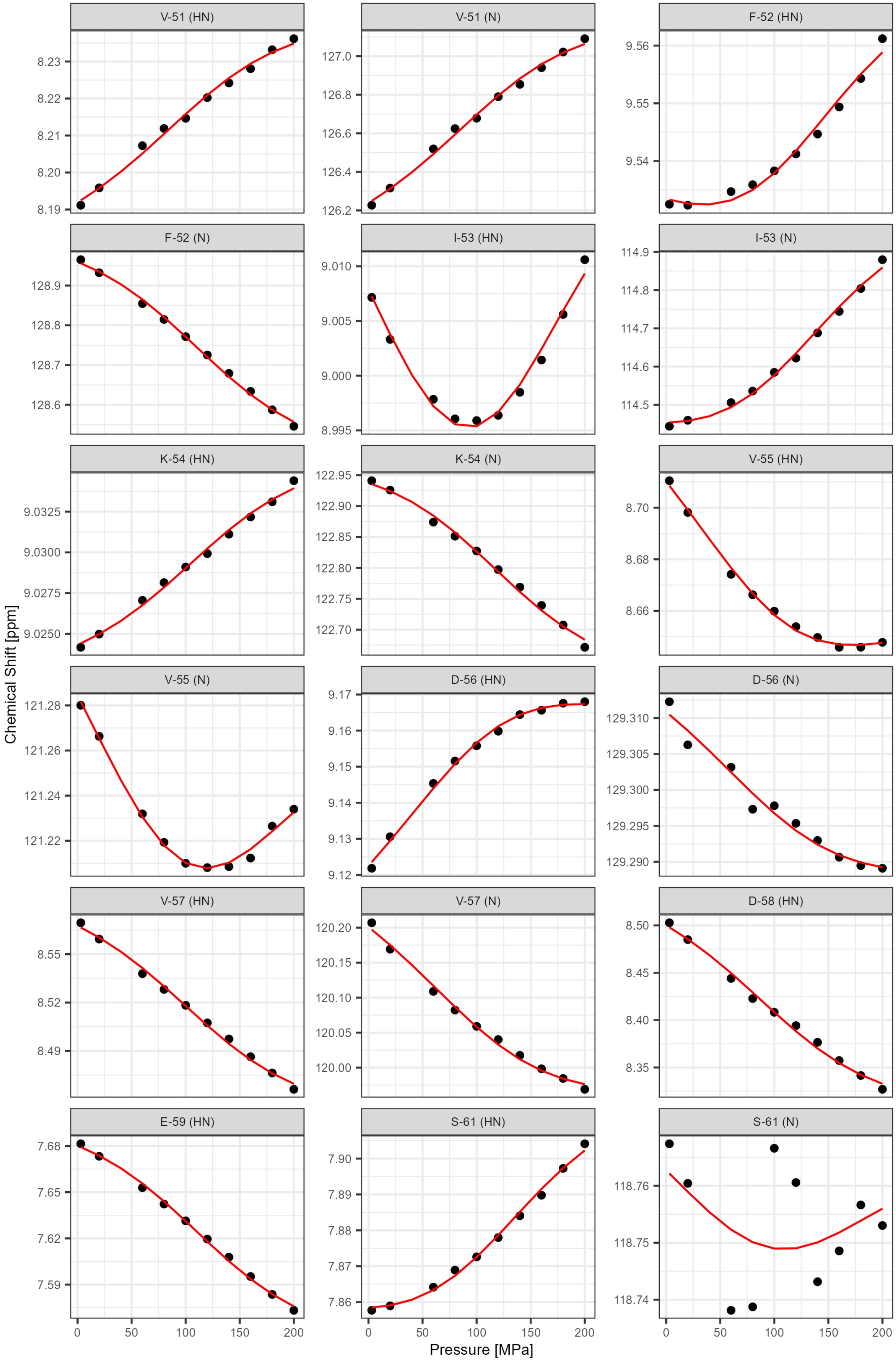

V-51 (HN)
V-51 (N)
F-52 (HN)
F-52 (N)
I-53 (HN)
I-53 (N)
K-54 (HN)
K-54 (N)
V-55 (HN)
V-55 (N)
D-56 (HN)
D-56 (N)
V-57 (HN)
V-57 (N)
D-58 (HN)
E-59 (HN)
S-61 (HN)
S-61 (N)
Chemical Shift [ppm]
Pressure [MPa]

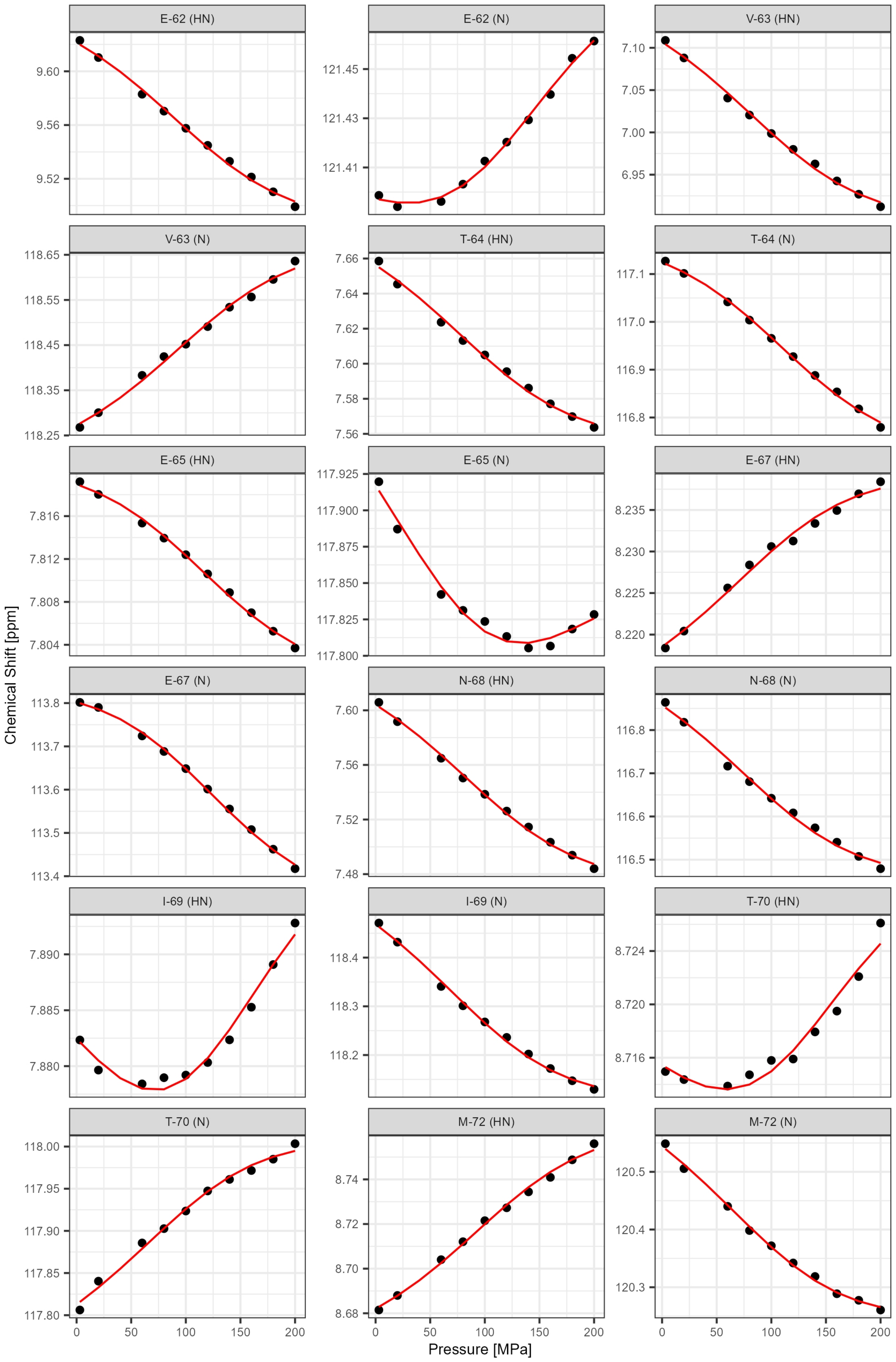

E-62 (HN)
E-62 (N)
V-63 (HN)
V-63 (N)
T-64 (HN)
T-64 (N)
E-65 (HN)
E-65 (N)
E-67 (HN)
E-67 (N)
N-68 (HN)
N-68 (N)
I-69 (HN)
I-69 (N)
T-70 (HN)
T-70 (N)
M-72 (HN)
M-72 (N)
Chemical Shift [ppm]
Pressure [MPa]

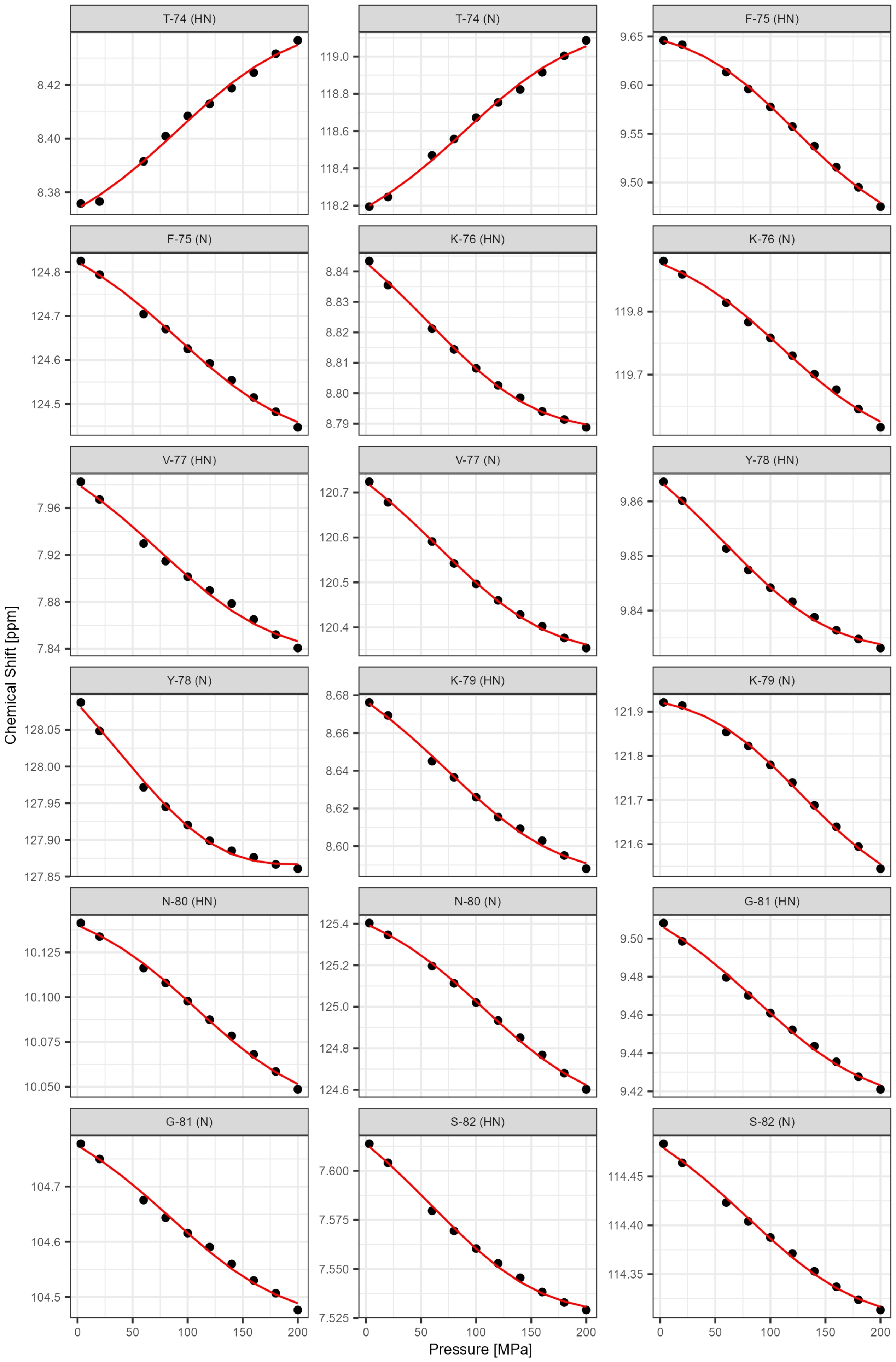

T-74 (HN)
T-74 (N)
F-75 (HN)
F-75 (N)
K-76 (HN)
K-76 (N)
V-77 (HN)
V-77 (N)
Y-78 (HN)
Y-78 (N)
K-79 (HN)
K-79 (N)
N-80 (HN)
N-80 (N)
G-81 (HN)
G-81 (N)
S-82 (HN)
S-82 (N)
Chemical Shift [ppm]
Pressure [MPa]
0
50
100
150
200

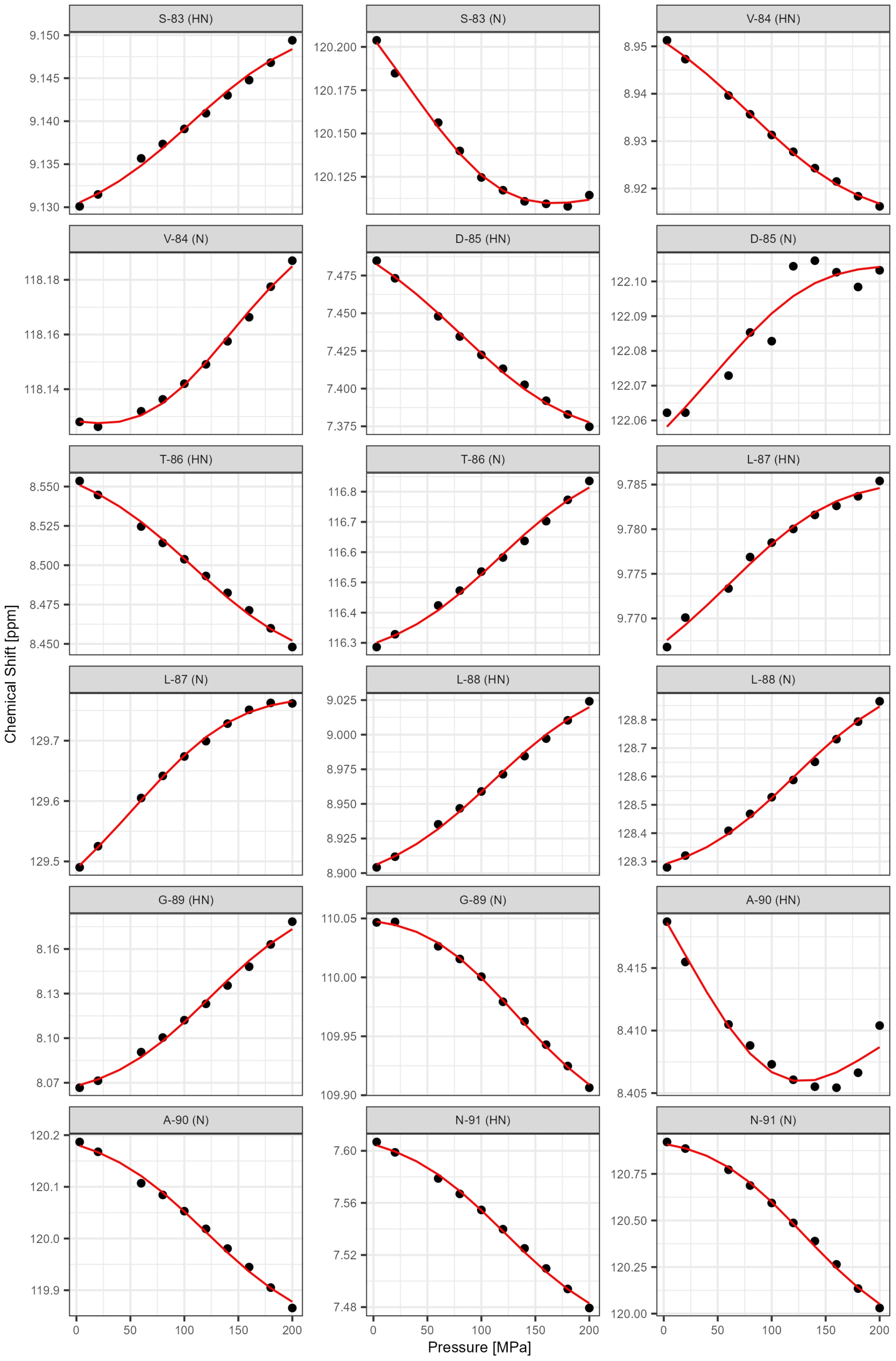

S-83 (HN)
S-83 (N)
V-84 (HN)
V-84 (N)
D-85 (HN)
D-85 (N)
T-86 (HN)
T-86 (N)
L-87 (HN)
L-87 (N)
L-88 (HN)
L-88 (N)
G-89 (HN)
G-89 (N)
A-90 (HN)
A-90 (N)
N-91 (HN)
N-91 (N)
Chemical Shift [ppm]
Pressure [MPa]

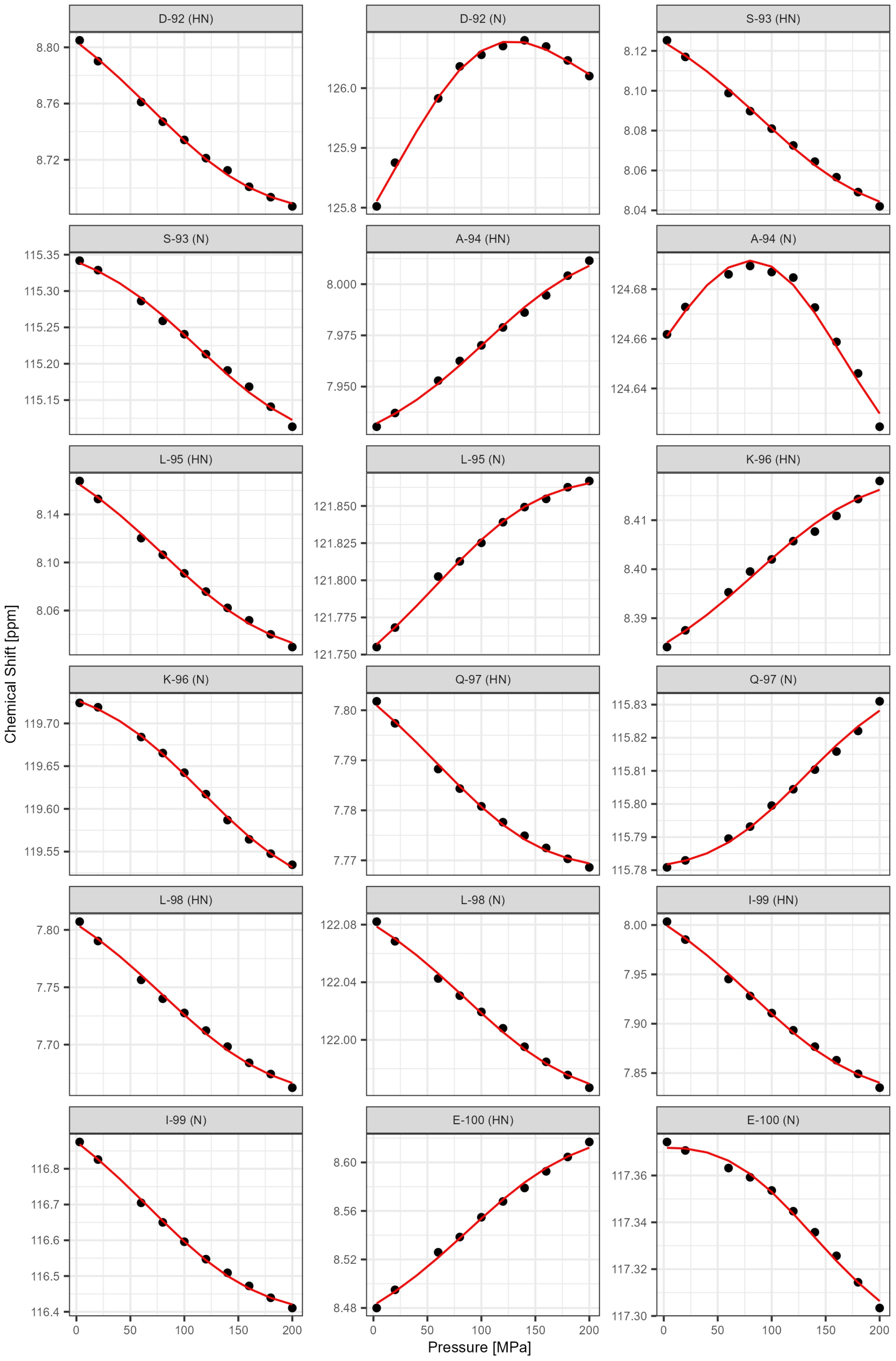
D-92 (HN)
D-92 (N)
S-93 (HN)
S-93 (N)
A-94 (HN)
A-94 (N)
L-95 (HN)
L-95 (N)
K-96 (HN)
K-96 (N)
Q-97 (HN)
Q-97 (N)
L-98 (HN)
L-98 (N)
I-99 (HN)
I-99 (N)
E-100 (HN)
E-100 (N)
Chemical Shift [ppm]
Pressure [MPa]

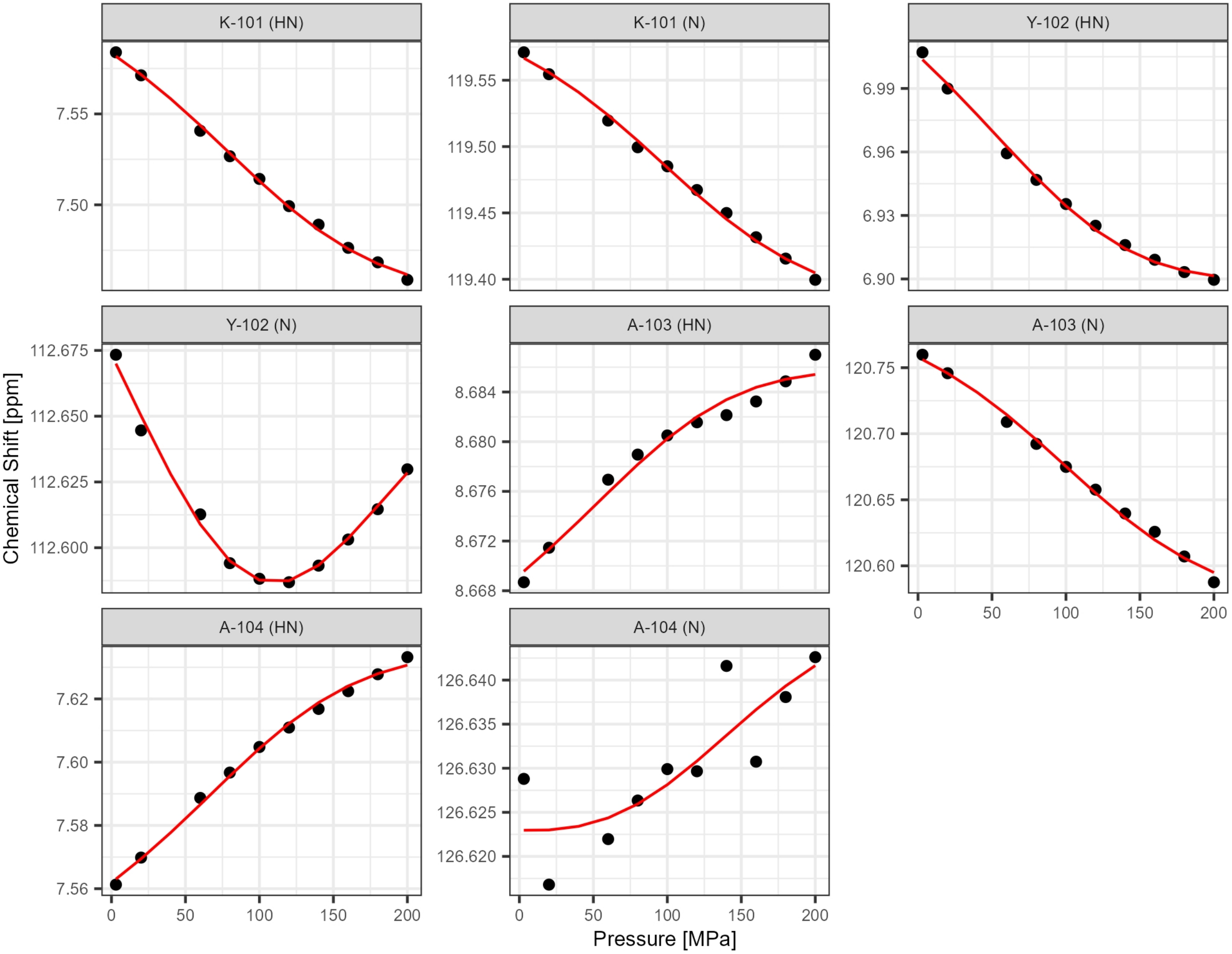
K-101 (HN)
K-101 (N)
Y-102 (HN)
Y-102 (N)
A-103 (HN)
A-103 (N)
A-104 (HN)
A-104 (N)
Chemical Shift [ppm]
Pressure [MPa]
7.55
7.50
119.55
119.50
119.45
119.40
6.99
6.96
6.93
6.90
112.675
112.650
112.625
112.600
8.684
8.680
8.676
8.672
8.668
120.75
120.70
120.65
120.60
7.62
7.60
7.58
7.56
126.640
126.635
126.630
126.625
126.620
0
50
100
150
200

nuc	res	res_nr	d1	d1err	d2	d2err	d3	d3err	converged
HN	V	2	8.242188	0.003343	8.344523	0.007286	8.293735	0.001985	TRUE
N	V	2	122.5084	0.016658	122.6044	0.036306	122.1089	0.009891	TRUE
HN	K	3	8.560135	0.008924	8.426298	0.01945	8.330054	0.005299	TRUE
N	K	3	129.4645	0.018496	130.1276	0.040313	129.9514	0.010983	TRUE
HN	I	4	8.605266	0.002153	8.599166	0.004691	8.631721	0.001278	TRUE
N	I	4	126.2388	0.028125	126.6355	0.061299	126.7806	0.0167	TRUE
HN	V	5	8.415768	0.000643	8.426006	0.001401	8.414107	0.000382	TRUE
N	V	5	127.6893	0.012345	128.1737	0.026905	127.5127	0.00733	TRUE
HN	T	6	8.736086	0.004743	8.568521	0.010337	8.590869	0.002816	TRUE
N	T	6	112.5469	0.015905	111.9109	0.034665	112.0173	0.009444	TRUE
HN	S	7	7.527674	0.005374	7.44412	0.011713	7.38867	0.003191	TRUE
N	S	7	112.8221	0.007691	112.3352	0.016762	112.4994	0.004567	TRUE
HN	Q	8	9.495934	0.000996	9.438046	0.002171	9.480282	0.000591	TRUE
N	Q	8	123.4131	0.016246	122.9607	0.035408	123.0325	0.009647	TRUE
HN	S	9	8.715059	0.005141	8.734831	0.011204	8.794732	0.003052	TRUE
N	S	9	113.9674	0.009314	113.946	0.0203	114.0831	0.005531	TRUE
HN	E	10	7.871548	0.001355	7.875347	0.002954	7.881683	0.000805	TRUE
N	E	10	123.4605	0.002241	123.4028	0.004883	123.3905	0.00133	TRUE
HN	F	11	7.96718	0.000857	7.849358	0.001868	7.934806	0.000509	TRUE
N	F	11	120.6007	0.012081	120.2991	0.02633	120.3434	0.007173	TRUE
HN	D	12	8.857787	0.003991	8.856192	0.008698	8.93263	0.00237	TRUE
N	D	12	117.3037	0.008564	117.0647	0.018666	117.0478	0.005085	TRUE
HN	S	13	7.985433	0.00651	7.856709	0.014188	7.784929	0.003865	TRUE
N	S	13	116.3627	0.00328	116.2316	0.007148	116.2624	0.001948	TRUE
HN	I	14	8.032082	0.01136	7.80606	0.024759	7.735902	0.006745	TRUE
N	I	14	121.511	0.017307	121.577	0.037721	121.2849	0.010277	TRUE
HN	I	15	7.711921	0.003878	7.539892	0.008453	7.562851	0.002303	TRUE
N	I	15	116.7641	0.031403	117.2229	0.068444	117.6633	0.018647	TRUE
HN	S	16	7.665841	0.002366	7.62067	0.005158	7.685745	0.001405	TRUE
N	S	16	111.4219	0.027013	110.7707	0.058875	110.7007	0.01604	TRUE
HN	Q	17	7.446104	0.001025	7.428198	0.002233	7.418246	0.000608	TRUE
N	Q	17	117.1061	0.006681	116.8978	0.014562	116.8914	0.003967	TRUE
HN	N	18	7.072666	0.004583	6.942349	0.009988	6.941563	0.002721	TRUE
N	N	18	116.1975	0.004346	116.1065	0.009472	116.0498	0.00258	TRUE
HN	E	19	9.011326	0.001682	9.019962	0.003666	9.044076	0.000999	TRUE
N	E	19	123.1102	0.031003	122.1739	0.067572	122.535	0.018409	TRUE
HN	L	20	8.34356	0.002713	8.370115	0.005913	8.398952	0.001611	TRUE
N	L	20	118.8096	0.096468	118.4473	0.210253	119.9264	0.057282	TRUE
HN	V	21	9.014388	0.004217	8.946845	0.009191	8.906372	0.002504	TRUE
N	V	21	127.2279	0.04965	127.7215	0.108213	128.2183	0.029482	TRUE
HN	I	22	9.343599	0.001241	9.362462	0.002704	9.32651	0.000737	TRUE
N	I	22	126.5617	0.022065	126.289	0.048092	126.0814	0.013102	TRUE
HN	V	23	8.865944	0.005593	8.742316	0.01219	8.71058	0.003321	TRUE
N	V	23	124.3597	0.021177	123.9086	0.046156	123.7687	0.012575	TRUE
HN	D	24	7.503646	0.003168	7.391434	0.006905	7.403745	0.001881	TRUE
N	D	24	123.4743	0.026916	122.0373	0.058664	122.1274	0.015983	TRUE
HN	F	25	9.109198	0.003071	9.196655	0.006693	9.126163	0.001823	TRUE
N	F	25	126.6864	0.005105	126.315	0.011126	126.5251	0.003031	TRUE
HN	F	26	8.776583	0.004185	8.712295	0.009122	8.699133	0.002485	TRUE

N	F	26	121.14	0.026479	120.9003	0.057712	120.905	0.015723	TRUE
HN	A	27	7.248039	0.009744	7.352271	0.021237	7.438549	0.005786	TRUE
N	A	27	120.3158	0.031501	120.1978	0.068656	120.2868	0.018705	TRUE
HN	E	28	9.639032	0.002042	9.668768	0.004452	9.664046	0.001213	TRUE
N	E	28	124.5603	0.054303	125.6344	0.118354	125.5103	0.032245	TRUE
HN	W	29	6.568044	0.001915	6.478047	0.004174	6.5145	0.001137	TRUE
N	W	29	110.2708	0.01995	110.3482	0.043481	110.7615	0.011846	TRUE
HN	C	30	6.615406	0.009018	6.353256	0.019655	6.354368	0.005355	TRUE
N	C	30	123.863	0.033537	123.9871	0.073094	123.511	0.019914	TRUE
HN	K	34	7.832953	0.003028	7.75243	0.006599	7.734889	0.001798	TRUE
N	K	34	120.7593	0.032178	121.4841	0.070132	121.6252	0.019107	TRUE
HN	R	35	8.167781	0.002732	8.066346	0.005953	8.06185	0.001622	TRUE
N	R	35	118.445	0.027314	118.1785	0.059531	117.8069	0.016219	TRUE
HN	I	36	7.505881	0.007349	7.354801	0.016017	7.271382	0.004364	TRUE
N	I	36	115.1078	0.007745	115.2211	0.016879	115.1011	0.004599	TRUE
HN	A	37	7.306518	0.000999	7.256808	0.002177	7.304208	0.000593	TRUE
N	A	37	126.0375	0.019838	125.7651	0.043237	125.6408	0.01178	TRUE
HN	F	39	7.669786	0.001624	7.608628	0.00354	7.673329	0.000964	TRUE
N	F	39	120.54	0.011624	120.8134	0.025334	121.0066	0.006902	TRUE
HN	Y	40	8.592675	0.001295	8.500535	0.002823	8.568918	0.000769	TRUE
N	Y	40	122.745	0.021645	122.2478	0.047176	122.2148	0.012853	TRUE
HN	E	41	8.055832	0.001166	8.004199	0.002542	8.043412	0.000693	TRUE
N	E	41	116.6163	0.007026	116.5721	0.015314	116.6631	0.004172	TRUE
HN	E	42	8.264486	0.000492	8.293046	0.001073	8.266442	0.000292	TRUE
N	E	42	122.4071	0.016709	122.9843	0.036417	122.6777	0.009921	TRUE
HN	C	43	8.301654	0.003833	8.220072	0.008354	8.216685	0.002276	TRUE
N	C	43	117.7851	0.013155	117.1231	0.028672	117.4495	0.007812	TRUE
HN	S	44	7.254766	0.003133	7.150498	0.006829	7.155377	0.00186	TRUE
N	S	44	118.5342	0.007541	118.2518	0.016435	118.4296	0.004478	TRUE
HN	K	45	6.667047	0.006489	6.499061	0.014143	6.489954	0.003853	TRUE
N	K	45	118.6371	0.004023	118.6878	0.008768	118.7547	0.002389	TRUE
HN	T	46	7.210083	0.00744	6.958814	0.016216	6.977371	0.004418	TRUE
N	T	46	114.3939	0.013326	114.1204	0.029044	114.0519	0.007913	TRUE
HN	Y	47	7.883661	0.00282	7.827983	0.006145	7.908533	0.001674	TRUE
N	Y	47	123.5835	0.00947	123.2941	0.02064	123.319	0.005623	TRUE
HN	T	48	7.228785	0.002231	7.109727	0.004862	7.16766	0.001325	TRUE
N	T	48	107.8093	0.008538	107.3831	0.018609	107.6209	0.00507	TRUE
HN	K	49	8.741593	0.000869	8.781462	0.001894	8.751752	0.000516	TRUE
N	K	49	120.7054	0.019723	120.9278	0.042987	121.0738	0.011711	TRUE
HN	M	50	7.833757	0.002762	7.74777	0.006021	7.759296	0.00164	TRUE
N	M	50	118.61	0.027929	119.3057	0.060872	119.5017	0.016584	TRUE
HN	V	51	8.176074	0.003703	8.228444	0.00807	8.240373	0.002199	TRUE
N	V	51	125.9355	0.062505	126.9357	0.136229	127.1703	0.037115	TRUE
HN	F	52	9.543637	0.00354	9.490312	0.007716	9.571554	0.002102	TRUE
N	F	52	129.0366	0.021182	128.8541	0.046166	128.4705	0.012578	TRUE
HN	I	53	9.034269	0.001952	8.920056	0.004255	9.022497	0.001159	TRUE
N	I	53	114.5102	0.031928	114.1172	0.069588	115.0106	0.018959	TRUE
HN	K	54	9.022057	0.000773	9.028247	0.001686	9.035789	0.000459	TRUE
N	K	54	122.97	0.020186	122.9238	0.043995	122.6217	0.011986	TRUE
HN	V	55	8.767648	0.004065	8.543275	0.008859	8.6561	0.002414	TRUE

N	V	55	121.3957	0.005286	120.9359	0.01152	121.2703	0.003139	TRUE
HN	D	56	9.087391	0.002983	9.222056	0.006502	9.164174	0.001772	TRUE
N	D	56	129.323	0.003613	129.2785	0.007874	129.2885	0.002145	TRUE
HN	V	57	8.59404	0.00672	8.516407	0.014647	8.45232	0.00399	TRUE
N	V	57	120.3199	0.017598	119.8909	0.038356	119.9646	0.01045	TRUE
HN	D	58	8.55916	0.013634	8.366693	0.029716	8.310021	0.008096	TRUE
HN	E	59	7.700399	0.00537	7.652085	0.011705	7.553603	0.003189	TRUE
HN	S	61	7.86299	0.003207	7.826953	0.006991	7.917947	0.001905	TRUE
N	S	61	118.7854	0.027404	118.6901	0.059726	118.7649	0.016272	TRUE
HN	E	62	9.66113	0.007077	9.533485	0.015423	9.485923	0.004202	TRUE
N	E	62	121.42	0.004741	121.2984	0.010332	121.4923	0.002815	TRUE
HN	V	63	7.181132	0.011044	6.931056	0.024071	6.89502	0.006558	TRUE
N	V	63	118.1633	0.028007	118.5042	0.061041	118.6738	0.01663	TRUE
HN	T	64	7.693974	0.006334	7.565154	0.013804	7.556281	0.003761	TRUE
N	T	64	117.1895	0.01506	117.0335	0.032822	116.7179	0.008942	TRUE
HN	E	65	7.821106	0.00077	7.817411	0.001678	7.800516	0.000457	TRUE
N	E	65	118.0514	0.013644	117.5074	0.029737	117.8622	0.008102	TRUE
HN	E	67	8.20968	0.001848	8.240737	0.004029	8.239154	0.001098	TRUE
N	E	67	113.8293	0.015792	113.8507	0.034419	113.3245	0.009377	TRUE
HN	N	68	7.649342	0.006535	7.497935	0.014244	7.473317	0.003881	TRUE
N	N	68	117.0185	0.029087	116.4597	0.063396	116.4581	0.017272	TRUE
HN	I	69	7.896809	0.002062	7.831551	0.004494	7.901556	0.001224	TRUE
N	I	69	118.6271	0.018593	118.0673	0.040525	118.1103	0.011041	TRUE
HN	T	70	8.723278	0.002286	8.686165	0.004982	8.731126	0.001357	TRUE
N	T	70	117.7214	0.016798	118.0475	0.036612	118.0067	0.009975	TRUE
HN	M	72	8.659179	0.004852	8.731216	0.010575	8.764014	0.002881	TRUE
N	M	72	120.687	0.015774	120.1757	0.03438	120.2487	0.009367	TRUE
HN	T	74	8.354035	0.004727	8.417078	0.010303	8.44403	0.002807	TRUE
N	T	74	117.8981	0.058363	118.8289	0.127204	119.1811	0.034656	TRUE
HN	F	75	9.657672	0.006907	9.672014	0.015055	9.433031	0.004102	TRUE
N	F	75	124.94	0.022326	124.57	0.04866	124.4037	0.013257	TRUE
HN	K	76	8.873277	0.00236	8.762221	0.005143	8.788052	0.001401	TRUE
N	K	76	119.9248	0.015104	119.81	0.032918	119.5718	0.008968	TRUE
HN	V	77	8.038174	0.011894	7.839235	0.025924	7.833025	0.007063	TRUE
N	V	77	120.902	0.013855	120.2644	0.030196	120.3371	0.008227	TRUE
HN	Y	78	9.880422	0.001465	9.819246	0.003194	9.832752	0.00087	TRUE
N	Y	78	128.2581	0.01387	127.5954	0.03023	127.883	0.008236	TRUE
HN	K	79	8.714902	0.004961	8.58309	0.010812	8.582796	0.002946	TRUE
N	K	79	121.9248	0.017112	122.0447	0.037295	121.4442	0.010161	TRUE
HN	N	80	10.15838	0.00506	10.1122	0.011027	10.03321	0.003004	TRUE
N	N	80	125.5678	0.034816	125.1305	0.075882	124.4655	0.020673	TRUE
HN	G	81	9.536653	0.004503	9.439868	0.009814	9.411632	0.002674	TRUE
N	G	81	104.8793	0.022191	104.5341	0.048366	104.4512	0.013177	TRUE
HN	S	82	7.658993	0.004336	7.494705	0.009451	7.527121	0.002575	TRUE
N	S	82	114.5487	0.009031	114.3198	0.019682	114.2984	0.005362	TRUE
HN	S	83	9.126374	0.001639	9.136972	0.003573	9.151965	0.000973	TRUE
N	S	83	120.2945	0.005608	119.9436	0.012222	120.1262	0.00333	TRUE
HN	V	84	8.964612	0.001316	8.918696	0.002869	8.912852	0.000782	TRUE
N	V	84	118.1446	0.003952	118.0537	0.008614	118.2101	0.002347	TRUE
HN	D	85	7.526055	0.005963	7.383988	0.012997	7.365298	0.003541	TRUE

N	D	85	122.0248	0.014641	122.147	0.03191	122.1029	0.008694	TRUE
HN	T	86	8.572872	0.00698	8.519042	0.015214	8.431619	0.004145	TRUE
N	T	86	116.2241	0.041259	116.3467	0.089924	116.9373	0.024499	TRUE
HN	L	87	9.757996	0.001655	9.791501	0.003607	9.785459	0.000983	TRUE
N	L	87	129.3184	0.010383	129.9469	0.022629	129.7684	0.006165	TRUE
HN	L	88	8.883862	0.007103	8.933367	0.015481	9.04474	0.004218	TRUE
N	L	88	128.2371	0.033311	128.2561	0.072601	128.9924	0.01978	TRUE
HN	G	89	8.06059	0.008226	8.052616	0.017929	8.202258	0.004885	TRUE
N	G	89	110.04	0.005367	110.1245	0.011698	109.8627	0.003187	TRUE
HN	A	90	8.436282	0.002423	8.366	0.005281	8.413776	0.001439	TRUE
N	A	90	120.2136	0.022569	120.1883	0.049189	119.7999	0.013401	TRUE
HN	N	91	7.61485	0.006671	7.617194	0.014538	7.450249	0.003961	TRUE
N	N	91	120.892	0.044518	121.2936	0.097028	119.7788	0.026434	TRUE
HN	D	92	8.859189	0.005249	8.664169	0.01144	8.680181	0.003117	TRUE
N	D	92	125.4411	0.017368	126.9108	0.037853	125.9174	0.010313	TRUE
HN	S	93	8.151477	0.004331	8.06443	0.009438	8.032851	0.002571	TRUE
N	S	93	115.3796	0.015236	115.2916	0.033206	115.0747	0.009047	TRUE
HN	A	94	7.911796	0.004975	7.966942	0.010843	8.02345	0.002954	TRUE
N	A	94	124.5766	0.008015	124.9431	0.017469	124.5811	0.004759	TRUE
HN	L	95	8.219826	0.006459	8.040149	0.014077	8.017692	0.003835	TRUE
N	L	95	121.692	0.005475	121.922	0.011934	121.8689	0.003251	TRUE
HN	K	96	8.373157	0.003105	8.410962	0.006767	8.420347	0.001844	TRUE
N	K	96	119.7552	0.006486	119.7076	0.014136	119.485	0.003851	TRUE
HN	Q	97	7.819316	0.00174	7.754535	0.003792	7.768104	0.001033	TRUE
N	Q	97	115.7827	0.0039	115.7607	0.008499	115.843	0.002316	TRUE
HN	L	98	7.860269	0.008602	7.673531	0.018749	7.650685	0.005108	TRUE
N	L	98	122.1208	0.006501	121.9863	0.01417	121.9552	0.00386	TRUE
HN	I	99	8.06459	0.009265	7.8527	0.020194	7.821085	0.005502	TRUE
N	I	99	117.0959	0.018137	116.315	0.03953	116.3873	0.01077	TRUE
HN	E	100	8.43551	0.008737	8.587959	0.019041	8.629668	0.005188	TRUE
N	E	100	117.3604	0.00578	117.4337	0.012598	117.2808	0.003432	TRUE
HN	K	101	7.633606	0.005566	7.462537	0.012131	7.448383	0.003305	TRUE
N	K	101	119.6152	0.010548	119.4743	0.02299	119.3771	0.006263	TRUE
HN	Y	102	7.070736	0.005865	6.828793	0.012783	6.900888	0.003483	TRUE
N	Y	102	112.8131	0.00725	112.2285	0.015801	112.682	0.004305	TRUE
HN	A	103	8.65926	0.002643	8.696348	0.005761	8.685548	0.00157	TRUE
N	A	103	120.8017	0.011846	120.6732	0.025818	120.5661	0.007034	TRUE
HN	A	104	7.528193	0.00451	7.648142	0.00983	7.635509	0.002678	TRUE
N	A	104	126.6267	0.012487	126.6039	0.027216	126.6491	0.007415	TRUE
