# Supplementary material for: Solution structure and pressure response of thioredoxin-1 of Plasmodium falciparum
Source: PLoS One. 2024 Apr 18;19(4):e0301579. doi: 10.1371/journal.pone.0301579 (PMC11025842; doi:10.1371/journal.pone.0301579)
Supplement: S4 File — (PDF) [file pone.0301579.s004.pdf]

- ***Pf*Trx – oxidized state**

**Transitions** i - j	K_{ij} (at 0.1 MPa)	ΔG^0_{ij} [kJ mol^{-1}]	$\Delta V^0{}_{ij}$ [mL mol^{-1}]	$\Delta\beta^{0'}{}_{ij}$ [mL MPa^{-1} mol^{-1}]
1 – 2		2.46 ± 0.19	-28.41 ± 0.99	-0.0540 ± 0.0088
1 – 3		5.67 ± 0.59	-60.6 ± 2.5	-0.016 ± 0.028

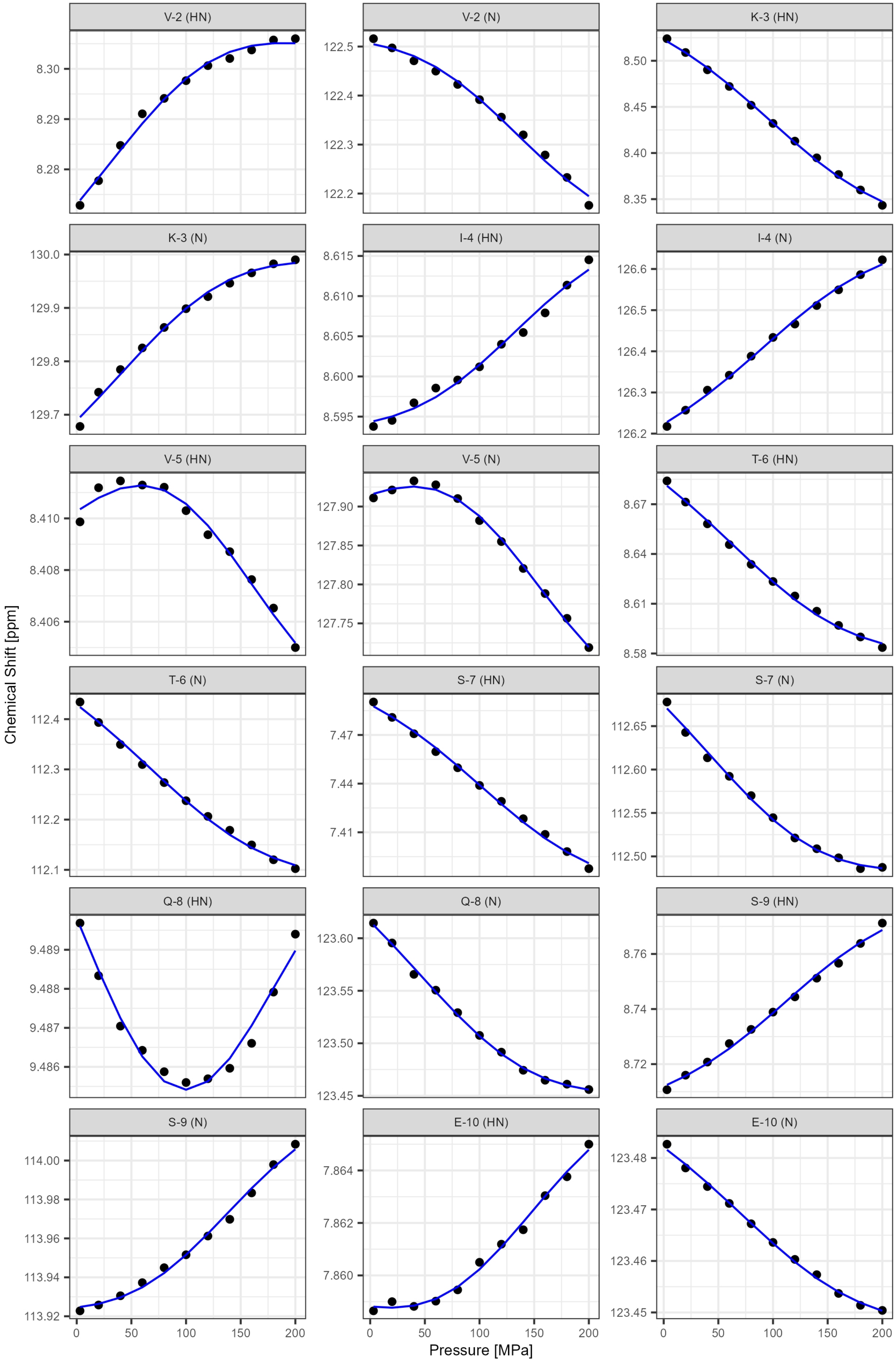

V-2 (HN)
V-2 (N)
K-3 (HN)
K-3 (N)
I-4 (HN)
I-4 (N)
V-5 (HN)
V-5 (N)
T-6 (HN)
T-6 (N)
S-7 (HN)
S-7 (N)
Q-8 (HN)
Q-8 (N)
S-9 (HN)
S-9 (N)
E-10 (HN)
E-10 (N)
Chemical Shift [ppm]
Pressure [MPa]
0
50
100
150
200

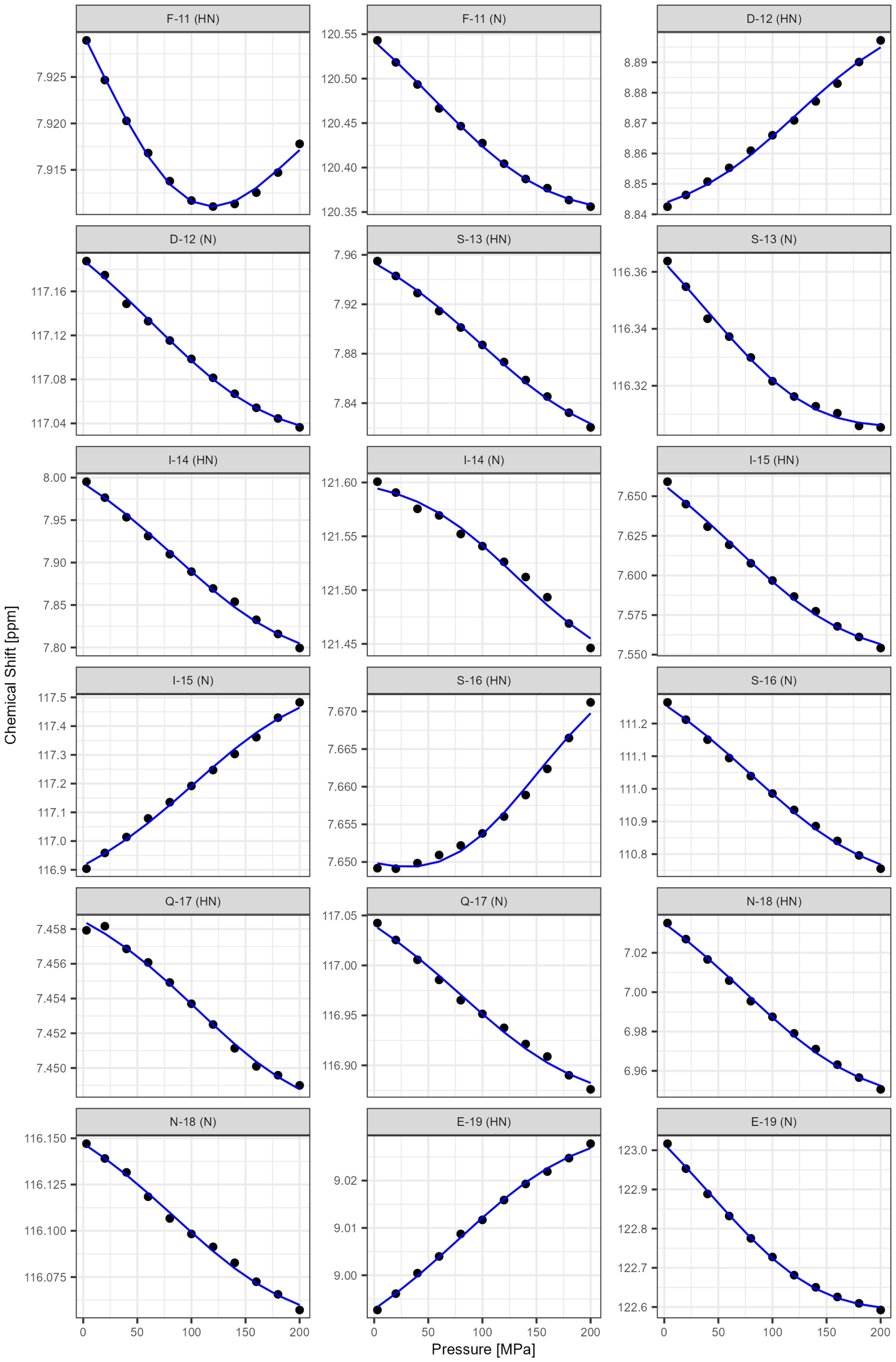

F-11 (HN)
F-11 (N)
D-12 (HN)
D-12 (N)
S-13 (HN)
S-13 (N)
I-14 (HN)
I-14 (N)
I-15 (HN)
I-15 (N)
S-16 (HN)
S-16 (N)
Q-17 (HN)
Q-17 (N)
N-18 (HN)
N-18 (N)
E-19 (HN)
E-19 (N)
Chemical Shift [ppm]
Pressure [MPa]

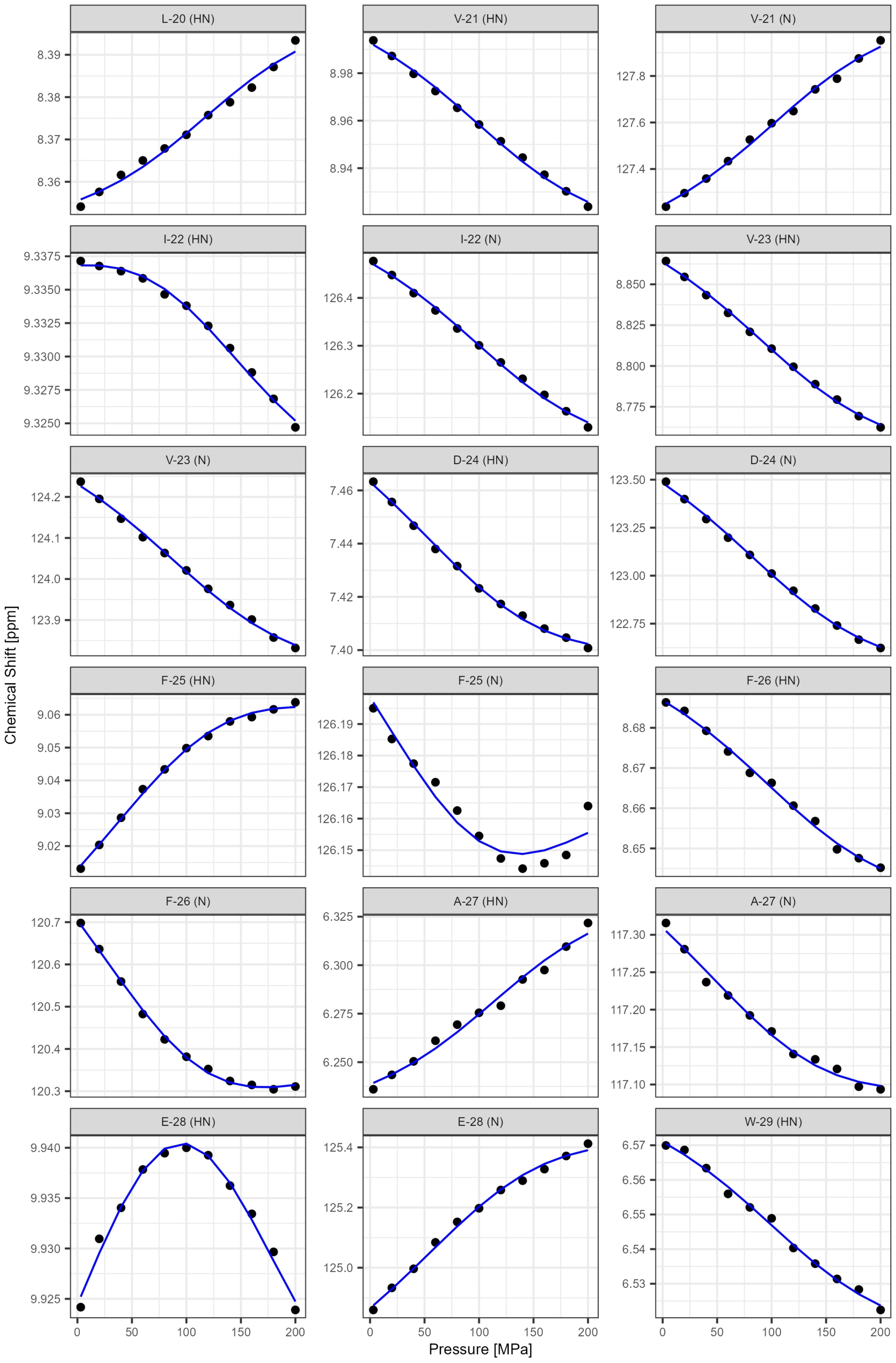
L-20 (HN)
V-21 (HN)
V-21 (N)
I-22 (HN)
I-22 (N)
V-23 (HN)
V-23 (N)
D-24 (HN)
D-24 (N)
F-25 (HN)
F-25 (N)
F-26 (HN)
F-26 (N)
A-27 (HN)
A-27 (N)
E-28 (HN)
E-28 (N)
W-29 (HN)
Chemical Shift [ppm]
Pressure [MPa]

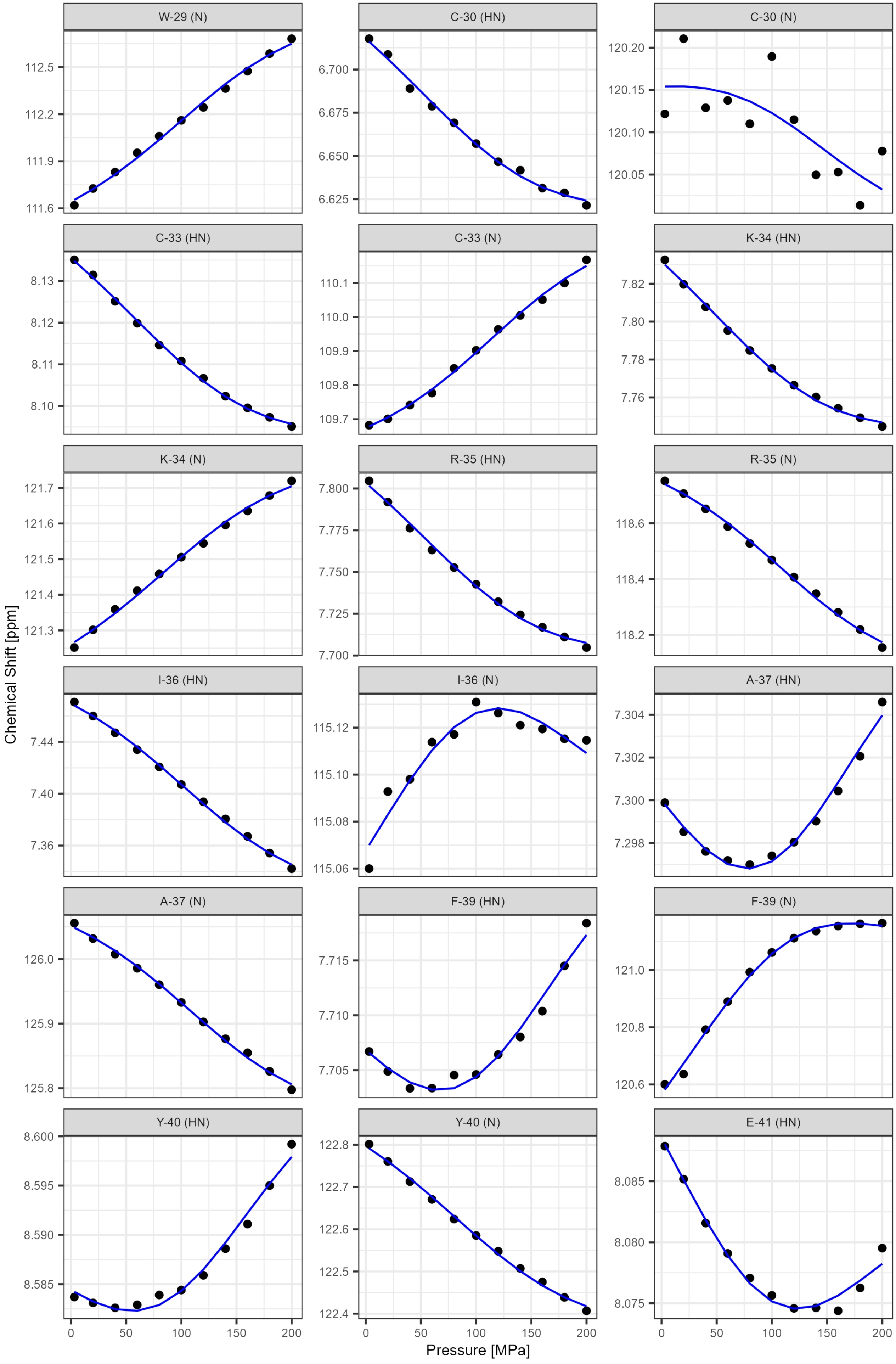

W-29 (N)
C-30 (HN)
C-30 (N)
C-33 (HN)
C-33 (N)
K-34 (HN)
K-34 (N)
R-35 (HN)
R-35 (N)
I-36 (HN)
I-36 (N)
A-37 (HN)
A-37 (N)
F-39 (HN)
F-39 (N)
Y-40 (HN)
Y-40 (N)
E-41 (HN)
Chemical Shift [ppm]
Pressure [MPa]

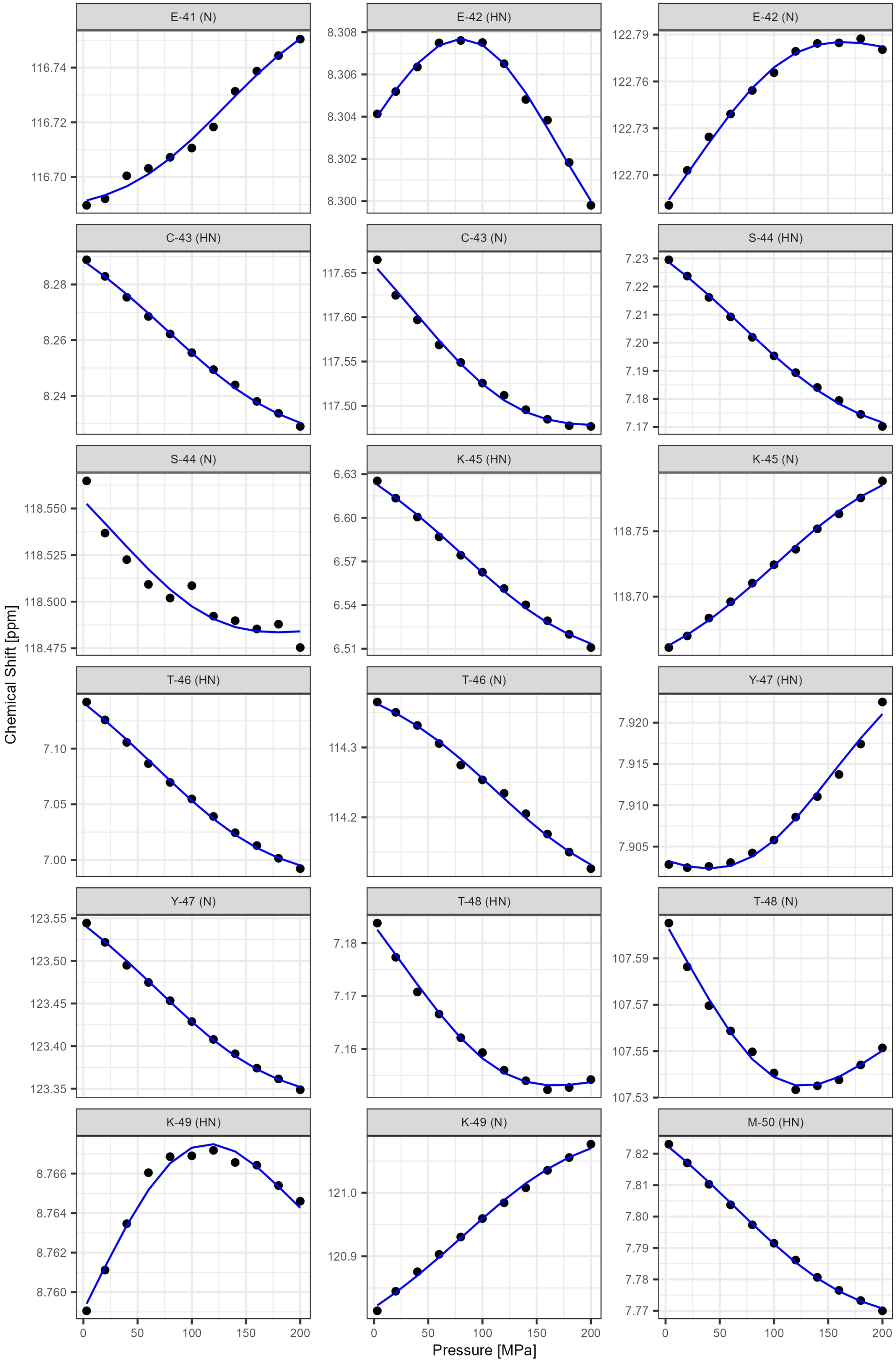
E-41 (N)
E-42 (HN)
E-42 (N)
C-43 (HN)
C-43 (N)
S-44 (HN)
S-44 (N)
K-45 (HN)
K-45 (N)
T-46 (HN)
T-46 (N)
Y-47 (HN)
Y-47 (N)
T-48 (HN)
T-48 (N)
K-49 (HN)
K-49 (N)
M-50 (HN)
Chemical Shift [ppm]
Pressure [MPa]

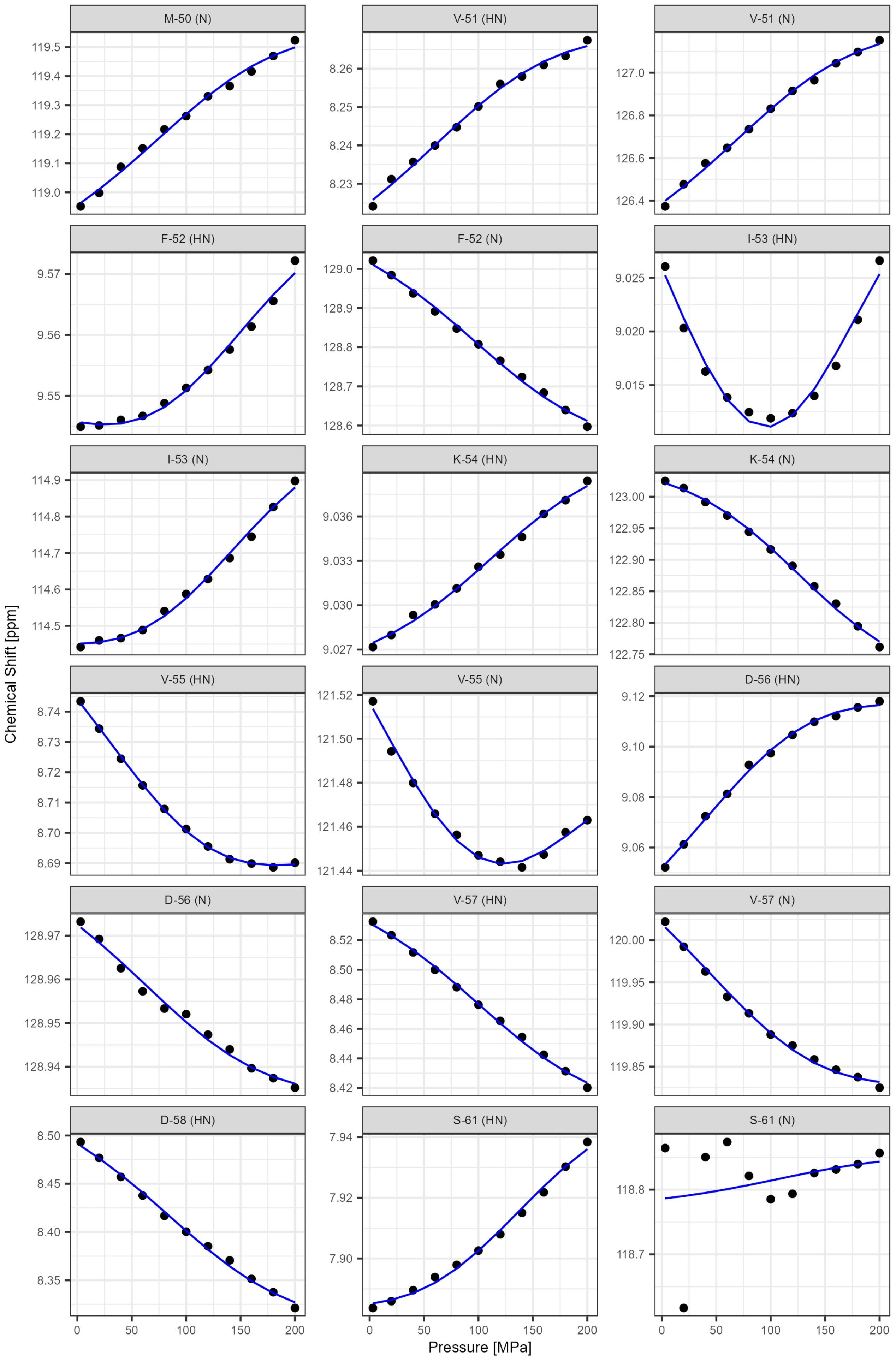

M-50 (N)
V-51 (HN)
V-51 (N)
F-52 (HN)
F-52 (N)
I-53 (HN)
I-53 (N)
K-54 (HN)
K-54 (N)
V-55 (HN)
V-55 (N)
D-56 (HN)
D-56 (N)
V-57 (HN)
V-57 (N)
D-58 (HN)
S-61 (HN)
S-61 (N)
Chemical Shift [ppm]
Pressure [MPa]

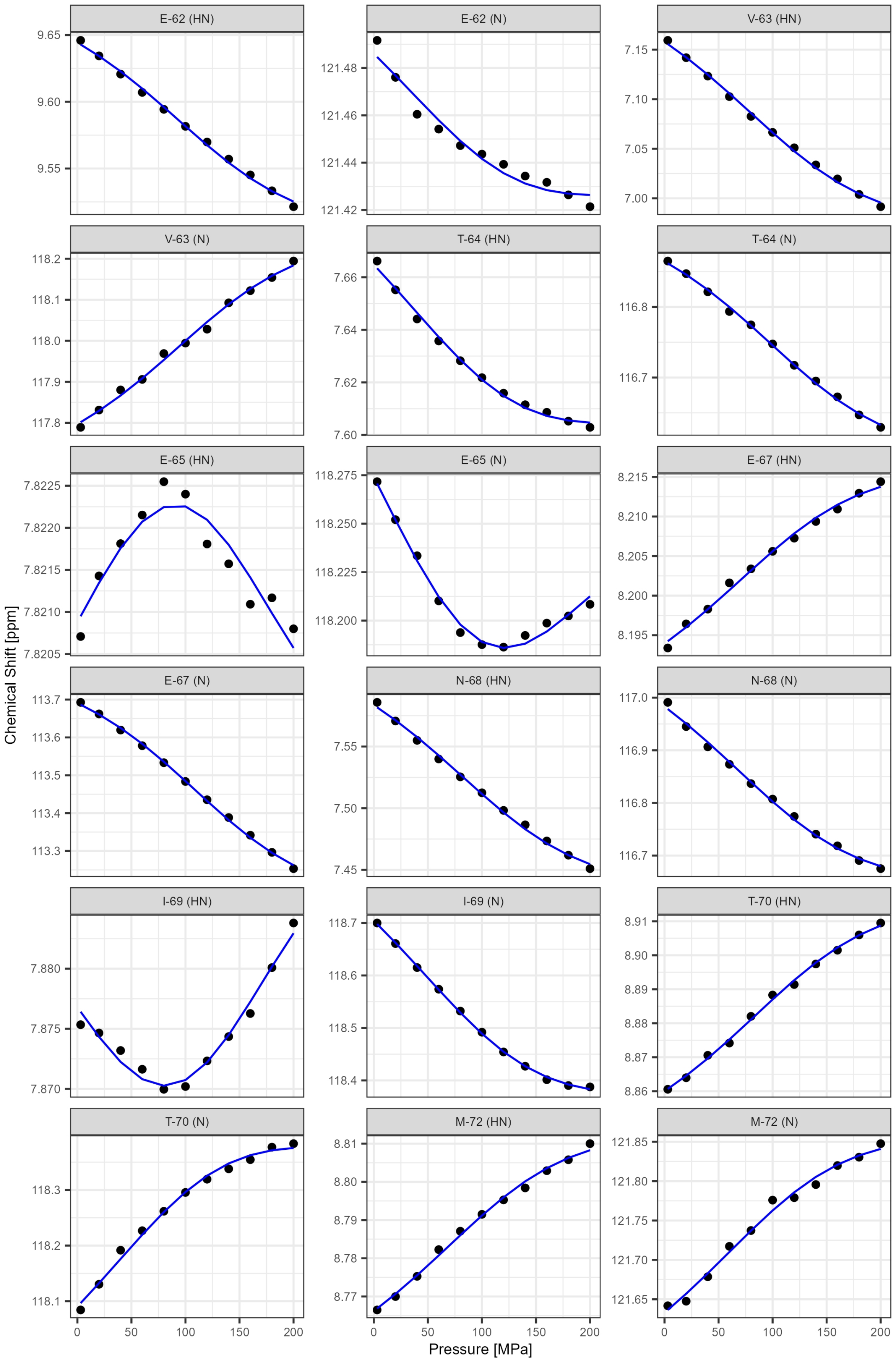
E-62 (HN)
E-62 (N)
V-63 (HN)
V-63 (N)
T-64 (HN)
T-64 (N)
E-65 (HN)
E-65 (N)
E-67 (HN)
E-67 (N)
N-68 (HN)
N-68 (N)
I-69 (HN)
I-69 (N)
T-70 (HN)
T-70 (N)
M-72 (HN)
M-72 (N)
Chemical Shift [ppm]
Pressure [MPa]

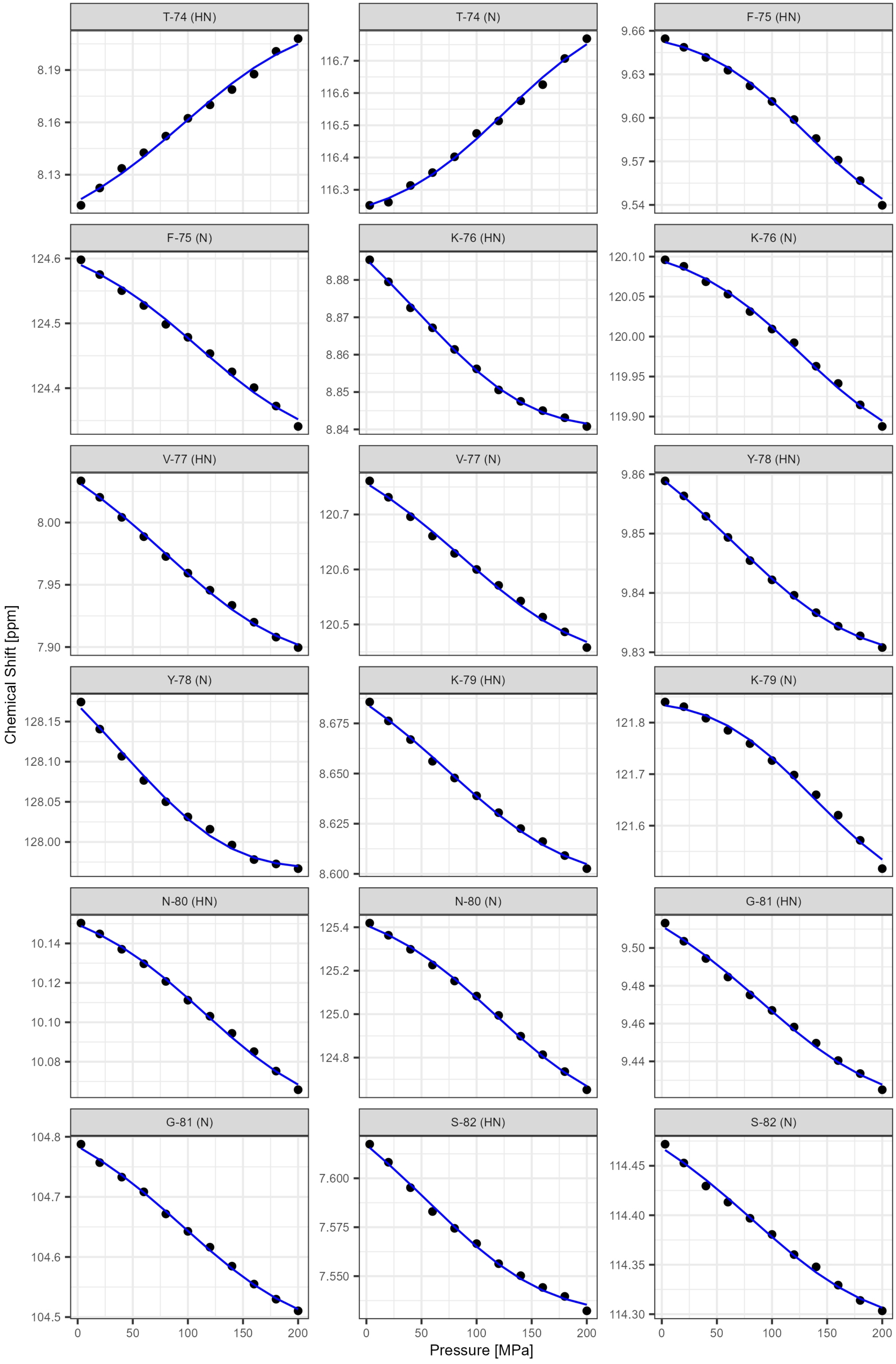

T-74 (HN)
T-74 (N)
F-75 (HN)
F-75 (N)
K-76 (HN)
K-76 (N)
V-77 (HN)
V-77 (N)
Y-78 (HN)
Y-78 (N)
K-79 (HN)
K-79 (N)
N-80 (HN)
N-80 (N)
G-81 (HN)
G-81 (N)
S-82 (HN)
S-82 (N)
Chemical Shift [ppm]
Pressure [MPa]
0
50
100
150
200

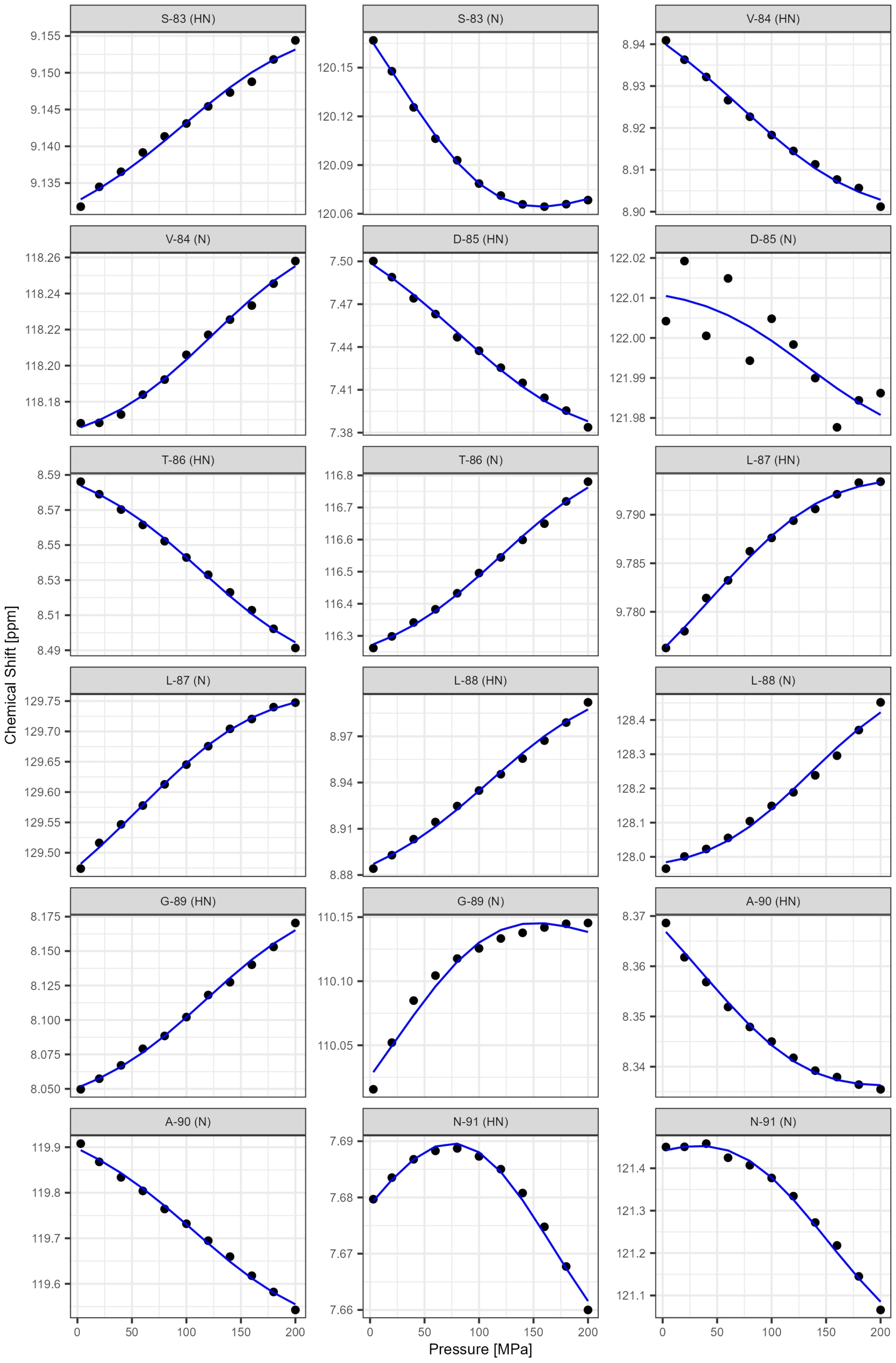
S-83 (HN)
S-83 (N)
V-84 (HN)
V-84 (N)
D-85 (HN)
D-85 (N)
T-86 (HN)
T-86 (N)
L-87 (HN)
L-87 (N)
L-88 (HN)
L-88 (N)
G-89 (HN)
G-89 (N)
A-90 (HN)
A-90 (N)
N-91 (HN)
N-91 (N)
Chemical Shift [ppm]
Pressure [MPa]

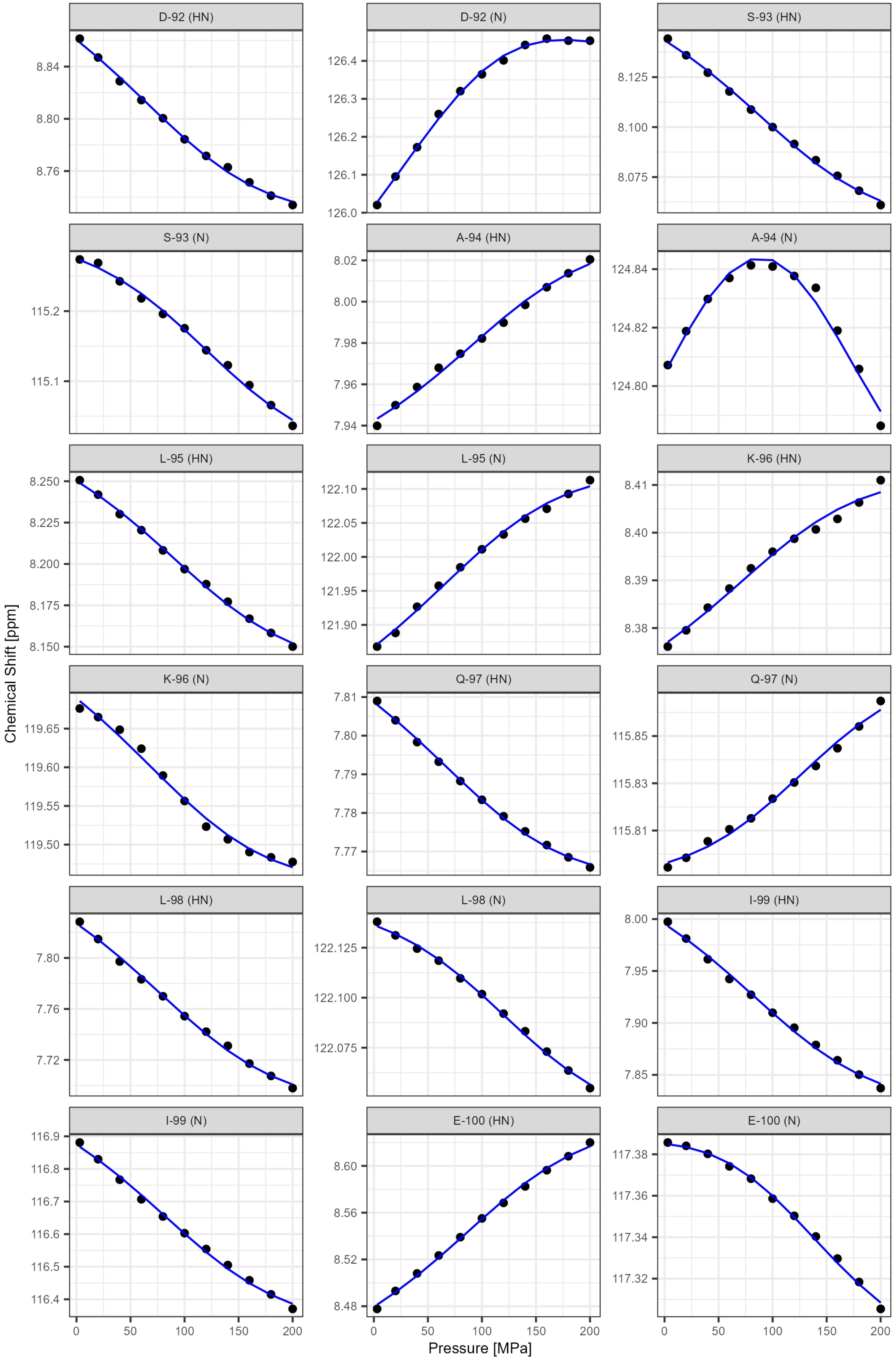

D-92 (HN)
D-92 (N)
S-93 (HN)
S-93 (N)
A-94 (HN)
A-94 (N)
L-95 (HN)
L-95 (N)
K-96 (HN)
K-96 (N)
Q-97 (HN)
Q-97 (N)
L-98 (HN)
L-98 (N)
I-99 (HN)
I-99 (N)
E-100 (HN)
E-100 (N)
Chemical Shift [ppm]
Pressure [MPa]

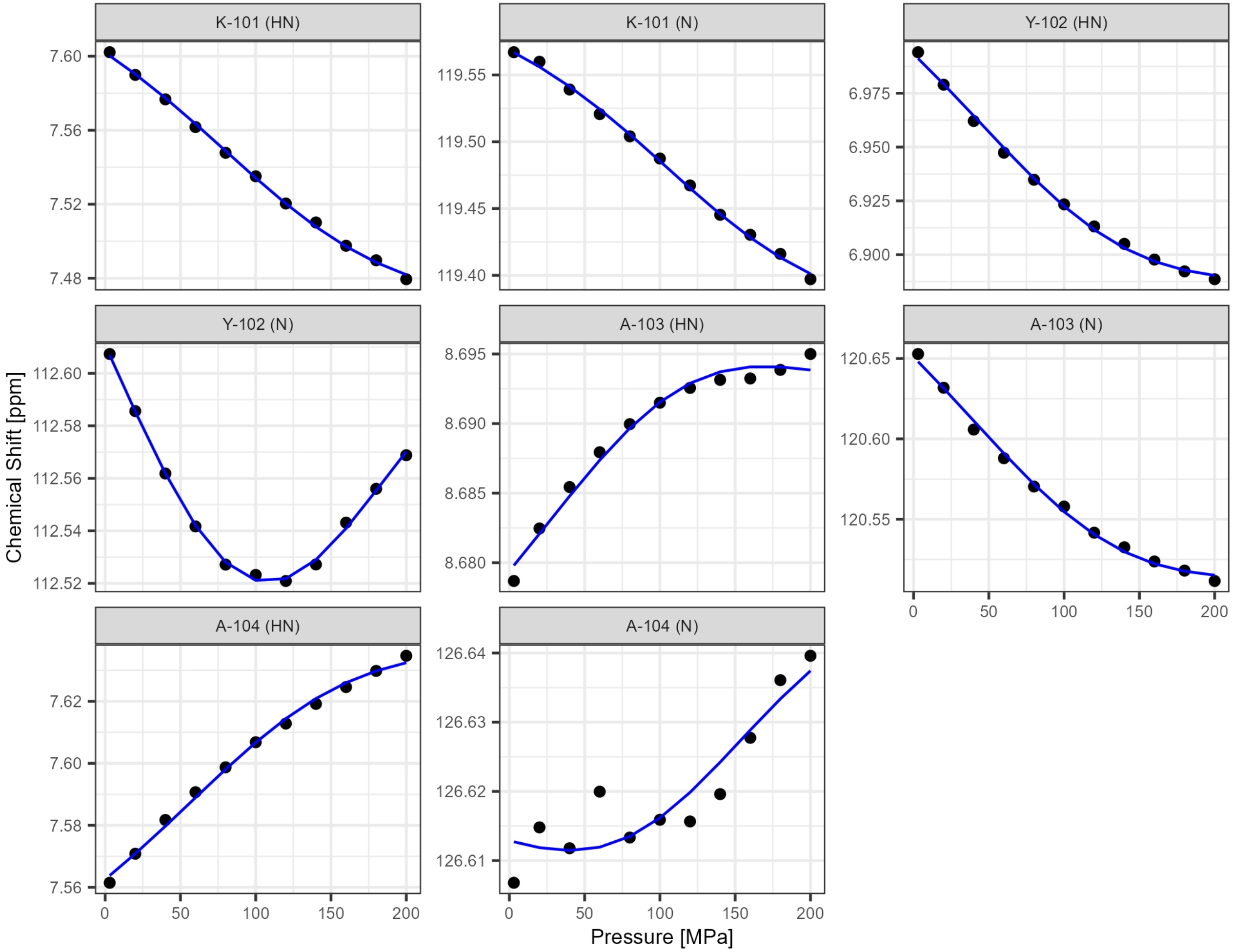

K-101 (HN)
K-101 (N)
Y-102 (HN)
Y-102 (N)
A-103 (HN)
A-103 (N)
A-104 (HN)
A-104 (N)
Chemical Shift [ppm]
Pressure [MPa]

nuc	res	res_nr	d1	d1err	d2	d2err	d3	d3err	converged
HN	V	2	8.243872	0.002913	8.345372	0.005609	8.302192	0.001715	TRUE
N	V	2	122.5072	0.028691	122.6147	0.055243	122.0876	0.016891	TRUE
HN	K	3	8.581766	0.006715	8.417575	0.012929	8.314701	0.003953	TRUE
N	K	3	129.4647	0.023937	130.2276	0.046089	129.9789	0.014092	TRUE
HN	I	4	8.593787	0.002305	8.589096	0.004438	8.619586	0.001357	TRUE
N	I	4	126.0785	0.022059	126.4998	0.042472	126.6762	0.012986	TRUE
HN	V	5	8.405624	0.000822	8.425445	0.001583	8.401197	0.000484	TRUE
N	V	5	127.8207	0.013015	128.2564	0.02506	127.6069	0.007662	TRUE
HN	T	6	8.735864	0.005404	8.564709	0.010405	8.578053	0.003181	TRUE
N	T	6	112.5945	0.018694	112.0688	0.035994	112.0777	0.011005	TRUE
HN	S	7	7.519473	0.005797	7.435496	0.011163	7.371857	0.003413	TRUE
N	S	7	112.8134	0.011834	112.3423	0.022786	112.4878	0.006967	TRUE
HN	Q	8	9.498716	0.000731	9.464532	0.001407	9.492948	0.00043	TRUE
N	Q	8	123.7268	0.00712	123.3545	0.013709	123.4536	0.004191	TRUE
HN	S	9	8.698691	0.004163	8.729578	0.008015	8.781984	0.002451	TRUE
N	S	9	113.9299	0.006605	113.8802	0.012717	114.0364	0.003888	TRUE
HN	E	10	7.860451	0.00054	7.852001	0.00104	7.867626	0.000318	TRUE
N	E	10	123.4976	0.001648	123.4489	0.003172	123.4468	0.00097	TRUE
HN	F	11	7.958949	0.000989	7.849829	0.001904	7.926854	0.000582	TRUE
N	F	11	120.6542	0.007745	120.2856	0.014913	120.3482	0.00456	TRUE
HN	D	12	8.836269	0.003884	8.846456	0.007479	8.909079	0.002287	TRUE
N	D	12	117.2688	0.006371	117.0115	0.012268	117.0245	0.003751	TRUE
HN	S	13	7.996112	0.006039	7.877625	0.011629	7.798844	0.003555	TRUE
N	S	13	116.4081	0.003646	116.2551	0.007021	116.3079	0.002147	TRUE
HN	I	14	8.070493	0.010423	7.83934	0.020069	7.776462	0.006136	TRUE
N	I	14	121.6009	0.016334	121.6285	0.03145	121.4094	0.009616	TRUE
HN	I	15	7.710338	0.006102	7.539606	0.011748	7.547503	0.003592	TRUE
N	I	15	116.7468	0.037485	117.2005	0.072175	117.5739	0.022068	TRUE
HN	S	16	7.657411	0.002316	7.62125	0.004459	7.68019	0.001363	TRUE
N	S	16	111.4687	0.025116	110.8317	0.048359	110.6991	0.014786	TRUE
HN	Q	17	7.461196	0.000694	7.454022	0.001336	7.446748	0.000408	TRUE
N	Q	17	117.1083	0.012753	116.9002	0.024556	116.8607	0.007508	TRUE
HN	N	18	7.074063	0.003809	6.952238	0.007335	6.942605	0.002243	TRUE
N	N	18	116.1827	0.005737	116.0764	0.011046	116.0468	0.003377	TRUE
HN	E	19	8.977608	0.001593	9.024269	0.003067	9.031369	0.000938	TRUE
N	E	19	123.3213	0.014713	122.2858	0.02833	122.6015	0.008662	TRUE
HN	L	20	8.348658	0.004137	8.362599	0.007965	8.399653	0.002435	TRUE
HN	V	21	9.014809	0.003879	8.953095	0.007468	8.913245	0.002283	TRUE
N	V	21	127.0327	0.04804	127.6023	0.092499	128.0613	0.028282	TRUE
HN	I	22	9.33434	0.000824	9.348082	0.001587	9.319999	0.000485	TRUE
N	I	22	126.5869	0.017397	126.2692	0.033496	126.0784	0.010242	TRUE
HN	V	23	8.901269	0.003892	8.790658	0.007494	8.747496	0.002291	TRUE
N	V	23	124.3869	0.020975	123.9247	0.040385	123.7779	0.012348	TRUE
HN	D	24	7.501206	0.002755	7.375723	0.005305	7.399479	0.001622	TRUE
N	D	24	123.8442	0.033864	122.7476	0.065203	122.5057	0.019936	TRUE
HN	F	25	8.972276	0.002497	9.11188	0.004807	9.060022	0.00147	TRUE
N	F	25	126.2639	0.012158	126.0262	0.023409	126.1719	0.007157	TRUE
HN	F	26	8.701187	0.002759	8.660604	0.005312	8.637385	0.001624	TRUE
N	F	26	121.1041	0.016869	119.6951	0.03248	120.3717	0.009931	TRUE

HN	A	27	6.221205	0.010185	6.260649	0.019611	6.334803	0.005996	TRUE
N	A	27	117.4547	0.021561	116.9673	0.041515	117.0947	0.012693	TRUE
HN	E	28	9.889938	0.002114	10.02341	0.00407	9.908353	0.001245	TRUE
N	E	28	124.5457	0.039302	125.5949	0.075674	125.4194	0.023138	TRUE
HN	W	29	6.586174	0.003532	6.544398	0.0068	6.514625	0.002079	TRUE
N	W	29	111.3121	0.073439	112.227	0.141402	112.8395	0.043234	TRUE
HN	C	30	6.776909	0.007005	6.58156	0.013488	6.620117	0.004124	TRUE
N	C	30	120.1259	0.111215	120.2786	0.214137	119.9768	0.065473	TRUE
HN	C	33	8.158868	0.001504	8.082656	0.002896	8.093078	0.000885	TRUE
N	C	33	109.5685	0.030118	109.8083	0.057991	110.2636	0.017731	TRUE
HN	K	34	7.88851	0.004255	7.698952	0.008193	7.744652	0.002505	TRUE
N	K	34	121.0772	0.030474	121.6281	0.058675	121.7703	0.01794	TRUE
HN	R	35	7.862533	0.005832	7.667678	0.011229	7.702746	0.003433	TRUE
N	R	35	118.8962	0.031701	118.519	0.061039	118.0473	0.018663	TRUE
HN	I	36	7.507238	0.005739	7.405591	0.011051	7.320441	0.003379	TRUE
N	I	36	114.9729	0.016031	115.325	0.030867	115.0782	0.009438	TRUE
HN	A	37	7.30937	0.000896	7.271681	0.001725	7.309821	0.000527	TRUE
N	A	37	126.1167	0.013802	125.9512	0.026574	125.7524	0.008125	TRUE
HN	F	39	7.720436	0.002101	7.664033	0.004046	7.727431	0.001237	TRUE
N	F	39	119.9599	0.04302	122.0939	0.082831	121.068	0.025326	TRUE
HN	Y	40	8.595279	0.002103	8.548369	0.004049	8.607774	0.001238	TRUE
N	Y	40	122.9596	0.020654	122.4651	0.039769	122.3643	0.012159	TRUE
HN	E	41	8.109562	0.001855	8.03188	0.003572	8.084724	0.001092	TRUE
N	E	41	116.6887	0.006369	116.6768	0.012264	116.7699	0.00375	TRUE
HN	E	42	8.292962	0.000563	8.336238	0.001085	8.293531	0.000332	TRUE
N	E	42	122.5699	0.007015	122.9649	0.013507	122.7639	0.00413	TRUE
HN	C	43	8.314527	0.002545	8.23444	0.004899	8.222679	0.001498	TRUE
N	C	43	117.8087	0.014233	117.2926	0.027405	117.488	0.008379	TRUE
HN	S	44	7.257966	0.00237	7.168609	0.004563	7.165185	0.001395	TRUE
N	S	44	118.6225	0.021293	118.3835	0.040998	118.4924	0.012535	TRUE
HN	K	45	6.671647	0.005399	6.527663	0.010395	6.497973	0.003178	TRUE
N	K	45	118.6232	0.005319	118.7261	0.010241	118.81	0.003131	TRUE
HN	T	46	7.217074	0.006546	6.975374	0.012605	6.98098	0.003854	TRUE
N	T	46	114.4183	0.013767	114.2938	0.026507	114.0774	0.008105	TRUE
HN	Y	47	7.912112	0.001988	7.871858	0.003829	7.931335	0.001171	TRUE
N	Y	47	123.641	0.007615	123.3296	0.014663	123.3329	0.004483	TRUE
HN	T	48	7.214789	0.002325	7.103788	0.004478	7.1585	0.001369	TRUE
N	T	48	107.7062	0.005733	107.3347	0.011038	107.5795	0.003375	TRUE
HN	K	49	8.745268	0.001198	8.796895	0.002306	8.759452	0.000705	TRUE
N	K	49	120.7119	0.014403	121.0369	0.027732	121.1056	0.008479	TRUE
HN	M	50	7.850753	0.001619	7.761791	0.003117	7.766033	0.000953	TRUE
N	M	50	118.7006	0.044559	119.4925	0.085795	119.5648	0.026232	TRUE
HN	V	51	8.202432	0.003117	8.275724	0.006002	8.26916	0.001835	TRUE
N	V	51	126.0165	0.042901	127.1871	0.082604	127.2153	0.025256	TRUE
HN	F	52	9.553732	0.002732	9.513897	0.00526	9.582457	0.001608	TRUE
N	F	52	129.1507	0.026247	128.7709	0.050537	128.5371	0.015452	TRUE
HN	I	53	9.057763	0.002428	8.934825	0.004675	9.040355	0.001429	TRUE
N	I	53	114.5174	0.033743	114.1061	0.064971	115.0595	0.019865	TRUE
HN	K	54	9.024855	0.000739	9.030709	0.001423	9.040567	0.000435	TRUE
N	K	54	123.0514	0.014232	123.0334	0.027403	122.6962	0.008378	TRUE

HN	V	55	8.795985	0.001551	8.614654	0.002987	8.695559	0.000913	TRUE
N	V	55	121.6273	0.006551	121.2171	0.012614	121.4975	0.003857	TRUE
HN	D	56	9.000935	0.003429	9.174917	0.006602	9.114488	0.002019	TRUE
N	D	56	128.9923	0.00389	128.9286	0.007491	128.933	0.00229	TRUE
HN	V	57	8.565298	0.005809	8.472902	0.011185	8.40239	0.00342	TRUE
N	V	57	120.1532	0.012915	119.7016	0.024867	119.831	0.007603	TRUE
HN	D	58	8.558454	0.010741	8.358034	0.020681	8.302263	0.006323	TRUE
HN	S	61	7.887158	0.004269	7.860724	0.00822	7.954637	0.002513	TRUE
N	S	61	118.7701	0.195773	118.8105	0.376948	118.8558	0.115253	TRUE
HN	E	62	9.68683	0.006947	9.564964	0.013375	9.504424	0.00409	TRUE
N	E	62	121.5356	0.012131	121.365	0.023357	121.4295	0.007142	TRUE
HN	V	63	7.229331	0.008467	7.010926	0.016303	6.973735	0.004985	TRUE
N	V	63	117.66	0.030125	118.0513	0.058005	118.252	0.017735	TRUE
HN	T	64	7.71254	0.004467	7.548948	0.0086	7.606837	0.00263	TRUE
N	T	64	116.9406	0.00959	116.729	0.018465	116.5891	0.005646	TRUE
HN	E	65	7.817644	0.000647	7.830276	0.001246	7.818921	0.000381	TRUE
N	E	65	118.4106	0.008424	117.9039	0.016221	118.2566	0.00496	TRUE
HN	E	67	8.18418	0.001602	8.214852	0.003084	8.21594	0.000943	TRUE
N	E	67	113.8016	0.015272	113.5259	0.029405	113.1675	0.008991	TRUE
HN	N	68	7.638614	0.007536	7.47153	0.014511	7.436452	0.004437	TRUE
N	N	68	117.1368	0.018788	116.65	0.036175	116.6486	0.011061	TRUE
HN	I	69	7.894816	0.002013	7.822825	0.003876	7.893687	0.001185	TRUE
N	I	69	118.9191	0.008179	118.2018	0.015749	118.3751	0.004815	TRUE
HN	T	70	8.83973	0.002545	8.900829	0.004901	8.915867	0.001498	TRUE
N	T	70	117.8669	0.024849	118.6297	0.047846	118.3673	0.014629	TRUE
HN	M	72	8.744999	0.003047	8.811989	0.005866	8.812672	0.001794	TRUE
N	M	72	121.5145	0.021364	121.8986	0.041135	121.8561	0.012577	TRUE
HN	T	74	8.084574	0.007733	8.169956	0.014889	8.221417	0.004552	TRUE
N	T	74	116.1917	0.035683	116.2352	0.068705	116.8971	0.021007	TRUE
HN	F	75	9.656209	0.006733	9.681253	0.012965	9.508373	0.003964	TRUE
N	F	75	124.6529	0.018667	124.5042	0.035943	124.298	0.01099	TRUE
HN	K	76	8.915606	0.001564	8.814712	0.003011	8.840773	0.00092	TRUE
N	K	76	120.1169	0.012286	120.1025	0.023656	119.8362	0.007233	TRUE
HN	V	77	8.089913	0.005934	7.915136	0.011425	7.883983	0.003493	TRUE
N	V	77	120.8717	0.018438	120.53	0.035502	120.4234	0.010855	TRUE
HN	Y	78	9.873862	0.000736	9.827341	0.001417	9.828545	0.000433	TRUE
N	Y	78	128.3217	0.014365	127.8092	0.027659	127.9726	0.008457	TRUE
HN	K	79	8.722072	0.004131	8.606084	0.007955	8.595073	0.002432	TRUE
N	K	79	121.8205	0.027414	121.9809	0.052783	121.4242	0.016139	TRUE
HN	N	80	10.16698	0.004363	10.12881	0.0084	10.04866	0.002568	TRUE
N	N	80	125.5529	0.03291	125.2612	0.063366	124.482	0.019374	TRUE
HN	G	81	9.543483	0.005066	9.449884	0.009753	9.41401	0.002982	TRUE
N	G	81	104.8763	0.011305	104.6157	0.021767	104.4637	0.006655	TRUE
HN	S	82	7.665759	0.005316	7.506449	0.010236	7.530482	0.00313	TRUE
N	S	82	114.5367	0.010947	114.329	0.021078	114.2835	0.006445	TRUE
HN	S	83	9.125517	0.002208	9.145312	0.00425	9.15692	0.0013	TRUE
N	S	83	120.2846	0.003524	119.8685	0.006785	120.0908	0.002074	TRUE
HN	V	84	8.958854	0.002457	8.901199	0.004732	8.898649	0.001447	TRUE
N	V	84	118.1531	0.006734	118.1675	0.012967	118.2805	0.003965	TRUE
HN	D	85	7.54717	0.007031	7.400949	0.013537	7.372504	0.004139	TRUE

N	D	85	122.0118	0.020543	122.0181	0.039554	121.971	0.012094	TRUE
HN	T	86	8.60438	0.005374	8.560353	0.010347	8.472755	0.003163	TRUE
N	T	86	116.1844	0.030867	116.3377	0.059432	116.8916	0.018171	TRUE
HN	L	87	9.764199	0.001082	9.804101	0.002083	9.793612	0.000637	TRUE
N	L	87	129.3196	0.011284	129.832	0.021728	129.7661	0.006643	TRUE
HN	L	88	8.860425	0.007654	8.923654	0.014737	9.010132	0.004506	TRUE
N	L	88	127.9871	0.047765	127.8112	0.091969	128.5764	0.02812	TRUE
HN	G	89	8.029544	0.007695	8.070902	0.014816	8.194483	0.00453	TRUE
N	G	89	109.8866	0.021743	110.3835	0.041866	110.111	0.012801	TRUE
HN	A	90	8.393602	0.002553	8.303927	0.004915	8.337982	0.001503	TRUE
N	A	90	119.9899	0.024715	119.7526	0.047587	119.4813	0.01455	TRUE
HN	N	91	7.644872	0.002575	7.781909	0.004958	7.639524	0.001516	TRUE
N	N	91	121.2976	0.032301	121.9803	0.062193	120.8939	0.019016	TRUE
HN	D	92	8.927018	0.006204	8.713923	0.011946	8.725527	0.003652	TRUE
N	D	92	125.5834	0.019713	127.1024	0.037957	126.3944	0.011605	TRUE
HN	S	93	8.174641	0.003772	8.082597	0.007263	8.050255	0.002221	TRUE
N	S	93	115.3142	0.015219	115.2452	0.029304	114.9836	0.00896	TRUE
HN	A	94	7.91347	0.005829	7.998186	0.011224	8.030736	0.003432	TRUE
N	A	94	124.7098	0.007626	125.0805	0.014683	124.7416	0.004489	TRUE
HN	L	95	8.287325	0.00406	8.178714	0.007817	8.135942	0.00239	TRUE
N	L	95	121.7426	0.015443	122.1412	0.029734	122.126	0.009091	TRUE
HN	K	96	8.360776	0.003705	8.410517	0.007134	8.411954	0.002181	TRUE
N	K	96	119.8014	0.020842	119.4452	0.040129	119.4488	0.01227	TRUE
HN	Q	97	7.830732	0.001724	7.760411	0.003319	7.762721	0.001015	TRUE
N	Q	97	115.7895	0.005913	115.7917	0.011384	115.8805	0.003481	TRUE
HN	L	98	7.884414	0.006747	7.707145	0.01299	7.684661	0.003972	TRUE
N	L	98	122.1495	0.003915	122.127	0.007537	122.0354	0.002305	TRUE
HN	I	99	8.060371	0.009062	7.863466	0.017449	7.819461	0.005335	TRUE
N	I	99	117.084	0.029194	116.4484	0.056212	116.3176	0.017187	TRUE
HN	E	100	8.424494	0.006438	8.588402	0.012396	8.637777	0.00379	TRUE
N	E	100	117.3792	0.00444	117.4292	0.00855	117.2792	0.002614	TRUE
HN	K	101	7.654661	0.004273	7.493419	0.008227	7.465695	0.002515	TRUE
N	K	101	119.6174	0.007702	119.4883	0.01483	119.3667	0.004534	TRUE
HN	Y	102	7.066038	0.005052	6.820941	0.009727	6.889654	0.002974	TRUE
N	Y	102	112.7737	0.003687	112.1573	0.007099	112.6341	0.002171	TRUE
HN	A	103	8.664383	0.001982	8.717376	0.003816	8.691653	0.001167	TRUE
N	A	103	120.7534	0.009018	120.4052	0.017364	120.5174	0.005309	TRUE
HN	A	104	7.521648	0.004594	7.655176	0.008845	7.636967	0.002704	TRUE
N	A	104	126.6251	0.011511	126.5691	0.022163	126.6517	0.006776	TRUE
