# Supplementary material for: Solution structure and pressure response of thioredoxin-1 of Plasmodium falciparum
Source: PLoS One. 2024 Apr 18;19(4):e0301579. doi: 10.1371/journal.pone.0301579 (PMC11025842; doi:10.1371/journal.pone.0301579)
Supplement: Attachment — Submitted filename: PONE-D-23-42130_reviewer.docx [file pone.0301579.s005.docx]

In this manuscript by Munte et al., the authors described the structural characterization and pressure induced response of Thioredoxin-1 (reduced and oxidized state) of Plasmodium falciparum using solution NMR techniques. Both reduced and oxidized state of Thioredoxin-1 showed common structural fold with minor structural differences. They evaluated the pressure induced response of Thioredoxin-1 in its reduced and oxidized state in a pressure range of 0.1MPa to 200 MPa. This manuscript is well written. I recommend this article for the publication in “PLOS ONE” with minor revisions. The following corrections have been suggested:

1. There are some typographical errors in the manuscript and should be corrected.
2. Keywords should be in alphabetical order.
3. Sentence “These three states are sub-states from the two main states of the protein, the reduced and oxidized states Sr and So” (line 42-43) should be modified.
4. The typo error in the sentence “Because of the required additivity of the chemical shifts the Euclidean distance has been used in the]se calculations.” should be corrected (line 194).
5. Sentence “The Ramachandran Plot resulted in additional allowed regions, 0.9% in generously allowed regions, and 0.0% in disallowed regions” should be modified to include information on most favorable regions (line 313-315).
