# Supplementary material for: Solution structure and pressure response of thioredoxin-1 of Plasmodium falciparum
Source: PLoS One. 2024 Apr 18;19(4):e0301579. doi: 10.1371/journal.pone.0301579 (PMC11025842; doi:10.1371/journal.pone.0301579)
Supplement: Attachment — Submitted filename: Response_to_reviewers-3.docx [file pone.0301579.s006.docx]

Dear Dr. Ravi Pratap Barnwal,

We have corrected the manuscript according to your and the reviewer’s suggestions. We added all necessary experimental conditions. Especially, a complete chemical shift list has already been published and has been stored in the BioMagResBank, accession numbers 6442 and 16147. We also corrected the funding information and removed it from Acknowledgements. Please add to the funding information the sentence "The funders had no role in study design, data collection and analysis, decision to publish, or preparation of the manuscript". We also gave the full name of DFG, namely Deutsche Forschungsgemeinschaft.

**Reviewer 1**

*1. There are some typographical errors in the manuscript and should be corrected.*

We screened the text for typographical errors.

*2. Keywords should be in alphabetical order.*

We ordered them as suggested.

*3. Sentence “These three states are sub-states from the two main states of the protein, the reduced and oxidized states Sr and So” (line 42-43) should be modified.*

We modified the sentence and replaced it by “These three conformational states were identified in the reduced as well as in the oxidized form of the protein, therefore, they represent sub-states of the two main oxidation states of *Pf*Trx-1.” We hope the meaning is now clearer

*4. The typo error in the sentence “Because of the required additivity of the chemical shifts the Euclidean distance has been used in the]se calculations.” should be corrected (line 194).*

We corrected this.

*5. Sentence “The Ramachandran Plot resulted in additional allowed regions, 0.9% in generously allowed regions, and 0.0% in disallowed regions” should be modified to include information on most favorable regions (line 313-315).*

We added this information.
